# Visible Light Photocatalysis of 6π Heterocyclization

**DOI:** 10.1002/anie.201705333

**Published:** 2017-07-04

**Authors:** Niels Münster, Nicholas A. Parker, Lucy van Dijk, Robert S. Paton, Martin D. Smith

**Affiliations:** ^1^ Chemistry Research Laboratory University of Oxford 12 Mansfield Road Oxford OX1 3TA UK

**Keywords:** catalysis, DFT calculations, energy transfer, photochemistry, visible light

## Abstract

Photo‐mediated 6π cyclization is a valuable method for the formation of fused heterocyclic systems. Here we demonstrate that irradiation of cyclic 2‐aryloxyketones with blue LED light in the presence of an Ir^III^ complex leads to efficient and high yielding arylation across a panoply of substrates by energy transfer. 2‐Arylthioketones and 2‐arylaminoketones also cyclize effectively under these conditions. Quantum calculation demonstrates that the reaction proceeds via conrotatory ring closure in the triplet excited state. Subsequent suprafacial 1,4‐hydrogen shift and epimerization leads to the observed cis‐fused products.

Six‐electron cyclization reactions are of fundamental importance within contemporary synthetic chemistry. Most investigations in this field have focussed on thermal processes, with relatively little work on the corresponding photochemical reactions.^1^ Within this field, photochemical 6π heterocyclization offers a distinct strategic approach to the synthesis of complex heterocyclic systems. Chapman demonstrated that irradiation of *N*‐aryl enamines led to the formation of indolines bearing predominantly *trans*‐stereochemistry across the ring junction (Scheme 1). This reaction, which is isoelectronic with the pentadienyl anion electrocyclization,^2^ was proposed to occur through excited state electrocyclization followed by a suprafacial 1,4‐hydrogen shift.^3^ A related transformation was disclosed by Schultz, who reported that photocyclization of 2‐aryloxyketones and 2‐arylthioketones could be achieved with pyrex‐filtered UV irradiation to yield fused dihydrobenzofurans and dihydrobenzothiophenes, respectively.^4^, ^5^, ^6^ This reaction offers a diastereoselective route to *cis*‐fused heterocycles useful in a range of synthetic endeavours, which we exploited in a recent total synthesis of morphine.^7^ Although the Schultz photocyclization reaction was the key stereodefining step in our total synthesis, the reactivity of the products under high intensity UV light and the sensitivity of conversion to varying substitution on the aromatic ring motivated us to find an alternative method to achieve this valuable transformation. In principle, visible light photoredox catalysis^8^ or triplet energy transfer^9^ from an appropriately activated sensitizer could both offer solutions to this problem. In considering the feasibility of these two approaches, we rationalized that the estimated reduction potentials of cyclic aryloxyketones (*E* ${}_{1/2}$ ≈−2.2 V vs. SCE) lie outside of the reducing ability of most excited state photocatalysts.^10^ In contrast, the triplet energy of cyclic aryloxyketones is ≈60 kcal mol^−1^, which is of a similar magnitude to the triplet energies of some excited state iridium photocatalysts.^11^ This principle has been elegantly applied to the energy transfer mediated [2+2] cycloaddition of olefins^12^ and other processes.^13^ Consequently, we prepared a test 2‐aryloxyketone substrate **1 a** through treatment of the epoxide derived from 3‐methylcyclohexenone with phenol in the presence of potassium carbonate in acetonitrile,^14^ and examined its reactivity in the presence of a range of photocatalysts under irradiation with blue LED light (Table 1).^15^

**Scheme 1:**
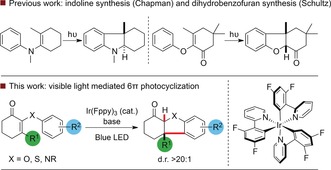
Previous work and approach to visible light mediated 6π cyclization.

**Table 1:** Optimization: photocyclization of 2‐aryloxyketones. 
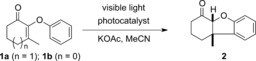

Entry	Catalyst	Conversion [%]	Yield [%]
1	Ru(bpy)_3_Cl_2_	0	–
2	Ru(phen)_3_Cl_2_	0	–
3	[Ir(dtbbpy)(ppy)_2_]PF_6_	<5	trace
4	*fac*‐Ir(ppy)_3_	45	44
5	[Ir(dF(CF_3_)ppy)_2_(dtbbpy)]PF_6_	80	77
6	Ir(Fppy)_3_	100	95

[a] Reaction conditions: **1 a** (0.05 mmol), KOAc (1.0 equiv), catalyst (1 mol %), 12 W blue LED, MeCN ([**1 a**]=0.05 mol dm^−3^), 60 °C, 16 h. Conversion and yields measured by ^1^H NMR spectroscopy vs. internal standard.

Ruthenium(II) catalysts proved to be ineffective (entries 1 and 2),^16^ and hence we examined photocatalysts with significantly higher triplet energies. [Ir(dtbbpy)(ppy)_2_]PF_6_ (entry 3)^17^ afforded a trace of product, but we observed significant reactivity with *fac*‐Ir(ppy)_3_ leading to **2** in 44 % yield (entry 4). Significantly higher conversion was observed with [Ir(dF(CF_3_)ppy)_2_(dtbbpy)]PF_6_ (77 % yield), and Ir(Fppy)_3_ gave complete conversion and a 95 % yield of **2**; only the *cis*‐dihydrobenzofuran isomer was isolated under these reaction conditions.^18^ Addition of triplet quenchers (TEMPO or 1,1‐diphenylethylene) inhibited the reaction entirely and only starting material was isolated, consistent with an energy transfer mechanism.^19^ With a working substrate synthesis and cyclization procedure in hand, we examined the scope and limitations of this visible light mediated process (Table 2). We initially explored cyclization of cyclohexenone substrates with different substituents on the aromatic ring. Substituents in the 6‐position were well tolerated including alkyl groups (**3**, 90 %) and halogens (difluoro‐derivative **4**, 87 %; chloro‐ **5**, 89 %; bromo‐ **6** 92 %; iodo‐ **7**, 78 %). Substrates bearing electron‐donating groups such as **8** and **9** both cyclize effectively; in these cases the competing [2+2] cycloaddition is likely disfavoured by geometrical constraints precluding close approach of the enone and allyl groups. Introducing 7‐substitution does not have any deleterious effects on the reaction, and tetracycle **10** can be produced in 93 % yield. *meta*‐Substituents on cyclohexenone‐derived aryloxy substrates generally cyclize to give regioisomeric ratios in which the 7‐isomer is the minor product: 9/7‐methyl **11** (91 % combined yield, ratio 2.2:1) and 9/7 dimethylamino **12** (68 % combined yield, ratio 2.3:1). It is worth noting that electron‐rich substrates efficiently cyclize to products such as **12** in high yields; under high intensity UV irradiation this cyclization does not proceed.4e Symmetrical disubstituted substrates cyclize effectively whether electron donating (**13**, 94 % yield) or electron withdrawing (**14**, 92 % yield). A similar trend is observed with groups in the 8‐position: pyridine **15** (76 % yield), and both electron withdrawing groups (aldehyde **16**, 75 % yield; ester **17**, 95 % yield; nitrile **18**, 86 % yield) and electron donating groups (methoxy **19**, 90 % yield; benzyloxy **20**, 95 % yield; amide **21**, 90 % yield; thioalkyl **22**, 88 % yield; alkyl **23**, 91 % yield) are tolerated without incident. Using an isophorone‐derived substrate bearing a *gem*‐dialkyl substituent on the cyclohexane ring4c does not affect the reaction significantly, and the product **24** an be isolated in 95 % yield. Variation in the β‐position of the cyclohexenone is also possible: ethyl **25** (93 % yield) and isopropyl **26** (80 % yield; 15:1 d.r.) groups can be accommodated at the all‐carbon quaternary stereocentre without reduction in overall yield. Extension to a derivative of progesterone was also possible, yielding **27** in 84 % yield (d.r. 4:1). Cyclization of substrates bearing hydrogen in the β‐position of the enone is possible, though these are more challenging substrates and can undergo competing oxidation to benzofurans. This can be circumvented somewhat by performing the reaction in the presence of 8‐methylquinoline. Under these conditions cyclization to afford **28** occurred in 88 % yield, and introduction of groups around the aromatic ring of this template was well tolerated as exemplified by 8‐methyl **29** (87 % yield) and 8‐phenyl **30** (80 % yield) products. Introduction of an electron withdrawing group (**31**, 87 % yield), halogens (**32**, 76 % yield and **33**, 56 % yield), or an electron‐donating group (**34**, 85 % yield) in this position did not affect the efficacy of the procedure. The *cis*‐stereochemistry at the ring junction was confirmed by single crystal X‐ray diffraction of **2**, **3**, **22** and **28**.^20^ We also examined the photocyclization of aryloxycyclopentenones, which were significantly more challenging to work with than the corresponding cyclohexenones. In general, longer reaction times are required and the process is sensitive to the presence of electron withdrawing groups on the aromatic ring, but high yielding transformations can be achieved. Thus **35** can be generated in high yield (87 %) as a single diastereoisomer. Halogens can be tolerated in the 6‐position on the aromatic ring (chloro‐ **36**, 36 % yield; bromo‐ **37** 65 % yield iodo‐ **38**, 45 % yield). In contrast to the cyclohexenone series, *meta*‐substituents on aryloxy substrates generally cyclize to give regioisomeric ratios in which the 7‐isomer is the major product (9/7‐dimethylamino **39**; 67 % combined yield, ratio 1:1.6). Other electron donating substituents in the 8‐position on the aromatic ring are similarly effective: 8‐methoxy **40** (83 % yield) and 8‐methyl **41** (86 % yield). Electron withdrawing groups are particularly challenging (8‐formyl **42**, 23 % yield and ester **43**, 38 % yield), leading to lower conversions and yields. The fused pyridine **44** can be generated in 52 % yield. Variation in the β‐position of the cyclopentanone is also remarkably well tolerated: ethyl **45** (86 % yield), benzyl **46** (71 % yield) and cyclohexyl **47** (86 % yield) groups can be installed at the fully substituted centre without issue. The *cis*‐stereochemistry at the ring junction was confirmed by single crystal X‐ray diffraction of **40**. Cyclization of 2‐arylthioketones and 2‐arylaminoketones is also effective under these conditions. Dihydrothiophenes can be be formed in high yields, affording both six (**49**, 91 % yield) and five‐membered fused ring systems (**50**, 87 % yield). In a related process, *N*‐methylated 2‐arylaminoketones cyclize to afford dihydroindoles in good yields in both five and six ring series (**51**, 77 % yield; **52**, 84 % yield). Different *N*‐substituents are tolerated without incident as exemplified by *N*‐allyl **53** (52 % yield) and *N*‐benzyl **54** (88 % yield) derivatives. This approach can also be extended to generate fused tetracylic systems such as **55** (77 % yield) by cyclization of a tetrahydroquinoline substrate. In general, the cyclization procedure is highly effective and demonstrates remarkable insensitivity to substitution, particularly in the cyclohexenone series. The scalability of this process is demonstrated by a 5.00 mmol reaction (1.01 g) to generate **2**, which proceeds in 92 % yield with a photocatalyst loading of 0.05 mol %. A series of chemo‐and diastereoselective derivatizations were also performed on this compound.^15^ In our hands the reaction was also insensitive to the presence of moisture. However, several substrates possessed distinct reactivity or were surprisingly unreactive under these reaction conditions (Scheme 2). Substrate **56**, which contains an α,β,γ,δ‐unsaturated ketone, undergoes efficient intermolecular [2+2] cycloaddition (71 % yield) rather than the arylation reaction to afford cyclobutane **57**, in which stereochemistry was confirmed by single crystal X‐ray diffraction. This is consistent with previous observations on the rate of [2+2] cycloadditions versus the arylation reaction.^21^

**Scheme 2:**
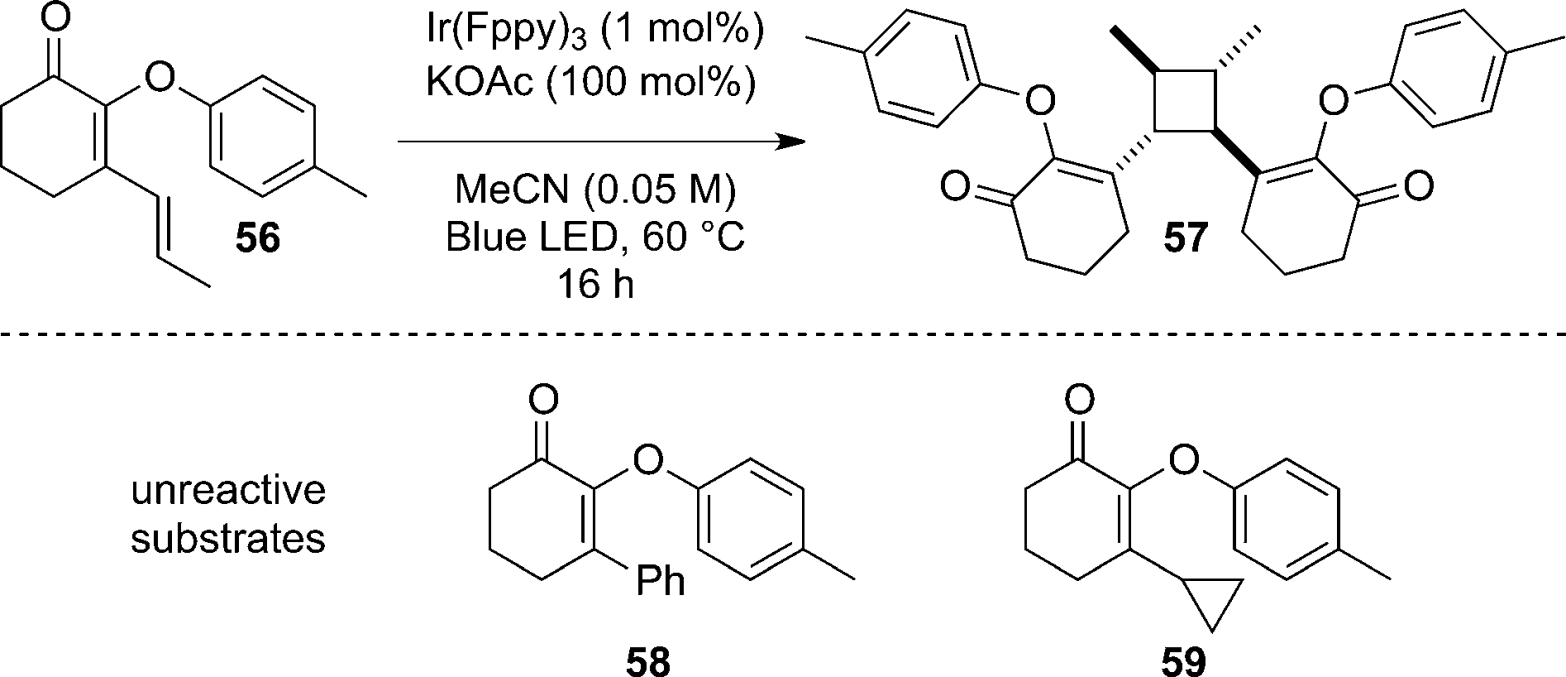
2‐Aryloxyketone substrates with distinct reactivity profiles.

**Table 2:** Scope of triplet energy transfer mediated 6π heterocyclization. 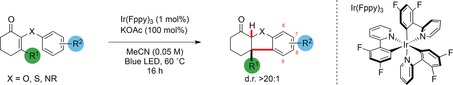

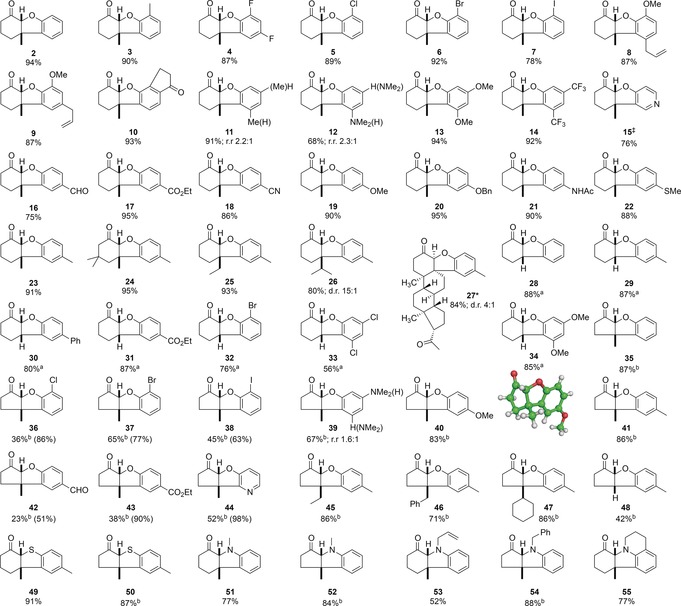

Reaction conditions: 0.3 mmol enone, 0.01 equiv Ir(Fppy)_3_, 1.0 equiv KOAc, 12 W blue LED, MeCN ([enone]=0.05 m); reaction time 16 h. Yields are for isolated material. d.r. (diastereoisomeric ratio) determined by ^1^H NMR spectroscopy. Positions around the benzofuran core are indicated in red numerals. [≠] Reaction time 72 h. [*] Reaction time 36 h. [a] Reaction conducted in the presence of 8‐methylquinoline (0.2 equiv); reaction time 2–7 h. ^b^Reaction conducted using NaOAc (1.0 equiv) in EtOAc; reaction time 40 h. Numbers in parentheses indicate yields calculated on the basis of recovered starting material. r.r. (regioisomeric ratio) determined by ^1^H NMR spectroscopy; minor regioisomer is indicated by parentheses.

β‐Aryl substrate **58** and β‐cyclopropyl substrate **59** were both unreactive under the optimized reaction conditions and were re‐isolated unchanged. To probe the reaction mechanism (Figure 1 A) and divergent reactivity of some of the substrates investigated, we turned to computations (M06‐2X+SMD/def2‐TZVP).^22^ The calculated energy required to promote cyclohexanone **1 a** from singlet (S_0_) to π–π* triplet (T_1_) state is 58.2 kcal mol^−1^.^23^ This is well matched with the emissive energy of the Ir(Fppy)_3_ catalyst (experimentally 60.1 kcal mol^−1^).^11^ Triplet excitation of cyclopentenone **1 b**, which required longer reaction times, requires an additional 4.1 kcal mol^−1^ of energy. Substrate **58** forms a more stable triplet state in comparison to **1 a** (48.8 kcal mol^−1^; Figure 1 B). However, heterocyclization is kinetically and thermodynamically less favorable: TS_AB_ has a higher barrier and this step is endergonic by 3.8 kcal mol^−1^. The intersystem crossing (ISC) from the triplet states of unreactive substrates **58** and **59** involves smaller changes in energy and in dihedral twisting relative to **1 b**. This is consistent with more efficient relaxation from the triplet back to the singlet ground state for these substrates. Heterocyclization proceeds via triplet intermediate ^**3**^ **A**,which preferentially occurs in a conrotatory fashion (Figure 1 B, with several other substrates shown in SI). Conrotatory closure through TS_AB_, is favoured over disrotatory closure by more than 10 kcal mol^−1^ (Δ*G* ^≠^ 13.5 kcal mol^−1^). This preference is consistent with the expectation that a conrotatory 6π electrocyclic TS is Möbius aromatic in the triplet excited state.^24^ However, computed magnetic criteria of aromaticity (NICS, ACID, magnetic susceptibility exaltation) do not show the shielding/cyclic ring current characteristics of a pericyclic reaction (see the Supporting Information). Ring‐closed intermediate ^**3**^ **B** lies downhill by 3.5 kcal mol^−1^ (with a β‐phenyl group as in **58**, this step is uphill by a similar amount, which may also contribute towards lower reactivity) and must undergo intersystem crossing (ISC) back to the singlet state. Interestingly we find the open‐shell singlet structure of ^**1**^ **B** to be 7 kcal mol^−1^ more stable than the closed‐shell (e.g. ylide) structure. ^**1**^ **B** and ^**3**^ **B** geometries, along with that of the minimum energy crossing point (MECP) are very similar, which facilitates ISC. The subsequent suprafacial [1,4]‐H shift is Woodward–Hoffmann allowed in the singlet ground state, with a computed barrier of 14.2 kcal mol^−1^. This sigmatropic rearrangement is extremely exergonic and leads to *trans*‐fused **C**. The diastereomeric *cis*‐fused product could arise through base‐mediated epimerization of **C**—it is significantly more stable by 15.3 kcal mol^−1^—or from the non‐stereospecific intermolecular deprotonation/reprotonation of **B**. The extremely large preference (ca. 10 kcal mol^−1^) for conrotatory closure was obtained for all substrates and so it is implausible that the *cis*‐fused skeleton is the result of disrotatory cyclization.

**Figure 1:**
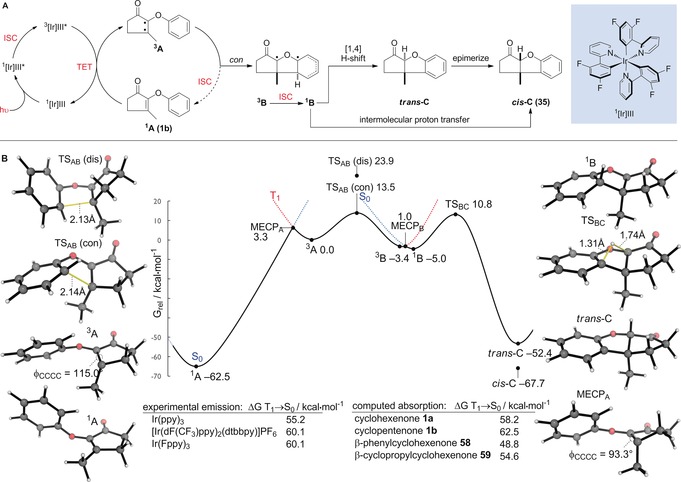
A) Proposed mechanism of 6π heterocyclization of **1 b** to form **35**. B) M06‐2X/def2‐TZVP+SMD(EtOAc)//M06‐2X/def2‐TZVP computed Gibbs energy profile (kcal mol^−1^) for the cyclization of **1 b** to form **35** taking place on singlet and triplet surfaces. ISC=intersystem crossing; TET=triplet energy transfer; MECP=minimum energy crossing point; dis=disrotatory; con=conrotatory.

In conclusion, we have described an operationally convenient approach to the synthesis of fused dihydrobenzofurans, dihydroindoles and dihydrothiophenes using an iridium(III) sensitizer in the presence of visible light to mediate energy transfer. This is a versatile reaction that enables the synthesis of fused heterocyclic ring systems with a wide variety of substituents in good yields. Quantum calculations demonstrate that this reaction proceeds in the triplet state with a large preference for conrotatory ring closure, but without the magnetic characteristics of an electrocyclic mechanism.

## Conflict of interest

The authors declare no conflict of interest.

## Supporting information

As a service to our authors and readers, this journal provides supporting information supplied by the authors. Such materials are peer reviewed and may be re‐organized for online delivery, but are not copy‐edited or typeset. Technical support issues arising from supporting information (other than missing files) should be addressed to the authors.

Supplementary
