# Supplementary material for: Visible Light Photocatalysis of 6π Heterocyclization
Source: Angew Chem Int Ed Engl. 2017 Jul 4;56(32):9468–72. doi: 10.1002/anie.201705333 (PMC5575533; doi:10.1002/anie.201705333)
Supplement: Supplementary file 1 — Supplementary [file ANIE-56-9468-s001.pdf]

Angewandte Chemie
Eine Zeitschrift der Gesellschaft Deutscher Chemiker

Supporting Information

Visible Light Photocatalysis of 6π Heterocyclization

Niels Münster, Nicholas A. Parker, Lucy van Dijk, Robert S. Paton, and Martin D. Smith**

anie_201705333_sm_miscellaneous_information.pdf

Supporting Information

Contents

I. General Information S2
II. Catalyst Synthesis S3
III. Synthesis of Substrates S4
IV. Optimization Data S36
V. Photocyclization S39
VI. Derivatization S69
VII. Computational Procedures S73
VIII. References S87
IX. NMR Spectra S89

I. General Information

Reactions requiring moisture-sensitive reagents were carried out in flame-dried glassware, under an atmosphere of argon (balloon pressure). Dichloromethane, tetrahydrofuran and acetonitrile were purified by filtration through activated alumina columns employing the method of Grubbs et al.[1] Water was purified by an Elix® UV-10 system. If needed, solvents were degassed by sparging with argon for 30 min. For photocylization reactions, degassed HPLC grade solvents were used without any further purification. Commercially available reagents were used directly as supplied by major chemical suppliers without further purification. Petrol 40-60 (PE) refers to the fraction of petroleum ether which boils in the range 40-60 °C. Brine refers to a saturated aqueous solution of sodium chloride.

A reactor consisting of 12 W blue LED strips wrapped around the inside of a 260 x 160 mm metal tin was used for all photoreactions. The temperature was monitored with an internal thermometer and was adjusted with a stream of nitrogen if necessary. Reactions were generally placed 2-3 cm away from the lights.

Silica gel chromatography was carried out using Merck Geduran® Silicagel (40-63 µm particle size). Thin layer chromatography (TLC) was carried out using pre-coated, aluminium backed plates (Merck Kieselgel 60 F254). Visualisation was achieved with ultraviolet irradiation (254 nm) and staining with potassium permanganate solution. NMR spectroscopy was carried out using Bruker Avance spectrometers in the deuterated solvent stated, using the residual non-deuterated solvent signal as an internal reference (^{1}H NMR: $CDCl_3$ (7.26); ^{13}C NMR: $CDCl_3$ (77.16); ^{19}F NMR: $CFCl_3$ (0.00)). Chemical shifts are quoted in ppm, based on appearance rather than interpretation. Signal patterns are indicated as: br s, broad singlet; s, singlet; d, doublet; t, triplet; q, quartet; quin, quintet; m, multiplet. Coupling constants, *J*, are quoted to the nearest 0.1 Hz and are presented as observed. All ^{19}F NMR spectra are reported as proton/fluorine decoupled unless otherwise stated.

Infrared spectra were prepared as a neat film and were recorded using a Bruker Tensor 27 FTIR spectrometer using an ATR module. Absorption maxima are quoted in wavenumbers for the range 3500-600 cm^{-1}.

Low resolution mass spectrometry was carried out using electrospray ionization (ESI) and was performed on a Micromass LCT Premier Spectrometer. HRMS was carried out using Bruker MicroTDF and Micromass GCT spectrometers under ESI or ammonia chemical ionization (ACI)/electron ionization (EI) conditions respectively. The mass reported is that containing the most abundant isotopes.

Melting points were determined using a Reichert melting point apparatus or a Leica VMTG heated-stage microscope equipped with a Testo 720 thermometer and are reported uncorrected

II. Catalyst Synthesis

Tris[2-(4,6-difluorophenyl)pyridinato-C^2,*N*]iridium(III)

$$[Ir(Fppy)_2Cl]_2 \xrightarrow[69\%]{\text{Fppy, AgOTf}} Ir(Fppy)_3$$

To a 50 mL round-bottomed flask fitted with a condenser was added $[Ir(Fppy)_2Cl]_2$ (450 mg, 0.37 mmol) and 2-ethoxyethanol (15 mL). The system was evacuated carefully and backfilled with argon three times, followed by addition of silver trifluoromethanesulfonate (285 mg, 1.11 mmol) and 2-(2,4-difluorophenyl)pyridine (0.34 ml, 2.22 mmol). The mixture was heated to reflux (125 °C) for 18 hours, before being cooled to room temperature. The solvent was removed under reduced pressure by azeotropic distillation with toluene. Purification by flash chromatography (CH_2Cl_2) afforded tris[2-(4,6-difluorophenyl)pyridinato-C^2,*N*]iridium(III) as a yellow-green solid (391 mg, 0.51 mmol, 69%, ratio *fac*/*mer* = 10:1). The spectral data matched that previously reported in the literature.[2]

^{1}H NMR (400 MHz, $CDCl_3$) δ = 8.33-8.28 (m, 3H), 7.72-7.66 (m, 3H), 6.93 (ddd, *J* = 7.3, 5.7, 1.4 Hz, 3H), 6.40 (ddd, *J* = 13.0, 9.0, 2.5 Hz, 3H), 6.25 (dd, *J* = 9.0, 2.5 Hz, 3H).

III. Synthesis of Substrates

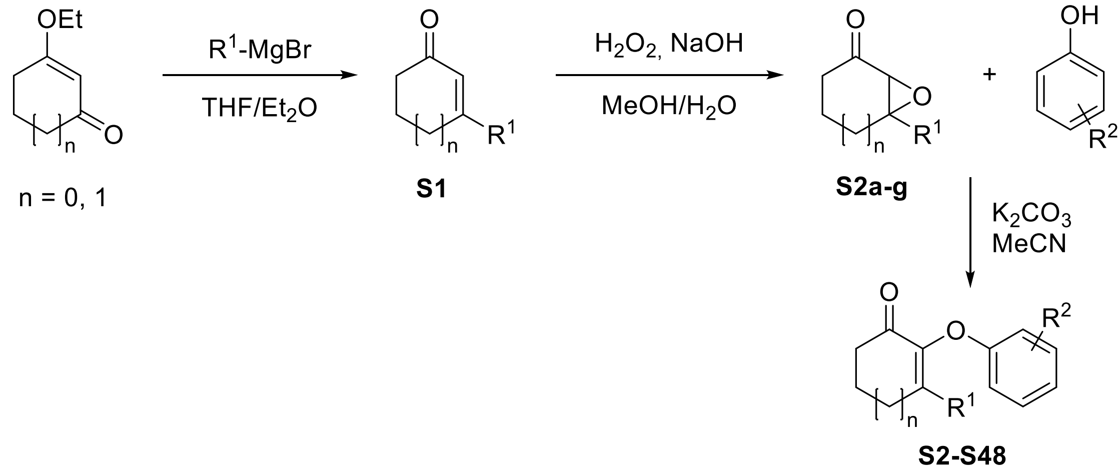

Scheme 1 General synthetic route to the enone substrates.

Synthesis of Enones

2-Cyclohexen-1-one, 3-methyl-2-cyclohexenone, isophorone, 2-cyclopenten-1-one, 3-methyl-2-cyclopentenone and progesterone were commercial available and used without further purification. 3-Ethyl-2-cyclohexenone,[3] 3-isopropyl-2-cyclohexenone,[3] 3-phenyl-2-cyclohexenone,[3] 3-allyl-2-cyclohexenone,[4] 3-ethyl-2-cyclopentenone,[3] 3-benzyl-2-cyclopentenone[5] and 3-cyclohexyl-2-cyclopentenone[6] were synthesized according to literature procedures.

3-Cyclopropylcyclohex-2-en-1-one (S1)

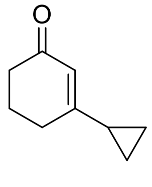

Magnesium turnings (1.04 g, 42.8 mmol) were suspended in Et_2O (15 mL) and 5 mL of a solution of cyclopropylbromide (5.18 g, 42.8 mmol) in Et_2O (15 mL) was added. The mixture was heated to reflux until the Grignard formation initiated. The heating bath was then removed and the rest of the cyclopropylbromide solution was added dropwise to the reaction mixture. After finishing the addition, the mixture was heated to reflux for 1 h. THF (15 mL) was added to reaction mixture and subsequently heated to reflux for an additional 1 h. The resulting suspension was cooled to 0 °C and 3-ethoxycyclohexanone (1.50 g, 10.7 mmol) was added dropwise. The cooling bath was then removed and the reaction mixture was stirred at room temperature for 30 min. Subsequently, the suspension was cooled to 0 °C and quenched by the addition of 1 M HCl (80 mL). The mixture was extracted with Et_2O (3 × 50 mL). The combined organic extracts were washed with sat. aq. $NaHCO_3$ (50 mL) and brine (50 mL), dried over $MgSO_4$ and concentrated under reduced pressure. Purification of the residue *via* flash chromatography (PE:Et_2O, 2:1) afforded the enone **S1** (1.18 g, 8.66 mmol, 81%) as a pale yellow liquid. The spectral data matched that previously reported in the literature.[7]

^{1}H NMR (400 MHz, $CDCl_3$) δ = 5.85 (s, 1H), 2.34 (dd, J = 6.7, 6.7 Hz, 2H), 2.13 (dd, J = 6.0, 6.0 Hz, 2H), 2.00-1.90 (m, 2H), 1.60-1.50 (m, 1H), 0.95-0.86 (m, 2H), 0.79-0.72 (m, 2H).

^{13}C NMR (101 MHz, $CDCl_3$) δ = 199.3, 168.7, 123.3, 37.6, 27.0, 22.6, 18.0, 8.3.

<u>Synthesis of Epoxides S2</u>

2,3-Epoxy-3-methylcyclohexanone,[8] 2,3-epoxycyclohexanone,[9] 2,3-epoxyisophorone,[10] 2,3-epoxy-3-phenylcyclohexanone,[11] 2,3-epoxycyclopentanone[12] and 2,3-epoxy-3-methylcyclopentanone[13] were prepared according to literature procedures.

General Procedure A:

To a 3-necked round-bottomed flask equipped with a magnetic stirring bar was added enone (1.0 eq.) and methanol (1 mL per mmol substrate). The solution was cooled to 0 °C before addition of aq. H_2O_2 (30%, 3.0 eq.), followed by aq. NaOH solution (3 M, 0.2 eq.) with vigorous stirring. After complete consumption of the enone by TLC analysis, the reaction mixture was poured into a separating funnel containing ice (15 mL) and saturated aqueous sodium chloride solution (15 mL) and extracted with dichloromethane (3 × 20 mL). The combined organic extracts were dried (Na_2SO_4), filtered and concentrated *in vacuo*. The crude product was then used directly in **General Procedure B** if volatile or purified by flash chromatography.

2,3-Epoxy-3-ethylcyclohexanone (S2a)

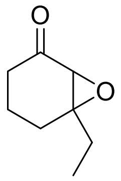

To a solution of aq. H_2O_2 (30%, 5.2 mL, 51.9 mmol) in MeOH (20 mL) was added 3-ethyl-2-cyclohexenone (2.15 g, 17.3 mmol) at 2-3 °C (internal thermometer). Aq. NaOH (3 M, 1.1 mL, 3.5 mmol) was then added over 20 min ensuring that the temperature stayed below 10 °C. After stirring for 45 min the reaction mixture was poured into water (20 mL) and extracted with CH_2Cl_2 (4 × 20 mL). The combined organic extracts were dried over Na_2SO_4, filtered and concentrated under reduced pressure. The crude 2,3-epoxy-3-methylcyclohexanone (**S2a**) (2.33 g, 16.6 mmol, 96%) was used without any further purification. The spectral data matched that previously reported in the literature.[14]

^{1}H NMR (400 MHz, $CDCl_3$) δ = 3.10 (s, 1H), 2.51 (ddd, J = 17.7, 4.7, 4.7 Hz, 1H), 2.17-1.60 (m, 7H), 0.98 (t, J = 7.5 Hz, 3H).

2,3-Epoxy-3-isopropylcyclohexanone (S2b)

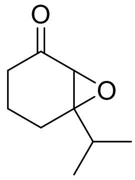

To a solution of 3-isopropyl-2-cyclohexenone (784 mg, 5.67 mmol) in MeOH (10 mL) was added aq. H_2O_2 (30%, 1.7 mL, 17.0 mmol) at 5 °C (internal thermometer). Aq. NaOH (3 M, 0.37 mL, 1.13 mmol) was then added dropwise over 10 min. The reaction mixture was stirred for 1 h while keeping the temperature around 5 °C. The cooling bath was removed and the mixture was stirred at room temperature for further 2 h. Another portion of aq. NaOH (3 M, 0.57 mL, 1.70 mmol) was added and the reaction was stirred for an additional 3 h. The reaction was poured into water/brine (1:1, 20 mL) and extracted with CH_2Cl_2 (3 × 20 mL). The combined

organic extracts were dried over Na_2SO_4, filtered and concentrated under reduced pressure. Purification of the residue *via* flash chromatography (PE:Et_2O, 4:1) afforded the title compound **S2b** (584 mg, 3.79 mmol, 67%) as pale yellow liquid. The spectral data matched that previously reported in the literature.[14]

^{1}H NMR (400 MHz, $CDCl_3$) δ = 3.08 (s, 1H), 2.52 (ddd, *J* = 17.6, 5.0, 5.0 Hz, 1H), 2.14-1.79 (m, 4H), 1.72-1.59 (m, 2H), 1.04 (d, *J* = 6.8 Hz, 3H), 0.97 (d, *J* = 7.0 Hz, 3H).

^{13}C NMR (101 MHz, $CDCl_3$) δ = 207.4, 69.4, 61.0, 36.3, 34.2, 23.1, 18.0, 17.8.

2,3-Epoxy-3-cyclopropylcyclohexanone (S2c)

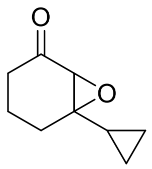

To a solution of aq. H_2O_2 (30%, 2.6 mL, 26.0 mmol) in MeOH (8 mL) was added 3-cyclopropyl-2-cyclohexenone (**S1**) (1.18 g, 8.66 mmol) at 2-3 °C (internal thermometer). Aq. NaOH (3 M, 1.1 mL, 3.46 mmol) was then added dropwise over 20 min. After stirring for 30 min at 0 °C the reaction was warmed to room temperature and was stirred at this temperature for an additional 90 min. The reaction was poured into water/brine (1:1, 30 mL) and extracted with CH_2Cl_2 (3 × 30 mL). The combined organic extracts were dried over Na_2SO_4, filtered and concentrated under reduced pressure. Purification of the residue *via* flash chromatography (PE:Et_2O, 5:1) afforded the title compound **S2c** (1.05 g, 6.90 mmol, 80%) as a pale yellow liquid.

^{1}H NMR (400 MHz, $CDCl_3$) δ = 3.03 (s, 1H), 2.54–2.44 (m, 1H), 2.22–1.84 (m, 4H), 1.72-1.58 (m, 1H), 1.22–1.12 (m, 1H), 0.59–0.48 (m, 1H), 0.45-0.26 (m, 3H).

^{13}C NMR (101 MHz, $CDCl_3$) δ = 206.8, 60.0, 60.9, 36.2, 27.2, 17.5, 14.8, 2.2, 0.6.

FTIR (neat) ν = 3010, 2947, 1713, 1456, 1403, 1326, 1272, 1250, 1054, 1029, 974, 912, 892, 815, 777.

HRMS (ESI) *m/z* calcd. for $C_9H_{12}O_2$ $[M+H]^+$: 153.0910; found: 153.0910.

2,3-Epoxy-3-allylcyclohexanone (S2d)

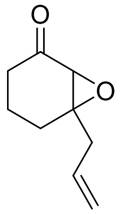

To a solution of crude 3-allyl-2-cyclohexenone (2.91 g, 21.4 mmol) in MeOH (25 mL) was added aq. H_2O_2 (30%, 6.4 mL, 64.2 mmol) at 0 °C. Then aq. NaOH (3 M, 2.8 mL, 8.56 mmol) was added dropwise over 10 min. After stirring for 15 min at 0 °C the reaction mixture was warmed to room temperature and stirred at this temperature for 2 h. The reaction was poured into water/brine (1:1, 40 mL) and extracted with CH_2Cl_2 (3 × 40 mL). The combined organic extracts were dried over Na_2SO_4, filtered and concentrated under reduced pressure. Purification of the residue *via* flash chromatography (PE:Et_2O, 5:1) afforded the title compound **S2d** (2.17 g, 14.3 mmol, 67% over 2 steps) as a pale yellow liquid. The spectral data matched that previously reported in the literature.[15]

^{1}H NMR (400 MHz, $CDCl_3$) δ = 5.81-5.69 (m, 1H), 5.20-5.16 (m, 1H), 5.16-5.11 (m, 1H), 3.12 (s, 1H), 2.56-2.37 (m, 3H), 2.18-1.83 (m, 4H), 1.77-1.60 (m, 1H).

^{13}C NMR (101 MHz, $CDCl_3$) δ = 206.6, 131.6, 119.3, 64.6, 60.4, 40.3, 35.9, 26.5, 17.4.

2,3-Epoxy-3-ethylcyclopentanone (S2e)

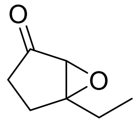

Prepared according to **General Procedure A** with 3-ethyl-2-cyclopenten-1-one (275 mg, 2.5 mmol), aq. H_2O_2 (30%, 0.75 ml, 7.50 mmol) and methanol (5 ml) for 2 h to afford epoxide **S2e** as a colourless oil (141 g, 1.18 mmol, 45%) which was used without any further purification. The spectral data matched that previously reported in the literature.[16]

^{1}H NMR (400 MHz, $CDCl_3$) δ = 3.17 (s, 1H), 2.51–2.21 (m, 2H), 2.18–2.04 (m, 2H), 2.01–1.78 (m, 2H), 1.01 (t, *J* = 7.5 Hz, 3H).

^{13}C NMR (101 MHz, $CDCl_3$) δ = 211.2, 69.9, 60.2, 32.7, 25.7, 24.9, 9.2.

2,3-Epoxy-3-benzylcyclopentanone (S2f)

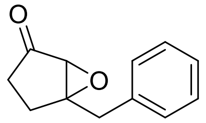

Prepared according to **General Procedure A** with 3-benzyl-2-cyclopenten-1-one (517 mg, 3.00 mmol), aq. H_2O_2 (30%, 0.90 ml, 9.00 mmol) and methanol (6 ml) for 4 h. Purification *via* flash column chromatography (PE:Et_2O, 2:1) afforded epoxide **S2f** as a yellow oil (430 mg, 2.28 mmol, 76%). The spectral data matched that previously reported in the literature.[16]

^{1}H NMR (400 MHz, $CDCl_3$) δ = 7.37–7.27 (m, 2H), 7.27–7.23 (m, 1H), 7.23–7.16 (m, 2H), 3.16 (s, 1H), 3.15 (d, *J* = 14.6 Hz, 1H), 3.10 (d, *J* = 14.6 Hz, 1H), 2.33 (ddd, *J* = 17.5, 9.2, 8.1 Hz, 1H), 2.18 (dd, *J* = 13.7, 9.3 Hz, 1H), 2.05 (dd, *J* = 17.8, 9.2 Hz, 1H), 1.92 (ddd, *J* = 13.8, 9.2, 8.1 Hz, 1H).

^{13}C NMR (101 MHz, $CDCl_3$) δ = 210.5, 135.6, 129.5, 128.7, 127.2, 69.1, 60.2, 38.5, 32.8, 25.6.

2,3-Epoxy-3-cyclohexylcyclopentanone (S2g)

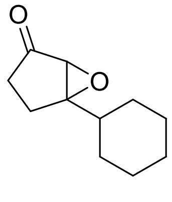

Prepared according to **General Procedure A** with 3-cyclohexyl-2-cyclopenten-1-one (361 mg, 2.20 mmol), aq. H_2O_2 (30%, 0.66 ml, 6.60 mmol) and methanol (4.4 ml) for 4 h. Purification *via* flash column chromatography (PE:Et_2O, 5:1) afforded epoxide **S2g** as a colourless oil (254 mg, 1.41 mmol, 64%).

^{1}H NMR (400 MHz, $CDCl_3$) δ = 3.18 (s, 1H), 2.42–2.29 (m, 1H), 2.29–2.21 (m, 1H), 2.14–2.02 (m, 1H), 2.02–1.89 (m, 1H), 1.85–1.77 (m, 2H), 1.77–1.59 (m, 4H), 1.34–1.04 (m, 5H).

^{13}C NMR (101 MHz, $CDCl_3$) δ = 211.4, 72.3, 59.8, 39.7, 32.5, 29.0, 28.6, 26.2, 26.1, 25.9, 23.7.FTIR (neat) ν/cm^{-1} = 2928, 2854, 1747, 1450, 1402, 1262, 1183, 930, 851, 809, 727.

Synthesis of 2-aryloxyketones and 2-arylthioketones

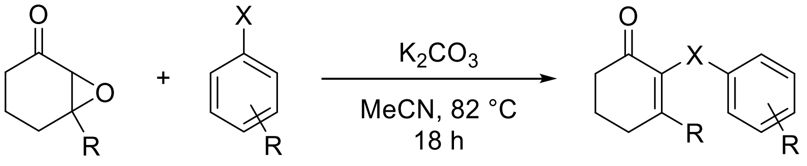

1, S3-S50

General Procedure B (in analogy to literature procedure):[17]

The appropriate phenol (1.5 eq.), epoxide (1.0 eq.) and potassium carbonate (1.2 eq.) were added to a round-bottomed flask fitted with a condenser. The system was evacuated and backfilled with argon three times. Acetonitrile (anhydrous, degassed, 4 mL/mmol epoxide) was then added and the mixture heated to 82 °C for the appropriate time under an atmosphere of argon before being cooled to room temperature. The reaction mixture was poured into aq. NaOH (1 M, 10 mL/mmol) and extracted with Et_2O (3 × 10 mL/mmol). The combined organic extracts were washed with brine (5 mL/mmol), dried over Na_2SO_4, filtered and concentrated under reduced pressure. Purification of the residue *via* flash chromatography (see experimental methods section for specific details) afforded the corresponding 2-aryloxyketone.

3-Methyl-2-phenoxycyclohex-2-en-1-one (1)

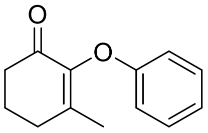

Prepared according to **General Procedure B** with 2,3-epoxy-3-methylcyclohexanone (631 mg, 5.00 mmol), phenol (706 mg, 7.50 mmol), K_2CO_3 (829 mg, 6.00 mmol) and acetonitrile (20 mL). Reaction time: 16 h. Purification *via* flash chromatography (PE:Et_2O, 2:1→1:1) afforded the title compound **1** as a colourless solid (778 mg, 3.84 mmol, 77%).

m.p. 46-47 °C ($CHCl_3$).

^{1}H NMR (400 MHz, $CDCl_3$) δ = 7.30–7.24 (m, 2H), 7.02–6.95 (m, 1H), 6.89–6.82 (m, 2H), 2.61–2.51 (m, 4H), 2.15–2.04 (m, 2H), 1.93 (s, 3H).

^{13}C NMR (101 MHz, $CDCl_3$) δ = 193.1, 157.6, 148.9, 144.2, 129.5, 121.7, 114.7, 38.5, 31.7, 22.2, 18.0.

FTIR (neat) ν/cm^{-1} = 2948, 1681, 1641, 1591, 1491, 1455, 1430, 1377, 1348, 1302, 1221, 1187, 1165, 1145, 1129, 1074, 1027, 930, 806, 756.

HRMS (ESI) *m/z* calcd. for $C_{13}H_{15}O_2$ $[M+H]^+$: 203.1067; found: 203.1067.

3-Methyl-2-(*o*-tolyloxy)cyclohex-2-en-1-one (S3)

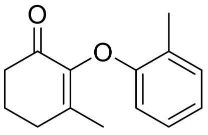

Prepared according to **General Procedure B** with 2,3-epoxy-3-methylcyclohexanone (631 mg, 5.00 mmol), *o*-cresol (811 mg, 7.50 mmol), K_2CO_3 (829 mg, 6.00 mmol) and acetonitrile (20 mL). Reaction time: 15 h. Purification *via* flash chromatography (PE:Et_2O, 2:1) afforded the title

compound **S3** as a colourless solid (792 mg, 3.66 mmol, 73%).

m.p. 64-65 °C ($CHCl_3$).

^{1}H NMR (400 MHz, $CDCl_3$) δ = 7.16 (d, *J* = 7.3 Hz, 1H), 7.02 (dd, *J* = 7.6, 7.6 Hz, 1H), 6.87 (dd, *J* = 7.4, 7.4 Hz, 1H), 6.48 (d, *J* = 8.1 Hz, 1H), 2.57–2.49 (m, 4H), 2.37 (s, 3H), 2.12–2.03 (m, 2H), 1.91 (s, 3H).

^{13}C NMR (101 MHz, $CDCl_3$) δ = 193.0, 155.8, 148.3, 144.8, 131.2, 126.6, 126.5, 121.6, 111.9, 38.6, 31.7, 22.2, 18.0, 16.4.

FTIR (neat) ν/cm^{-1} = 2948, 1681, 1640, 1604, 1587, 1489, 1455, 1432, 1377, 1348, 1327, 1305, 1228, 1193, 1181, 1146, 1133, 1045, 1028, 930, 776.

HRMS (EI) *m/z* calcd. for $C_{14}H_{16}O_2$ [M]$^+$: 216.1150; found: 216.1150.

2-(2,4-Difluorophenoxy)-3-methylcyclohex-2-en-1-one (S4)

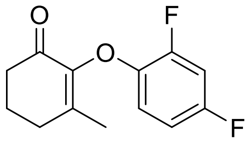

Prepared according to **General Procedure B** with 2,3-epoxy-3-methylcyclohexanone (631 mg, 5.00 mmol), 2,4-difluorophenol (976 mg, 7.50 mmol), K_2CO_3 (829 mg, 6.00 mmol) and acetonitrile (20 mL). Reaction time: 14 h. Purification *via* flash chromatography (PE:Et_2O, 2:1) afforded the title compound **S4** as a colourless solid (998 mg, 4.19 mmol, 84%).

m.p. 87-88 °C ($CHCl_3$).

^{1}H NMR (400 MHz, $CDCl_3$) δ = 6.91-6.81 (m, 1H), 6.73-6.61 (m, 2H), 2.57-2.46 (m, 4H), 2.10-2.00 (m, 2H), 1.95 (s, 3H).

^{13}C NMR (101 MHz, $CDCl_3$) δ = 192.5, 157.1 (dd, *J* = 242, 10.3 Hz), 151.8 (dd, *J* = 249, 12.3 Hz), 148.6, 144.5, 142.0 (d, *J* = 10.7 Hz), 116.2 (dd, *J* = 9.5, 2.2 Hz), 110.4 (dd, *J* = 22.9, 3.9 Hz), 105.2 (dd, *J* = 26.9, 22.1 Hz), 38.4, 31.7, 22.1, 17.9.

^{19}F NMR (377 MHz, $CDCl_3$) δ = −119.2, −129.7.

FTIR (neat) ν/cm^{-1} = 3072, 1679, 1645, 1620, 1506, 1459, 1430, 1379, 1348, 1329, 1304, 1251, 1203, 1185, 1144, 1129, 1099, 965, 931, 877, 806, 725.

HRMS (CI) *m/z* calcd. for $C_{13}H_{13}O_3F_2$ [M+H]$^+$: 239.0884; found: 239.0888.

2-(2-Chlorophenoxy)-3-methylcyclohex-2-en-1-one (S5)

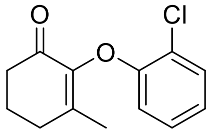

Prepared according to **General Procedure B** with 2,3-epoxy-3-methylcyclohexanone (631 mg, 5.00 mmol), 2-chlorophenol (964 mg, 7.50 mmol), K_2CO_3 (829 mg, 6.00 mmol) and acetonitrile (20 mL). Reaction time: 14 h. Purification *via* flash chromatography (PE:Et_2O, 1:1) afforded the title compound **S5** as a colourless solid (982 mg, 4.15 mmol, 83%).

m.p. 67-68 °C ($CHCl_3$).

^{1}H NMR (400 MHz, $CDCl_3$) δ = 7.38 (dd, *J* = 7.8, 1.5 Hz, 1H), 7.08 (ddd, *J* = 7.8, 7.8, 1.5 Hz, 1H), 6.90 (ddd, *J* = 7.8, 7.8, 1.3 Hz, 1H), 6.59 (dd, *J* = 8.2, 1.4 Hz, 1H), 2.59-2.49 (m, 4H), 2.13-2.04 (m, 2H), 1.93 (s, 3H).

^{13}C NMR (101 MHz, $CDCl_3$) δ = 192.3, 153.2, 149.1, 144.3, 130.7, 127.5, 122.5, 122.4, 114.1, 38.4, 31.7, 22.1, 18.0.

FTIR (neat) ν/cm^{-1} = 2947, 1683, 1644, 1586, 1478, 1447, 1377, 1328, 1304, 1267, 1239, 1188, 1145, 1124, 1058, 1036, 931, 883, 751, 676.

HRMS (ESI) *m/z* calcd. for $C_{13}H_{13}O_2ClNa$ $[M+Na]^+$: 259.0496; found: 259.0495.

2-(2-Bromophenoxy)-3-methylcyclohex-2-en-1-one (S6)

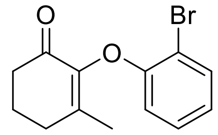

Prepared according to **General Procedure B** with 2,3-epoxy-3-methylcyclohexanone (631 mg, 5.00 mmol), 2-bromophenol (1.30 g, 7.50 mmol), K_2CO_3 (829 mg, 6.00 mmol) and acetonitrile (20 mL). Reaction time: 14 h. Purification *via* flash chromatography (PE:Et_2O, 1:1) afforded the title compound **S6** as a colourless solid (1.14 g, 4.07 mmol, 81%).

m.p. 94-95 °C ($CHCl_3$).

^{1}H NMR (400 MHz, $CDCl_3$) δ = 7.55 (d, *J* = 7.9 Hz, 1H), 7.13 (dd, *J* = 8.2, 8.2 Hz, 1H), 6.84 (dd, *J* = 7.6, 7.6 Hz, 1H), 6.56 (dd, *J* = 8.2, 1.1 Hz, 1H), 2.59-2.50 (m, 4H), 2.12-2.03 (m, 2H), 1.93 (s, 3H).

^{13}C NMR (101 MHz, $CDCl_3$) δ = 192.3, 154.1, 149.2, 144.4, 133.7, 128.3, 122.9, 113.9, 111.4, 38.4, 31.7, 22.1, 18.1.

FTIR (neat) ν/cm^{-1} = 2947, 1682, 1643, 1585, 1472, 1442, 1377, 1348, 1328, 1265, 1236, 1187, 1144, 1133, 1117, 1045, 1030, 885, 751.

HRMS (ESI) *m/z* calcd. for $C_{13}H_{14}O_2Br$ $[M+H]^+$: 281.0172; found: 281.0173.

2-(2-Iodophenoxy)-3-methylcyclohex-2-en-1-one (S7)

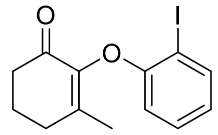

Prepared according to **General Procedure B** with 2,3-epoxy-3-methylcyclohexanone (631 mg, 5.00 mmol), 2-iodophenol (1.65 g, 7.50 mmol), K_2CO_3 (829 mg, 6.00 mmol) and acetonitrile (20 mL). Reaction time: 14 h. Purification *via* flash chromatography (PE:EtOAc, 2:1) afforded the title compound **S7** as a orange solid (1.43 g, 4.38 mmol, 88%).

m.p. 122-123 °C ($CHCl_3$).

^{1}H NMR (400 MHz, $CDCl_3$) δ = 7.78 (dd, *J* = 7.9, 1.5 Hz, 1H), 7.16 (ddd, *J* = 8.3, 7.2, 1.3 Hz, 1H), 6.71 (ddd, *J* = 7.6, 7.6, 1.3 Hz, 1H), 6.49 (dd, *J* = 8.2, 1.3 Hz, 1H), 2.59-2.49 (m, 4H), 2.13-2.03 (m, 2H), 1.92 (s, 3H).

^{13}C NMR (101 MHz, $CDCl_3$) δ = 192.2, 156.4, 149.1, 144.7, 139.7, 129.3, 123.5, 112.9, 85.4, 38.4, 31.7, 22.1, 18.3.

FTIR (neat) ν/cm^{-1} = 2946, 1682, 1641, 1580, 1466, 1438, 1376, 1348, 1327, 1303, 1264, 1233, 1187, 1144, 1132, 1019, 930, 885, 811, 752.

HRMS (EI) *m/z* calcd. for $C_{13}H_{13}O_2I$ $[M]^+$: 327.9960; found: 327.9967.

2-(5-Allyl-2-methoxyphenoxy)-3-methylcyclohex-2-en-1-one (S8)

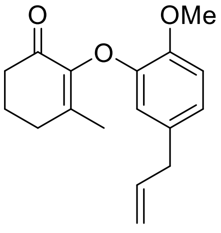

Prepared according to **General Procedure B** with 2,3-epoxy-3-methylcyclohexanone (315 mg, 2.50 mmol), chavibetol[18] (615 mg, 3.75 mmol), K_2CO_3 (415 mg, 3.00 mmol) and acetonitrile (10 mL). Reaction time: 14 h. Purification *via* flash chromatography (PE:Et_2O, 1:1) afforded the title compound **S8** as a colourless solid (527 mg, 1.94 mmol, 77%).

m.p. 95-96 °C ($CHCl_3$).

^{1}H NMR (400 MHz, $CDCl_3$) δ = 6.85 (d, *J* = 8.2 Hz, 1H), 6.73 (dd, *J* = 8.2, 2.0 Hz, 1H), 6.36 (d, *J* = 2.0 Hz, 1H), 5.95-5.82 (m, 1H), 5.03 (t, *J* = 1.4 Hz, 1H), 5.01-4.97 (m, 1H), 3.89 (s, 3H), 3.23 (d, *J* = 6.6 Hz, 2H), 2.58-2.48 (m, 4H), 2.10-2.00 (m, 2H), 1.92 (s, 3H).

^{13}C NMR (101 MHz, $CDCl_3$) δ = 192.5, 148.1, 147.2, 146.8, 144.5, 137.7, 132.4, 121.9, 115.6, 113.8, 112.7, 56.4, 39.6, 38.6, 31.8, 22.3, 18.1.

FTIR (neat) ν/cm^{-1} = 2931, 2835, 1681, 1639, 1586, 1510, 1426, 1376, 1329, 1304, 1260, 1227, 1190, 1152, 1029, 995, 970, 868, 807.

HRMS (ESI) *m/z* calcd. for $C_{17}H_{21}O_3$ $[M+H]^+$: 273.1485; found: 273.1484.

2-(4-Allyl-2-methoxyphenoxy)-3-methylcyclohex-2-en-1-one (S9)

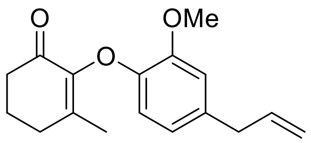

Prepared according to **General Procedure B** with 2,3-epoxy-3-methylcyclohexanone (315 mg, 2.50 mmol), eugenol (615 mg, 3.75 mmol), K_2CO_3 (415 mg, 3.00 mmol) and acetonitrile (10 mL). Reaction time: 14 h. Purification *via* flash chromatography (PE:Et_2O, 1:1) afforded the title compound **S9** as a colourless solid (450 mg, 1.65 mmol, 66%).

m.p. 63-64 °C ($CHCl_3$).

^{1}H NMR (400 MHz, $CDCl_3$) δ = 6.75 (d, *J* = 1.8 Hz, 1H), 6.58 (dd, *J* = 8.1, 1.8 Hz, 1H), 6.48 (d, *J* = 8.1 Hz, 1H), 5.93 (ddt, *J* = 16.9, 10.1, 6.8 Hz, 1H), 5.11-5.00 (m, 2H), 3.90 (s, 3H), 3.31 (d, *J* = 6.7 Hz, 2H), 2.56-2.46 (m, 4H), 2.10-2.00 (m, 2H), 1.92 (s, 3H).

^{13}C NMR (101 MHz, $CDCl_3$) δ = 192.6, 148.6, 148.2, 145.3, 144.5, 137.7, 133.9, 120.3, 115.8, 113.1 (2C), 56.2, 40.0, 38.6, 31.8, 22.2, 18.1.

FTIR (neat) ν/cm^{-1} = 3001, 2936, 1681, 1638, 1595, 1506, 1464, 1452, 1419, 1377, 1347, 1262, 1218, 1188, 1153, 1033, 995, 930, 877, 810, 748.

HRMS (ESI) *m/z* calcd. for $C_{17}H_{20}O_3Na$ $[M+Na]^+$: 295.1305; found: 295.1302.

4-((2-Methyl-6-oxocyclohex-1-en-1-yl)oxy)-2,3-dihydro-1*H*-inden-1-one (S10)

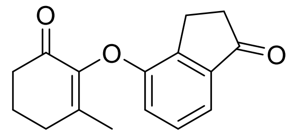

Prepared according to **General Procedure B** with 2,3-epoxy-3-methylcyclohexanone (315 mg, 2.50 mmol), 4-hydroxy-1-indanone (555 mg, 3.75 mmol), K_2CO_3 (415 mg, 3.00 mmol) and acetonitrile (10 mL). Reaction time: 14 h. Purification *via* flash chromatography (PE:EtOAc, 2:1 → 1:1) afforded the title compound **S10** as a colourless solid (476 mg, 1.86 mmol, 74%).

m.p. 126-128 °C ($CHCl_3$).

^{1}H NMR (400 MHz, $CDCl_3$) δ = 7.48 (d, *J* = 7.5 Hz, 1H), 7.22 (dd, *J* = 7.7, 7.7 Hz, 1H), 6.72 (dd, *J* = 7.9, 0.7 Hz, 1H), 3.24-3.15 (m, 2H), 2.77-2.68 (m, 2H), 2.62-2.51 (m, 4H), 2.16-2.05 (m, 2H), 1.96 (s, 3H).

^{13}C NMR (101 MHz, $CDCl_3$) δ = 207.2, 192.5, 155.1, 149.0, 144.3, 143.6, 139.3, 128.6, 117.3, 116.9, 38.4, 36.3, 31.7, 22.6, 22.1, 18.0.

FTIR (neat) ν/cm^{-1} = 2926, 1709, 1679, 1641, 1591, 1476, 1456, 1437, 1377, 1348, 1328, 1303, 1285, 1263, 1250, 1190, 1177, 1145, 1131, 1047, 1030, 951, 820, 777, 729.

HRMS (ESI) *m/z* calcd. for $C_{16}H_{17}O_3$ $[M+H]^+$: 257.1172; found: 257.1171.

3-Methyl-2-(*m*-tolyloxy)cyclohex-2-en-1-one (S11)

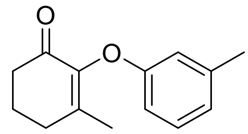

Prepared according to **General Procedure B** with 2,3-epoxy-3-methylcyclohexanone (315 mg, 2.5 mmol), *m*-cresol (405 mg, 3.75 mmol), K_2CO_3 (415 mg, 3.0 mmol) and acetonitrile (10 mL). Reaction time: 16 h. Purification *via* flash chromatography (PE:Et_2O, 1:1) afforded the title compound **S11** as a colourless oil (417 mg, 1.93 mmol, 77%).

^{1}H NMR (400 MHz, $CDCl_3$) δ = 7.12 (dd, *J* = 7.8, 7.8 Hz, 1H), 6.78 (d, *J* = 7.5 Hz, 1H), 6.67 (s, 1H), 6.61 (dd, *J* = 8.2, 2.4 Hz, 1H), 2.58–2.51 (m, 4H), 2.30 (s, 3H), 2.12–2.04 (m, 2H), 1.90 (s, 3H).

^{13}C NMR (101 MHz, $CDCl_3$) δ = 193.1, 157.6, 148.7, 144.3, 139.7, 129.3, 122.6, 115.5, 111.4, 38.5, 31.7, 22.2, 21.6, 18.0.

FTIR (neat) ν/cm^{-1} = 2921, 1683, 1641, 1607, 1586, 1487, 1431, 1377, 1348, 1327, 1305, 1282, 1256, 1192, 1157, 1126, 1030, 938, 859, 777.

HRMS (EI) *m/z* calcd. for $C_{14}H_{16}O_2$ $[M]^+$: 216.1150; found: 216.1150

2-(3-(Dimethylamino)phenoxy)-3-methylcyclohex-2-en-1-one (S12)

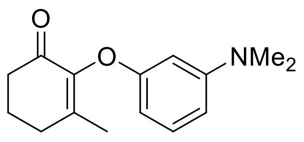

Prepared according to **General Procedure B** with 2,3-epoxy-3-methylcyclohexanone (631 mg, 5.00 mmol), 3-(dimethylamino)phenol (1.03 g, 7.50 mmol), K_2CO_3 (829 mg, 6.00 mmol) and acetonitrile (20 mL). Reaction time: 16 h. Purification *via* flash chromatography (PE:Et_2O, 1:1) afforded the title compound **S12** as a colourless solid (638 mg, 2.60 mmol, 52%).

m.p. 53-54 °C ($CHCl_3$).

^1H NMR (400 MHz, $CDCl_3$) δ = 7.10-7.01 (m, 1H), 6.40-6.30 (m, 2H), 6.10-6.04 (m, 1H), 2.92 (s, 6H), 2.58-2.48 (m, 4H), 2.12-2.02 (m, 2H), 1.91 (s, 3H).

^{13}C NMR (101 MHz, $CDCl_3$) δ = 193.2, 158.7, 152.2, 148.6, 144.4, 129.7, 106.6, 101.8, 99.9, 40.6, 38.6, 31.8, 22.3, 18.1.

FTIR (neat) ν/cm^{-1} = 2922, 1683, 1639, 1610, 1572, 1501, 1449, 1377, 1350, 1325, 1303, 1232, 1188, 1153, 999, 823, 755.

HRMS (CI) *m/z* calcd. for $C_{15}H_{20}NO_3$ $[M+H]^+$: 246.1494; found: 246.1496.

2-(3,5-Dimethoxyphenoxy)-3-methylcyclohex-2-en-1-one (S13)

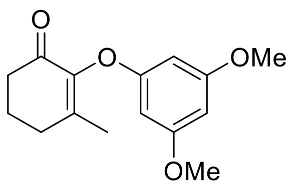

Prepared according to **General Procedure B** with 2,3-epoxy-3-methylcyclohexanone (631 mg, 5.00 mmol), 3,5-dimethoxyphenol (1.16 g, 7.50 mmol), K_2CO_3 (829 mg, 6.00 mmol) and acetonitrile (20 mL). Reaction time: 16 h. Purification *via* flash chromatography (PE:Et_2O, 1:1) afforded the title compound **S13** as a colourless solid (762 mg, 2.90 mmol, 58%).

m.p. 54-55 °C ($CHCl_3$).

^1H NMR (400 MHz, $CDCl_3$) δ = 6.09 (dd, J = 2.0, 2.0 Hz, 1H), 6.02 (d, J = 2.0 Hz, 2H), 3.75 (s, 6H), 2.56-2.47 (m, 4H), 2.11-2.00 (m, 2H), 1.89 (s, 3H).

^{13}C NMR (101 MHz, $CDCl_3$) δ = 192.9, 161.6, 159.4, 149.1, 144.1, 93.8, 93.7, 55.4, 38.5, 31.7, 22.2, 18.0.

FTIR (neat) ν/cm^{-1} = 2952, 1682, 1596, 1475, 1377, 1304, 1196, 1152, 1063, 928, 822, 681.

HRMS (CI) *m/z* calcd. for $C_{15}H_{19}O_4$ $[M+H]^+$: 263.1283; found: 263.1285.

2-(3,5-Bis(trifluoromethyl)phenoxy)-3-methylcyclohex-2-en-1-one (S14)

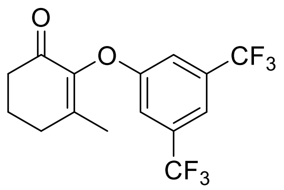

Prepared according to **General Procedure B** with 2,3-epoxy-3-methylcyclohexanone (315 mg, 2.50 mmol), 3,5-bis(trifluoromethyl)phenol (615 mg, 3.75 mmol), K_2CO_3 (415 mg, 3.00 mmol) and acetonitrile (10 mL). Reaction time: 16 h. Purification *via* flash chromatography (PE:Et_2O, 2:1) afforded the title compound **S14** as a colourless solid (450 mg, 1.33 mmol, 53%).

m.p. 122-124 °C ($CHCl_3$).

^{1}H NMR (400 MHz, $CDCl_3$) δ = 7.48 (s, 1H), 7.22 (s, 2H), 2.64-2.53 (m, 4H), 2.16-2.06 (m, 2H), 1.94 (s, 3H).

^{13}C NMR (101 MHz, $CDCl_3$) δ = 192.1, 158.3, 150.1, 143.4, 133.0 (q, J = 33.3 Hz), 123.2 (q, J = 275 Hz), 115.6, 115.2, 38.3, 31.7, 22.1, 18.0.

^{19}F NMR (377 MHz, $CDCl_3$) δ = −62.9.

FTIR (neat) ν/cm^{-1} = 1686, 1648, 1611, 1461, 1375, 1278, 1173, 1128, 949, 879, 682, 669.

HRMS (ESI) *m/z* calcd. for $C_{15}H_{13}O_2F_6$ [M+H]$^+$: 339.0820; found: 339.0821.

3-Methyl-2-(pyridin-4-yloxy)cyclohex-2-en-1-one (S15)

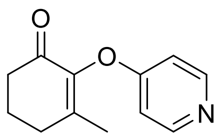

Prepared according to **General Procedure B** with 2,3-epoxy-3-methylcyclohexanone (631 mg, 5.00 mmol), 4-hydroxypyridine (523 mg, 5.50 mmol), K_2CO_3 (726 mg, 5.25 mmol) and acetonitrile (20 mL). Reaction time: 14 h. Purification *via* flash chromatography (CH_2Cl_2:MeOH, 30:1) afforded the title compound **S15** as a colourless solid (156 mg, 0.77 mmol, 15%).

m.p. 56-58 °C ($CHCl_3$).

^{1}H NMR (400 MHz, $CDCl_3$) δ = 8.41 (d, J = 5.1 Hz, 2H), 6.74 (d, J = 5.5 Hz, 2H), 2.61-2.52 (m, 4H), 2.14-2.04 (m, 2H), 1.89 (s, 3H).

^{13}C NMR (101 MHz, $CDCl_3$) δ = 192.1, 163.8, 151.4, 149.7, 143.2, 110.6, 38.3, 31.7, 22.1, 18.0.

FTIR (neat) ν/cm^{-1} = 1947, 1680, 1644, 1584, 1494, 1455, 1417, 1377, 1349, 1329, 1303, 1261, 1207, 1189, 1144, 1126, 1030, 992, 930, 886.

HRMS (ESI) *m/z* calcd. for $C_{12}H_{14}NO_2$ [M+H]$^+$: 204.1019; found: 204.1019.

4-((2-Methyl-6-oxocyclohex-1-en-1-yl)oxy)benzaldehyde (S16)

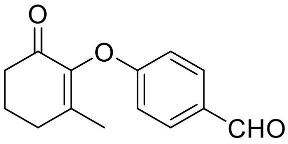

Prepared according to **General Procedure B** with 2,3-epoxy-3-methylcyclohexanone (631 mg, 5.00 mmol), 4-hydroxybenzaldehyde (916 mg, 7.50 mmol), K_2CO_3 (829 mg, 6.00 mmol) and acetonitrile (20 mL). Reaction time: 14 h. Purification *via* flash chromatography (PE:Et_2O, 1:2 → 1:3) afforded the title compound **S16** as a colourless solid (892 mg, 3.87 mmol, 77%).

m.p. 77-79 °C ($CHCl_3$).

^{1}H NMR (400 MHz, $CDCl_3$) δ = 9.88 (s, 1H), 7.80 (d, *J* = 8.7 Hz, 2H), 6.94 (d, *J* = 8.7 Hz, 2H), 2.62-2.52 (m, 4H), 2.15-2.05 (m, 2H), 1.91 (s, 3H).

^{13}C NMR (101 MHz, $CDCl_3$) δ = 192.3, 190.9, 162.6, 149.5, 143.7, 132.1, 131.0, 115.3, 38.3, 31.7, 22.1, 18.0.

FTIR (neat) ν/cm^{-1} = 2944, 1680, 1642, 1596, 1582, 1504, 1428, 1377, 1348, 1305, 1234, 1188, 1155, 1127, 1029, 930, 883, 834, 785.

HRMS (ESI) *m/z* calcd. for $C_{14}H_{14}O_3Na$ $[M+Na]^+$: 253.0835; found: 253.0836.

Ethyl 4-((2-methyl-6-oxocyclohex-1-en-1-yl)oxy)benzoate (S17)

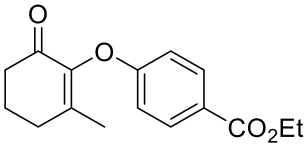

Prepared according to **General Procedure B** with 2,3-Epoxy-3-methylcyclohexanone (631 mg, 5.00 mmol), Ethyl 4-hydroxybenzoate (1.25 g, 7.50 mmol), K_2CO_3 (829 mg, 6.00 mmol) and acetonitrile (20 mL). Reaction time: 14 h. Purification *via* flash chromatography (PE:Et_2O, 1:1) afforded the title compound **S17** as a colourless solid (1.24 g, 4.51 mmol, 90%).

m.p. 55-65 °C ($CHCl_3$).

^{1}H NMR (400 MHz, $CDCl_3$) δ = 7.96 (d, *J* = 8.9 Hz, 2H), 6.85 (d, *J* = 8.9 Hz, 2H), 4.33 (q, *J* = 7.1 Hz, 2H), 2.60–2.51 (m, 4H), 2.14–2.04 (m, 2H), 1.90 (s, 3H), 1.36 (t, *J* = 7.1 Hz, 3H).

^{13}C NMR (101 MHz, $CDCl_3$) δ = 192.5, 166.3, 161.3, 149.2, 143.9, 131.7, 124.1, 114.5, 60.8, 38.4, 31.7, 22.1, 18.0, 14.5.

FTIR (neat) ν/cm^{-1} = 2981, 1710, 1682, 1644, 1602, 1505, 1456, 1377, 1367, 1327, 1306, 1273, 1236, 1187, 1161, 1145, 1100, 930, 883, 851, 771.

HRMS (ESI) *m/z* calcd. for $C_{16}H_{19}O_4$ $[M+H]^+$: 275.1278; found: 275.1276.

4-((2-Methyl-6-oxocyclohex-1-en-1-yl)oxy)benzonitrile (S18)

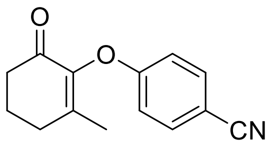

Prepared according to **General Procedure B** with 2,3-epoxy-3-methylcyclohexanone (315 mg, 2.50 mmol), 4-hydroxybenzonitrile (447 mg, 3.75 mmol), K_2CO_3 (415 mg, 3.00 mmol) and acetonitrile (10 mL). Reaction time: 14 h. Purification *via* flash chromatography (PE:Et_2O, 1:2) afforded the title compound **S18** as a colourless solid (428 mg, 1.88 mmol, 75%).

m.p. 118-120 °C ($CHCl_3$).

^{1}H NMR (400 MHz, $CDCl_3$) δ = 7.55 (d, *J* = 8.4 Hz, 2H), 6.89 (d, *J* = 8.6 Hz, 2H), 2.61-2.51 (m, 4H), 2.14-2.04 (m, 2H), 1.91 (s, 3H).

^{13}C NMR (101 MHz, $CDCl_3$) δ = 192.2, 160.9, 149.7, 143.5, 134.2, 119.2, 115.7, 105.2, 38.3, 31.7, 22.1, 18.0.

FTIR (neat) ν/cm^{-1} = 2952, 2221, 1668, 1644, 1602, 1498, 1455, 1419, 1378, 1330, 1305, 1241, 1192, 1168, 1144, 1126, 1030, 929, 886, 841, 715.

HRMS (ESI) *m/z* calcd. for $C_{14}H_{14}NO_2$ $[M+H]^+$: 228.1019; found: 228.1021.

2-(4-Methoxyphenoxy)-3-methylcyclohex-2-en-1-one (S19)

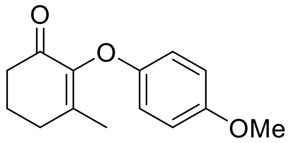

Prepared according to **General Procedure B** with 2,3-epoxy-3-methylcyclohexanone (631 mg, 5.00 mmol), 4-methoxyphenol (931 mg, 7.50 mmol), K_2CO_3 (829 mg, 6.00 mmol) and acetonitrile (20 mL). Reaction time: 14 h. Purification via flash chromatography (PE:Et_2O, 1:1) afforded the title compound **S19** as a colourless oil (998 mg, 4.30 mmol, 86%).

^{1}H NMR (400 MHz, $CDCl_3$) δ = 6.82–6.71 (m, 4H), 3.74 (s, 3H), 2.57-2.46 (m, 4H), 2.11-2.00 (m, 2H), 1.91 (s, 3H).

^{13}C NMR (101 MHz, $CDCl_3$) δ = 193.3, 154.5, 151.8, 148.6, 144.8, 115.5, 114.7, 55.8, 38.6, 31.7, 22.2, 18.1.

FTIR (neat) ν/cm^{-1} = 2948, 1678, 1639, 1502, 1456, 1442, 1377, 1348, 1242, 1209, 1185, 1164, 1129, 1104, 1032, 882, 754, 742.

HRMS (ESI) *m/z* calcd. for $C_{14}H_{17}O_3$ $[M+H]^+$: 233.1172; found: 233.1171.

2-(4-(Benzyloxy)phenoxy)-3-methylcyclohex-2-en-1-one (S20)

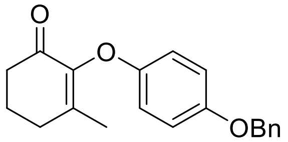

Prepared according to **General Procedure B** with 2,3-epoxy-3-methylcyclohexanone (315 mg, 2.50 mmol), 4-benzyloxyphenol (751 mg, 3.75 mmol), K_2CO_3 (415 mg, 3.00 mmol) and acetonitrile (10 mL). Reaction time: 14 h. Purification *via* flash chromatography (PE:Et_2O, 1:1→1:2) and subsequent recrystallization from *n*-hexane/EtOAc afforded the title compound **S20** as a colourless solid (347 mg, 1.12 mmol, 45%).

m.p. 127-129 °C ($CHCl_3$).

^{1}H NMR (400 MHz, $CDCl_3$) δ = 7.46-7.28 (m, 5H), 6.87 (d, J = 9.0 Hz, 2H), 6.77 (d, J = 9.0 Hz, 2H), 5.00 (s, 2H), 2.56-2.49 (m, 4H), 2.11-2.01 (m, 2H), 1.91 (s, 3H)

^{13}C NMR (101 MHz, $CDCl_3$) δ = 193.3, 153.7, 151.9, 148.7, 144.8, 137.4, 128.7, 128.0, 127.6, 115.8, 115.5, 70.7, 38.6, 31.7, 22.2, 18.1.

FTIR (neat) ν/cm^{-1} = 2949, 1680, 1500, 1454, 1377, 1304, 1205, 1130, 1025, 930, 826, 742, 698.

HRMS (ESI) *m/z* calcd. for $C_{20}H_{20}O_3Na$ [M+Na]$^+$: 331.1305; found: 331.1301.

***N*-(4-((2-Methyl-6-oxocyclohex-1-en-1-yl)oxy)phenyl)acetamide (S21)**

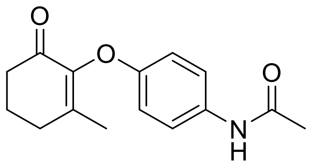

Prepared according to **General Procedure B** with 2,3-epoxy-3-methylcyclohexanone (315 mg, 2.50 mmol), 4-acetamidophenol (567 mg, 3.75 mmol), K_2CO_3 (415 mg, 3.00 mmol) and acetonitrile (10 mL). Reaction time: 20 h. Purification *via* flash chromatography (PE:EtOAc, 1:3) afforded the title compound **S21** as a colourless solid (408 mg, 1.57 mmol, 63%).

m.p. 137-139 °C ($CHCl_3$).

^{1}H NMR (400 MHz, $CDCl_3$) δ = 7.77 (br, 1H), 7.25 (d, J = 9.0 Hz, 2H), 6.70 (d, J = 9.0 Hz, 2H), 2.58-2.50 (m, 4H), 2.12-2.03 (m, 5H), 1.89 (s, 3H).

^{13}C NMR (101 MHz, $CDCl_3$) δ = 193.6, 168.6, 154.0, 149.7, 144.3, 132.3, 121.9, 114.8, 38.5, 31.7, 24.3, 22.2, 18.1.

FTIR (neat) ν/cm^{-1} = 3311, 2948, 1666, 1608, 1543, 1505, 1410, 1375, 1307, 1215, 1168, 1145, 1131, 1030, 930, 883, 832, 794.

HRMS (ESI) *m/z* calcd. for $C_{15}H_{18}O_3N$ [M+H]$^+$: 260.1281; found: 260.1279.

3-Methyl-2-(4-(methylthio)phenoxy)cyclohex-2-en-1-one (S22)

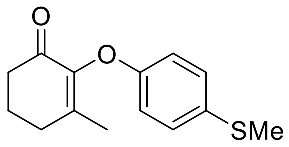

Prepared according to **General Procedure B** with 2,3-epoxy-3-methylcyclohexanone (315 mg, 2.50 mmol), 4-(mercaptomethyl)phenol (526 mg, 3.75 mmol), K_2CO_3 (415 mg, 3.00 mmol) and acetonitrile (10 mL). Reaction time: 16 h. Purification *via* flash chromatography (PE:Et_2O, 3:1) afforded the title compound **S22** as a colourless solid (505 mg, 2.03 mmol, 81%).

m.p. 45-47 °C ($CHCl_3$).

^{1}H NMR (400 MHz, $CDCl_3$) δ = 7.21 (d, *J* = 8.8 Hz, 2H), 6.78 (d, *J* = 8.8 Hz, 2H), 2.57-2.49 (m, 4H), 2.42 (s, 3H), 2.11-2.02 (m, 2H), 1.90 (s, 3H).

^{13}C NMR (101 MHz, $CDCl_3$) δ = 193.0, 156.2, 149.0, 144.2, 130.1, 115.4, 38.5, 31.7, 22.2, 18.0, 17.9.

FTIR (neat) ν/cm^{-1} = 2920, 1680, 1640, 1590, 1487, 1429, 1377, 1303, 1274, 1227, 1169, 1130, 930, 882, 824.

HRMS (ESI) *m/z* calcd. for $C_{14}H_{16}O_2SNa$ [M+Na]$^+$: 271.0763; found: 271.0762.

3-Methyl-2-(*p*-tolyloxy)cyclohex-2-en-1-one (S23)

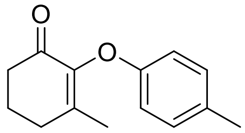

Prepared according to **General Procedure B** with 2,3-epoxy-3-methylcyclohexanone (315 mg, 2.50 mmol), *p*-cresol (405 mg, 3.75 mmol), K_2CO_3 (415 mg, 3.00 mmol) and acetonitrile (10 mL). Reaction time: 14 h. Purification *via* flash chromatography (PE:Et_2O, 2:1) afforded the title compound **S23** as a colourless oil (470 mg, 2.17 mmol, 87%).

^{1}H NMR (400 MHz, $CDCl_3$) δ = 7.04 (d, *J* = 8.3 Hz, 2H), 6.73 (d, *J* = 8.5 Hz, 2H), 2.58-2.49 (m, 4H), 2.27 (s, 3H), 2.12-2.02 (m, 2H), 1.90 (s, 3H).

^{13}C NMR (101 MHz, $CDCl_3$) δ = 193.2, 155.6, 148.6, 144.5, 131.0, 130.1, 114.5, 38.6, 31.7, 22.2, 20.7, 18.1.

FTIR (neat) ν/cm^{-1} = 2951, 1680, 1640, 1608, 1588, 1504, 1454, 1430, 1412, 1377, 1347, 1327, 1302, 1220, 1187, 1168, 1145, 1129, 1105, 1059, 1028, 829, 881, 747.

HRMS (ESI) *m/z* calcd. for $C_{14}H_{17}O_2$ [M+H]$^+$: 217.1223; found: 217.1221.

3,5,5-Trimethyl-2-(*p*-tolyloxy)cyclohex-2-en-1-one (S24)

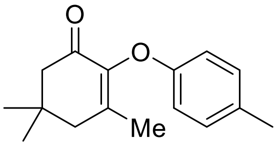

Prepared according to **General Procedure B** with 2,3-epoxyisophorone (771 mg, 5.00 mmol), *p*-cresol (811 mg, 7.50 mmol), K_2CO_3 (829 mg, 6.00 mmol) and acetonitrile (20 mL). Reaction time: 14 h. Purification *via* flash chromatography (PE:Et_2O, 4:1) afforded the title compound **S24** as a colourless solid (373 mg, 1.53 mmol, 31%). The spectral data matched that previously reported in the literature.[19]

m.p. 80-82 °C ($CHCl_3$) (Lit: 83-84 °C).[19]

^{1}H NMR (400 MHz, $CDCl_3$) δ = 7.04 (d, *J* = 8.2 Hz, 2H), 6.73 (d, *J* = 8.5 Hz, 2H), 2.42 (s, 2H), 2.40 (s, 2H), 2.27 (s, 3H), 1.88 (s, 3H), 1.14 (s, 6H).

^{13}C NMR (101 MHz, $CDCl_3$) δ = 193.1, 155.7, 145.8, 143.9, 131.0, 130.1, 114.7, 52.0, 45.7, 33.3, 28.6, 20.7, 18.2.

FTIR (neat) ν/cm^{-1} = 2958, 1683, 1645, 1609, 1505, 1466, 1377, 1315, 1224, 1197, 1178, 1112, 977, 818.

HRMS (ESI) *m/z* calcd. for $C_{16}H_{20}O_2Na$ [M+Na]$^+$: 267.1355; found: 267.1352.

3-Ethyl-2-(*p*-tolyloxy)cyclohex-2-en-1-one (S25)

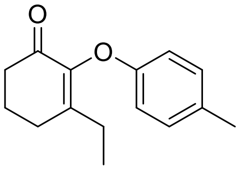

Prepared according to **General Procedure B** with 2,3-epoxy-3-ethylcyclohexanone (**S2a**) (350 mg, 2.50 mmol), *p*-cresol (405 mg, 3.75 mmol), K_2CO_3 (415 mg, 3.00 mmol) and acetonitrile (10 mL). Reaction time: 14 h. Purification *via* flash chromatography (PE:Et_2O, 1:1) afforded the title compound **S25** as a yellow oil (380 mg, 1.65 mmol, 66%).

^{1}H NMR (400 MHz, $CDCl_3$) δ = 7.03 (d, *J* = 8.1 Hz, 2H), 6.73 (d, *J* = 8.6 Hz, 2H), 2.57-2.49 (m, 4H), 2.30 (q, *J* = 7.8 Hz, 2H), 2.27 (s, 3H), 2.12-2.02 (m, 2H), 1.05 (t, *J* = 7.6 Hz, 3H).

^{13}C NMR (101 MHz, $CDCl_3$) δ = 193.4, 155.9, 153.5, 143.7, 130.9, 130.0, 114.5, 38.6, 29.2, 24.8, 22.5, 20.6, 11.7.

FTIR (neat) ν/cm^{-1} = 2937, 1681, 1634, 1608, 1505, 1457, 1350, 1329, 1301, 1287, 1262, 1221, 1182, 1168, 1127, 1106, 1072, 926, 905, 873, 814.

HRMS (ESI) *m/z* calcd. for $C_{15}H_{18}O_2Na$ [M+Na]$^+$: 253.1199; found: 253.1199.

3-Isopropyl-2-(*p*-tolyloxy)cyclohex-2-en-1-one (S26)

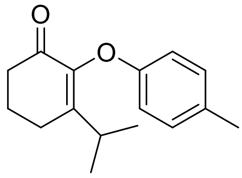

Prepared according to **General Procedure B** with 2,3-epoxy-3-isopropylcyclohexanone (**S2b**) (290 mg, 1.88 mmol), *p*-cresol (305 mg, 2.82 mmol), K_2CO_3 (312 mg, 2.26 mmol) and acetonitrile (8 mL). Reaction time: 20 h. Purification *via* flash chromatography (PE:Et_2O, 3:1→2:1) afforded the title compound **S26** as a yellowish solid (282 mg, 1.15 mmol, 61%).

m.p. 40-41 °C ($CHCl_3$).

^{1}H NMR (400 MHz, $CDCl_3$) δ = 7.04 (d, *J* = 8.2 Hz, 2H), 6.73 (d, *J* = 8.6 Hz, 2H), 3.14 (sep, *J* = 6.9 Hz, 1H), 2.56-2.45 (m, 4H), 2.27 (s, 3H), 2.10-2.00 (m, 2H), 1.03 (d, *J* = 6.9 Hz, 6H).

^{13}C NMR (101 MHz, $CDCl_3$) δ = 193.6, 156.8, 155.9, 142.7, 130.9, 130.0, 114.4, 38.6, 28.4, 24.5, 22.6, 20.6, 20.0.

FTIR (neat) ν/cm^{-1} = 2964, 2871, 1683, 1627, 1505, 1454, 1349, 1325, 1305, 1222, 1168, 1141, 1114, 950, 897, 816.

HRMS (ESI) *m/z* calcd. for $C_{16}H_{20}O_2Na$ $[M+Na]^+$: 267.1355; found: 267.1354.

(*E*)-3-(Prop-1-en-1-yl)-2-(*p*-tolyloxy)cyclohex-2-en-1-one (56)

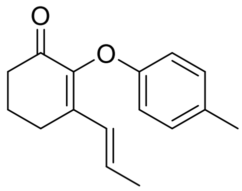

Prepared according to **General Procedure B** with 2,3-epoxy-3-allylcyclohexanone (**S2d**) (380 mg, 2.50 mmol), *p*-cresol (405 mg, 3.75 mmol), K_2CO_3 (415 mg, 3.00 mmol) and acetonitrile (10 mL). Reaction time: 20 h. Purification *via* flash chromatography (PE:Et_2O, 2:1) afforded the title compound **56** as a yellow solid (475 mg, 1.96 mmol, 78%, >20:1 d.r.).

m.p. 56-57 °C ($CHCl_3$).

^{1}H NMR (400 MHz, $CDCl_3$) δ = 7.05 (d, *J* = 8.1 Hz, 2H), 6.76 (d, *J* = 8.6 Hz, 2H), 6.65 (dd, *J* = 15.9, 1.9 Hz, 1H), 6.24 (dq, *J* = 15.9, 6.8 Hz, 1H), 2.67 (t, *J* = 6.1 Hz, 2H), 2.54 (dd, *J* = 7.3, 6.0 Hz, 2H), 2.27 (s, 3H), 2.14-2.05 (m, 2H), 1.86 (dd, *J* = 6.8, 1.6 Hz, 3H).

^{13}C NMR (101 MHz, $CDCl_3$) δ = 193.8, 156.0, 143.8, 142.7, 135.0, 131.2, 130.1, 126.3, 114.8, 38.6, 25.3, 22.0, 20.7, 19.5.

FTIR (neat) ν/cm^{-1} = 2943, 1676, 1633, 1612, 1585, 1505, 1443, 1415, 1377, 1365, 1352, 1330, 1299, 1191, 1193, 1124, 1106, 1093, 971, 923, 914, 814.

HRMS (ESI) *m/z* calcd. for $C_{16}H_{18}O_2Na$ $[M+Na]^+$: 265.1199; found: 265.1199.

2-(*p*-Tolyloxy)-5,6-dihydro-[1,1'-biphenyl]-3(4*H*)-one (58)

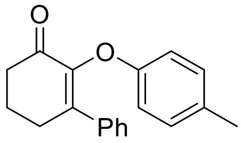

Prepared according to **General Procedure B** with 2,3-epoxy-3-phenylcyclohexanone (470 mg, 2.50 mmol), *p*-cresol (405 mg, 3.75 mmol), K_2CO_3 (415 mg, 3.00 mmol) and acetonitrile (10 mL). Reaction time: 16 h. Purification *via* flash chromatography (PE:Et_2O, 2:1) afforded the title compound **58** as a yellow oil (625 mg, 2.24 mmol, 90%).

^{1}H NMR (400 MHz, $CDCl_3$) δ = 7.52-7.45 (m, 2H), 7.36-7.28 (m, 3H), 7.01 (d, *J* = 8.6 Hz, 2H), 6.73 (d, *J* = 8.4 Hz, 2H), 2.93 (t, *J* = 6.0 Hz, 2H), 2.65 (d, *J* = 6.7 Hz, 2H), 2.25 (s, 3H), 2.25-2.17 (m, 2H).

^{13}C NMR (101 MHz, $CDCl_3$) δ = 194.0, 155.7, 146.4, 143.8, 136.7, 131.1, 130.0, 129.1, 128.4, 127.8, 115.0, 38.8, 31.1, 22.7, 20.7.

FTIR (neat) ν/cm^{-1} = 2947, 1681, 1605, 1505, 1444, 1414, 1351, 1328, 1302, 1277, 1217, 1191, 1169, 1139, 1125, 1106, 1090, 1035, 1014, 915, 849, 820, 771, 695.

HRMS (ESI) *m/z* calcd. for $C_{19}H_{18}O_2Na$ $[M+Na]^+$: 301.1199; found: 301.1195.

3-Cyclopropyl-2-(*p*-tolyloxy)cyclohex-2-en-1-one (59)

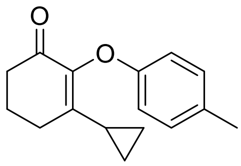

Prepared according to **General Procedure B** with 2,3-epoxy-3-cyclopropylcyclohexanone (**S2c**) (304 mg, 2.00 mmol), *p*-cresol (324 mg, 3.00 mmol), K_2CO_3 (332 mg, 2.40 mmol) and acetonitrile (8 mL). Reaction time: 20 h. Purification *via* flash chromatography (PE:Et_2O, 2:1) afforded the title compound **59** as a green oil (424 mg, 1.75 mmol, 87%).

^{1}H NMR (400 MHz, $CDCl_3$) δ = 7.05 (d, *J* = 8.2 Hz, 2H), 6.79 (d, *J* = 8.6 Hz, 2H), 2.51 (dd, *J* = 6.9, 5.8 Hz, 2H), 2.27 (s, 3H), 2.16-1.95 (m, 5H), 0.92-0.74 (m, 4H).

^{13}C NMR (101 MHz, $CDCl_3$) δ = 192.2, 155.8, 153.4, 144.9, 130.9, 130.0, 114.6, 38.4, 23.7, 22.0, 20.7, 12.1, 6.9.

FTIR (neat) ν/cm^{-1} = 3010, 2947, 1672, 1605, 1505, 1453, 1424, 1374, 1347, 1328, 1309, 1237, 1216, 1167, 1139, 1114, 1046, 1009, 963, 903, 814, 748, 666.

HRMS (ESI) *m/z* calcd. for $C_{16}H_{18}O_2Na$ $[M+Na]^+$: 265.1199; found: 265.1198.

2-Phenoxycyclohex-2-en-1-one (S28)

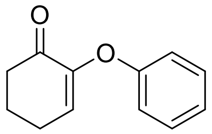

Prepared according to **General Procedure B** with 2,3-epoxycyclohexanone (561 mg, 5.00 mmol), phenol (706 mg, 7.50 mmol), K_2CO_3 (829 mg, 6.00 mmol) and acetonitrile (20 mL). Reaction time: 15 h. Purification *via* flash chromatography (PE:Et_2O, 2:1) afforded the title compound **S28** as a colourless solid (601 mg, 3.19 mmol, 64%). The spectral data matched that previously reported in the literature.[20]

m.p. 39-41 °C ($CHCl_3$).

^{1}H NMR (400 MHz, $CDCl_3$) δ = 7.35-7.28 (m, 2H), 7.11-7.04 (m, 1H), 6.99-6.92 (m, 2H), 6.27 (t, *J* = 4.5 Hz, 1H), 2.60 (dd, *J* = 6.7, 6.7 Hz, 2H), 2.51-2.42 (m, 2H), 2.13-2.03 (m, 2H).

^{13}C NMR (101 MHz, $CDCl_3$) δ = 193.9, 156.4, 150.1, 129.7, 129.7, 123.5, 118.3, 38.9, 24.9, 23.0.

FTIR (neat) ν/cm^{-1} = 2949, 1689, 1629, 1591, 1490, 1456, 1432, 1353, 1290, 1249, 1219, 1164, 1141, 1109, 1074, 979, 910, 804, 756.

HRMS (ESI) *m/z* calcd. for $C_{12}H_{12}O_2Na$ $[M+Na]^+$: 211.0729; found: 211.0730.

2-(*p*-Tolyloxy)cyclohex-2-en-1-one (S29)

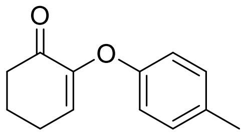

Prepared according to **General Procedure B** with 2,3-epoxycyclohexanone (561 mg, 5.00 mmol), *p*-cresol (811 mg, 7.50 mmol), K_2CO_3 (829 mg, 6.00 mmol) and acetonitrile (20 mL). Reaction time: 14 h. Purification *via* flash chromatography (PE:Et_2O, 2:1) afforded the title compound **S29** as a colourless solid (767 mg, 3.79 mmol, 76%).

m.p. 69-70 °C ($CHCl_3$).

^{1}H NMR (400 MHz, $CDCl_3$) δ = 7.10 (d, *J* = 8.4 Hz, 2H), 6.85 (d, *J* = 8.4 Hz, 2H), 6.15 (t, *J* = 4.5 Hz, 1H), 2.58 (dd, *J* = 6.9, 6.9 Hz, 2H), 2.46-2.39 (m, 2H), 2.30 (s, 3H), 2.09-2.00 (m, 2H).

^{13}C NMR (101 MHz, $CDCl_3$) δ = 194.0, 153.8, 150.6, 133.2, 130.3, 128.0, 118.7, 38.9, 24.8, 23.0, 20.8.

FTIR (neat) ν/cm^{-1} = 2925, 1688, 1608, 1506, 1433, 1351, 1287, 1249, 1220, 1166, 1140, 1108, 1046, 979, 910, 816.

HRMS (ESI) *m/z* calcd. for $C_{13}H_{14}O_2Na$ $[M+Na]^+$: 225.0886; found: 225.0890.

2-([1,1'-Biphenyl]-4-yloxy)cyclohex-2-en-1-one (S30)

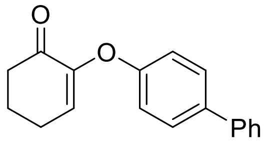

Prepared according to **General Procedure B** with 2,3-epoxycyclohexanone (561 mg, 5.00 mmol), 4-phenylphenol (811 mg, 7.50 mmol), K_2CO_3 (829 mg, 6.00 mmol) and acetonitrile (20 mL). Reaction time: 14 h. Purification *via* flash chromatography (PE:Et_2O, 2:1→1:1) afforded the title compound **S30** as a colourless solid (654 mg, 2.47 mmol, 49%).

m.p. 130-132 °C ($CHCl_3$).

^{1}H NMR (400 MHz, $CDCl_3$) δ = 7.57-7.49 (m, 4H), 7.46-7.39 (m, 2H), 7.35-7.29 (m, 1H), 7.02 (d, *J* = 8.7 Hz, 2H), 6.37 (t, *J* = 4.5 Hz, 1H), 2.61 (dd, *J* = 6.7, 6.7 Hz, 2H), 2.53-2.46 (m, 2H), 2.13-2.04 (m, 2H).

^{13}C NMR (101 MHz, $CDCl_3$) δ = 193.9, 156.0, 149.9, 140.6, 136.5, 130.4, 128.9, 128.5, 127.1, 127.0, 118.4, 39.0, 24.9, 23.0.

FTIR (neat) ν/cm^{-1} = 3032, 2948, 1687, 1628, 1604, 1514, 1484, 1451, 1430, 1413, 1352, 1318, 1287, 1266, 1248, 1223, 1186, 1140, 1108, 1076, 1046, 910, 834, 764, 698.

HRMS (EI) *m/z* calcd. for $C_{18}H_{16}O_3$ $[M]^+$: 264.1150; found: 264.1146.

Ethyl 4-((6-oxocyclohex-1-en-1-yl)oxy)benzoate (S31)

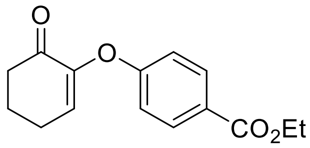

Prepared according to **General Procedure B** with 2,3-epoxycyclohexanone (448 mg, 4.00 mmol), ethyl 4-hydroxybenzoate (997 mg, 6.00 mmol), K_2CO_3 (663 mg, 4.20 mmol) and acetonitrile (16 mL). Reaction time: 18 h. Purification *via* flash column chromatography (PE:Et_2O, 2:1) afforded the title compound **S31** as a colourless solid (540 mg, 2.06 mmol, 51%).

m.p. = 58–60 °C ($CHCl_3$).

^{1}H NMR (400 MHz, $CDCl_3$) δ = 8.06–7.96 (m, 2H), 6.99–6.90 (m, 2H), 6.57 (t, *J* = 4.5 Hz, 1H), 4.36 (q, *J* = 7.1 Hz, 2H), 2.66–2.59 (m, 2H), 2.56 (td, *J* = 6.0, 4.5 Hz, 2H), 2.19–2.08 (m, 2H), 1.39 (t, *J* = 7.1 Hz, 3H).

^{13}C NMR (101 MHz, $CDCl_3$) δ = 193.2, 166.1, 160.8, 148.5, 133.7, 131.6, 124.9, 116.3, 60.8, 38.8, 24.9, 22.8, 14.4.

FTIR (neat) ν/cm^{-1} 2939.1, 1688.3, 1600.4, 1504.2, 1273.5, 1249.2, 1226.2, 1160.7, 1097.5, 1015.1, 909.2, 850.7, 768.8, 752.0, 693.5.

HRMS (ESI) *m/z* calcd. for $C_{15}H_{16}O_4Na$ $[M+Na]^+$: 283.0941; found: 283.0938.

2-(2-Bromophenoxy)cyclohex-2-en-1-one (S32)

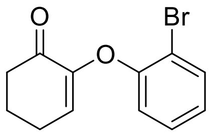

Prepared according to **General Procedure B** with 2,3-epoxycyclohexanone (448 mg, 4.00 mmol), 2-bromophenol (1.04 g, 6.00 mmol), K_2CO_3 (663 mg, 4.20 mmol) and acetonitrile (16 mL). Reaction time: 18 h. Purification *via* flash column chromatography (PE:Et_2O, 2:1) afforded the title compound **S32** as a colourless solid (601.2 mg, 2.25 mmol, 56%).

m.p. = 55–57 °C ($CHCl_3$).

^{1}H NMR (400 MHz, $CDCl_3$) δ = 7.51 (dd, *J* = 8.0, 1.6 Hz, 1H), 7.19–7.14 (m, 1H), 6.91 (ddd, *J* = 7.9, 7.4, 1.5 Hz, 1H), 6.84 (dd, *J* = 8.1, 1.5 Hz, 1H), 6.06 (t, *J* = 4.5 Hz, 1H), 2.56–2.51 (m, 2H), 2.39 (td, *J* = 6.0, 4.5 Hz, 2H), 2.06–1.96 (m, 2H).

^{13}C NMR (101 MHz, $CDCl_3$) δ = 193.1, 152.9, 149.4, 133.9, 128.7, 128.6, 125.2, 119.8, 114.4, 38.9, 24.8, 22.9.

FTIR (neat) ν/cm^{-1} 3015.5, 2950.9, 1687.4, 1631.7, 1582.6, 1471.9, 1442.6, 1351.9, 1249.9, 1228.2, 1140.9, 1104.7, 1029.4, 911.9, 745.8, 665.0.

HRMS (ESI)) *m/z* calcd. for $C_{12}H_{12}O_2Br$ [M+H]$^+$: 267.0015; found: 267.0014.

2-(3,5-Dichlorophenoxy)cyclohex-2-en-1-one (S33)

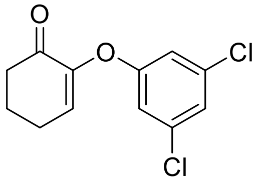

Prepared according to **General Procedure B** with 2,3-epoxycyclohexanone (280 mg, 2.50 mmol), 3,5-dichlorophenol (611 mg, 3.75 mmol), K_2CO_3 (415 mg, 3.00 mmol) and acetonitrile (10 mL). Reaction time: 14 h. Purification *via* flash chromatography (PE:Et_2O, 2:1) afforded the title compound **S33** as a colourless solid (190 mg, 0.74 mmol, 30%).

m.p. 116-117 °C ($CHCl_3$).

^{1}H NMR (400 MHz, $CDCl_3$) δ = 7.03 (t, *J* = 1.7 Hz, 1H), 6.80 (d, *J* = 1.7 Hz, 2H), 6.56 (t, *J* = 4.5 Hz, 1H), 2.59 (dd, *J* = 6.7, 6.7 Hz, 2H), 2.57-2.49 (m, 2H), 2.15-2.05 (m, 2H).

^{13}C NMR (101 MHz, $CDCl_3$) δ = 193.2, 158.2, 148.3, 135.6, 134.5, 123.2, 115.8, 38.8, 25.0, 22.8.

FTIR (neat) ν/cm^{-1} = 2981, 1686, 1587, 1462, 1437, 1383, 1251, 1148, 1113, 1073, 943, 833.

HRMS (EI) *m/z* calcd. for $C_{12}H_{10}O_2Cl_2$ [M]$^+$: 256.0058; found: 256.0058.

2-(3,5-Dimethoxyphenoxy)cyclohex-2-en-1-one (S34)

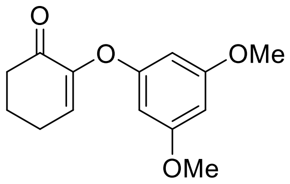

Prepared according to **General Procedure B** with 2,3-epoxycyclohexanone (280 mg, 2.50 mmol), 3,5-dimethoxyphenol (578 mg, 3.75 mmol), K_2CO_3 (415 mg, 3.00 mmol) and acetonitrile (10 mL). Reaction time: 16 h. Purification *via* flash chromatography (PE:Et_2O, 2:1) afforded the title compound **S34** as a colourless solid (329 mg, 1.33 mmol, 53%).

m.p. 62-64 °C ($CHCl_3$).

^{1}H NMR (400 MHz, $CDCl_3$) δ = 6.39 (t, *J* = 4.5 Hz, 1H), 6.18 (dd, *J* = 2.2, 2.2 Hz, 1H), 6.12 (d, *J* = 2.2 Hz, 2H), 3.76 (s, 6H), 2.58 (dd, *J* = 6.7, 6.7 Hz, 2H), 2.52-2.45 (m, 2H), 2.12-2.03 (m, 2H).

^{13}C NMR (101 MHz, $CDCl_3$) δ = 193.8, 161.6, 158.5, 149.4, 161.3, 96.5, 95.3, 55.5, 38.9, 24.9, 23.0.

FTIR (neat) ν/cm^{-1} = 2981, 1690, 1597, 1474, 1382, 1248, 1204, 1150, 1066, 955, 822.

HRMS (ESI) *m/z* calcd. for $C_{14}H_{16}O_4Na$ $[M+Na]^+$: 271.0941; found: 271.0938.

3-Methyl-2-phenoxycyclopent-2-en-1-one (1b)

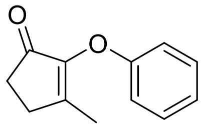

Prepared according to **General Procedure B** with 2,3-epoxy-3-methylcyclopentanone (561 mg, 5.00 mmol), phenol (706 mg, 7.50 mmol), K_2CO_3 (831 mg, 6.00 mmol) and acetonitrile (20 mL). Reaction time: 18 h. Purification *via* flash column chromatography (PE:Et_2O, 2:1→3:2) afforded the title compound **1b** as a colourless solid (815 mg, 4.33 mmol, 87%).

m.p. = 64–66 °C ($CHCl_3$).

^{1}H NMR (400 MHz, $CDCl_3$) δ = 7.31–7.21 (m, 2H), 7.05–6.96 (m, 1H), 6.92–6.84 (m, 2H), 2.65–2.56 (m, 2H), 2.55–2.46 (m, 2H), 2.01 (s, 3H).

^{13}C NMR (101 MHz, $CDCl_3$) δ = 201.7, 160.2, 156.7, 149.3, 129.6, 122.5, 115.6, 32.9, 27.8, 15.4.

FTIR (neat) ν/cm^{-1} = 2912, 1707, 1654, 1590, 1490, 1379, 1330, 1220, 1167, 1088, 894, 831, 816, 757, 721, 688, 638.

HRMS (ESI) *m/z* calcd. for $C_{12}H_{13}O_2$ $[M+H]^+$: 189.0910; found: 189.0911.

2-(2-Chlorophenoxy)-3-methylcyclopent-2-en-1-one (S36)

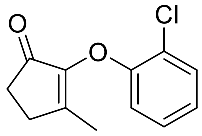

Prepared according to **General Procedure B** with 2,3-epoxy-3-methylcyclopentanone (280 mg, 2.50 mmol), 2-chlorophenol (482 mg, 3.75 mmol), K_2CO_3 (415 mg, 3.00 mmol) and acetonitrile (10 ml). Reaction

time: 18 h. Purification *via* flash column chromatography (PE:Et_2O, 2:1→1:1) afforded the title compound **S36** as a colourless solid (453 mg, 2.03 mmol, 81%).

m.p. = 98–100 °C ($CHCl_3$).

^1H NMR (400 MHz, $CDCl_3$) δ = 7.37 (dd, *J* = 7.9, 1.7 Hz, 1H), 7.11 (td, *J* = 7.9, 1.6 Hz, 1H), 6.96 (td, *J* = 7.7, 1.5 Hz, 1H), 6.72 (dd, *J* = 8.2, 1.5 Hz, 1H), 2.65–2.55 (m, 2H), 2.55–2.45 (m, 2H), 2.04 (s, 3H).

^{13}C NMR (101 MHz, $CDCl_3$) δ = 200.9, 160.0, 152.2, 149.1, 130.7, 127.6, 123.4, 123.0, 115.9, 32.8, 27.7, 15.3.

FTIR (neat) ν/cm^{-1} = 2928, 1699, 1651, 1583, 1476, 1444, 1381, 1334, 1270, 1236, 1191, 1089, 1058, 1036, 835, 758, 697, 668, 626.

HRMS (ESI) *m/z* calcd. for $C_{12}H_{12}O_2Cl$ $[M+H]^+$: 223.0520; found: 223.0522.

2-(2-Bromophenoxy)-3-methylcyclopent-2-en-1-one (S37)

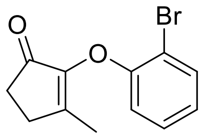

Prepared according to **General Procedure B** with 2,3-epoxy-3-methylcyclopentanone (561 mg, 5.00 mmol), 2-bromophenol (1.30 g, 7.50 mmol), K_2CO_3 (831 mg, 6.00 mmol) and acetonitrile (20 mL). Reaction time: 18 h. Purification *via* flash column chromatography (PE:Et_2O, 2:1→1:1) afforded the title compound **S37** as a colourless solid (1.11 g, 4.17 mmol, 83%).

m.p. = 105–107 °C ($CHCl_3$).

^1H NMR (400 MHz, $CDCl_3$) δ = 7.55 (dd, *J* = 7.9, 1.6 Hz, 1H), 7.16 (ddd, *J* = 8.2, 7.4, 1.6 Hz, 1H), 6.90 (ddd, *J* = 8.0, 7.8, 1.3 Hz, 1H), 6.69 (dd, *J* = 8.2, 1.5 Hz, 1H), 2.66–2.56 (m, 2H), 2.55–2.46 (m, 2H), 2.04 (s, 3H).

^{13}C NMR (101 MHz, $CDCl_3$) δ = 200.8, 160.1, 153.2, 149.3, 133.7, 128.3, 123.9, 115.7, 112.0, 32.9, 27.8, 15.4.

FTIR (neat) ν/cm^{-1} = 2913, 1706, 1653, 1577, 1471, 1331, 1268, 1237, 1084, 1029, 747.

HRMS (ESI) *m/z* calcd. for $C_{12}H_{12}O_2Br$ $[M+H]^+$: 267.0015; found: 267.0017.

2-(2-Iodophenoxy)-3-methylcyclopent-2-en-1-one (S38)

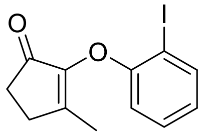

Prepared according to **General Procedure B** with 2,3-epoxy-3-methylcyclopentanone (280 mg, 2.50 mmol), 2-iodophenol (825 mg, 3.75 mmol), K_2CO_3 (415 mg, 3.00 mmol) and acetonitrile (10 ml). Reaction time: 18 h. Purification *via* flash column chromatography (PE:Et_2O, 2:1→1:1) afforded the title compound **S38** as a colourless solid (611 mg, 1.95 mmol, 78%).

m.p. = 96–98 °C ($CDCl_3$).

^{1}H NMR (400 MHz, $CDCl_3$) δ = 7.78 (dd, *J* = 7.8, 1.6 Hz, 1H), 7.20 (td, *J* = 7.5, 1.6 Hz, 1H), 6.77 (td, *J* = 7.6, 1.4 Hz, 1H), 6.61 (dd, *J* = 8.2, 1.4 Hz, 1H), 2.66–2.57 (m, 2H), 2.55–2.46 (m, 2H), 2.04 (s, 3H).

^{13}C NMR (101 MHz, $CDCl_3$) δ = 200.9, 160.4, 155.6, 149.5, 139.8, 129.4, 124.3, 114.5, 85.8, 32.9, 27.8, 15.5.

FTIR (neat) ν/cm^{-1} = 3057, 2921, 1704, 1653, 1578, 1467, 1437, 1381, 1331, 1268, 1230, 1192, 1120, 1088, 1019, 830, 756, 655, 627.

HRMS (ESI) *m/z* calcd. for $C_{12}H_{11}O_2INa$ [M+Na]$^+$: 336.9696; found: 336.9695.

2-(3-(Dimethylamino)phenoxy)-3-methylcyclopent-2-en-1-one (S39)

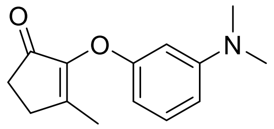

Prepared according to **General Procedure B** with 2,3-epoxy-3-methylcyclopentanone (280 mg, 2.50 mmol), 3-(dimethylamino)phenol (514 mg, 3.75 mmol), K_2CO_3 (415 mg, 3.00 mmol) and acetonitrile (10 ml). Reaction time: 18 h. Purification *via* flash column chromatography (PE:Et_2O, 1:1) afforded the title compound **S39** as a red-brown solid (417 mg, 1.80 mmol, 72%).

m.p. = 57–59 °C ($CHCl_3$).

^{1}H NMR (400 MHz, $CDCl_3$) δ = 7.07 (t, *J* = 8.2 Hz, 1H), 6.39 (ddd, *J* = 8.3, 2.5, 0.8 Hz, 1H), 6.36 (t, *J* = 2.4 Hz, 1H), 6.12 (ddd, *J* = 8.1, 2.3, 0.8 Hz, 1H), 2.92 (s, 6H), 2.64–2.55 (m, 2H), 2.52–2.45 (m, 2H), 2.01 (s, 3H).

^{13}C NMR (101 MHz, $CDCl_3$) δ = 201.9, 159.8, 157.8, 152.1, 149.4, 129.7, 107.2, 102.8, 100.6, 40.6, 32.9, 27.8, 15.4.

FTIR (neat) ν/cm^{-1} = 2914, 2805, 1710, 1653, 1607, 1572, 1500, 1438, 1383, 1333, 1219, 1142, 1088, 997, 880, 823, 752, 684.

HRMS (EI) *m/z* calcd. for $C_{14}H_{17}NO_2$ [M]$^+$: 231.1254; found: 231.1257.

2-(4-Methoxyphenoxy)-3-methylcyclopent-2-en-1-one (S40)

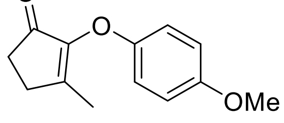

Prepared according to **General Procedure B** with 2,3-epoxy-3-methylcyclopentanone (280 mg, 2.50 mmol), 4-methoxyphenol (466 mg, 3.75 mmol), K_2CO_3 (415 mg, 3.00 mmol) and acetonitrile (10 ml). Reaction time: 18 h. Purification *via* flash column chromatography (PE:Et_2O, 2:1) afforded the title compound **S40** as a colourless solid (488 mg, 2.24 mmol, 89%).

m.p. = 49–51 °C ($CHCl_3$).

^{1}H NMR (400 MHz, $CDCl_3$) δ = 6.88–6.81 (m, 2H), 6.81–6.74 (m, 2H), 3.75 (s, 3H), 2.62–2.53 (m, 2H), 2.52–2.43 (m, 2H), 2.00 (s, 3H).

^{13}C NMR (101 MHz, $CDCl_3$) δ = 202.0, 159.3, 155.1, 150.7, 149.9, 116.8, 114.7, 55.8, 32.9, 27.7, 15.4.

FTIR (neat) ν/cm^{-1} = 1703, 1651, 1503, 1458, 1444, 1382, 1333, 1247, 1207, 1183, 1089, 1030, 826, 807, 758, 732, 667.

HRMS (CI) *m/z* calcd. for $C_{13}H_{15}O_3$ $[M+H]^+$: 219.1016; found: 219.1012.

3-Methyl-2-(*p*-tolyloxy)cyclopent-2-en-1-one (S41)

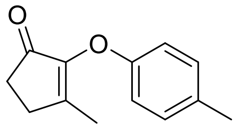

Prepared according to **General Procedure B** with 2,3-epoxy-3-methylcyclopentanone (280 mg, 2.50 mmol), *p*-cresol (406 mg, 3.75 mmol), K_2CO_3 (415 mg, 3.00 mmol) and acetonitrile (10 ml). Reaction time: 18 h. Purification *via* flash column chromatography (PE:Et_2O, 2:1→3:2) afforded the title compound **S41** as a colourless solid (436 mg, 2.15 mmol, 86%).

m.p. = 65–67 °C ($CHCl_3$).

^{1}H NMR (400 MHz, $CDCl_3$) δ = 7.05 (d, *J* = 8.2 Hz, 2H), 6.79 (d, *J* = 8.8 Hz, 2H), 2.64–2.55 (m, 2H), 2.53–2.45 (m, 2H), 2.28 (s, 3H), 2.01 (s, 3H).

^{13}C NMR (101 MHz, $CDCl_3$) δ = 201.9, 159.7, 154.6, 149.5, 131.9, 130.0, 115.5, 32.9, 27.8, 20.7, 15.4.

FTIR (neat) ν/cm^{-1} = 3031, 2924, 1699, 1656, 1607, 1504, 1435, 1385, 1333, 1219, 1167, 1091, 845, 820, 624.

HRMS (ESI) *m/z* calcd. for $C_{13}H_{15}O_2$ $[M+H]^+$: 203.1067; found: 203.1066.

4-((2-Methyl-5-oxocyclopent-1-en-1-yl)oxy)benzaldehyde (S42)

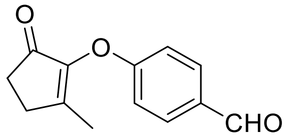

Prepared according to **General Procedure B** with 2,3-epoxy-3-methylcyclopentanone (280 mg, 2.50 mmol), 4-hydroxybenzaldehyde (438 mg, 3.75 mmol), K_2CO_3 (415 mg, 3.00 mmol) and acetonitrile (10 ml). Reaction time: 18 h. Purification *via* flash column chromatography (PE:Et_2O, 1:1→0:1) afforded the title compound **S42** as a colourless solid (264 mg, 1.21 mmol, 49%).

m.p. = 62–64 °C ($CHCl_3$).

^{1}H NMR (400 MHz, $CDCl_3$) δ = 9.89 (s, 1H), 7.82 (d, *J* = 8.9 Hz, 2H), 6.98 (d, *J* = 8.5 Hz, 2H), 2.70–2.62 (m, 2H), 2.56–2.49 (m, 2H), 2.05 (s, 3H).

^{13}C NMR (101 MHz, $CDCl_3$) δ = 200.8, 190.8, 161.4, 161.3, 148.5, 132.0, 131.4, 116.0, 32.9, 27.9, 15.4.

FTIR (neat) ν/cm^{-1} = 2909, 2848, 2757, 1711, 1684, 1656, 1595, 1579, 1504, 1431, 1406, 1378, 1329, 1300, 1235, 1164, 1082, 860, 834, 813, 678, 648.

HRMS (ESI) *m/z* calcd. for $C_{13}H_{12}O_3Na$ $[M+Na]^+$: 239.0679; found: 239.0679.

Ethyl 4-((2-methyl-5-oxocyclopent-1-en-1-yl)oxy)benzoate (S43)

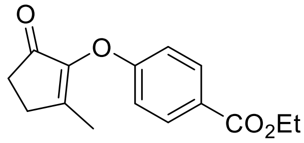

Prepared according to **General Procedure B** with 2,3-epoxy-3-methylcyclopentanone (561 mg, 5.00 mmol), ethyl 4-hydroxybenzoate (997 mg, 7.50 mmol), K_2CO_3 (831 mg, 6.00 mmol) and acetonitrile (20 mL). Reaction time: 18 h. Purification *via* flash column chromatography (PE:Et_2O, 2:1→1:1) afforded the title compound **S43** as a colourless solid (1.11 g, 4.11 mmol, 82%).

m.p. = 71–73 °C ($CHCl_3$).

^{1}H NMR (400 MHz, $CDCl_3$) δ = 8.01–7.93 (m, 2H), 6.92–6.85 (m, 2H), 4.34 (q, *J* = 7.1 Hz, 2H), 2.68–2.61 (m, 2H), 2.55–2.48 (m, 2H), 2.03 (s, 3H), 1.36 (t, *J* = 7.1 Hz, 3H).

^{13}C NMR (101 MHz, $CDCl_3$) δ = 201.1, 166.2, 160.9, 160.2, 148.8, 131.7, 124.8, 115.2, 60.9, 32.9, 27.8, 15.4, 14.5.

FTIR (neat) ν/cm^{-1} = 1703, 1657, 1600, 1505, 1332, 1275, 1230, 1165, 1101, 1086, 1019, 860, 772, 685.

HRMS (ESI) *m/z* calcd. for $C_{15}H_{16}O_4Na$ $[M+Na]^+$: 283.0941; found: 283.0942.

3-Methyl-2-(pyridin-3-yloxy)cyclopent-2-en-1-one (S44)

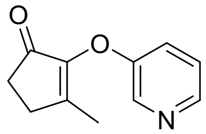

Prepared according to **General Procedure B** with 2,3-epoxy-3-methylcyclopentanone (561 mg, 5.00 mmol), 3-hydroxypyridine (713 mg, 7.50 mmol), K_2CO_3 (831 mg, 6.00 mmol) and acetonitrile (20 mL). Reaction time: 18 h. Purification *via* flash column chromatography (EtOAc) afforded the title compound **S44** as a brown solid (357 mg, 1.89 mmol, 38%).

m.p. = 56–58 °C ($CHCl_3$).

^{1}H NMR (400 MHz, $CDCl_3$) δ = 8.32–8.26 (m, 2H), 7.25–7.13 (m, 2H), 2.68–2.61 (m, 2H), 2.55–2.48 (m, 2H), 2.06 (s, 3H).

^{13}C NMR (101 MHz, $CDCl_3$) δ = 201.0, 160.7, 153.1, 148.8, 144.0, 138.7, 124.0, 122.8, 32.9, 27.8, 15.4.

FTIR (neat) ν/cm^{-1} = 1698, 1654, 1572, 1478, 1425, 1335, 1252, 1228, 1187, 1021, 807, 707, 613.

HRMS (ESI) *m/z* calcd. for $C_{11}H_{12}O_2N$ $[M+H]^+$: 190.0863; found: 190.0863.

3-Ethyl-2-(*p*-tolyloxy)cyclopent-2-en-1-one (S45)

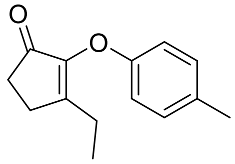

Prepared according to **General Procedure B** with 2,3-epoxy-3-ethylcyclopentanone (**S2e**) (126 mg, 1.00 mmol) and *p*-cresol (162 mg,

1.50 mmol), K_2CO_3 (166 mg, 1.20 mmol) and acetonitrile (4 mL). Purification *via* flash column chromatography (PE:Et_2O, 2:1→1:1) afforded the title compound **S45** as a yellow oil (110 mg, 0.51 mmol, 51%).

^{1}H NMR (400 MHz, $CDCl_3$) δ = 7.05 (d, *J* = 8.3 Hz, 2H), 6.77 (d, *J* = 8.4 Hz, 2H), 2.65–2.58 (m, 2H), 2.52–2.47 (m, 2H), 2.43 (q, *J* = 7.7 Hz, 2H), 2.28 (s, 3H), 1.12 (t, *J* = 7.6 Hz, 3H).

^{13}C NMR (101 MHz, $CDCl_3$) δ = 202.2, 165.0, 154.7, 148.6, 131.8, 130.1, 115.3, 32.9, 25.1, 22.5, 20.7, 11.5.

FTIR (neat) ν/cm^{-1} = 2972, 2924, 1710, 1648, 1609, 1505, 1461, 1351, 1298, 1219, 1167, 1090, 1063, 816, 762, 619.

HRMS (ESI) *m/z* calcd. for $C_{14}H_{17}O_2$ [M+H]$^+$: 217.1223; found: 217.1224.

3-Benzyl-2-(*p*-tolyloxy)cyclopent-2-en-1-one (S46)

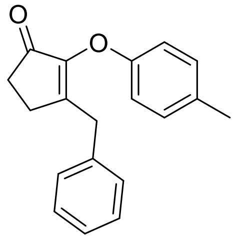

Prepared according to **General Procedure B** with 2,3-epoxy-3-benzylcyclopentanone (**S2f**) (369 mg, 1.96 mmol), *p*-cresol (324 mg, 3.00 mmol), K_2CO_3 (325 mg, 2.35 mmol) and acetonitrile (8 mL). Purification *via* flash column chromatography (PE:Et_2O, 3:1) afforded the title compound **S46** as a yellow oil (398 mg, 1.43 mmol, 73%).

^{1}H NMR (400 MHz, $CDCl_3$) δ = 7.29–7.17 (m, 3H), 7.16–7.10 (m, 2H), 7.04 (d, *J* = 8.6 Hz, 2H), 6.79 (d, *J* = 8.6 Hz, 2H), 3.68 (s, 2H), 2.48–2.41 (m, 2H), 2.41–2.35 (m, 2H), 2.26 (s, 3H).

^{13}C NMR (101 MHz, $CDCl_3$) δ = 201.9, 161.2, 154.6, 148.8, 136.8, 132.0, 130.1, 129.1, 128.9, 127.0, 115.5, 35.5, 32.9, 25.1, 20.7.

FTIR (neat) ν/cm^{-1} = 3028, 2922, 1710, 1650, 1608, 1504, 1454, 1406, 1343, 1228, 1208, 1168, 1079, 817, 756, 701.

HRMS (ESI) *m/z* calcd. for $C_{19}H_{19}O_2$ [M+H]$^+$: 279.1380; found: 279.1380.

3-Cyclohexyl-2-(*p*-tolyloxy)cyclopent-2-en-1-one (S47)

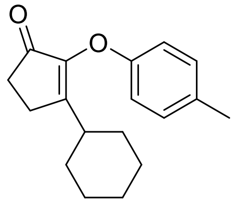

Prepared according to **General Procedure B** with 2,3-epoxy-3-cyclohexylcyclopentanone (**S2g**) (196 mg, 1.09 mmol), *p*-cresol (176 mg, 1.63 mmol), K_2CO_3 (181 mg, 1.31 mmol) and acetonitrile (4.4 mL). Purification *via* flash column chromatography (PE:Et_2O, 3:1→2:1) afforded the title compound **S47** as a yellow oil that solidified on standing (262 mg, 0.970 mmol, 89%).

m.p. = 42–44 °C ($CHCl_3$).

^{1}H NMR (400 MHz, $CDCl_3$) δ = 7.05 (d, *J* = 8.5 Hz, 2H), 6.78 (d, *J* = 8.5 Hz, 2H), 2.71 (tt, *J* = 11.9, 3.4 Hz, 1H), 2.63–2.55 (m, 2H), 2.51–2.42 (m, 2H), 2.28 (s, 3H), 1.82–1.65 (m, 5H), 1.46–1.11 (m, 5H).

^{13}C NMR (101 MHz, $CDCl_3$) δ = 202.3, 168.2, 154.7, 147.8, 131.7, 130.1, 115.3, 38.2, 32.9, 30.3, 26.0, 25.9, 23.0, 20.7.

FTIR (neat) ν/cm^{-1} = 2925, 2853, 1711, 1643, 1609, 1505, 1448, 1337, 1167, 1105, 1072, 846, 816.

HRMS (ESI) *m/z* calcd. for $C_{18}H_{23}O_2$ [M+H]$^+$: 271.1693; found: 271.1692.

2-(*p*-Tolyloxy)cyclopent-2-en-1-one (S48)

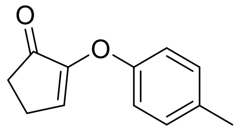

Prepared according to **General Procedure B** with 2,3-epoxycyclopentanone (491 mg, 5.00 mmol), *p*-cresol (811 mg, 7.5 mmol), K_2CO_3 (831 mg, 7.50 mmol) and acetonitrile (20 mL). Purification *via* flash column chromatography (PE:Et_2O, 2:1→1:1) afforded the title compound **S48** as a colourless solid (524 mg, 2.78 mmol, 56%).

m.p. = 67–69 °C ($CHCl_3$).

^{1}H NMR (400 MHz, $CDCl_3$) δ = 7.13 (d, *J* = 8.3 Hz, 2H), 6.98 (d, *J* = 8.4 Hz, 2H), 6.41 (t, *J* = 1.9 Hz, 1H), 2.53–2.45 (m, 4H), 2.30 (s, 3H).

^{13}C NMR (101 MHz, $CDCl_3$) δ = 202.0, 156.7, 153.4, 134.4, 133.6, 130.3, 119.6, 33.3, 21.9, 20.8.

FTIR (neat) ν/cm^{-1} = 2924, 1712, 1625, 1607, 1505, 1447, 1404, 1339, 1300, 1263, 1219, 1166, 1080, 1018, 929, 843, 785.

HRMS (ESI) *m/z* calcd. for $C_{12}H_{13}O_2$ [M+H]$^+$: 189.0910; found: 189.0911.

3-Methyl-2-(*p*-tolylthio)cyclohex-2-en-1-one (S49)

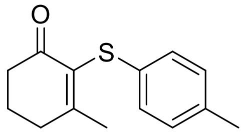

Prepared according to **General Procedure B** with 2,3-epoxy-3-methylcyclohexanone (631 mg, 5.00 mmol), *p*-thiocresol (807 mg, 6.50 mmol), K_2CO_3 (829 mg, 6.00 mmol) and acetonitrile (20 mL). Reaction time: 1 h. Purification *via* flash chromatography (PE:Et_2O, 3:1) afforded the title compound **S49** as a colourless solid (729 mg, 3.14 mmol, 63%).

m.p. 78-80 °C ($CHCl_3$).

^{1}H NMR (400 MHz, $CDCl_3$) δ = 7.06 (d, *J* = 8.2 Hz, 2H), 7.02 (d, *J* = 8.2 Hz, 2H), 2.59 (dd, *J* = 6.0, 6.0 Hz, 2H), 2.53 (dd, *J* = 6.7, 6.7 Hz, 2H), 2.27 (s, 6H), 2.07-1.98 (m, 2H).

^{13}C NMR (101 MHz, $CDCl_3$) δ = 21.1, 21.9, 24.6, 34.2, 38.5, 128.0, 129.7, 130.4, 133.1, 135.5, 168.8, 194.8.

FTIR (neat) ν/cm^{-1} = 2955, 1662, 1576, 1491, 1419, 1406, 1373, 1347, 1326, 1304, 1275, 1211, 1194, 1176, 1137, 1118, 1087, 1060, 1047, 1013, 988, 967, 801.

HRMS (ESI) *m/z* calcd. for $C_{14}H_{16}OSNa$ [M+Na]$^+$: 255.0814; found: 255.0814.

3-Methyl-2-(*p*-tolylthio)cyclopent-2-en-1-one (S50)

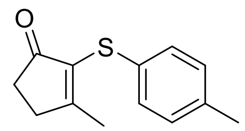

Prepared according to **General Procedure B** with 2,3-epoxy-3-methylcyclopentanone (280 mg, 2.50 mmol), *p*-thiocresol (466 mg, 3.75 mmol), K_2CO_3 (415 mg, 3.00 mmol) and acetonitrile (12.5 mL). Reaction time: 18 h. Purification *via* flash column chromatography (PE:Et_2O, 2:1) afforded the title compound **S50** as a brown solid (340 mg, 1.56 mmol, 62%).

m.p. 53–55 °C ($CHCl_3$).

^1H NMR (400 MHz, $CDCl_3$) δ = 7.13 (d, *J* = 8.2 Hz, 2H), 7.03 (d, *J* = 8.0 Hz, 2H), 2.75–2.67 (m, 2H), 2.55–2.48 (m, 2H), 2.28 (s, 3H), 2.25 (s, 3H).

^{13}C NMR (101 MHz, $CDCl_3$) δ = 205.3, 181.1, 136.4, 133.8, 130.9, 129.8, 129.3, 34.3, 32.5, 21.1, 19.1.

FTIR (film) ν/cm^{-1} = 1695, 1601, 1490, 1424, 1397, 1374, 1266, 1156, 1116, 1087, 1038, 1015, 960, 941, 803, 666.

HRMS (EI) *m/z* calcd. for $C_{13}H_{14}OS$ $[M]^+$: 218.0765; found: 218.0754.

Synthesis of Progesteron Substrate S27

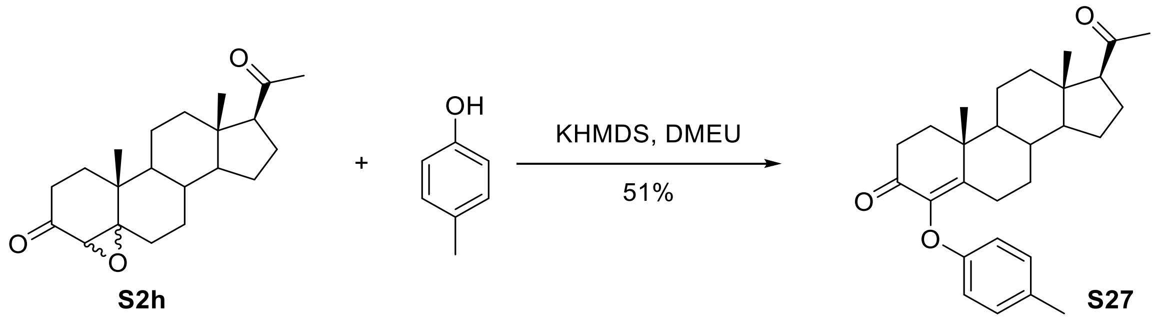

p-Cresol (263 mg, 2.43 mmol) was dissolved in THF (2.0 mL) and KHMDS (0.5 M in toluene, 0.66 mL, 0.33 mmol) was added dropwise at room temperature. The solution was stirred at room temperature for 10 min, then 1,3-dimethyl-2-imidazolidinone (0.47 mL, 4.42 mmol) and a solution of progesterone epoxide **S2h**[21] (729 mg, 2.21 mmol, ratio α:β = ca. 1:4) in THF (2.4 mL) were added. The reaction mixture was refluxed for 74 h after which it was poured into NaOH (1 M, 20 mL) and extracted with Et_2O (3 × 30 mL). The combined organic extracts were washed with brine (30 mL) and dried over Na_2SO_4. Purification of the residue *via* flash chromatography (PE:Et_2O, 1:1) afforded the corresponding enone **S27** (473 mg, 1.12 mmol, 51%) as a yellow solid.

m.p. 74-75°C ($CHCl_3$).

^1H NMR (400 MHz, $CDCl_3$) δ = 7.03 (d, *J* = 8.2 Hz, 2H), 6.70 (d, *J* = 8.5 Hz, 2H), 2.96-2.81 (m, 1H), 2.64-2.42 (m, 3H), 2.26 (s, 3H), 2.24-1.39 (m, 14H), 1.33-0.90 (m, 8H), 0.67 (s, 3H).

^{13}C NMR (101 MHz, $CDCl_3$) δ = 209.5, 192.6, 157.0, 155.8, 141.6, 131.0, 130.1, 114.5, 63.6, 56.1, 54.2, 44.0, 39.3, 38.8, 35.2, 35.0, 34.2, 31.7, 31.2, 24.4, 24.0, 23.0, 21.1, 20.7, 17.8, 13.5.

FTIR (neat) ν/cm^{-1} = 2940, 1686, 1605, 1505, 1448, 1358, 1316, 1288, 1220, 1189, 1167, 1149, 1113, 1062, 1017, 935, 908, 813, 751.

HRMS (ESI) *m/z* calcd. for $C_{28}H_{37}O_3$ $[M+H]^+$: 421.2737; found: 421.1738.

Synthesis of 2-arylaminoketones

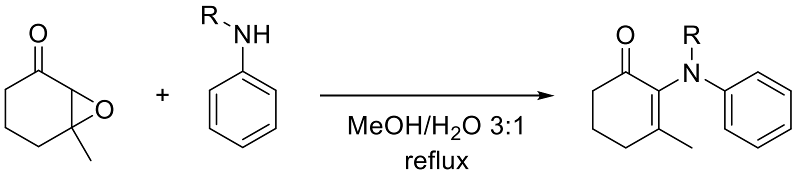

S51-S55

General Procedure C:

The appropriate aniline (1.2 eq.) and epoxide (1.0 eq.) were added to a round-bottomed flask fitted with a condenser. Methanol (1.8 mL/mmol epoxide) and water (0.6 mL/mmol epoxide) were added and the resulting solution was degassed by sparging with argon for 30 min. The mixture was heated to reflux for the appropriate time under an atmosphere of argon before being cooled to room temperature. The reaction mixture was poured into water (10 mL/mmol) and extracted with Et_2O (3 × 10 mL/mmol). The combined organic extracts were washed with brine (5 mL/mmol), dried over Na_2SO_4, filtered and concentrated under reduced pressure. Purification of the residue *via* flash chromatography (see experimental methods section for specific details) afforded the corresponding 2-arylaminoketone.

3-Methyl-2-(methyl(phenyl)amino)cyclohex-2-en-1-one (S51)

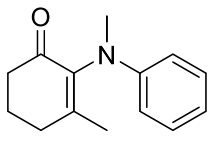

Prepared according to **General Procedure C** with 2,3-epoxy-3-methylcyclohexanone (631 mg, 5.00 mmol), *N*-methylaniline (0.65 mL, 6.00 mmol), methanol (9 mL) and water (3 mL). Reaction time: 68 h. Purification *via* flash chromatography (PE:Et_2O, 2:1) afforded the title compound **S51** as a yellow oil (757 mg, 3.52 mmol, 70%).

^1H NMR (400 MHz, $CDCl_3$) δ = 7.20-7.13 (m, 2H), 6.71-6.64 (m, 1H), 6.57-6.51 (m, 2H), 3.02 (s, 3H), 2.58-2.47 (m, 4H), 2.12-2.01 (m, 2H), 1.88 (s, 3H).

^{13}C NMR (101 MHz, $CDCl_3$) δ = 196.2, 158.8, 148.4, 139.8, 129.2, 116.8, 111.7, 38.9, 37.9, 32.3, 22.2, 20.2.

FTIR (neat) ν/cm^{-1} = 2939, 1677, 1598, 1501, 1377, 1341, 1290, 1227, 1180, 1119, 926, 748.

HRMS (ESI) *m/z* calcd. for $C_{14}H_{18}ON$ $[M+H]^+$: 216.1383; found: 216.1385.

3-Methyl-2-(methyl(phenyl)amino)cyclopent-2-en-1-one (S52)

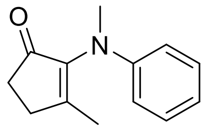

Prepared according to **General Procedure C** with 2,3-epoxy-3-methylcyclopentanone (280 mg, 2.50 mmol), *N*-methylaniline (0.33 mL, 3.00 mmol), methanol (4.5 mL) and water (1.5 mL). Reaction time: 15.5 h. Purification *via* flash chromatography (PE:Et_2O, 3:1→2:1) afforded the title compound **S52** as a yellow oil (470 mg, 2.36 mmol, 93%).

^{1}H NMR (400 MHz, $CDCl_3$) δ = 7.29-7.14 (m, 2H), 6.78-6.69 (m, 1H), 6.63-6.55 (m, 2H), 3.13 (s, 3H), 2.67-2.59 (m, 2H), 2.53-2.46 (m, 2H), 1.92 (s, 3H).

^{13}C NMR (101 MHz, $CDCl_3$) δ = 206.3, 168.2, 147.4, 144.2, 129.1, 117.8, 113.1, 37.4, 33.8, 29.5, 17.6.

FTIR (neat) ν/cm^{-1} = 2909, 1703, 1639, 1597, 1498, 1387, 1357, 1286, 1221, 1186, 1156, 1110, 1033, 949, 811, 747, 692.

HRMS (ESI) *m/z* calcd. for $C_{13}H_{16}ON$ $[M+H]^+$: 202.1226; found: 202.1227.

2-(Allyl(phenyl)amino)-3-methylcyclohex-2-en-1-one (S53)

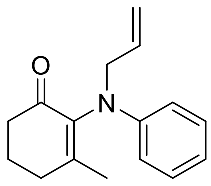

Prepared according to **General Procedure C** with 2,3-epoxy-3-methylcyclohexanone (315 mg, 2.50 mmol), *N*-allylaniline (0.41 mL, 3.00 mmol), methanol (4.5 mL) and water (1.5 mL). Reaction time: 68 h. Purification *via* flash chromatography (PE:Et_2O, 3:1) afforded the title compound **S53** as a yellow oil (97 mg, 0.40 mmol, 16%).

^{1}H NMR (400 MHz, $CDCl_3$) δ = 7.19-7.11 (m, 2H), 6.72-6.66 (m, 1H), 6.60-5.54 (m, 2H), 5.91 (ddt, *J* = 16.4, 11.1, 6.0 Hz, 1H), 5.24 (dd, *J* = 17.2, 1.6 Hz, 1H), 5.13 (dd, *J* = 10.2, 1.4 Hz, 1H), 3.99 (bs, 2H), 2.59-2.47 (m, 4H), 2.10-2.00 (m, 2H), 1.91 (s, 3H).

^{13}C NMR (101 MHz, $CDCl_3$) δ = 196.4, 159.7, 147.9, 138.6, 135.3, 129.2, 117.2, 116.9, 112.5, 53.9, 38.9, 32.5, 22.2, 20.8.

FTIR (neat) ν/cm^{-1} = 2925, 1674, 1625, 1597, 1499, 1456, 1427, 1379, 1321, 1285, 1246, 1200, 11173, 1134, 1059, 1034, 748.

HRMS (ESI) *m/z* calcd. for $C_{16}H_{20}ON$ $[M+H]^+$: 242.1539; found: 242.1539.

2-(Benzyl(phenyl)amino)-3-methylcyclopent-2-en-1-one (S54)

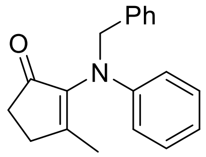

Prepared according to **General Procedure C** with 2,3-epoxy-3-methylcyclopentanone (280 mg, 2.50 mmol), *N*-benzylaniline (550 mg, 3.00 mmol), methanol (4.5 mL) and water (1.5 mL). Reaction time: 21 h. Purification *via* flash chromatography (PE:Et_2O, 4:1→3:1) afforded the title compound **S54** as a yellow soild (239 mg, 0.860 mmol, 34%).

m.p. 99-101°C ($CHCl_3$)

^{1}H NMR (400 MHz, $CDCl_3$) δ = 7.39-7.30 (m, 3H), 7.30-7.19 (m, 2H), 7.19-7.11 (m, 2H), 6.79-6.68 (m, 1H), 6.65-6.58 (m, 2H), 4.82 (s, 2H), 2.64-2.56 (m, 2H), 2.53-2.38 (m, 2H), 1.94 (s, 3H).

^{13}C NMR (101 MHz, $CDCl_3$) δ = 206.3, 169.0, 147.2, 142.8, 139.3, 129.2, 128.6, 127.0, 126.9, 118.4, 113.9, 53.8, 33.8, 29.5, 18.0.

FTIR (neat) ν/cm^{-1} = 3028, 2893, 2856, 1694, 1646, 1597, 1497, 1450, 1441, 1388, 1270, 1208, 1132, 1056, 990, 975, 810, 748, 739, 715, 691, 668.

HRMS (ESI) *m/z* calcd. for $C_{19}H_{20}ON$ $[M+H]^+$: 278.1539; found: 278.1538.

2-(3,4-Dihydroquinolin-1(2*H*)-yl)-3-methylcyclohex-2-en-1-one (S55)

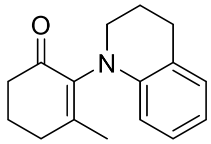

Prepared according to **General Procedure C** with 2,3-epoxy-3-methylcyclohexanone (315 mg, 2.50 mmol), 1,2,3,4-tetrahydroquinoline (0.38 mL, 3.00 mmol), methanol (4.5 mL) and water (1.5 mL). Reaction time: 68 h. Purification *via* flash chromatography (PE:Et_2O, 3:1) afforded the title compound **S55** as a yellow solid (147 mg, 0.61 mmol, 24%).

m.p. 64-66 °C ($CHCl_3$).

^{1}H NMR (400 MHz, $CDCl_3$) δ = 6.96 (d, *J* = 7.3 Hz, 1H), 6.89 (dd, *J* = 7.7, 7.7 Hz, 1H), 6.55 (ddd, *J* = 7.3, 7.3, 1.1 Hz, 1H), 6.13 (dd, *J* = 8.1, 0.7 Hz, 1H), 3.44 (ddd, *J* = 11.4, 8.4, 3.5 Hz, 1H), 3.16-3.08 (m, 1H), 2.91 (ddd, *J* = 15.7, 9.4, 5.4 Hz, 1H), 2.76 (dt, *J* = 15.9, 5.3 Hz, 1H), 2.62-2.44 (m, 4H), 2.15-1.95 (m, 4H), 1.92 (s, 3H).

^{13}C NMR (101 MHz, $CDCl_3$) δ = 195.9, 159.2, 144.4, 138.8, 129.4, 126.9, 121.5, 116.4, 111.4, 49.1, 39.0, 32.3, 28.1, 22.3, 22.2, 20.1.

FTIR (neat) ν/cm^{-1} = 2928, 1674, 1627, 1600, 1576, 1495, 1456, 1375, 1303, 1278, 1219, 1195, 1167, 743.

HRMS (ESI) *m/z* calcd. for $C_{16}H_{20}ON$ $[M+H]^+$: 242.1539; found: 242.1539.

IV. Optimization Data

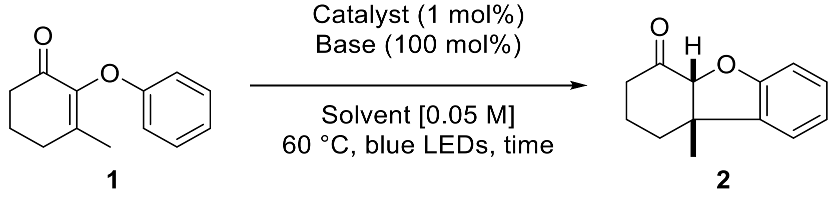

Entry	Catalyst	Base	Solvent	t	Conv.[a]	yield[a]
1	$Ru(bpy)_3Cl_2$	KOAc	MeCN	16 h	0%	0%
2	$Ru(phen)_3Cl_2$	KOAc	MeCN	16 h	0%	0%
3	$[Ir(dtbbpy)(ppy)_2]PF_6$	KOAc	MeCN	16 h	<5%	<5%
4	*fac*-$Ir(ppy)_3$	KOAc	MeCN	16 h	45%	45%
5	$[Ir(dFCF_3ppy)_2(dtbbpy)]PF_6$	KOAc	MeCN	16 h	80%	77%
**6**	**$Ir(Fppy)_3$**	**KOAc**	**MeCN**	**16 h**	**100%**	**95% (94%)**
7	$Ir(Fppy)_3$	KOAc	MeCN	3 h	48%	47%
8	$Ir(Fppy)_3$	NaOAc	MeCN	3 h	84%	80%
9	$Ir(Fppy)_3$	$KHCO_3$	MeCN	3 h	85%	78%
10	$Ir(Fppy)_3$	$NaHCO_3$	MeCN	3 h	84%	80%
11	$Ir(Fppy)_3$	KOAc	EtOAc	3 h	100%	91% (89%)
12	$Ir(Fppy)_3$	NaOAc	EtOAc	3 h	100%	82%
13	$Ir(Fppy)_3$	$KHCO_3$	EtOAc	3 h	100%	82%
14	$Ir(Fppy)_3$	$NaHCO_3$	EtOAc	3 h	100%	80%
15	$Ir(Fppy)_3$	NaOAc	acetone	3 h	100%	80%
16	$Ir(Fppy)_3$	$KHCO_3$	acetone	3 h	100%	86%
17	$Ir(Fppy)_3$	KOAc	Benzene	16 h	100%	82%
18	$Ir(Fppy)_3$	-	Benzene	16 h	100%	51%
19	$Ir(Fppy)_3$	-	MeCN	13 h	100%	57%[b]
20	$Ir(Fppy)_3$	KOAc	MeCN	13 h	100%	95%[c]
21	$Ir(Fppy)_3$	KOAc	MeCN	13 h	100%	87%[d]
22	-	KOAc	MeCN	13 h	0%	0%
23	$Ir(Fppy)_3$	KOAc	MeCN	13 h	0%	0%[e]

The reactions were carried out at a 0.05 mmol scale. [a] Yields and conversion were determined by ^1H NMR analysis of crude reaction mixture using 1,3,5-trimethoxybenzene as an internal standard. Isolated yields are in parentheses. [b] 10:1 d.r., The water content of the acetonitrile was 10 ppm (determined by Karl-Fischer titration). [c] 10 eq of water were added. [d] Reaction was conducted in an air atmosphere with non-degassed solvent. [e] no light.

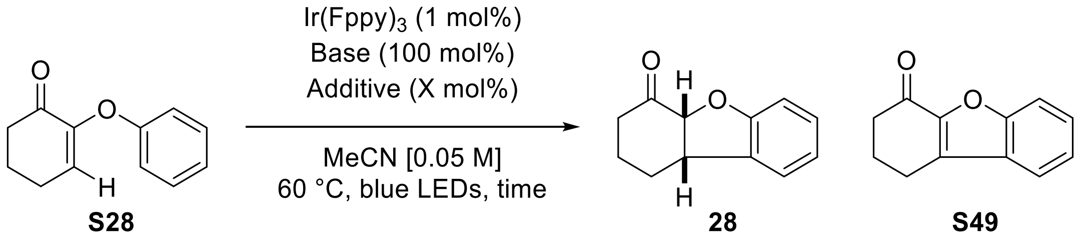

Entry	Base	Additive	t	Conv.[a)]	Yield[a)] (28)	Yield[a)] (S49)
1	KOAc	-	2 h	100%	75% (73%)	4%
2	KOAc	Quinoline (0.2 eq)	2 h	100%	81%	3%
3[b)]	KOAc	Quinoline (0.2 eq)	3 h	95%	67%	7%
4[c)]	KOAc	Quinoline (0.2 eq)	1 h	100%	78%	4%
5	KOAc	6-Methylquinoline (0.2 eq)	2 h	85%	80%	5%
**6**	**KOAc**	**8-Methylquinoline (0.2 eq)**	**2 h**	**100%**	**92% (88%)**	**3%**
7	KOAc	Lutidine (0.2 eq)	2 h	100%	85%	3%
8	KOAc	DIPEA (0.2 eq)	2 h	100%	82%	<1%
9	-	Quinoline (0.2 eq)	2 h	68%	18%	18%
10	-	DIPEA (0.2 eq)	2 h	100%	73%	0%
11	KOAc	8-Methylquinoline (0.5 eq)	2 h	100%	92%	3%

The reactions were carried out at a 0.05 mmol scale. [a)] Yields and conversion were determined by ^{1}H NMR analysis of crude reaction mixture using 1,3,5-trimethoxybenzene as an internal standard. Isolated yields are in parentheses (on an 0.30 mmol scale). [b)] Reaction was conducted at 30 °C. [c)] EtOAc was used as solvent.

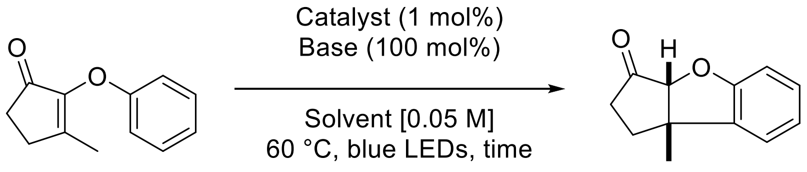

Entry	Catalyst	Base	Solvent	Time	Conv.[a)]	yield[a)]
1	*fac*-Ir(ppy)$_3$	KOAc	MeCN	66 h	0%	0%
2	[Ir(dtbbpy)(ppy)$_2$]PF$_6$	KOAc	MeCN	24 h	0%	0%
3	[Ir(dFCF$_3$ppy)$_2$(bpy)]PF$_6$	KOAc	MeCN	24 h	0%	0%
4	[Ir(dFCF$_3$ppy)$_2$(dtbbpy)]PF$_6$	KOAc	MeCN	24 h	0%	0%
5	Ir(Fppy)$_3$	KOAc	MeCN	24 h	66%	66%[c)]
6	[Ir(Fppy)$_2$(pic)]	NaOAc	MeCN	24 h	30%	33%
7	Ir(Fppy)$_3$	NaOAc	MeCN	24 h	70%	58%
8	Ir(Fppy)$_3$	None	MeCN	24 h	82%	60%
9	Ir(Fppy)$_3$	NH_4OAc	MeCN	24 h	25%	0%
10	Ir(Fppy)$_3$	Bu_4NOAc	MeCN	24 h	0%	0%
11	Ir(Fppy)$_3$	K_2CO_3	MeCN	24 h	0%	0%
12	Ir(Fppy)$_3$	K_3PO_4	MeCN	24 h	0%	0%
13	Ir(Fppy)$_3$	NaOH	MeCN	24 h	0%	0%
14	Ir(Fppy)$_3$	$PhSO_2Na$	MeCN	24 h	29%	29%
15	Ir(Fppy)$_3$	$PhCO_2Na$	MeCN	24 h	72%	62%
16	Ir(Fppy)$_3$	Cl_3CO_2Na	MeCN	24 h	69%	66%
17	Ir(Fppy)$_3$	Na_2CO_3	MeCN	24 h	74%	69%
18	Ir(Fppy)$_3$	$NaHCO_3$	MeCN	24 h	78%	76%
19	Ir(Fppy)$_3$	Cs_2CO_3	MeCN	72 h	0%	0%
20	Ir(Fppy)$_3$	Li_2CO_3	MeCN	72 h	23%	11%
21	Ir(Fppy)$_3$	$KHCO_3$	MeCN	72 h	45%	40%
22	Ir(Fppy)$_3$	$NaHCO_3$	MeCN	72 h	68%	61%
23	Ir(Fppy)$_3$	$NaHCO_3$	EtOH	24 h	0%	0%
24	Ir(Fppy)$_3$	$NaHCO_3$	DMSO	24 h	25%	12%
25	Ir(Fppy)$_3$	$NaHCO_3$	NMP[b)]	24 h	55%	16%
26	Ir(Fppy)$_3$	$NaHCO_3$	Acetone	24 h	91%	62%
27	Ir(Fppy)$_3$	KOAc	Acetone	24 h	87%	66%[c)]
28	Ir(Fppy)$_3$	$NaHCO_3$	EtOAc	24 h	76%	63%
**29**	**Ir(Fppy)$_3$**	**NaOAc**	**EtOAc**	**24 h**	**95%**	**87%**
30	Ir(Fppy)$_3$	KOAc	EtOAc	24 h	97%	87%[c)]
31	Ir(Fppy)^3	NaOAc	EtOAc	40 h	100%	(87%)[d)]

The reactions were carried out at a 0.05 mmol scale. [a)] Yields and conversion were determined by ^{1}H NMR analysis of crude reaction mixture using 1,3,5-trimethoxybenzene as an internal standard. Isolated yields are in parentheses [b)] NMP = 1-methyl-2-pyrrolidinone. [c)] Additional side product visible by ^{1}H NMR [d)] Reaction carried out at a 0.30 mmol scale.

V. Photocyclization

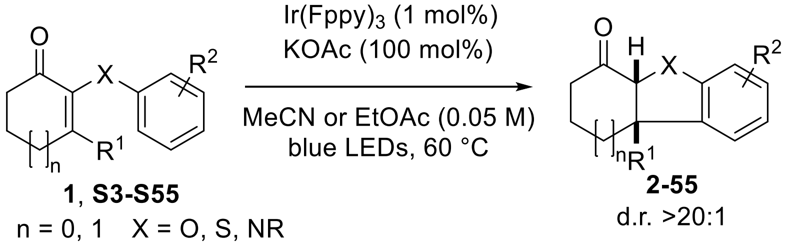

1, **S3-S55** **2-55**
n = 0, 1 X = O, S, NR d.r. >20:1

General Procedure D:

In a 7 mL screw-topped vial, the appropriate enone (1.0 eq), potassium acetate (or sodium acetate) (1.0 eq) and Ir(Fppy)$_3$ (0.01 eq) were dissolved in acetonitrile (or ethyl acetate) (degassed, 6 mL/0.3 mmol substrate) and sealed under an atmosphere of argon. The reaction mixture was stirred at 60 °C while irradiating with a 12 W blue LED lamp (λ = 450 nm) for the appropriate time. The solvent was removed under reduced pressure and the residue was purified *via* flash chromatography (see experimental methods section for specific details) to afford the corresponding dihydrobenzofuran.

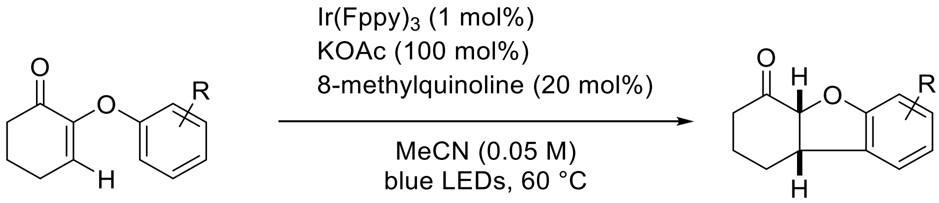

S28-S34 **28-34**
d.r. >20:1

General Procedure E:

In a 7 mL screw-topped vial, the appropriate enone (1.0 eq), potassium acetate (1.0 eq), 8-methylquinoline (0.2 eq) and Ir(Fppy)$_3$ (0.01 eq) were dissolved in acetonitrile (degassed, 6 mL/0.3 mmol substrate) and sealed under an atmosphere of argon. The reaction mixture was stirred at 60 °C while irradiating with a 12 W blue LED lamp (λ = 450 nm) for the appropriate time. The reaction mixture was then poured onto 1 M HCl (30 mL/mmol enone) and extracted with Et_2O (3 × 50 mL/mmol enone). The combined organic extracts were washed with brine, dried (Na_2SO_4) and concentrated under reduced pressure. Purification of the residue *via* flash chromatography (see experimental methods section for specific details) afforded the corresponding dihydrobenzofuran. N.B: The corresponding benzofuran which is formed as a minor by-product (~5%) in this reaction is usually hard to separate from the desired product due to very similar R_f-values.

(4a*R,9b*S**)-9b-Methyl-2,3,4a,9b-tetrahydrodibenzo[*b*,*d*]furan-4(*1H*)-one (2)**

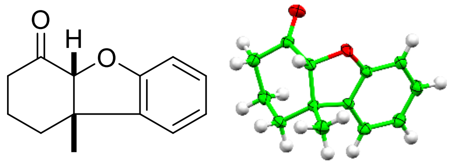

Prepared according to **General Procedure D** with **1** (60.7 mg, 0.30 mmol), KOAc (29 mg, 0.30 mmol), Ir(Fppy)$_3$ (2.3 mg, 0.003 mmol) and acetonitrile (6 mL). Reaction time: 16 h. Purification *via* flash chromatography (PE:Et$_2$O, 2:1) afforded the title compound **2** as a colourless solid (57.2 mg, 0.28 mmol, 94%, >20:1 d.r.).

Gram scale, with 0.05 mol% catalyst: Prepared according to **General Procedure D** (in a 21 mL screw-topped vial) with **1** (1.01 g, 5.00 mmol), KOAc (491 mg, 5.00 mmol), Ir(Fppy)$_3$ (1.9 mg, 0.0025 mmol) and acetonitrile (20 mL). Reaction time: 16 h. Purification *via* flash chromatography (PE:Et$_2$O, 2:1) afforded the title compound **2** as a colourless solid (931 mg, 4.60 mmol, 92%, >20:1 d.r.). The spectral data for **2** matched those obtained on a smaller scale.

m.p. 99-100 °C (CHCl$_3$).

^{1}H NMR (400 MHz, CDCl$_3$) δ = 7.20-7.13 (m, 1H), 7.07-7.02 (m, 1H), 6.97-6.89 (m, 2H), 4.46 (s, 1H), 2.61-2.51 (m, 1H), 2.36 (ddd, J = 15.9, 9.9, 6.3 Hz, 1H), 2.09-1.99 (m, 1H), 1.97-1.77 (m, 2H), 1.75-1.61 (m, 1H), 1.44 (s, 3H).

^{13}C NMR (101 MHz, CDCl$_3$) δ = 208.7, 159.0, 133.6, 128.8, 122.1, 121.8, 110.5, 91.8, 50.2, 38.4, 34.5, 28.3, 20.8.

FTIR (neat) ν/cm^{-1} = 2959, 2925, 1725, 1595, 1474, 1459, 1251, 1198, 1183, 1029, 987, 829, 755.

HRMS (ESI) *m/z* calcd. for $C_{13}H_{14}O_2Na$ [M+Na]$^+$: 225.0886; found: 225.0886.

(4a*R,9b*S**)-6,9b-Dimethyl-2,3,4a,9b-tetrahydrodibenzo[*b*,*d*]furan-4(1*H*)-one (3)**

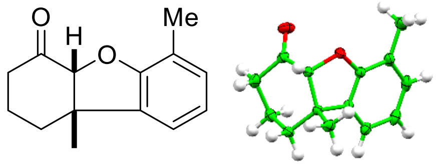

Prepared according to **General Procedure D** with **S3** (64.9 mg, 0.30 mmol), KOAc (29 mg, 0.30 mmol), Ir(Fppy)$_3$ (2.3 mg, 0.003 mmol) and acetonitrile (6 mL). Reaction time: 16 h. Purification *via* flash chromatography (PE:Et$_2$O, 2:1) afforded the title compound **3** as a colourless solid (58.4 mg, 0.27 mmol, 90%, >20:1 d.r.).

m.p. 93-94°C (CHCl$_3$).

^{1}H NMR (400 MHz, CDCl$_3$) δ = 7.01-6.95 (m, 1H), 6.91-6.81 (m, 2H), 4.42 (s, 1H), 2.62-2.52 (m, 1H), 2.35 (ddd, J = 16.0, 9.8, 6.4 Hz, 1H), 2.27 (s, 3H), 2.09-1.96 (m, 1H), 1.92-1.74 (m, 2H), 1.73-1.60 (m, 1H), 1.43 (s, 3H).

^{13}C NMR (101 MHz, CDCl$_3$) δ = 208.7, 157.5, 132.9, 130.0, 121.7, 120.7, 119.4, 91.6, 50.4, 38.4, 34.8, 28.0, 20.7, 15.3.

FTIR (neat) ν/cm^{-1} = 2925, 2867, 1726, 1596, 1460, 1309, 1260, 1197, 1177, 1138, 1067, 1032, 989, 913, 858, 827, 775.

HRMS (ESI) *m/z* calcd. for $C_{14}H_{16}O_2Na$ [M+Na]$^+$: 239.1043; found: 239.1043.

(4a*R,9b*S**)-6,8-Difluoro-9b-methyl-2,3,4a,9b-tetrahydrodibenzo[*b,d*]furan-4(1*H*)-one (4)**

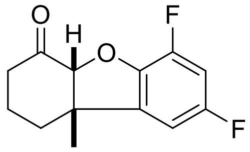

Prepared according to **General Procedure D** with **S4** (71.5 mg, 0.30 mmol), KOAc (29 mg, 0.30 mmol), Ir(Fppy)$_3$ (2.3 mg, 0.003 mmol) and acetonitrile (6 mL). Reaction time: 16 h. Purification *via* flash chromatography (PE:EtOAc, 3:1) afforded the title compound **4** as a colourless solid (62.3 mg, 0.26 mmol, 90%, >20:1 d.r.).

m.p. 118-120 °C ($CHCl_3$).

^{1}H NMR (400 MHz, $CDCl_3$) δ = 6.72 (ddd, *J* = 10.1, 9.1, 2.4 Hz, 1H), 6.58 (ddd, *J* = 7.3, 2.4, 1.0 Hz, 1H), 4.55 (s, 1H), 2.65-2.56 (m, 1H), 2.38 (ddd, *J* = 15.9, 9.7, 6.3 Hz, 1H), 2.05-1.88 (m, 2H), 1.88-1.78 (m, 1H), 1.75-1.62 (m, 1H).1.45 (s, 3H).

^{13}C NMR (101 MHz, $CDCl_3$) δ = 206.9, 157.7 (dd, *J* = 242, 8.1 Hz), 146.8 (dd, *J* = 251, 12.0 Hz), 137.1, 104.8 (dd, *J* = 24.5, 3.9 Hz), 104.2 (dd, *J* = 27.8, 21.0 Hz), 92.2, 51.3, 38.3, 34.4, 27.6, 20.8.

^{19}F NMR (377 MHz, $CDCl_3$) δ = −118.5 (t, *J* = 8.8 Hz), −132.8 (d, *J* = 10.1 Hz).

FTIR (neat) ν/cm^{-1} = 2930, 1718, 1638, 1613, 1484, 1453, 1378, 1351, 1324, 1313, 1225, 1180, 1157, 1109, 1079, 1030, 998, 985, 968, 916, 891, 873, 851.

HRMS (ESI) *m/z* calcd. for $C_{13}H_{12}O_2F_2Na$ [M+Na]$^+$: 261.0698; found: 261.0698.

(4a*R,9b*S**)-6-Chloro-9b-methyl-2,3,4a,9b-tetrahydrodibenzo[*b,d*]furan-4(1*H*)-one (5)**

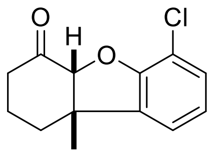

Prepared according to **General Procedure D** with **S5** (71.0 mg, 0.30 mmol), KOAc (29 mg, 0.30 mmol), Ir(Fppy)$_3$ (2.3 mg, 0.003 mmol) and acetonitrile (6 mL). Reaction time: 16 h. Purification *via* flash chromatography (PE:Et_2O, 2:1) afforded the title compound **5** as a colourless solid (63.2 mg, 0.27 mmol, 89%, >20:1 d.r.).

m.p. 80-82 °C ($CHCl_3$).

^{1}H NMR (400 MHz, $CDCl_3$) δ = 7.17 (dd, *J* = 7.8, 1.3 Hz, 1H), 6.94 (dd, *J* = 7.4, 1.3 Hz, 1H), 6.88 (d, *J* = 7.6, 7.6 Hz, 1H), 4.53 (s, 1H), 2.66-2.57 (m, 1H), 2.37 (ddd, *J* = 16.0, 9.6, 6.5 Hz, 1H), 2.05-1.96 (m, 1H), 1.95-1.85 (m, 1H), 1.85-1.75 (m, 1H), 1.74-1.60 (m, 1H), 1.45 (s, 3H).

^{13}C NMR (101 MHz, $CDCl_3$) δ = 207.2, 155.0, 135.6, 129.2, 122.9, 120.5, 115.9, 92.2, 51.2, 38.1, 34.8, 27.8, 20.7.

FTIR (neat) ν/cm^{-1} = 2960, 1929, 1725, 1606, 1588, 1449, 1378, 1320, 1259, 1230, 1213, 1190, 1146, 1109, 1095, 1053, 1026, 986, 932, 904, 865, 827, 777.

HRMS (ESI) *m/z* calcd. for $C_{13}H_{13}O_2ClNa$ [M+Na]$^+$: 259.0496; found: 259,0497.

(4a*R,9b*S**)-6-Bromo-9b-methyl-2,3,4a,9b-tetrahydrodibenzo[*b*,*d*]furan-4(1*H*)-one (6)**

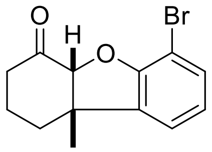

Prepared according to **General Procedure D** with **S6** (84.0 mg, 0.30 mmol), KOAc (29 mg, 0.30 mmol), Ir(Fppy)$_3$ (2.3 mg, 0.003 mmol) and acetonitrile (6 mL). Reaction time: 16 h. Purification *via* flash chromatography (PE:Et_2O, 2:1) afforded the title compound **6** as a colourless solid (77.2 mg, 0.28 mmol, 92%, >20:1 d.r.).

m.p. 88-90 °C ($CHCl_3$).

^{1}H NMR (400 MHz, $CDCl_3$) δ = 7.32 (dd, *J* = 7.9, 1.2 Hz, 1H), 6.98 (dd, *J* = 7.4, 1.2 Hz, 1H), 6.83 (dd, *J* = 7.8, 7.6 Hz, 1H), 4.52 (s, 1H), 2.66-2.57 (m, 1H), 2.36 (ddd, *J* = 15.9, 9.5, 6.6 Hz, 1H), 2.04-1.95 (m, 1H), 1.95-1.85 (m, 1H), 1.85-1.75 (m, 1H), 1.75-1.61 (m, 1H), 1.44 (s, 3H).

^{13}C NMR (101 MHz, $CDCl_3$) δ = 207.2, 156.4, 135.3, 132.0, 123.3, 121.1, 103.4, 92.0, 51.4, 38.1, 34.8, 27.8, 20.7.

FTIR (neat) ν/cm^{-1} = 2961, 1713, 1603, 1583, 1463, 1453, 1438, 1424, 1323, 1261, 1233, 1220, 1189, 1171, 1104, 1089, 1053, 1023, 983, 973, 935, 903, 889, 859, 828, 776.

HRMS (ESI) *m/z* calcd. for $C_{13}H_{14}O_2Br$ [M+H]$^+$: 281.0172; found: 281.0173.

(4a*R,9b*S**)-6-Iodo-9b-methyl-2,3,4a,9b-tetrahydrodibenzo[*b*,*d*]furan-4(1*H*)-one (7)**

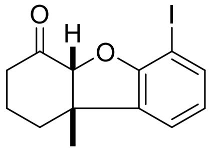

Prepared according to **General Procedure D** with **S7** (98.4 mg, 0.30 mmol), KOAc (29 mg, 0.30 mmol), Ir(Fppy)$_3$ (2.3 mg, 0.003 mmol) and acetonitrile (6 mL). Reaction time: 16 h. Purification *via* flash chromatography (PE:Et_2O, 1:1) afforded the title compound **7** as a colourless solid (77.4 mg, 0.24 mmol, 78%, >20:1 d.r.).

m.p. 120-103 °C ($CHCl_3$).

^{1}H NMR (400 MHz, $CDCl_3$) δ = 7.51 (dd, *J* = 7.8, 1.4 Hz, 1H), 7.00 (dd, *J* = 7.3, 1.1 Hz, 1H), 6.70 (dd, *J* = 7.6, 7.6 Hz, 1H), 4.51 (s, 1H), 2.65-2.55 (m, 1H), 2.36 (ddd, *J* = 16.0, 9.8, 6.3 Hz, 1H), 2.04-1.95 (m, 1H), 1.95-1.84 (m, 1H), 1.84-1.73 (m, 1H), 1.73-1.58 (m, 1H), 1.43 (s, 3H).

^{13}C NMR (101 MHz, $CDCl_3$) δ = 207.2, 159.5, 137.5, 133.8, 123.6, 122.0, 91.3, 74.5, 51.6, 38.1, 34.7, 27.8, 20.6.

FTIR (neat) ν/cm^{-1} = 2960, 1723, 1597, 1432, 1208, 1185, 1085, 1083, 1050, 1026, 987, 970, 901, 864, 829, 772.

HRMS (ESI) *m/z* calcd. for $C_{13}H_{13}O_2INa$ $[M+Na]^+$: 350.9852; found: 350.9853.

(4a*R,9b*S**)-9-Allyl-6-methoxy-9b-methyl-2,3,4a,9b-tetrahydrodibenzo[*b,d*]furan-4(1*H*)-one (8)**

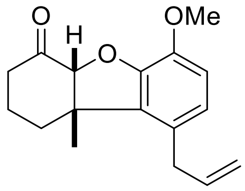

Prepared according to **General Procedure D** with **S8** (81.7 mg, 0.30 mmol), KOAc (29 mg, 0.30 mmol), Ir(Fppy)$_3$ (2.3 mg, 0.003 mmol) and acetonitrile (6 mL). Reaction time: 16 h. Purification *via* flash chromatography (PE:Et_2O, 1:1) afforded the title compound **8** as a colourless solid (71.0 mg, 0.26 mmol, 87%, >20:1 d.r.).

m.p. 54-55 °C ($CHCl_3$).

^{1}H NMR (400 MHz, $CDCl_3$) δ = 6.75 (d, *J* = 8.3 Hz, 1H), 6.68 (d, *J* = 8.3 Hz, 1H), 6.00-5.87 (m, 1H), 5.08 (dq, *J* = 10.1, 1.5 Hz, 1H), 5.01 (dq, *J* = 17.1, 1.7 Hz, 1H), 4.41 (s, 1H), 3.88 (s, 3H), 3.36 (dd, *J* = 6.2, 1.6 Hz, 2H), 2.70-2.55 (m, 1H), 2.46-2.32 (m, 1H), 2.13-1.99 (m, 1H), 1.96-1.72 (m, 3H), 1.51 (s, 3H).

^{13}C NMR (101 MHz, $CDCl_3$) δ = 208.3, 147.5, 143.4, 137.3, 131.9, 127.9, 123.7, 116.3, 112.1, 92.2, 56.2, 51.8, 37.9, 35.2, 34.3, 26.5, 20.3.

FTIR (neat) ν/cm^{-1} = 2956, 1723, 1625, 1582, 1504, 1460, 1429, 1223, 1194, 1167, 1145, 1117, 1050, 996, 952, 910 863, 848, 802, 731, 686.

HRMS (ESI) *m/z* calcd. for $C_{17}H_{21}O_3$ $[M+H]^+$: 273.1485; found: 273.1481.

(4a*R,9b*S**)-8-Allyl-6-methoxy-9b-methyl-2,3,4a,9b-tetrahydrodibenzo[*b,d*]furan-4(1*H*)-one (9)**

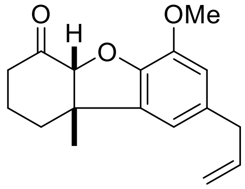

Prepared according to **General Procedure D** with **S9** (81.7 mg, 0.30 mmol), KOAc (29 mg, 0.30 mmol), Ir(Fppy)$_3$ (2.3 mg, 0.003 mmol) and acetonitrile (6 mL). Reaction time: 16 h. Purification *via* flash chromatography (PE:Et_2O, 1:1) afforded the title compound **9** as a colourless solid (71.1 mg, 0.26 mmol, 87%, >20:1 d.r.).

m.p. 92-93 °C ($CHCl_3$).

^{1}H NMR (400 MHz, $CDCl_3$) δ = 6.60 (s, 1H), 6.49 (s, 1H), 6.01-5.87 (m, 1H), 5.12-5.02 (m, 2H), 4.45 (s, 1H), 3.88 (s, 3H), 3.33 (d, *J* = 6.7 Hz, 2H), 2.68-2.57 (m, 1H), 2.40-2.28 (m, 1H), 2.03-1.92 (m, 1H), 1.91-1.79 (m, 1H), 1.79-1.64 (m, 2H), 1.42 (s, 3H).

^{13}C NMR (101 MHz, $CDCl_3$) δ = 208.2, 145.6, 144.5, 137.8, 135.0, 134.8, 115.9, 114.0, 112.5, 92.6, 56.2, 51.0, 40.3, 38.3, 34.9, 27.4, 20.7.

FTIR (neat) ν/cm^{-1} = 2923, 2851, 1723, 1605, 1490, 1452, 1323, 1281, 1227, 1204, 1185, 1138, 1058, 1029, 991, 912, 848, 808, 748.

HRMS (ESI) *m/z* calcd. for $C_{17}H_{20}O_3Na$ [M+Na]$^+$: 295.1305; found: 295.1302.

(5b*S,9a*R**)-5b-Methyl-1,2,5b,7,8,9a-hexahydro-3*H*-indeno[4,5-*b*]benzofuran-3,9(6*H*)-dione (10)**

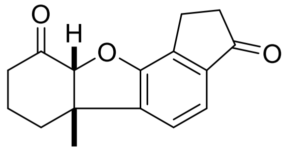

Prepared according to **General Procedure D** with **S10** (76.9 mg, 0.30 mmol), KOAc (29 mg, 0.30 mmol), Ir(Fppy)$_3$ (2.3 mg, 0.003 mmol) and acetonitrile (6 mL). Reaction time: 16 h. Purification *via* flash chromatography (PE:EtOAc, 1:1) afforded the title compound **10** as a pale yellow solid (71.7 mg, 0.28 mmol, 93%, >20:1 d.r.).

m.p. 149-151 °C ($CHCl_3$).

^{1}H NMR (400 MHz, $CDCl_3$) δ = 7.40 (d, *J* = 7.6 Hz, 1H), 7.08 (d, *J* = 7.6 Hz, 1H), 4.58 (s, 1H), 3.22-3.03 (m, 2H), 2.71 (t, *J* = 5.9 Hz, 2H), 2.61-2.52 (m, 1H), 2.45-2.34 (m, 1H), 2.15-2.05 (m, 1H), 2.01-1.84 (m, 2H), 1.72-1.59 (m, 1H), 1.47 (s, 3H).

^{13}C NMR (101 MHz, $CDCl_3$) δ = 207.6, 206.3, 156.0, 139.5, 139.1, 136.9, 121.4, 117.8, 92.3, 50.5, 38.4, 36.5, 34.3, 28.2, 22.1, 20.9.

FTIR (neat) ν/cm^{-1} = 2928, 1705, 1690, 1619, 1587, 1463, 1449, 1429, 1401, 1355, 1338, 1314, 1283, 1263, 1221, 1199, 1173, 1100, 1049, 998, 965, 899, 848, 817, 744, 732, 703.

HRMS (ESI) *m/z* calcd. for $C_{16}H_{17}O_3$ [M+H]$^+$: 257.1172; found: 257.1173.

(4a*R,9b*S**)-9,9b-Dimethyl-2,3,4a,9b-tetrahydrodibenzo[*b,d*]furan-4(1*H*)-one and (4a*R**,9b*S**)-7,9b-dimethyl-2,3,4a,9b-tetrahydrodibenzo[*b,d*]furan-4(1*H*)-one (11)**

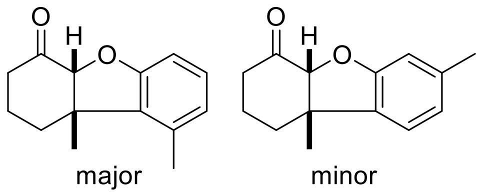

Prepared according to **General Procedure D** with **S11** (64.9 mg, 0.30 mmol), KOAc (29 mg, 0.30 mmol), Ir(Fppy)$_3$ (2.3 mg, 0.003 mmol) and acetonitrile (6 mL). Reaction time: 16 h. Purification *via* flash chromatography (PE:Et_2O, 2:1) afforded the title compound **11** as a regioisomeric mixture in form of a colourless oil (r.r. 2.2:1, 59.0 mg, 0.27 mmol, 91%, >20:1 d.r.).

<u>Analytical Data for Major Regioisomer:</u>

^{1}H NMR (400 MHz, $CDCl_3$) δ = 7.05 (dd, *J* = 7.8, 7.8 Hz, 1H), 6.79-6.72 (m, 1H), 6.68 (d, *J* = 7.6 Hz, 1H), 4.40 (s, 1H), 2.62-2.49 (m, 1H), 2.49-2.33 (m, 1H), 2.32 (s, 3H), 2.15-1.58 (m, 4H), 1.52 (s, 3H).

^{13}C NMR (101 MHz, $CDCl_3$) δ = 209.0, 159.2, 134.2, 130.6, 128.7, 124.2, 108.3, 91.6, 50.9, 38.0, 33.6, 26.1, 20.1, 18.3.

Analytical Data for Regioisomeric Mixture:

FTIR (neat) ν/cm^{-1} = 2957, 2866, 1722, 1613, 1589, 1457, 1379, 1310, 1245, 1202, 1142, 1066, 1029, 991, 946, 924, 862, 806, 774, 741.

HRMS (ESI) *m/z* calcd. for $C_{14}H_{16}O_2Na$ [M+Na]$^+$: 239.1043; found: 239.1043.

(4a*R,9b*S**)-9-(Dimethylamino)-9b-methyl-2,3,4a,9b-tetrahydrodibenzo[*b,d*]furan-4(1*H*)-one (12a) and (4a*R**,9b*S**)-7-(dimethylamino)-9b-methyl-2,3,4a,9b-tetrahydrodibenzo[*b,d*]furan-4(1*H*)-one (12b)**

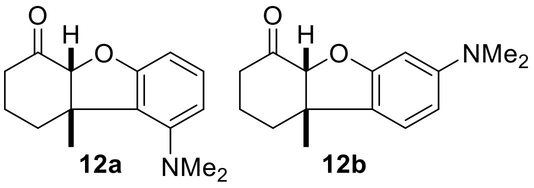

Prepared according to **General Procedure D** with **S12** (73.6 mg, 0.30 mmol), KOAc (29 mg, 0.30 mmol), Ir(Fppy)$_3$ (2.3 mg, 0.003 mmol) and acetonitrile (6 mL). Reaction time: 16 h. Purification *via* flash chromatography (PE:Et_2O, 2:1) afforded the title compounds **12a** (35.2 mg, 0.14 mmol, 48%, >20:1 d.r.) and **12b** (15.2 mg, 0.062 mmol, 21%, >20:1 d.r.) as colourless solids.

Analytical Data for **12a**:

m.p. 57-59 °C ($CHCl_3$).

^{1}H NMR (400 MHz, $CDCl_3$) δ = 7.13 (dd, *J* = 7.9, 7.9 Hz, 1H), 6.83 (d, *J* = 8.0 Hz, 1H), 6.72 (d, *J* = 7.8 Hz, 1H), 4.37 (s, 1H), 2.62 (s, 6H), 2.57-2.44 (m, 2H), 2.41-2.32 (m, 1H), 1.99-1.83 (m, 2H), 1.77-1.64 (m, 1H), 1.56 (s, 3H).

^{13}C NMR (101 MHz, $CDCl_3$) δ = 209.1, 160.0, 152.9, 129.6, 128.2, 115.8, 107.4, 92.0, 51.5, 46.5, 38.5, 33.4, 27.3, 21.1.

FTIR (neat) ν/cm^{-1} = 2936, 1723, 1606, 1588, 1483, 1438, 1308, 1252, 1190, 1038, 1010, 901, 864, 780, 741, 669.

HRMS (ESI) *m/z* calcd. for $C_{15}H_{20}O_2N$ [M+H]$^+$: 246.1489; found: 246.1489.

Analytical Data for **12b**:

m.p. 109-111 °C ($CHCl_3$).

^{1}H NMR (400 MHz, $CDCl_3$) δ = 6.88 (d, *J* = 8.3 Hz, 1H), 6.35 (d, *J* = 2.5 Hz, 1H), 6.29 (dd, *J* = 8.2, 2.3 Hz, 1H), 4.42 (s, 1H), 2.92 (s, 6H), 2.59-2.49 (m, 1H), 2.33 (ddd, *J* = 15.9, 9.8, 6.4 Hz, 1H), 2.06-1.97 (m, 1H), 1.93-1.61 (m, 3H), 1.39 (s, 3H).

^{13}C NMR (101 MHz, $CDCl_3$) δ = 209.2, 160.6, 152.0, 122.1, 121.0, 106.0, 95.2, 92.2, 49.8, 40.9, 38.6, 34.7, 28.5, 20.9.

FTIR (neat) ν = 2924, 1722, 1625, 1582, 1509, 1446, 1356, 1321, 1245, 1218, 1178, 1139, 1067, 990, 926, 902, 859, 818, 788.

HRMS (ESI) *m/z* calcd. for $C_{15}H_{20}O_2N$ [M+H]$^+$: 246.1489; found: 246.1485.

(4a*R,9b*S**)-7,9-Dimethoxy-9b-methyl-2,3,4a,9b-tetrahydrodibenzo[*b,d*]furan-4(1*H*)-one (13)**

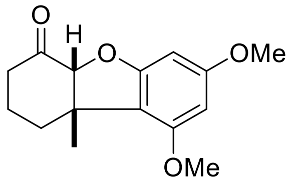

Prepared according to **General Procedure D** with **S13** (78.7 mg, 0.30 mmol), KOAc (29 mg, 0.30 mmol), Ir(Fppy)$_3$ (2.3 mg, 0.003 mmol) and acetonitrile (6 mL). Reaction time: 16 h. Purification *via* flash chromatography (PE:Et_2O, 1:1) afforded the title compound **13** as a colourless solid (74.2 mg, 0.28 mmol, 94%, >20:1 d.r.).

m.p. 67-69 °C ($CHCl_3$).

^{1}H NMR (400 MHz, $CDCl_3$) δ = 6.15 (s, 1H), 6.02 (s, 1H), 4.39 (s, 1H), 3.76 (s, 3H), 3.75 (s, 3H), 2.61-2.47 (m, 1H), 2.41-2.21 (m, 2H), 1.97-1.82 (m, 1H), 1.80-1.62 (m, 2H), 1.46 (s, 3H).

^{13}C NMR (101 MHz, $CDCl_3$) δ = 209.1, 162.1, 161.1, 157.0, 111.4, 92.5, 92.5, 88.9, 55.7, 55.4, 50.5, 38.3, 33.1, 26.6, 20.6.

FTIR (neat) ν/cm^{-1} = 2955, 1724, 1621, 1597, 1499, 1456, 1424, 1349, 1209, 1145, 1115, 1083, 1037, 993, 862.

HRMS (ESI) *m/z* calcd. for $C_{15}H_{18}O_4Na$ [M+Na]$^+$: 285.1097; found: 285.1098.

(4a*R,9b*S**)-9b-Methyl-7,9-bis(trifluoromethyl)-2,3,4a,9b-tetrahydrodibenzo[*b,d*]furan-4(1*H*)-one (14)**

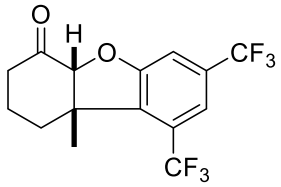

Prepared according to **General Procedure D** with **S14** (101.5 mg, 0.30 mmol), KOAc (29 mg, 0.30 mmol), Ir(Fppy)$_3$ (2.3 mg, 0.003 mmol) and acetonitrile (6 mL). Reaction time: 16 h. Purification *via* flash chromatography (PE:Et_2O, 2:1) afforded the title compound **14** as a colourless solid (93.5 mg, 0.28 mmol, 92%, >20:1 d.r.).

m.p. 101-103 °C ($CHCl_3$).

^{1}H NMR (400 MHz, $CDCl_3$) δ = 7.46 (s, 1H), 7.38 (s, 1H), 4.61 (s, 1H), 2.59-2.40 (m, 2H), 2.32-2.21 (m, 1H), 2.12-1.97 (m, 2H), 1.86-1.72 (m, 1H), 1.55 (s, 3H).

^{13}C NMR (101 MHz, $CDCl_3$) δ = 206.7, 161.1, 135.1, 132.3, 131.9, 123.2 (q, *J* = 276 Hz), 123.1 (q, *J* = 265 Hz), 116.7, 111.9, 92.7, 52.3, 38.8, 32.8, 27.0, 20.9.

^{19}F NMR (377 MHz, $CDCl_3$) δ = −58.4, −62.9.

FTIR (neat) ν/cm^{-1} = 2943, 1726, 1591, 1466, 1432, 1410, 1384, 1352, 1335, 1318, 1277, 1244, 1229, 1211, 1167, 1142, 1116, 1097, 1075, 1030, 999, 984, 922, 899, 850, 833, 750.

HRMS (ESI) *m/z* calcd. for $C_{15}H_{12}O_2F_6Na$ [M+Na]$^+$: 361.0634; found: 361.0633.

(5a*R,9a*S**)-9a-Methyl-7,8,9,9a-tetrahydrobenzofuro[3,2-c]pyridin-6(5a*H*)-one (15)**

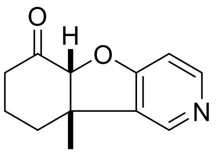

Prepared according to **General Procedure D** with **S15** (61.0 mg, 0.30 mmol), KOAc (29 mg, 0.30 mmol), Ir(Fppy)$_3$ (2.3 mg, 0.003 mmol) and acetonitrile (6 mL). Reaction time: 72 h. Purification *via* flash chromatography ($CHCl_3$:MeOH, 40:1) afforded the title compound **15** as a colourless solid (46.4 mg, 0.23 mmol, 76%, >20:1 d.r.).

m.p. 133-134 °C ($CHCl_3$).

^{1}H NMR (400 MHz, $CDCl_3$) δ = 8.38 (d, *J* = 5.5 Hz, 1H), 8.26 (s, 1H), 6.88 (d, *J* = 5.5 Hz, 1H), 4.54 (s, 1H), 2.61-2.51 (m, 1H), 2.39 (ddd, *J* = 16.1, 9.7, 6.6 Hz, 1H), 2.13-2.04 (m, 1H), 2.03-1.86 (m, 2H), 1.75-1.62 (m, 1H), 1.50 (s, 3H).

^{13}C NMR (101 MHz, $CDCl_3$) δ = 206.9, 165.8, 150.8, 144.1, 130.5, 106.6, 92.4, 49.0, 38.0, 34.4, 28.4, 20.6.

FTIR (neat) ν/cm^{-1} = 2963, 1728, 1608, 1586, 1477, 1455, 1423, 1283, 1165, 1023, 983, 967, 902, 864, 844, 823.

HRMS (ESI) *m/z* calcd. for $C_{12}H_{14}O_2N$ $[M+H]^+$: 204.1019; found: 204.1021.

(5a*R,9a*S**)-9a-Methyl-6-oxo-5a,6,7,8,9,9a-hexahydrodibenzo[*b,d*]furan-2-carbaldehyde (16)**

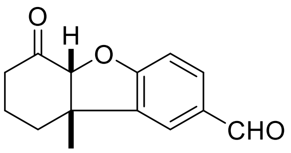

Prepared according to **General Procedure D** with **S16** (69.1 mg, 0.30 mmol), KOAc (29 mg, 0.30 mmol), Ir(Fppy)$_3$ (2.3 mg, 0.003 mmol) and acetonitrile (6 mL). Reaction time: 16 h. Purification *via* flash chromatography (PE:Et_2O, 1:2) afforded the title compound **16** as a colourless solid (51.9 mg, 0.23 mmol, 75%, >20:1 d.r.).

m.p. 104-106 °C ($CHCl_3$).

^{1}H NMR (400 MHz, $CDCl_3$) δ = 9.86 (s, 1H), 7.72 (dd, *J* = 8.2, 1.6 Hz, 1H), 7.65 (d, *J* = 1.6 Hz, 1H), 7.04 (d, *J* = 8.2 Hz, 1H), 4.61 (s, 1H), 2.60-2.51 (m, 1H), 2.39 (ddd, *J* = 16.0, 9.9, 6.3 Hz, 1H), 2.15-2.06 (m, 1H), 2.02-1.86 (m, 2H), 1.72-1.59 (m, 1H), 1.47 (s, 3H).

^{13}C NMR (101 MHz, $CDCl_3$) δ = 207.1, 190.6, 164.3, 135.3, 134.1, 131.6, 122.6, 110.8, 92.6, 49.6, 38.2, 34.1, 28.4, 20.7.

FTIR (neat) ν/cm^{-1} = 2962, 1725, 1683, 1605, 1588, 1478, 1455, 1438, 1335, 1264, 1193, 1122, 1063, 1024, 986, 969, 916, 860.

HRMS (ESI) *m/z* calcd. for $C_{14}H_{15}O_3$ $[M+H]^+$: 231.1016; found: 231.1017.

Ethyl (5a*R,9a*S**)-9a-methyl-6-oxo-5a,6,7,8,9,9a-hexahydrodibenzo[*b,d*]furan-2-carboxylate (17)**

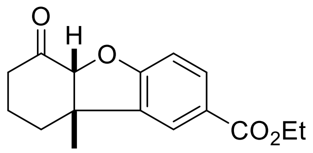

Prepared according to **General Procedure D** with **S17** (82.3 mg, 0.30 mmol), KOAc (29 mg, 0.30 mmol), Ir(Fppy)$_3$ (2.3 mg, 0.003 mmol) and acetonitrile (6 mL). Reaction time: 16 h. Purification *via* flash chromatography (PE:Et_2O, 1:1) afforded the title compound **17** as a colourless solid (78.1 mg, 0.28 mmol, 95%, >20:1 d.r.).

m.p. 67-69 °C ($CHCl_3$).

^{1}H NMR (400 MHz, $CDCl_3$) δ = 7.93 (dd, *J* = 8.4, 1.8 Hz, 1H), 7.76 (d, *J* = 1.7 Hz, 1H), 6.94 (d, *J* = 8.4 Hz, 1H), 4.56 (s, 1H), 4.35 (q, *J* = 7.1 Hz, 2H), 2.61-2.51 (m, 1H), 2.38 (ddd, *J* = 15.9, 9.9, 6.3 Hz, 1H), 2.15-2.05 (m, 1H), 1.99-1.83 (m, 2H), 1.73-1.59 (m, 1H), 1.46 (s, 3H), 1.38 (t, *J* = 7.1 Hz, 3H).

^{13}C NMR (101 MHz, $CDCl_3$) δ = 207.7, 166.4, 162.9, 134.1, 131.7, 124.5, 124.1, 110.2, 92.5, 61.0, 49.9, 38.3, 34.3, 28.5, 20.8, 14.5.

FTIR (neat) ν/cm^{-1} = 2960, 1707, 1610, 1478, 1456, 1390, 1366, 1336, 1276, 1240, 1216, 1189, 1172, 1100, 1065, 1024, 985, 912, 861, 836, 772.

HRMS (ESI) *m/z* calcd. for $C_{16}H_{18}O_4Na$ $[M+Na]^+$: 297.1097; found: 297.1097.

(5a*R,9a*S**)-9a-Methyl-6-oxo-5a,6,7,8,9,9a-hexahydrodibenzo[*b,d*]furan-2-carbonitrile (18)**

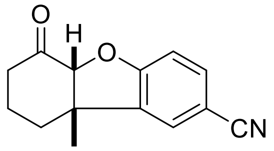

Prepared according to **General Procedure D** with **S18** (68.2 mg, 0.30 mmol), KOAc (29 mg, 0.30 mmol), Ir(Fppy)$_3$ (2.3 mg, 0.003 mmol) and acetonitrile (6 mL). Reaction time: 16 h. Purification *via* flash chromatography (PE:Et_2O, 1:1) afforded the title compound **18** as a colourless solid (58.3 mg, 0.26 mmol, 86%, >20:1 d.r.).

m.p. 144-146 °C ($CHCl_3$).

^{1}H NMR (400 MHz, $CDCl_3$) δ = 7.50 (dd, *J* = 8.3, 1.7 Hz, 1H), 7.33 (d, *J* = 1.6 Hz, 1H), 6.99 (d, *J* = 8.4 Hz, 1H), 4.59 (s, 1H), 2.60-2.50 (m, 1H), 2.40 (ddd, *J* = 15.9, 10.0, 6.1 Hz, 1H), 2.10-1.85 (m, 3H), 1.71-1.58 (m, 1H), 1.46 (s, 3H).

^{13}C NMR (101 MHz, $CDCl_3$) δ = 206.9, 162.5, 135.4, 134.2, 126.4, 119.3, 111.6, 105.2, 92.3, 50.0, 38.2, 34.1, 28.4, 20.7.

FTIR (neat) ν/cm^{-1} = 2964, 2222, 1724, 1610, 1478, 1455, 1329, 1312, 1265, 1219, 1190, 1067, 1025, 986, 969, 911, 856.

HRMS (ESI) *m/z* calcd. for $C_{14}H_{14}O_2N$ $[M+H]^+$: 228.1019; found: 228.1019.

(4a*R,9b*S**)-8-Methoxy-9b-methyl-2,3,4a,9b-tetrahydrodibenzo[*b,d*]furan-4(1*H*)-one (19)**

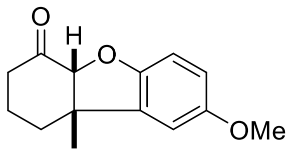

Prepared according to **General Procedure D** with **S19** (69.7 mg, 0.30 mmol), KOAc (29 mg, 0.30 mmol), Ir(Fppy)$_3$ (2.3 mg, 0.003 mmol) and acetonitrile (6 mL). Reaction time: 16 h. Purification *via* flash chromatography (PE:Et$_2$O, 1:1) afforded the title compound **19** as a colourless solid (62.6 mg, 0.27 mmol, 90%, >20:1 d.r.).

m.p. 102 °C (CHCl$_3$).

^{1}H NMR (400 MHz, CDCl$_3$) δ = 6.83 (d, *J* = 8.6 Hz, 1H), 6.69 (dd, *J* = 8.6, 2.7 Hz, 1H), 6.81 (d, *J* = 2.6 Hz, 1H), 4.44 (s, 1H), 3.76 (s, 3H), 2.61-2.49 (m, 1H), 2.41-2.29 (m, 1H), 2.10-1.97 (m, 1H), 1.96-1.76 (m, 2H), 1.76-1.61 (m, 1H), 1.43 (s, 3H).

^{13}C NMR (101 MHz, CDCl$_3$) δ = 208.8, 155.2, 153.0, 134.6, 113.2, 110.5, 108.5, 92.1, 56.1, 50.6, 38.6, 34.4, 28.2, 20.9.

FTIR (neat) ν/cm^{-1} = 2972, 1708, 1484, 1468, 1452, 1425, 1372, 1298, 1260, 1239, 1220, 1177, 1121, 1087, 1067, 1028, 986, 970, 935, 913, 890, 856, 811, 788.

HRMS (ESI) *m/z* calcd. for $C_{14}H_{17}O_3$ [M+H]$^+$: 233.1172; found: 233.1174.

(4a*R,9b*S**)-8-(Benzyloxy)-9b-methyl-2,3,4a,9b-tetrahydrodibenzo[*b,d*]furan-4(1*H*)-one (20)**

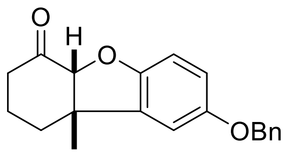

Prepared according to **General Procedure D** with **S20** (92.5 mg, 0.30 mmol), KOAc (29 mg, 0.30 mmol), Ir(Fppy)$_3$ (2.3 mg, 0.003 mmol) and acetonitrile (6 mL). Reaction time: 16 h. Purification *via* flash chromatography (PE:Et$_2$O, 1:1) afforded the title compound **20** as a colourless solid (88.0 mg, 0.28 mmol, 95%, >20:1 d.r.).

m.p. 89-91 °C (CHCl$_3$).

^{1}H NMR (400 MHz, CDCl$_3$) δ = 7.46-7.29 (m, 5H), 6.84 (d, *J* = 8.6 Hz, 1H), 6.77 (dd, *J* = 8.6, 2.6 Hz, 1H), 6.69 (d, *J* = 2.6 Hz, 1H), 4.99 (s, 2H), 4.44 (s, 1H), 2.60-2.50 (m, 1H), 2.35 (ddd, *J* = 15.8, 9.9, 6.1 Hz, 1H), 2.08-1.98 (m, 1H), 1.96-1.76 (m, 2H), 1.75-1.61 (m, 1H), 1.42 (s, 3H).

^{13}C NMR (101 MHz, CDCl$_3$) δ = 208.8, 154.4, 153.3, 137.2, 134.7, 128.7, 128.1, 127.7, 114.3, 110.5, 109.8, 92.0, 71.2, 50.6, 38.6, 34.4, 28.2, 20.9.

FTIR (neat) ν/cm^{-1} = 2927, 1723, 1482, 1380, 1271, 1176, 1027, 989, 909, 862, 805, 740, 698.

HRMS (ESI) *m/z* calcd. for $C_{20}H_{21}O_3$ [M+H]$^+$: 309.1485; found: 309.1487.

N-((5aR*,9aS*)-9a-Methyl-6-oxo-5a,6,7,8,9,9a-hexahydrodibenzo[b,d]furan-2-yl)acetamide (21)

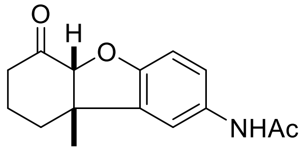

Prepared according to **General Procedure D** with **S21** (77.8 mg, 0.30 mmol), KOAc (29 mg, 0.30 mmol), Ir(Fppy)$_3$ (2.3 mg, 0.003 mmol) and acetonitrile (6 mL). Reaction time: 16 h. Purification *via* flash chromatography (EtOAc:PE, 4:1) afforded the title compound **21** as a colourless solid (69.8 mg, 0.27 mmol, 90%, >20:1 d.r.).

m.p. 178-180 °C ($CHCl_3$).

^{1}H NMR (400 MHz, $CDCl_3$) δ = 7.50 (d, *J* = 2.4 Hz, 1H), 7.37 (br s, 1H), 6.98 (dd, *J* = 8.5, 2.2 Hz, 1H), 6.81 (d, *J* = 8.2 Hz, 1H), 4.46 (s, 1H), 2.60-2.48 (m, 1H), 2.35 (ddd, *J* = 15.8, 10.0, 6.0 Hz, 1H), 2.13 (s, 3H), 2.10-2.00 (m, 1H), 1.96-1.77 (m, 2H), 1.73-1.59 (m, 1H), 1.42 (s, 3H).

^{13}C NMR (101 MHz, $CDCl_3$) δ = 208.6, 168.3, 155.7, 134.2, 132.4, 120.8, 115.1, 110.4, 92.1, 50.6, 38.6, 34.3, 28.3, 24.5, 20.9.

FTIR (neat) ν/cm^{-1} = 3253, 2933, 1715, 1647, 1618, 1557, 1479, 1428, 1378, 1314, 1249, 1222, 1178, 1123, 1079, 1029, 991, 970, 918, 875, 864, 820, 807, 762, 734.

HRMS (ESI) *m/z* calcd. for $C_{15}H_{17}O_3NNa$ [M+Na]$^+$: 282.1101; found: 282.1099.

(4aR*,9bS*)-9b-Methyl-8-(methylthio)-2,3,4a,9b-tetrahydrodibenzo[b,d]furan-4(1H)-one (22)

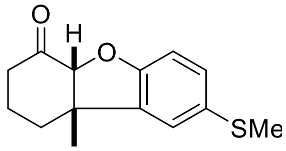

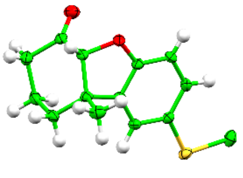

Prepared according to **General Procedure D** with **S22** (74.5 mg, 0.30 mmol), KOAc (29 mg, 0.30 mmol), Ir(Fppy)$_3$ (2.3 mg, 0.003 mmol) and acetonitrile (6 mL). Reaction time: 16 h. Purification *via* flash chromatography (PE:Et_2O, 1:1) afforded the title compound **22** as a colourless solid (65.5 mg, 0.26 mmol, 88%, >20:1 d.r.).

m.p. 69-71 °C ($CHCl_3$).

^{1}H NMR (400 MHz, $CDCl_3$) δ = 7.15 (d, *J* = 8.4 Hz, 1H), 7.03 (d, *J* = 1.9 Hz, 1H), 6.87 (*J* = 8.3 Hz, 1H), 4.47 (s, 1H), 2.60-2.50 (m, 1H), 2.45 (s, 3H), 2.36 (ddd, *J* = 15.7, 10.2, 6.3 Hz, 1H), 2.09-1.98 (m, 1H), 1.97-1.77 (m, 2H), 1.75-1.61 (m, 1H), 1.43 (s, 3H).

^{13}C NMR (101 MHz, $CDCl_3$) δ = 208.3, 157.8, 134.6, 130.1, 129.5, 123.1, 111.1, 92.0, 50.4, 38.5, 34.4, 28.3, 20.8, 18.4.

FTIR (neat) ν/cm^{-1} = 2921, 1722, 1467, 1308, 1252, 1183, 1102, 1027, 987, 929, 904, 867, 844, 813, 762, 688.

HRMS (ESI) *m/z* calcd. for $C_{14}H_{16}O_2SNa$ [M+Na]$^+$: 271.0763; found: 271.0764.

(4a*R,9b*S**)-8,9b-Dimethyl-2,3,4a,9b-tetrahydrodibenzo[*b,d*]furan-4(1*H*)-one (23)**

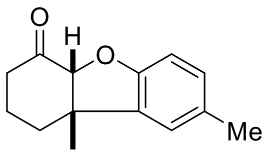

Prepared according to **General Procedure D** with **S23** (64.9 mg, 0.30 mmol), KOAc (29 mg, 0.30 mmol), Ir(Fppy)$_3$ (2.3 mg, 0.003 mmol) and acetonitrile (6 mL). Reaction time: 16 h. Purification *via* flash chromatography (PE:Et_2O, 1:1) afforded the title compound **23** as a colourless solid (59.0 mg, 0.27 mmol, 91%, >20:1 d.r.).

m.p. 87-88 °C ($CHCl_3$).

^{1}H NMR (400 MHz, $CDCl_3$) δ = 6.96 (d, *J* = 8.1 Hz, 1H), 6.84 (s, 1H), 6.81 (d, *J* = 8.1 Hz, 1H), 4.43 (s, 1H), 2.60-2.48 (m, 1H), 2.41-2.29 (m, 1H), 2.29 (s, 3H), 2.08-1.97 (m, 1H), 1.96-1.75 (m, 2H), 1.75-1.60 (m, 1H), 1.42 (s, 3H).

^{13}C NMR (101 MHz, $CDCl_3$) δ = 208.9, 156.9, 133.5, 131.1, 129.2, 122.6, 110.0, 91.9, 50.3, 38.5, 34.5, 28.2, 21.0, 20.9.

FTIR (neat) ν/cm^{-1} = 2961, 2922, 1710, 1453, 1423, 1372, 1325, 1312, 1253, 1226, 1125, 1070, 1045, 1026, 969, 934, 911, 875, 811, 782.

HRMS (ESI) *m/z* calcd. for $C_{14}H_{16}O_2Na$ [M+Na]$^+$: 239.1043; found: 239.1043.

(4a*R,9b*S**)-2,2,8,9b-Tetramethyl-2,3,4a,9b-tetrahydrodibenzo[*b,d*]furan-4(1*H*)-one (24)**

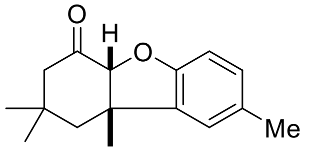

Prepared according to **General Procedure D** with **S24** (73.3 mg, 0.30 mmol), KOAc (29 mg, 0.30 mmol), Ir(Fppy)$_3$ (2.3 mg, 0.003 mmol) and acetonitrile (6 mL). Reaction time: 16 h. Purification *via* flash chromatography (PE:Et_2O, 2:1) afforded the title compound **24** as a colourless solid (69.8 mg, 0.29 mmol, 95%, >20:1 d.r.). The spectral data matched that previously reported in the literature.[19]

m.p. 73-74°C ($CHCl_3$) (Lit: 72-73 °C).[19]

^{1}H NMR (400 MHz, $CDCl_3$) δ = 6.92 (d, *J* = 7.0 Hz, 1H), 6.85-6.79 (m, 2H), 4.50 (s, 1H), 2.36 (d, *J* = 12.9 Hz, 1H), 2.27 (s, 3H), 2.26-2.16 (m, 2H), 1.92 (d, *J* = 14.9 Hz, 1H), 1.38 (s, 3H), 1.10 (s, 3H), 0.58 (s, 3H).

^{13}C NMR (101 MHz, $CDCl_3$) δ = 208.2, 155.8, 134.3, 130.8, 128.9, 122.3, 110.2, 90.8, 51.9, 49.4, 46.0, 36.1, 32.5, 32.4, 27.1, 21.1.

FTIR (neat) ν/cm^{-1} = 2958, 1726, 1485, 1370, 1315, 1254, 1181, 1124, 1074, 1027, 987, 952, 915, 809, 789, 752.

HRMS (ESI) *m/z* calcd. for $C_{16}H_{20}O_2Na$ [M+Na]$^+$: 267.1356; found: 267.1358.

(4a*R,9b*S**)-9b-Ethyl-8-methyl-2,3,4a,9b-tetrahydrodibenzo[*b,d*]furan-4(1*H*)-one (25)**

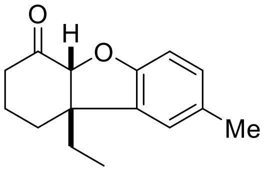

Prepared according to **General Procedure D** with **S25** (69.1 mg, 0.30 mmol), KOAc (29 mg, 0.30 mmol), Ir(Fppy)$_3$ (2.3 mg, 0.003 mmol) and acetonitrile (6 mL). Reaction time: 16 h. Purification *via* flash chromatography (PE:Et_2O, 2:1) afforded the title compound **25** as a colourless solid (64.5 mg, 0.28 mmol, 93%, >20:1 d.r.).

m.p. 73-75 °C ($CHCl_3$).

^{1}H NMR (400 MHz, $CDCl_3$) δ = 6.96 (d, *J* = 8.3 Hz, 1H), 6.83-6.77 (m, 2H), 4.52 (s, 1H), 2.58-2.49 (m, 1H), 2.38-2.29 (m, 1H), 2.29 (s, 3H), 2.05 (m, 1H), 1.97-1.55 (m, 5H), 0.85 (t, *J* = 7.4 Hz, 3H).

^{13}C NMR (101 MHz, $CDCl_3$) δ = 209.2, 157.6, 131.5, 130.8, 129.2, 123.4, 109.9, 90.1, 54.1, 38.5, 33.8, 22.1, 21.1, 20.6, 8.6.

FTIR (neat) ν/cm^{-1} = 2925, 1724, 1484, 1460, 1310, 1244, 1202, 1179, 1075, 1048, 999, 910, 876, 859, 810, 789.

HRMS (ESI) *m/z* calcd. for $C_{15}H_{19}O_2$ $[M+H]^+$: 231.1380; found: 231.1380.

(4a*R,9b*R**)-9b-Isopropyl-8-methyl-2,3,4a,9b-tetrahydrodibenzo[*b,d*]furan-4(1*H*)-one (26)**

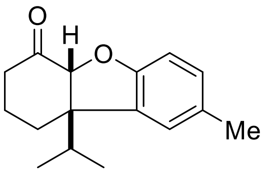

Prepared according to **General Procedure D** with **S26** (73.3 mg, 0.30 mmol), KOAc (29 mg, 0.30 mmol), Ir(Fppy)$_3$ (2.3 mg, 0.003 mmol) and acetonitrile (6 mL). Reaction time: 16 h. Purification *via* flash chromatography (PE:Et_2O, 1:2) afforded the title compound **26** as a colourless solid (59.0 mg, 0.24 mmol, 80%, 15:1 d.r.).

m.p. 58-60 °C ($CHCl_3$).

^{1}H NMR (400 MHz, $CDCl_3$) δ = 6.96 (d, *J* = 8.7 Hz, 1H), 6.81 (s, 1H), 6.78 (d, *J* = 8.1 Hz, 1H), 4.58 (s, 1H), 2.58-2.49 (m, 1H), 2.34-2.23 (m, 1H), 2.29 (s, 3H), 2.02-1.84 (m, 4H), 1.63-1.49 (m, 1H), 0.92 (d, *J* = 6.8 Hz, 3H), 0.87 (d, *J* = 6.8 Hz, 3H).

^{13}C NMR (101 MHz, $CDCl_3$) δ = 209.3, 157.9, 130.5, 130.5, 129.2, 124.3, 109.7, 88.6, 57.3, 38.2, 36.1, 30.0, 21.1, 20.4, 17.9, 17.7.

FTIR (neat) ν/cm^{-1} = 2874, 1723, 1489, 1310, 1248, 1203, 1180, 986, 910, 809, 780.

HRMS (ESI) *m/z* calcd. for $C_{16}H_{20}O_2Na$ $[M+Na]^+$: 267.1356; found: 267.1355.

Progesterone derivative 27

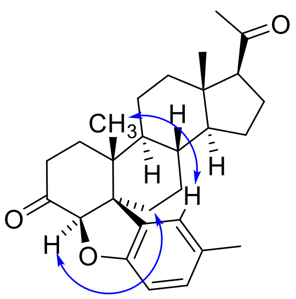

Prepared according to **General Procedure D** with **S27** (126.2 mg, 0.30 mmol), KOAc (29 mg, 0.30 mmol), Ir(Fppy)$_3$ (2.3 mg, 0.003 mmol) and acetonitrile (6 mL). Reaction time: 39 h. Purification *via* flash chromatography (PE:Et_2O, 2:1) afforded the title compound **27** as a colourless solid (106.6 mg, 0.25 mmol, 84%, 4:1 d.r.).

The product stereochemistry was assigned by nOe analysis, with blue arrows indicating through-space interaction.

<u>Analytical Data for major diastereomer:</u>

m.p. = 202-204°C ($CHCl_3$).

^{1}H NMR (500 MHz, $CDCl_3$) δ = 6.97 (d, *J* = 8.1 Hz, 1H), 6.91 (s, 1H), 6.71 (d, *J* = 8.1 Hz, 1H), 4.44 (s, 1H), 2.65 (dd, *J* = 18.5, 7.5 Hz, 1H), 2.53 (t, *J* = 9.1 Hz, 1H), 2.36-2.25 (m, 4H), 2.21-2.08 (m, 5H), 2.06-1.99 (m, 1H), 1.76-1.40 (m, 10H), 1.28-1.18 (m, 1H), 1.18-1.09 (m, 1H), 1.07 (s, 3H), 1.03-0.89 (m 2H), 0.63 (s, 3H).

^{13}C NMR (126 MHz, $CDCl_3$) δ = 209.5, 208.5, 157.6, 132.0, 129.9, 129.4, 127.0, 109.6, 91.1, 63.6, 58.1, 56.4, 45.3, 43.9, 38.7, 38.5, 35.1, 34.8, 33.3, 31.7, 29.2, 28.5, 24.5, 23.0, 21.7, 21.2, 17.4, 13.4.

FTIR (neat) ν/cm^{-1} = 2928, 1722, 1702, 1490, 1449, 1387, 1356, 1242, 1209, 984, 957, 911, 809, 731.

HRMS (ESI) *m/z* calcd. for $C_{28}H_{36}O_3Na$ $[M+Na]^+$: 443.2557; found: 443.2554.

(4a*R,9b*S**)-2,3,4a,9b-Tetrahydrodibenzo[*b*,*d*]furan-4(1*H*)-one (28)**

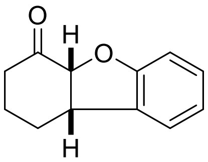

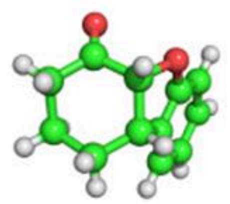

Prepared according to **General Procedure E** with **S28** (56.5 mg, 0.30 mmol), KOAc (29 mg, 0.30 mmol), 8-methylquinoline (8.2 µL, 0.06 mmol), Ir(Fppy)$_3$ (2.3 mg, 0.003 mmol) and acetonitrile (6 mL). Reaction time: 2 h. Purification *via* flash chromatography (PE:Et_2O, 1:1) afforded the title compound **28** as a colourless solid (49.5 mg, 0.26 mmol, 88%, >20:1 d.r.).

m.p. 52-53 °C ($CHCl_3$).

^{1}H NMR (400 MHz, $CDCl_3$) δ = 7.16 (dd, *J* = 7.8, 7.8 Hz, 1H), 7.11 (d, *J* = 7.4 Hz, 1H), 6.96-6.89 (m, 2H), 4.95 (d, *J* = 9.6 Hz, 1H), 4.14-4.03 (m, 1H), 2.58-2.38 (m, 2H), 2.24-2.11 (m, 1H), 2.04-1.91 (m, 2H), 1.86-1.73 (m, 1H).

^{13}C NMR (101 MHz, $CDCl_3$) δ = 208.4, 159.6, 128.8, 128.4, 123.4, 121.6, 110.4, 85.3, 44.9, 38.9, 26.6, 21.4.

FTIR (neat) ν/cm^{-1} = 2940, 1725, 1595, 1474, 1459, 1315, 1257, 1221, 1194, 1160, 1105, 1016, 1001, 961, 913, 871, 829, 754, 669.

HRMS (ESI) *m/z* calcd. for $C_{12}H_{12}O_2Na$ [M+Na]$^+$: 211.0730; found: 211.0731.

(4a*R,9b*S**)-8-Methyl-2,3,4a,9b-tetrahydrodibenzo[*b,d*]furan-4(1*H*)-one (29)**

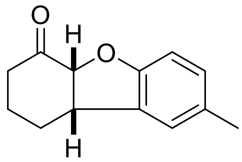

Prepared according to **General Procedure E** with **S29** (60.7 mg, 0.30 mmol), KOAc (29 mg, 0.30 mmol), 8-methylquinoline (8.2 µL, 0.06 mmol), Ir(Fppy)$_3$ (2.3 mg, 0.003 mmol) and acetonitrile (6 mL). Reaction time: 2 h. Purification *via* flash chromatography (PE:Et_2O, 1:1) afforded the title compound **29** as a colourless solid (52.6 mg, 0.26 mmol, 87%, >20:1 d.r.).

m.p. 60-61 °C ($CHCl_3$).

^{1}H NMR (400 MHz, $CDCl_3$) δ = 6.95 (d, *J* = 8.0 Hz, 1H), 6.91 (s, 1H), 6.82 (d, *J* = 8.0 Hz, 1H), 4.91 (d, (d, *J* = 9.6 Hz, 1H), 4.10-4.00 (m, 1H), 2.56-2.37 (m, 2H), 2.28 (s, 3H), 2.20-2.09 (m, 1H), 2.02-1.90 (m, 2H), 1.86-1.73 (m, 1H).

^{13}C NMR (101 MHz, $CDCl_3$) δ = 208.5, 157.6, 130.9, 129.2, 128.4, 124.0, 109.9, 85.4, 45.0, 38.9, 26.6, 21.5, 21.0.

FTIR (neat) ν/cm^{-1} = 2936, 1726, 1485, 1312, 1238, 1189, 1117, 1055, 1003, 915, 870, 811.

HRMS (ESI) *m/z* calcd. for $C_{13}H_{15}O_2$ [M+H]$^+$: 203.1067; found: 203.1069.

(4a*R,9b*S**)-8-Phenyl-2,3,4a,9b-tetrahydrodibenzo[*b,d*]furan-4(1*H*)-one (30)**

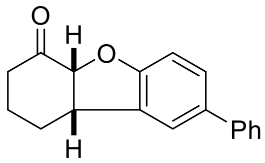

Prepared according to **General Procedure E** with **S30** (79.2 mg, 0.30 mmol), KOAc (29 mg, 0.30 mmol), 8-methylquinoline (8.2 µL, 0.06 mmol), Ir(Fppy)$_3$ (2.3 mg, 0.003 mmol) and acetonitrile (6 mL). Reaction time: 2 h. Purification *via* flash chromatography (PE:EtOAc, 2:1) afforded the title compound **30** as a pale yellow solid (63.3 mg, 0.24 mmol, 80%, >20:1 d.r.).

m.p. 93-94 °C ($CHCl_3$).

^{1}H NMR (400 MHz, $CDCl_3$) δ = 7.55-7.49 (m, 2H), 7.45-7.37 (m, 3H), 7.34-7.28 (m, 2H), 7.01 (d, *J* = 8.3 Hz, 1H), 5.01 (d, *J* = 9.6 Hz, 1H), 4.21-4.11 (m, 1H), 2.61-2.40 (m, 2H), 2.28-2.15 (m, 1H), 2.10-1.94 (m, 2H), 1.91-1.77 (m, 1H).

^{13}C NMR (101 MHz, $CDCl_3$) δ = 208.3, 159.4, 141.2, 135.3, 129.2, 128.9, 128.0, 127.0, 126.9, 122.3, 110.2, 85.7, 44.9, 38.9, 26.6, 21.5.

FTIR (neat) ν/cm^{-1} = 2940, 1727, 1612, 1475, 1301, 1237, 1197, 1156, 1117, 1055, 1003, 915, 893, 824, 763, 699.

HRMS (ESI) *m/z* calcd. for $C_{18}H_{17}O_2$ [M+H]$^+$: 265.1223; found: 265.1225.

Ethyl (5a*R,9a*S**)-6-oxo-5a,6,7,8,9,9a-hexahydrodibenzo[*b,d*]furan-2-carboxylate (31)**

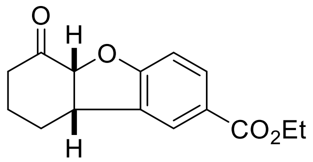

Prepared according to **General Procedure E** with **S31** (78.1 mg, 0.30 mmol), KOAc (29 mg, 0.30 mmol), 8-methylquinoline (8.2 µL, 0.06 mmol), Ir(Fppy)$_3$ (2.3 mg, 0.003 mmol) and acetonitrile (6 mL). Reaction time: 3 h. Purification *via* flash chromatography (PE:Et_2O, 2:1) afforded the title compound **31** as a colourless oil (68.2 mg, 0.26 mmol, 87%, >20:1 d.r.).

^{1}H NMR (400 MHz, $CDCl_3$) δ = 7.92 (ddd, *J* = 8.4, 1.8, 0.7 Hz, 1H), 7.81 (t, *J* = 1.3 Hz, 1H), 6.94 (d, *J* = 8.4 Hz, 1H), 5.04 (d, *J* = 9.8 Hz, 1H), 4.34 (dq, *J* = 7.1, 2.0 Hz, 2H), 4.15-4.07 (m, 1H), 2.57-2.40 (m, 2H), 2.28-2.14 (m, 1H), 2.06-1.95 (m, 2H), 1.85-1.73 (m, 1H), 1.37 (t, *J* = 7.1 Hz, 3H).

^{13}C NMR (101 MHz, $CDCl_3$) δ = 207.4, 166.4, 163.6, 131.7, 129.0, 125.4, 124.3, 110.1, 86.1, 60.9, 44.4, 38.7, 26.5, 21.4, 14.5.

FTIR (neat) ν/cm^{-1} = 2940, 1709, 1612, 1480, 1440, 1391, 1366, 1332, 1282, 1253, 1216, 1198, 1164, 1102, 1053, 1009, 915, 867, 834.

HRMS (ESI) *m/z* calcd. for $C_{15}H_{16}O_4Na$ [M+Na]$^+$: 283.0941; found: 283.0941.

(4a*R,9b*S**)-6-Bromo-2,3,4a,9b-tetrahydrodibenzo[*b,d*]furan-4(1*H*)-one (32)**

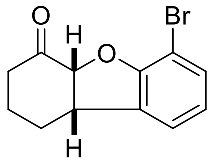

Prepared according to **General Procedure E** with **S32** (80.1 mg, 0.30 mmol), KOAc (29 mg, 0.30 mmol), 8-methylquinoline (8.2 µL, 0.06 mmol), Ir(Fppy)$_3$ (2.3 mg, 0.003 mmol) and acetonitrile (6 mL). Reaction time: 13 h. Purification *via* flash chromatography (PE:Et_2O, 2:1) afforded the title compound **32** as a colourless solid (59.0 mg, 0.22 mmol, 74%, >20:1 d.r.).

m.p. 99-100 °C ($CHCl_3$).

^{1}H NMR (400 MHz, $CDCl_3$) δ = 7.31 (dt, *J* = 8.0, 0.9 Hz, 1H), 7.04 (dt, *J* = 7.4, 1.1 Hz, 1H), 6.80 (dd, *J* = 7.7, 7.7 Hz, 1H), 5.00 (d, *J* = 9.5 Hz, 1H), 4.22-4.10 (m, 1H), 2.61-2.49 (m, 1H), 2.49-2.37 (m, 1H), 2.22-2.08 (m, 1H), 2.02-1.90 (m, 2H), 1.85-1.71 (m, 1H).

^{13}C NMR (101 MHz, $CDCl_3$) δ = 206.7, 157.1, 132.0, 130.1, 123.0, 122.4, 103.4, 85.5, 45.8, 38.8, 26.6, 21.4.

FTIR (neat) ν/cm^{-1} = 2941, 1728, 1679, 1603, 1583, 1444, 1313, 1265, 1215, 1197, 1099, 1078, 1059, 1000, 963, 916, 882, 867, 830, 804, 769.

HRMS (ESI) *m/z* calcd. for $C_{12}H_{11}O_2BrNa$ [M+Na]$^+$: 288.9835; found: 288.9835.

(4a*R,9b*S**)-7,9-Dichloro-2,3,4a,9b-tetrahydrodibenzo[*b*,*d*]furan-4(1*H*)-one (33)**

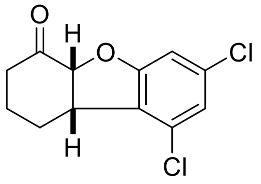

Prepared according to **General Procedure E** with **S33** (77.1 mg, 0.30 mmol), KOAc (29 mg, 0.30 mmol), 8-methylquinoline (8.2 µL, 0.06 mmol), Ir(Fppy)$_3$ (2.3 mg, 0.003 mmol) and acetonitrile (6 mL). Reaction time: 7 h. Purification *via* flash chromatography (PE:Et_2O, 2:1) afforded the title compound **33** as a colourless solid (43.5 mg, 0.17 mmol, 56%, >20:1 d.r.).

m.p. 87-88 °C ($CHCl_3$).

^{1}H NMR (400 MHz, $CDCl_3$) δ = 6.90 (d, *J* = 1.6 Hz, 1H), 6.80 (d, *J* = 1.6 Hz, 1H), 4.95 (d, *J* = 9.6 Hz, 1H), 4.10-4.01 (m, 1H), 2.65-2.46 (m, 2H), 2.30-2.18 (m, 1H), 1.99-1.89 (m, 2H), 1.83-1.71 (m, 1H).

^{13}C NMR (101 MHz, $CDCl_3$) δ = 206.7, 160.7, 134.9, 130.8, 126.3, 122.1, 109.8, 85.7, 44.7, 37.4, 25.9, 20.7.

FTIR (neat) ν/cm^{-1} = 2949, 1727, 1687, 1605, 1581, 1460, 1407, 1310, 1233, 1182, 1144, 1108, 1073, 1055, 1011, 956, 910, 885, 836.

HRMS (ESI) *m/z* calcd. for $C_{12}H_{10}O_2Cl_2Na$ $[M+Na]^+$: 278.9950; found: 278.9951.

(4a*R,9b*S**)-7,9-Dimethoxy-2,3,4a,9b-tetrahydrodibenzo[*b*,*d*]furan-4(1*H*)-one (34)**

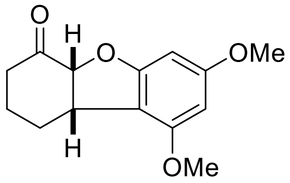

Prepared according to **General Procedure E** with **S34** (74.5 mg, 0.30 mmol), KOAc (29 mg, 0.30 mmol), 8-methylquinoline (8.2 µL, 0.06 mmol), Ir(Fppy)$_3$ (2.3 mg, 0.003 mmol) and acetonitrile (6 mL). Reaction time: 2 h. Purification *via* flash chromatography (PE:Et_2O, 1:1) afforded the title compound **34** as a colourless solid (63.3 mg, 0.26 mmol, 85%, >20:1 d.r.). The spectral data matched that previously reported in the literature.[22]

m.p. 80-81 °C ($CHCl_3$) (Lit: 91-92 °C).[22]

^{1}H NMR (400 MHz, $CDCl_3$) δ = 6.16 (d, *J* = 2.0 Hz, 1H), 6.02 (d, *J* = 2.0 Hz, 1H), 4.90 (d, *J* = 9.5 Hz, 1H), 4.09-4.00 (m, 1H), 3.77 (s, 3H), 3.76 (s, 3H), 2.59-2.37 (m, 2H), 2.18-2.02 (m, 1H), 1.97-1.78 (m, 3H).

^{13}C NMR (101 MHz, $CDCl_3$) δ = 208.7, 162.1, 161.5, 157.1, 107.6, 92.2, 88.8, 86.0, 55.7, 55.4, 43.7, 38.1, 26.4, 21.1.

FTIR (neat) ν/cm^{-1} = 2942, 1724, 1623, 1601, 1501, 1455, 1440, 1426, 1331, 1217, 1200, 1142, 1099, 1061, 1046, 1011, 965, 933, 912, 812.

HRMS (ESI) *m/z* calcd. for $C_{14}H_{17}O_4$ $[M+H]^+$: 249.1121; found: 249.1122.

(3a*R,8b*S**)-8b-Methyl-1,2,3a,8b-tetrahydro-3*H*-cyclopenta[*b*]benzofuran-3-one (35)**

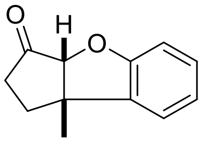

Prepared according to **General Procedure D** with **1b** (56.5 mg, 0.30 mmol), NaOAc (24.6 mg, 0.30 mmol), Ir(Fppy)$_3$ (2.3 mg, 0.003 mmol) and ethyl acetate (6 mL). Reaction time: 40 h. Purification *via* flash chromatography (PE:Et_2O, 3:1→2:1) afforded the title compound **35** as a colourless oil (49.0 mg, 0.26 mmol, 87%, >20:1 d.r.).

^{1}H NMR (400 MHz, $CDCl_3$) δ = 7.23–7.10 (m, 2H), 6.96 (td, *J* = 7.4, 1.0 Hz, 1H), 6.84 (dt, *J* = 7.9, 0.8 Hz, 1H), 4.35 (s, 1H), 2.52–2.30 (m, 2H), 2.19–2.02 (m, 2H), 1.53 (s, 3H).

^{13}C NMR (101 MHz, $CDCl_3$) δ = 214.8, 158.7, 132.9, 129.0, 123.0, 122.0, 110.3, 90.1, 49.9, 37.1, 33.0, 26.8.

FTIR (neat) ν/cm^{-1} = 2959, 2869, 1750, 1595, 1475, 1460, 1243, 1182, 1016, 982, 827,750, 642.

HRMS (ESI) *m/z* calcd. for $C_{12}H_{12}O_2Na$ [M+Na]$^+$: 211.0730; found: 211.0731.

(3a*R,8b*S**)-5-Chloro-8b-methyl-1,2,3a,8b-tetrahydro-3*H*-cyclopenta[*b*]benzofuran-3-one (36)**

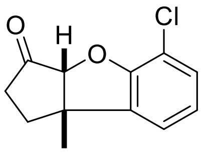

Prepared according to **General Procedure D** with **S36** (66.8 mg, 0.30 mmol), NaOAc (24.6 mg, 0.30 mmol), Ir(Fppy)$_3$ (2.3 mg, 0.003 mmol) and ethyl acetate (6 mL). Reaction time: 40 h. Purification *via* flash chromatography (PE:Et_2O, 3:1→2:1) afforded the title compound **36** as a colourless solid (24.3 mg, 0.109 mmol, 36%, >20:1 d.r.) and starting material (38.4 mg, 0.17 mmol, 57% recovery, 86% BRSM).

m.p. = 99–101 °C ($CHCl_3$).

^{1}H NMR (400 MHz, $CDCl_3$) δ = 7.18 (dd, *J* = 7.9, 1.2 Hz, 1H), 7.08 (dd, *J* = 7.5, 1.2 Hz, 1H), 6.91 (t, *J* = 7.7 Hz, 1H), 4.45 (s, 1H), 2.56–2.28 (m, 2H), 2.28–2.02 (m, 2H), 1.54 (s, 3H).

^{13}C NMR (101 MHz, $CDCl_3$) δ = 213.0, 154.7, 134.8, 129.5, 123.1, 121.3, 115.7, 90.4, 50.9, 37.1, 33.2, 26.7.

FTIR (neat) ν/cm^{-1} = 2961, 1753, 1605, 1586, 1446, 1403, 1326, 1211, 1117, 1052, 1014, 979, 829, 779, 736, 646.

HRMS (ESI) *m/z* calcd. for $C_{12}H_{12}O_2Cl$ [M+H]$^+$: 223.0520; found: 223.0524.

(3a*R,8b*S**)-5-Bromo-8b-methyl-1,2,3a,8b-tetrahydro-3*H*-cyclopenta[*b*]benzofuran-3-one (37)**

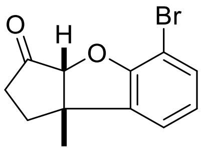

Prepared according to **General Procedure D** with **S37** (80.1 mg, 0.30 mmol), NaOAc (24.6 mg, 0.30 mmol), Ir(Fppy)$_3$ (2.3 mg, 0.003 mmol) and ethyl acetate (6 mL). Reaction time: 40 h. Purification *via* flash column chromatography (PE:Et$_2$O, 3:1→2:1) afforded the title compound **37** as a colourless solid (52.0mg, 0.195 mmol, 65% (77% BRSM), >20:1 d.r.) and starting material (12.6 mg, 0.047 mmol, 16% recovery).

m.p. = 88–90 °C (CHCl$_3$).

^{1}H NMR (400 MHz, CDCl$_3$) δ = 7.33 (dd, *J* = 7.9, 1.2 Hz, 1H), 7.12 (dd, *J* = 7.5, 1.2 Hz, 1H), 6.84 (t, *J* = 7.7 Hz, 1H), 4.44 (s, 1H), 2.53–2.32 (m, 2H), 2.23–2.03 (m, 2H), 1.54 (s, 3H).

^{13}C NMR (101 MHz, CDCl$_3$) δ = 213.0, 156.1, 134.5, 132.3, 123.5, 122.0, 103.2, 90.1, 51.1, 37.1, 33.3, 26.8.

FTIR (neat) ν/cm^{-1} = 2948, 1750, 1443, 1406, 1324, 1263, 1236, 1211, 1175, 1093, 1051, 1014, 986, 971, 828, 804, 778, 736, 642.

HRMS (ESI) *m/z* calcd. for $C_{12}H_{11}O_2BrNa$ [M+Na]$^+$: 288.9835; found: 288.9835.

(3a*R,8b*S**)-5-Iodo-8b-methyl-1,2,3a,8b-tetrahydro-3*H*-cyclopenta[*b*]benzofuran-3-one (348)**

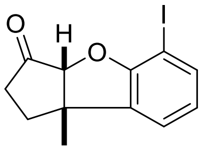

Prepared according to **General Procedure D** with **S38** (94.2 mg, 0.30 mmol), NaOAc (24.6 mg, 0.30 mmol), Ir(Fppy)$_3$ (2.3 mg, 0.003 mmol) and ethyl acetate (6 mL). Reaction time: 40 h. Purification *via* flash chromatography (PE:Et$_2$O, 3:1→2:1) afforded the title compound **38** as a colourless oil (42.2 mg, 0.13 mmol, 45% (63% BRSM), >20:1 d.r.) and starting material (28.0 mg, 0.089 mmol, 30% recovery).

^{1}H NMR (400 MHz, CDCl$_3$) δ = 7.55 (dd, *J* = 7.8, 1.2 Hz, 1H), 7.17 (dd, *J* = 7.4, 1.2 Hz, 1H), 6.75 (t, *J* = 7.6 Hz, 1H), 4.45 (s, 1H), 2.56–2.33 (m, 2H), 2.24–2.05 (m, 2H), 1.56 (s, 3H).

^{13}C NMR (101 MHz, CDCl$_3$) δ = 213.1, 159.2, 137.9, 133.2, 123.9, 122.9, 89.5, 74.2, 51.4, 37.1, 33.4, 26.8.

FTIR (neat) ν/cm^{-1} = 2959, 1751, 1596, 1433, 1324, 1209, 1175, 1091, 1050, 1014, 980, 828, 775, 738, 642.

HRMS (ESI) *m/z* calcd. for $C_{12}H_{11}O_2INa$ [M+Na]$^+$: 336.9696; found: 336.9695.

(3a*R,8b*S**)-8-(Dimethylamino)-8b-methyl-1,2,3a,8b-tetrahydro-3*H*-cyclopenta[*b*]benzofuran-3-one (39b) and (3a*R**,8b*S**)-6-(dimethylamino)-8b-methyl-1,2,3a,8b-tetrahydro-3*H*-cyclopenta[*b*]benzofuran-3-one (39b)**

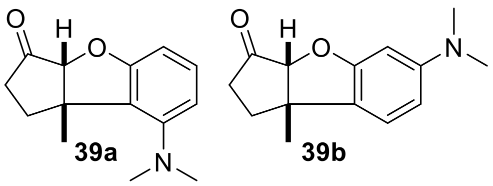

Prepared according to **General Procedure D** with **S39** (69.4 mg, 0.30 mmol), NaOAc (24.6 mg, 0.30 mmol), Ir(Fppy)$_3$ (2.3 mg, 0.003 mmol) and ethyl acetate (6 mL). Reaction time: 40 h. Purification *via* flash chromatography (PE:Et_2O, 4:1→1:1) afforded the title compound **39a** as a colourless oil (17.9 mg, 0.077 mmol, 26%) along with the title compound **39b** as a light pink solid (28.5 mg, 0.12 mmol, 41% (76% BRSM), >20:1 d.r.) and starting material (8.6 mg, 0.037 mmol, 12% recovery).

Analytical Data for **39a**:

^{1}H NMR (400 MHz, $CDCl_3$) δ = 7.14 (t, *J* = 8.0 Hz, 1H), 6.84 (dd, *J* = 8.0, 0.9 Hz, 1H), 6.64 (dd, *J* = 8.0, 0.9 Hz, 1H), 4.27 (s, 1H), 2.93–2.78 (m, 1H), 2.67 (s, 6H), 2.53–2.38 (m, 1H), 2.27–1.88 (m, 2H), 1.62 (s, 3H).

^{13}C NMR (101 MHz, $CDCl_3$) δ = 215.8, 159.9, 153.4, 129.9, 127.3, 115.7, 107.2, 90.8, 50.6, 46.6, 37.3, 31.7, 25.7.

FTIR (neat) ν/cm^{-1} = 2975, 2937, 2861, 2824, 2783, 1753, 1589, 1484, 1438, 1329, 1257, 1178, 1045, 1012, 987, 800, 744, 636.

HRMS (ESI) *m/z* calcd. for $C_{14}H_{18}O_2N$ [M+H]$^+$: 232.1332; found: 232.1334.

Analytical Data for **39b**:

m.p. = 108–110 °C ($CHCl_3$).

^{1}H NMR (400 MHz, $CDCl_3$) δ = 7.01 (d, *J* = 8.3 Hz, 1H), 6.33 (dd, *J* = 8.3, 2.3 Hz, 1H), 6.23 (d, *J* = 2.3 Hz, 1H), 4.31 (s, 1H), 2.91 (s, 6H), 2.46–2.32 (m, 2H), 2.22– 1.98 (m, 2H), 1.49 (s, 3H).

^{13}C NMR (101 MHz, $CDCl_3$) δ = 215.8, 160.3, 152.1, 122.9, 120.2, 106.5, 94.6, 90.7, 49.5, 40.9, 37.0, 32.7, 27.0.

FTIR (neat) ν/cm^{-1} = 2955, 2918, 2869, 2803, 1751, 1624, 1579, 1510, 1445, 1357, 1241, 1138, 1076, 987, 880, 816, 790, 729, 642.

HRMS (ESI) *m/z* calcd. for $C_{14}H_{18}O_2N$ [M+H]$^+$: 232.1332; found: 232.1334.

(3a*R,8b*S**)-7-Methoxy-8b-methyl-1,2,3a,8b-tetrahydro-3*H*-cyclopenta[b]benzofuran-3-one (40)**

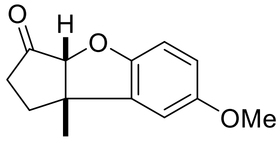

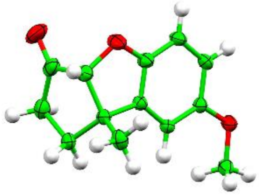

Prepared according to **General Procedure D** with **S40** (65.5 mg, 0.30 mmol), NaOAc (24.6 mg, 0.30 mmol), Ir(Fppy)$_3$ (2.3 mg, 0.003 mmol) and ethyl acetate (6 mL). Reaction time: 40 h. Purification *via* flash chromatography (PE:Et$_2$O, 3:1→2:1) afforded the title compound **40** as a colourless solid (54.4 mg, 0.25 mmol, 83%, >20:1 d.r.).

m.p. = 85–87 °C ($CHCl_3$).

^{1}H NMR (400 MHz, $CDCl_3$) δ = 6.78–6.66 (m, 3H), 4.32 (s, 1H), 3.77 (s, 3H), 2.51–2.33 (m, 2H), 2.22–2.02 (m, 2H), 1.52 (s, 3H).

^{13}C NMR (101 MHz, $CDCl_3$) δ = 215.3, 155.3, 152.8, 133.8, 113.9, 110.4, 108.9, 90.4, 56.1, 50.4, 37.0, 32.8, 26.7.

FTIR (neat) ν/cm^{-1} = 2962, 1753, 1477, 1426, 1292, 1214, 1181, 1152, 1124, 1030, 987, 862, 816, 790, 731, 659, 638.

HRMS (ESI) *m/z* calcd. for $C_{13}H_{14}O_3Na$ [M+Na]$^+$: 241.0835; found: 241.0837.

(3a*R,8b*S**)-7,8b-Dimethyl-1,2,3a,8b-tetrahydro-3*H*-cyclopenta[*b*]benzofuran-3-one (41)**

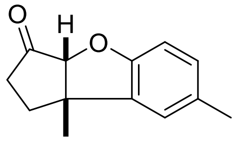

Prepared according to **General Procedure D** with **S41** (60.7 mg, 0.30 mmol), NaOAc (24.6 mg, 0.30 mmol), Ir(Fppy)$_3$ (2.3 mg, 0.003 mmol) and ethyl acetate (6 mL). Reaction time: 40 h. Purification *via* flash chromatography (PE:Et$_2$O, 3:1→2:1) afforded the title compound **41** as a colourless oil (51.9 mg, 0.26 mmol, 86%, >20:1 d.r.).

^{1}H NMR (400 MHz, $CDCl_3$) δ = 6.97 (s, 1H), 6.96 (d, *J* = 8.0 Hz, 1H), 6.73 (d, *J* = 7.9 Hz, 1H), 4.32 (s, 1H), 2.50–2.33 (m, 2H), 2.30 (s, 3H), 2.19–2.01 (m, 2H), 1.52 (s, 3H).

^{13}C NMR (101 MHz, $CDCl_3$) δ = 215.2, 156.7, 132.8, 131.4, 129.5, 123.4, 109.9, 90.2, 50.0, 37.1, 32.0, 26.9, 21.0.

FTIR (neat) ν/cm^{-1} = 2959, 2865, 1752, 1609, 1483, 1451, 1402, 1253, 1186, 1014, 985, 886, 809, 785, 737, 636.

HRMS (ESI) *m/z* calcd. for $C_{13}H_{14}O_2Na$ [M+Na]$^+$: 225.0886; found: 225.0888.

(3a*R,8b*S**)-8b-Methyl-3-oxo-2,3,3a,8b-tetrahydro-1*H*-cyclopenta[*b*]benzofuran-7-carbaldehyde (42)**

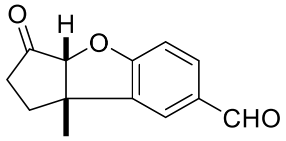

Prepared according to **General Procedure D** with **S42** (64.8 mg, 0.30 mmol), NaOAc (24.6 mg, 0.30 mmol), Ir(Fppy)$_3$ (2.3 mg, 0.003 mmol) and ethyl acetate (6 mL). Reaction time: 40 h. Purification *via* flash chromatography (PE:Et$_2$O, 1:1→0:1) afforded the title compound **42** as a yellow oil (15.0 mg, 0.069 mmol, 23% (51% BRSM), >20:1 d.r.) and starting material (35.4 mg, 0.16 mmol, 55% recovery).

^{1}H NMR (400 MHz, CDCl$_3$) δ = 9.87 (s, 1H), 7.81 (d, *J* = 1.4 Hz, 1H), 7.72 (dd, *J* = 8.3, 1.7 Hz, 1H), 6.96 (d, *J* = 8.3 Hz, 1H), 4.50 (s, 1H), 2.58–2.29 (m, 2H), 2.23–2.06 (m, 2H), 1.58 (s, 3H).
^{13}C NMR (101 MHz, CDCl$_3$) δ = 212.9, 190.6, 164.0, 135.0, 134.1, 131.9, 124.0, 110.7, 91.3, 49.4, 37.0, 33.2, 26.8.

FTIR (neat) ν/cm^{-1} = 2961, 1754, 1686, 1605, 1588, 1480, 1454, 1437, 1329, 1254, 1191, 1078, 977, 936, 894, 822, 729, 644.

HRMS (ESI) *m/z* calcd. for $C_{13}H_{13}O_3$ [M+H]$^+$: 217.0859; found: 217.0862.

Ethyl (3a*R,8b*S**)-8b-methyl-3-oxo-2,3,3a,8b-tetrahydro-1*H*-cyclopenta[b]benzofuran-7-carboxylate (43)**

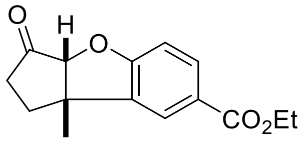

Prepared according to **General Procedure D** with **S43** (78.0 mg, 0.30 mmol), NaOAc (24.6 mg, 0.30 mmol), Ir(Fppy)$_3$ (2.3 mg, 0.003 mmol) and ethyl acetate (6 mL). Reaction time: 40 h. Purification *via* flash chromatography (PE:Et$_2$O, 3:1→2:1) afforded the title compound **43** as a clear oil (30.0mg, 0.12 mmol, 38% (90% BRSM), >20:1 d.r.) and starting material (44.7 mg, 0.17 mmol, 57% recovery).

^{1}H NMR (400 MHz, CDCl$_3$) δ = 7.98–7.87 (m, 2H), 6.86 (d, *J* = 8.4 Hz, 1H), 4.45 (s, 1H), 4.35 (q, *J* = 7.1 Hz, 2H), 2.55–2.34 (m, 2H), 2.22–2.04 (m, 2H), 1.56 (s, 3H), 1.38 (t, *J* = 7.1 Hz, 3H).

^{13}C NMR (101 MHz, CDCl$_3$) δ = 213.5, 166.3, 162.6, 133.6, 131.8, 125.1, 124.8, 110.1, 91.0, 61.0, 49.6, 37.1, 33.1, 26.8, 14.5.

FTIR (neat) ν/cm^{-1} = 2961, 1755, 1705, 1610, 1481, 1452, 1366, 1287, 1261,1240, 1174, 1100, 1014, 980, 835, 772, 744, 662.

HRMS (ESI) *m/z* calcd. for $C_{15}H_{17}O_4$ [M+H]$^+$: 261.1121; found: 261.1123.

(5a*R,8a*S**)-8a-Methyl-5a,7,8,8a-tetrahydro-6*H*-cyclopenta[4,5]furo[3,2-*b*]pyridine-6-one (44)**

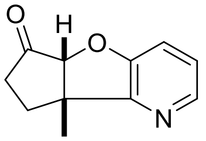

Prepared according to **General Procedure D** with **S44** (56.8 mg, 0.30 mmol), NaOAc (24.6 mg, 0.30 mmol), Ir(Fppy)$_3$ (2.3 mg, 0.003 mmol) and ethyl acetate (6 mL). Reaction time: 40 h. Purification *via* flash chromatography (PE:Et_2O, 2:1→0:1) afforded the title compound **44** as a yellow oil which solidified on standing (29.8 mg, 0.16 mmol, 52% (98% BRSM), >20:1 d.r.) and starting material (26.4 mg, 0.14 mmol, 46% recovery,).

m.p. = 67–69 °C ($CDCl_3$).

^{1}H NMR (400 MHz, $CDCl_3$) δ = 8.17 (dd, *J* = 4.5, 1.7 Hz, 1H), 7.15–7.03 (m, 2H), 4.47 (s, 1H), 2.68–2.56 (m, 1H), 2.56–2.40 (m, 1H), 2.18–2.01 (m, 2H), 1.58 (s, 3H).

^{13}C NMR (101 MHz, $CDCl_3$) δ = 213.7, 153.8, 152.3, 143.6, 123.2, 117.0, 89.8, 49.4, 37.1, 31.7, 25.1.

FTIR (neat) ν/cm^{-1} = 2963, 1752, 1602, 1575, 1425, 1266, 1169, 1098, 964, 835, 796, 734, 647.

HRMS (ESI) *m/z* calcd. for $C_{11}H_{10}O_2N$ $[M-H]^-$: 188.0717; found: 188.0714.

(3a*R,8b*S**)-8b-Ethyl-7-methyl-1,2,3a,8b-tetrahydro-3*H*-cyclopenta[*b*]benzofuran-3-one (45)**

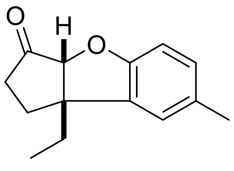

Prepared according to **General Procedure D** with **S45** (64.9 mg, 0.30 mmol), NaOAc (24.6 mg, 0.30 mmol), Ir(Fppy)$_3$ (2.3 mg, 0.003 mmol) and ethyl acetate (6 mL). Reaction time: 40 h. Purification *via* flash chromatography (PE:Et_2O, 3:1) afforded the title compound **45** as a colourless, viscous oil. (55.5 mg, 0.26 mmol, 86%, >20:1 d.r.).

^{1}H NMR (400 MHz, $CDCl_3$) δ = 7.00–6.92 (m, 2H), 6.72 (d, *J* = 8.0 Hz, 1H), 4.38 (s, 1H), 2.49–2.32 (m, 2H), 2.31 (s, 3H), 2.19–2.02 (m, 2H), 1.92–1.74 (m, 2H), 0.85 (t, *J* = 7.4 Hz, 3H).

^{13}C NMR (101 MHz, $CDCl_3$) δ = 215.5, 157.5, 131.2, 130.9, 129.6, 123.9, 109.7, 88.5, 54.4, 36.8, 32.9, 31.3, 21.1, 9.2.

FTIR (neat) ν/cm^{-1} = 2963, 1753, 1610, 1483, 1460, 1241, 1184, 991, 889, 810, 785.

HRMS (ESI) *m/z* calcd. for $C_{14}H_{17}O_2$ $[M+H]^+$: 217.1223; found: 217.1225.

(3a*R,8bR*)-8b-Benzyl-7-methyl-1,2,3a,8b-tetrahydro-3*H*-cyclopenta[*b*]benzofuran-3-one (46)**

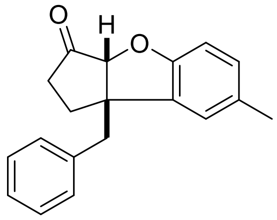

Prepared according to **General Procedure D** with **S46** (83.5 mg, 0.30 mmol), NaOAc (24.6 mg, 0.30 mmol), Ir(Fppy)$_3$ (2.3 mg, 0.003 mmol) and ethyl acetate (6 mL). Reaction time: 40 h. Purification *via* flash chromatography (PE:Et_2O, 3:1) afforded the title compound **46** as a colourless, viscous oil. (59.1 mg, 0.21 mmol, 71%, >20:1 d.r.).

^{1}H NMR (400 MHz, $CDCl_3$) δ = 7.38–7.14 (m, 3H), 7.10–7.01 (m, 2H), 6.97 (dd, *J* = 8.1, 1.8 Hz, 1H), 6.81 (d, *J* = 1.8 Hz, 1H), 6.69 (d, *J* = 8.3 Hz, 1H), 4.52 (s, 1H), 3.10 (d, *J* = 13.4 Hz, 1H), 3.01 (d, *J* = 13.4 Hz, 1H), 2.46–2.21 (m, 3H), 2.30 (s, 3H) 2.11–1.94 (m, 1H).

^{13}C NMR (101 MHz, $CDCl_3$) δ = 215.5, 157.2, 136.7, 131.1, 130.8, 130.3, 129.8, 128.3, 127.0, 124.3, 109.9, 87.5, 54.6, 45.4, 36.7, 29.7, 21.0.

FTIR (neat) ν/cm^{-1} = 2920, 1751, 1604, 1484, 1454, 1402, 1235, 1191, 996, 969, 910, 811, 784, 767, 730, 702.

HRMS (ESI) *m/z* calcd. for $C_{19}H_{18}O_2Na$ [M+Na]$^+$: 301.1199; found: 301.1200.

(3a*R,8b*R**)-8b-Cyclohexyl-7-methyl-1,2,3a,8b-tetrahydro-3*H*-cyclopenta[*b*]benzofuran-3-one (47)**

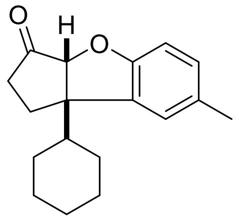

Prepared according to **General Procedure D** with **S47** (81.1 mg, 0.30 mmol), NaOAc (24.6 mg, 0.30 mmol), Ir(Fppy)$_3$ (2.3 mg, 0.003 mmol) and ethyl acetate (6 mL). Reaction time: 40 h. Purification *via* flash chromatography (PE:Et_2O, 5:1→4:1) afforded the title compound **47** as a colourless solid. (69.5 mg, 0.26 mmol, 86%, >20:1 d.r.).

m.p. = 116–118 °C ($CHCl_3$).

^{1}H NMR (400 MHz, $CDCl_3$) δ = 7.00–6.92 (m, 2H), 6.70 (d, *J* = 8.2 Hz, 1H), 4.41 (s, 1H), 2.50–2.33 (m, 2H), 2.31 (s, 3H), 2.20–1.97 (m, 2H), 1.84 1.74 (m, 3H), 1.74–1.64 (m, 1H), 1.64–1.52 (m, 1H), 1.58 (s, 1H), 1.30–1.18 (m, 3H), 1.18–0.97 (m, 1H), 0.73–0.58 (m, 1H).

^{13}C NMR (101 MHz, $CDCl_3$) δ = 216.1, 157.9, 130.8, 129.6, 129.4, 125.0, 109.6, 88.4, 57.4, 47.3, 36.9, 29.6, 28.2, 27.4, 26.6, 26.4, 26.3, 21.2.

FTIR (neat) ν/cm^{-1} = 2926, 2853, 1752, 1610, 1484, 1450, 1196, 1115, 997, 884, 810, 784.

HRMS (ESI) *m/z* calcd. for $C_{18}H_{22}O_2Na$ [M+Na]$^+$: 293.1512; found: 293.1503.

(3a*R,8b*S**)-7-Methyl-1,2,3a,8b-tetrahydro-3*H*-cyclopenta[*b*]benzofuran-3-one (48)**

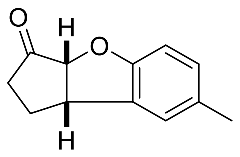

Prepared according to **General Procedure D** with **S48** (56.5 mg, 0.30 mmol), NaOAc (24.6 mg, 0.30 mmol), Ir(Fppy)$_3$ (2.3 mg, 0.003 mmol) and ethyl acetate (6 mL). Reaction time: 40 h. Purification *via* flash chromatography (PE:Et$_2$O, 3:1→2:1) afforded the title compound **48** as a colourless, viscous oil. (23.8 mg, 0.13 mmol, 42%).

^{1}H NMR (400 MHz, CDCl$_3$) δ = 7.03 (d, *J* = 1.7 Hz, 1H), 6.96 (dd, *J* = 8.2, 1.8 Hz, 1H), 6.72 (d, *J* = 8.1 Hz, 1H), 4.75 (d, *J* = 8.8, 1.4 Hz, 1H), 4.24–4.14 (m, 1H), 2.46–2.32 (m, 2H), 2.30 (s, 3H), 2.27–2.21 (m, 1H), 2.19–2.05 (m, 1H).

^{13}C NMR (101 MHz, CDCl$_3$) δ = 215.1, 157.3, 131.3, 129.5, 128.4, 124.9, 109.7, 84.1, 43.0, 35.1, 25.7, 21.0.

FTIR (neat) ν/cm^{-1} = 2922, 1750, 1612, 1485, 1459, 1402, 1282, 1246, 1196, 1048, 985, 810.

HRMS (ESI) *m/z* calcd. for $C_{12}H_{13}O_2$ [M+H]$^+$: 189.0910; found: 189.0912.

(4a*R,9b*S**)-8,9b-Dimethyl-2,3,4a,9b-tetrahydrodibenzo[b,d]thiophen-4(1*H*)-one (49)**

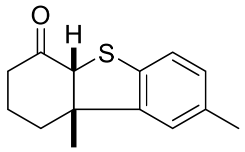

Prepared according to **General Procedure D** with **S49** (69.7 mg, 0.30 mmol), KOAc (29 mg, 0.30 mmol), Ir(Fppy)$_3$ (2.3 mg, 0.003 mmol) and acetonitrile (6 mL). Reaction time: 16 h. Purification *via* flash chromatography (PE:Et$_2$O, 4:1) afforded the title compound **49** as a colourless solid (63.5 mg, 0.27 mmol, 91%, >20:1 d.r.).

m.p. 60-62°C (CHCl$_3$) (Lit: 56-57 °C).[23]

^{1}H NMR (400 MHz, CDCl$_3$) δ = 7.09 (d, *J* = 7.9 Hz, 1H), 6.99 (d, *J* = 7.9 Hz, 1H), 6.84 (s, 1H), 3.94 (s, 1H), 2.79 (ddd, *J* = 15.5, 7.9, 7.9 Hz, 1H), 2.39-2.31 (m, 1H), 2.32 (s, 3H), 2.22-2.12 (m, 1H), 1.91-1.82 (m, 2H), 1.70-1.61 (m, 1H), 1.45 (s, 3H).

^{13}C NMR (101 MHz, CDCl$_3$) δ = 208.5, 146.3, 135.8, 135.0, 128.9, 123.6, 122.4, 65.2, 54.3, 37.6, 34.3, 25.7, 21.3, 21.3.

FTIR (neat) ν/cm^{-1} = 2929, 1709, 1454, 1414, 1314, 1268, 1229, 1184, 1125, 1104, 1043, 976, 880, 835, 803.

HRMS (ESI) *m/z* calcd. for $C_{14}H_{16}OSNa$ [M+Na]$^+$: 255.0814; found: 255.0815.

(3a*R,8b*S**)-7,8b-Dimethyl-1,2,3a,8b-tetrahydro-3*H*-benzo[*b*]cyclopenta[*d*]thiophen-3-one (50)**

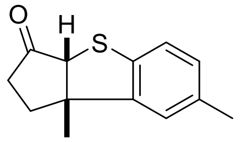

Prepared according to **General Procedure D** with **S50** (65.5 mg, 0.30 mmol), NaOAc (24.6 mg, 0.30 mmol), Ir(Fppy)$_3$ (2.3 mg, 0.003 mmol) and ethyl acetate (6 mL). Reaction time: 40 h. Purification *via* flash chromatography (PE:Et$_2$O, 4:1→3:1) afforded the title compound **50** as a pale yellow solid (57.0 mg, 0.26 mmol, 87%, >20:1 d.r.).

m.p. 83–85 °C ($CHCl_3$).

^{1}H NMR (400 MHz, $CDCl_3$) δ = 7.05–6.93 (m, 3H), 3.60 (s, 1H), 2.57–2.36 (m, 2H), 2.31 (s, 3H), 2.25–2.04 (m, 2H), 1.49 (s, 3H).

^{13}C NMR (101 MHz, $CDCl_3$) δ = 216.8, 144.9, 136.2, 135.1, 129.3, 124.1, 121.8, 62.8, 56.5, 37.7, 35.1, 26.4, 21.2.

FTIR (neat) ν/cm^{-1} = 2970, 2956, 2913, 2866, 1739, 1466, 1403, 1372, 1323, 1253, 1173, 1148, 1121, 1052, 1039, 986, 883, 816, 708, 629.

HRMS (ESI) *m/z* calcd. for $C_{13}H_{15}OS$ [M+H]$^+$: 219.0838; found: 219.0841.

(4a*S,9a*R**)-4a,9-Dimethyl-2,3,4,4a,9,9a-hexahydro-1*H*-carbazol-1-one (51)**

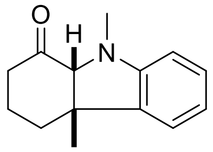

Prepared according to **General Procedure D** with **S51** (64.5 mg, 0.30 mmol), KOAc (29 mg, 0.30 mmol), Ir(Fppy)$_3$ (2.3 mg, 0.003 mmol) and acetonitrile (6 mL). Reaction time: 16 h. Purification *via* flash chromatography (PE:Et_2O, 6:1) afforded the title compound **51** as a colourless oil (49.9 mg, 0.23 mmol, 77%, >20:1 d.r.).

^{1}H NMR (400 MHz, $CDCl_3$) δ = 7.16 (ddd, *J* = 7.6, 7.6, 1.2 Hz, 1H), 7.02 (dd, *J* = 7.3, 0.9 Hz, 1H), 6.80 (ddd, *J* = 7.4, 7.4, 0.9 Hz, 1H), 6.58 (d, *J* = 7.8 Hz, 1H), 3.14 (s, 1H), 2.73-2.64 (m, 1H), 2.70 (s, 3H), 2.38-2.28 (m, 1H), 1.95-1.70 (m, 3H), 1.66-1.55 (m, 1H), 1.40 (s, 3H).

^{13}C NMR (101 MHz, $CDCl_3$) δ = 212.1, 151.5, 137.9, 128.2, 121.5, 119.3, 108.6, 81.9, 48.8, 38.2, 36.5, 35.2, 24.4, 21.3.

FTIR (neat) ν/cm^{-1} = 2956, 1711, 1604, 1482, 1451, 1307, 1258, 1123, 1021, 976, 742.

HRMS (ESI) *m/z* calcd. for $C_{14}H_{18}ON$ [M+H]$^+$: 216.1383; found: 216.1383.

(3a*R,8b*S**)-4,8b-Dimethyl-1,3a,4,8b-tetrahydrocyclopenta[*b*]indol-3(2*H*)-one (52)**

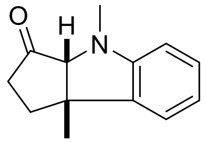

Prepared according to **General Procedure D** with **S52** (60.4 mg, 0.30 mmol), NaOAc (24.6 mg, 0.30 mmol), Ir(Fppy)$_3$ (2.3 mg, 0.003 mmol) and ethyl acetate (6 mL). Reaction time: 40 h. Purification *via* flash chromatography (PE:Et_2O, 4:1→3:1) afforded the title compound **52** as a colourless oil (50.9 mg, 0.25 mmol, 84%, >20:1 d.r.).

^{1}H NMR (400 MHz, $CDCl_3$) δ = 7.13 (t, *J* = 7.9 Hz, 1H), 7.08 (d, *J* = 7.0 Hz, 1H), 6.74 (t, *J* = 7.4 Hz, 1H), 6.45 (d, *J* = 7.8 Hz, 1H), 3.23 (s, 1H), 2.91 (s, 3H), 2.48-2.33 (m, 1H), 2.33-2.22 (m, 1H), 2.22-2.11 (m, 1H), 2.05-1.93 (m, 1H), 1.48 (s, 3H).

^{13}C NMR (101 MHz, $CDCl_3$) δ = 216.0, 150.6, 135.8, 128.4, 122.0, 118.2, 107.0, 78.8, 49.7, 38.3, 35.2, 33.5, 24.9.

FTIR (neat) ν/cm^{-1} = 3050, 2956, 2868, 1740, 1605, 1484, 1451, 1372, 1303, 1219, 1157, 1120, 1093, 1020, 985, 784, 744.

HRMS (ESI) *m/z* calcd. for $C_{13}H_{16}ON$ $[M+H]^+$: 202.1226; found: 202.1227.

(4a*S,9a*R**)-9-Allyl-4a-methyl-2,3,4,4a,9,9a-hexahydro-1*H*-carbazol-1-one (53)**

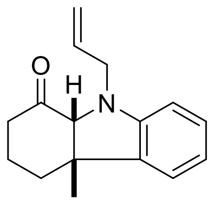

Prepared according to **General Procedure D** with **S53** (72.3 mg, 0.30 mmol), KOAc (29 mg, 0.30 mmol), Ir(Fppy)$_3$ (2.3 mg, 0.003 mmol) and acetonitrile (6 mL). Reaction time: 16 h. Purification *via* flash chromatography (PE:Et_2O, 7:1) afforded the title compound **53** as a colourless oil (37.4 mg, 0.16 mmol, 52%, >20:1 d.r.).

^{1}H NMR (400 MHz, $CDCl_3$) δ = 7.12 (ddd, *J* = 7.7, 7.7, 1.2 Hz, 1H), 7.00 (dd, *J* = 7.3, 1.0 Hz, 1H), 6.78 (ddd, *J* = 7.4, 7.4, 0.8 Hz, 1H), 6.58 (d, *J* = 7.8 Hz, 1H), 5.86-5.74 (m, 1H), 5.25 (dq, *J* = 17.2, 1.7 Hz, 1H), 5.18 (dq, *J* = 10.3, 1.5 Hz, 1H), 3.85-3.77 (m, 1H), 3.66-3.57 (m, 1H), 3.44 (s, 1H), 2.74-2.63 (m, 1H), 2.30 (ddd, *J* = 15.6, 6.2, 6.2 Hz, 1H), 1.95-1.69 (m, 3H), 1.64-1.54 (m, 1H), 1.40 (s, 3H).

^{13}C NMR (101 MHz, $CDCl_3$) δ = 212.4, 150.5, 137.5, 133.0, 128.1, 121.6, 119.0, 117.9, 108.6, 79.3, 50.6, 49.0, 38.2, 36.6, 25.2, 21.1.

FTIR (neat) ν/cm^{-1} = 2939, 2868, 1711, 1604, 1481, 1460, 1419, 1380, 1349, 1335, 1311, 1250, 1230, 1161, 1139, 1026, 991, 922, 742.

HRMS (ESI) *m/z* calcd. for $C_{16}H_{20}ON$ $[M+H]^+$: 242.1539; found: 242.1540.

(3a*R,8b*S**)-4-Benzyl-8b-methyl-1,3a,4,8b-tetrahydrocyclopenta[*b*]indol-3(2*H*)-one (54)**

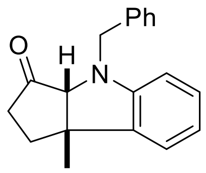

Prepared according to **General Procedure D** with **S54** (83.2 mg, 0.30 mmol), NaOAc (24.6 mg, 0.30 mmol), Ir(Fppy)$_3$ (2.3 mg, 0.003 mmol) and ethyl acetate (6 mL). Reaction time: 40 h. Purification *via* flash chromatography (PE:Et_2O, 7:1→6:1) afforded the title compound **54** as a yellow oil (73.6 mg, 0.27 mmol, 88%, >20:1 d.r.).

^{1}H NMR (400 MHz, $CDCl_3$) δ = 7.30-7.14 (m, 5H), 7.07-6.96 (m, 2H), 6.66 (td, *J* = 7.4, 0.9 Hz, 1H), 6.33 (d, *J* = 7.8 Hz, 1H), 4.52 (d, *J* = 15.9 Hz, 1H), 4.44 (d, *J* = 15.8 Hz, 1H), 3.32 (dd, *J* = 1.5, 0.8 Hz, 1H), 2.41-2.26 (m, 1H), 2.26-2.11 (m, 2H), 2.00-1.86 (m, 1H), 1.39 (s, 3H).

^{13}C NMR (101 MHz, $CDCl_3$) δ = 216.6, 149.7, 138.0, 135.3, 128.6, 128.4, 127.7, 127.2, 122.3, 118.1, 106.9, 75.3, 49.6, 49.5, 38.3, 34.8, 25.9.

FTIR (neat) ν/cm^{-1} = 3028, 2955, 1738, 1603, 1481, 1452, 1351, 1310, 1178, 1158, 1077, 1026, 913, 743, 698.

HRMS (ESI) *m/z* calcd. for $[M+H]^+$: ; found: .

(7a*R,11a*S**)-11a-Methyl-5,6,9,10,11,11a-hexahydro-4*H*-pyrido[3,2,1-jk]carbazol-8(7a*H*)-one (55)**

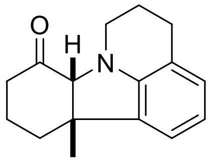

Prepared according to **General Procedure D** with **S55** (72.4 mg, 0.30 mmol), KOAc (29 mg, 0.30 mmol), $Ir(Fppy)_3$ (2.3 mg, 0.003 mmol) and acetonitrile (6 mL). Reaction time: 16 h. Purification *via* flash chromatography (PE:Et_2O, 7:1) afforded the title compound **55** as a colourless solid (55.8 mg, 0.23 mmol, 77%, >20:1 d.r.).

m.p. 85–87 °C ($CHCl_3$).

^{1}H NMR (400 MHz, $CDCl_3$) δ = 6.90 (d, *J* = 7.6 Hz, 1H), 6.86 (d, *J* = 7.3 Hz, 1H), 6.73 (d, *J* = 7.5, 7.5 Hz, 1H), 3.14 (s, 1H), 3.14-3.06 (m, 1H), 2.78-2.64 (m, 4H), 2.33 (dt, *J* = 15.0, 4.8 Hz, 1H), 2.16-1.85 (m, 4H), 1.84-1.71 (m, 1H), 1.68-1.59 (m, 1H), 1.39 (s, 3H).

^{13}C NMR (101 MHz, $CDCl_3$) δ = 211.9, 147.8, 136.7, 127.1, 120.8, 119.7, 119.0, 81.4, 49.5, 46.1, 38.3, 36.0, 23.9, 23.4, 21.8.

FTIR (neat) ν/cm^{-1} = 2934, 2816, 1710, 1597, 1480, 1458, 1340, 1312, 1263, 1237, 1182, 1139, 1055, 751.

HRMS (ESI) *m/z* calcd. for $C_{16}H_{20}ON$ $[M+H]^+$: 242.1539; found: 242.1538.

3,3'-((1*R,2*R**,3*S**,4*S**)-3,4-Dimethylcyclobutane-1,2-diyl)bis(2-(*p*-tolyloxy)cyclohex-2-en-1-one) (57)**

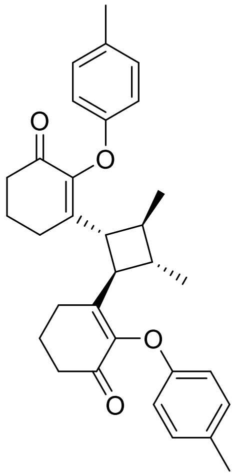

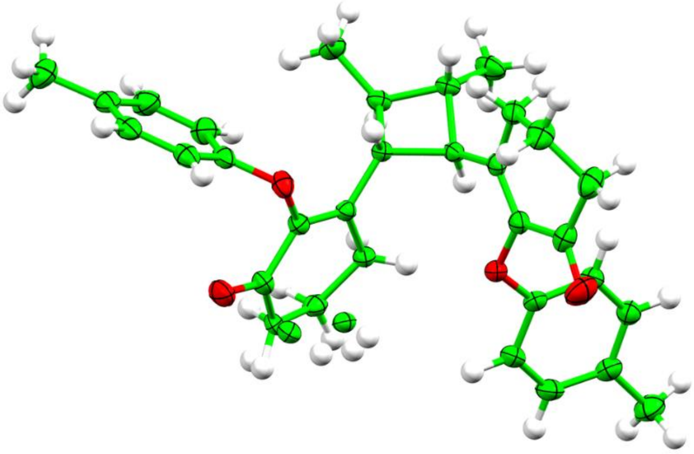

Prepared according to **General Procedure D** with **56** (72.7 mg, 0.30 mmol), KOAc (29 mg, 0.30 mmol), $Ir(Fppy)_3$ (2.3 mg, 0.003 mmol) and acetonitrile (6 mL). Reaction time: 21 h. Purification *via* flash chromatography (PE:Et_2O, 1:1) afforded the title compound **57** as a colourless solid (51.7 mg, 0.11 mmol, 71%, >20:1 d.r.).

m.p. = 120-122°C ($CHCl_3$).

^{1}H NMR (400 MHz, $CDCl_3$) δ = 7.02 (d, *J* = 8.2 Hz, 4H), 6.69 (d, *J* = 8.5 Hz, 4H), 3.12-3.01 (m, 2H), 2.45-2.17 (m, 14H), 1.95-1.65 (m, 6H), 0.97 (d, *J* = 6.0 Hz, 6H).

^{13}C NMR (101 MHz, $CDCl_3$) δ = 193.2, 156.2, 149.5, 144.3, 131.1, 130.0, 114.5, 44.2, 39.4, 38.2, 25.9, 21.9, 20.6, 19.0.

FTIR (neat) ν/cm^{-1} = 2941, 1681, 1606, 1504, 1453, 1352, 1330, 1300, 1213, 1168, 1140, 1111, 1046, 962, 903, 868, 811, 747.

HRMS (ESI) *m/z* calcd. for $C_{32}H_{37}O_4$ $[M+H]^+$: 485.2686; found: 485.2684.

VI. Derivatization

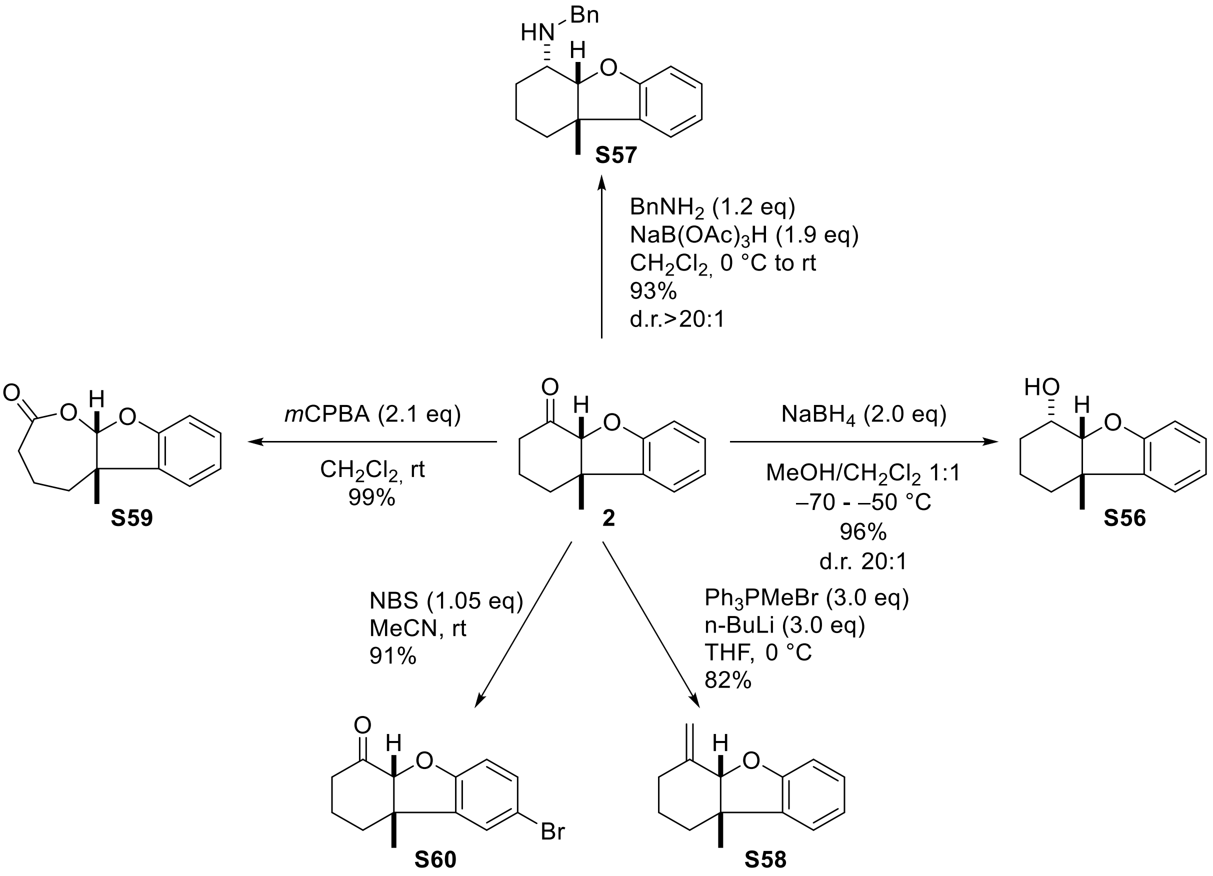

(4*S**,4a*R**,9b*S**)-9b-Methyl-1,2,3,4,4a,9b-hexahydrodibenzo[*b*,*d*]furan-4-ol (S56)

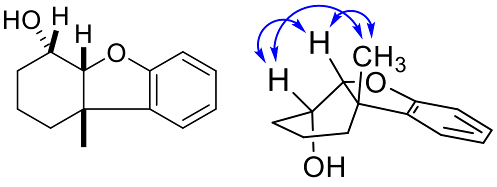

Ketone **2** (60.7 mg, 0.30 mmol) was dissolved in $MeOH/CH_2Cl_2$ (1:1, 3 mL) and cooled to −70 °C. $NaBH_4$ (22.7 mg, 0.60 mmol) was added in one portion and the solution was warmed to −50 °C over 30 min, then stirred for an additional 30 min. The reaction mixture was warmed to −10 °C slowly and was then quenched by addition of aq. NH_4Cl (10 mL). The aqueous layer was extracted with EtOAc (3 × 15 mL) and the combined organic extracts were dried over Na_2SO_4, filtered, and concentrated under reduced pressure. Purification of the residue *via* flash chromatography (PE:EtOAc, 2:1) afforded the alcohol **S56** (59.0 mg, 0.29 mmol, 96%, 20:1 d.r.) as a colourless solid.

The product stereochemistry was assigned by nOe analysis, with blue arrows indicating through-space interaction.

m.p. 69-71°C ($CHCl_3$).

^{1}H NMR (400 MHz, $CDCl_3$) δ = 7.14 (ddd, *J* = 7.7, 7.7, 1.4 Hz, 1H), 7.06 (dd, *J* = 7.4, 1.2 Hz, 1H), 6.90 (ddd, *J* = 7.4, 7.4, 0.8 Hz, 1H), 6.90 (d, *J* = 8.0 Hz, 1H), 4.34 (d, *J* = 3.5 Hz,

1H), 3.97-3.87 (m, 1H), 2.20 (br s, 1H), 1.96-1.86 (m, 1H), 1.65-1.50 (m, 3H), 1.50-1.24 (m, 2H), 1.39 (s, 3H).

^{13}C NMR (101 MHz, $CDCl_3$) δ = 157.9, 138.6, 128.0, 121.7, 121.1, 110.3, 90.6, 68.8, 45.1, 35.6, 30.0, 21.8, 20.0.

FTIR (neat) ν/cm^{-1} = 3357, 2932, 2862, 1595, 1473, 1455, 1444, 1378, 1327, 1315, 1281, 1237, 1206, 1174, 1147, 1116, 1101, 1081, 1066, 1014, 985, 888, 841, 741, 722.

HRMS (ESI) *m/z* calcd. for $C_{13}H_{16}O_2Na$ [M+Na]$^+$: 227.1043; found: 227.1044.

(4*S,4a*R**,9b*S**)-*N*-Benzyl-9b-methyl-1,2,3,4,4a,9b-hexahydrodibenzo[*b,d*]furan-4-amine (S57)**

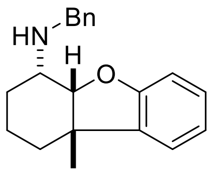

$NaHB(OAc)_3$ (80.5 mg, 0.38 mmol) was added in one portion to a solution of ketone **2** (40.5 mg, 0.20 mmol) and benzylamine (26 µL, 0.24 mmol) in CH_2Cl_2 (2.0 mL) at 0 °C. The reaction mixture was stirred at 0 °C for 3 h and then warmed to room temperature slowly over 1 h. After stirring for 10 h at room temperature the reaction was quenched by addition of aq. $NaHCO_3$ (20 mL) and extracted with CH_2Cl_2 (3 × 10 mL). The combined organic extracts were dried over Na_2SO_4, filtered and concentrated under reduced pressure. Purification of the residue *via* flash chromatography (PE:EtOAc, 2:1) afforded **S57** (54.9 mg, 0.19 mmol, 93%, >20:1 d.r.) as a colourless oil.

The product stereochemistry was assigned by nOe analysis in a similar manner to compound **S49**.

^{1}H NMR (500 MHz, $CDCl_3$) δ = 7.43-7.37 (m, 2H), 7.37-7-32 (m, 2H), 7.29-7.24 (m, 1H), 7.13 (ddd, *J* = 7.7, 7.7, 1.3 Hz, 1H), 7.06 (dd, *J* = 7.2, 1.2 Hz, 1H), 6.89 (ddd, *J* = 7.4, 7.4, 0.8 Hz, 1H), 6.85 (d, *J* = 8.0 Hz, 1H), 4.39 (d, *J* = 3.2 Hz, 1H), 3.96 (d, *J* = 3.0 Hz, 2H), 2.90-2.83 (m, 1H), 1.98-1.91 (m, 1H), 1.77 (br s, 1H), 1.61-1.50 (m, 2H), 1.46-1.28 (m, 3H), 1.36 (s, 3H).

^{13}C NMR (101 MHz, $CDCl_3$) δ = 158.3, 140.6, 139.0, 128.5, 128.3, 127.9, 127.0, 121.7, 120.7, 110.4, 89.3, 53.8, 50.7, 44.3, 36.3, 27.1, 21.5, 20.6.

FTIR (neat) ν/cm^{-1} = 2930, 1596, 1495, 1455, 1211, 1013, 957, 889, 745, 698.

HRMS (ESI) *m/z* calcd. for $C_{20}H_{24}O_1N$ [M+H]$^+$: 294.1852; found: 294.1852.

(4a*S,9b*S**)-9b-Methyl-4-methylene-1,2,3,4,4a,9b-hexahydrodibenzo[*b,d*]furan (S58)**

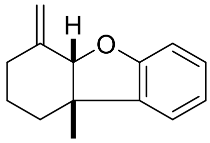

To a solution of PPh_3MeBr (322 mg, 0.90 mmol) in THF (1.5 mL) was added *n*-BuLi (2.5 M in *n*-hexane, 0.36 mL, 0.90 mmol) at 0 °C. After stirring for 30 min at 0 °C a solution of ketone **2** (60.7 mg, 0.30 mmol) in THF (1.5 mL) was added to the first solution. The reaction mixture was stirred for 40 min at 0 °C and was then quenched by addition of aq. NH_4Cl (10 mL). The mixture was extracted with Et_2O (3 × 10 mL). The combined organic extracts were dried over Na_2SO_4, filtered, and concentrated under reduced pressure. Purification of the residue *via*

flash chromatography (PE:Et_2O, 40:1) afforded **S58** (49.4 mg, 0.25 mmol, 82%, >20:1 d.r.) as a colourless solid.

m.p. 54-55°C ($CHCl_3$).

^{1}H NMR (400 MHz, $CDCl_3$) δ = 7.14 (ddd, *J* = 7.7, 7.7, 1.3 Hz, 1H), 7.08 (dd, *J* = 7.3, 1.1 Hz, 1H), 6.90 (ddd, *J* = 7.4, 7.4, 0.9 Hz, 1H), 6.83 (d, *J* = 8.0 Hz, 1H), 5.18 (s, 1H), 5.12 (s, 1H), 4.49 (s, 1H), 2.42 (m, 2H), 1.59-1.41 (m, 4H), 1.33 (s, 3H).

^{13}C NMR (101 MHz, $CDCl_3$) δ = 158.7, 143.4, 137.7, 128.0, 122.2, 120.8, 116.4, 110.1, 93.0, 45.8, 36.1, 29.8, 22.8, 21.4.

FTIR (neat) ν/cm^{-1} = 2981, 2933, 1595, 1474, 1456, 1379, 1379, 1239, 1207, 1151, 114, 1014, 999, 954, 911, 883, 844, 745.

HRMS (ESI) *m/z* calcd. for $C_{14}H_{17}O_1Na$ $[M+H]^+$: 201.1274; found: 201.1276.

(5a*S,10a*S**)-5a-Methyl-4,5,5a,10a-tetrahydrooxepino[2,3-*b*]benzofuran-2(3*H*)-one (S59)**

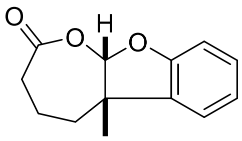

*m*CPBA (~75%, 94.0 mg, 0.42 mmol) was added to a solution of ketone **2** (60.7 mg, 0.30 mmol) in CH_2Cl_2 (3.0 mL). After stirring for 20 h at room temperature another portion of *m*CPBA (47 mg, 0.21 mmol) was added and the reaction mixture was stirred for additional 48 h at room temperature. The solution was diluted with CH_2Cl_2 (30 mL) and washed with aq. Na_2SO_3 (10 mL), aq. $NaHCO_3$ (10 mL) and brine (10 mL). The organic layer was dried over Na_2SO_4, filtered and concentrated under reduced pressure. Purification of the residue *via* flash chromatography (PE:Et_2O, 1:2) afforded **S59** (64.8 mg, 0.30 mmol, 99%, >20:1 d.r.) as a colourless gum.

The product stereochemistry was assigned by nOe analysis in a similar manner to compound **S49**.

^{1}H NMR (500 MHz, $CDCl_3$) δ = 7.21 (ddd, *J* = 7.8, 7.7, 1.2 Hz, 1H), 7.08 (dd, *J* = 7.5, 1.0 Hz, 1H), 7.00 (ddd, *J* = 7.4, 7.4, 0.9 Hz, 1H), 6.93 (d, *J* = 8.0 Hz, 1H), 5.92 (s, 1H), 2.67-2.59 (m, 1H), 2.50-2.40 (m, 1H), 2.16-2.06 (m, 1H), 1.98-1.83 (m, 3H), 1.35 (s, 3H).

^{13}C NMR (126 MHz, $CDCl_3$) δ = 170.4, 156.5, 133.3, 128.9, 122.7, 122.6, 110.5, 110.1, 47.9, 32.7, 30.9, 24.6, 17.8.

FTIR (neat) ν/cm^{-1} = 2962, 1746, 1600, 1478, 1459, 1382, 1345, 1303, 1276, 1236, 1194, 1131, 1102, 1083, 1055, 1012, 995, 968, 924, 909, 852, 838, 747.

HRMS (ESI) *m/z* calcd. for $C_{13}H_{14}O_3Na$ $[M+Na]^+$: 241.0835; found: 241.0836.

(4a*R,9b*S**)-8-Bromo-9b-methyl-2,3,4a,9b-tetrahydrodibenzo[*b*,*d*]furan-4(1*H*)-one (S60)**

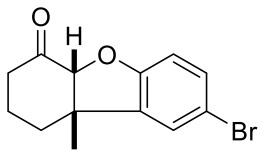

Ketone **2** (40.5 mg, 0.20 mmol) was dissolved in acetonitrile (2.0 mL) and the solution was cooled to 0 °C. NBS (37.4 mg, 0.21 mmol) was added and the reaction was stirred at 0 °C. After 1 h the cooling bath was removed and the reaction mixture was stirred at room temperature for an additional 24 h. The solvent was removed under reduced pressure and the residue was purified *via* flash chromatography (PE:Et_2O, 2:1) to afford **S60** (51.4 mg, 0.18 mmol, 91%, >20:1 d.r.) as a colourless solid.

m.p. 109-110°C ($CHCl_3$).

^{1}H NMR (500 MHz, $CDCl_3$) δ = 7.26 (dd, *J* = 8.5, 2.1 Hz, 1H), 7.14 (d, *J* = 2.1 Hz, 1H), 6.80 (d, *J* = 8.5 Hz, 1H), 4.47 (s, 1H), 2.59-2.51 (m, 1H), 2.36 (dddd, *J* = 15.7, 10.3, 6.5, 0.6 Hz, 1H), 2.07-1.99 (m, 1H), 1.97-1.88 (m, 1H), 1.83 (ddd, *J* = 14.1, 11.1, 3.2 Hz, 1H), 1.73-1.62 (m, 1H), 1.43 (s, 3H).

^{13}C NMR (126 MHz, $CDCl_3$) δ = 207.8, 258.2, 136.1, 131.6, 125.3, 113.7, 112.2, 92.1, 50.5, 38.4, 34.3, 28.3, 20.8.

FTIR (neat) ν/cm^{-1} = 2963, 1725, 1468, 1414, 1253, 1184, 1080, 1027, 987, 867, 812, 682.

HRMS (ESI) *m/z* calcd. for $C_{13}H_{13}O_2BrNa$ $[M+Na]^+$: 302.9991; found: 302.9992.

VII. Computational Procedures

Density functional theory (DFT) calculations were performed with Gaussian 09 rev. D.01.[24] All triplet and open-shell singlet species were described with unrestricted Kohn-Sham theory; converged densities were checked for their stability. Geometry optimisations used the M06-2X meta-GGA functional with the polarized triple-ζ Gaussian AO basis set def2-TZVP.[25,26] We and others have applied this method to the study of triplet-state reactivity previously;[27] uM06-2X performs well in describing radicals[28] and in this instance comparison with a B3LYP-D3(BJ)/def2-TZVP[29] energy profile showed negligible difference in thermochemistry or kinetics.

Minima and transition structures on the potential energy surface (PES) were confirmed as such by harmonic frequency analysis at the same level of theory. Minimum-energy crossing points (MECPs) along the seam of intersecting singlet (S_0) and triplet (T_1) surfaces were located using Harvey's *MECP* code interfaced to Gaussian 09, which minimizes the gradient difference vector between the two adiabatic surfaces.[30] A state-averaged vibrational analysis at the MECP was carried out with Glowacki's *glowfreq* code.[31] This confirmed the presence of 3N-6 positive, real normal modes which were used to compute the (quasi-harmonic) vibrational partition function and obtain the Gibbs energies of the crossing points. Note that an estimation of the rate of spin-forbidden intersystem crossing (ISC) also requires estimation of spin-hopping probabilities which were not been considered in this work.

Single point corrections with the SMD continuum solvation model[32] were applied to include the effect of ethyl acetate and acetonitrile solvents on the computed Gibbs energy profile. The reaction considered is unimolecular and so a standard state correction (from 1 atm to 1 mol/l) has no effect. Gibbs energies were evaluated at 298.15 K, using a quasi-RRHO treatment of vibrational entropies introduced by Grimme.[33] Vibrational entropies of frequencies below 100 cm^{-1} were obtained according to a free rotor description, using a smooth damping function to interpolate between the two limiting descriptions. This was implemented in Python as described previously.[34]

The Anisotropy of the Induced Current Density (ACID) method developed by Herges was used to probe the extent of cyclic electronic delocalisation in the cyclization transition structures ($^3TS_{AB}$) of **2** and **35**.[35] ACID plots were generated using the AICD-2.0.0 program at the B3LYP/6-311++G(d,p) level using the CSGT method.[36] The magnetic field is positioned perpendicular to the plane of the ring under investigation. Clockwise ring currents signify aromatic character while anti-clockwise ring currents are characteristic of anti-aromaticity.

Nucleus-Independent Chemical shift (NICS) calculations were carried out to further analyse the aromatic character of the triplet state transition state ($^3TS_{AB}$).[37] NMR calculations used the B3LYP/6-311++g(d,p) level of theory with Gauge-Including Atomic Orbitals.[38] Ghost atoms (Bq, after Shakespeare's Banquo) were positioned at the geometric ring centre and at positions 1Å above and below (i.e. NICS(0) and NICS(1)). The vector normal to the ring was obtained from the plane-of-best-fit, which was obtained by three dimensional least squares fitting using the ring heavy atoms. Shielding tensors were resolved into their out-of-plane (i.e.

σ_{zz}) components by taking the scalar product with the unit normal vector. All preparation and analysis was automated through Python scripts.[39]

Gaussian output files have been deposited and are freely available: 10.5281/zenodo.495385

All Python scripts have been made available - https://github.com/bobbypaton - under a creative commons CC-BY license.

Analysis of the reaction mechanism: we obtained the Gibbs energy profiles for products **2**, **28**, **35**. In each case we found the conrotatory pathway to be preferred by around 10 kcal/mol over the disrotatory closure. The cyclization TS is in every case the highest point along the intramolecular reaction coordinate. The structure of cyclized intermediate **B** is very similar in triplet and singlet states, where an open-shell singlet diradical is favoured over the closed-shell singlet. We located the MECP for **35**, which shows a small barrier (4.4 kcal/mol) for ISC from ^{3}**B** to ^{1}**B**. The intramolecular suprafacial [1,4]-H shift has a barrier of ca. 15 kcal/mol in which the conrotatory adduct leads to the *trans*-fused product, **C**. In the triplet state, this rearrangement is Woodward-Hoffmann disallowed and scans along the expected reaction coordinate showed extremely high energies. **TS$_{BC}$** is a closed-shell singlet which shows no wavefunction instability.

For each substrate the product cis-diastereomer is thermodynamically preferred by more than 5 kcal/mol. We have not computed epimerization activation barriers, however, we note that this could occur via (intermolecular) enolization in EtOAc or MeCN.

Figure S1. M06-2X/def2-TZVP(SMD=acetonitrile)//M06-2X/def2-TZVP quasiharmonic Gibbs energy profile (298K) for conrotatory and distrotatory reaction pathways leading to **2**.

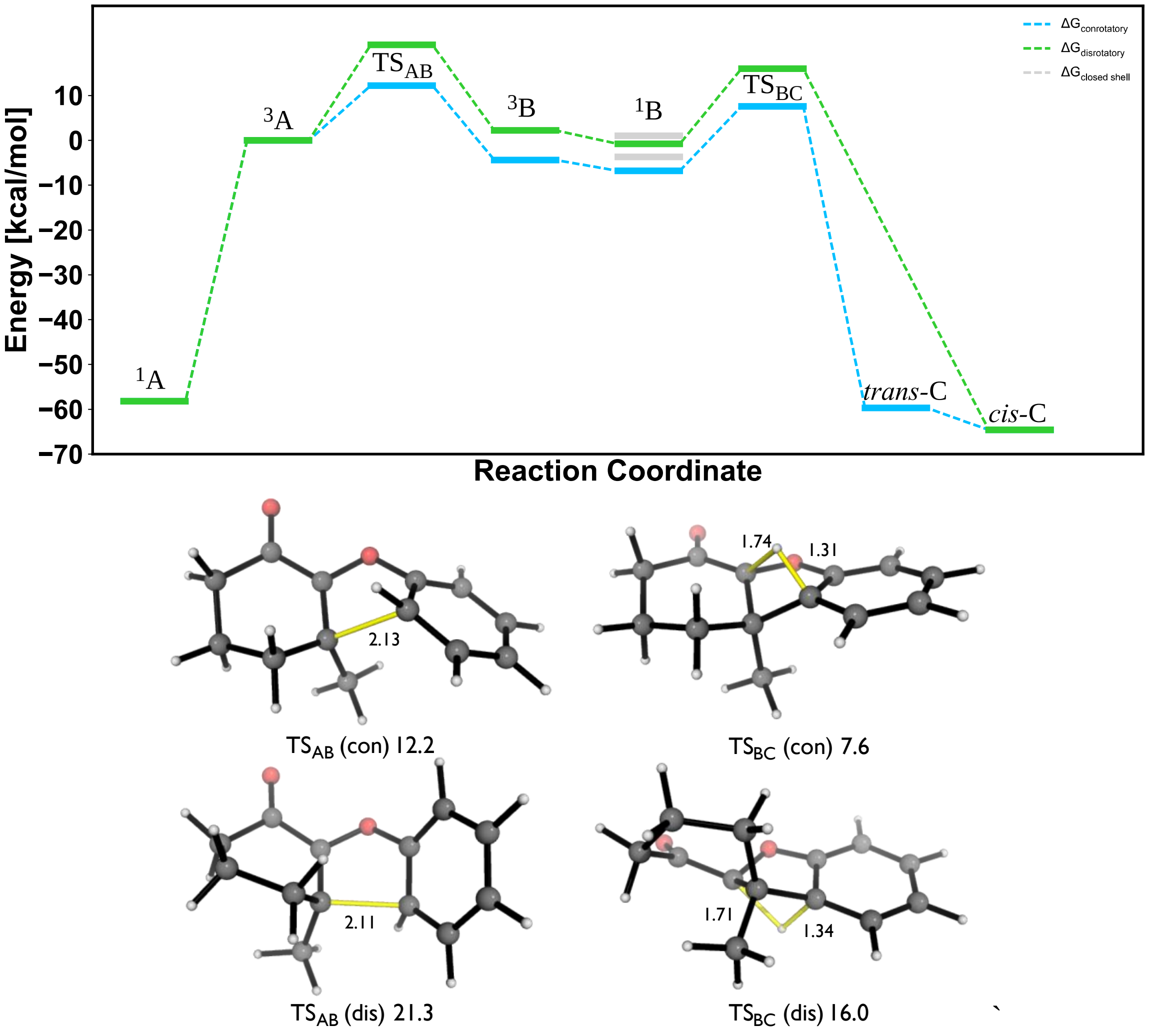

Figure S2. Comparison of M06-2X/def2-TZVP and B3LYP-D3(BJ)/def2-TZVP quasiharmonic Gibbs energy profile (298K) for conrotatory and distrotatory reaction pathways leading to **2**.

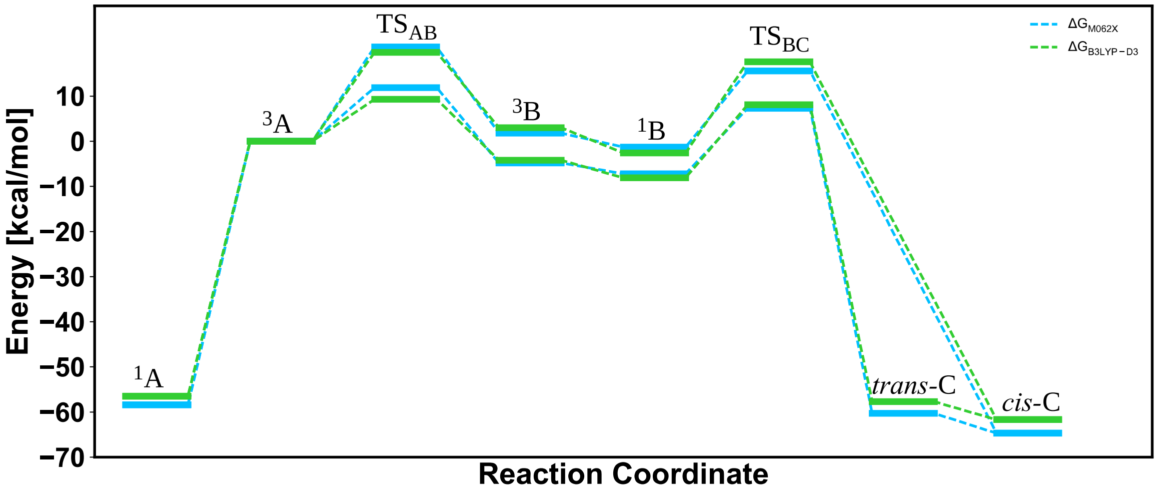

Table S1. Conrotatory and distrotatory reaction pathways leading to **2**. Absolute energy contributions to the Gibbs energy (Hartree) and imaginary frequencies (in cm^{-1}).

Product Compound No. 2		M06-2X/def2-TZVP						MeCN	B3LYP/def2-TZVP
		**E/au**	**ZPE/au**	**H/au**	**T.qh-S/au**	**qh-G(T)/au**	**imag. ν**	**E/au**	**E/au**
	^{1}A	-654.216665	0.241647	-653.96076	0.053237	-654.013997	-	-654.239042	-654.554779
	^{3}A	-654.120590	0.239631	-653.866592	0.054308	-653.920900	-	-654.143301	-654.464707
	^{3}A (n-p*)	-654.100776	0.237974	-653.848138	0.055072	-653.903210	-	-654.109619	-654.450318
Conrotatory path	TS_{AB}	-654.102839	0.239439	-653.850117	0.051828	-653.901945	-435.71	-654.124997	-654.449823
Conrotatory path	^{3}B	-654.131122	0.240864	-653.87702	0.051483	-653.928502	-	-654.153227	-654.471379
Conrotatory path	^{1}B (closed shell)	-654.126933	0.241957	-653.871859	0.050292	-653.922151	-	-654.154227	-654.471595
Conrotatory path	^{1}B (open shell)	-654.136063	0.240907	-653.881974	0.050345	-653.932319	-	-654.158124	-654.477581
Conrotatory path	TS_{BC}	-654.110872	0.238434	-653.859691	0.04961	-653.909301	-1182.47	-654.133402	-654.451737
Disrotatory path	TS_{AB}	-654.086737	0.238286	-653.834742	0.052791	-653.887533	-522.22	-654.108810	-654.433316
Disrotatory path	^{3}B	-654.119683	0.240317	-653.865841	0.052165	-653.918006	-	-654.141769	-654.459961
Disrotatory path	^{1}B (closed shell)	-654.121036	0.242094	-653.865748	0.050506	-653.916253	-	-654.146876	-654.465396
Disrotatory path	^{1}B (open shell)	-654.126380	0.240924	-653.87209	0.050827	-653.922920	-	-654.148364	-654.468920
Disrotatory path	TS_{BC}	-654.097604	0.23846	-653.846385	0.04966	-653.896046	-1271.63	-654.119743	-654.436669
	trans-C	-654.224721	0.244599	-653.967347	0.049674	-654.017022	-	-654.246444	-654.556707
	cis-C	-654.230653	0.243639	-653.974032	0.050031	-654.024063	-	-654.253078	-654.563005

Table S2. Conrotatory and distrotatory reaction pathways leading to **35** (for graphical PES see **Figure 1** in the main text). Absolute energy contributions to the Gibbs energy (Hartree) and imaginary frequencies (in cm^{-1}) for all structures.

Product Compound No. 35		M06-2X/def2-TZVP gas phase						in EtOAc
		E/au	ZPE/au	H/au	T.qh-S/au	qh-G(T)/au	imag. ν	E/au
	^{1}A	-614.905094	0.212762	-614.679085	0.051507	-614.730592	-	-614.925248
	^{3}A	-614.802221	0.210156	-614.578681	0.052389	-614.631070	-	-614.82227
	^{3}A (n-p*)	-614.800665	0.209569	-614.577604	0.052578	-614.630181	-	-614.818891
Conrotatory path	TS_{AB}	-614.782419	0.209919	-614.560217	0.049823	-614.610039	-436.30	-614.801912
	^{3}B	-614.811328	0.211432	-614.587679	0.049416	-614.637095	-	-614.830744
	^{1}B (closed shell)	-614.803772	0.212545	-614.579162	0.048151	-614.627313	-	-614.826994
	^{1}B (open shell)	-614.815095	0.211499	-614.591439	0.048265	-614.639704	-	-614.834520
	TS_{BC}	-614.787132	0.208862	-614.566549	0.047537	-614.614086	-1179.22	-614.807311
Disrotatory path	TS_{AB}	-614.764256	0.208941	-614.542896	0.050062	-614.592958	-502.10	-614.783497
	^{3}B	-614.800818	0.210909	-614.577619	0.049563	-614.627182	-	-614.819945
	^{1}B (closed shell)	-614.801167	0.212398	-614.57665	0.048285	-614.624935	-	-614.822938
	^{1}B (open shell)	-614.806000	0.210962	-614.58271	0.048659	-614.631370	-	-614.825179
	TS_{BC}	-614.776623	0.208544	-614.556399	0.047411	-614.603810	-1212.24	-614.795898
	trans-C	-614.894944	0.21504	-614.668172	0.047516	-614.715688	-	-614.913867
	cis-C	-614.918525	0.214554	-614.691951	0.048242	-614.740193	-	-614.937409

Figure S3. M06-2X/def2-TZVP(SMD=acetonitrile)//M06-2X/def2-TZVP quasiharmonic Gibbs energy profile showing conrotatory and distrotatory reaction pathways leading to **28**.

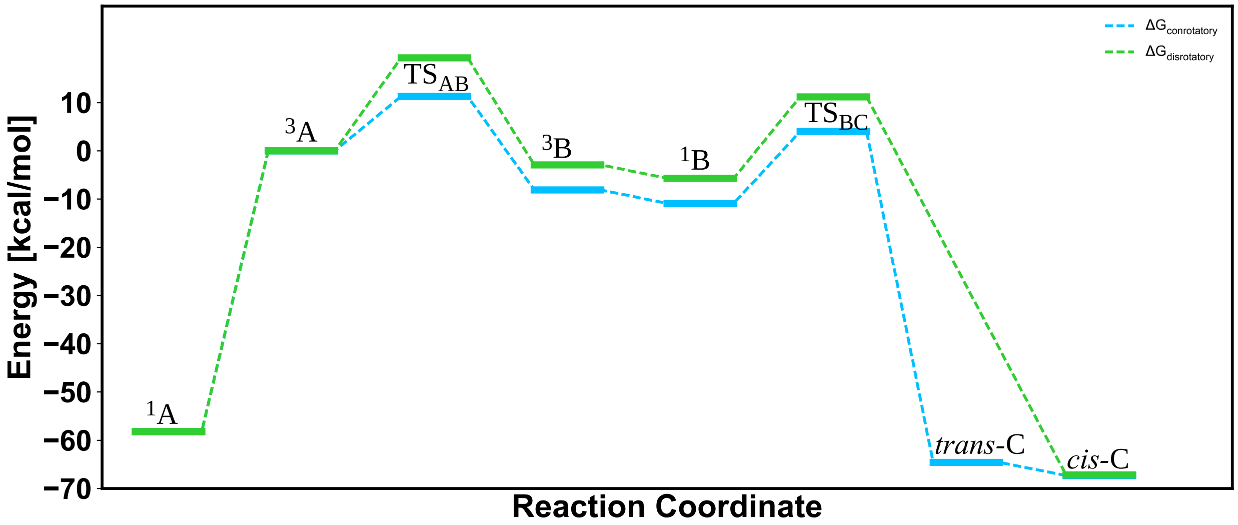

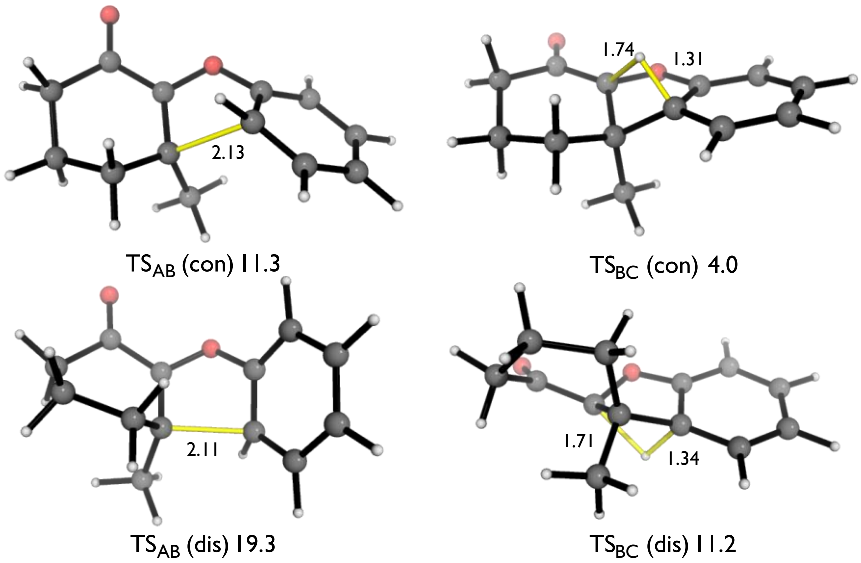

Table S3. Conrotatory and distrotatory reaction pathways leading to **28**. Absolute energy contributions to the Gibbs energy (Hartree) and imaginary frequencies (in cm^{-1}) for all structures.

Product Compound No. 28		M06-2X/def2-TZVP gas phase						in EtOAc
		E/au	ZPE/au	H/au	T.qh-S/au	qh-G(T)/au	imag. ν	E/au
	^{1}A	-614.902113	0.213855	-614.675765	0.049463	-614.725227	-	-614.923843
	^{3}A	-614.805275	0.211193	-614.581256	0.051215	-614.632471	-	-614.827887
Conrotatory path	TS$_{AB}$	-614.788728	0.211021	-614.565930	0.048956	-614.614886	-438.71	-614.810859
	^{3}B	-614.821316	0.212703	-614.596748	0.048985	-614.645733	-	-614.843630
	^{1}B (open shell)	-614.827466	0.213016	-614.602671	0.047799	-614.65047	-	-614.849505
	TS$_{BC}$	-614.801325	0.210414	-614.579622	0.046904	-614.626526	-1212.66	-614.823531
Disrotatory path	TS$_{AB}$	-614.774799	0.210814	-614.551951	0.049512	-614.601462	-530.77	-614.796528
	^{3}B	-614.811393	0.212531	-614.586733	0.049660	-614.636393	-	-614.833566
	^{1}B (open shell)	-614.818202	0.213146	-614.593101	0.048278	-614.641379	-	-614.840345
	TS$_{BC}$	-614.789284	0.210731	-614.567136	0.047267	-614.614403	-1262.29	-614.811613
	trans-C	-614.916417	0.216532	-614.688462	0.047208	-614.73567	-	-614.938727
	cis-C	-614.920069	0.216316	-614.692231	0.047394	-614.739625	-	-614.942708

Analysis of regioselectivity: for products **11**, **12** and **39** we computed the TSs leading to the two regioisomers (**Figure S4**): we believe that this step is irrerversible and hence governs the regioselectivity. The modest (ca. 2 :1) ortho-selectivity of cyclohexanones (**11**,**12**) is reproduced by our calculations, with the favoured TS occurring earlier in each case. The regioselectivity is smaller in the case of cyclopentanone **39**, although the calculations fail to predict the favouring of the para-product (1: 1.6). However, given the small Gibbs energy differences involved (< 1 kcal/mol), this is likely within DFT errors.

Figure S4. M06-2X/def2-TZVP(SMD=acetonitrile)//M06-2X/def2-TZVP “ortho” and “para” cyclization TSs leading to the two regioisomers of **12**, **11** and **39**.

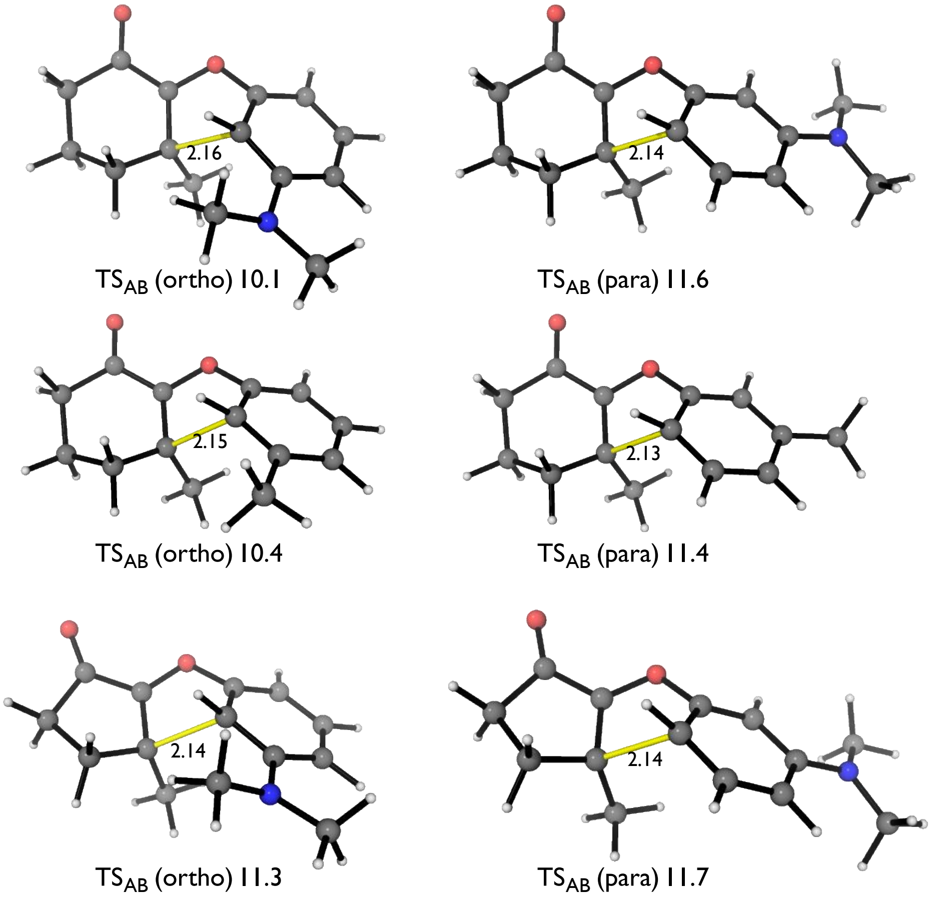

Table S4. “ortho” and “para” cyclization TSs leading to the two regioisomers of **12**, **11** and **39**. Absolute energy contributions to the Gibbs energy (Hartree) and imaginary frequencies (in cm^{-1}).

			**M06-2X/def2-TZVP gas phase**						**in solvent**
			**E/au**	**ZPE/au**	**H/au**	**T.qh-S/au**	**qh-G(T)/au**	**imag. ν**	**E/au**
**Product Compound No.12**		^1A	-788.^{17}3253	0.314984	-787.839594	0.062449	-787.902042	-	-788.199501
		^3A	-788.078153	0.313028	-787.746457	0.063197	-787.809654	-	-788.104256
	Ortho path	TS_{AB}	-788.062017	0.312473	-787.731886	0.060733	-787.792618	- 432.54	-788.087475
		^3B	-788.093234	0.31538	-787.76078	0.059083	-787.819863	-	-788.118128
	Para path	TS_{AB}	-788.060859	0.312424	-787.730684	0.061067	-787.791751	- 423.52	-788.086300
		^3B	-788.090567	0.314709	-787.758191	0.060705	-787.818895	-	-788.116167
**Product Compound No.11**		^1A	-693.527226	0.269385	-693.241845	0.056827	-693.298671	-	-693.550141
		^3A	-693.431250	0.267193	-693.147882	0.058108	-693.20599	-	-693.454458
	Ortho path	TS_{AB}	-693.414946	0.266624	-693.133151	0.055729	-693.18888	-462.01	-693.437460
		^3B	-693.444481	0.268145	-693.161367	0.054948	-693.216315	-	-693.467202
	Para path	TS_{AB}	-693.413452	0.266478	-693.131841	0.055606	-693.187446	-446.48	-693.435939
		^3B	-693.442477	0.268328	-693.159061	0.055334	-693.214395	-	-693.464925
**Product Compound No.39**		^1A	-748.862321	0.285773	-748.558897	0.060622	-748.619519	-	-748.885374
		^3A	-748.759968	0.283460	-748.458717	0.061591	-748.520308	-	-748.782947
	Ortho path	TS_{AB}	-748.742441	0.283451	-748.442467	0.058425	-748.500892	-432.56	-748.764684
		^3B	-748.774475	0.285637	-748.472619	0.057472	-748.530091	-	-748.796655
	Para path	TS_{AB}	-748.740582	0.282818	-748.441009	0.059109	-748.500118	-428.21	-748.763158
		^3B	-748.770889	0.285245	-748.469001	0.058699	-748.527699	-	-748.793418

Figure S5. M06-2X/def2-TZVP(SMD=acetonitrile)//M06-2X/def2-TZVP quasiharmonic Gibbs energy profile showing conrotatory reaction pathway which would lead to **58**.

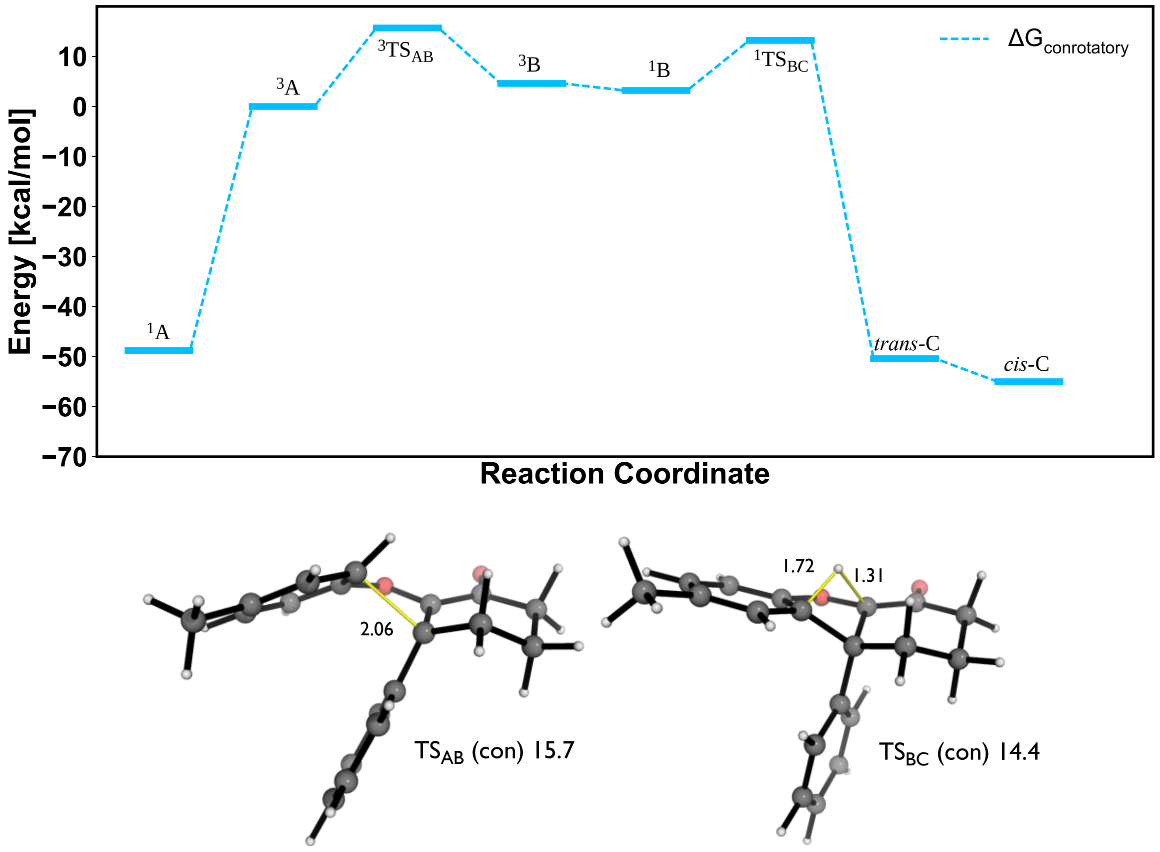

Table S5. Conrotatory reaction pathways leading to **58**. Absolute energy contributions to the Gibbs energy (Hartree) and imaginary frequencies (in cm^{-1}) for all structures.

Product Compound No. 58		M06-2X/def2-TZVP gas phase						in MeCN
		E/au	ZPE/au	H/au	T.qh-S/au	qh-G(T)/au	imag. ν	E/au
	^{1}A	-885.254679	0.322987	-884.912636	0.063937	-884.976573	-	-885.282494
	^{3}A	-885.172477	0.320647	-884.832603	0.064636	-884.897239	-	-885.201783
Conrotatory path	TS_{AB}	-885.149854	0.320401	-884.811306	0.06197	-884.873276	-467.17	-885.178136
	^{3}B	-885.169667	0.322132	-884.829444	0.061673	-884.891117	-	-885.197785
	^{1}B (open shell)	-885.175197	0.321863	-884.835211	0.060742	-884.895953	-	-885.20064
	TS_{BC}	-885.152537	0.319587	-884.815253	0.060034	-884.875287	-1224.94	-885.180861
	trans-C	-885.260919	0.325406	-884.917757	0.060139	-884.977896	-	-885.287864
	cis-C	-885.266658	0.325345	-884.923387	0.060629	-884.984016	-	-885.294231

Figure S6. M06-2X/def2-TZVP(SMD=acetonitrile)//M06-2X/def2-TZVP quasiharmonic Gibbs energy profile showing conrotatory reaction pathway which would lead to **59**.

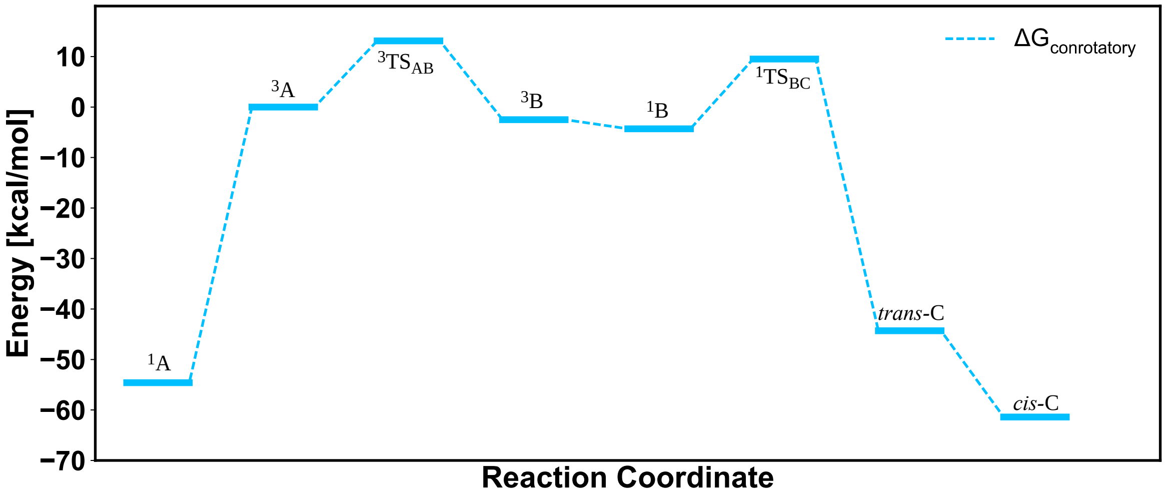

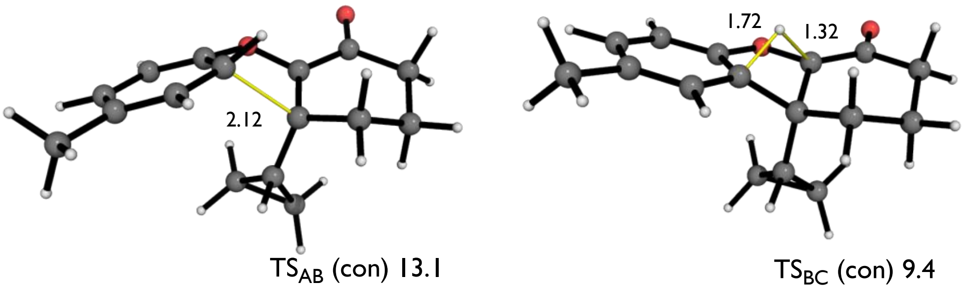

Table S6. Conrotatory reaction pathways leading to **59**. Absolute energy contributions to the Gibbs energy (Hartree) and imaginary frequencies (in cm^{-1}) for all structures.

Product Compound No. 59		M06-2X/def2-TZVP gas phase						in MeCN
		**E/au**	**ZPE/au**	**H/au**	**T.qh-S/au**	**qh-G(T)/au**	**imag. ν**	**E/au**
	^{1}A	-770.907566	0.304359	-770.5857	0.060143	-770.645842	-	-770.932651
	^{3}A	-770.816483	0.301965	-770.496828	0.061303	-770.558131	-	-770.842273
	^{3}A (n-p*)	-770.793267	0.300742	-770.474598	0.061932	-770.53653	-	-770.806526
Conrotatory path	TS_{AB}	-770.797873	0.301679	-770.479676	0.058518	-770.538195	-428.16	-770.822746
	^{3}B	-770.825045	0.303288	-770.505262	0.058236	-770.563498	-	-770.849463
	^{1}B (open shell)	-770.830593	0.303161	-770.510882	0.057368	-770.56825	-	-770.853171
	TS_{BC}	-770.804267	0.300993	-770.487229	0.056477	-770.54376	-1228.93	-770.82939
	trans-C	-770.913356	0.306842	-770.591173	0.055002	-770.646174	-	-770.921772
	cis-C	-770.921772	0.306248	-770.599186	0.05716	-770.656346	-	-770.947187

Figure S7. Comparison of substrate singlet (S_0) to triplet (T_1) M06-2X/def2-TZVP Gibbs energies (kcal/mol) and optimized dihedral angles (°) around the enone C=C double bond. Atom labelling for dihedral angles around the enone C-C double bond shown in table **S7**.

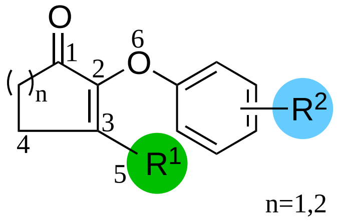

n=1,2

Product compound no.	M06-2X/def2-TZVP			
	**ΔG (^{1}A – ^{3}A)**	**^{3}A ϕ 1234**	**^{3}A ϕ 5321**	**^{3}A ϕ 6234**
**2**	58.2	29.6	-114.3	-155.2
**2** ($^3A_{(n-p*)}$)	78.0	-1.7	177.8	174.9
**11**	58.0	29.4	-114.4	-155.5
**12**	58.1	29.4	-114.4	-155.6
**26**	57.1	34.4	-111.7	-146.4
**28**	57.7	28.3	-115.0	-155.5
**35**	62.5	21.2	-115.0	-165.8
**35** ($^3A_{(n-p*)}$)	64.2	-13.3	-158.2	175.1
**39**	62.3	21.2	-115.1	-165.8
**58**	48.8	37.2	-122.0	-143.8
**59**	54.6	35.7	-108.0	-145.8
**59** ($^3A_{(n-p*)}$)	76.0	-4.6	176.3	171.9

Figure S8 Comparison of optimized triplet (T_1) and MECP (S_0/T_1) geometries for substrates leading to products **35**, **02**, **58** and **59**.

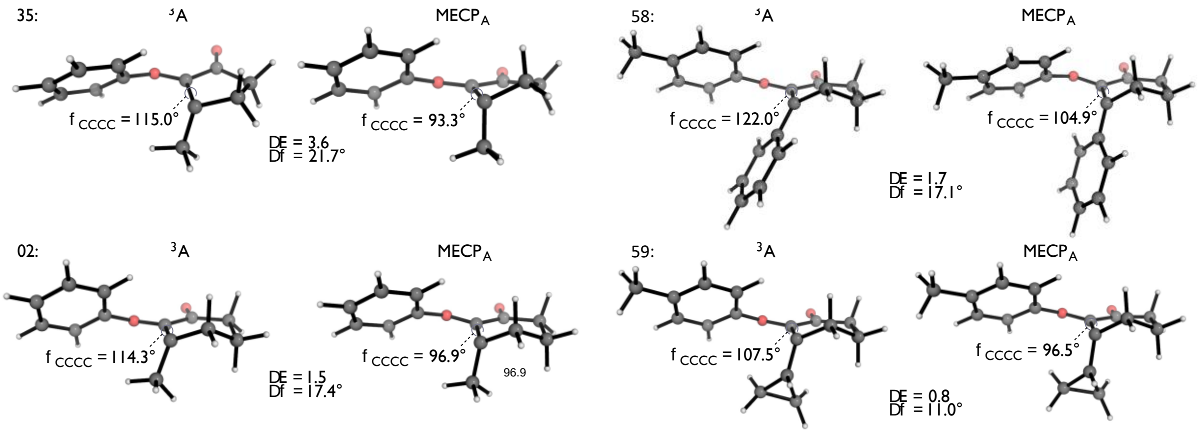

Analysis of the cyclization TS: Frontier Orbital correlation diagrams are shown below for a 5 membered ring system with (4n+2)π electrons undergoing a disrotatory 6π-cyclization in the singlet state, and a conrotatory 6π-cyclization and triplet spin states. Both cyclization transition states may be classified as aromatic in the Hückel and Zimmerman-Möbius sense,

respectively, and are expected to exhibit magnetic criteria associated with aromaticity. We performed out-of-plane $NICS_{zz}$, magnetic susceptibility exaltation and ACID calculations to verify this, beginning with the prototypical example of singlet-state and triplet-state 6π-electrocyclization of divinyl ether (**Figure S9**). Such computational approaches have been used to characterize the aromaticity of cyclization transition structures by Alabugin;[40] we have also done so for 6π-electrocyclizations/5-*endo-trig* cyclizations.[41]

For the simple prototypes the disrotatory S_0 TS is Cs symmetric, while the conrotatory T_1 TS optimizes in the C_1 point group – there is greater localization of the spin on one alkene in the TS. In both cases the NICS(0) values are negative at the ring centre, as are NICS(1) values above/below these points, the effect being more pronounced for the singlet TS. Such negative values are consistent with shielding from diatropic ring currents associated with aromaticity. These are clearly visible in the ACID calculations performed on the singlet TS, while the triplet does not show cyclic delocalization to the same extent. Negative values of the magnetic susceptibility exaltation for both TSs are consistent with aromaticity in each case. To summise these results, computed NICS values illustrate the pericyclic nature of the singlet disrotatory closure and the triplet conrotatory closure of divinyl ether. Greater aromaticity in the former case is apparent from more negative NICS values and cyclic delocalization is evident in the ACID analysis (which is not obtained for the triplet TS).

Figure S9. M06-2X/def2-TZVP optimised geometries and ACID plots for the 6π-electrocyclization TS of divinyl ether in singlet (disrotatory) and triplet (conrotatory) spin states. B3LYP/6-311++G(d,p) NICS (ppm) and magnetic susceptibility exaltation, Λ, values (ppm cgs).

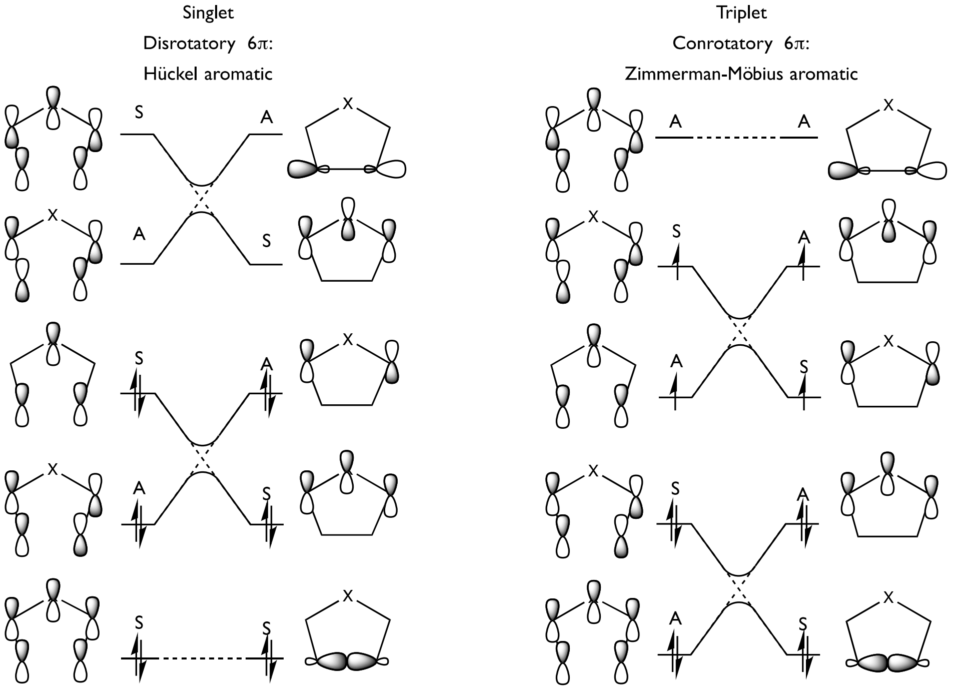

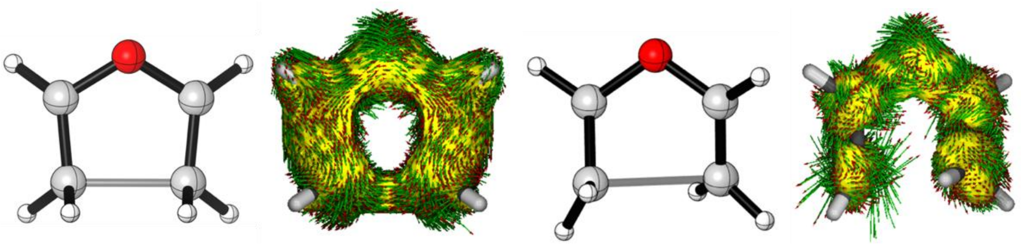

Divinyl ether		$NICS(-1)_{zz}$	$NICS(0)_{zz}$	$NICS(1)_{zz}$	Λ
Disrotatory	S_0	-13.3	-10.5	-23.7	-40.9
Conrotatory	T_1	-2.1	-13.1	-2.5	-38.7

Looking now at the real reactants, the same magnetic properties were calculated for conrotatory and disrotatory TSs (**Figures S10** and **S11**). NICS calculations show shielding above and below the forming five-membered ring only in the conrotatory TSs which is absent in the disrotatory TSs. The ACID analysis does not show cyclic delocalization around the 5-membered ring in either case. Shielding in the conrotatory TS is confined to regions above and below the plane which contrasts to the model substrate above, for which the shielding is largest in the center of the forming ring. It is likely that the negative NICS values obtained for the conrotatory TSs below are the result of local effects due to the aromatic ring and enone π-systems. We obtained similar NICS results for TSs in which the O is replaced by CH_2, thereby eliminating the possibility of cyclic delocalization, which supports this interpretation.

Finally, there is no change in the magnitude of the magnetic susceptibility exaltation in proceeding from the reactant to TS for substrates **2** and **35**, again indicative of little involvement of aromaticity in either TS. The marked preference for the conrotatory pathway is at least partly the result of diminished steric interactions, since the forming C-C bond is eclipsed in the disrotatory TS.

Figure S10. ACID and NICS data for conrotatory and disrotatory cyclization TSs ($^3TS_{AB}$) leading to the formation of **35**.

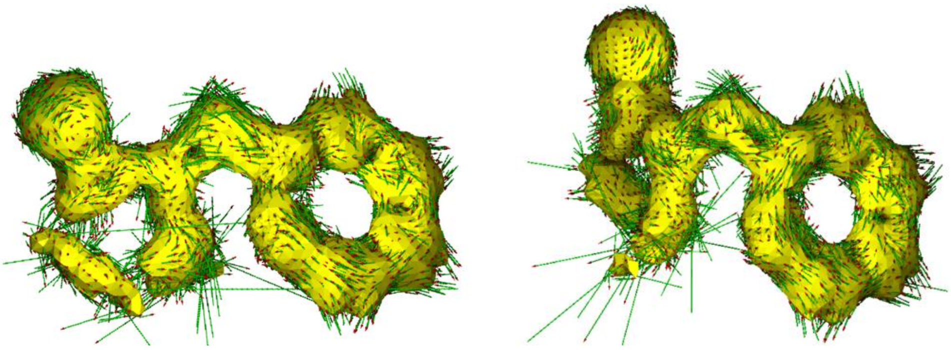

Product 35		$NICS(-1)_{zz}$	$NICS(0)_{zz}$	$NICS(1)_{zz}$
Conrotatory	TS_{AB}	-5.2	7.5	-5.9
Disrotatory	TS_{AB}	7.3	14.0	-1.0

Figure S11. ACID and NICS data for conrotatory and disrotatory cyclization TSs ($^3TS_{AB}$) leading to the formation of **2**.

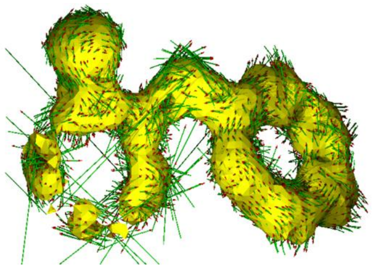

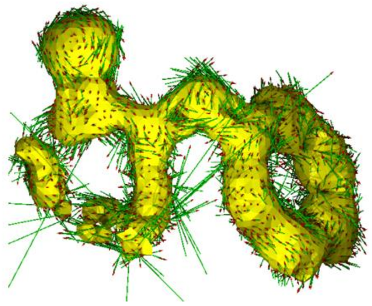

Product 2		NICS(-1)$_{zz}$	NICS(0)$_{zz}$	NICS(1)$_{zz}$
Conrotatory	TS_{AB}	-7.3	5.5	-7.4
Disrotatory	TS_{AB}	2.0	15.9	-1.4

VIII. References

[1] A. B. Pangborn, M. A. Giardello, R. H. Grubbs, R. K. Rosen, F. J. Timmers, *Organometallics* **1996**, *15*, 1518.
[2] A. B. Tamayo, B. D. Alleyne, P. I. Djurovich, S. Lamansky, I. Tsyba, N. N. Ho, R. Bau, M. E. Thompson, *J. Am. Chem. Soc*. **2003**, *125*, 7377.
[3] N. J. A. Martin, B. List, *J. Am. Chem. Soc.* **2006**, *128,* 13368.
[4] S. C. Coote, P. O'Brien, A. C. Whitwood, *Org. Biomol. Chem.* **2008**, *6*, 4299.
[5] J. B. Tuttle, S. G. Ouellet, D. W. C. MacMillan, *J. Am. Chem. Soc.* **2006**, *128* , 12662.
[6] S. Collins, Y. Hong, M. Mataoka, T. Nguyen, *J. Org. Chem.* **1990**, *55*, 3395.
[7] E. Piers, J. Banville, C. Kun Lau, I. Nagakura, *Can. J. Chem.*, **1982**, *60*, 2965.
[8] P. Yates, G. E. Lanford, *Can. J. Chem*. **1981**, 59, 344.
[9] D. Felix, C. Wintner, A. Eschenmoser, *Org. Synth.* **1976**, *55*, 52.
[10] B. Rissa, N. Rachiqi, A. El Louzi, A. Loupy, A. Petit, S. Fkih-Tétouania, *Tetrahedron* **2001**, *57*, 2761.
[11] David L. Coffen, D. G. Korzan, *J. Org. Chem.* **1971**, *36*, 390.
[12] S. G. Yang,J. P. Hwang,M. Young Park,K. Leeb, Y. H. Kim, *Tetrahedron* **2007**, *63*, 5184.
[13] M. T. Langin-Lanteri, J. Huet, *Synthesis* **1976**; *8*, 541.
[14] X. Wang, C. M. Reisinger, B. List, *J. Am. Chem. Soc.* **2008**, *130*, 6070.
[15] O. Lifchits, M. Mahlau, C. M. Reisinger, A. L., Christophe Farès, I. Polyak, G. Gopakumar, W. Thiel, B. List, *J. Am. Chem. Soc.* **2013**, *135*, 6677.
[16] A. Lee, C. M. Reisinger, B. List, *Adv. Synth. Catal.* **2012**, *354*, 1701.
[17] B. G. Jadhav and S. D. Samant, *Russ. J. Org. Chem.* **2014**, *50*, 1301.
[18] S. Chu, N. Münster, T. Balan, M. D. Smith, *Angew. Chem. Int. Ed.* **2016**, *55*, 14306.
[19] A. G. Schultz, R. D. Lucci, W. Y. Fu, M. H. Berger, J. Erhardt, W. K. Hagmand, *J. Am. Chem. Soc.* **1978**, *100*, 2150.
[20] K. Sato, S. Inoue, S. Kuranami, M. Ōhashi, *J. Chem. Soc., Perkin Trans. 1* **1977**, 1666.
[21] Synthesized according to literature known procedure: N. Fan, J.-J. Tang, H. Li, X.-J. Li, B. Luo, J.-M. Gao, *Eur. J. Med. Chem*. **2013**, *69*, 182.
[22] S. Nomura, K. Arimitsu, S. Yamaguchi, Y. Kosuga, Y. Kakimoto, T. Komai, K. Hasegawa, A. Nakanishi, T. Miyoshi, H. Iwasaki, M. Ozeki, I. Kawasaki, A. Kurume, S. Ohta, M. Yamashita *Chem. Pharm. Bull*. **2012**, *60*, 94.
[23] F. Toda , H. Miyamoto S. Kikuchi, J. Am. Chem. Soc. **1996**, *118*, 11315.
[24] Gaussian 09, Revision D.01, M. J. Frisch, G. W. Trucks, H. B. Schlegel, G. E. Scuseria, M. A. Robb, J. R. Cheeseman, G. Scalmani, V. Barone, B. Mennucci, G. A. Petersson, H. Nakatsuji, M. Caricato, X. Li, H. P. Hratchian, A. F. Izmaylov, J. Bloino, G. Zheng, J. L. Sonnenberg, M. Hada, M. Ehara, K. Toyota, R. Fukuda, J. Hasegawa, M. Ishida, T. Nakajima, Y. Honda, O. Kitao, H. Nakai, T. Vreven, J. A. Montgomery, Jr., J. E. Peralta, F. Ogliaro, M. Bearpark, J. J. Heyd, E. Brothers, K. N. Kudin, V. N. Staroverov, R. Kobayashi, J. Normand, K. Raghavachari, A. Rendell, J. C. Burant, S. S. Iyengar, J. Tomasi, M. Cossi, N. Rega, J. M. Millam, M. Klene, J. E. Knox, J. B. Cross, V. Bakken, C. Adamo, J. Jaramillo, R. Gomperts, R. E. Stratmann, O. Yazyev, A. J. Austin, R. Cammi, C. Pomelli, J. W.

Ochterski, R. L. Martin, K. Morokuma, V. G. Zakrzewski, G. A. Voth, P. Salvador, J. J. Dannenberg, S. Dapprich, A. D. Daniels, Ö. Farkas, J. B. Foresman, J. V. Ortiz, J. Cioslowski, and D. J. Fox, Gaussian, Inc., Wallingford CT, **2009**.
[25] Y. Zhao, D. Truhlar, *Theor. Chem. Acc.* **2008**, *120*, 215.
[26] (a) A. Schaefer, H. Horn and R. Ahlrichs, *J. Chem. Phys.* **1992**, *97*, 2571; (b) A. Schaefer, C. Huber, and R. Ahlrichs, *J. Chem. Phys*. **1994**, *100*, 5829.
[27] (a) R. A. Matute, K. N. Houk, *Angew. Chem. Int. Ed.* **2012**, *51*, 13097; (b) R. A. Matute, M. A. Garcia-Garibay, and K. N. Houk, *Org. Lett.* **2014**, *16*, 5232; (c) G. Jimeńez-Oseś, P. Liu, R. A. Matute, and K. N. Houk, *Angew. Chem. Int. Ed.* **2014**, *53*, 8664; (d) R. K. Mohamed, S. Mondal, K. Jorner, T. F. Delgado, V. V. Lobodin, H. Ottosson, and I. V. Alabugin, *J. Am. Chem. Soc.* **2015**, *137*(49), 15441; (e) E. Gorobets, N. E. Wong, R. S. Paton, and D J. Derksen, *Org. Lett.* **2017**, *19* (3), 484.
[28] D. G. Truhlar, *J. Phys. Chem. Lett.* **2008**, *112*, 1095.
[29](a) A. D. Becke, *J. Chem. Phys.* **1993**, *98*, 5648 (b) C. Lee, W. Yang, R. G. Parr, *Phys. Rev. B*, **1988**, *37*, 785. (c) S. H. Vosko, L. Wilk, M. Nusair, *Can. J. Phys.*, **1980**, *58*, 1200. (d) P. J. Stephens, F. J. Devlin, C. F. Chabalowski, and M. J. Frisch, *J. Phys. Chem.*, **1994**, *98*, 11623. (e) S. Grimme, J. Antony, S. Ehrlich, and S. Krieg, *J. Phys. Chem.*, **2010**, *132*, 154104.
[30] J. N. Harvey, M. Aschi, H. Schwarz, W. Koch, *Theor. Chem. Acc.* **1998,** *99*, 95.
[31] K. L. Gannon, M. A. Blitz, C. Liang, M. J. Pilling, P. W. Seakins, D. R. Glowacki, and J. N. Harvey, *Farad. Discuss.* **2010**, *147*, 173.
[32] A.V. Marenich, C.J. Cramer and D. G. Truhlar, *J. Phys. Chem. B* **2009**, *113*, 6378.
[33] S. Grimme, *Chem. Eur. J.* **2012**, *18*, 9955.
[34] I. Funes-Ardoiz, R. S. Paton, (2016) GoodVibes v1.0.1. http://doi.org/10.5281/zenodo.60811
[35] (a) R. Herges, D. Geuenich, *J. Phys. Chem. A* **2001**, *105*, 3214; (b) D. Geunenich, K. Hess, F. Kohler, R. Herges, *Chem. Rev.* **2005**, *105*, 3758.
[36] T. A. Keith, R. F. W. Bader, *Chem. Phys. Lett.* **1993**, *210*, 223.
[37] (a) P. von Ragué Schleyer, C. Maerker, A. Dransfeld, H. Jiao, N. J. R. van Eikema Hommes, *J. Am. Chem. Soc.* **1996**, *118*, 6317; (b) K. E. Jackson, and R. S. Paton "NMR prediction" chapter to appear in "Applied Theoretical Organic Chemistry", D. J. Tantillo (ed.) World Scientific Publishing.
[38] J. R. Cheeseman, G. W. Trucks, T. A. Keith, and M. J. Frisch, *J. Chem. Phys.*, **1996**, *104*, 5497.
[39] K. Jackson, PhD thesis, **2016**, University of Oxford: Oxford Research Archive uuid:16ccc310-0cf4-44f6-b999-adb451f0f873
[40] (a) K. Gilmore, M. Manoharan, J. I.-C. Wu, P. v. R. Schleyer, I. V. Alabugin *J. Am. Chem. Soc.* **2012**, *134*, 10584; (b) R. K. Mohamed, S. Mondal, K. Jorner, T. F. Delagdo, V. V. Lobodin, H. Orrosson, and I. V. Alabugin, *J. Am. Chem. Soc.* **2015**, *137*, 15441.
[41] (a) P. C. Knipe, M. Gredičak, A. Cernijenko, R. S. Paton, M. D. Smith, *Chem. Eur. J.* **2014**, *11*, 3005; (b) C. P. Johnston, A. Kothari, A., T. Sergeieva, S.I. Okovytyy, K. E. Jackson, R. S. Paton, M. D. Smith, *Nature Chem.* **2015**, *7*, 171; (c) Q. Peng, R. S. Paton, *Acc. Chem. Res.* **2016**, *49*, 1042.

IX. NMR Spectra

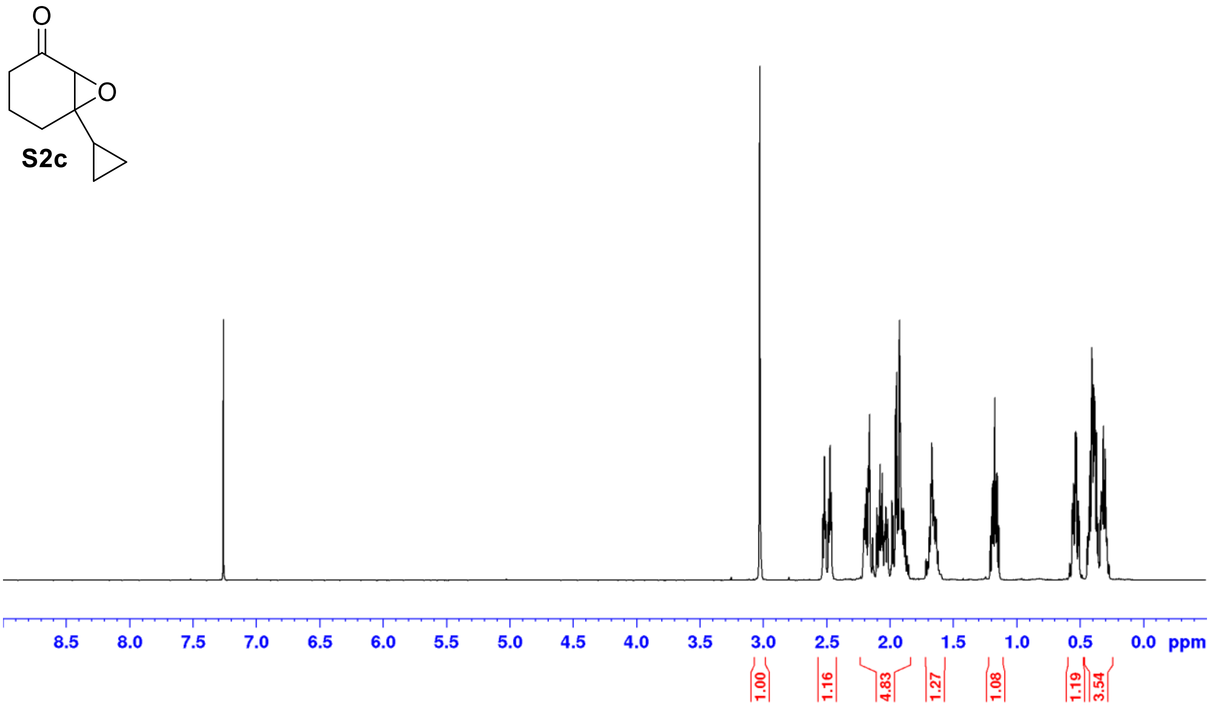

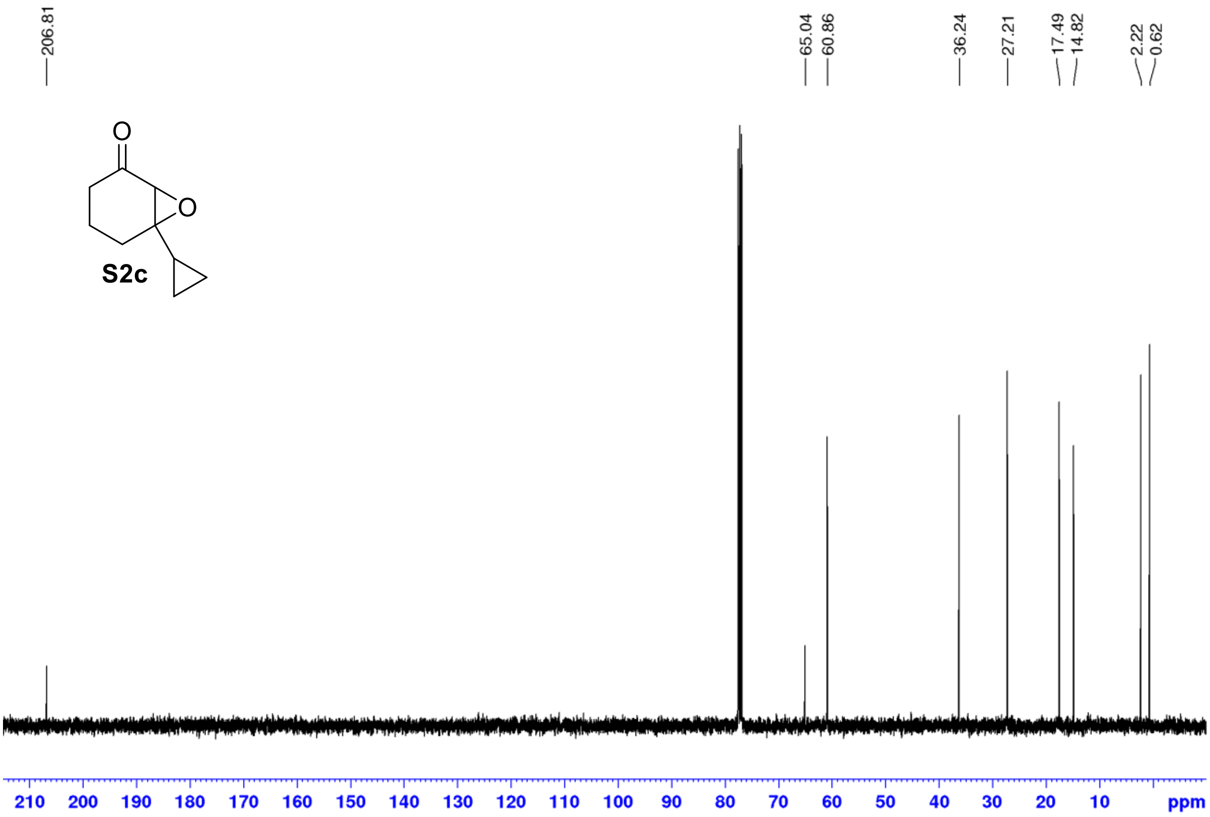

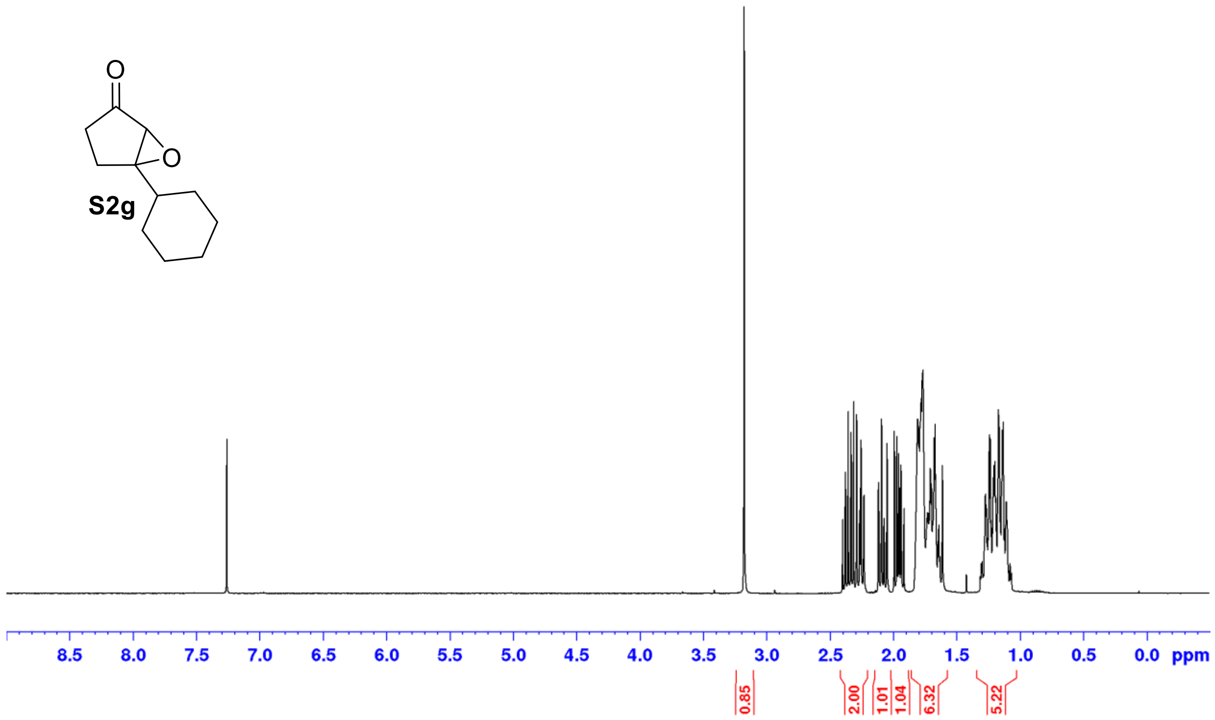

O
O
S2g
8.5
8.0
7.5
7.0
6.5
6.0
5.5
5.0
4.5
4.0
3.5
3.0
2.5
2.0
1.5
1.0
0.5
0.0
ppm
0.85
2.00
1.01
1.04
6.32
5.22

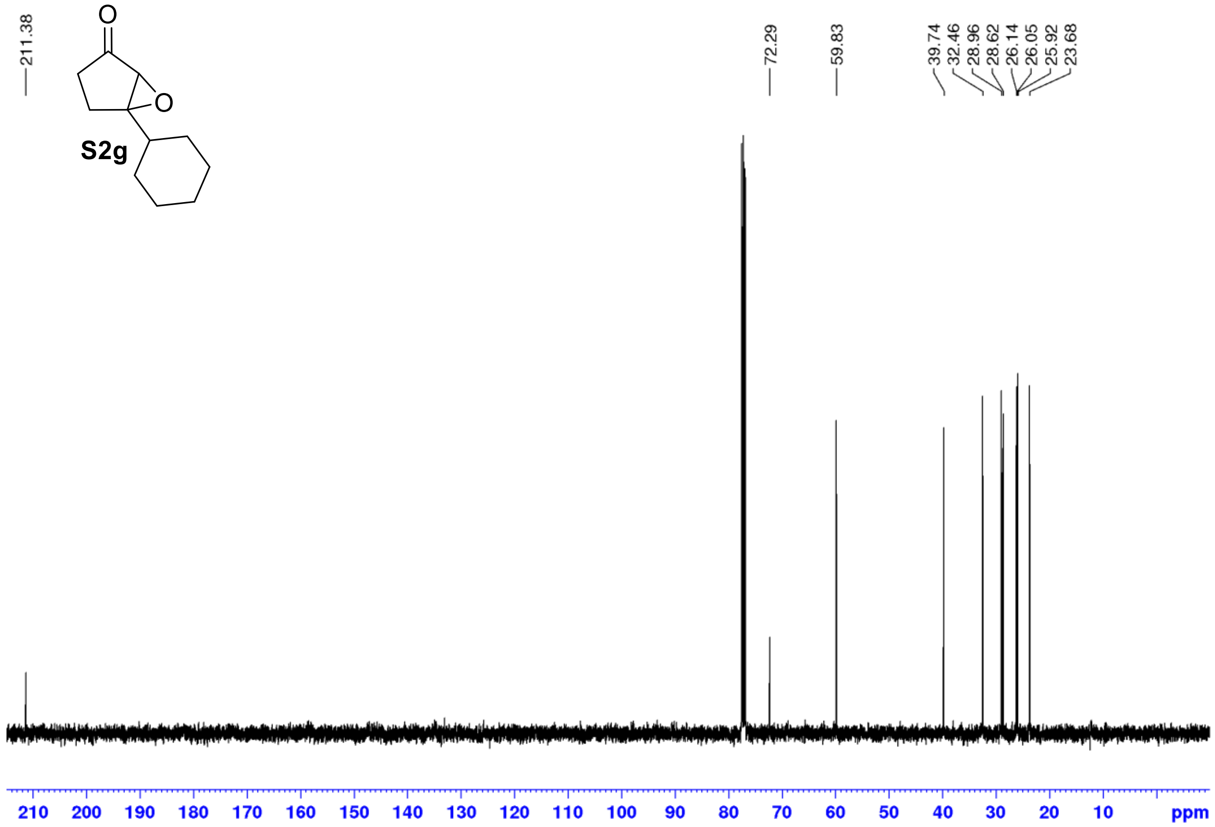

211.38
O
O
S2g
72.29
59.83
39.74
32.46
28.96
28.62
26.14
26.05
25.92
23.68
210
200
190
180
170
160
150
140
130
120
110
100
90
80
70
60
50
40
30
20
10
ppm

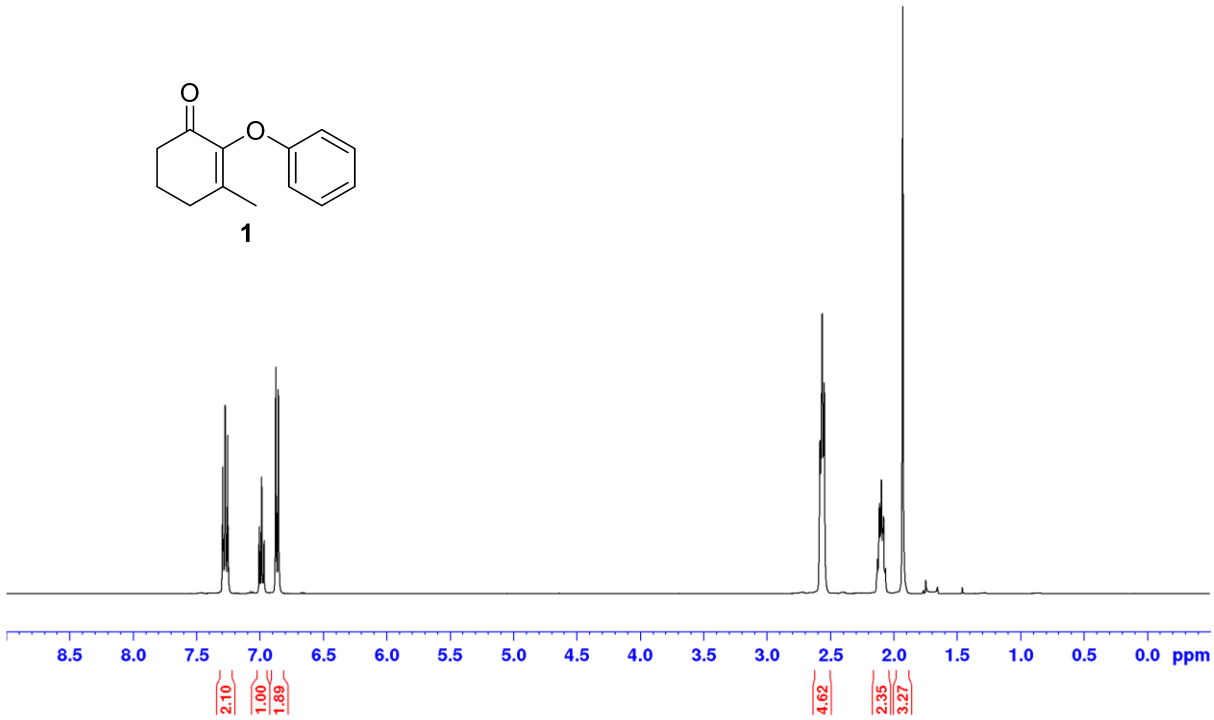

1
8.5 8.0 7.5 7.0 6.5 6.0 5.5 5.0 4.5 4.0 3.5 3.0 2.5 2.0 1.5 1.0 0.5 0.0 ppm
2.10 1.00 1.89 4.62 2.35 3.27

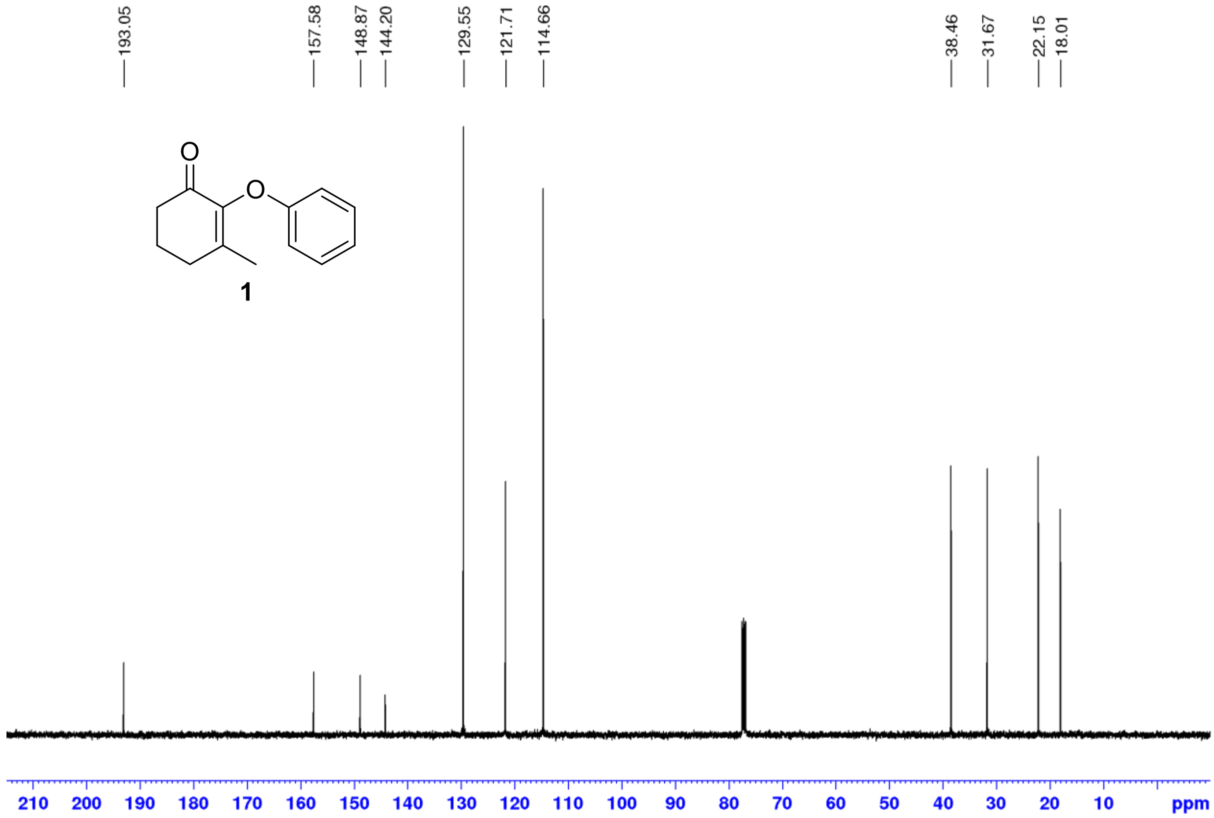

193.05
157.58
148.87
144.20
129.55
121.71
114.66
38.46
31.67
22.15
18.01
1
210 200 190 180 170 160 150 140 130 120 110 100 90 80 70 60 50 40 30 20 10 ppm

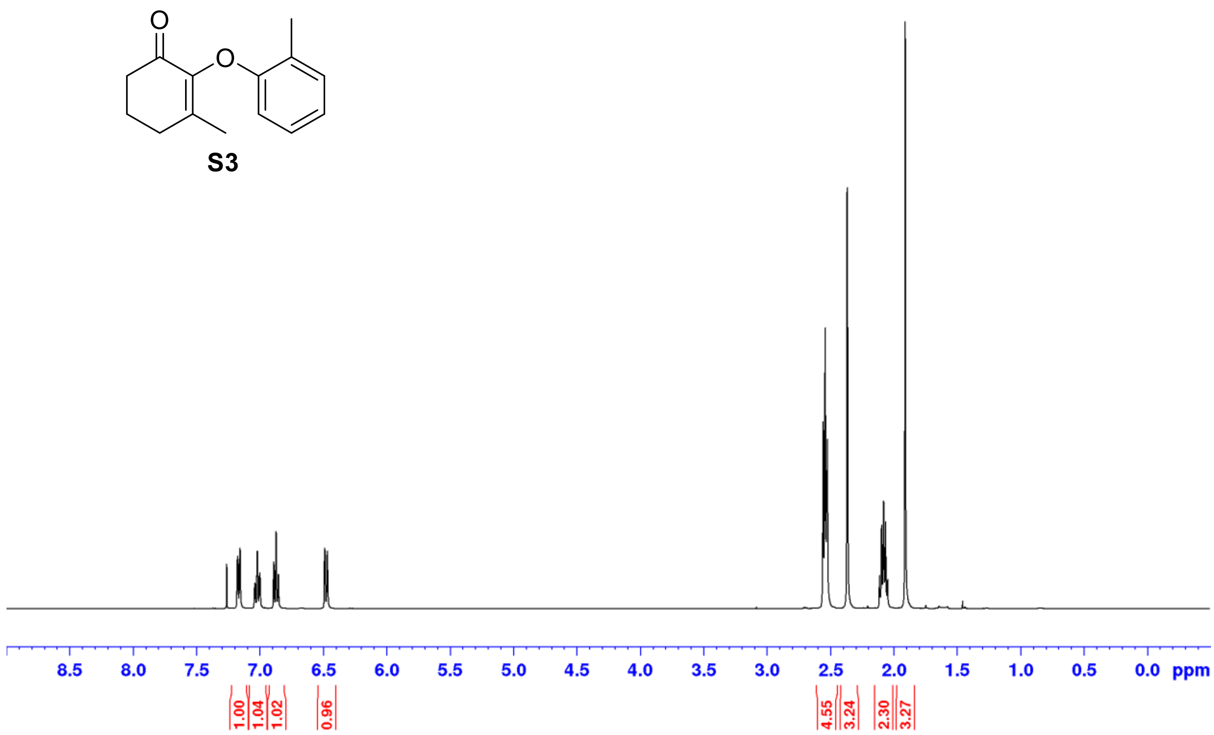

S3

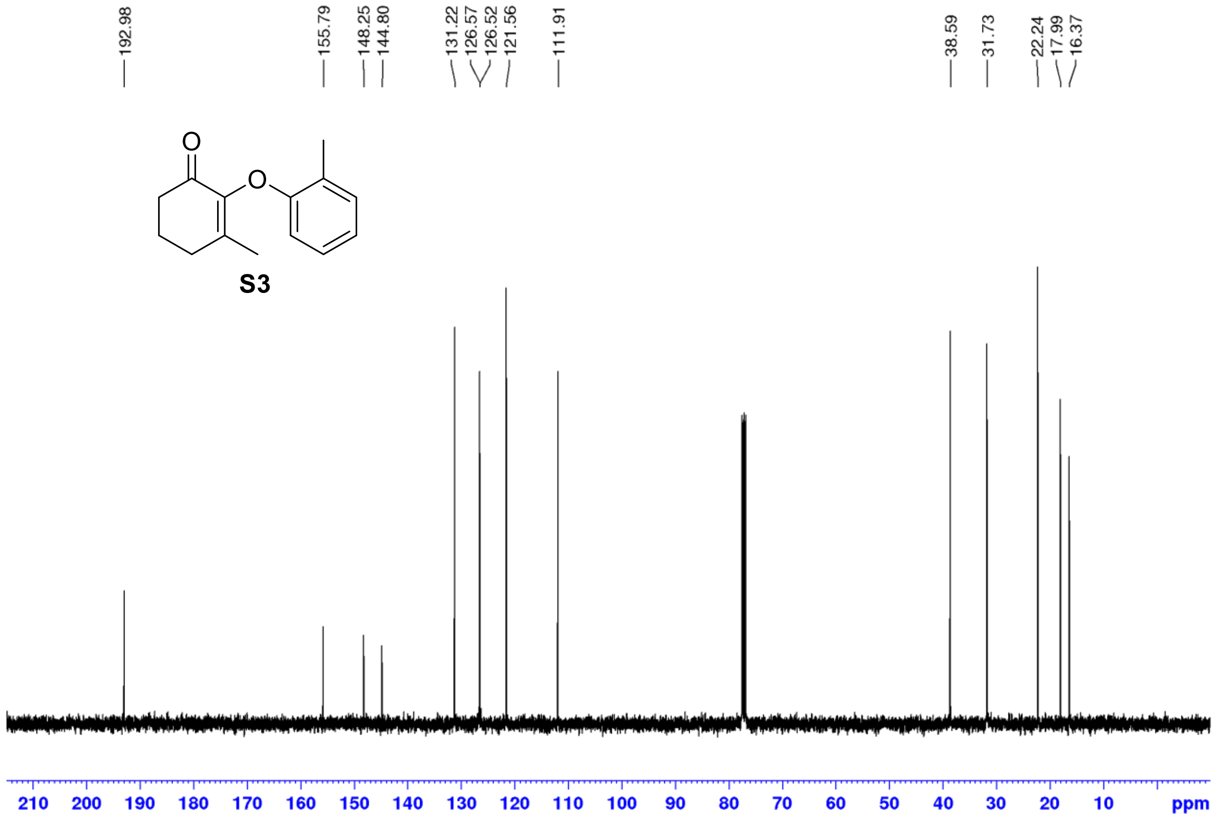

S3

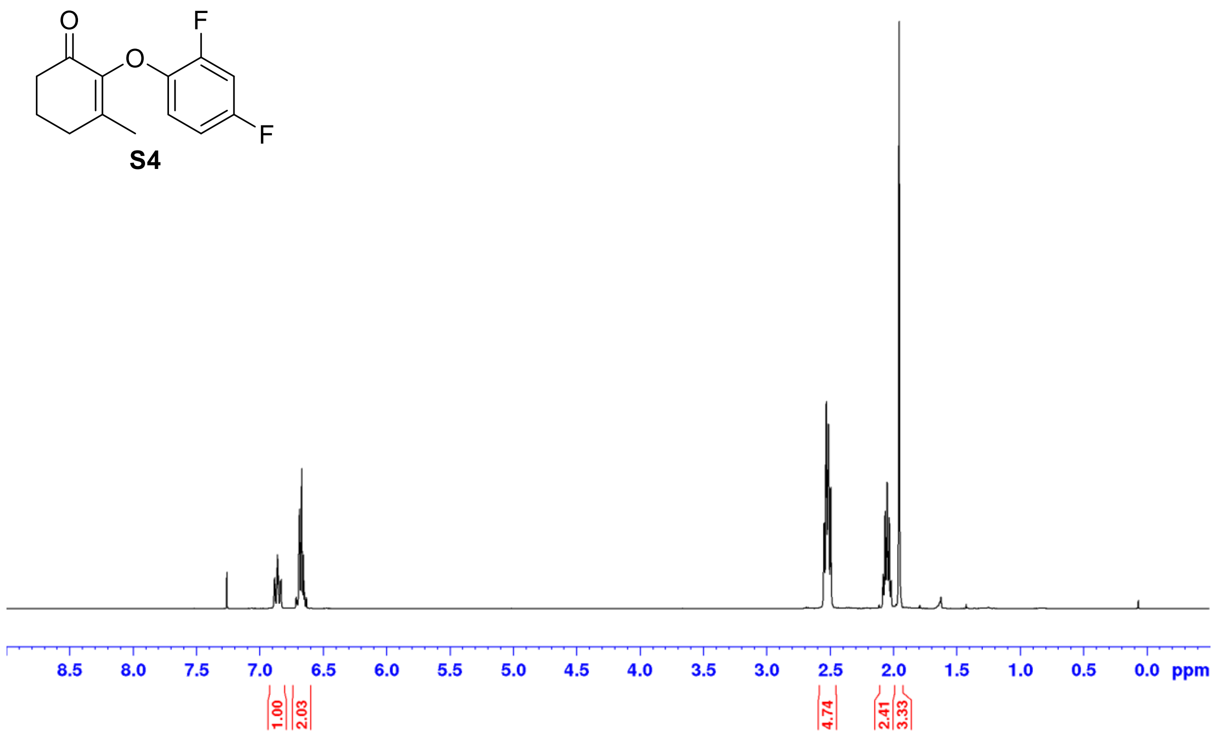

S4
8.5
8.0
7.5
7.0
6.5
6.0
5.5
5.0
4.5
4.0
3.5
3.0
2.5
2.0
1.5
1.0
0.5
0.0
ppm
1.00
2.03
4.74
2.41
3.33

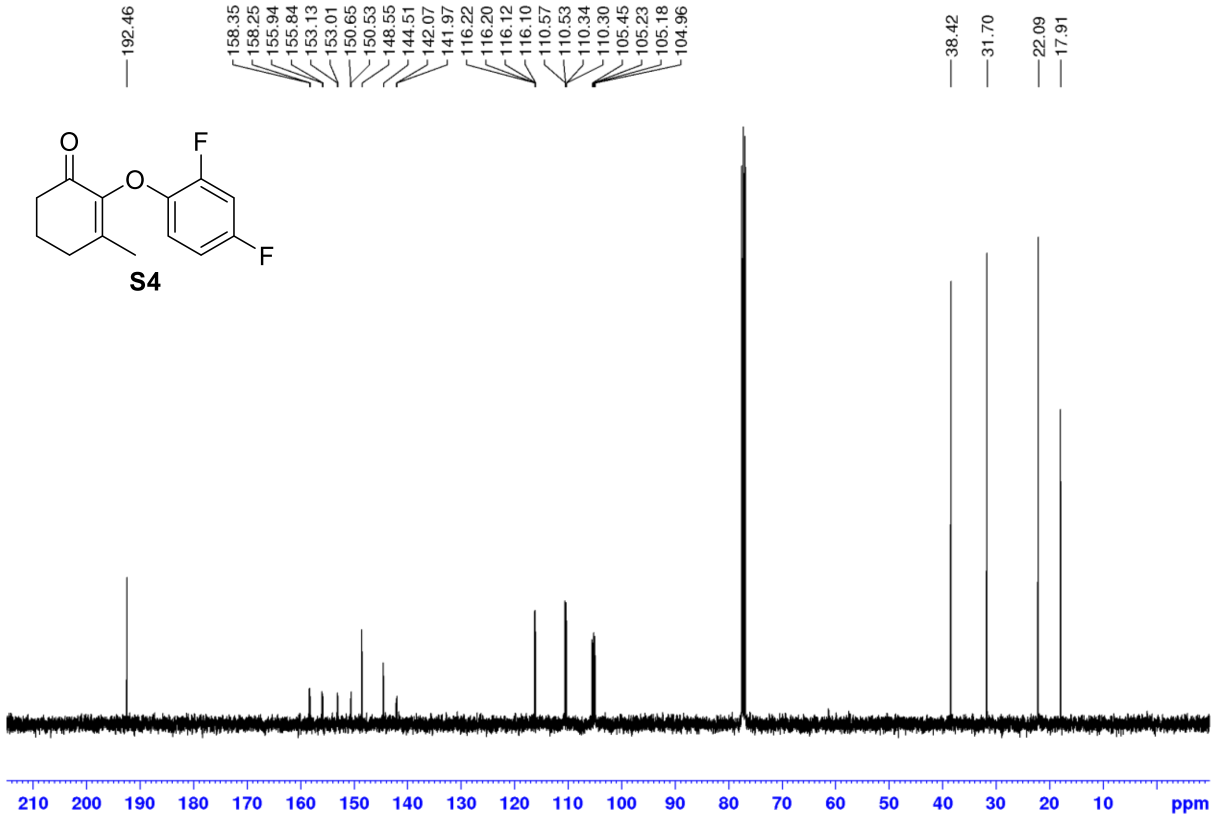

192.46
158.35
158.25
155.94
155.84
153.13
153.01
150.65
150.53
148.55
144.51
142.07
141.97
116.22
116.20
116.12
116.10
110.57
110.53
110.34
110.30
105.45
105.23
105.18
104.96
38.42
31.70
22.09
17.91
S4
210
200
190
180
170
160
150
140
130
120
110
100
90
80
70
60
50
40
30
20
10
ppm

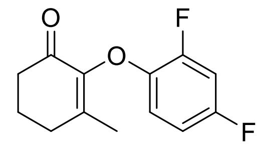
O
O
F
F

S4

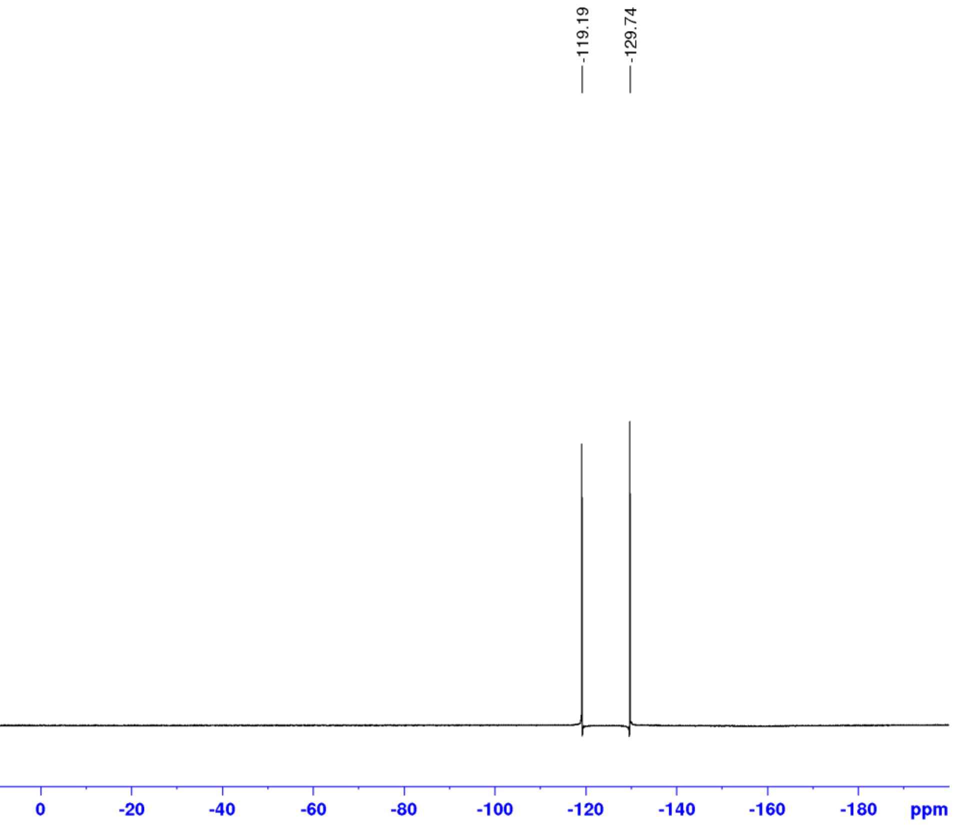
-119.19
-129.74
60
40
20
0
-20
-40
-60
-80
-100
-120
-140
-160
-180
ppm

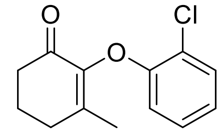
O
O
Cl

S5

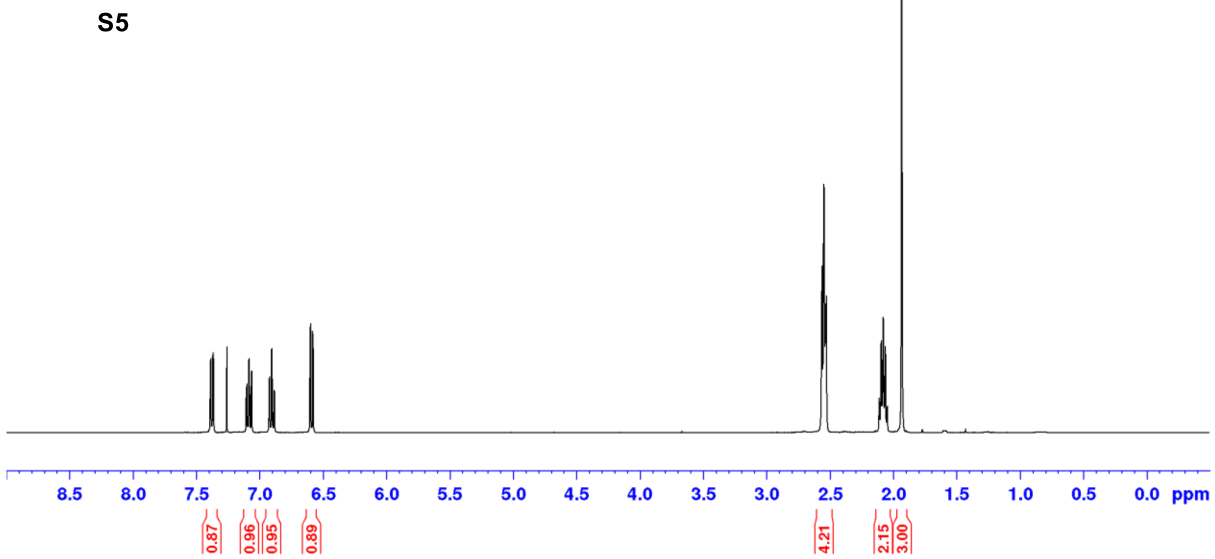
8.5
8.0
7.5
7.0
6.5
6.0
5.5
5.0
4.5
4.0
3.5
3.0
2.5
2.0
1.5
1.0
0.5
0.0
ppm
0.87
0.96
0.95
0.89
4.21
2.15
3.00

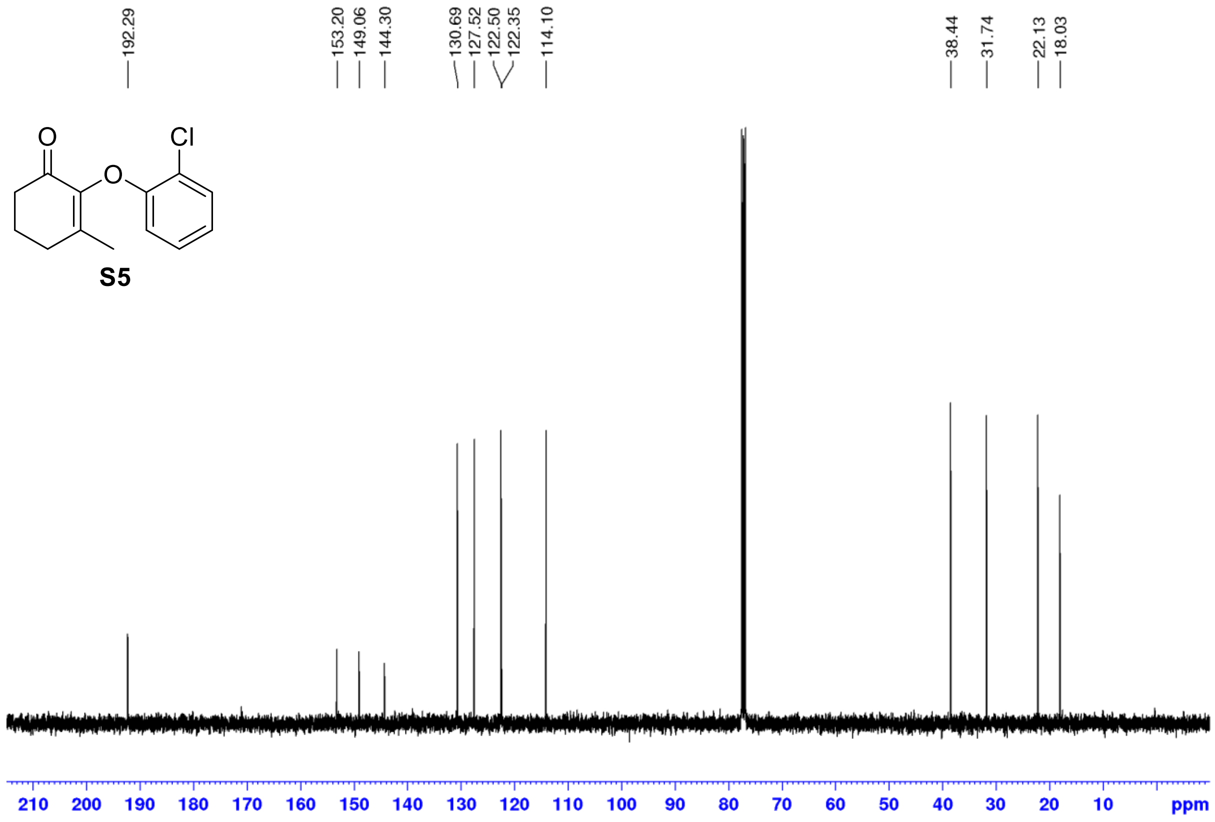
192.29
153.20
149.06
144.30
130.69
127.52
122.50
122.35
114.10
38.44
31.74
22.13
18.03
O
Cl
O
S5
210 200 190 180 170 160 150 140 130 120 110 100 90 80 70 60 50 40 30 20 10 ppm

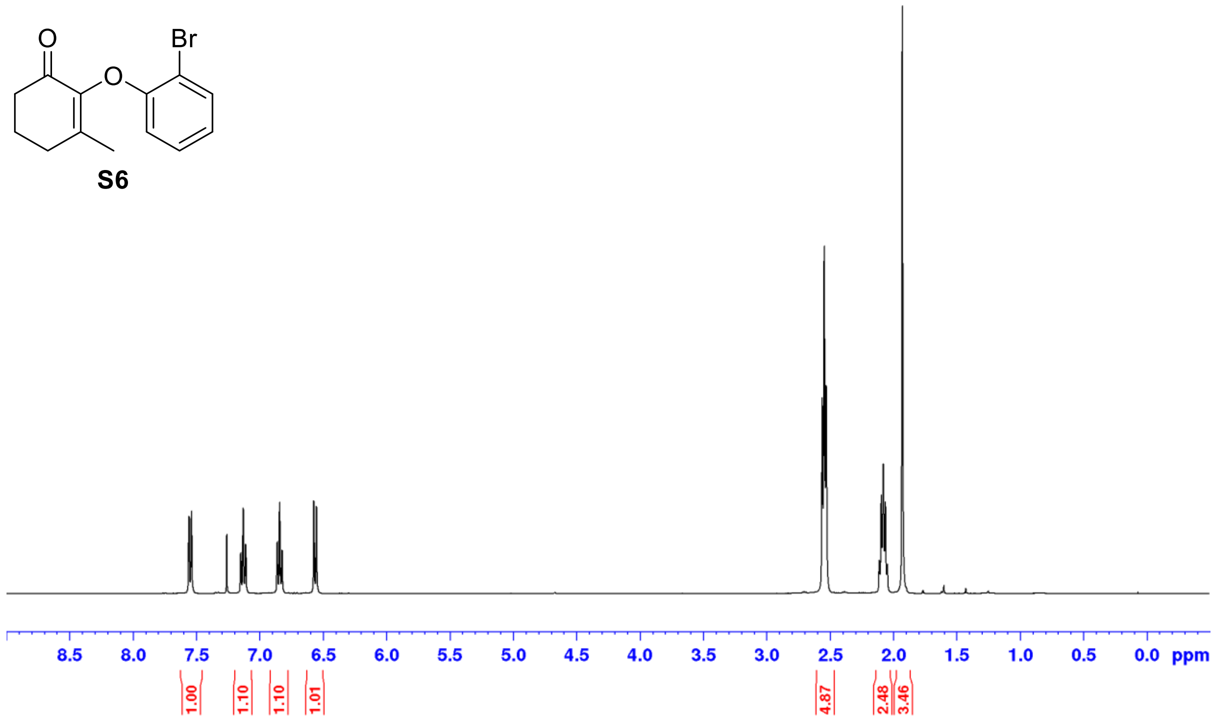
O
Br
O
S6
8.5 8.0 7.5 7.0 6.5 6.0 5.5 5.0 4.5 4.0 3.5 3.0 2.5 2.0 1.5 1.0 0.5 0.0 ppm
1.00
1.10
1.10
1.01
4.87
2.48
3.46

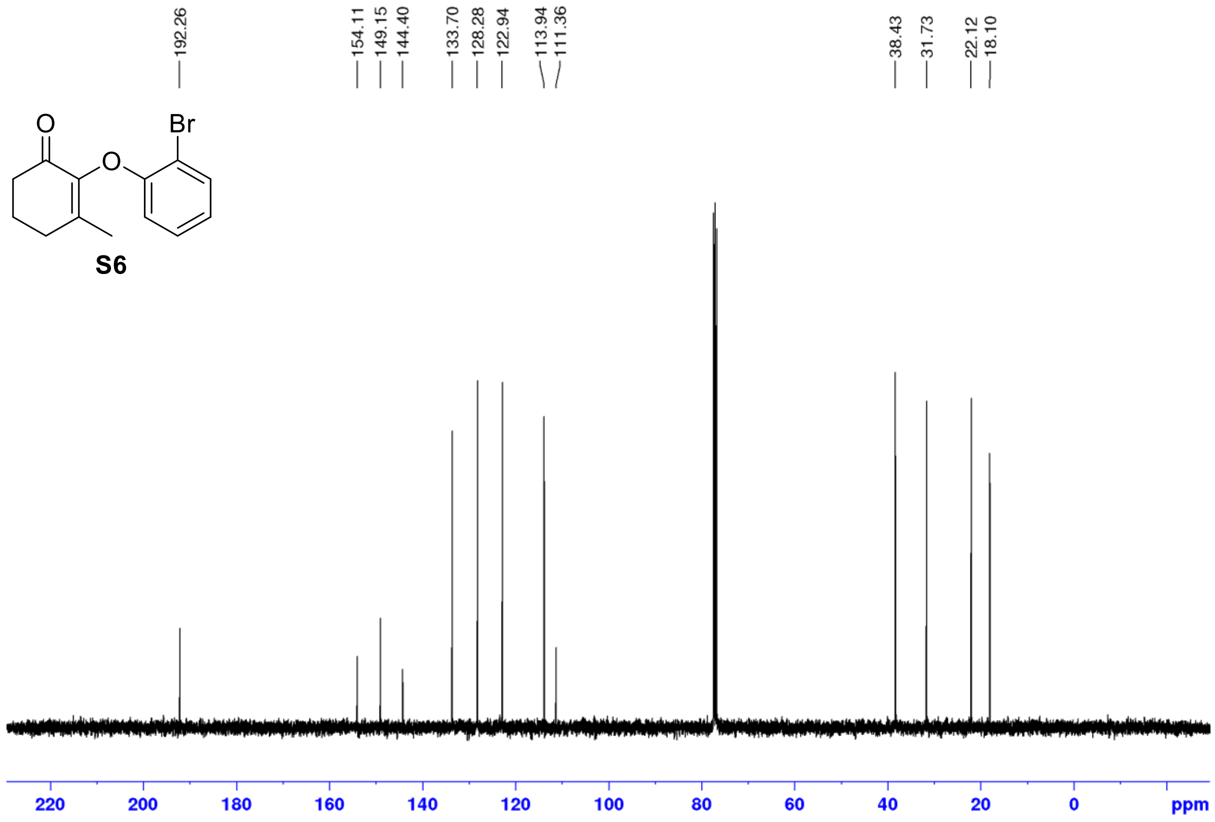
192.26
154.11
149.15
144.40
133.70
128.28
122.94
113.94
111.36
38.43
31.73
22.12
18.10
O
Br
O
S6
220
200
180
160
140
120
100
80
60
40
20
0
ppm

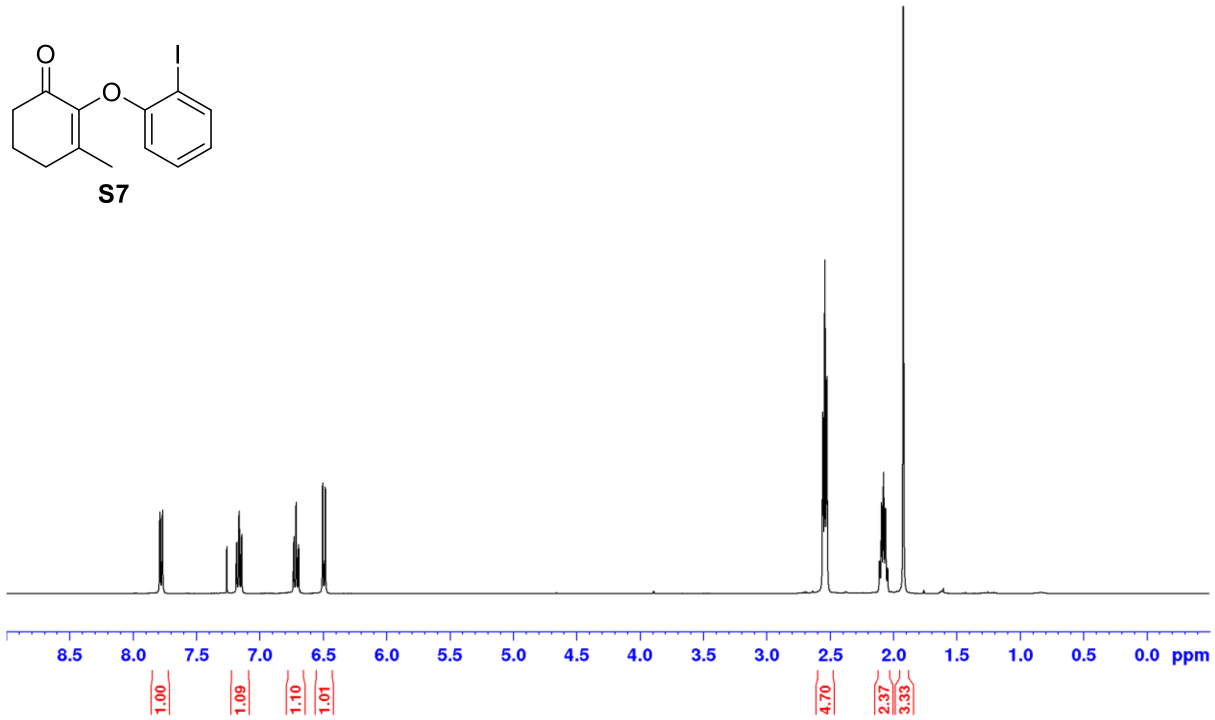
O
I
O
S7
8.5
8.0
7.5
7.0
6.5
6.0
5.5
5.0
4.5
4.0
3.5
3.0
2.5
2.0
1.5
1.0
0.5
0.0
ppm
1.00
1.09
1.10
1.01
4.70
2.37
3.33

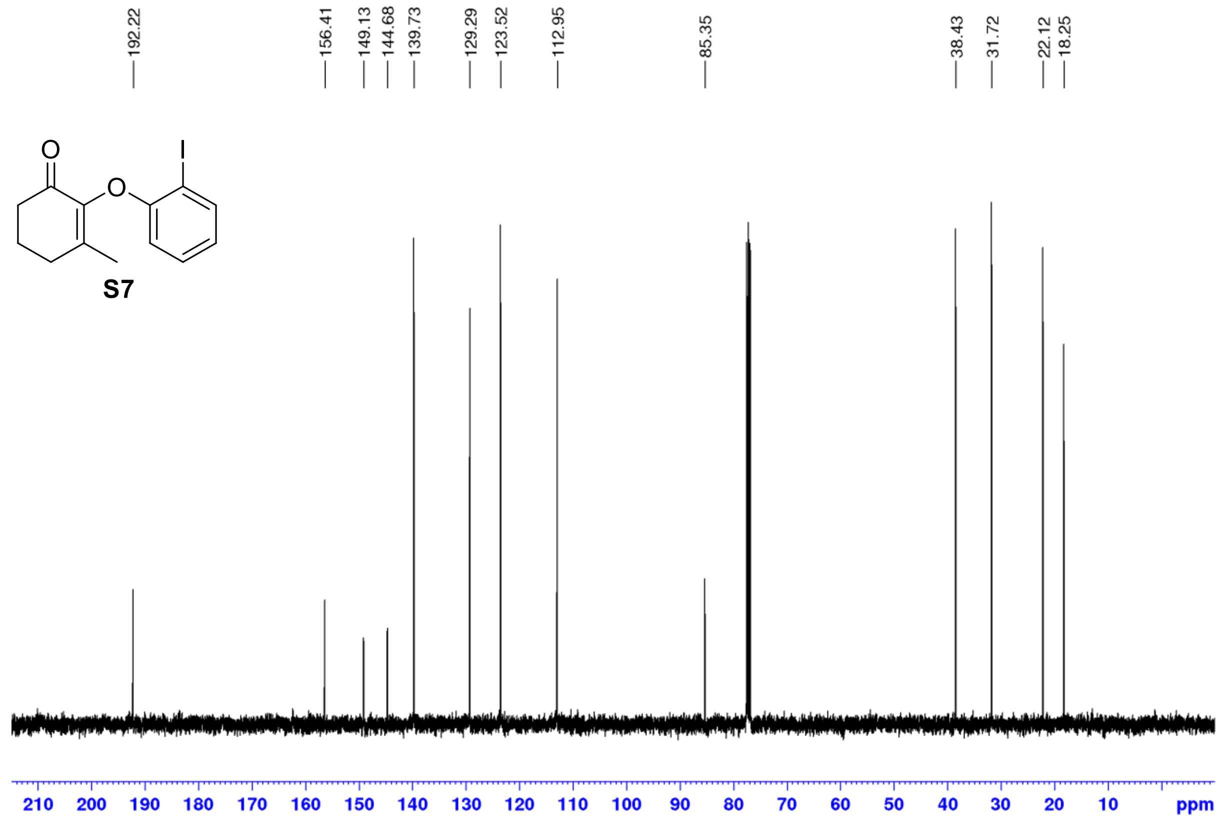
192.22
156.41
149.13
144.68
139.73
129.29
123.52
112.95
85.35
38.43
31.72
22.12
18.25
O
O
I
S7
210 200 190 180 170 160 150 140 130 120 110 100 90 80 70 60 50 40 30 20 10 ppm

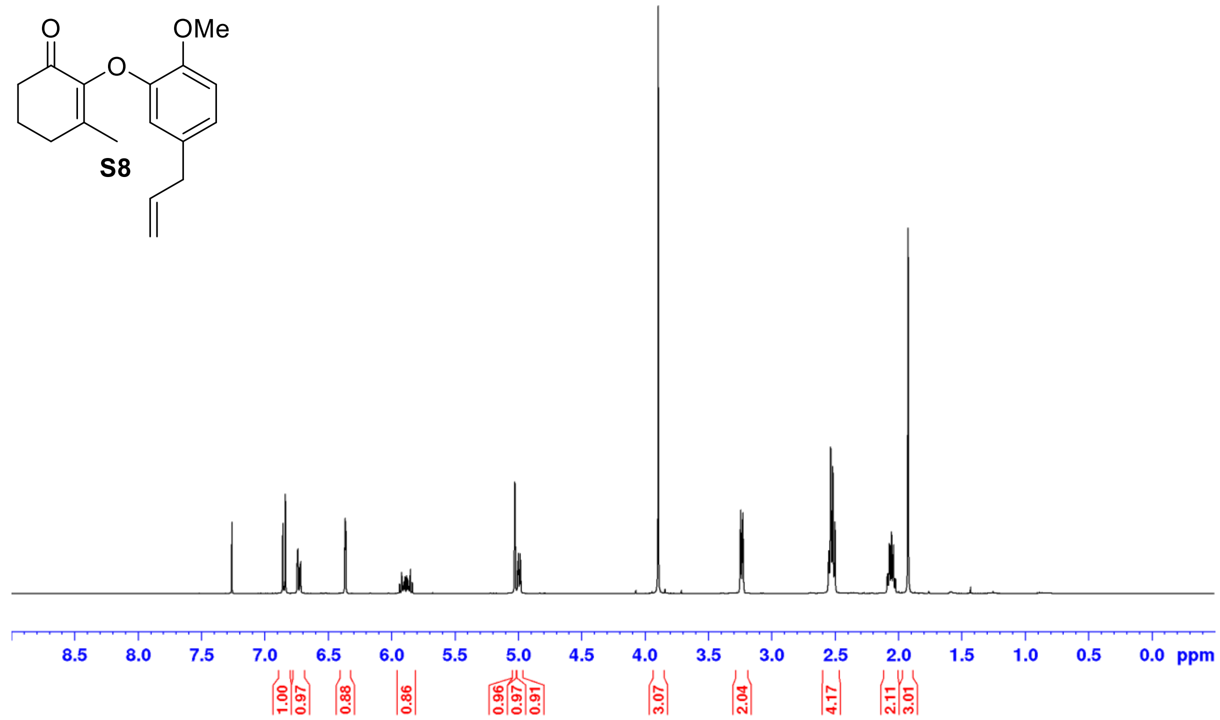
O
O
OMe
S8
8.5 8.0 7.5 7.0 6.5 6.0 5.5 5.0 4.5 4.0 3.5 3.0 2.5 2.0 1.5 1.0 0.5 0.0 ppm
1.00
0.97
0.88
0.86
0.96
0.97
0.91
3.07
2.04
4.17
2.11
3.01

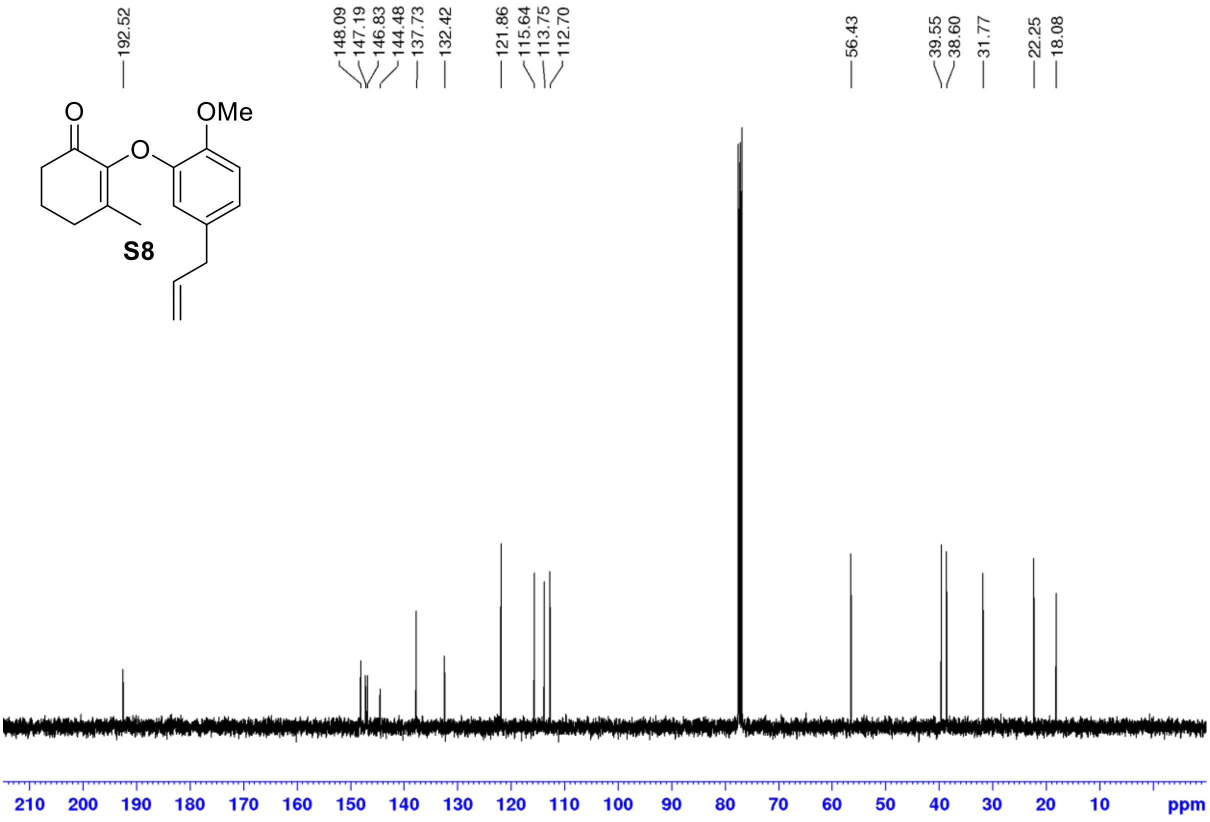
192.52
148.09
147.19
146.83
144.48
137.73
132.42
121.86
115.64
113.75
112.70
56.43
39.55
38.60
31.77
22.25
18.08
O
OMe
O
S8
210 200 190 180 170 160 150 140 130 120 110 100 90 80 70 60 50 40 30 20 10 ppm

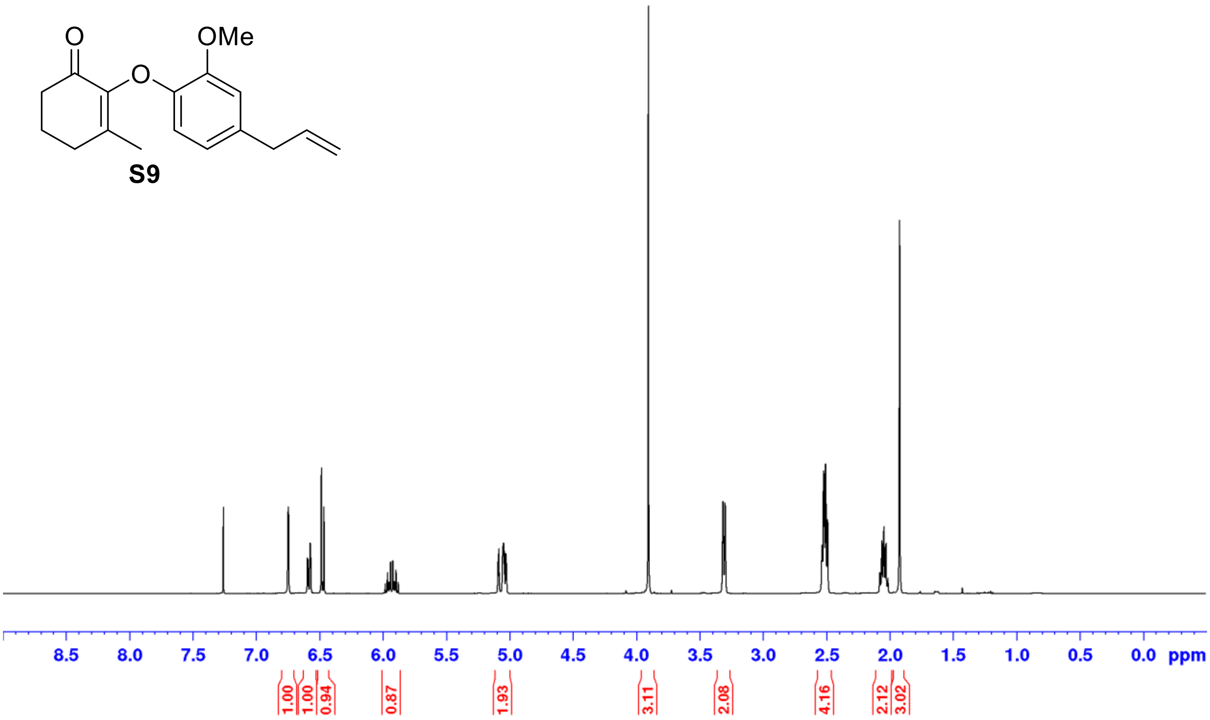
O
OMe
O
S9
8.5 8.0 7.5 7.0 6.5 6.0 5.5 5.0 4.5 4.0 3.5 3.0 2.5 2.0 1.5 1.0 0.5 0.0 ppm
1.00
1.00
0.94
0.87
1.93
3.11
2.08
4.16
2.12
3.02

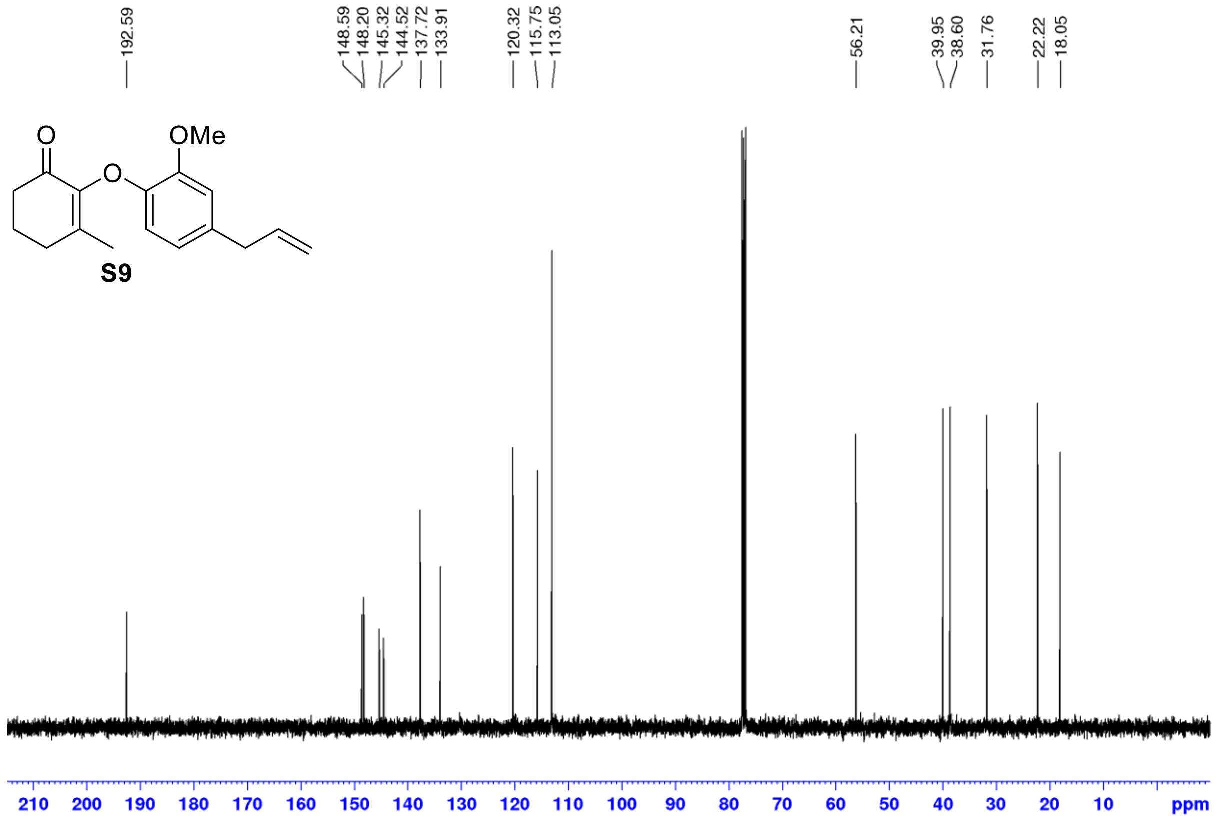
192.59
148.59
148.20
145.32
144.52
137.72
133.91
120.32
115.75
113.05
56.21
39.95
38.60
31.76
22.22
18.05
OMe
O
S9
210 200 190 180 170 160 150 140 130 120 110 100 90 80 70 60 50 40 30 20 10 ppm

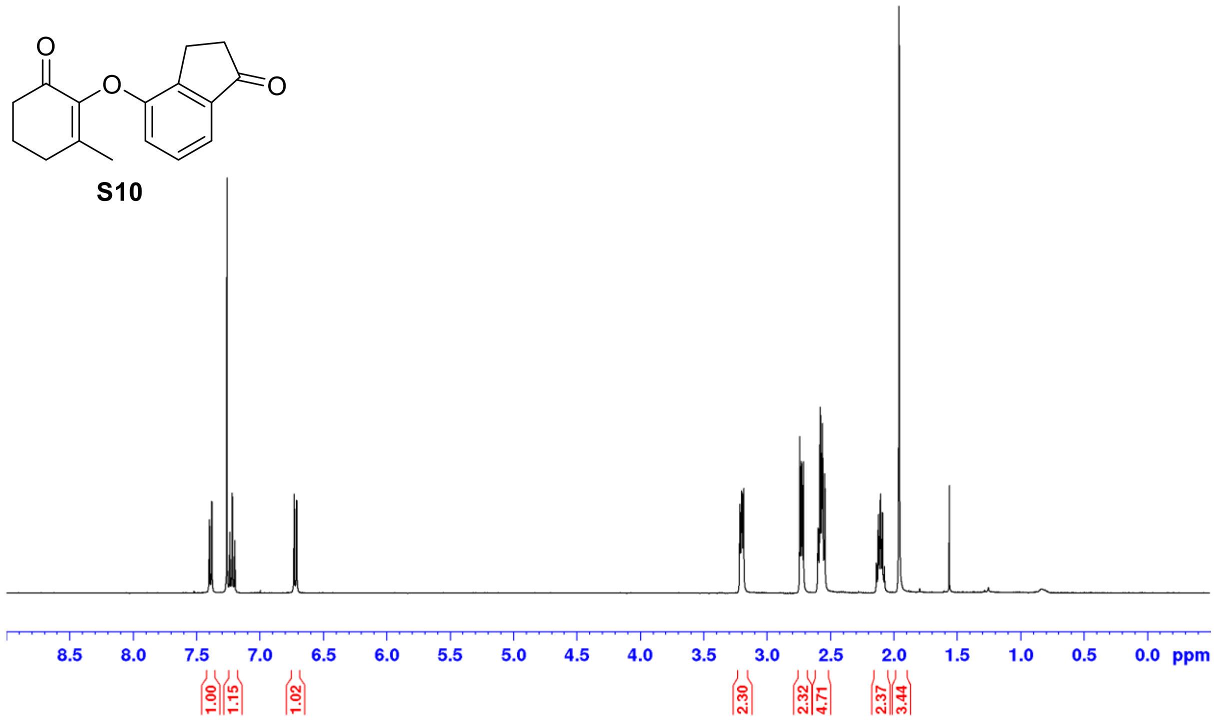
O
S10
8.5 8.0 7.5 7.0 6.5 6.0 5.5 5.0 4.5 4.0 3.5 3.0 2.5 2.0 1.5 1.0 0.5 0.0 ppm
1.00
1.15
1.02
2.30
2.32
4.71
2.37
3.44

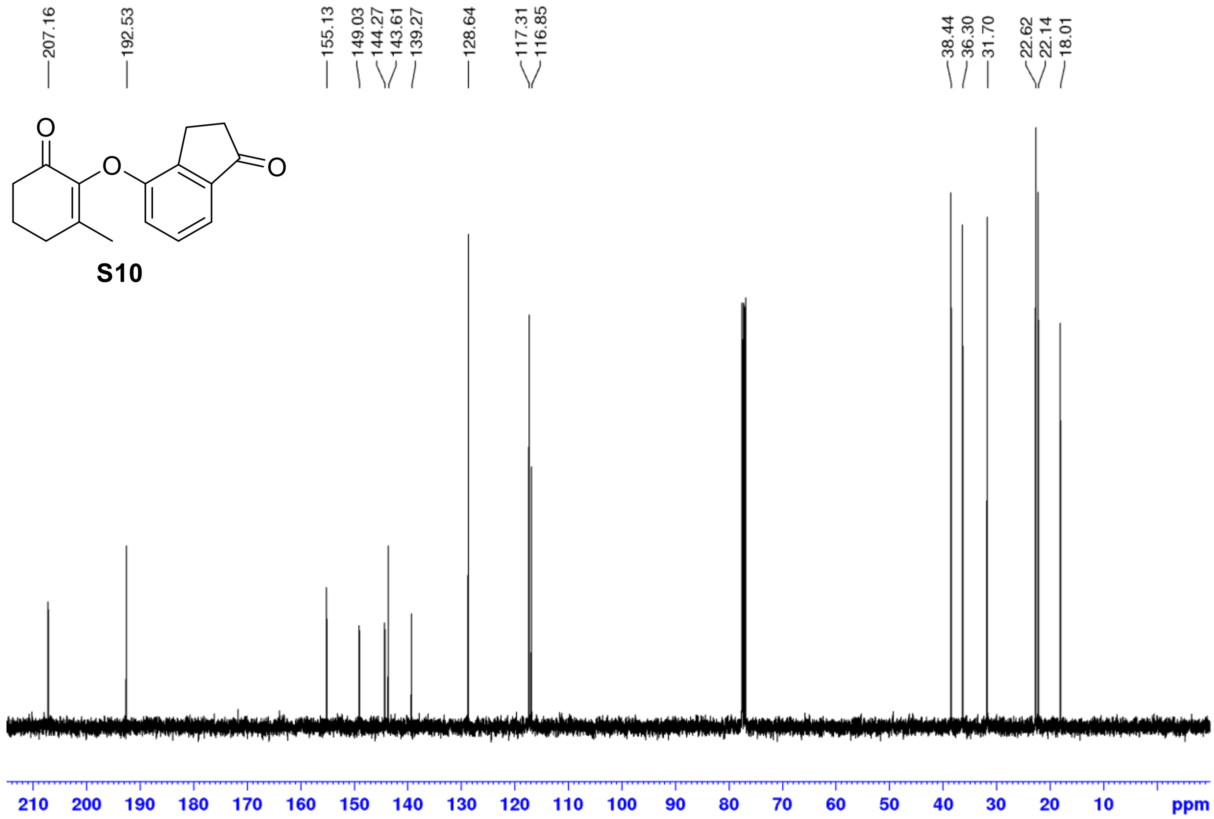
207.16
192.53
155.13
149.03
144.27
143.61
139.27
128.64
117.31
116.85
38.44
36.30
31.70
22.62
22.14
18.01
S10
ppm

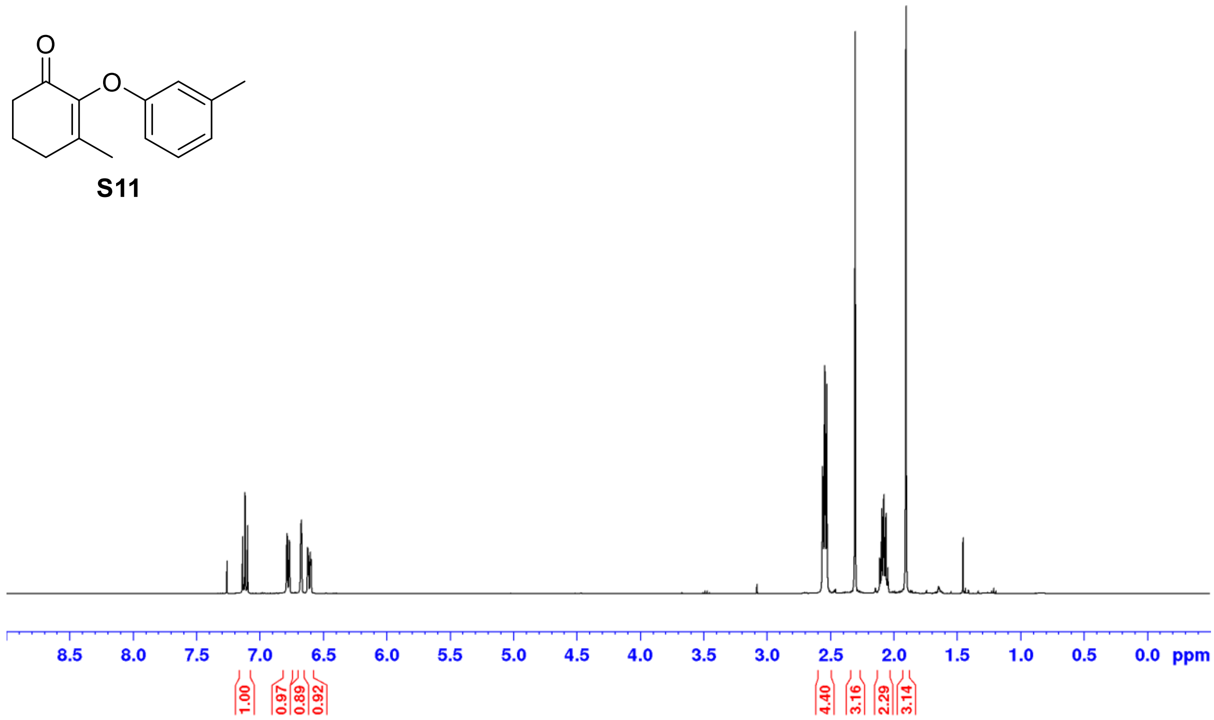
S11
ppm
1.00
0.97
0.89
0.92
4.40
3.16
2.29
3.14

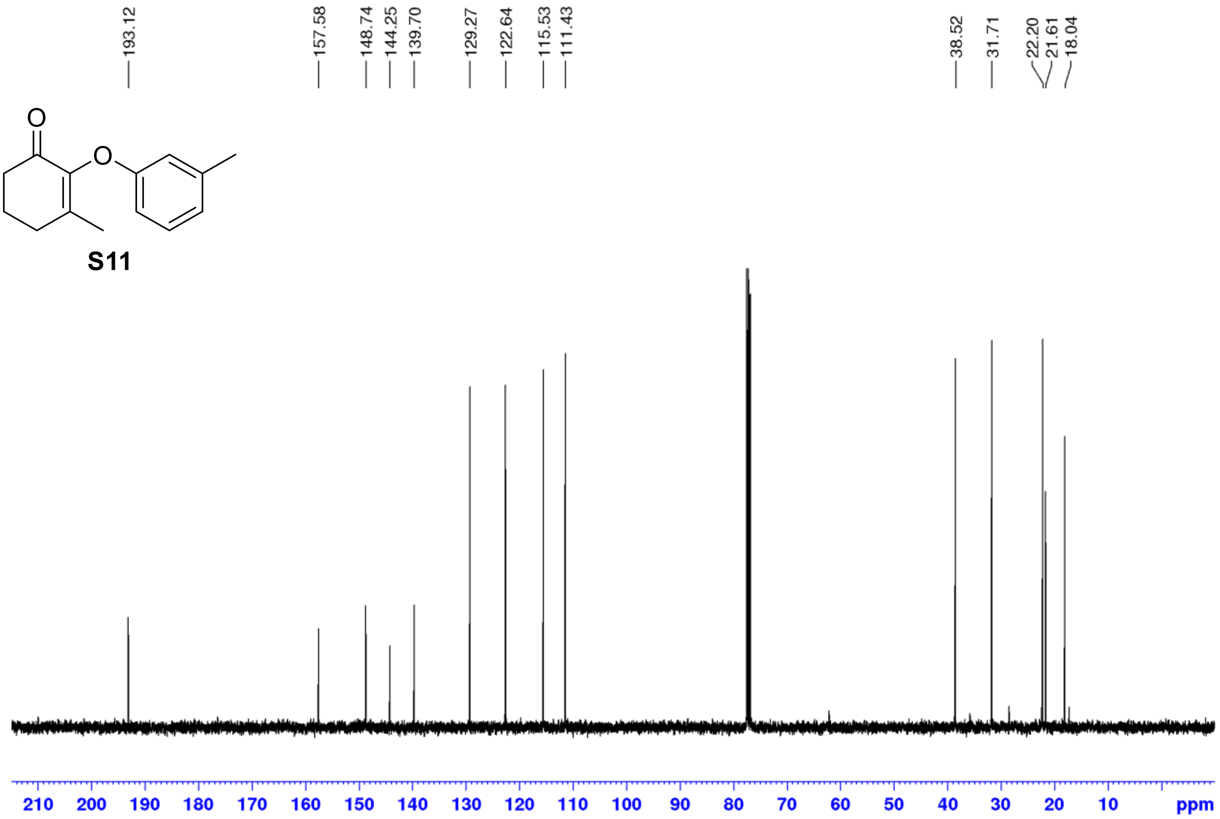
S11
193.12
157.58
148.74
144.25
139.70
129.27
122.64
115.53
111.43
38.52
31.71
22.20
21.61
18.04
210 200 190 180 170 160 150 140 130 120 110 100 90 80 70 60 50 40 30 20 10 ppm

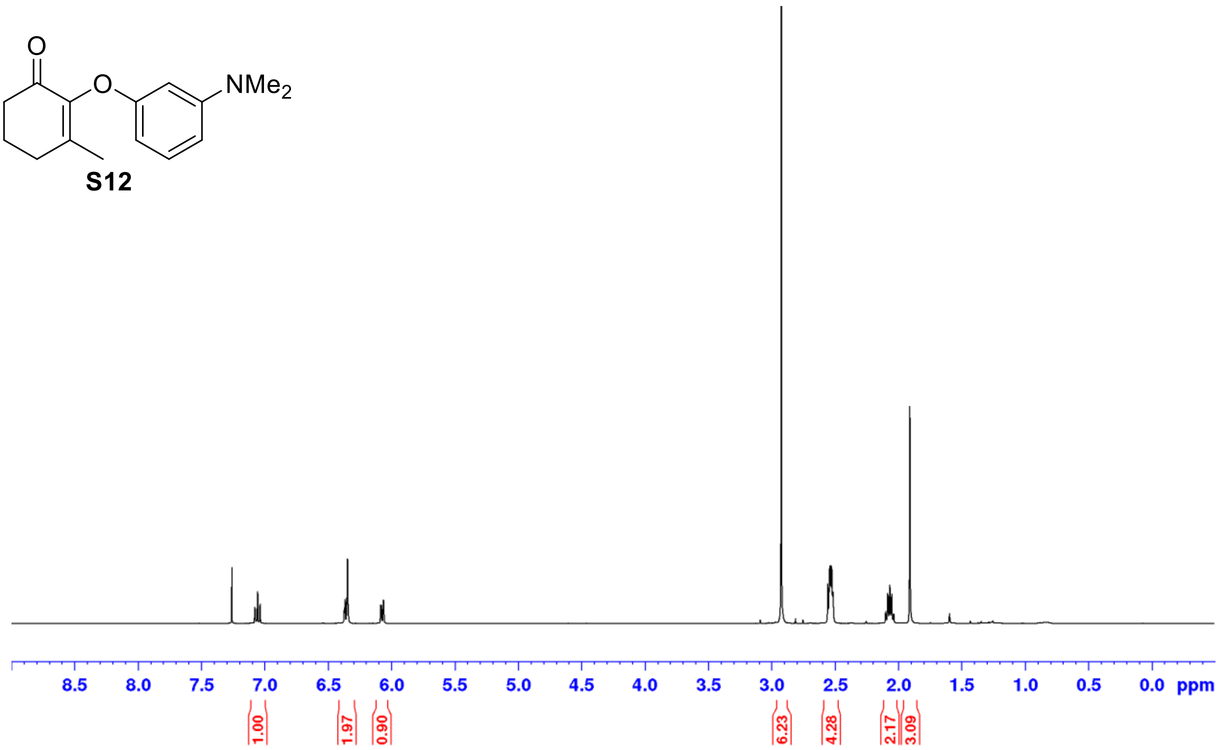
NMe2
S12
8.5 8.0 7.5 7.0 6.5 6.0 5.5 5.0 4.5 4.0 3.5 3.0 2.5 2.0 1.5 1.0 0.5 0.0 ppm
1.00
1.97
0.90
6.23
4.28
2.17
3.09

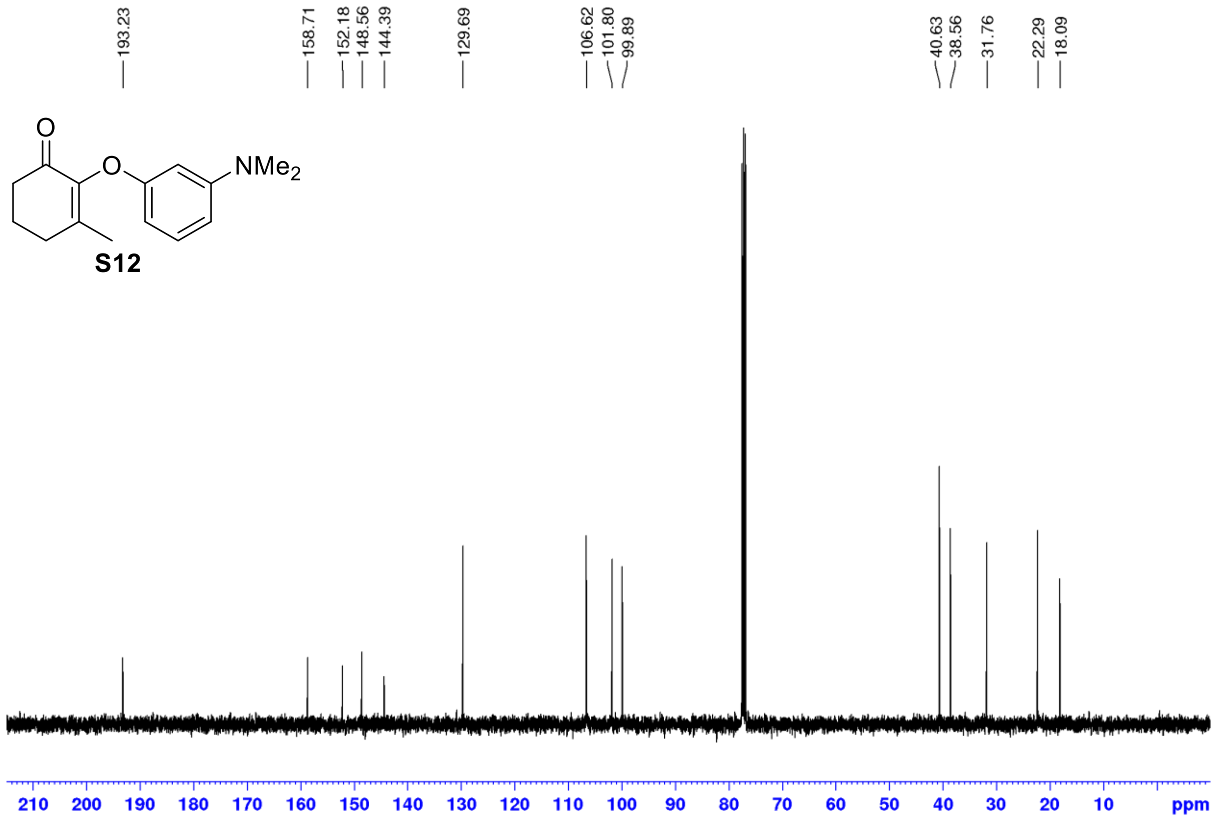

193.23
158.71
152.18
148.56
144.39
129.69
106.62
101.80
99.89
40.63
38.56
31.76
22.29
18.09
NMe2
210 200 190 180 170 160 150 140 130 120 110 100 90 80 70 60 50 40 30 20 10 ppm

S12

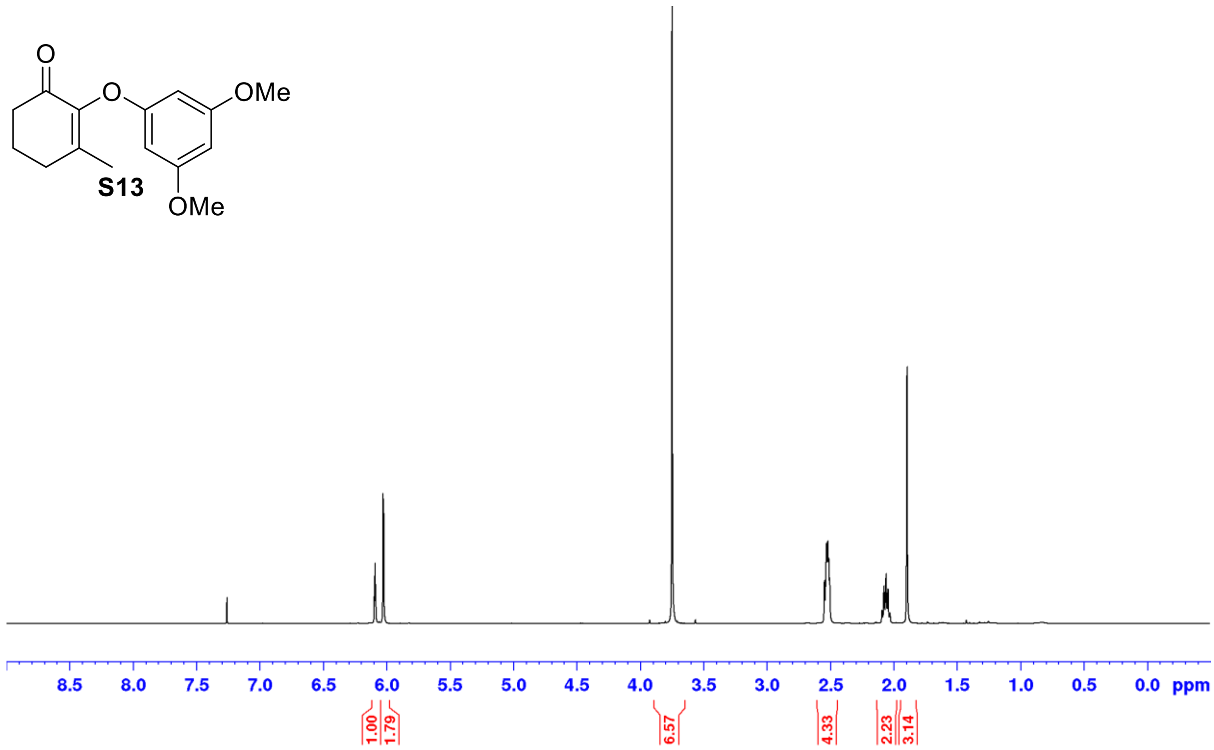

OMe
OMe
8.5 8.0 7.5 7.0 6.5 6.0 5.5 5.0 4.5 4.0 3.5 3.0 2.5 2.0 1.5 1.0 0.5 0.0 ppm
1.00
1.79
6.57
4.33
2.23
3.14

S13

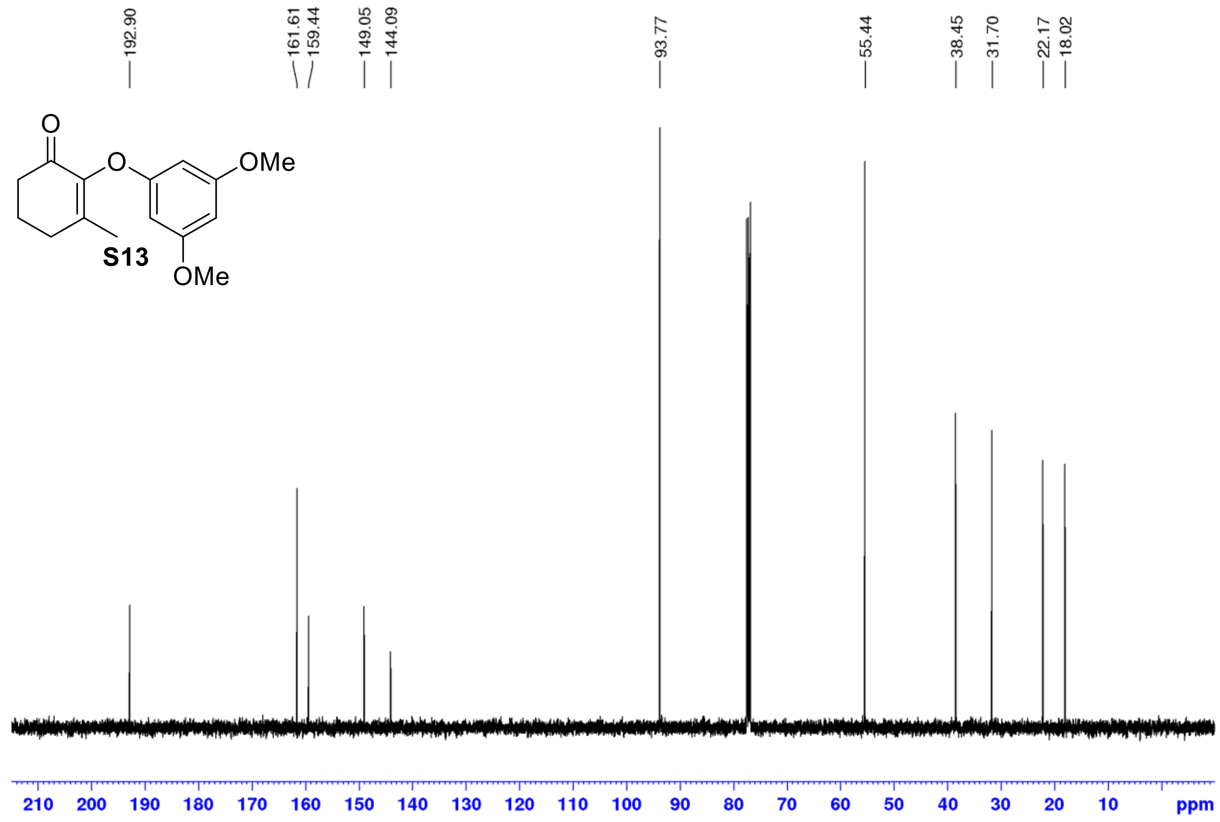
192.90
161.61
159.44
149.05
144.09
93.77
55.44
38.45
31.70
22.17
18.02
OMe
OMe
O
O
S13
210 200 190 180 170 160 150 140 130 120 110 100 90 80 70 60 50 40 30 20 10 ppm

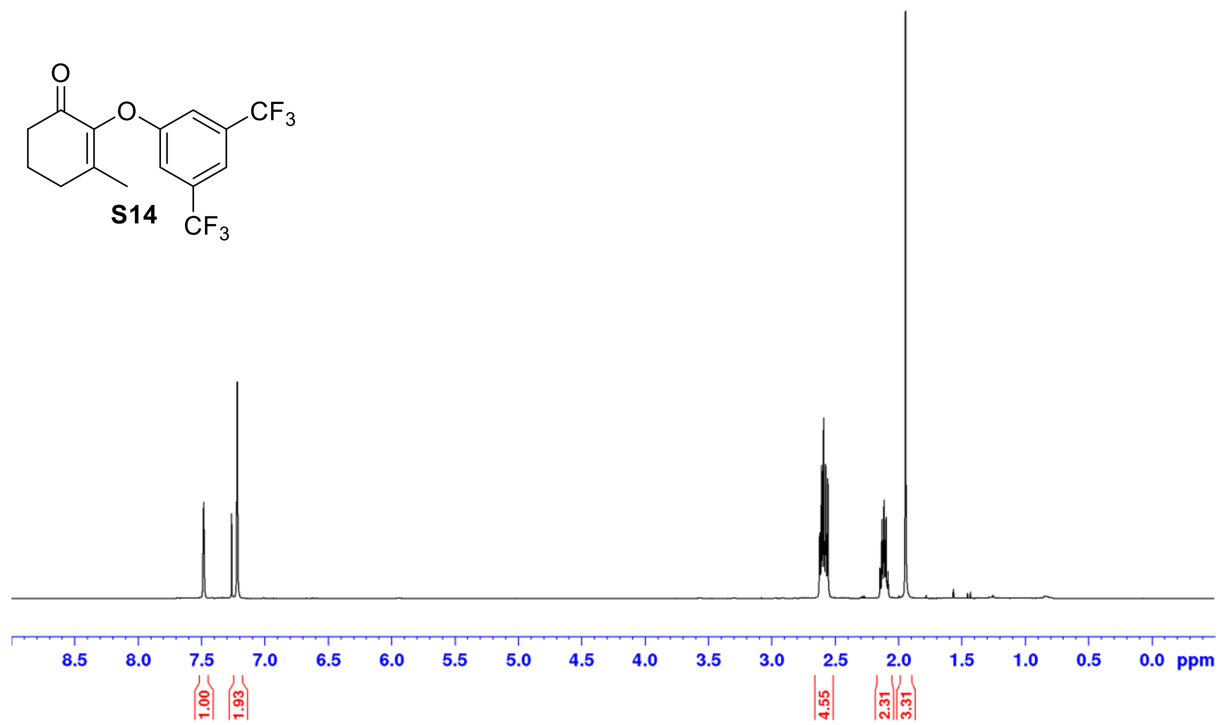
O
O
CF_3
CF_3
S14
8.5 8.0 7.5 7.0 6.5 6.0 5.5 5.0 4.5 4.0 3.5 3.0 2.5 2.0 1.5 1.0 0.5 0.0 ppm
1.00
1.93
4.55
2.31
3.31

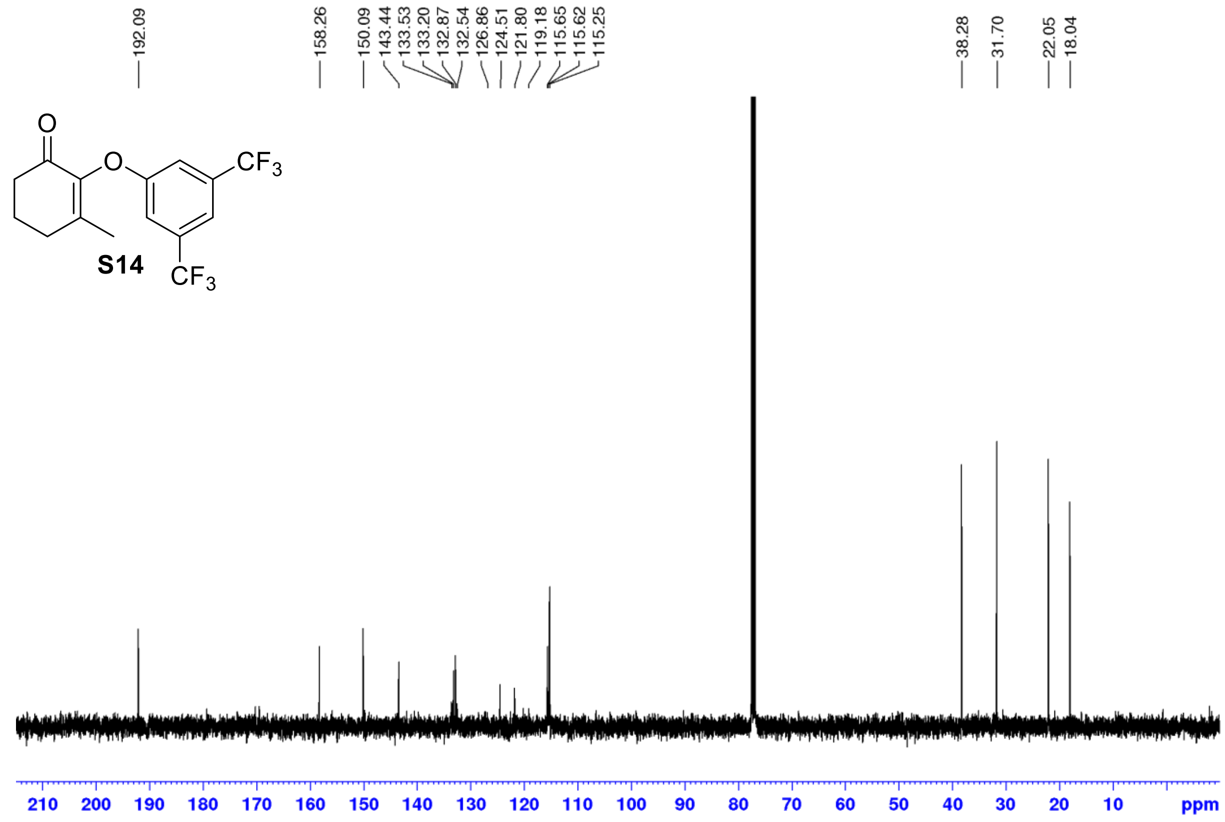
192.09
158.26
150.09
143.44
133.53
133.20
132.87
132.54
126.86
124.51
121.80
119.18
115.65
115.62
115.25
38.28
31.70
22.05
18.04
O
O
CF3
CF3
S14
210 200 190 180 170 160 150 140 130 120 110 100 90 80 70 60 50 40 30 20 10 ppm

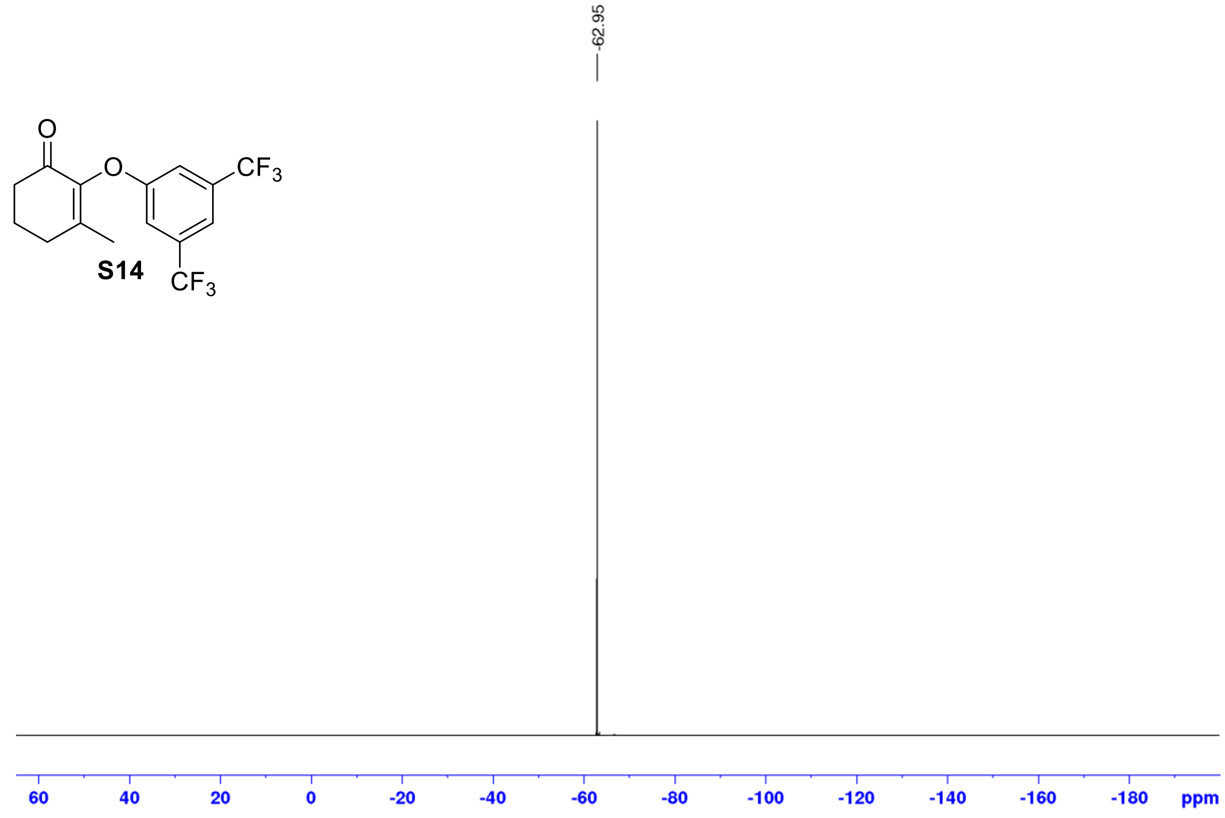
-62.95
O
O
CF3
CF3
S14
60 40 20 0 -20 -40 -60 -80 -100 -120 -140 -160 -180 ppm

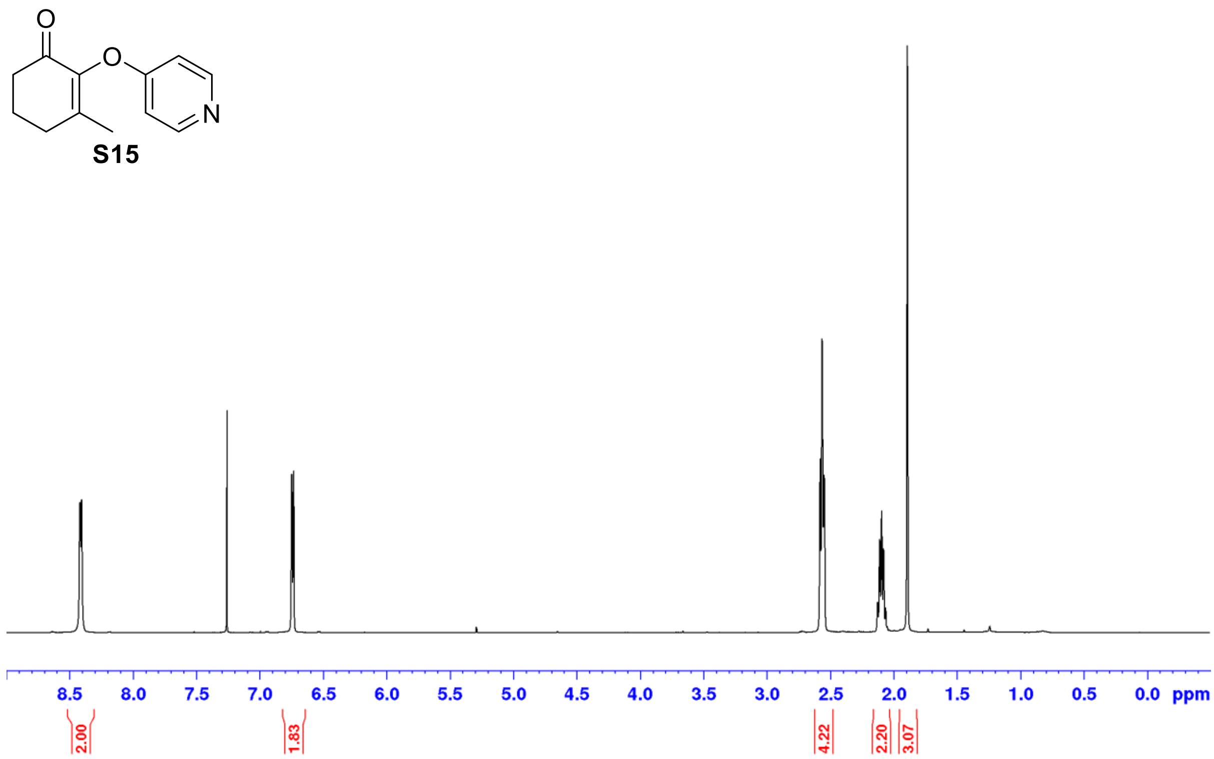
S15
8.5
8.0
7.5
7.0
6.5
6.0
5.5
5.0
4.5
4.0
3.5
3.0
2.5
2.0
1.5
1.0
0.5
0.0
ppm
2.00
1.83
4.22
2.20
3.07

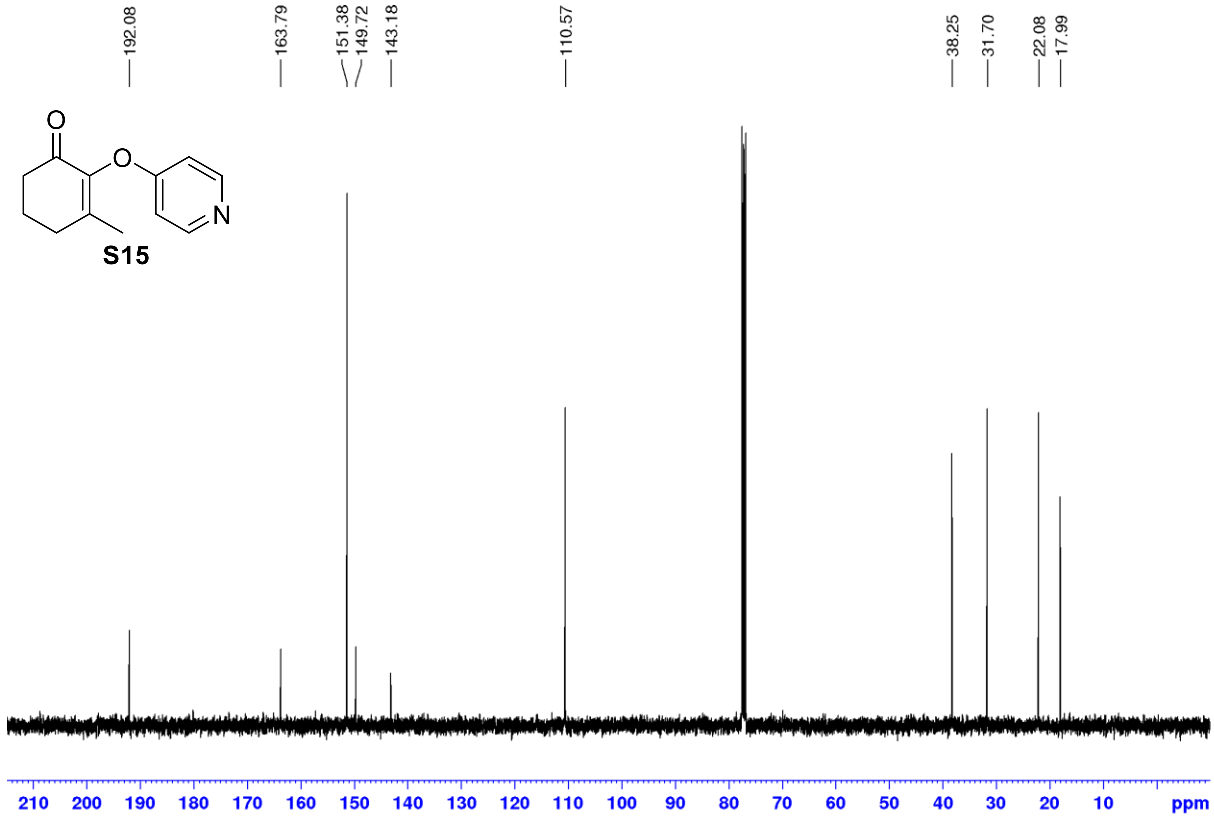
192.08
163.79
151.38
149.72
143.18
110.57
38.25
31.70
22.08
17.99
S15
210
200
190
180
170
160
150
140
130
120
110
100
90
80
70
60
50
40
30
20
10
ppm

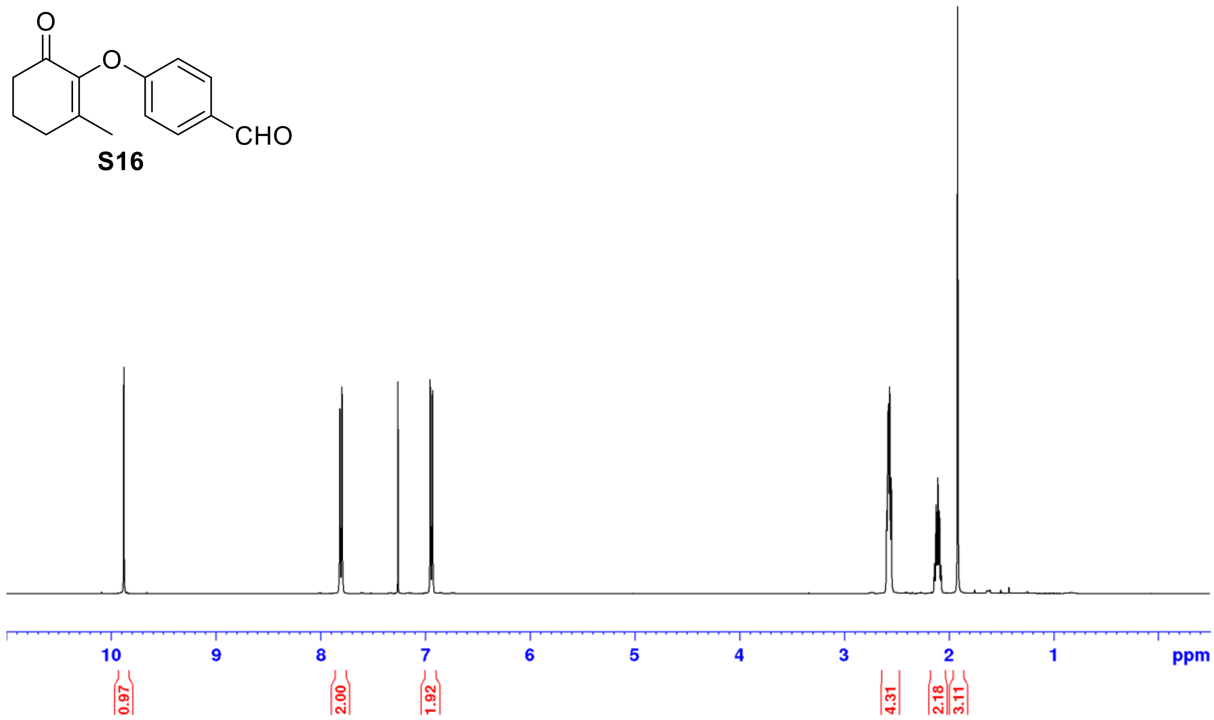
CHO
S16
10
9
8
7
6
5
4
3
2
1
ppm
0.97
2.00
1.92
4.31
2.18
3.11

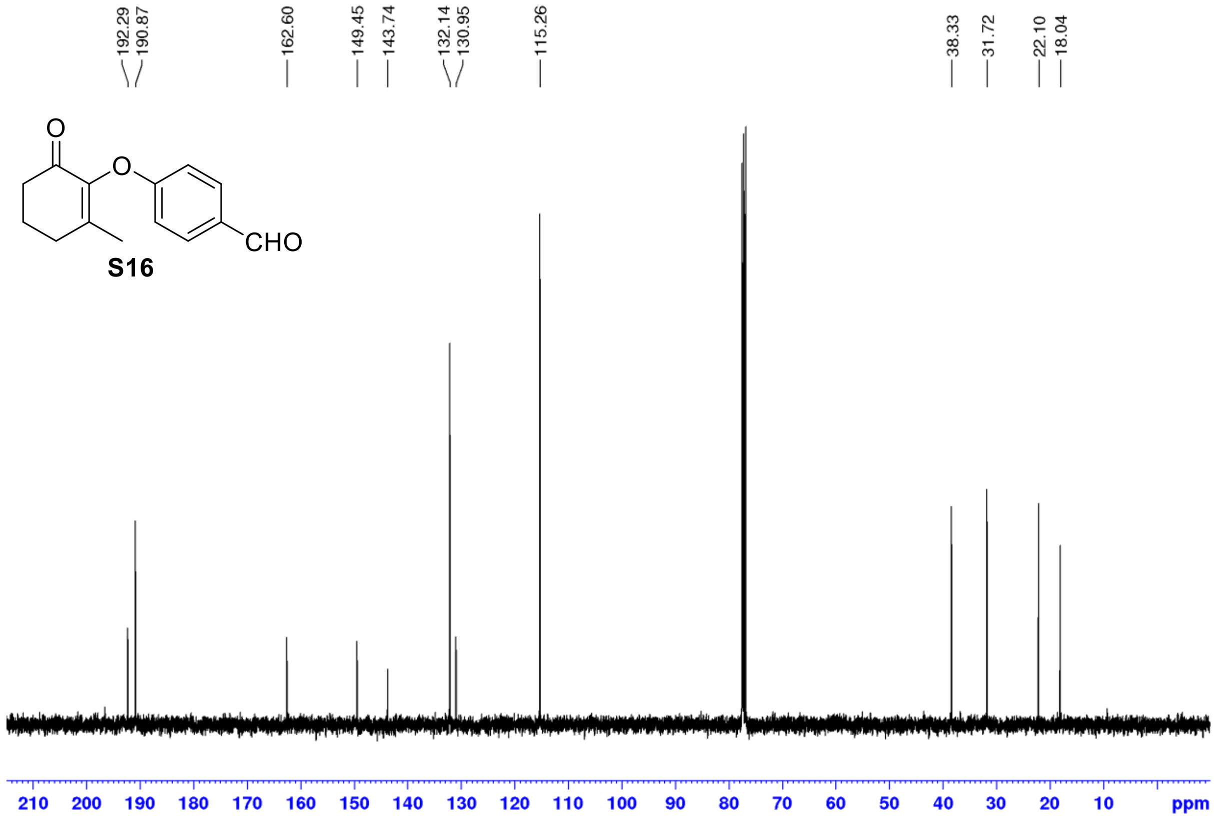
192.29
190.87
162.60
149.45
143.74
132.14
130.95
115.26
38.33
31.72
22.10
18.04
CHO
S16
210
200
190
180
170
160
150
140
130
120
110
100
90
80
70
60
50
40
30
20
10
ppm

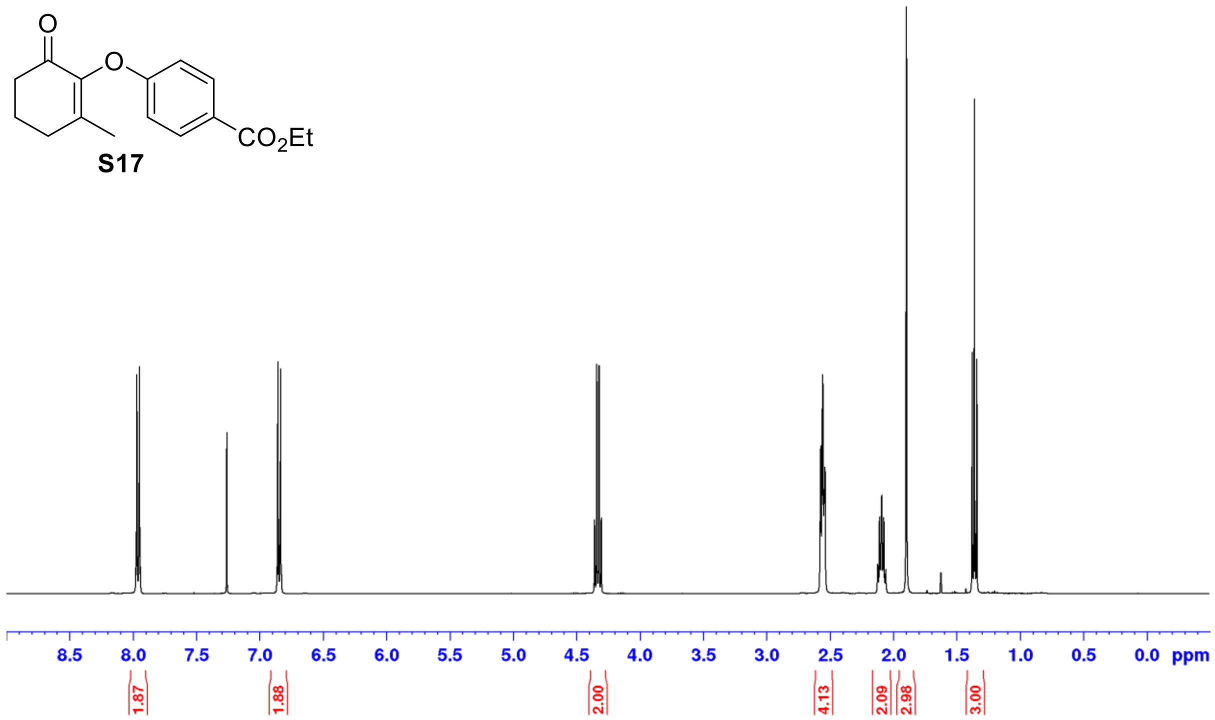
O
O
CO2Et
S17
8.5
8.0
7.5
7.0
6.5
6.0
5.5
5.0
4.5
4.0
3.5
3.0
2.5
2.0
1.5
1.0
0.5
0.0
ppm
1.87
1.88
2.00
4.13
2.09
2.98
3.00

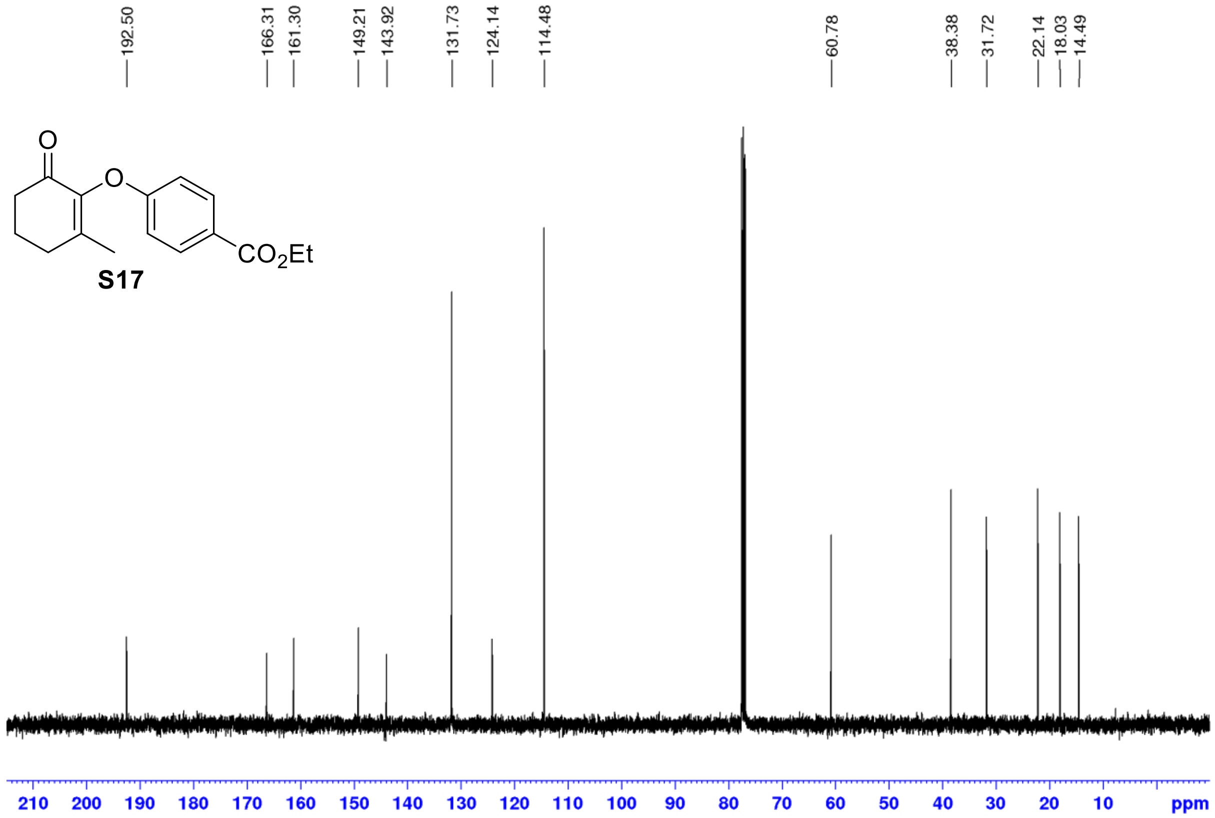
192.50
166.31
161.30
149.21
143.92
131.73
124.14
114.48
60.78
38.38
31.72
22.14
18.03
14.49
O
O
CO2Et
S17
210
200
190
180
170
160
150
140
130
120
110
100
90
80
70
60
50
40
30
20
10
ppm

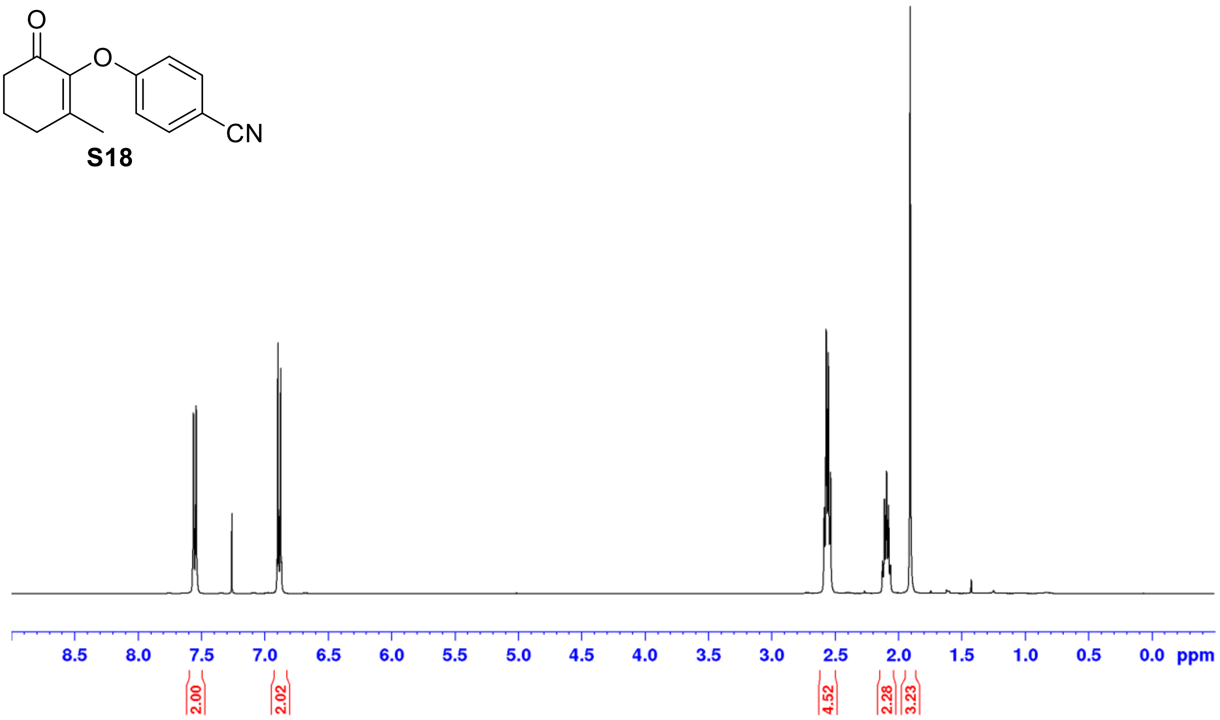
O
O
CN
S18
8.5
8.0
7.5
7.0
6.5
6.0
5.5
5.0
4.5
4.0
3.5
3.0
2.5
2.0
1.5
1.0
0.5
0.0
ppm
2.00
2.02
4.52
2.28
3.23

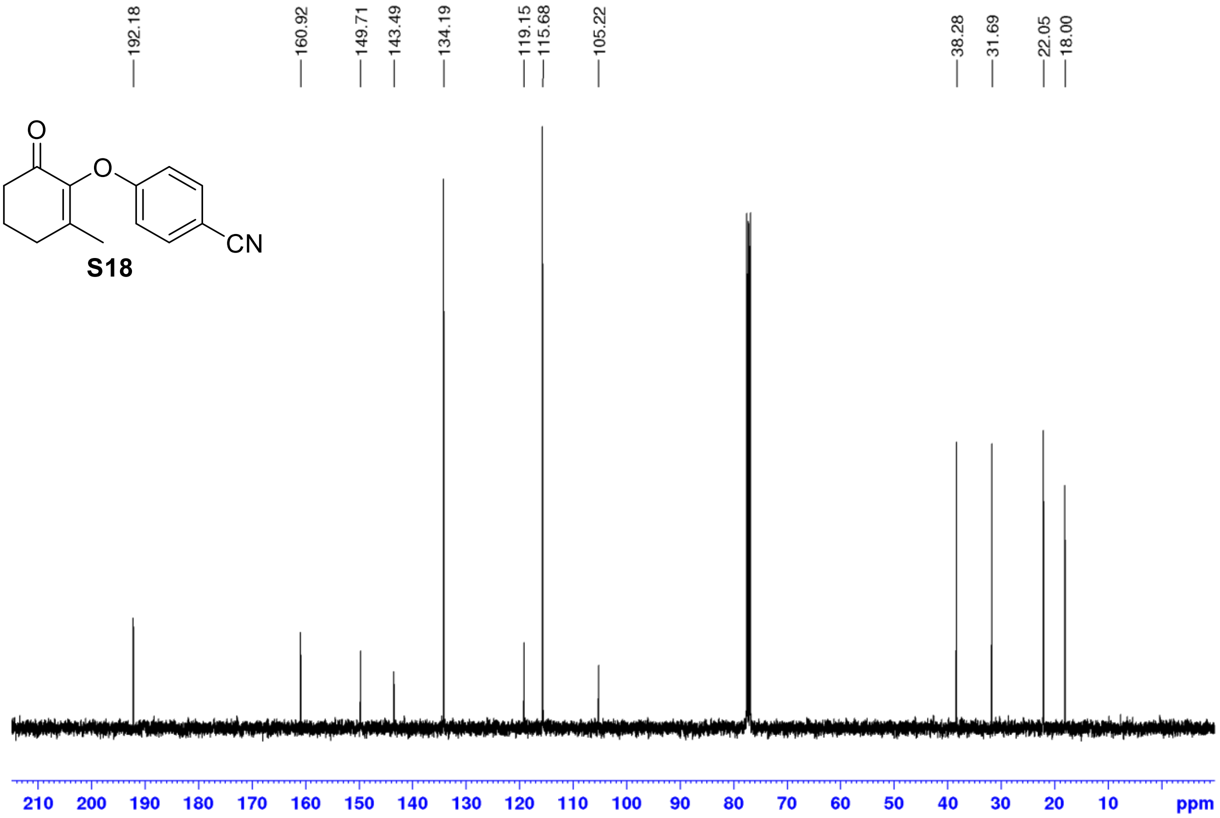
192.18
160.92
149.71
143.49
134.19
119.15
115.68
105.22
38.28
31.69
22.05
18.00
O
O
CN
S18
210
200
190
180
170
160
150
140
130
120
110
100
90
80
70
60
50
40
30
20
10
ppm

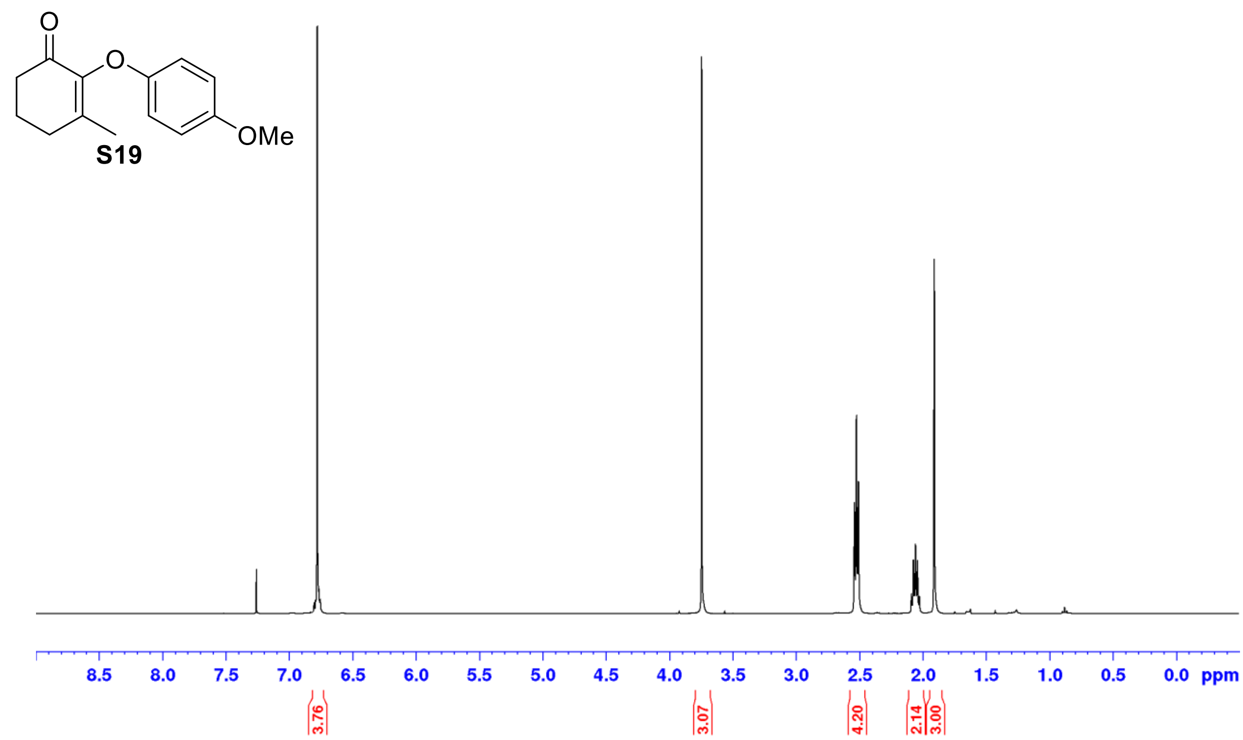
O
O
OMe
S19
8.5
8.0
7.5
7.0
6.5
6.0
5.5
5.0
4.5
4.0
3.5
3.0
2.5
2.0
1.5
1.0
0.5
0.0
ppm
3.76
3.07
4.20
2.14
3.00

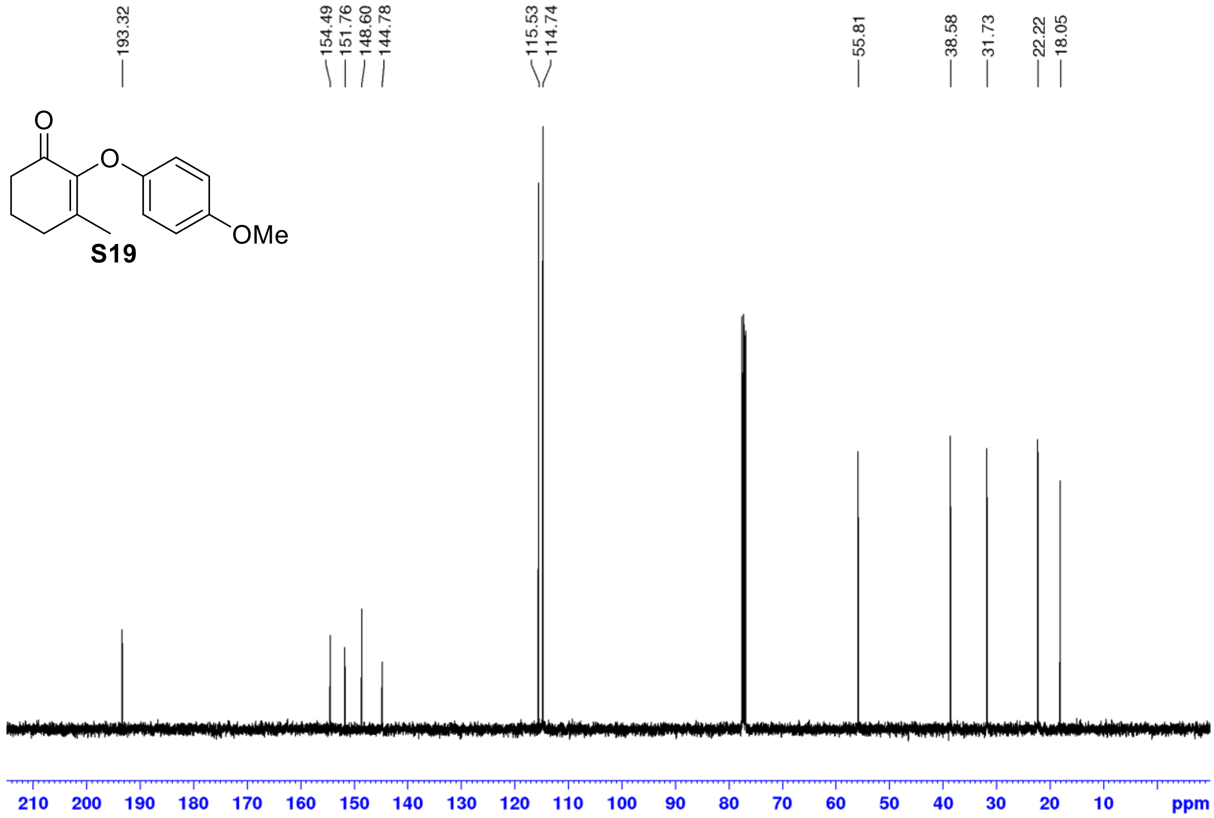
193.32
154.49
151.76
148.60
144.78
115.53
114.74
55.81
38.58
31.73
22.22
18.05
O
O
OMe
S19
210
200
190
180
170
160
150
140
130
120
110
100
90
80
70
60
50
40
30
20
10
ppm

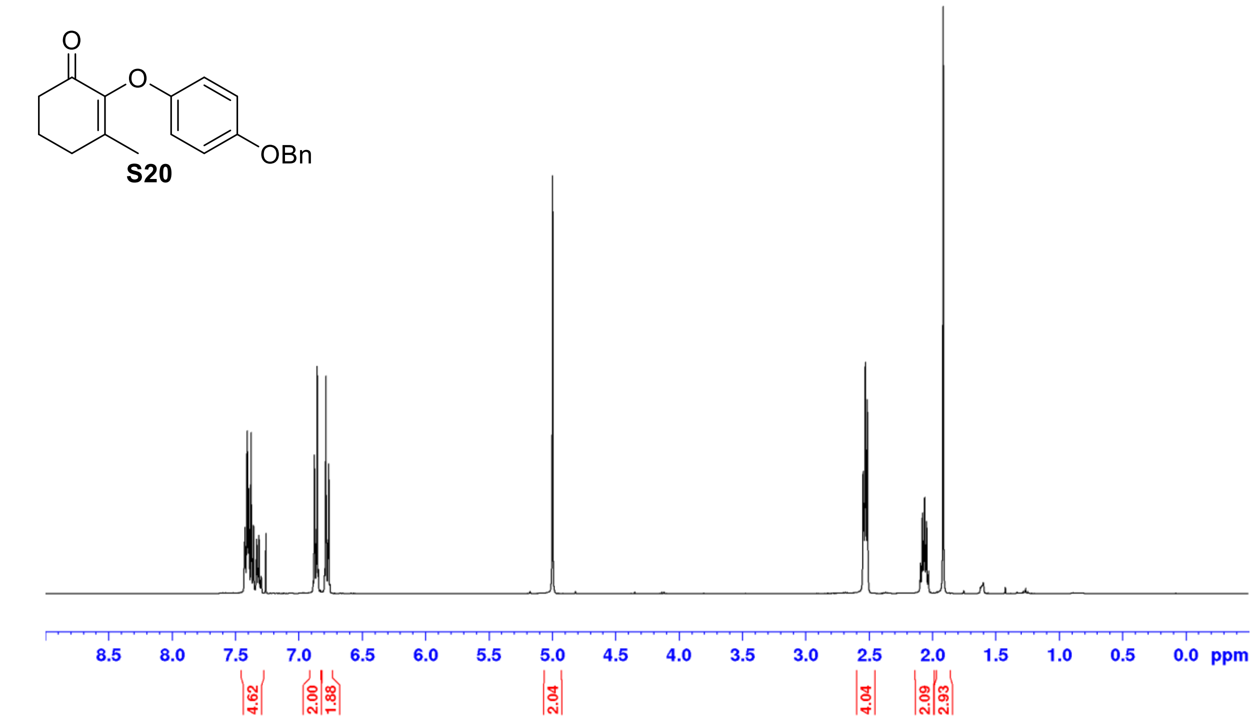
O
O
OBn
S20
8.5
8.0
7.5
7.0
6.5
6.0
5.5
5.0
4.5
4.0
3.5
3.0
2.5
2.0
1.5
1.0
0.5
0.0
ppm
4.62
2.00
1.88
2.04
4.04
2.09
2.93

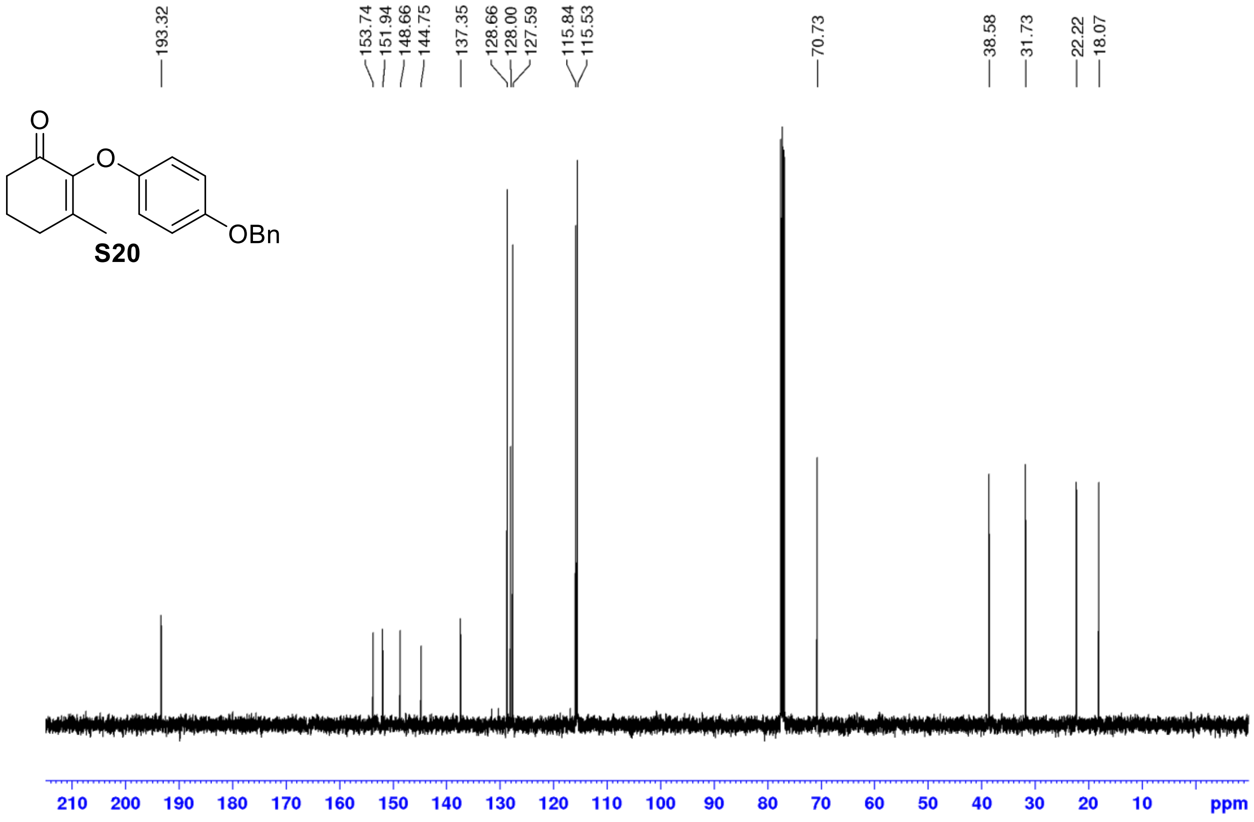
193.32
153.74
151.94
148.66
144.75
137.35
128.66
128.00
127.59
115.84
115.53
70.73
38.58
31.73
22.22
18.07
O
O
OBn
S20
210
200
190
180
170
160
150
140
130
120
110
100
90
80
70
60
50
40
30
20
10
ppm

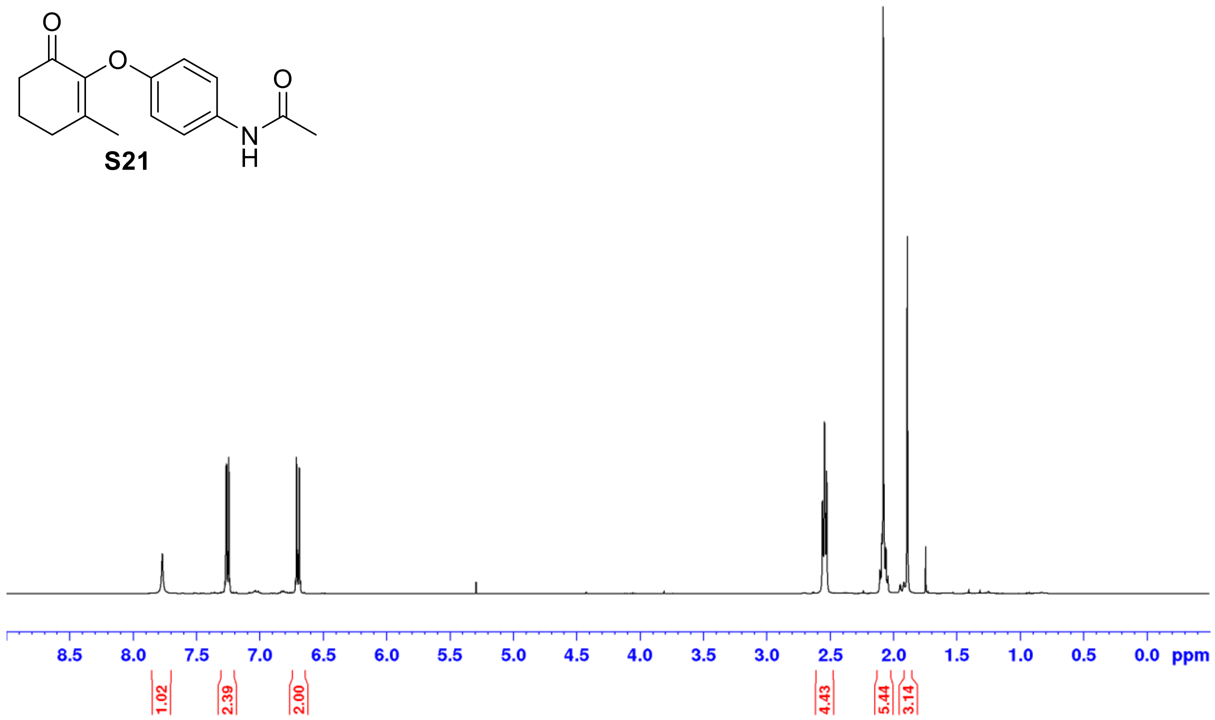
O
O
O
N
H
S21
8.5
8.0
7.5
7.0
6.5
6.0
5.5
5.0
4.5
4.0
3.5
3.0
2.5
2.0
1.5
1.0
0.5
0.0
ppm
1.02
2.39
2.00
4.43
5.44
3.14

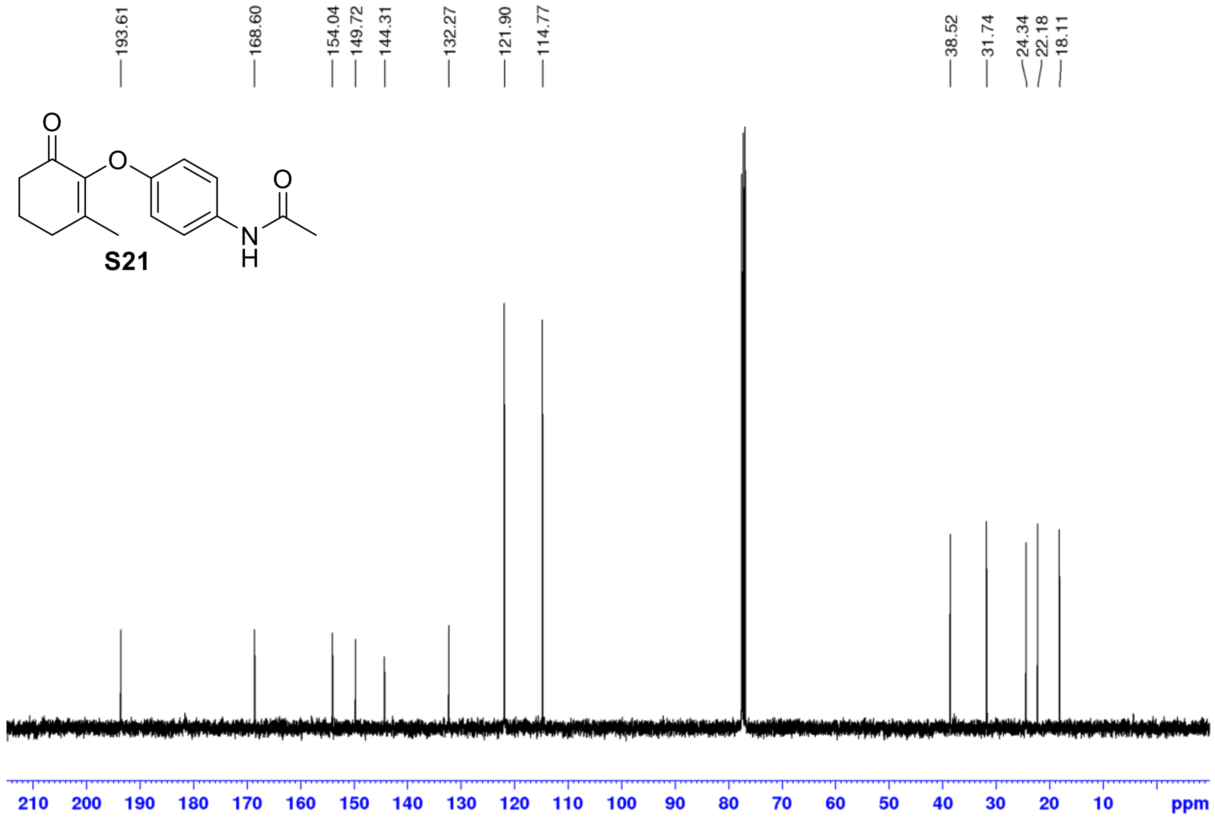
193.61
168.60
154.04
149.72
144.31
132.27
121.90
114.77
38.52
31.74
24.34
22.18
18.11
O
O
O
N
H
S21
210
200
190
180
170
160
150
140
130
120
110
100
90
80
70
60
50
40
30
20
10
ppm

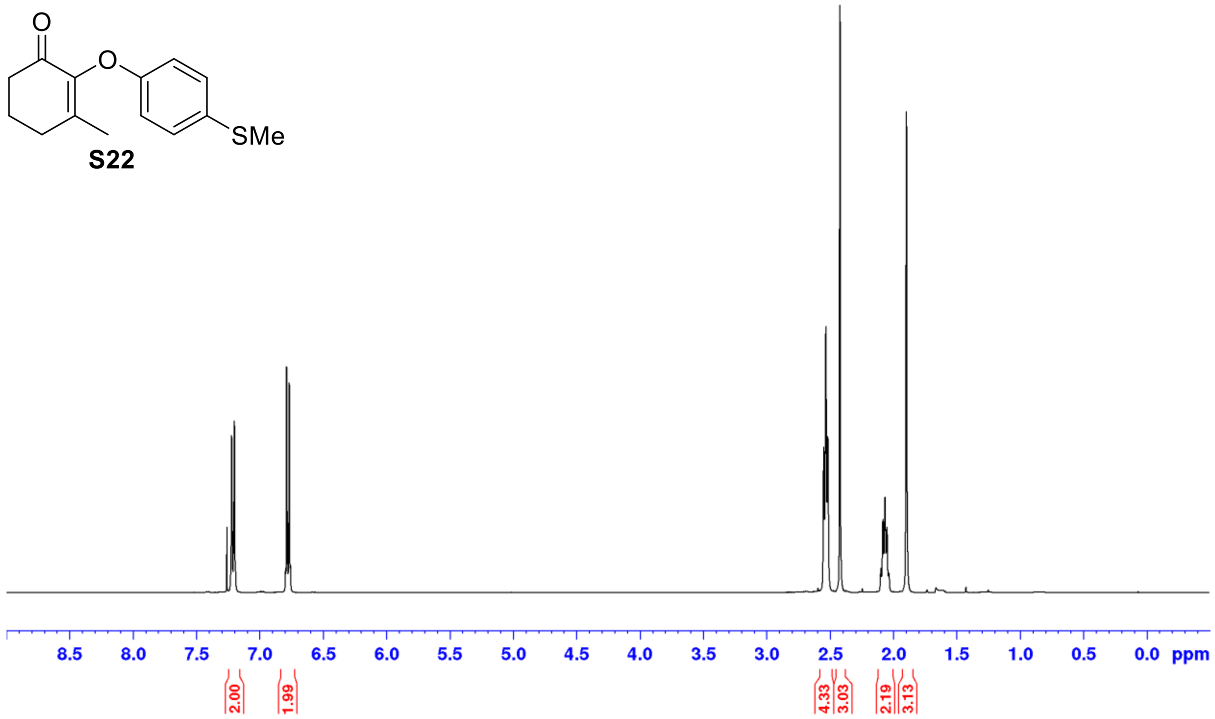

O
SMe
S22
8.5 8.0 7.5 7.0 6.5 6.0 5.5 5.0 4.5 4.0 3.5 3.0 2.5 2.0 1.5 1.0 0.5 0.0 ppm
2.00
1.99
4.33
3.03
2.19
3.13

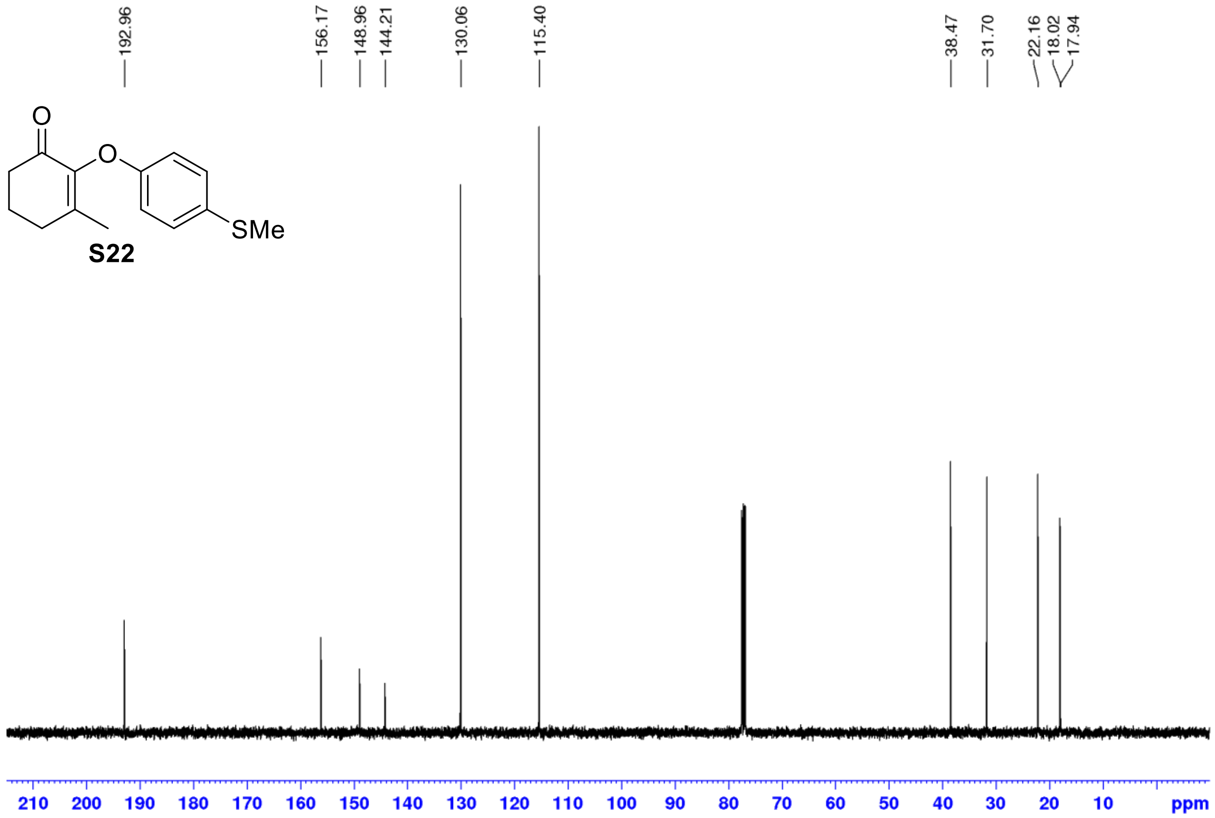

192.96
156.17
148.96
144.21
130.06
115.40
38.47
31.70
22.16
18.02
17.94
O
SMe
S22
210 200 190 180 170 160 150 140 130 120 110 100 90 80 70 60 50 40 30 20 10 ppm

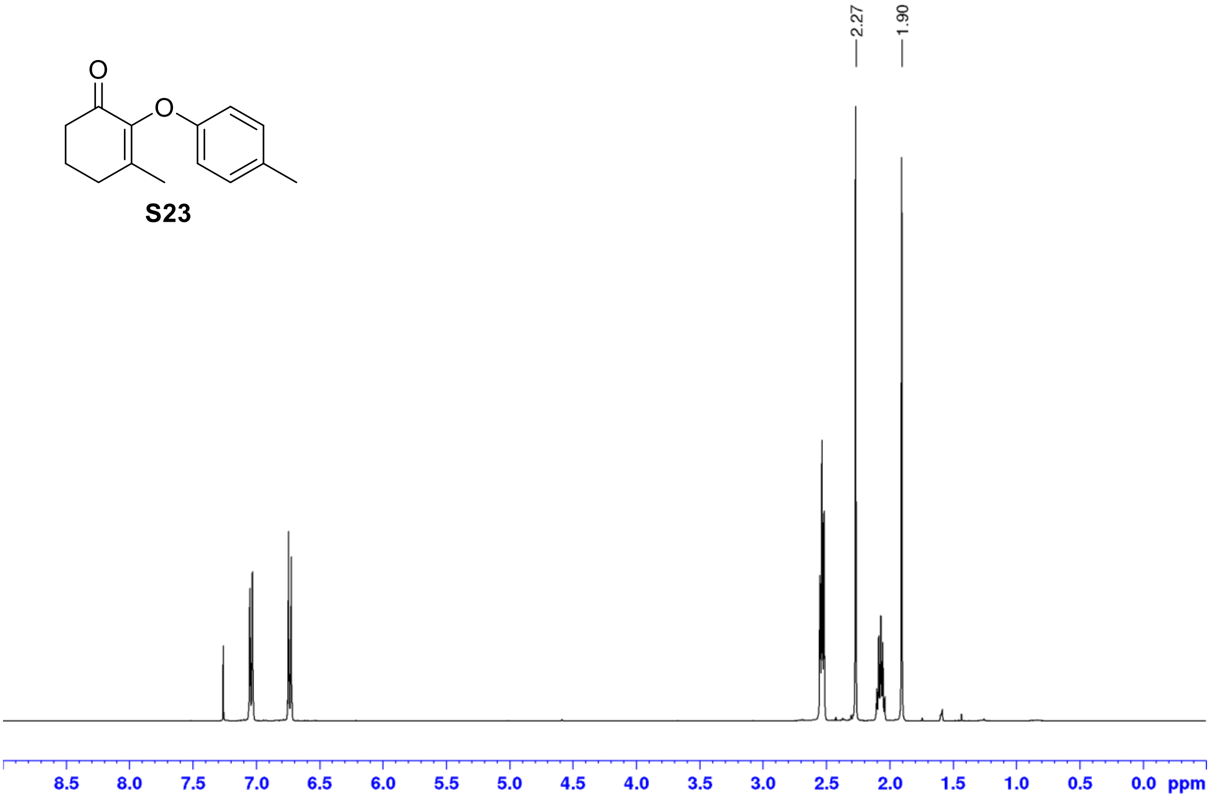
S23
2.27
1.90
8.5 8.0 7.5 7.0 6.5 6.0 5.5 5.0 4.5 4.0 3.5 3.0 2.5 2.0 1.5 1.0 0.5 0.0 ppm

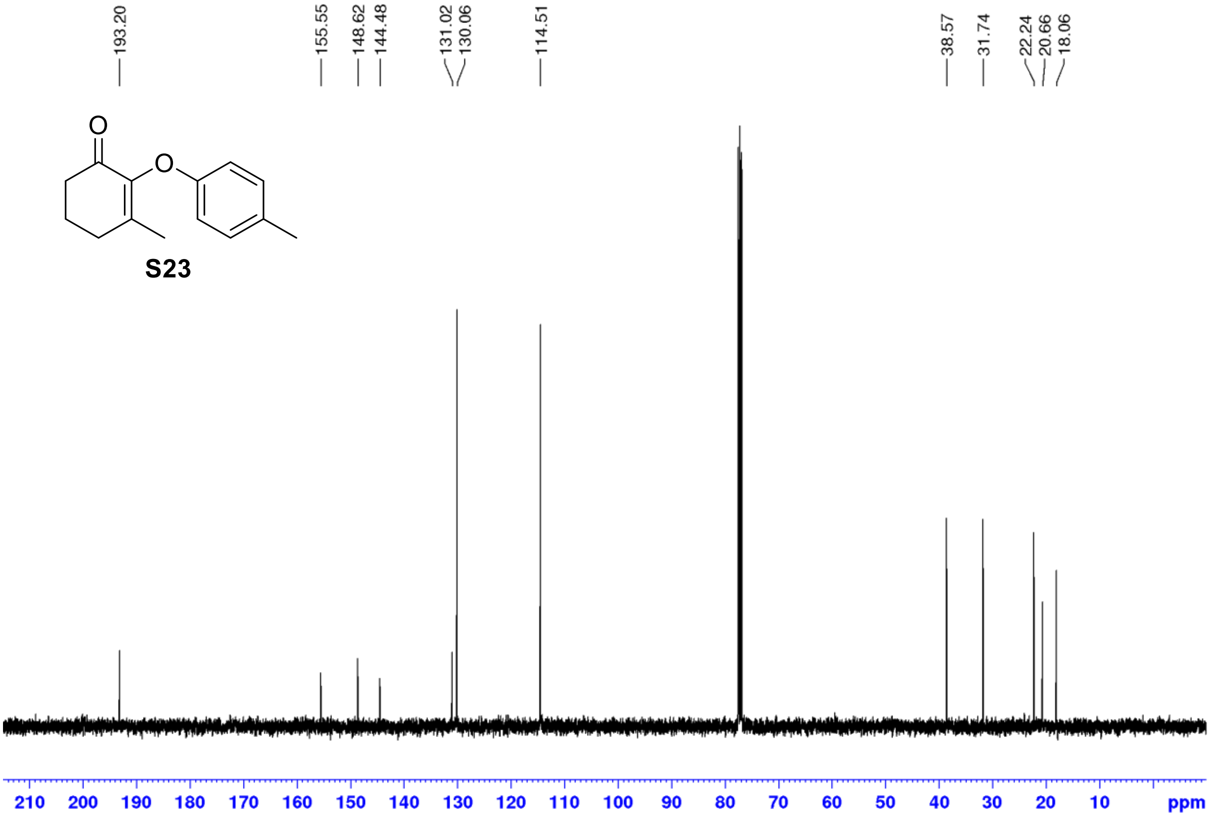
S23
193.20
155.55
148.62
144.48
131.02
130.06
114.51
38.57
31.74
22.24
20.66
18.06
210 200 190 180 170 160 150 140 130 120 110 100 90 80 70 60 50 40 30 20 10 ppm

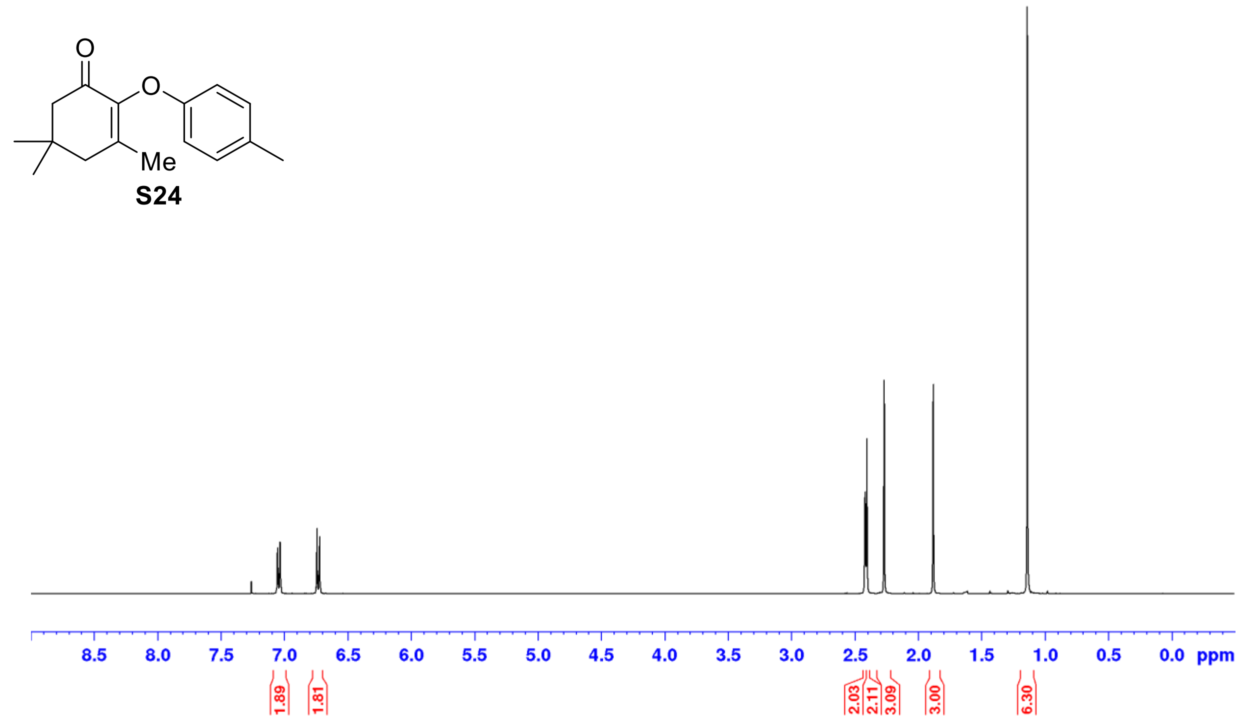
O
O
Me
S24
8.5
8.0
7.5
7.0
6.5
6.0
5.5
5.0
4.5
4.0
3.5
3.0
2.5
2.0
1.5
1.0
0.5
0.0
ppm
1.89
1.81
2.03
2.11
3.09
3.00
6.30

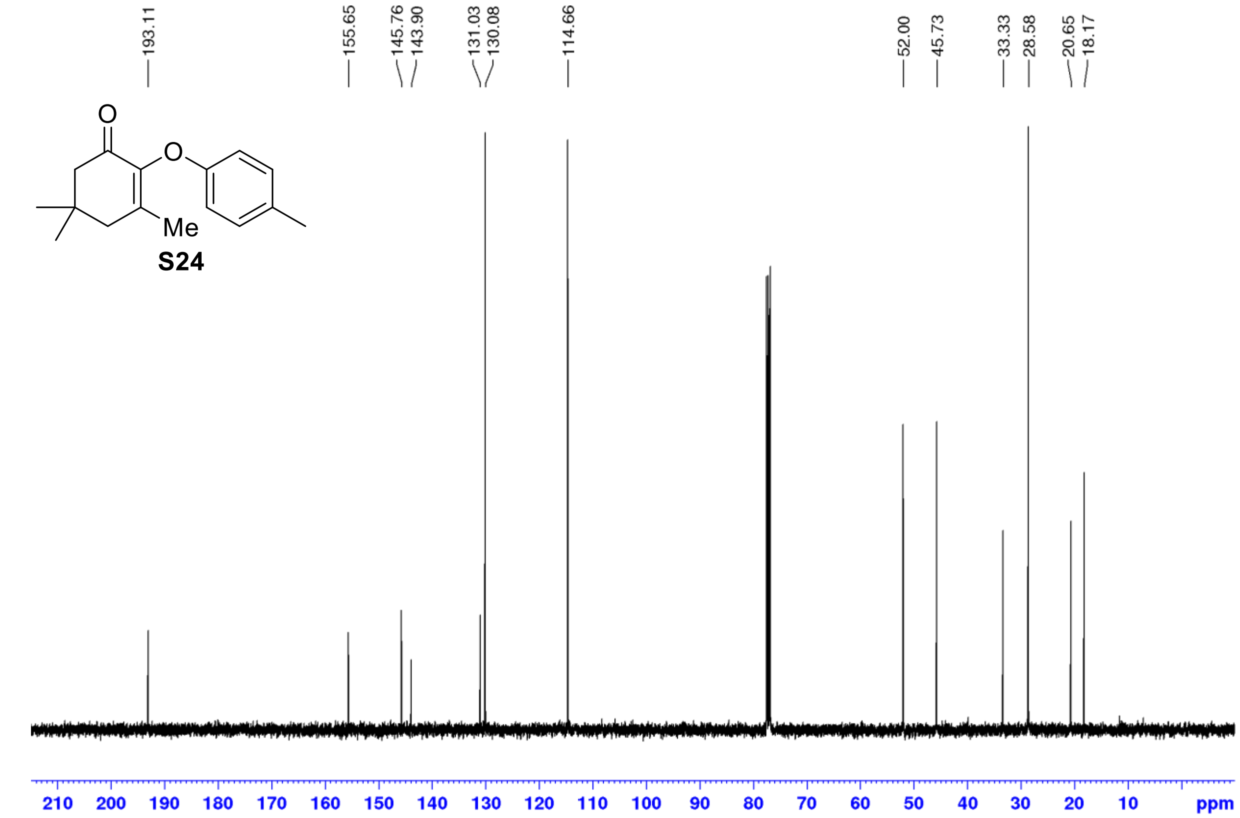
193.11
155.65
145.76
143.90
131.03
130.08
114.66
52.00
45.73
33.33
28.58
20.65
18.17
O
O
Me
S24
210
200
190
180
170
160
150
140
130
120
110
100
90
80
70
60
50
40
30
20
10
ppm

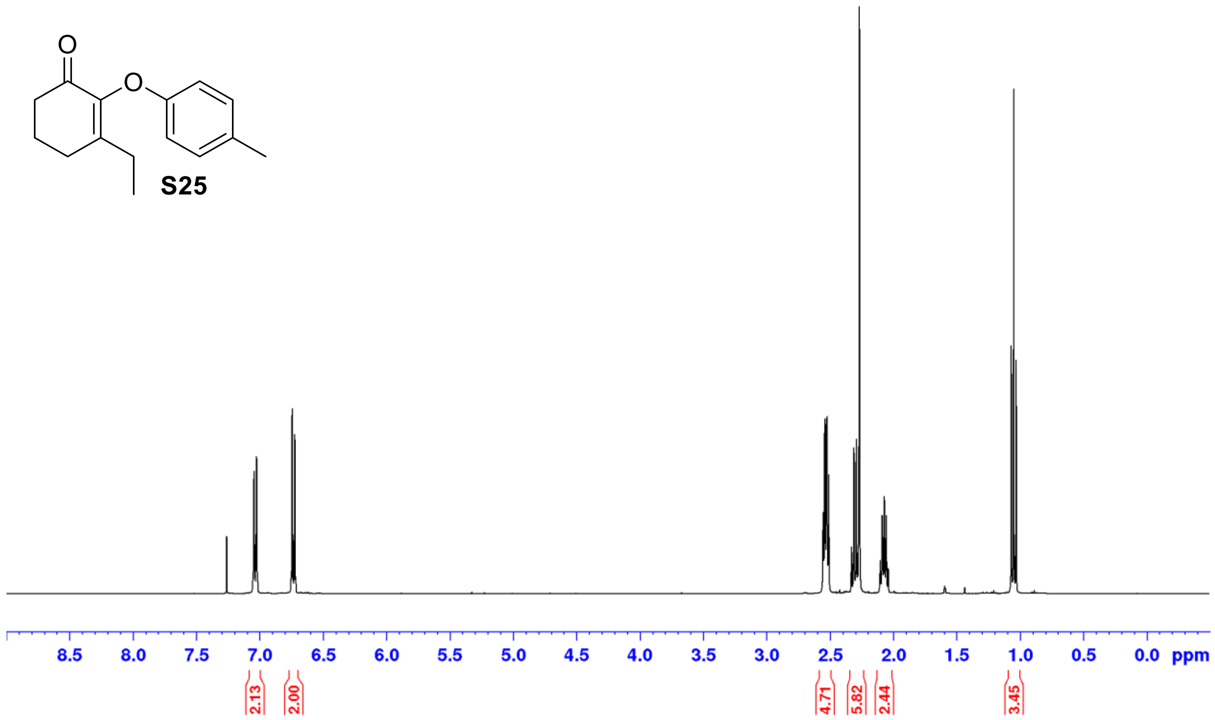
S25
8.5 8.0 7.5 7.0 6.5 6.0 5.5 5.0 4.5 4.0 3.5 3.0 2.5 2.0 1.5 1.0 0.5 0.0 ppm
2.13 2.00 4.71 5.82 2.44 3.45

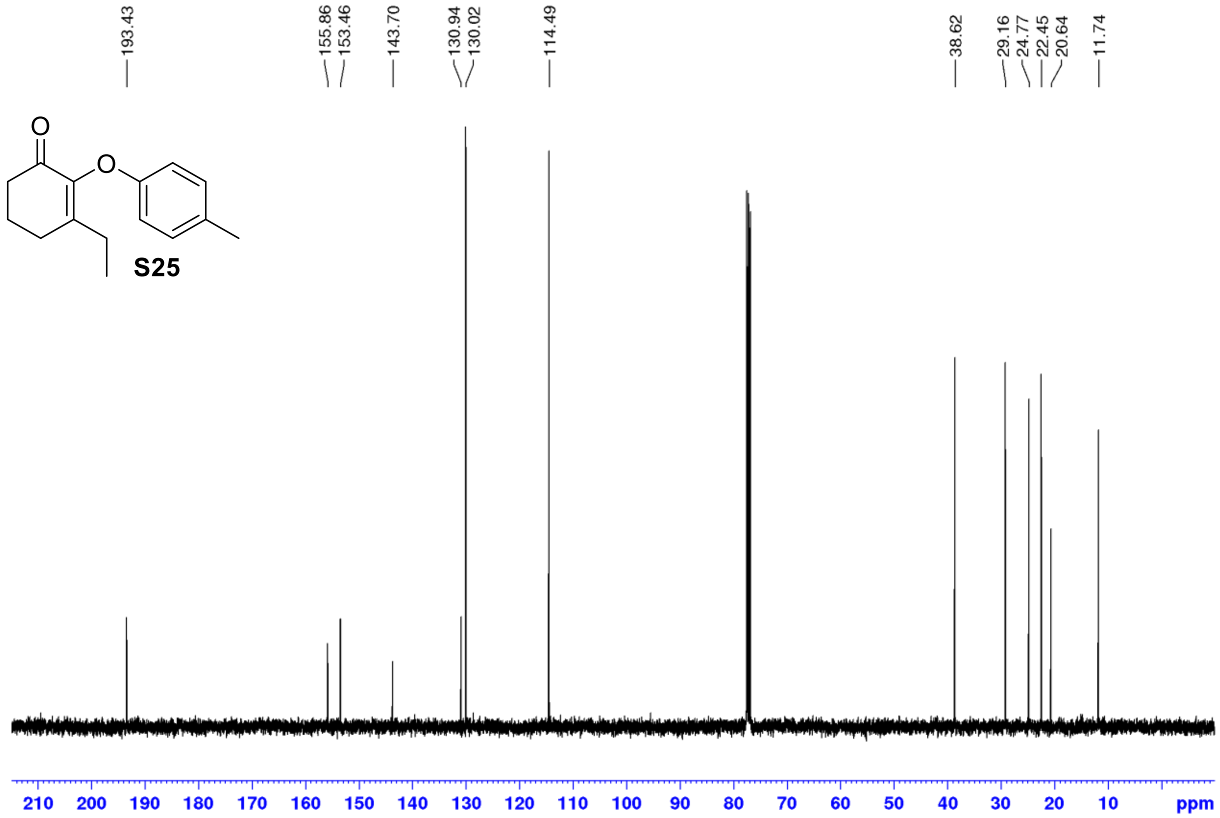
193.43
155.86 153.46
143.70
130.94 130.02
114.49
38.62
29.16 24.77 22.45 20.64
11.74
S25
210 200 190 180 170 160 150 140 130 120 110 100 90 80 70 60 50 40 30 20 10 ppm

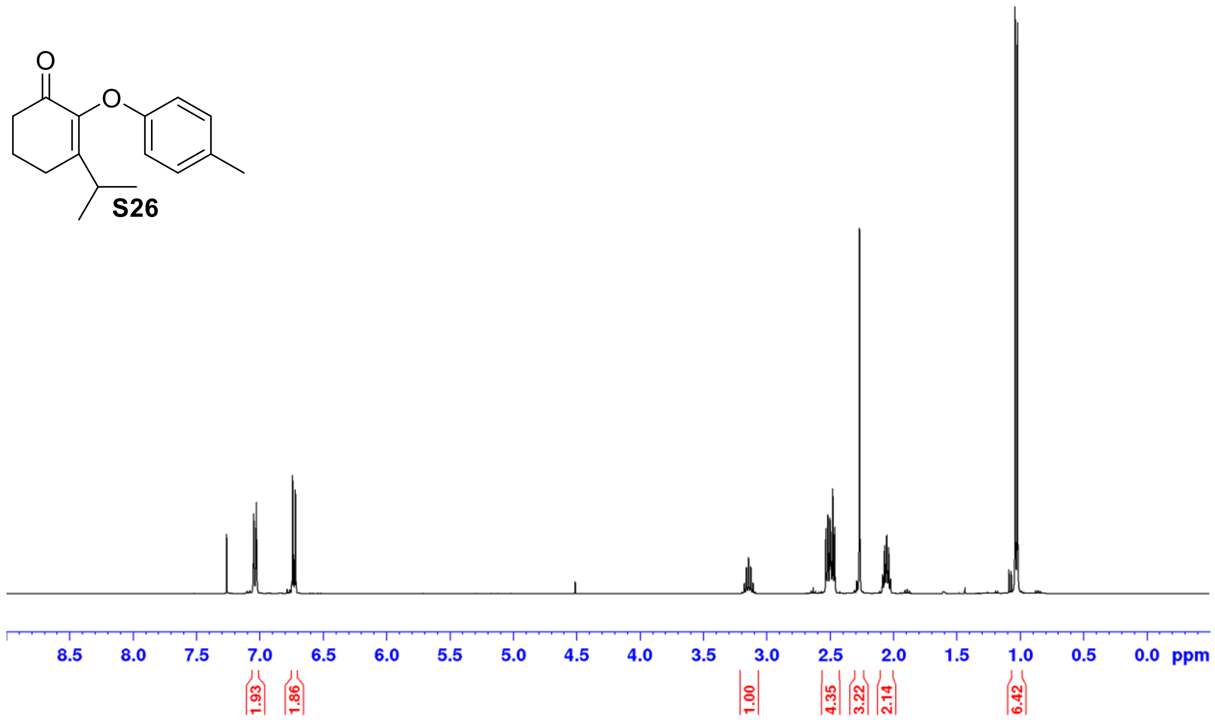
O
O
S26
8.5 8.0 7.5 7.0 6.5 6.0 5.5 5.0 4.5 4.0 3.5 3.0 2.5 2.0 1.5 1.0 0.5 0.0 ppm
1.93
1.86
1.00
4.35
3.22
2.14
6.42

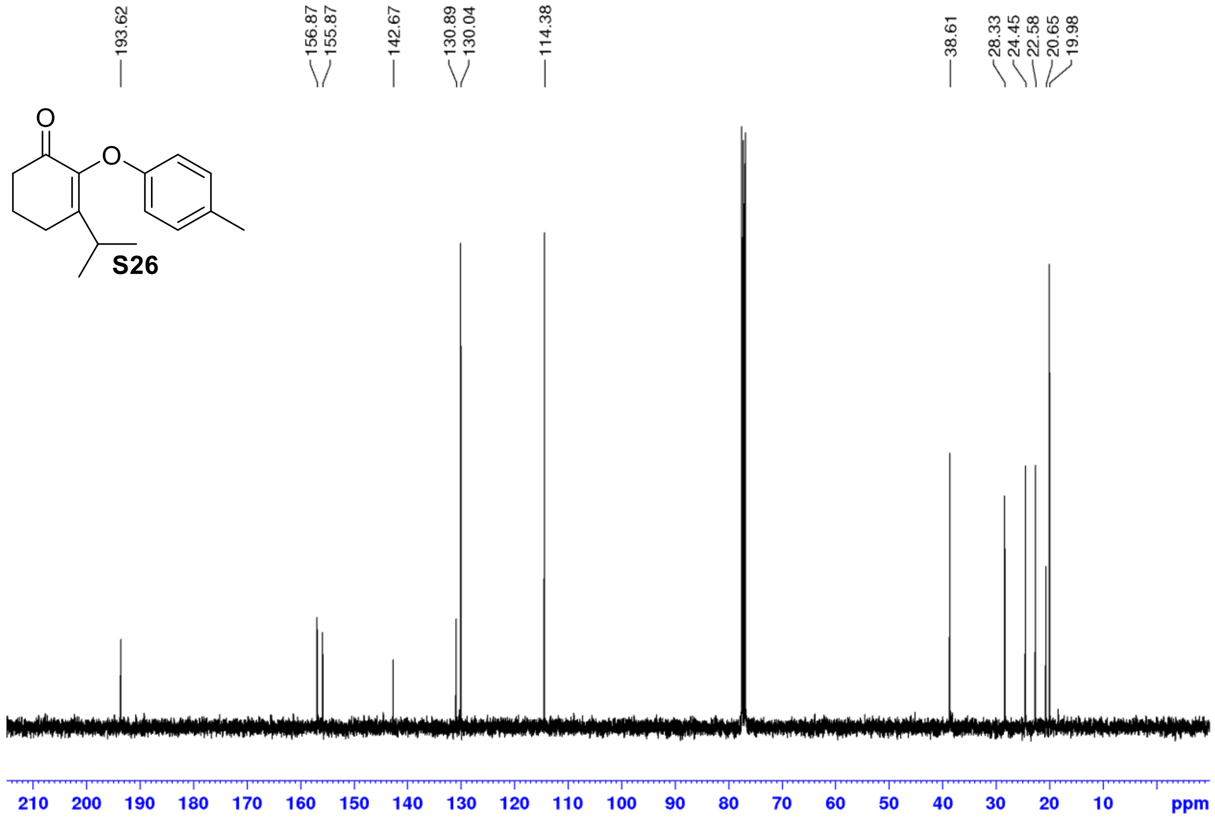
193.62
156.87
155.87
142.67
130.89
130.04
114.38
38.61
28.33
24.45
22.58
20.65
19.98
O
O
S26
210 200 190 180 170 160 150 140 130 120 110 100 90 80 70 60 50 40 30 20 10 ppm

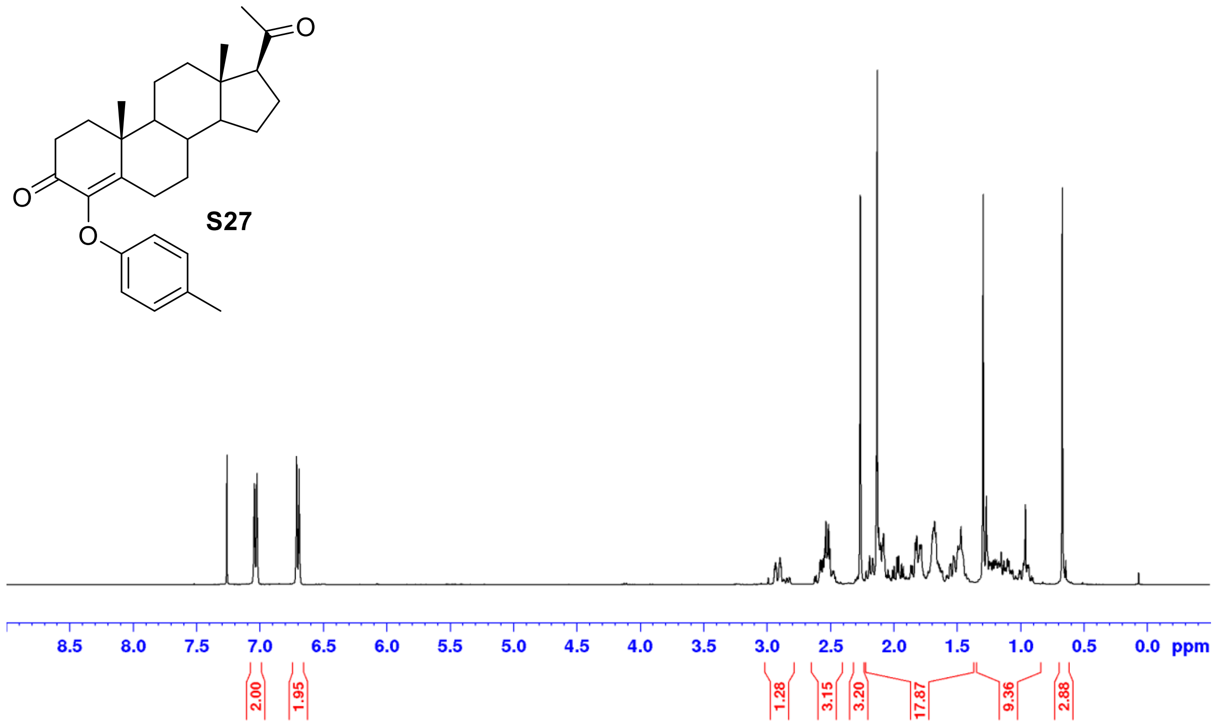

S27
8.5
8.0
7.5
7.0
6.5
6.0
5.5
5.0
4.5
4.0
3.5
3.0
2.5
2.0
1.5
1.0
0.5
0.0
ppm
2.00
1.95
1.28
3.15
3.20
17.87
9.36
2.88

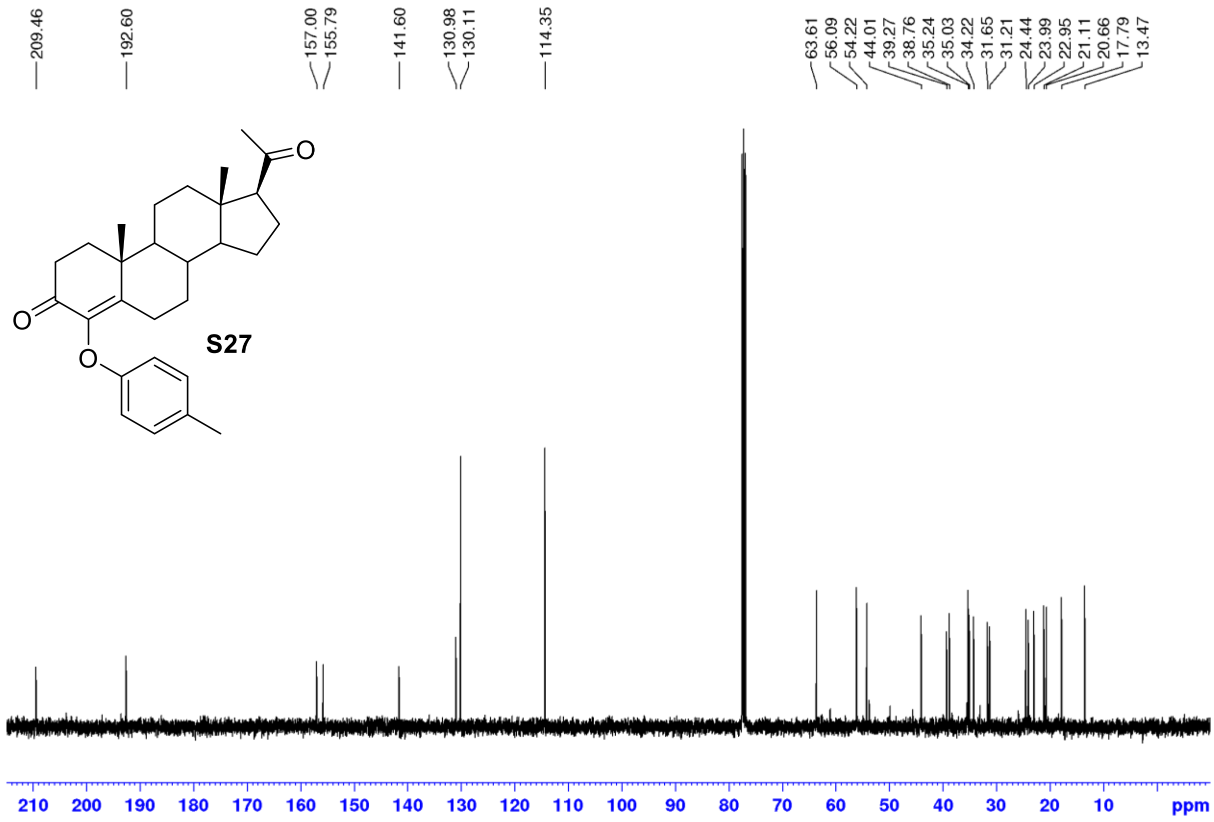

209.46
192.60
157.00
155.79
141.60
130.98
130.11
114.35
63.61
56.09
54.22
44.01
39.27
38.76
35.24
35.03
34.22
31.65
31.21
24.44
23.99
22.95
21.11
20.66
17.79
13.47
S27
210
200
190
180
170
160
150
140
130
120
110
100
90
80
70
60
50
40
30
20
10
ppm

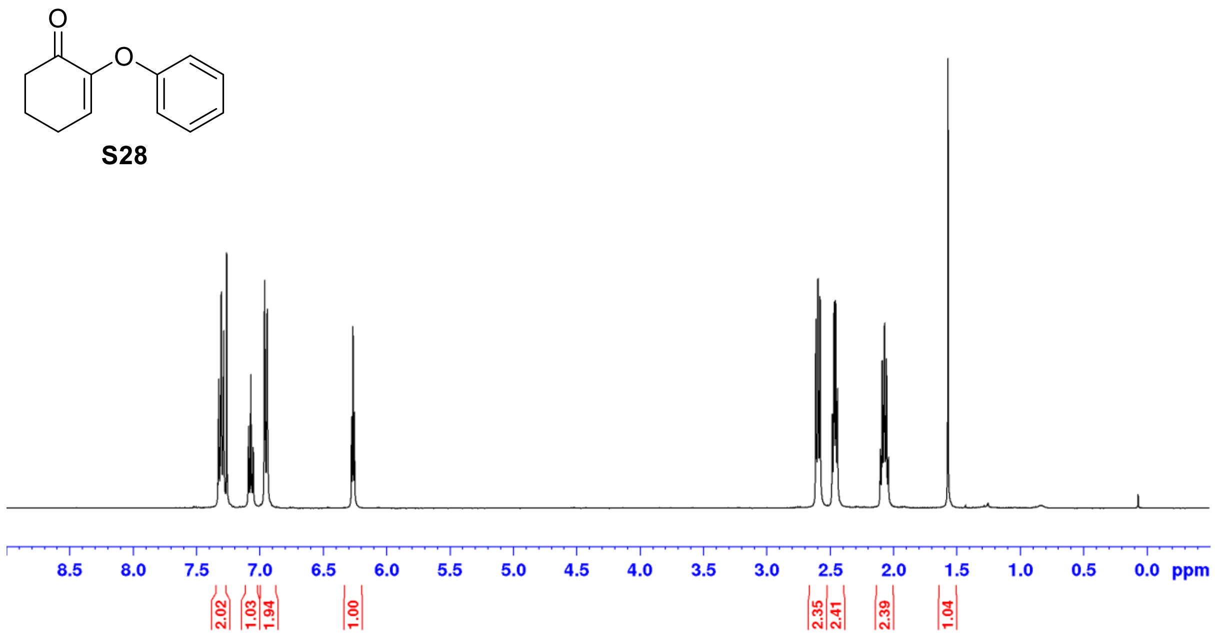
S28
2.02
1.03
1.94
1.00
2.35
2.41
2.39
1.04
8.5 8.0 7.5 7.0 6.5 6.0 5.5 5.0 4.5 4.0 3.5 3.0 2.5 2.0 1.5 1.0 0.5 0.0 ppm

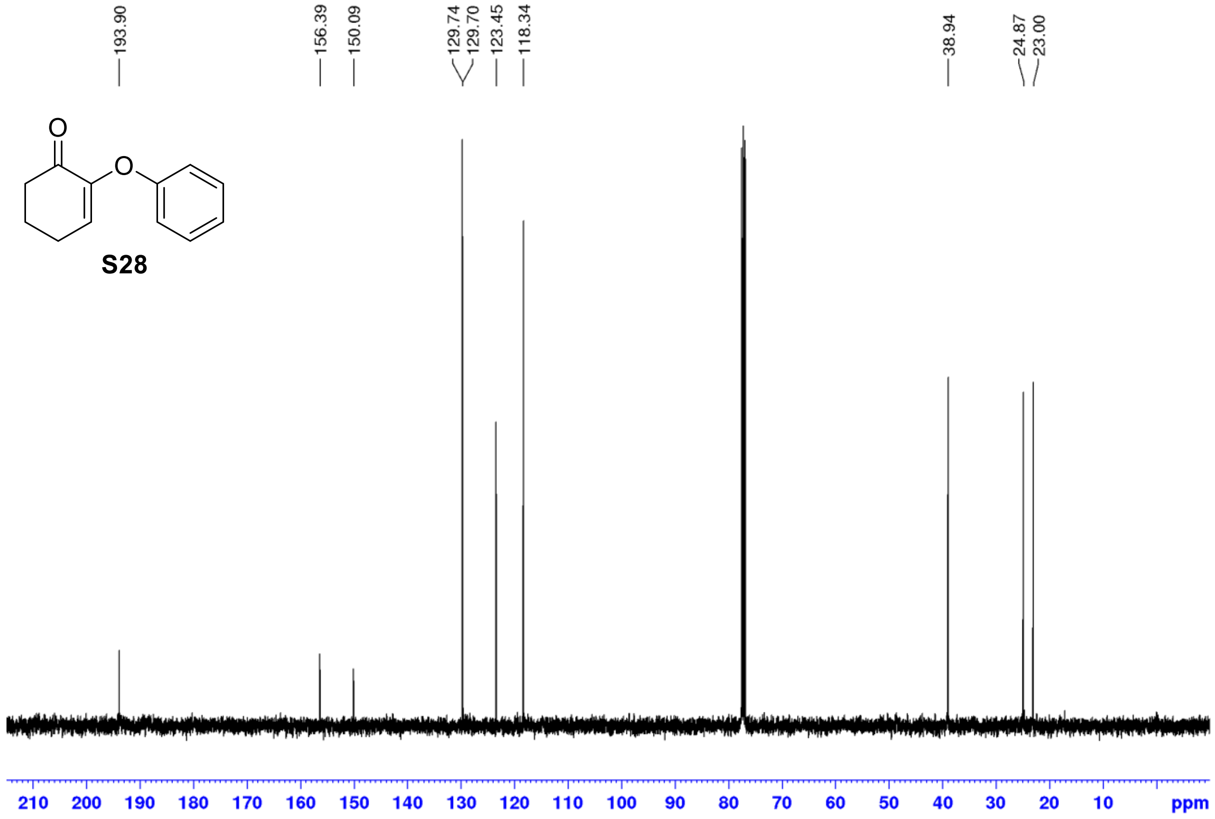
193.90
156.39
150.09
129.74
129.70
123.45
118.34
38.94
24.87
23.00
S28
210 200 190 180 170 160 150 140 130 120 110 100 90 80 70 60 50 40 30 20 10 ppm

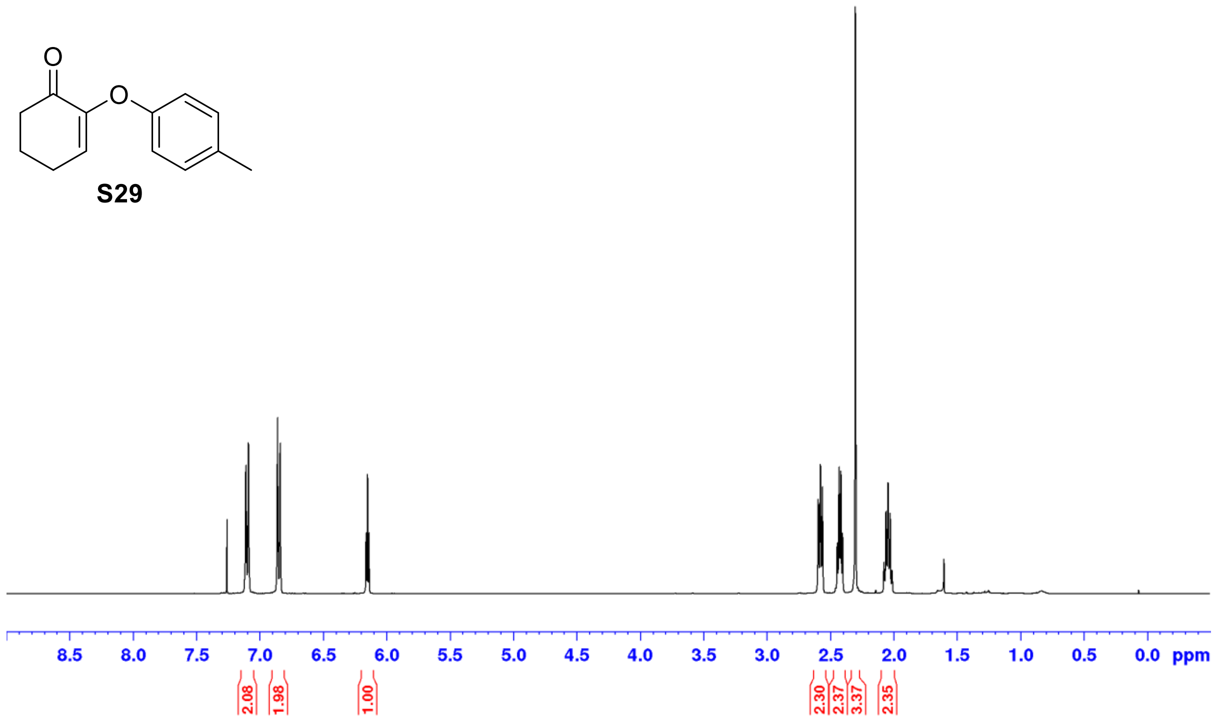
S29
8.5
8.0
7.5
7.0
6.5
6.0
5.5
5.0
4.5
4.0
3.5
3.0
2.5
2.0
1.5
1.0
0.5
0.0
ppm
2.08
1.98
1.00
2.30
2.37
3.37
2.35

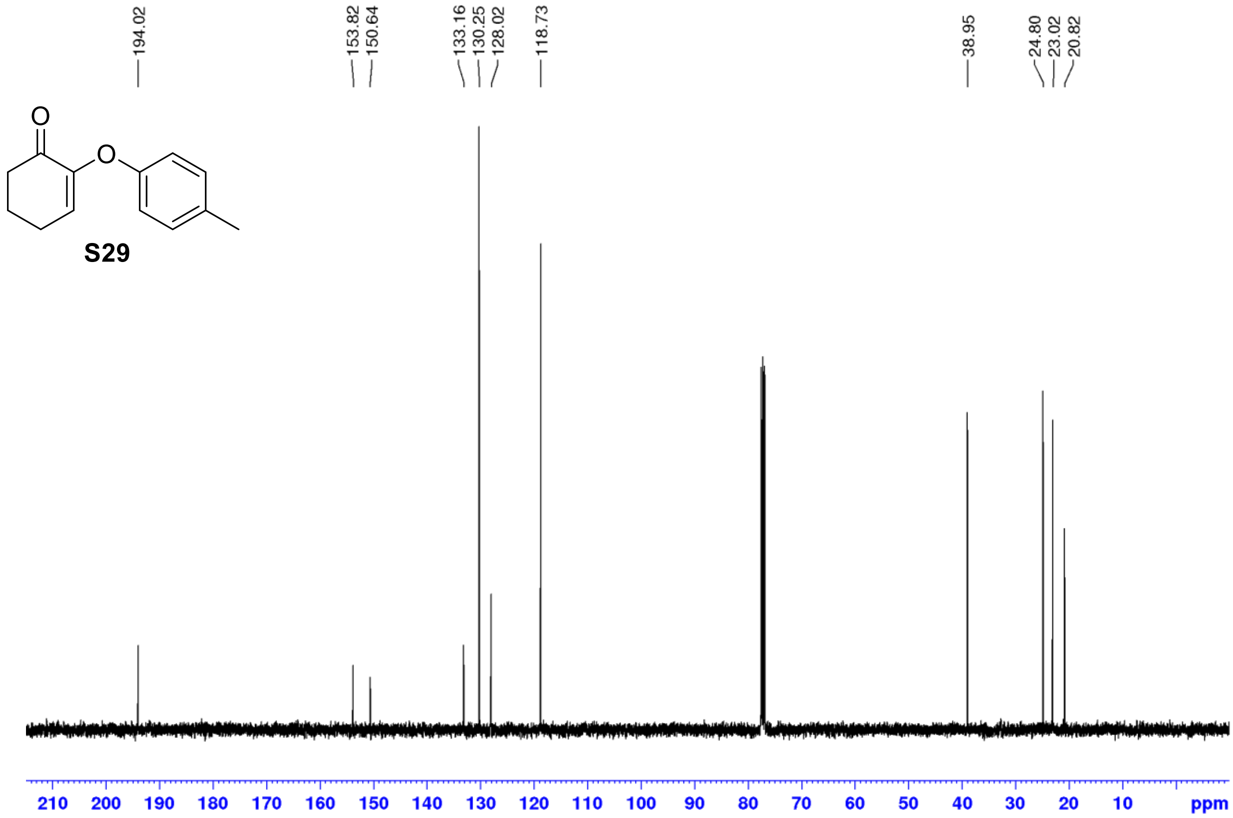
194.02
153.82
150.64
133.16
130.25
128.02
118.73
38.95
24.80
23.02
20.82
S29
210
200
190
180
170
160
150
140
130
120
110
100
90
80
70
60
50
40
30
20
10
ppm

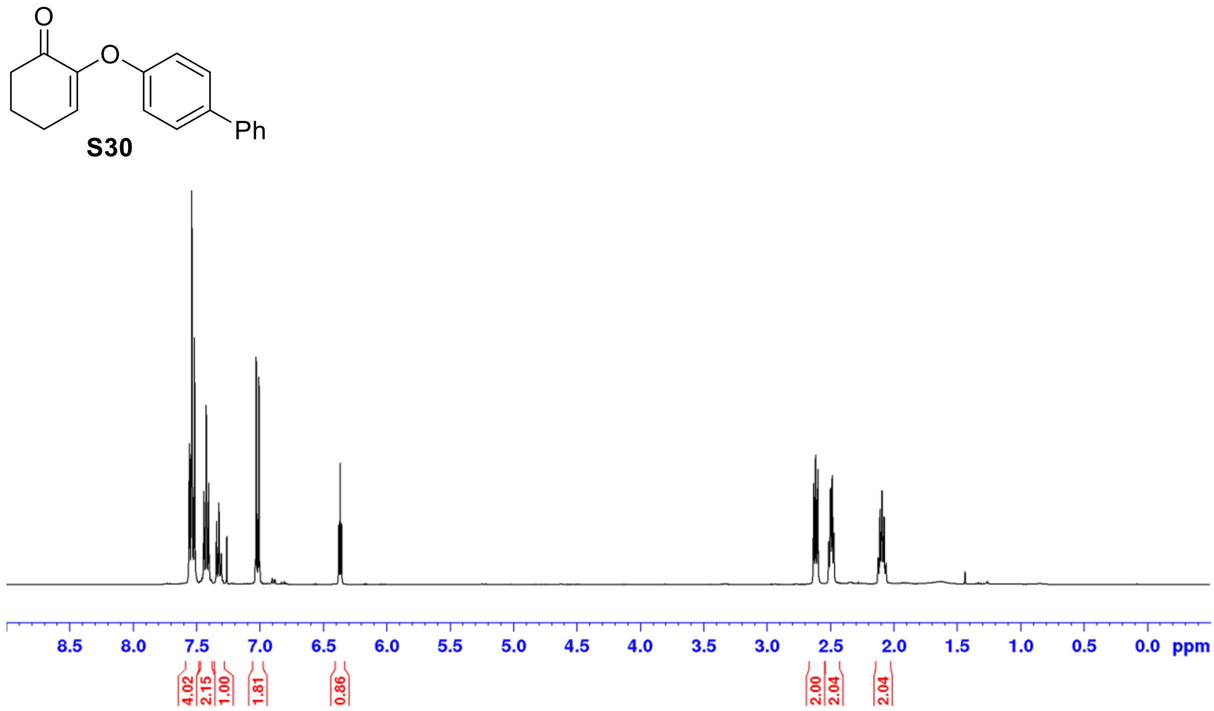
O
O
Ph
8.5
8.0
7.5
7.0
6.5
6.0
5.5
5.0
4.5
4.0
3.5
3.0
2.5
2.0
1.5
1.0
0.5
0.0
ppm
4.02
2.15
1.00
1.81
0.86
2.00
2.04
2.04

S30

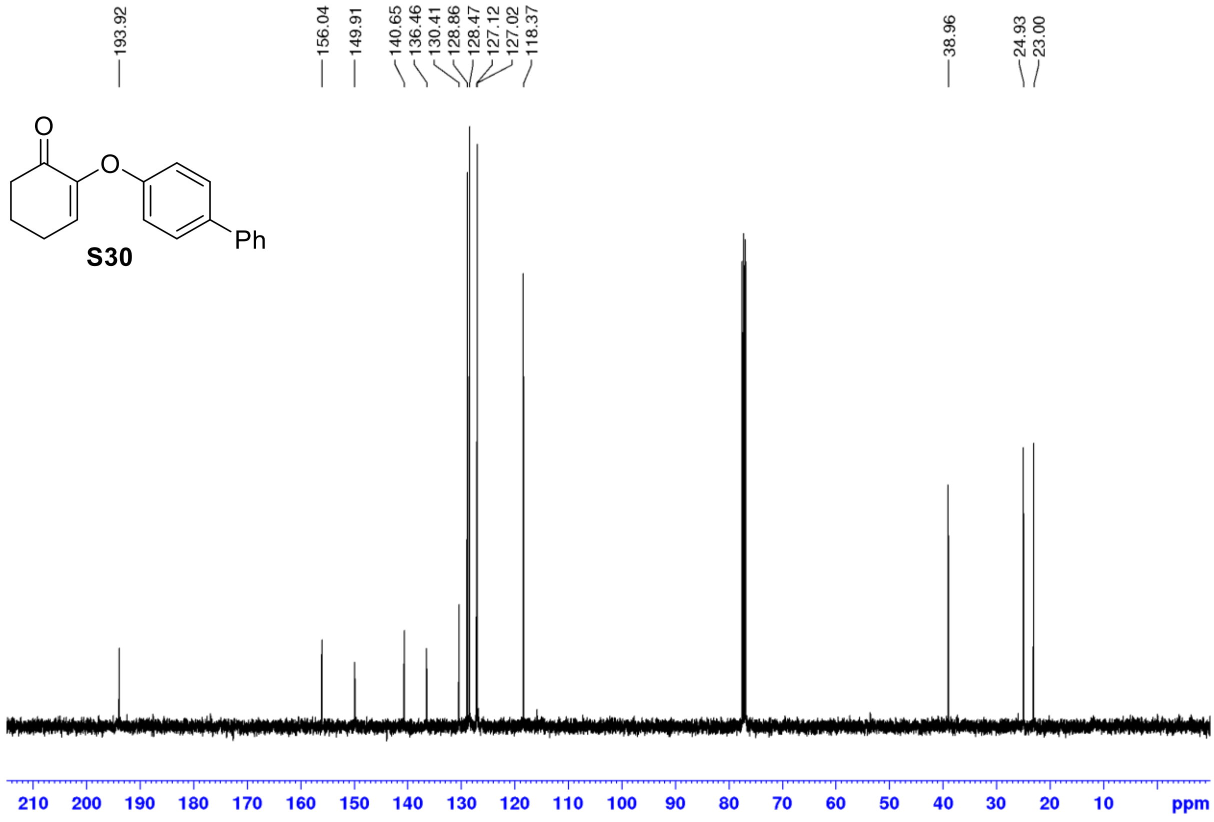
193.92
156.04
149.91
140.65
136.46
130.41
128.86
128.47
127.12
127.02
118.37
38.96
24.93
23.00
O
O
Ph
210
200
190
180
170
160
150
140
130
120
110
100
90
80
70
60
50
40
30
20
10
ppm

S30

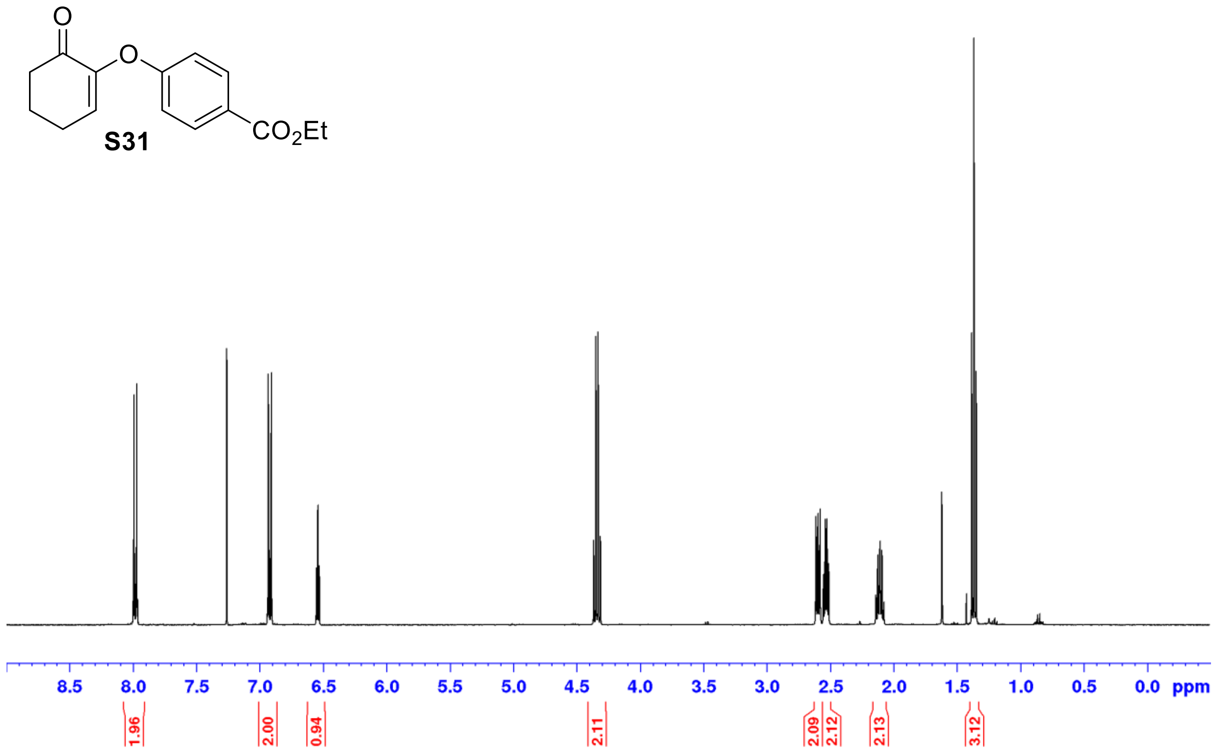
S31
CO2Et
8.5
8.0
7.5
7.0
6.5
6.0
5.5
5.0
4.5
4.0
3.5
3.0
2.5
2.0
1.5
1.0
0.5
0.0
ppm
1.96
2.00
0.94
2.11
2.09
2.12
2.13
3.12

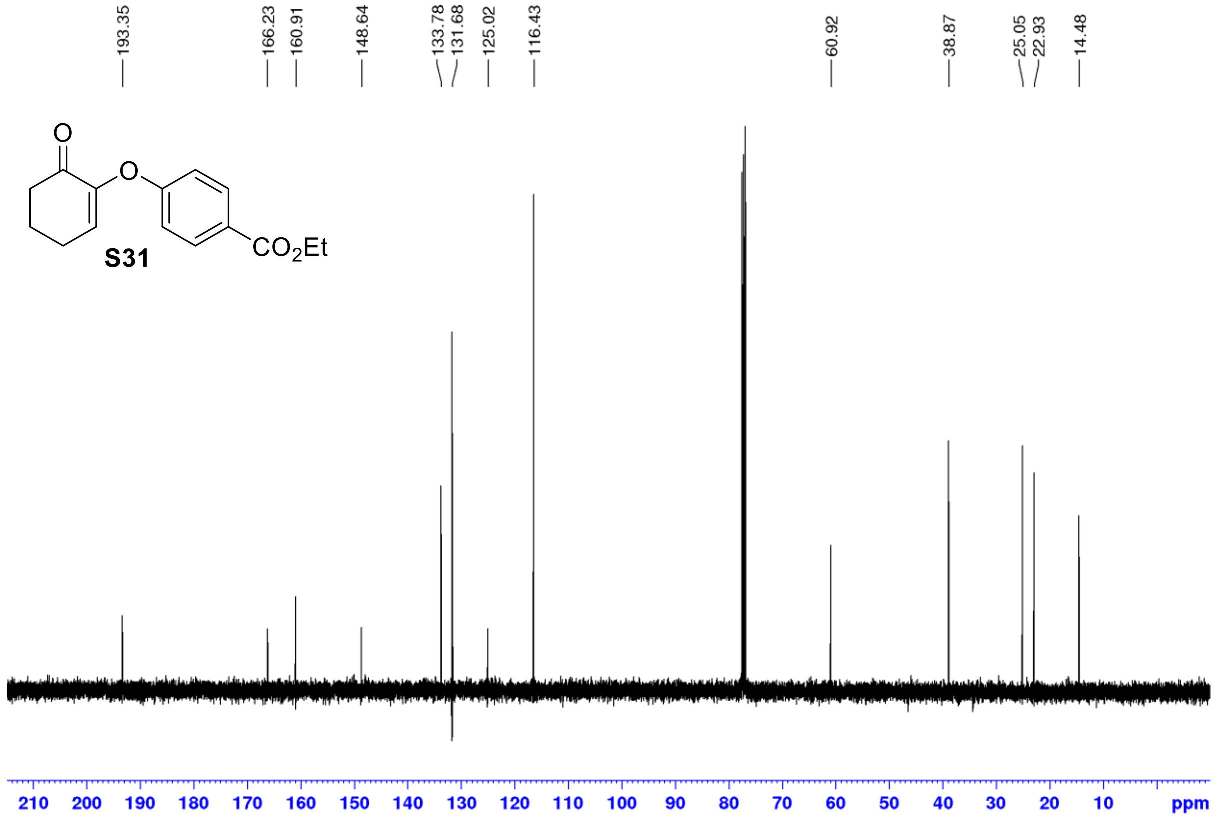
193.35
166.23
160.91
148.64
133.78
131.68
125.02
116.43
60.92
38.87
25.05
22.93
14.48
S31
CO2Et
210
200
190
180
170
160
150
140
130
120
110
100
90
80
70
60
50
40
30
20
10
ppm

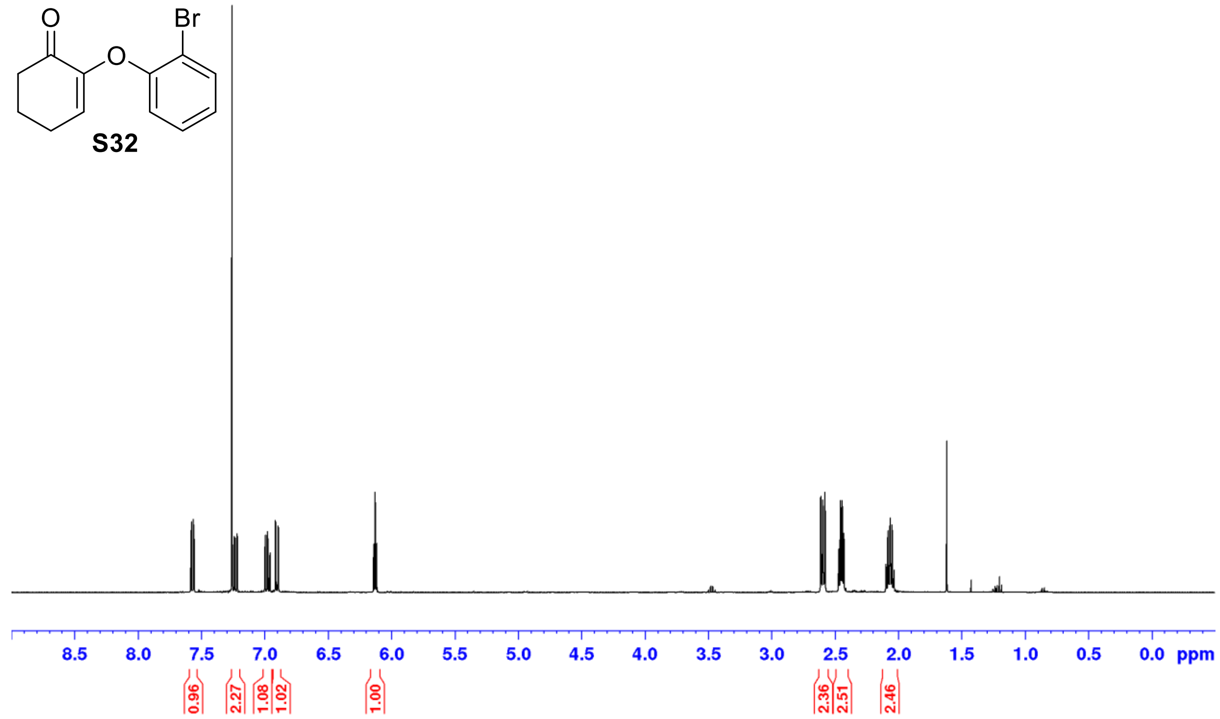
S32
8.5
8.0
7.5
7.0
6.5
6.0
5.5
5.0
4.5
4.0
3.5
3.0
2.5
2.0
1.5
1.0
0.5
0.0
ppm
0.96
2.27
1.08
1.02
1.00
2.36
2.51
2.46

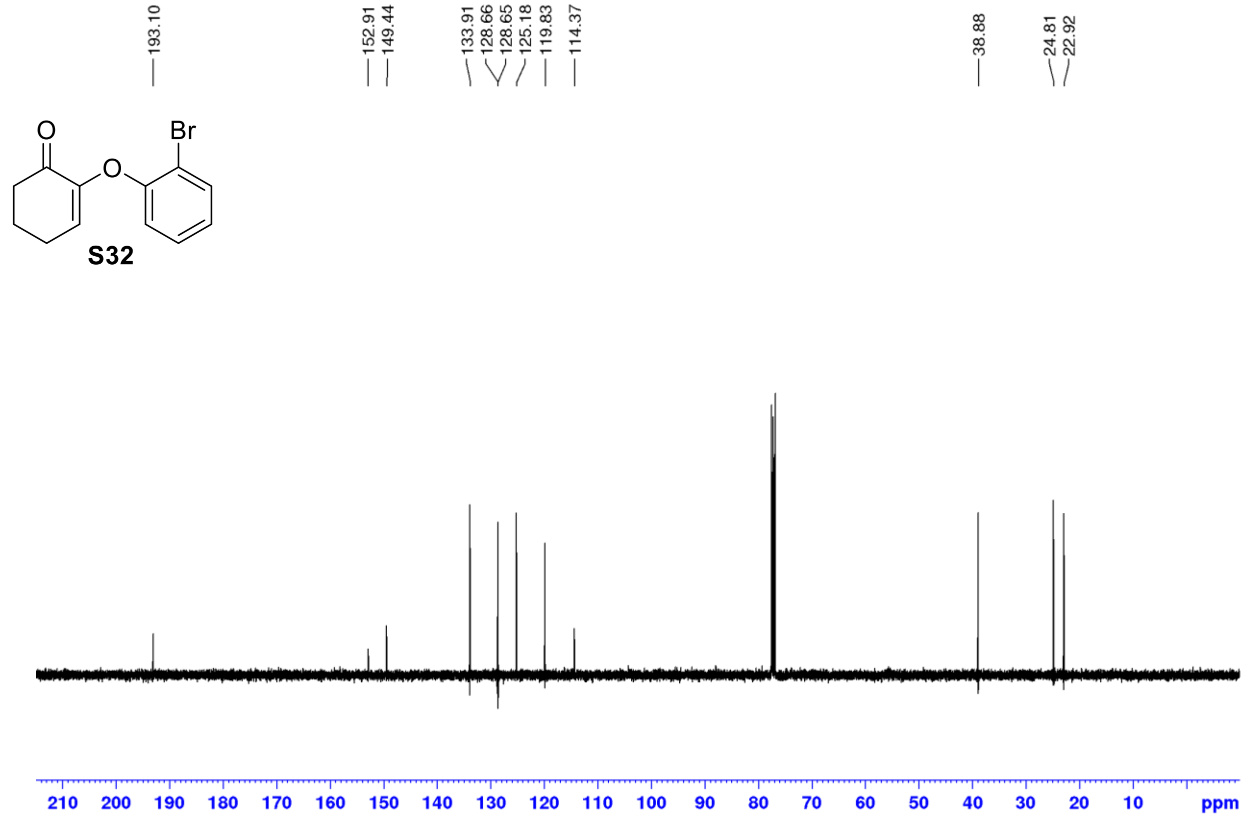
193.10
152.91
149.44
133.91
128.66
128.65
125.18
119.83
114.37
38.88
24.81
22.92
S32
210
200
190
180
170
160
150
140
130
120
110
100
90
80
70
60
50
40
30
20
10
ppm

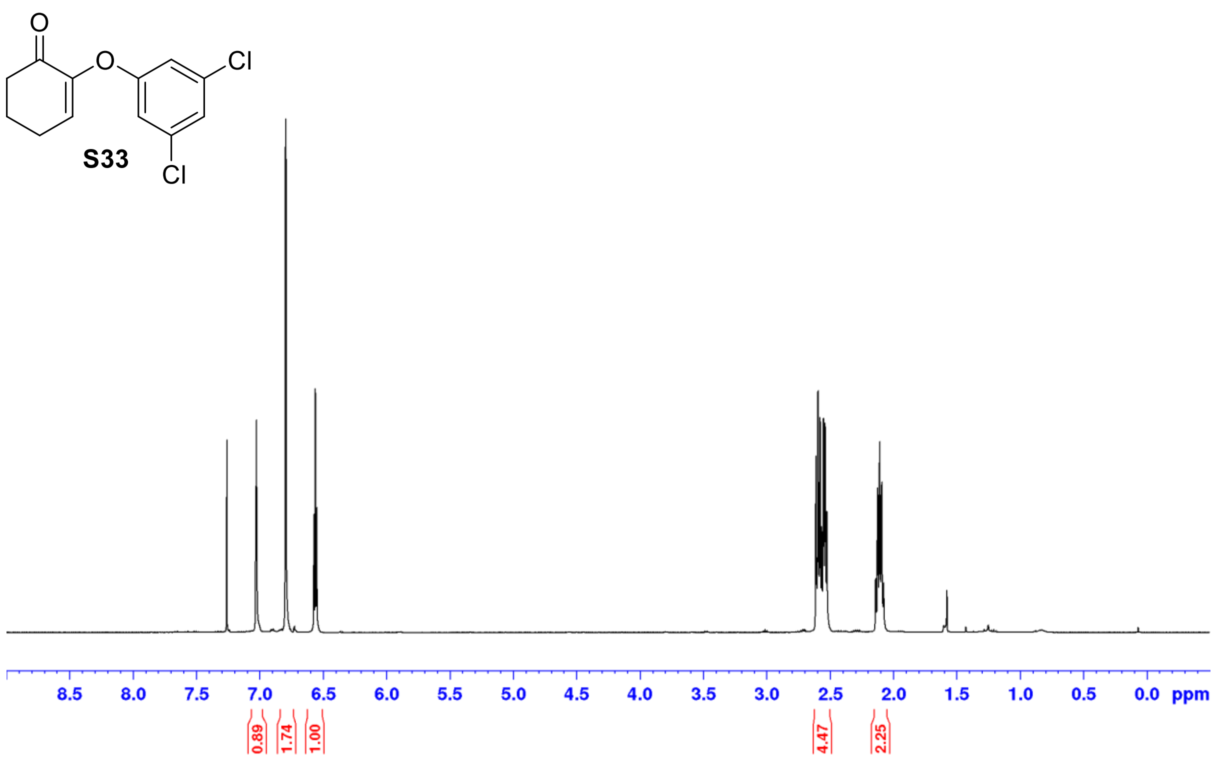
S33
O
O
Cl
Cl
8.5 8.0 7.5 7.0 6.5 6.0 5.5 5.0 4.5 4.0 3.5 3.0 2.5 2.0 1.5 1.0 0.5 0.0 ppm
0.89
1.74
1.00
4.47
2.25

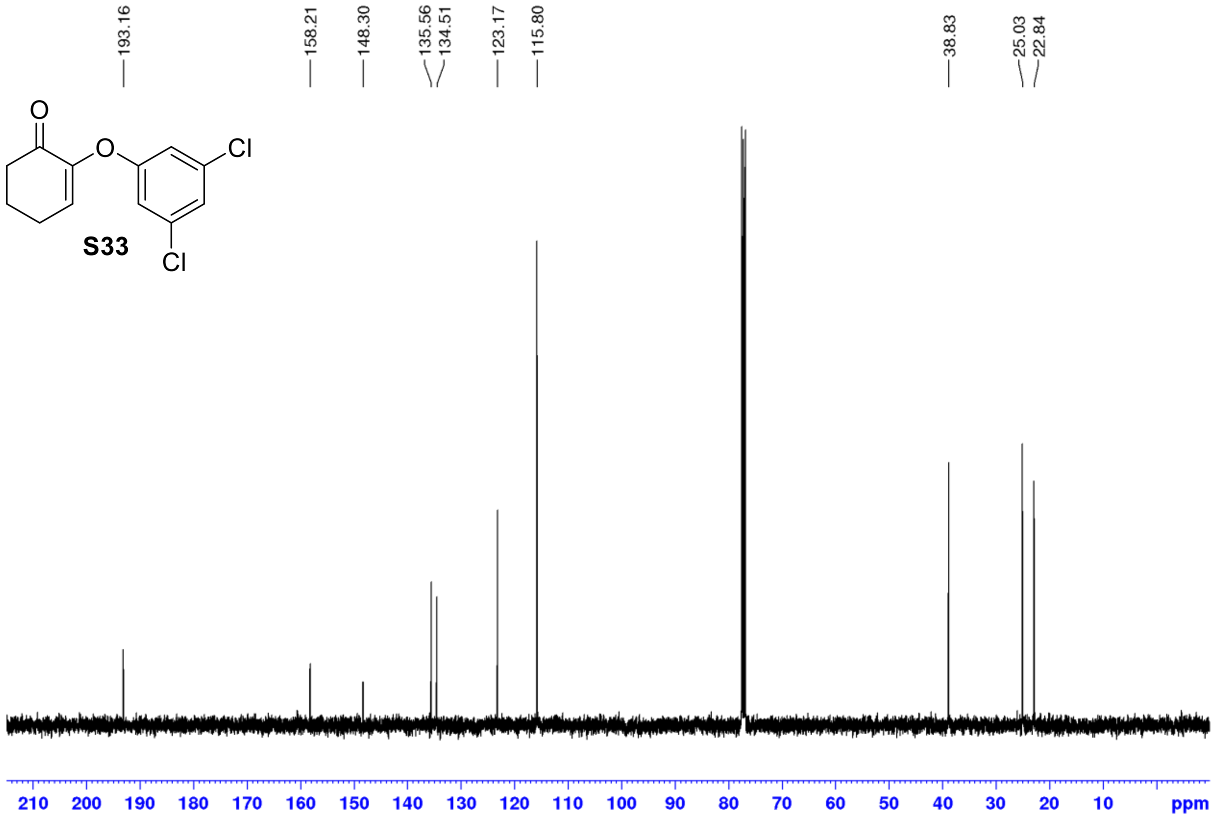
193.16
158.21
148.30
135.56
134.51
123.17
115.80
38.83
25.03
22.84
S33
O
O
Cl
Cl
210 200 190 180 170 160 150 140 130 120 110 100 90 80 70 60 50 40 30 20 10 ppm

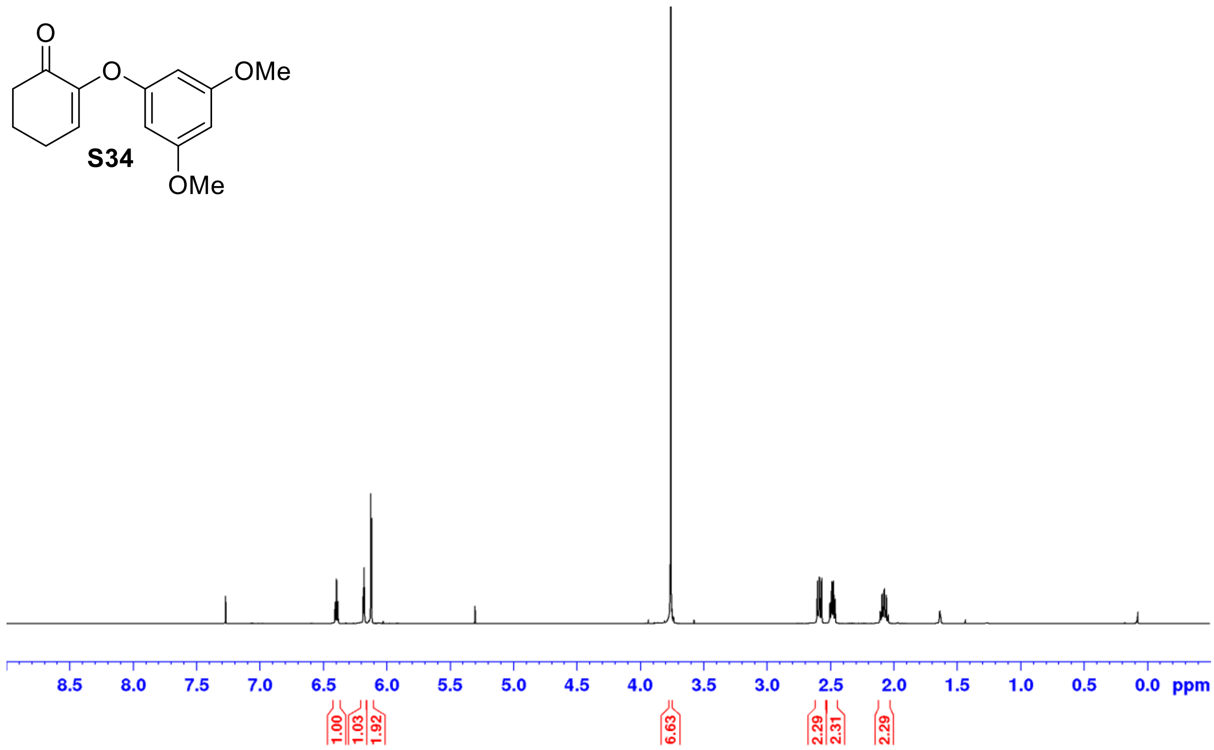
O
O
OMe
S34
OMe
8.5
8.0
7.5
7.0
6.5
6.0
5.5
5.0
4.5
4.0
3.5
3.0
2.5
2.0
1.5
1.0
0.5
0.0 ppm
1.00
1.03
1.92
6.63
2.29
2.31
2.29

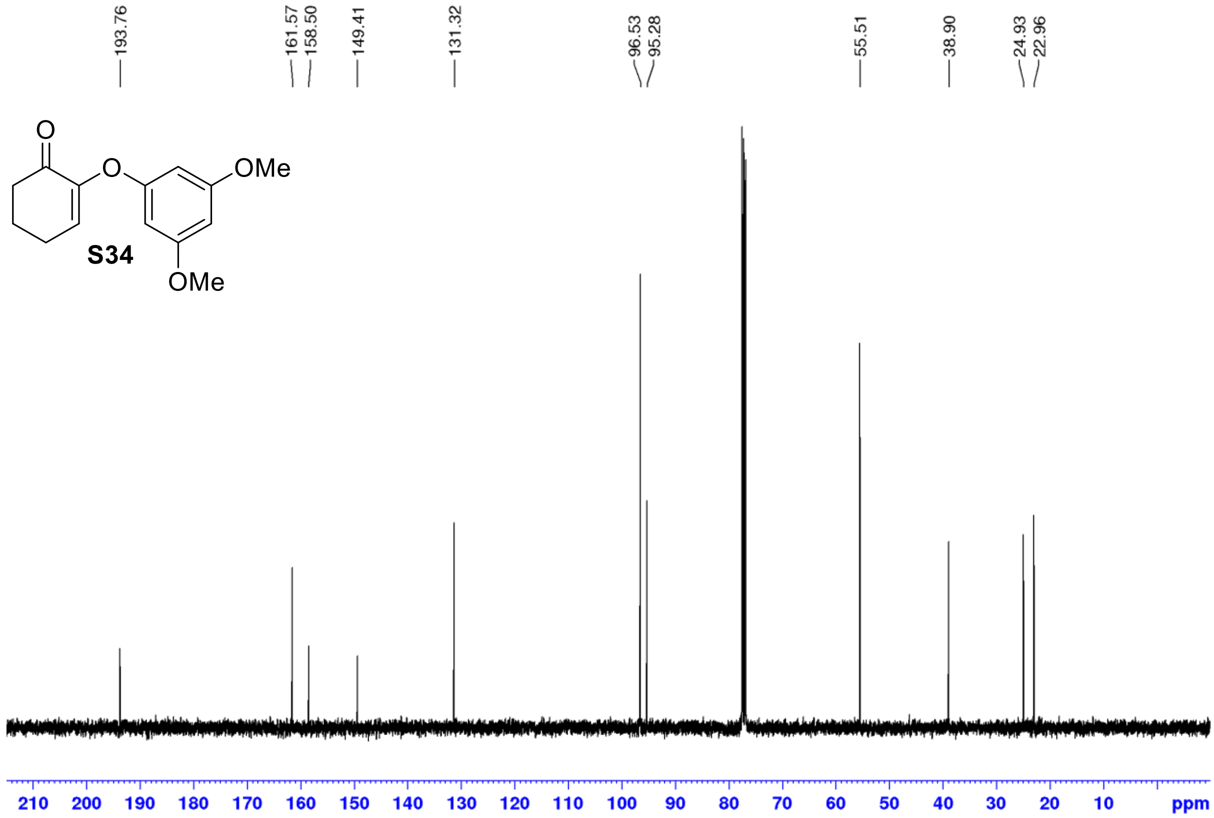
193.76
161.57
158.50
149.41
131.32
96.53
95.28
55.51
38.90
24.93
22.96
O
O
OMe
S34
OMe
210
200
190
180
170
160
150
140
130
120
110
100
90
80
70
60
50
40
30
20
10
ppm

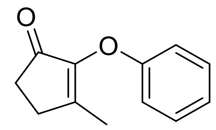

1b

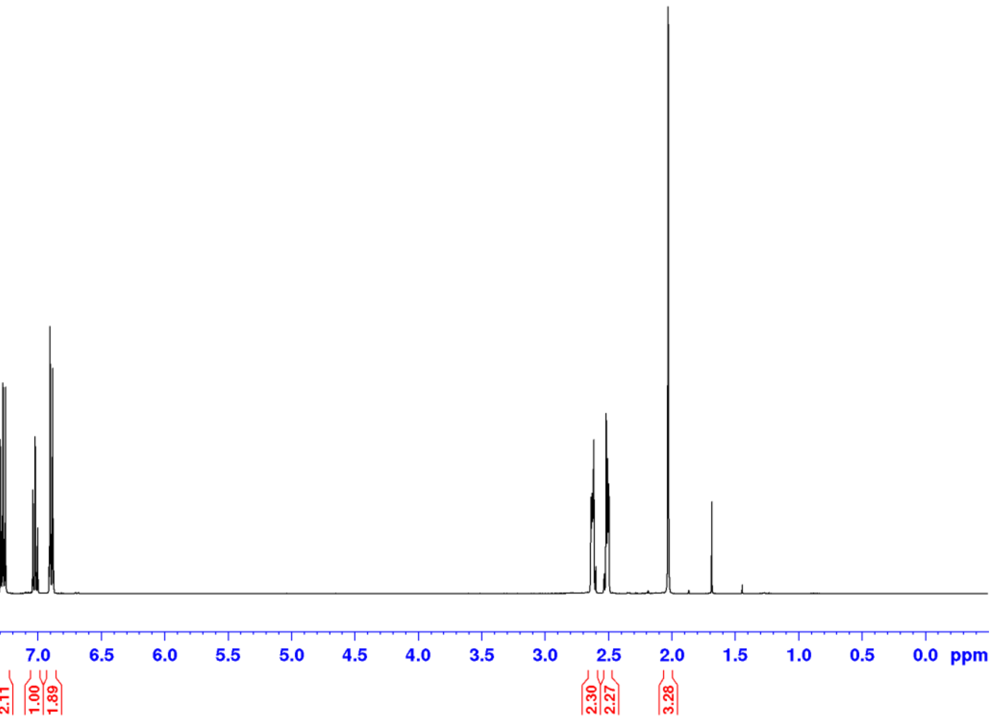

8.5
8.0
7.5
7.0
6.5
6.0
5.5
5.0
4.5
4.0
3.5
3.0
2.5
2.0
1.5
1.0
0.5
0.0
ppm
2.11
1.00
1.89
2.30
2.27
3.28

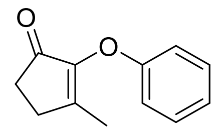

1b

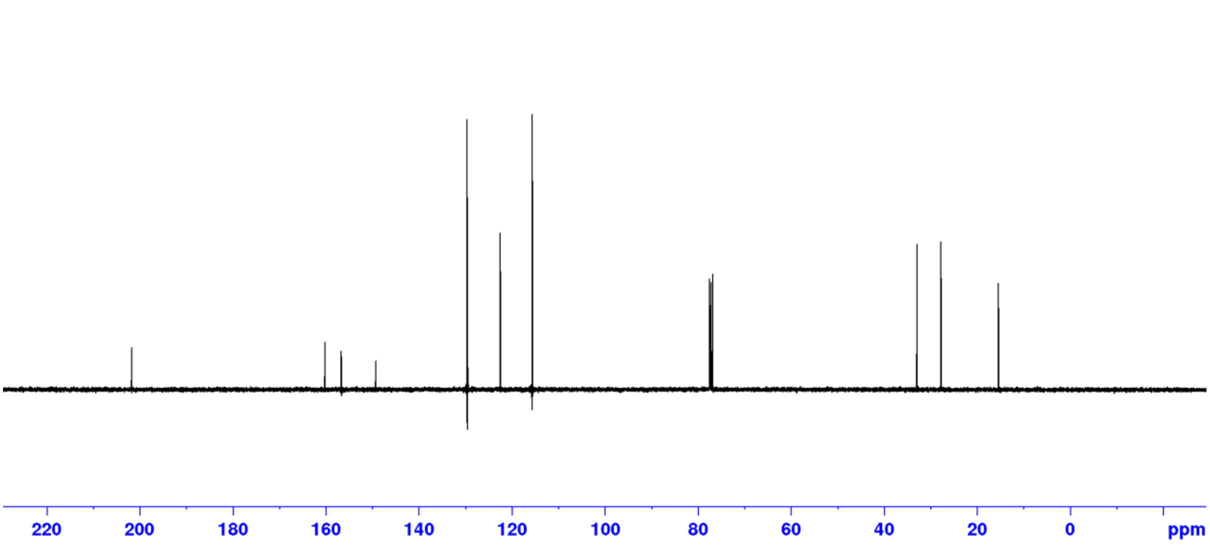

201.74
160.19
156.66
149.28
129.59
122.49
115.60
115.58
32.86
27.75
15.36
220
200
180
160
140
120
100
80
60
40
20
0
ppm

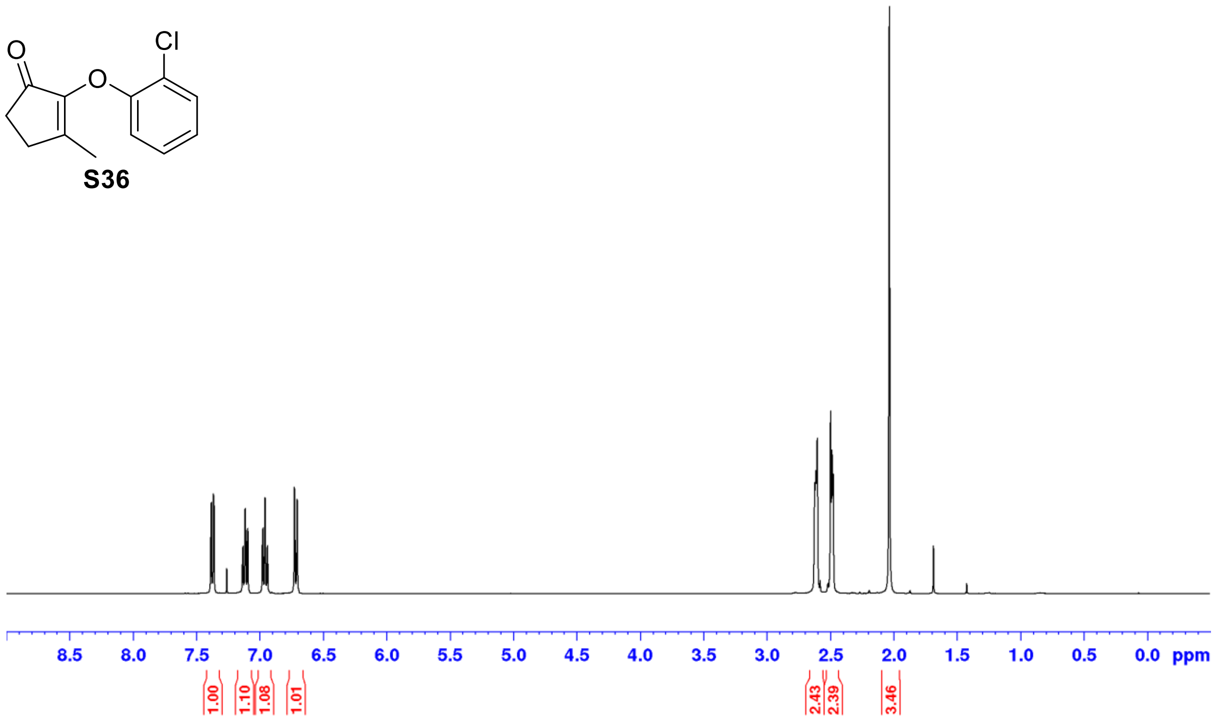

Cl
O
O
S36
8.5
8.0
7.5
7.0
6.5
6.0
5.5
5.0
4.5
4.0
3.5
3.0
2.5
2.0
1.5
1.0
0.5
0.0 ppm
1.00
1.10
1.08
1.01
2.43
2.39
3.46

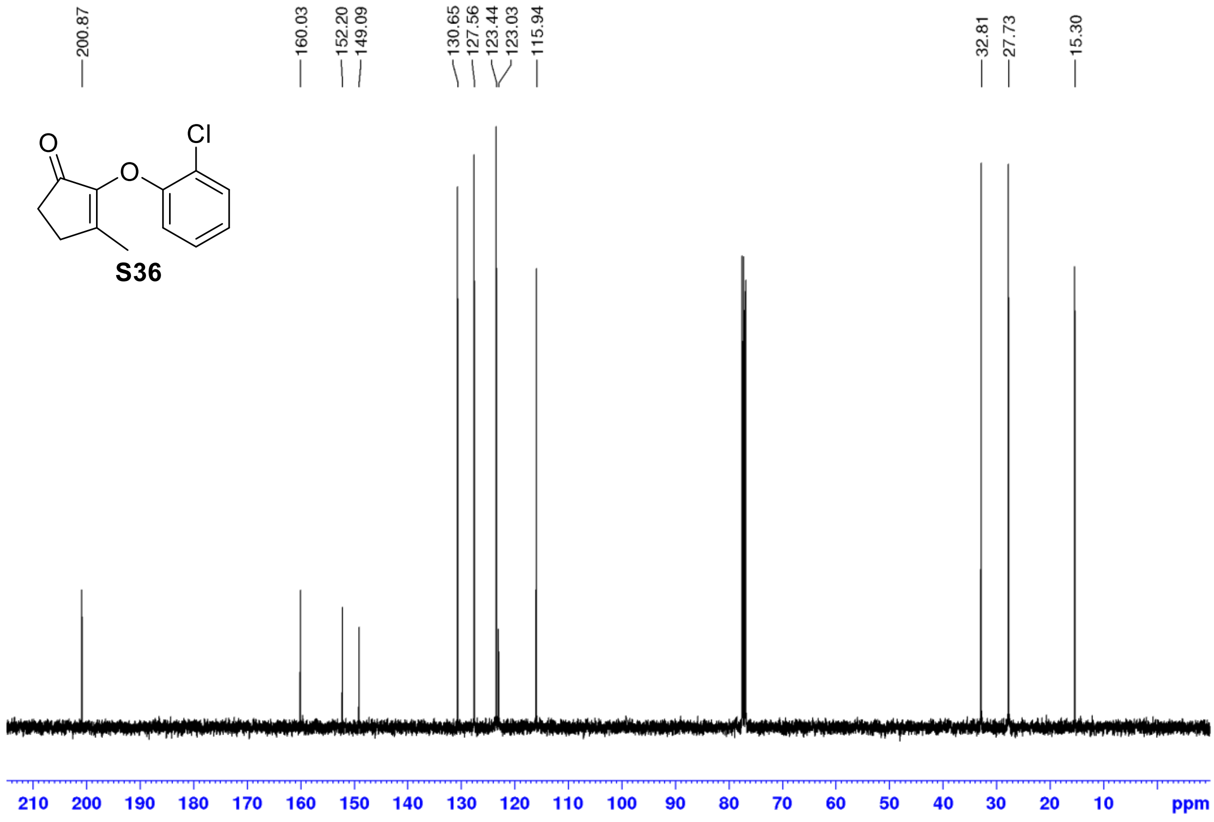

200.87
160.03
152.20
149.09
130.65
127.56
123.44
123.03
115.94
32.81
27.73
15.30
Cl
O
O
S36
210
200
190
180
170
160
150
140
130
120
110
100
90
80
70
60
50
40
30
20
10
ppm

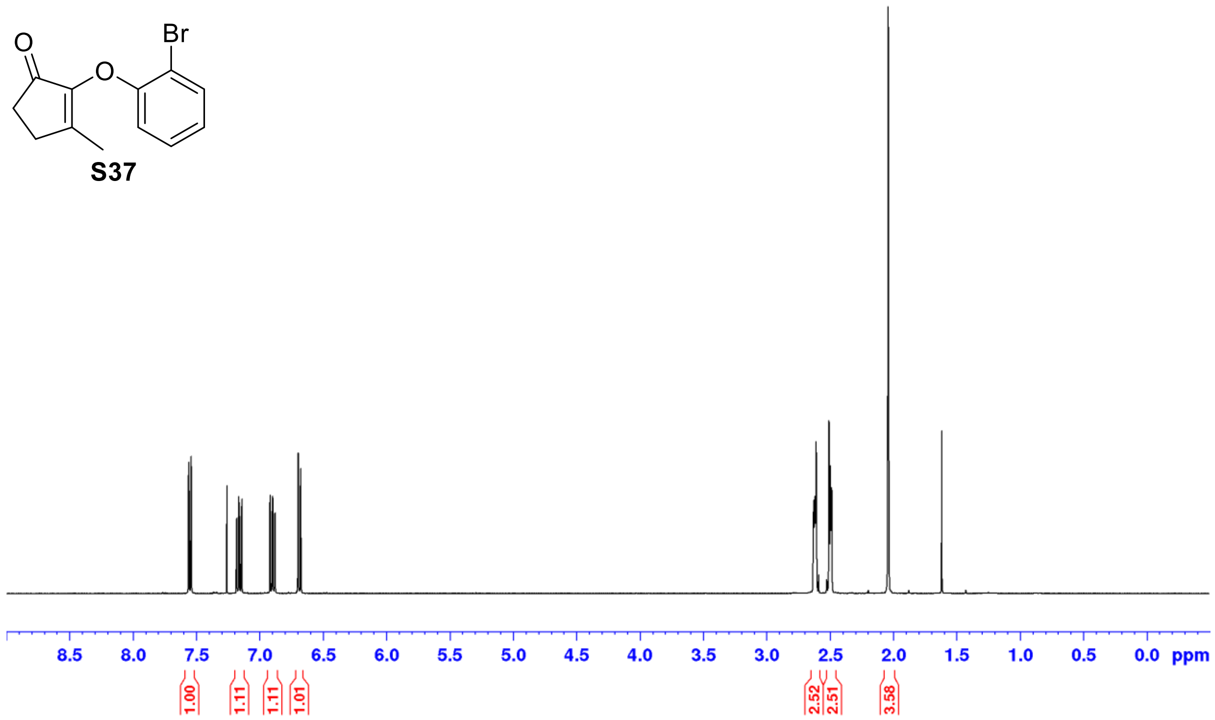
S37
Br
O
8.5 8.0 7.5 7.0 6.5 6.0 5.5 5.0 4.5 4.0 3.5 3.0 2.5 2.0 1.5 1.0 0.5 0.0 ppm
1.00 1.11 1.11 1.01 2.52 2.51 3.58

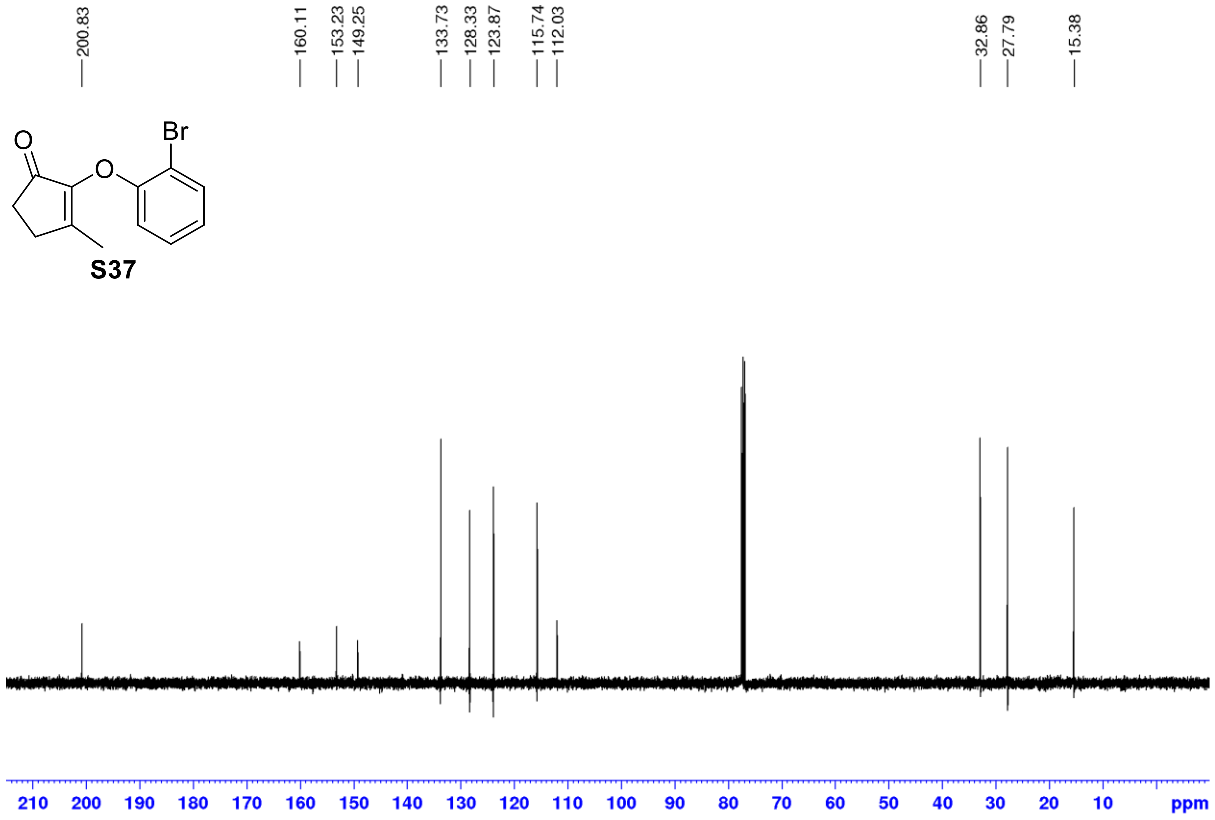
200.83 160.11 153.23 149.25 133.73 128.33 123.87 115.74 112.03 32.86 27.79 15.38
S37
Br
O
210 200 190 180 170 160 150 140 130 120 110 100 90 80 70 60 50 40 30 20 10 ppm

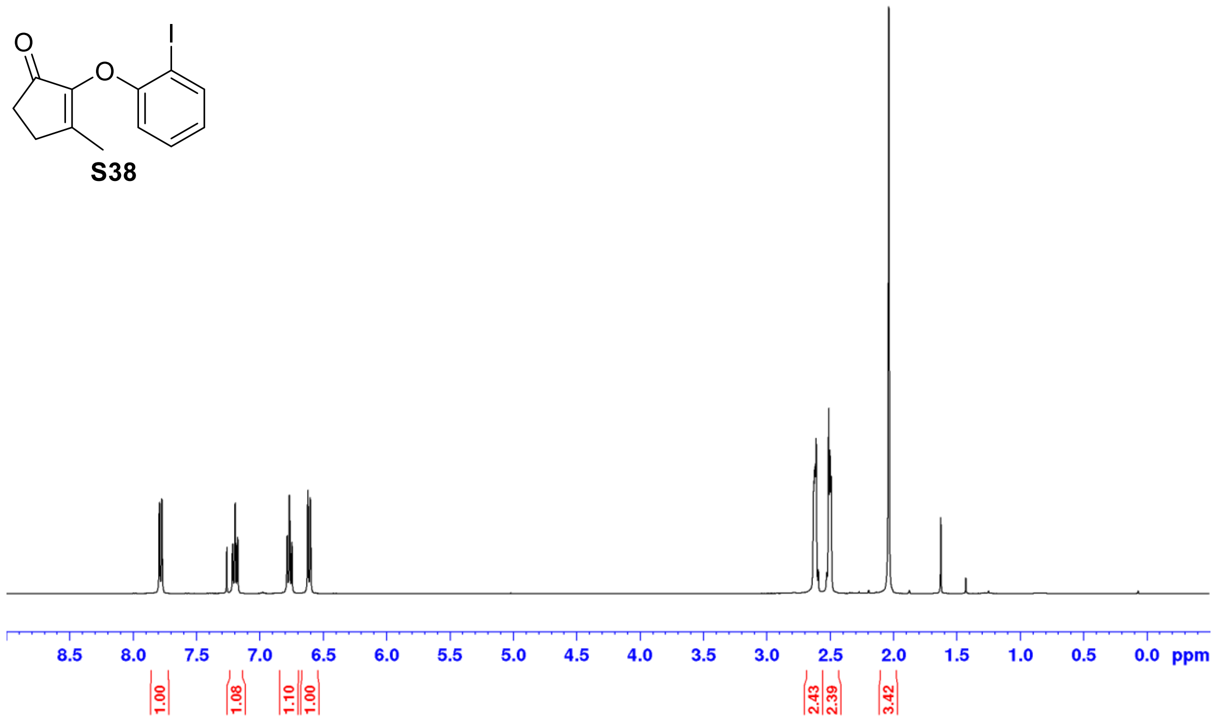
S38
8.5
8.0
7.5
7.0
6.5
6.0
5.5
5.0
4.5
4.0
3.5
3.0
2.5
2.0
1.5
1.0
0.5
0.0
ppm
1.00
1.08
1.10
1.00
2.43
2.39
3.42

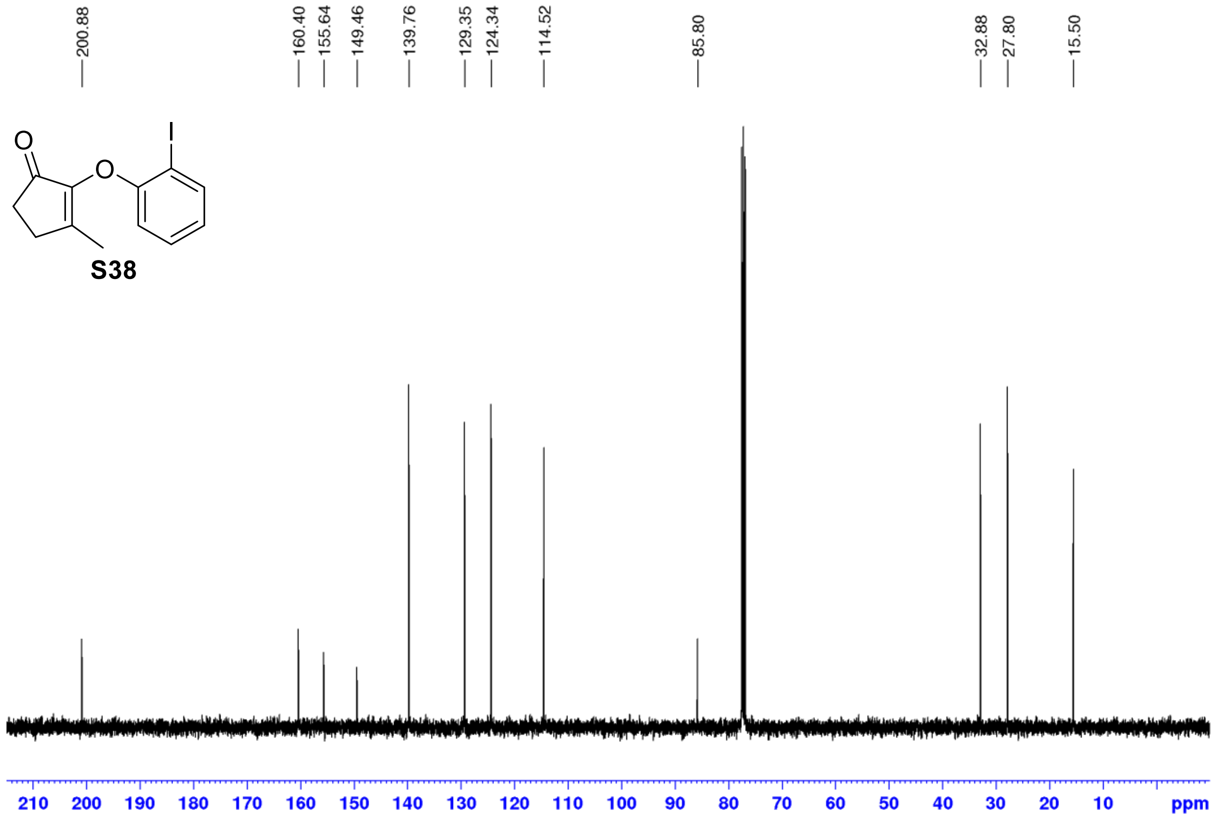
200.88
160.40
155.64
149.46
139.76
129.35
124.34
114.52
85.80
32.88
27.80
15.50
S38
210
200
190
180
170
160
150
140
130
120
110
100
90
80
70
60
50
40
30
20
10
ppm

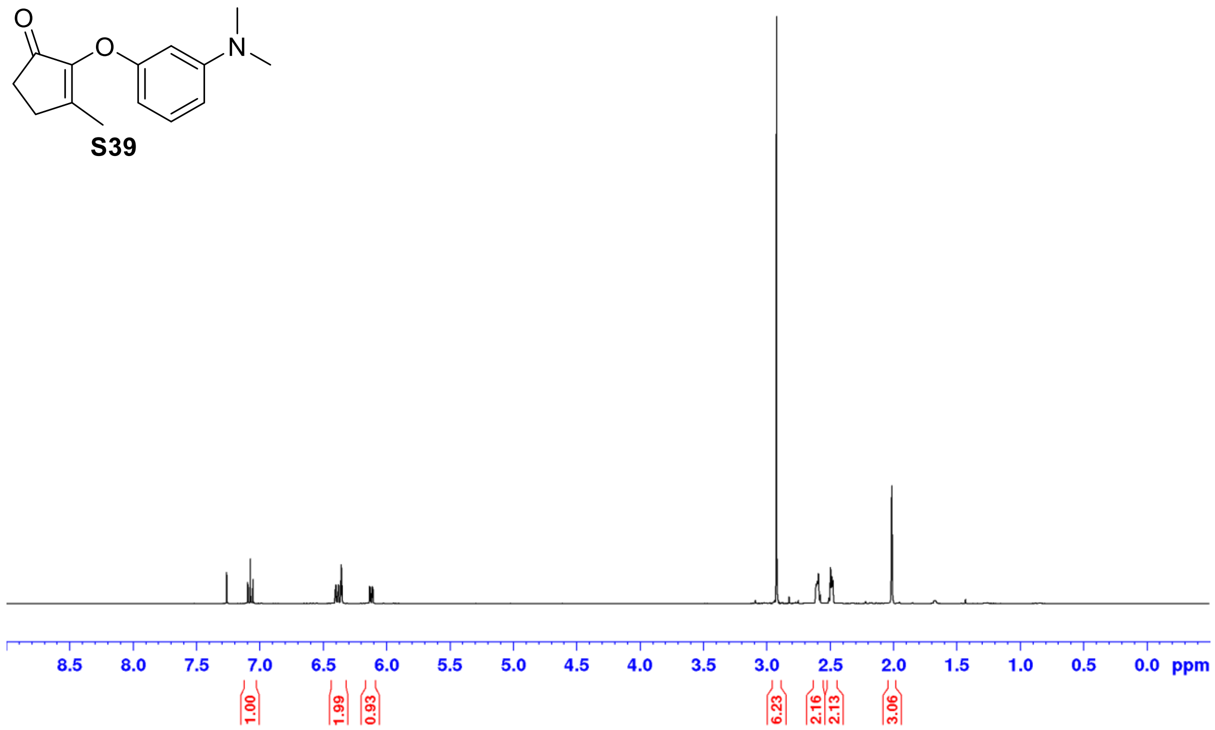
S39
8.5
8.0
7.5
7.0
6.5
6.0
5.5
5.0
4.5
4.0
3.5
3.0
2.5
2.0
1.5
1.0
0.5
0.0
ppm
1.00
1.99
0.93
6.23
2.16
2.13
3.06

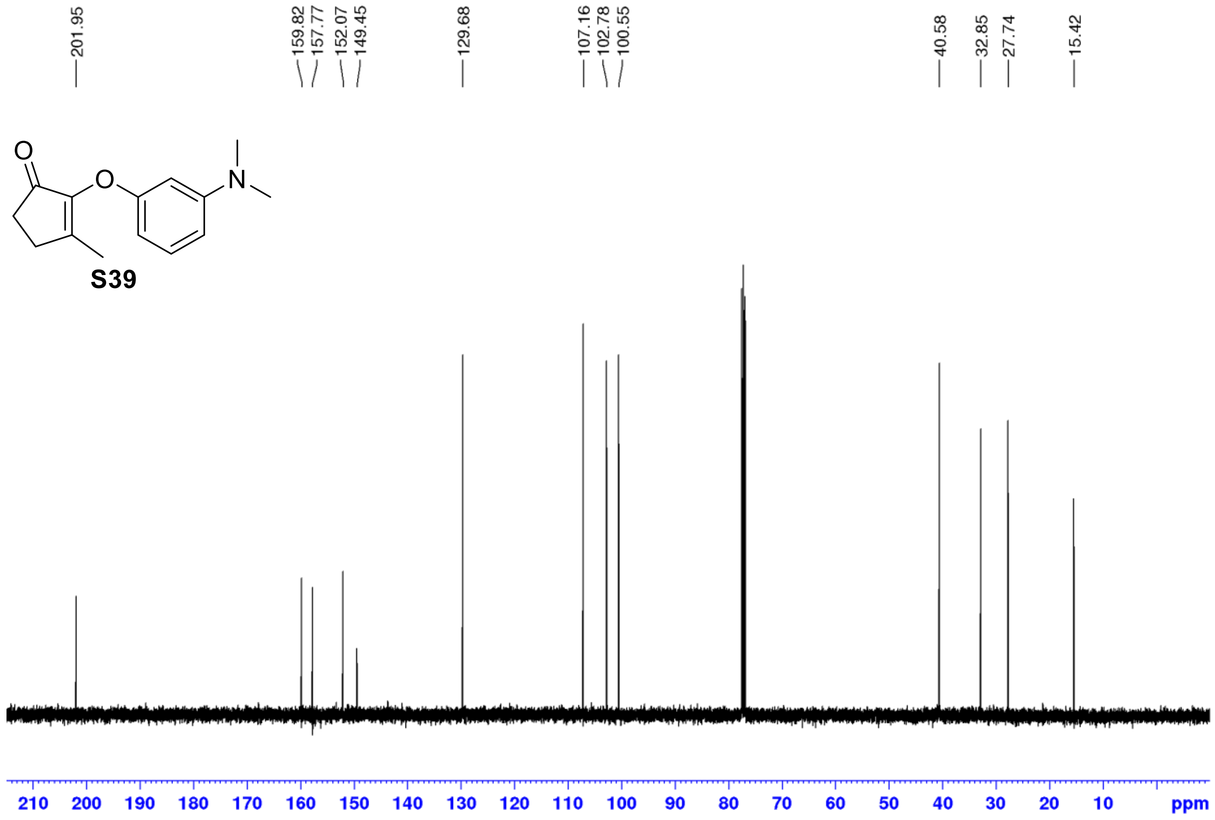
201.95
159.82
157.77
152.07
149.45
129.68
107.16
102.78
100.55
40.58
32.85
27.74
15.42
S39
210
200
190
180
170
160
150
140
130
120
110
100
90
80
70
60
50
40
30
20
10
ppm

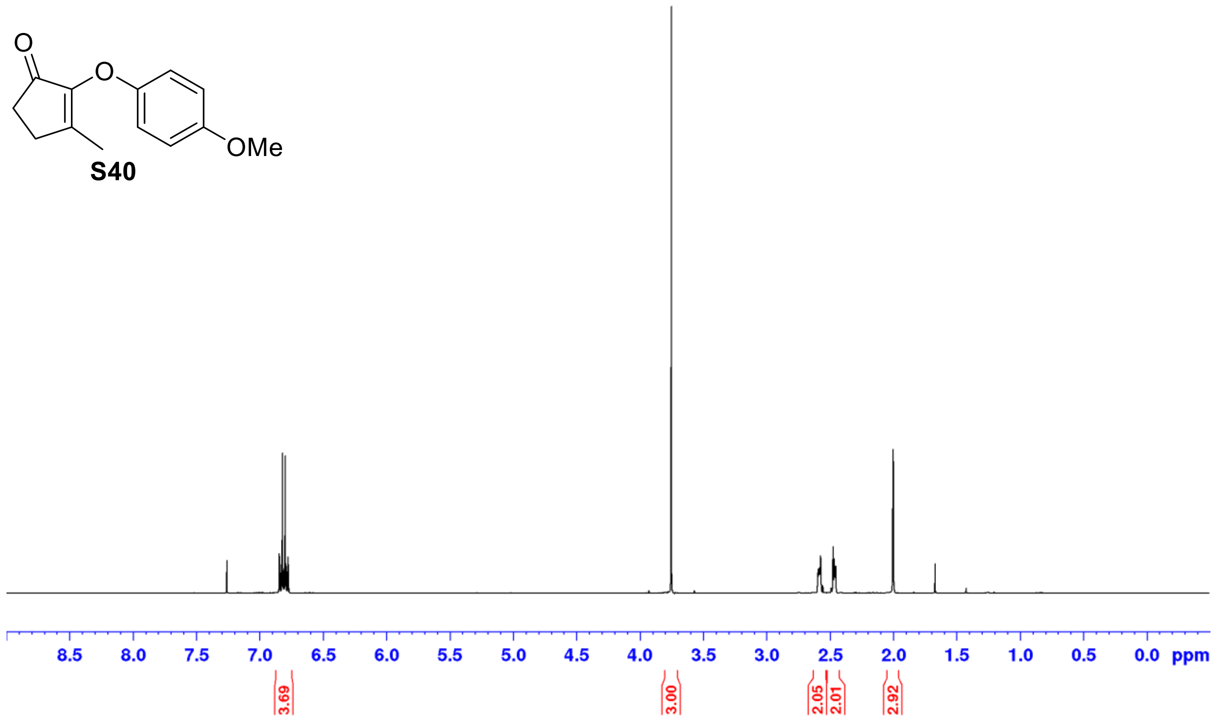

O
O
OMe
S40
8.5
8.0
7.5
7.0
6.5
6.0
5.5
5.0
4.5
4.0
3.5
3.0
2.5
2.0
1.5
1.0
0.5
0.0
ppm
3.69
3.00
2.05
2.01
2.92

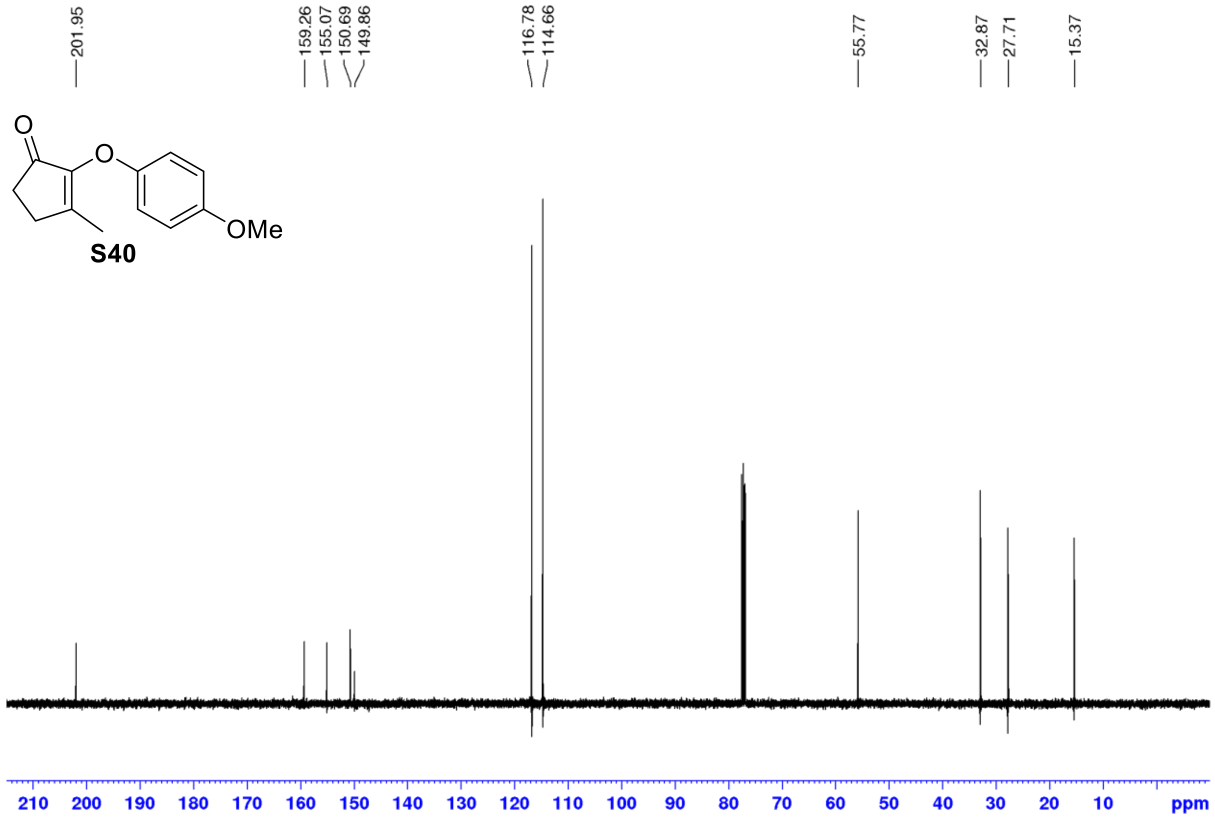

201.95
159.26
155.07
150.69
149.86
116.78
114.66
55.77
32.87
27.71
15.37
O
O
OMe
S40
210
200
190
180
170
160
150
140
130
120
110
100
90
80
70
60
50
40
30
20
10
ppm

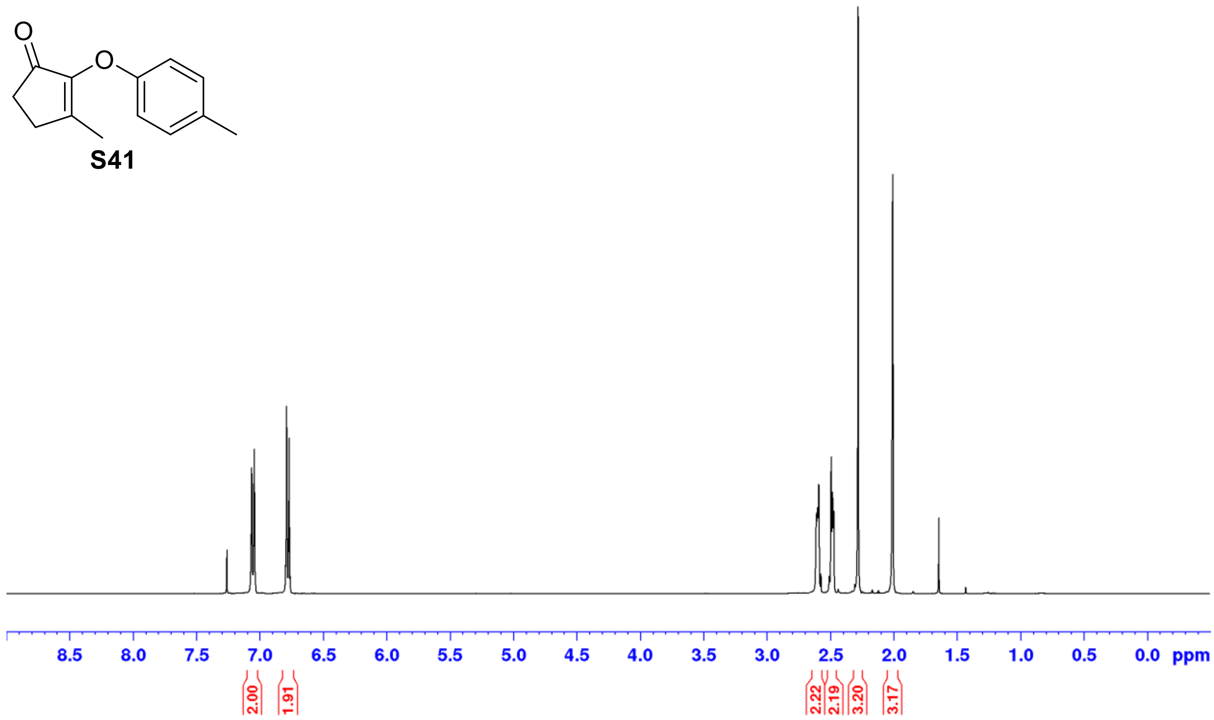
O
O
S41
8.5 8.0 7.5 7.0 6.5 6.0 5.5 5.0 4.5 4.0 3.5 3.0 2.5 2.0 1.5 1.0 0.5 0.0 ppm
2.00
1.91
2.22
2.19
3.20
3.17

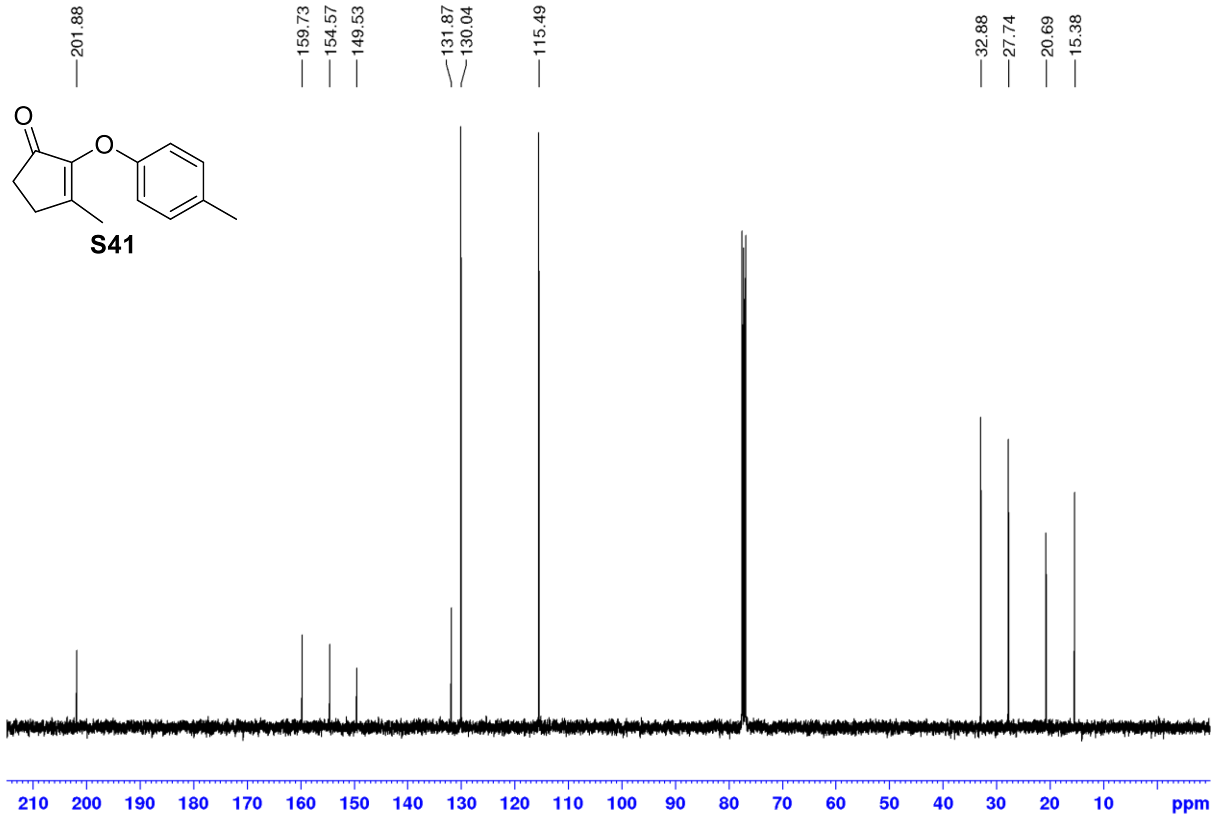
201.88
159.73
154.57
149.53
131.87
130.04
115.49
32.88
27.74
20.69
15.38
O
O
S41
210 200 190 180 170 160 150 140 130 120 110 100 90 80 70 60 50 40 30 20 10 ppm

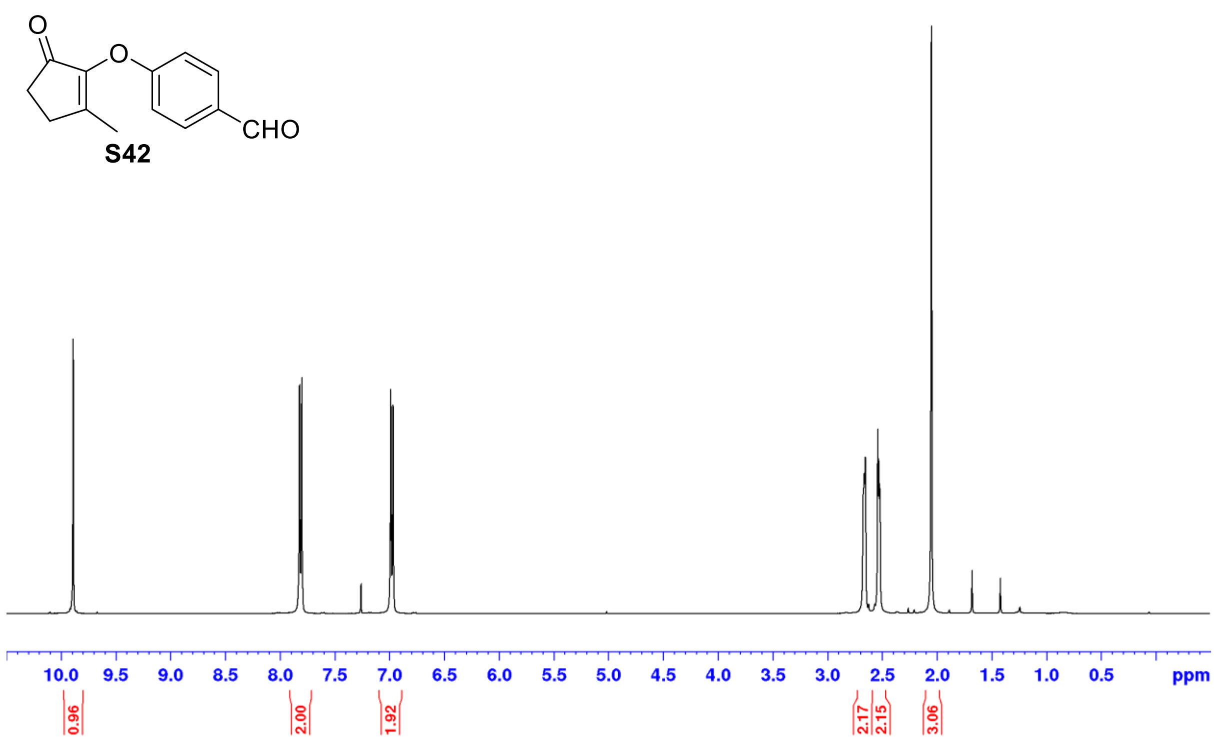
S42
CHO
ppm
0.96
2.00
1.92
2.17
2.15
3.06

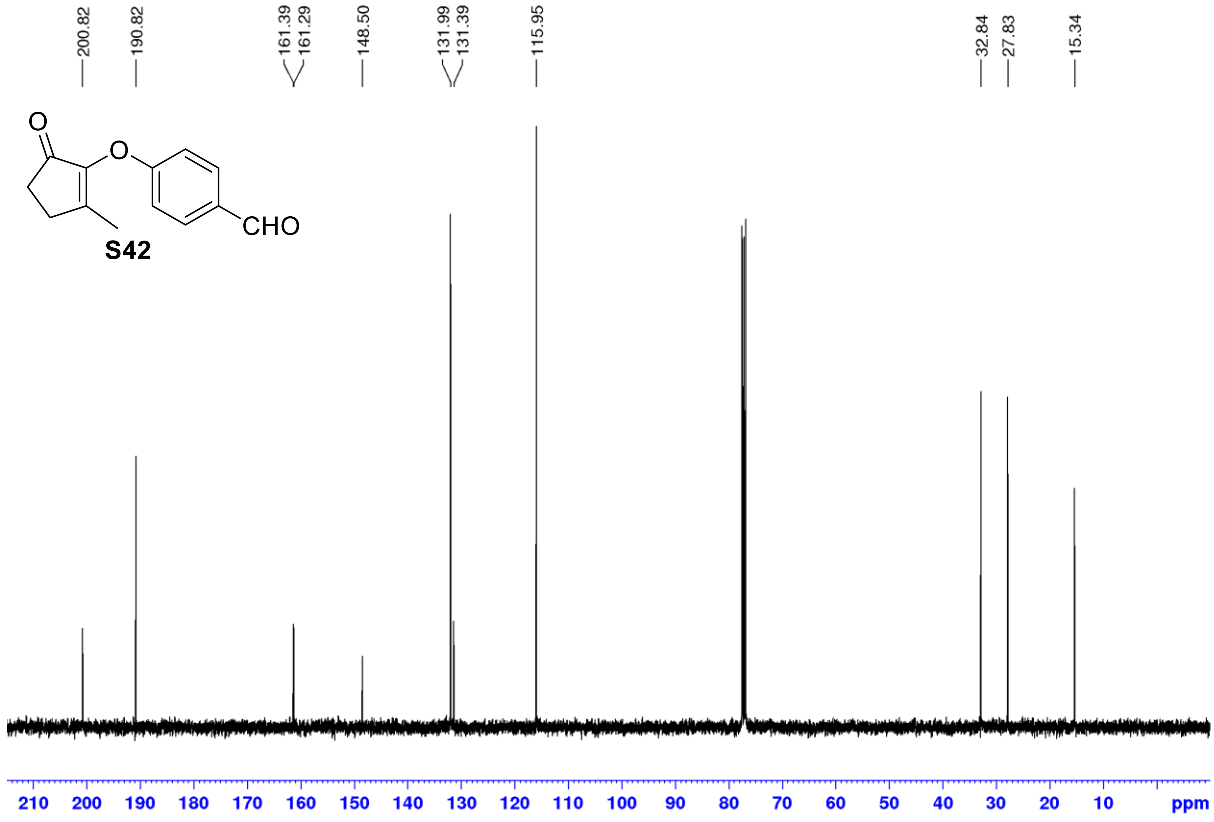
200.82
190.82
161.39
161.29
148.50
131.99
131.39
115.95
32.84
27.83
15.34
S42
CHO
ppm

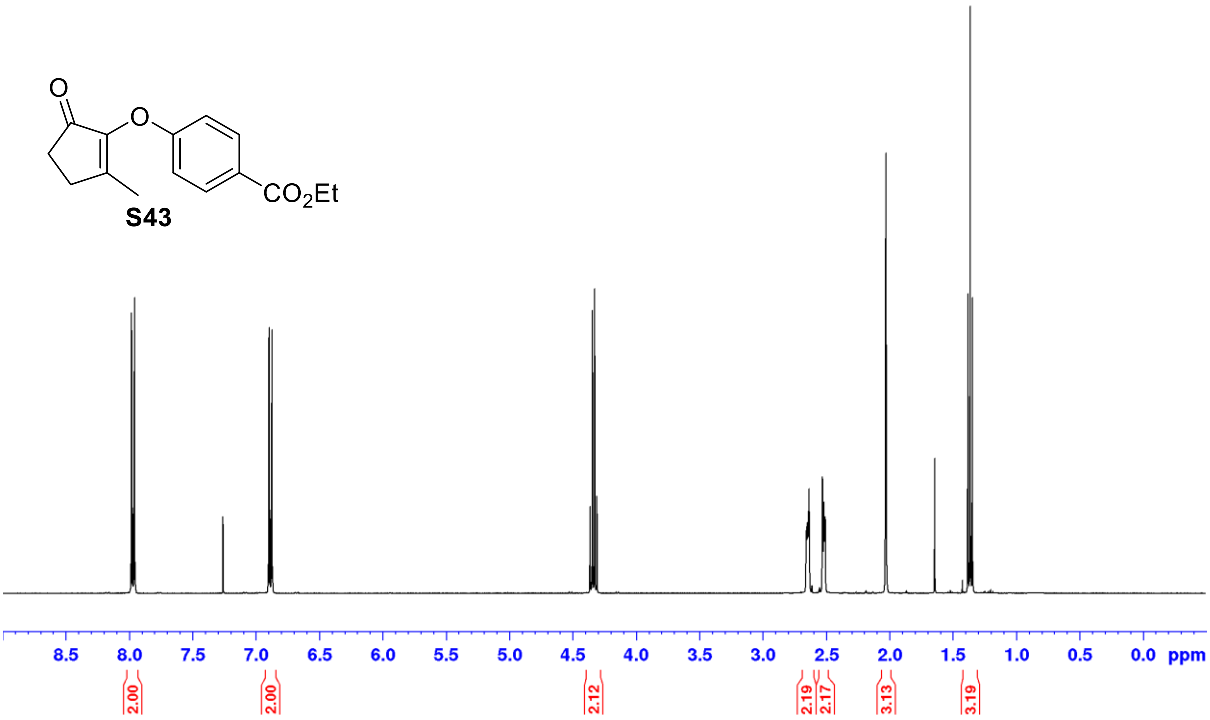
O
O
CO2Et
S43
8.5 8.0 7.5 7.0 6.5 6.0 5.5 5.0 4.5 4.0 3.5 3.0 2.5 2.0 1.5 1.0 0.5 0.0 ppm
2.00
2.00
2.12
2.19
2.17
3.13
3.19

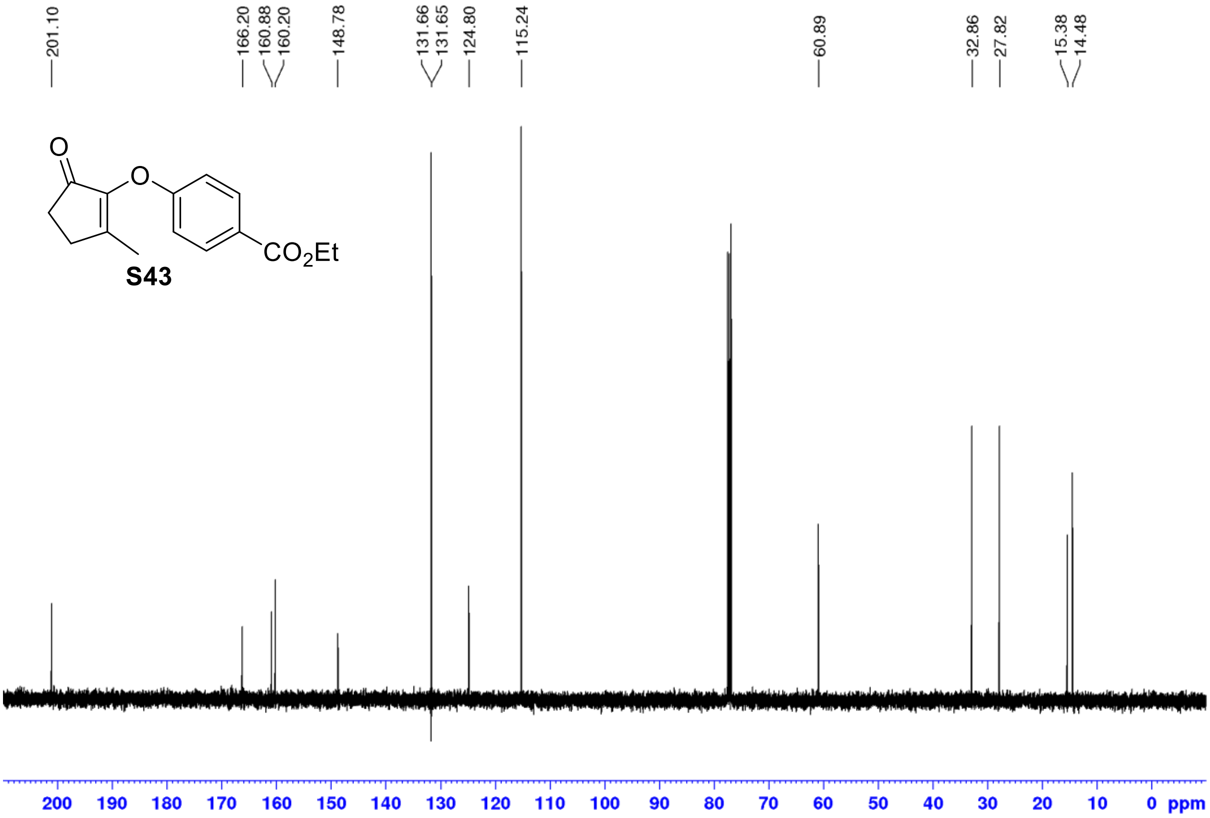
201.10
166.20
160.88
160.20
148.78
131.66
131.65
124.80
115.24
60.89
32.86
27.82
15.38
14.48
O
O
CO2Et
S43
200 190 180 170 160 150 140 130 120 110 100 90 80 70 60 50 40 30 20 10 0 ppm

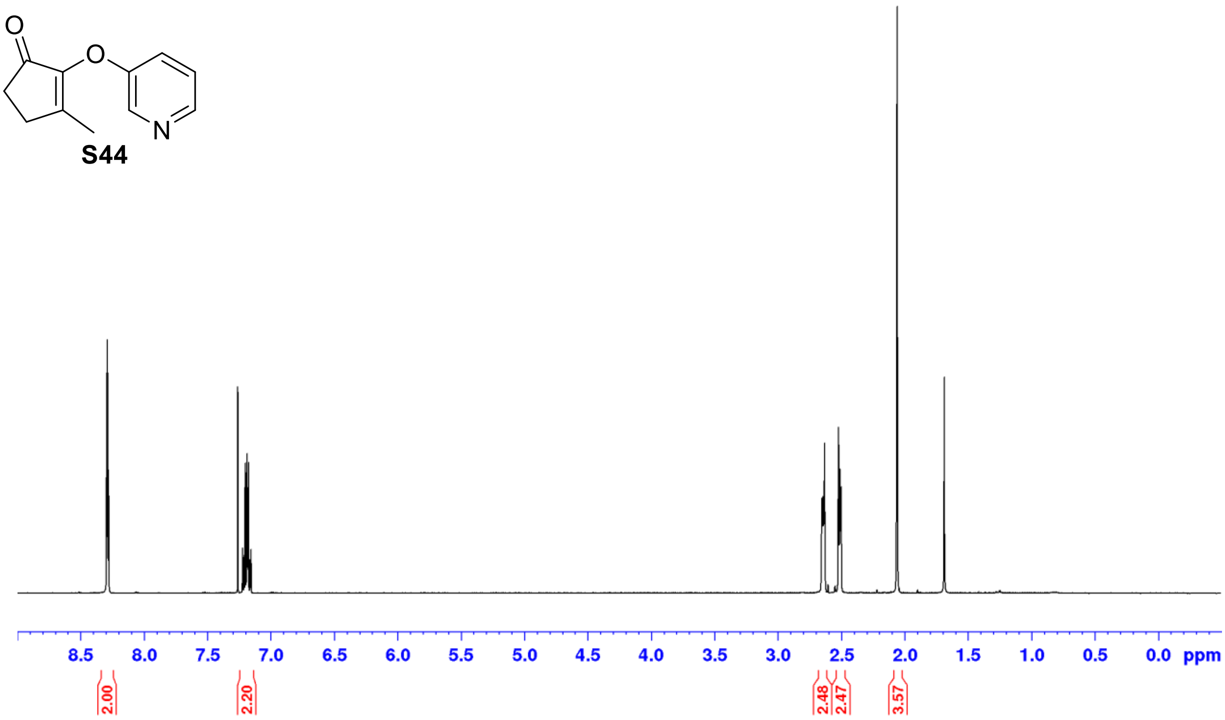
O
O
N
S44
8.5 8.0 7.5 7.0 6.5 6.0 5.5 5.0 4.5 4.0 3.5 3.0 2.5 2.0 1.5 1.0 0.5 0.0 ppm
2.00
2.20
2.48
2.47
3.57

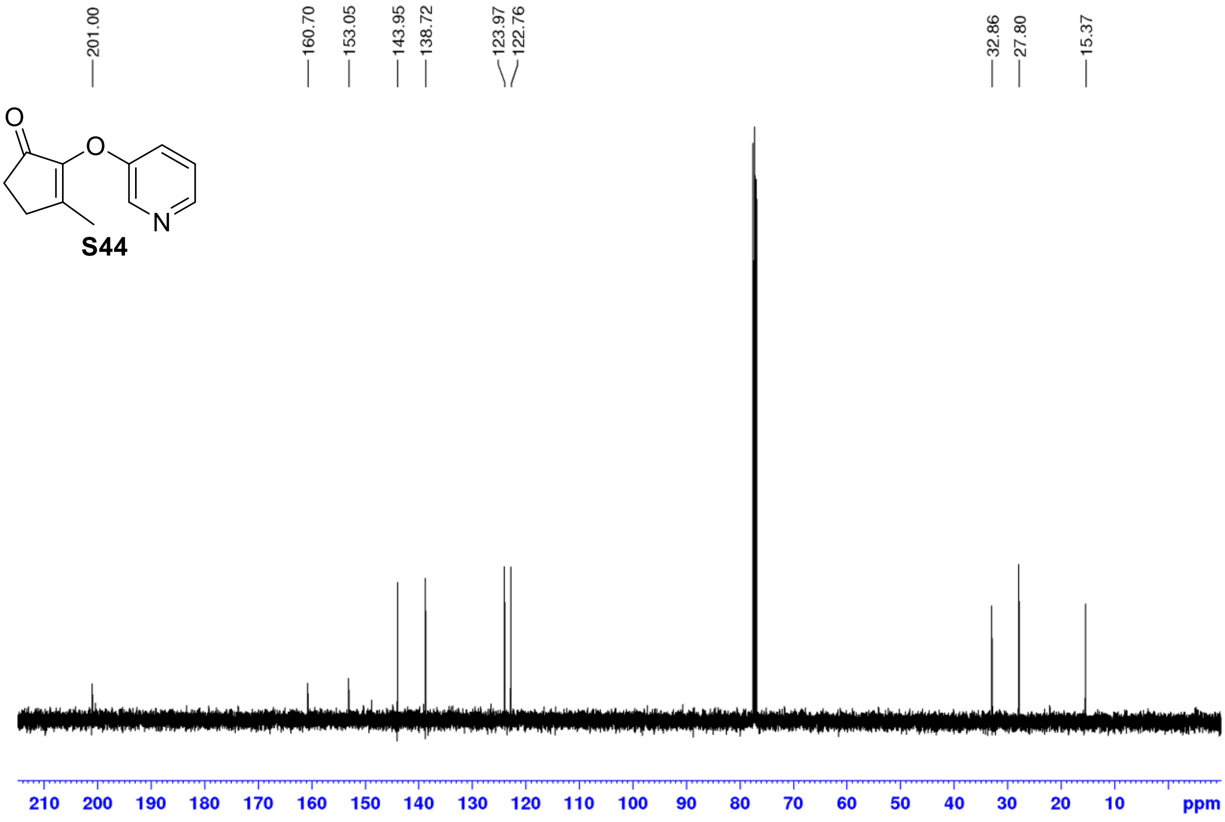
201.00
160.70
153.05
143.95
138.72
123.97
122.76
32.86
27.80
15.37
O
O
N
S44
210 200 190 180 170 160 150 140 130 120 110 100 90 80 70 60 50 40 30 20 10 ppm

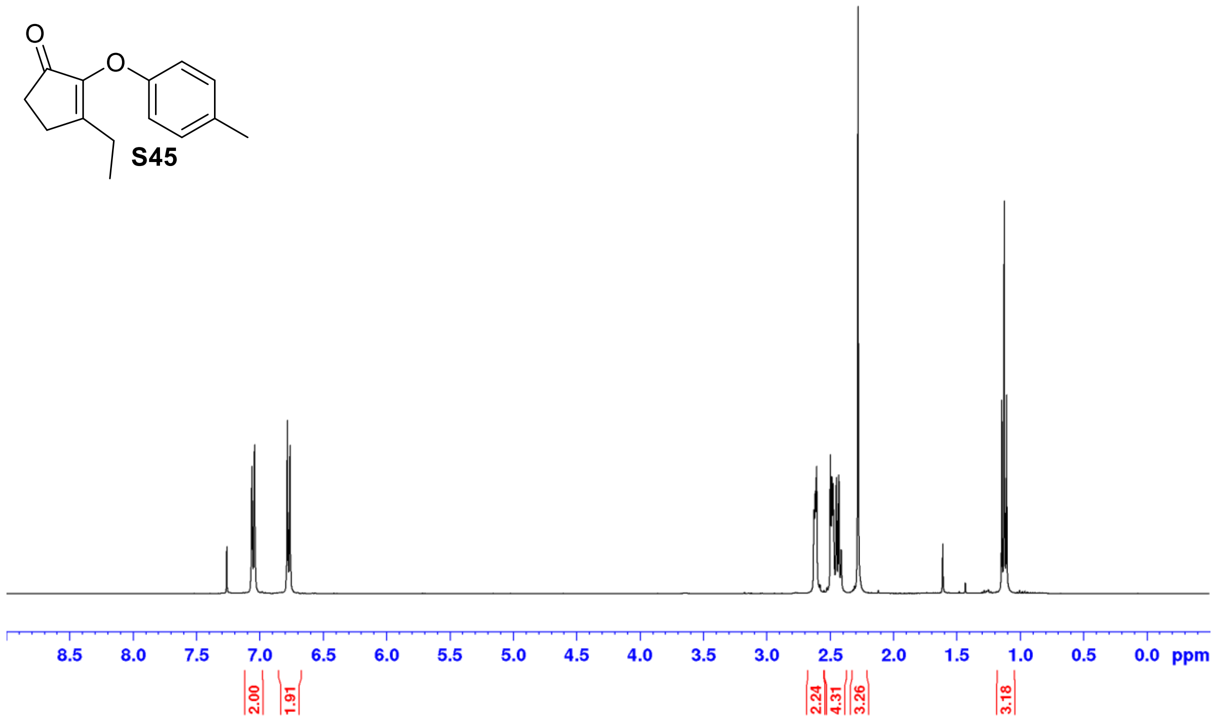

S45
8.5
8.0
7.5
7.0
6.5
6.0
5.5
5.0
4.5
4.0
3.5
3.0
2.5
2.0
1.5
1.0
0.5
0.0
ppm
2.00
1.91
2.24
4.31
3.26
3.18

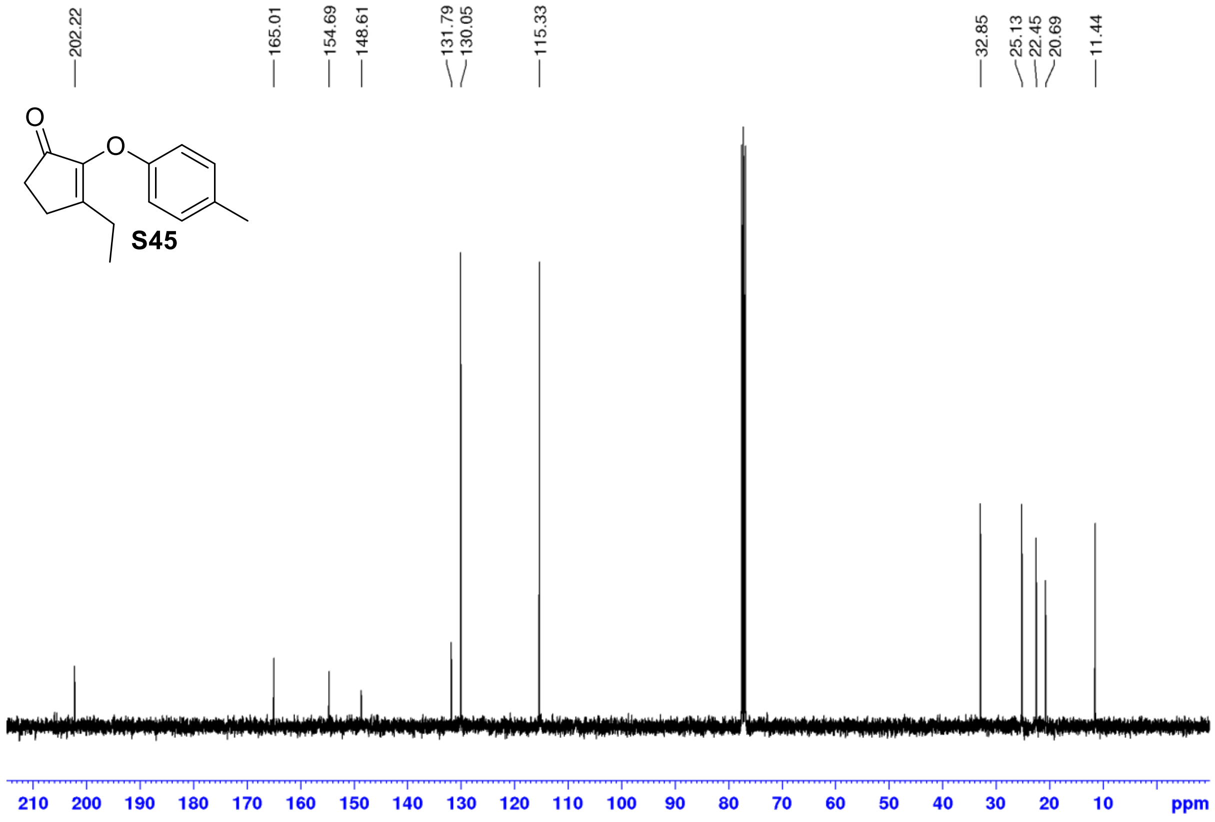

202.22
165.01
154.69
148.61
131.79
130.05
115.33
32.85
25.13
22.45
20.69
11.44
S45
210
200
190
180
170
160
150
140
130
120
110
100
90
80
70
60
50
40
30
20
10
ppm

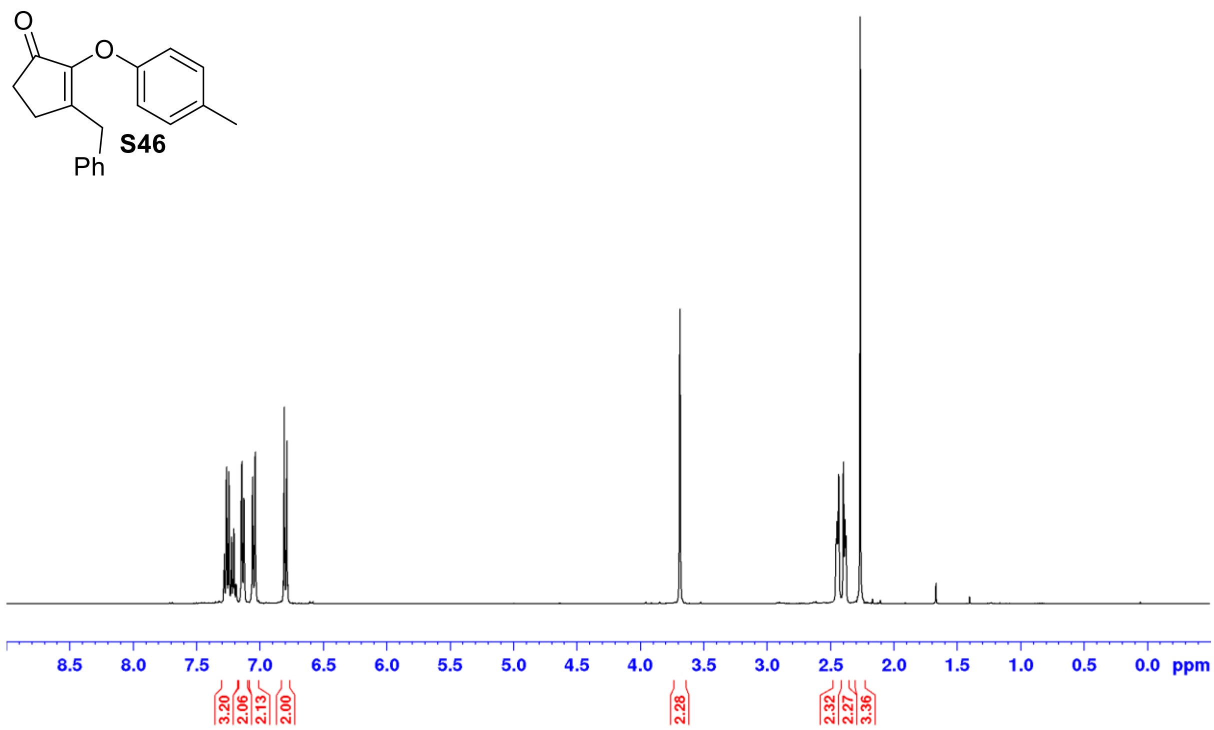
O
O
S46
Ph
8.5
8.0
7.5
7.0
6.5
6.0
5.5
5.0
4.5
4.0
3.5
3.0
2.5
2.0
1.5
1.0
0.5
0.0
ppm
3.20
2.06
2.13
2.00
2.28
2.32
2.27
3.36

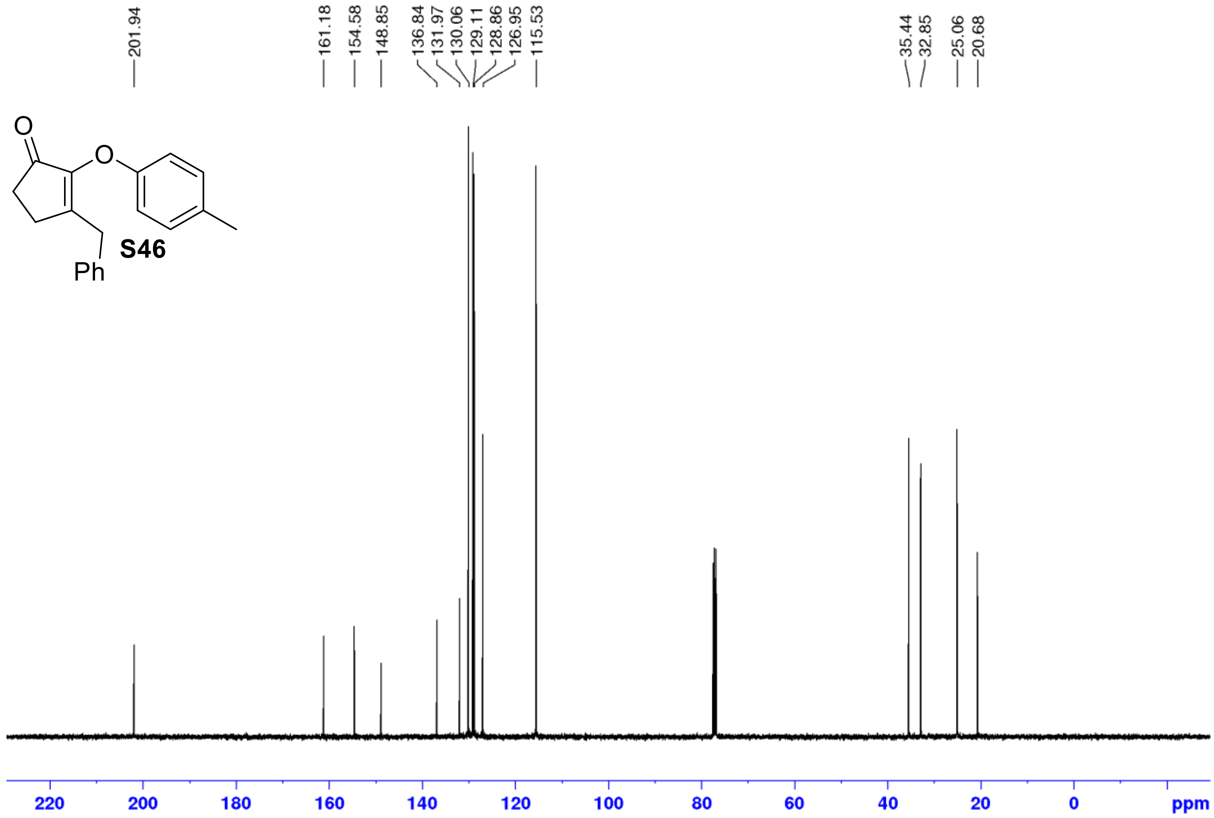
201.94
161.18
154.58
148.85
136.84
131.97
130.06
129.11
128.86
126.95
115.53
35.44
32.85
25.06
20.68
O
O
S46
Ph
220
200
180
160
140
120
100
80
60
40
20
0
ppm

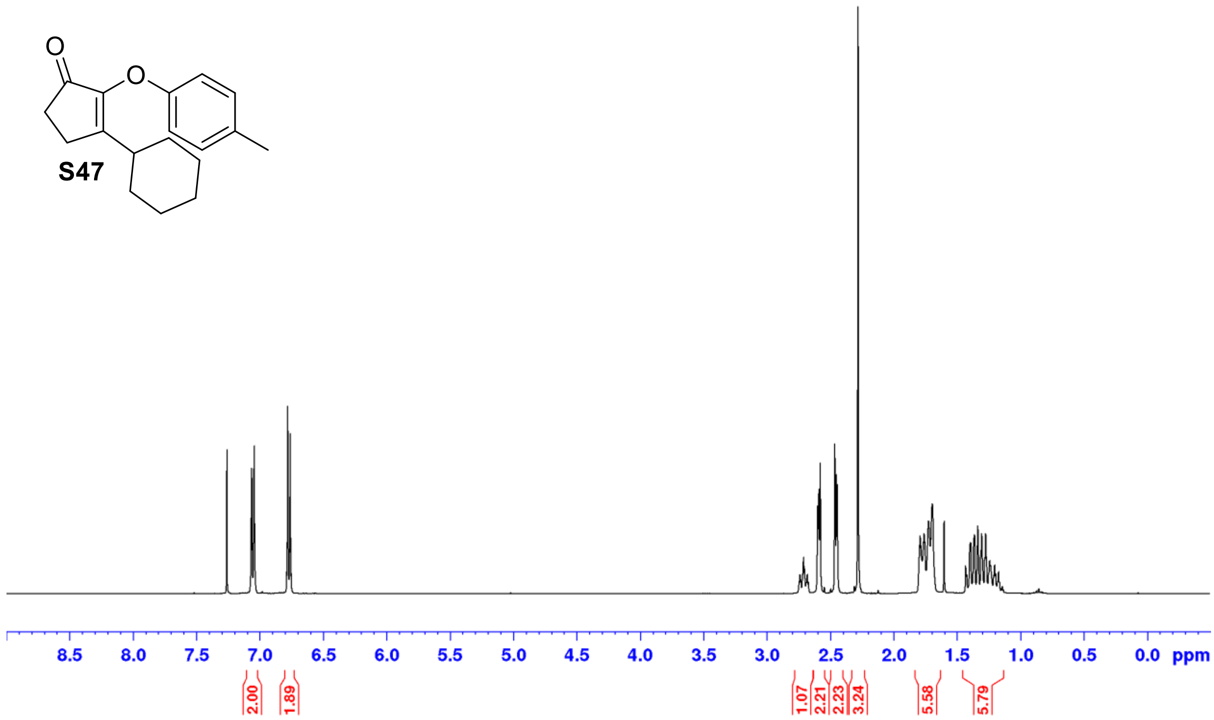
O
O
S47
8.5 8.0 7.5 7.0 6.5 6.0 5.5 5.0 4.5 4.0 3.5 3.0 2.5 2.0 1.5 1.0 0.5 0.0 ppm
2.00 1.89 1.07 2.21 2.23 3.24 5.58 5.79

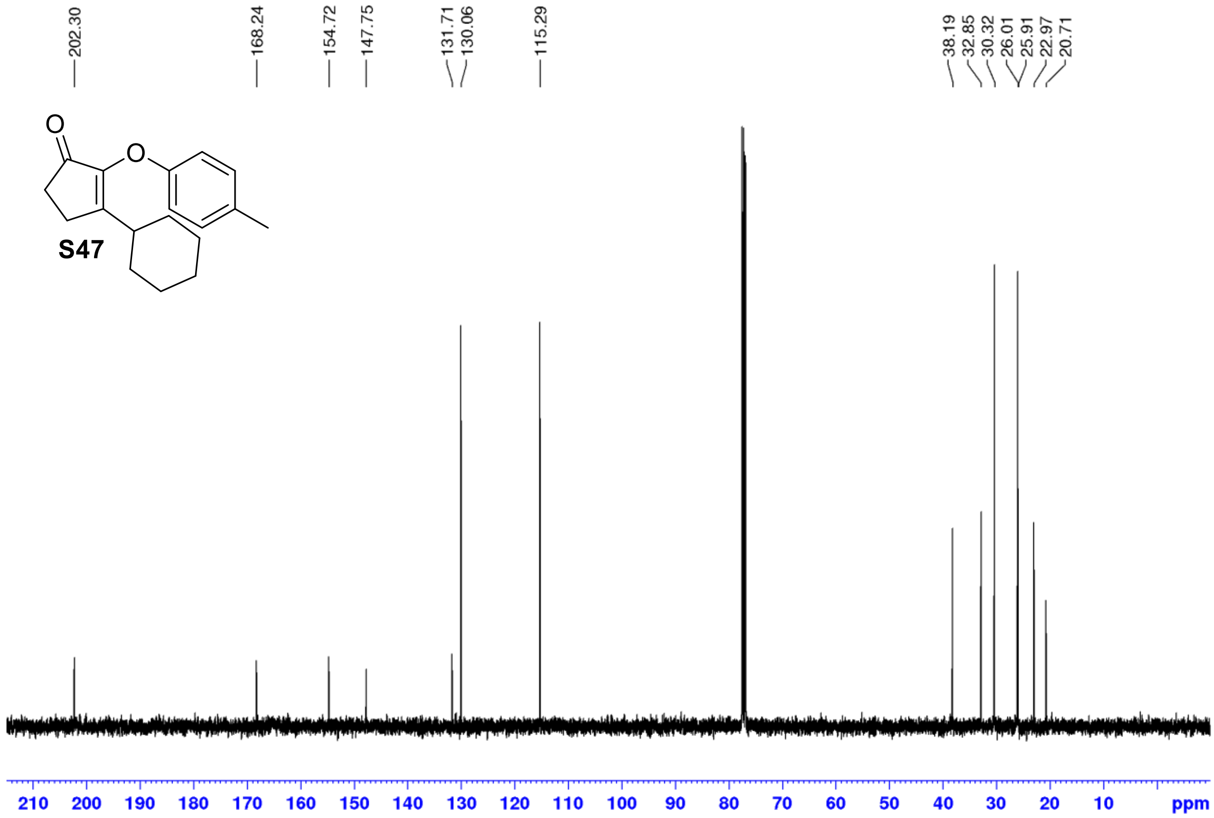
202.30
168.24
154.72
147.75
131.71
130.06
115.29
38.19
32.85
30.32
26.01
25.91
22.97
20.71
O
O
S47
210 200 190 180 170 160 150 140 130 120 110 100 90 80 70 60 50 40 30 20 10 ppm

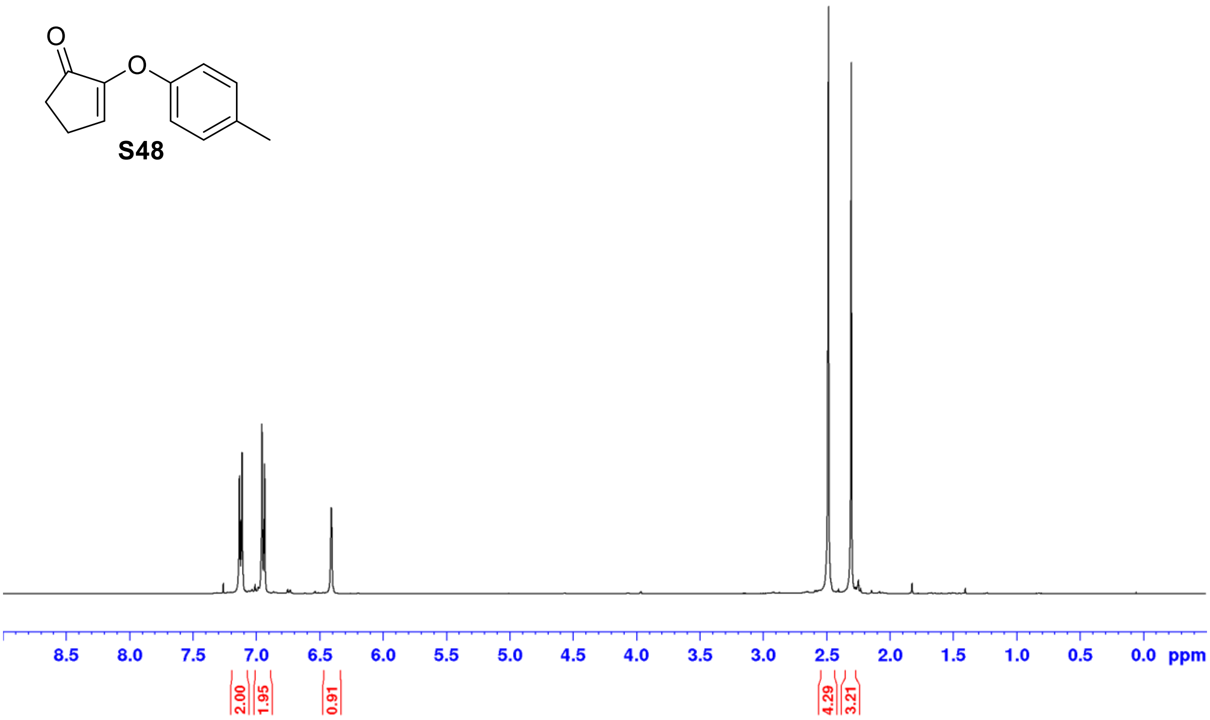
O
O
S48
8.5
8.0
7.5
7.0
6.5
6.0
5.5
5.0
4.5
4.0
3.5
3.0
2.5
2.0
1.5
1.0
0.5
0.0
ppm
2.00
1.95
0.91
4.29
3.21

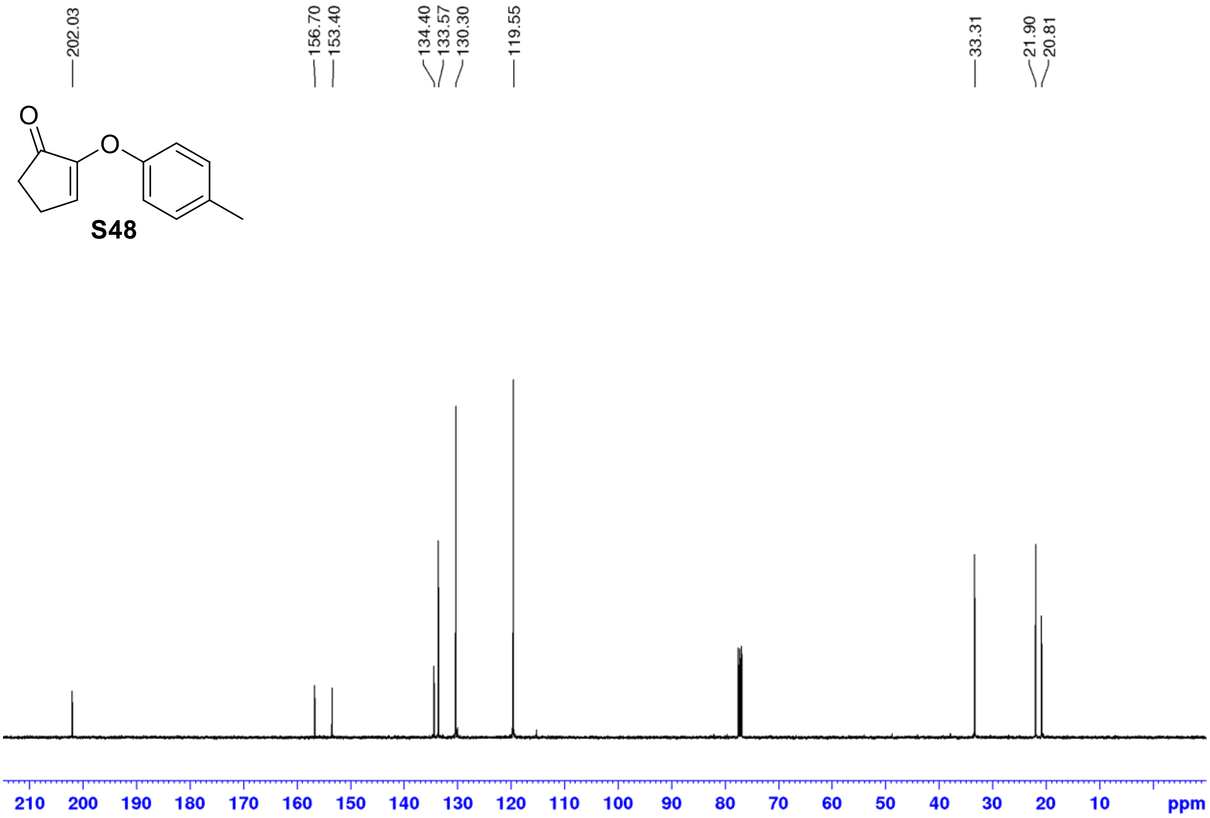
202.03
156.70
153.40
134.40
133.57
130.30
119.55
33.31
21.90
20.81
O
O
S48
210
200
190
180
170
160
150
140
130
120
110
100
90
80
70
60
50
40
30
20
10
ppm

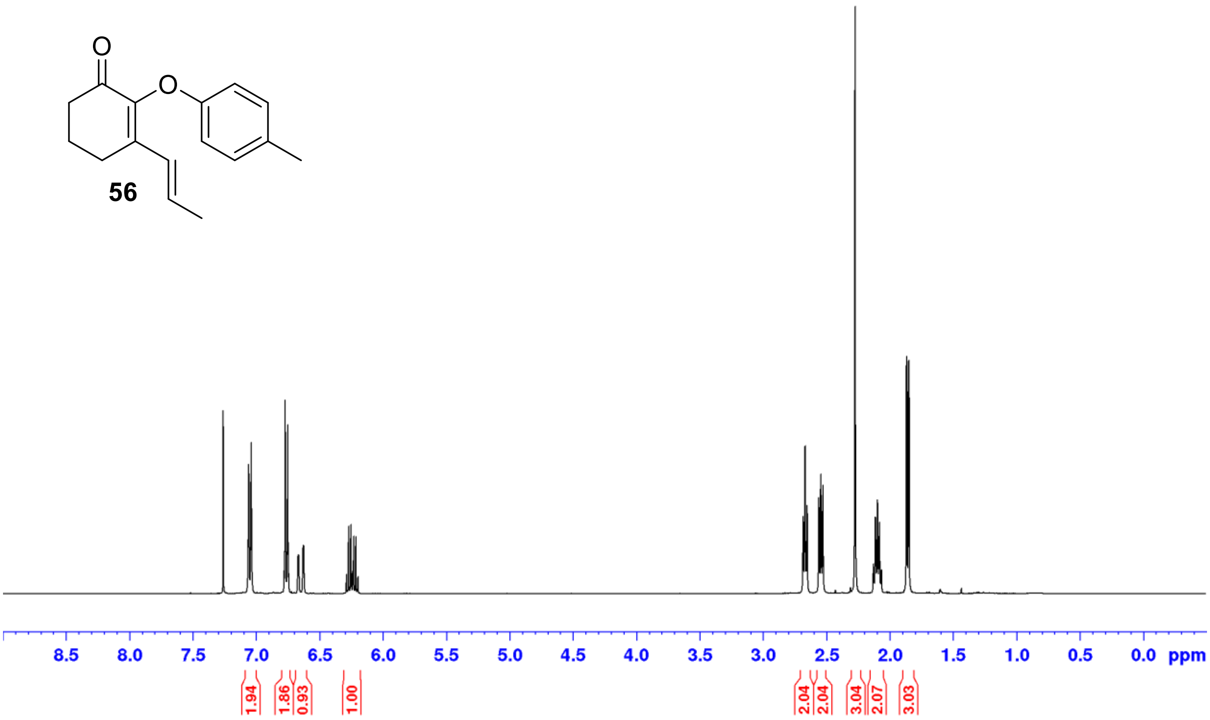
56
8.5
8.0
7.5
7.0
6.5
6.0
5.5
5.0
4.5
4.0
3.5
3.0
2.5
2.0
1.5
1.0
0.5
0.0 ppm
1.94
1.86
0.93
1.00
2.04
2.04
3.04
2.07
3.03

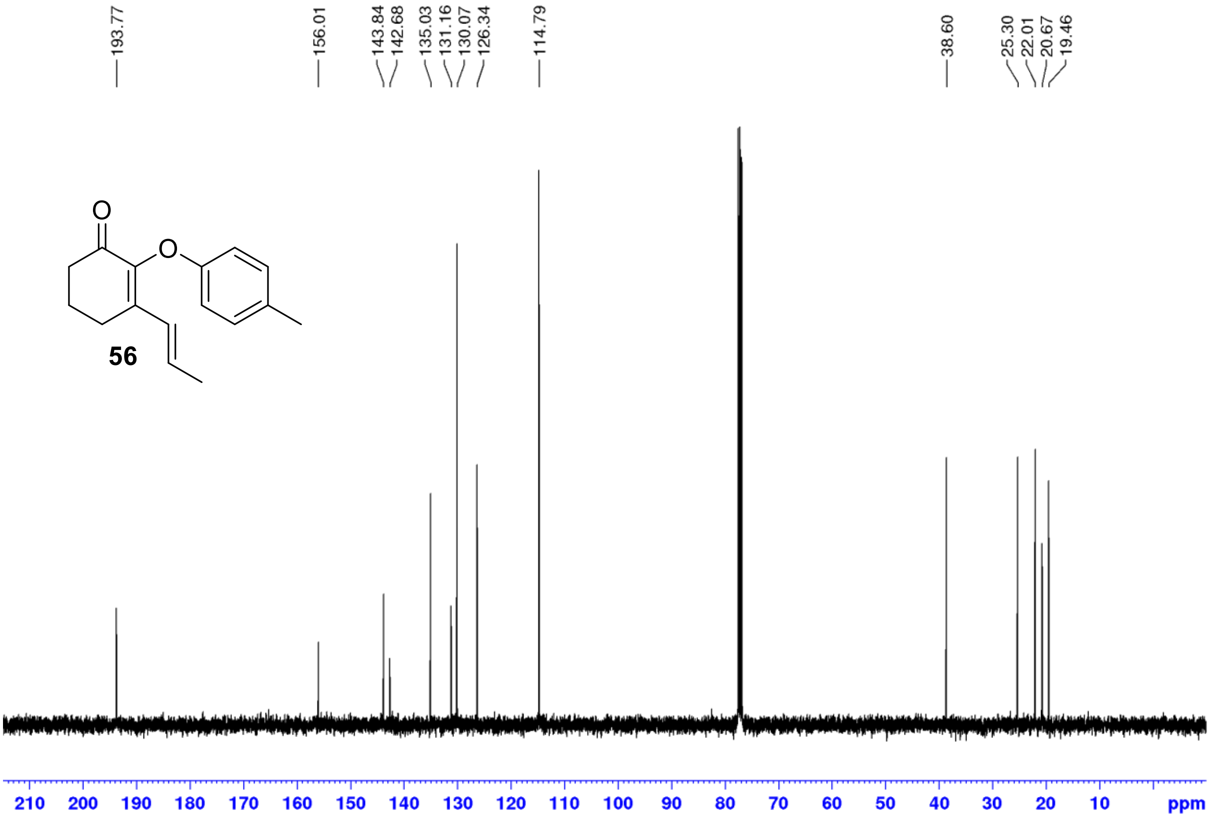
193.77
156.01
143.84
142.68
135.03
131.16
130.07
126.34
114.79
38.60
25.30
22.01
20.67
19.46
56
210
200
190
180
170
160
150
140
130
120
110
100
90
80
70
60
50
40
30
20
10
ppm

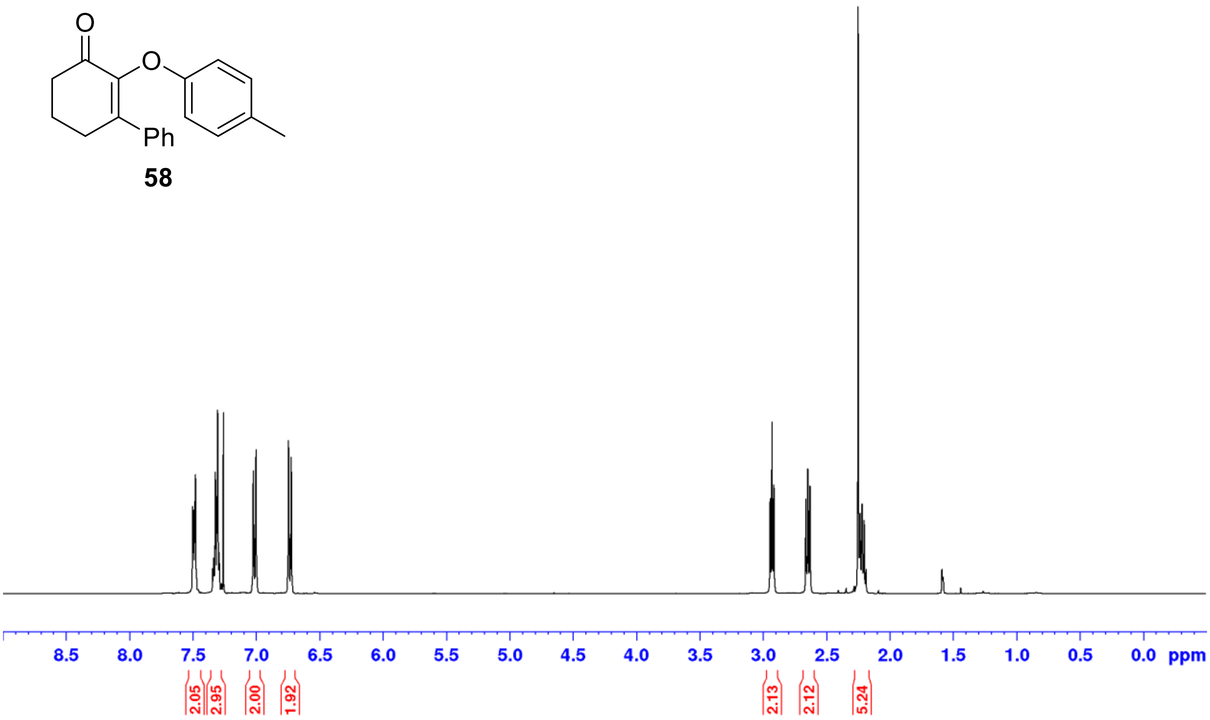

O
O
Ph
8.5
8.0
7.5
7.0
6.5
6.0
5.5
5.0
4.5
4.0
3.5
3.0
2.5
2.0
1.5
1.0
0.5
0.0
ppm
2.05
2.95
2.00
1.92
2.13
2.12
5.24

58

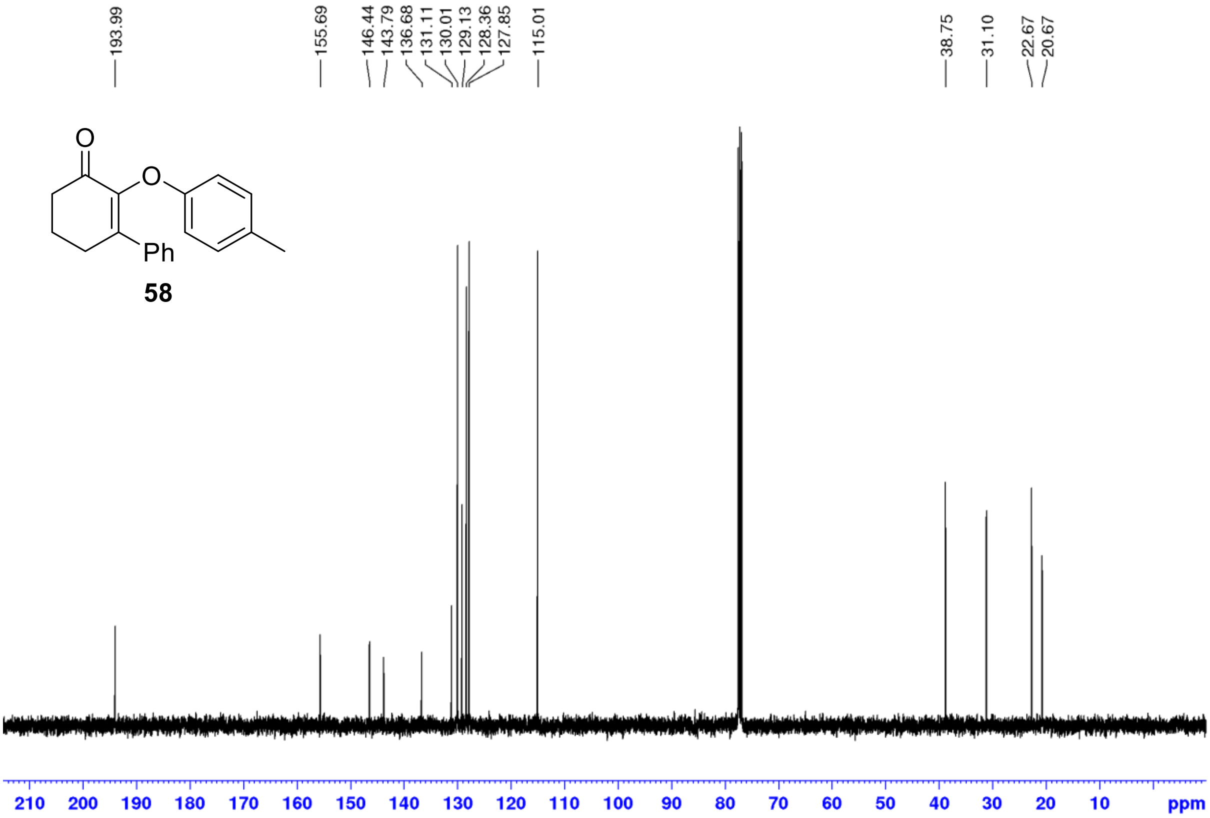

193.99
155.69
146.44
143.79
136.68
131.11
130.01
129.13
128.36
127.85
115.01
38.75
31.10
22.67
20.67
O
O
Ph
210
200
190
180
170
160
150
140
130
120
110
100
90
80
70
60
50
40
30
20
10
ppm

58

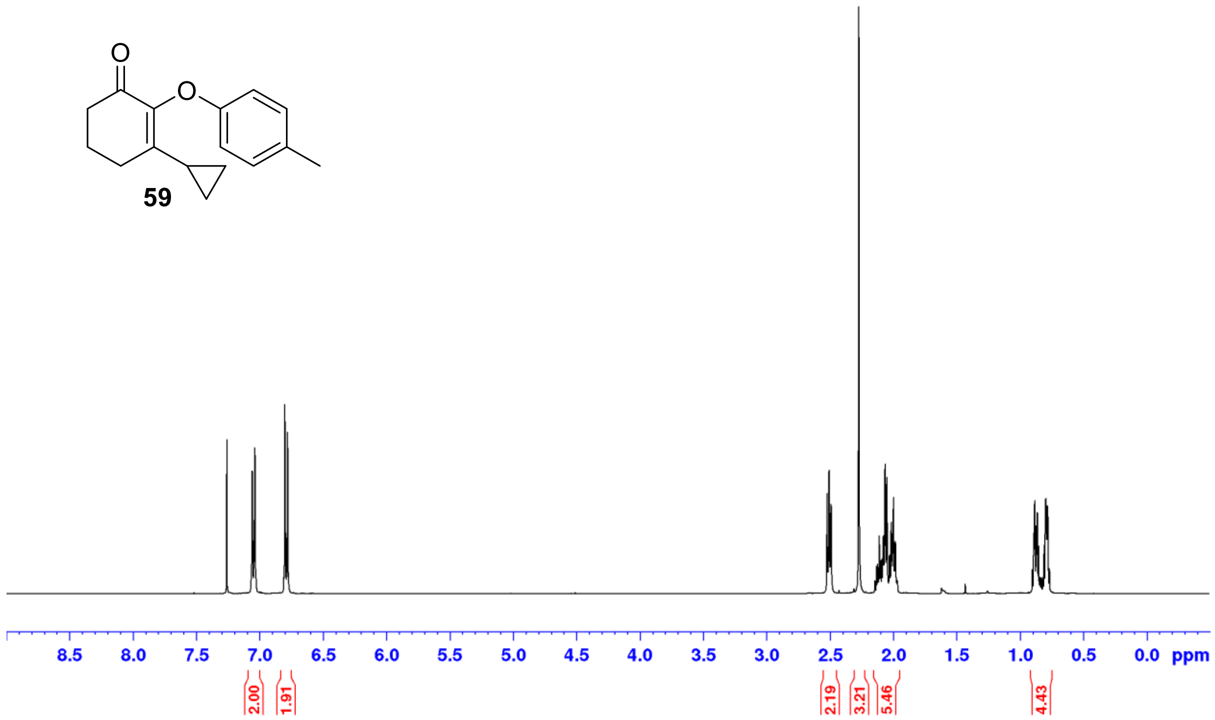
59
8.5
8.0
7.5
7.0
6.5
6.0
5.5
5.0
4.5
4.0
3.5
3.0
2.5
2.0
1.5
1.0
0.5
0.0 ppm
2.00
1.91
2.19
3.21
5.46
4.43

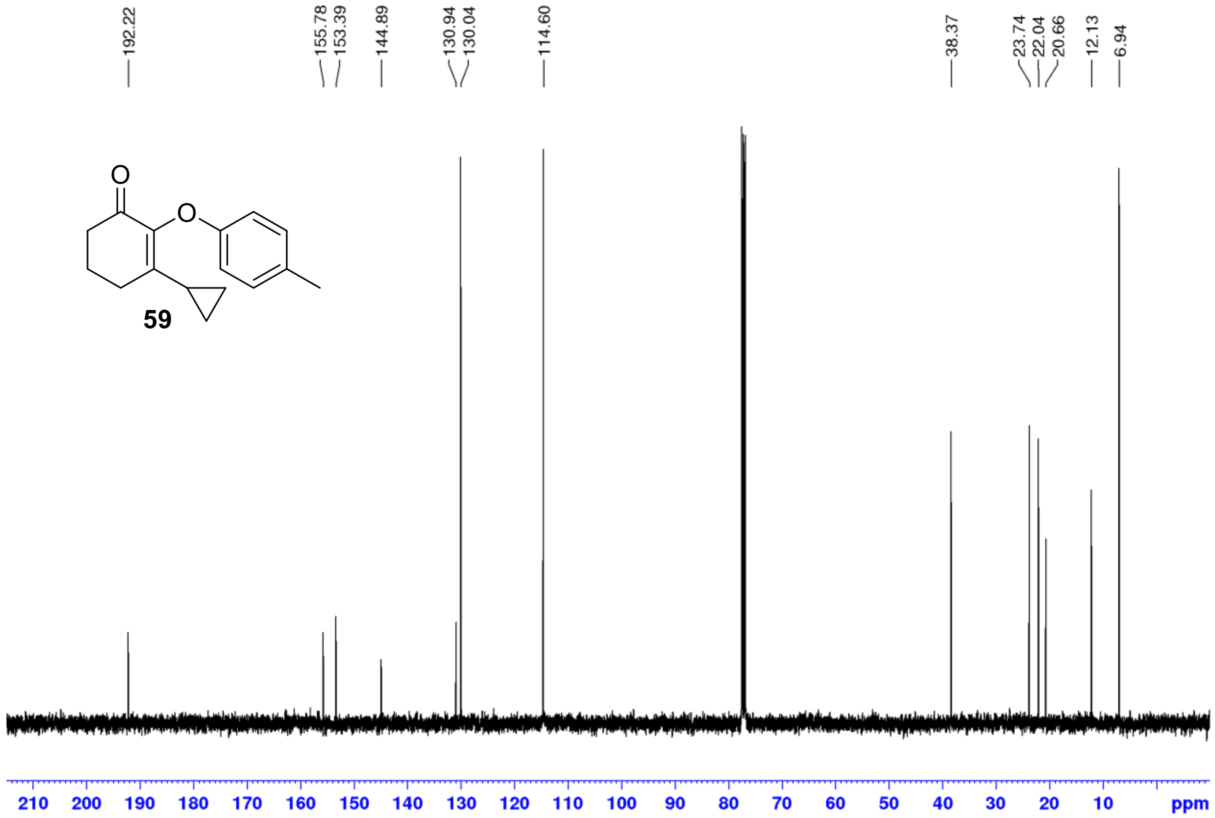
192.22
155.78
153.39
144.89
130.94
130.04
114.60
38.37
23.74
22.04
20.66
12.13
6.94
59
210
200
190
180
170
160
150
140
130
120
110
100
90
80
70
60
50
40
30
20
10
ppm

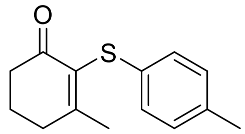

S49

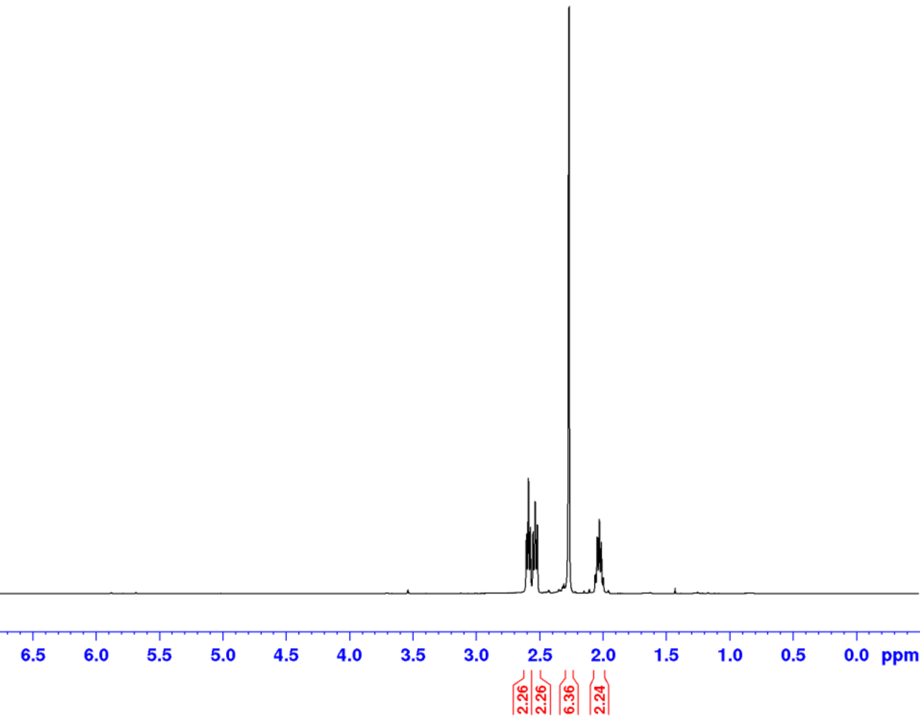
8.5 8.0 7.5 7.0 6.5 6.0 5.5 5.0 4.5 4.0 3.5 3.0 2.5 2.0 1.5 1.0 0.5 0.0 ppm
1.94
2.00
2.26
2.26
6.36
2.24

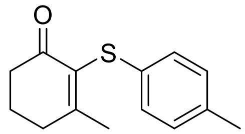

S49

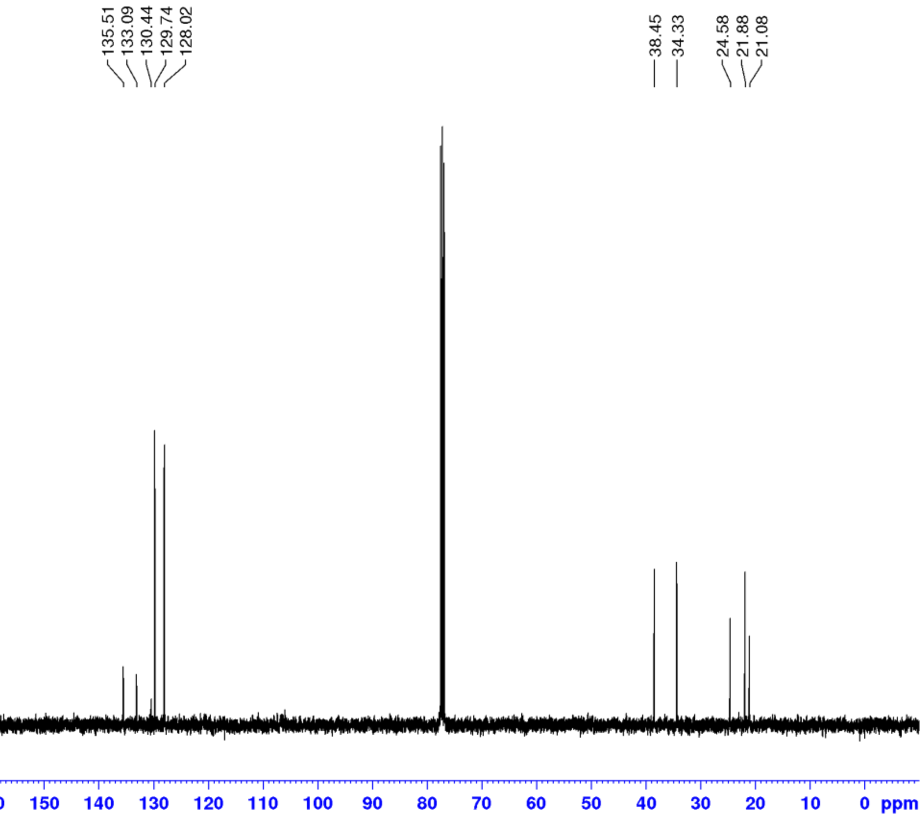
194.75
168.82
135.51
133.09
130.44
129.74
128.02
38.45
34.33
24.58
21.88
21.08
200 190 180 170 160 150 140 130 120 110 100 90 80 70 60 50 40 30 20 10 0 ppm

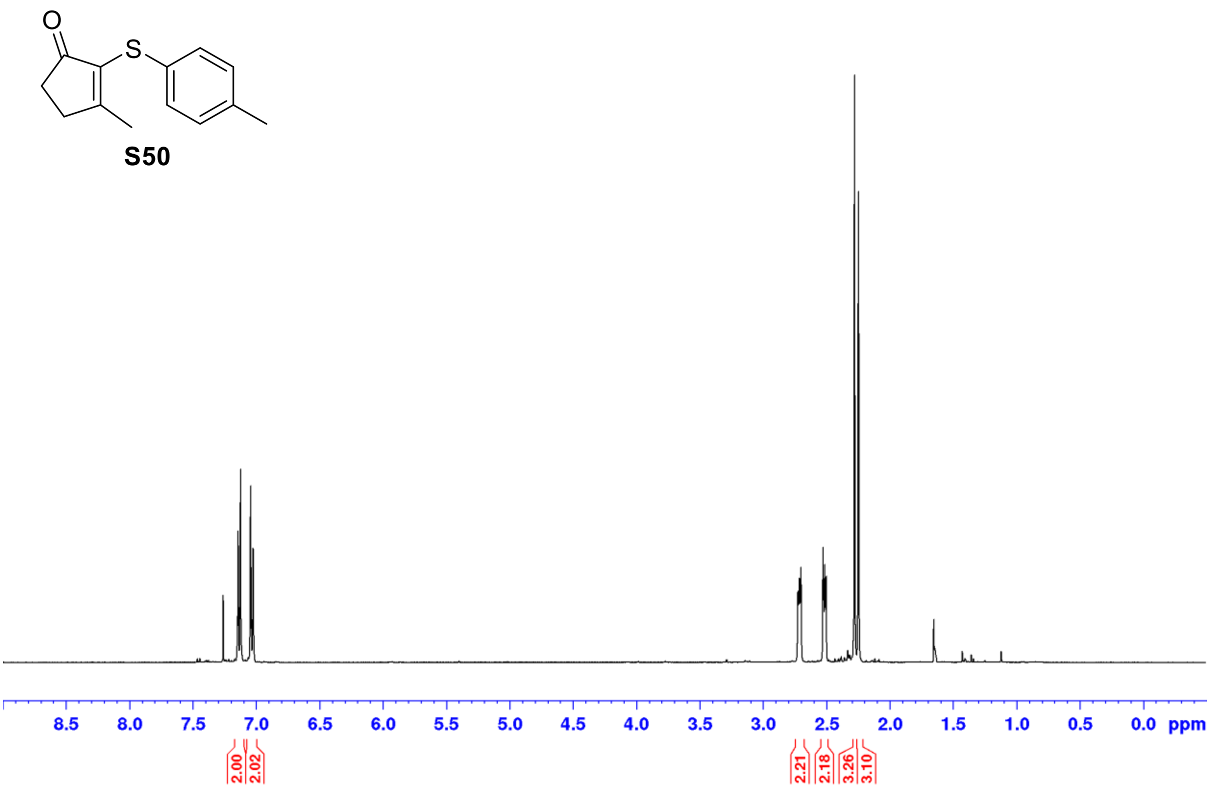

O
S
S50
8.5
8.0
7.5
7.0
6.5
6.0
5.5
5.0
4.5
4.0
3.5
3.0
2.5
2.0
1.5
1.0
0.5
0.0 ppm
2.00
2.02
2.21
2.18
3.26
3.10

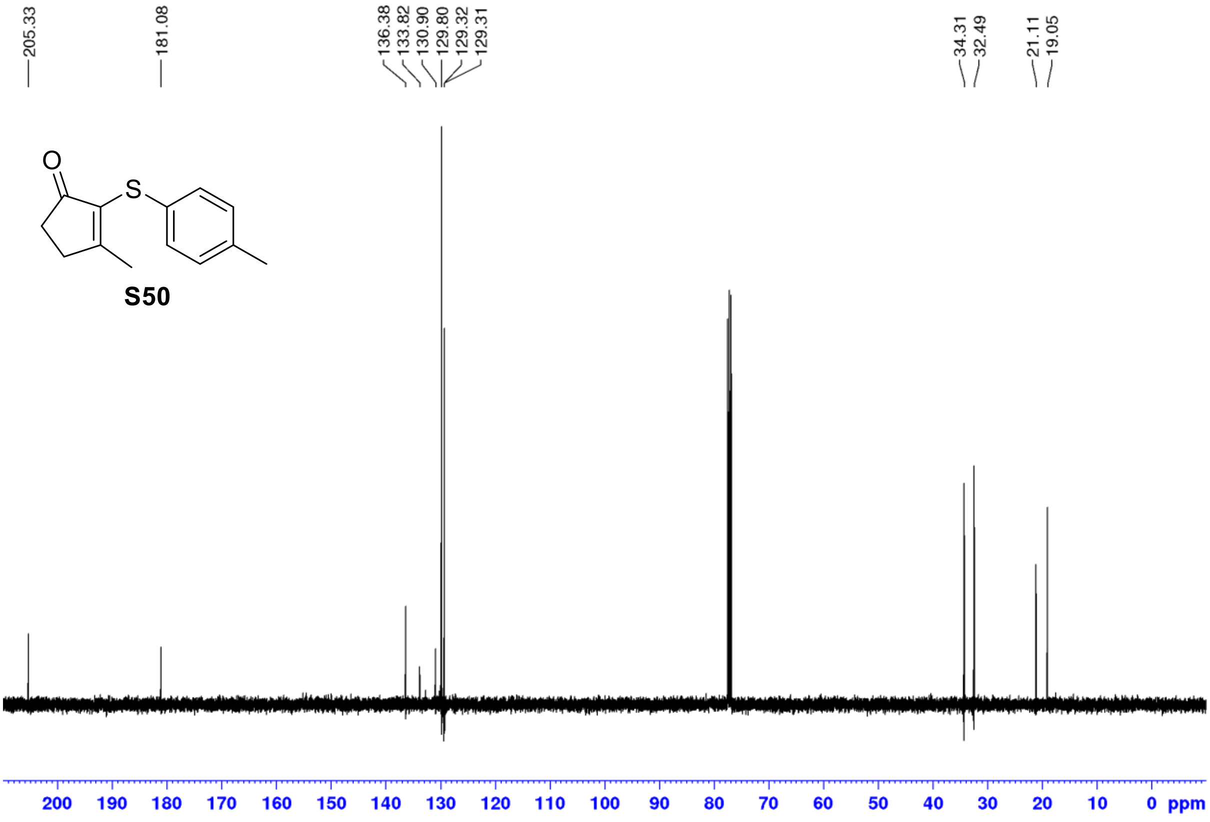

205.33
181.08
136.38
133.82
130.90
129.80
129.32
129.31
34.31
32.49
21.11
19.05
O
S
S50
200
190
180
170
160
150
140
130
120
110
100
90
80
70
60
50
40
30
20
10
0 ppm

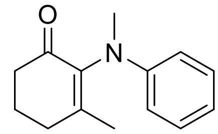

S51

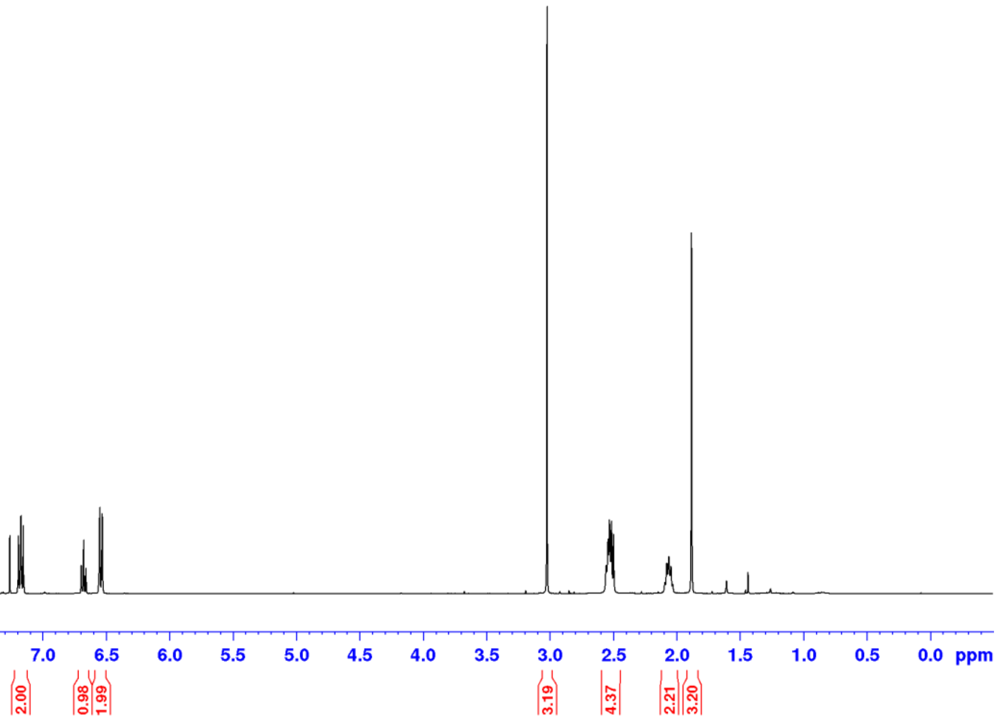
8.5 8.0 7.5 7.0 6.5 6.0 5.5 5.0 4.5 4.0 3.5 3.0 2.5 2.0 1.5 1.0 0.5 0.0 ppm
2.00
0.98
1.99
3.19
4.37
2.21
3.20

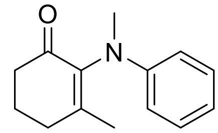

S51

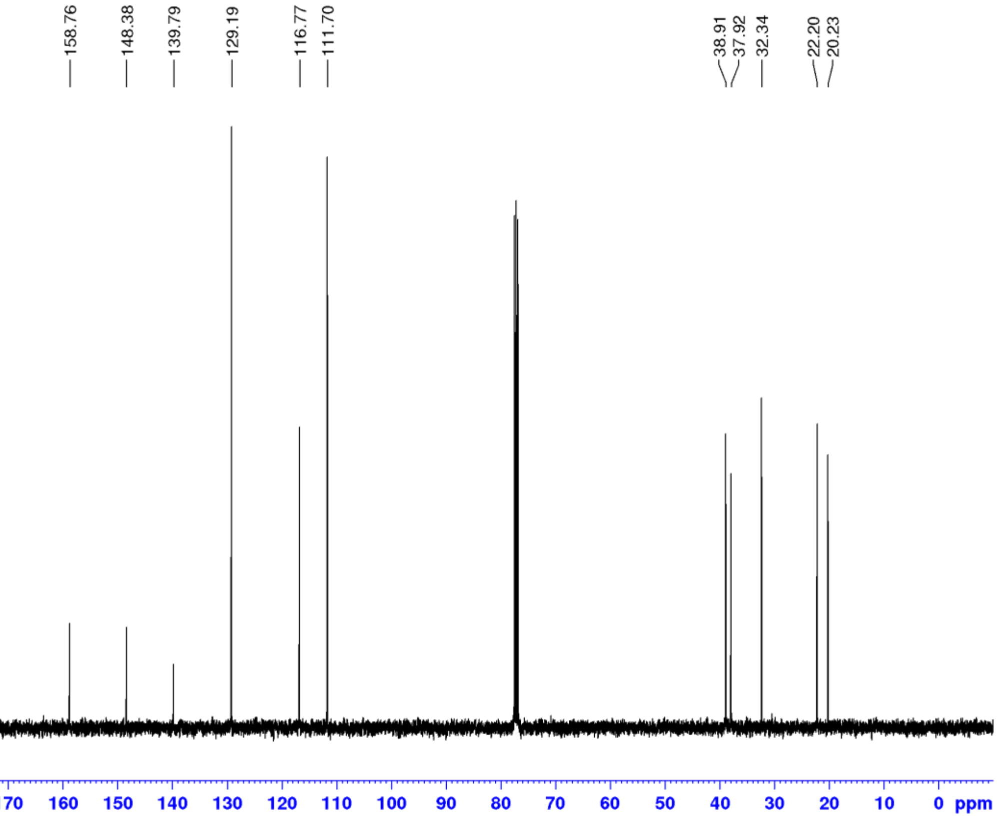
196.18
158.76
148.38
139.79
129.19
116.77
111.70
38.91
37.92
32.34
22.20
20.23
200 190 180 170 160 150 140 130 120 110 100 90 80 70 60 50 40 30 20 10 0 ppm

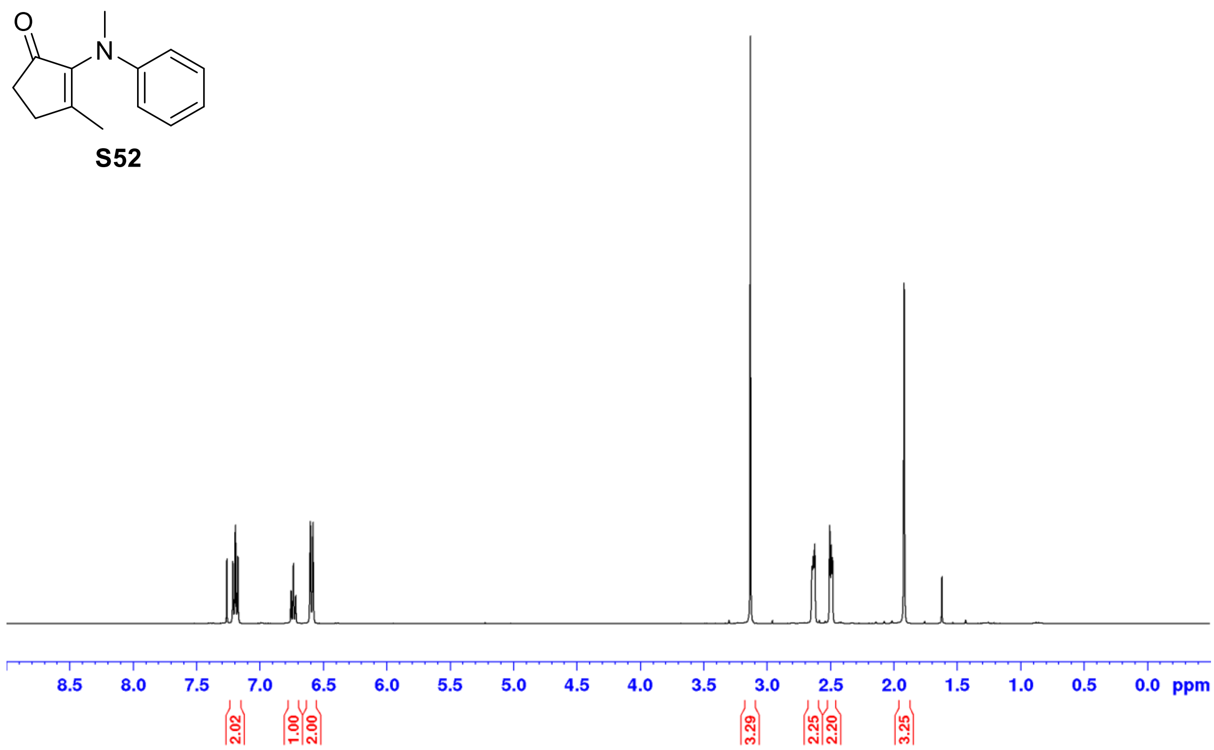
O
N
S52
8.5 8.0 7.5 7.0 6.5 6.0 5.5 5.0 4.5 4.0 3.5 3.0 2.5 2.0 1.5 1.0 0.5 0.0 ppm
2.02
1.00
2.00
3.29
2.25
2.20
3.25

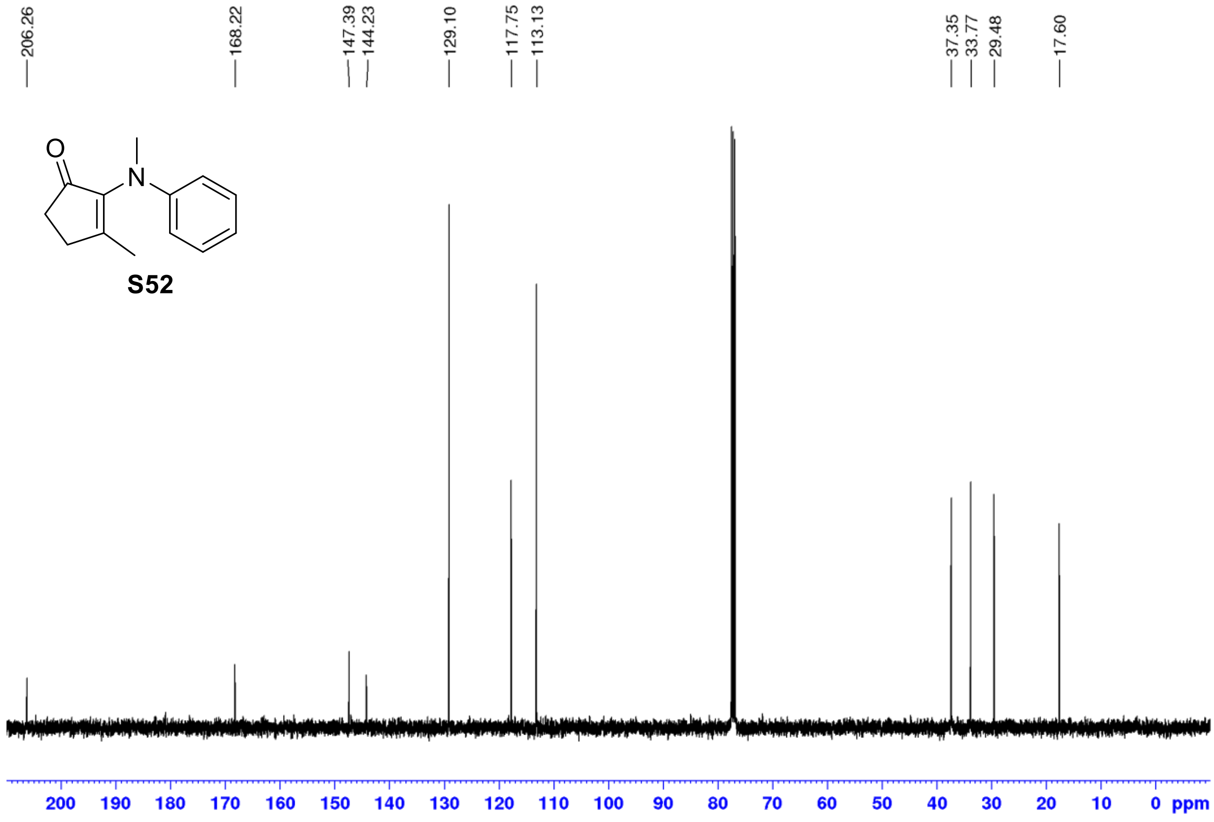
206.26
168.22
147.39
144.23
129.10
117.75
113.13
37.35
33.77
29.48
17.60
O
N
S52
200 190 180 170 160 150 140 130 120 110 100 90 80 70 60 50 40 30 20 10 0 ppm

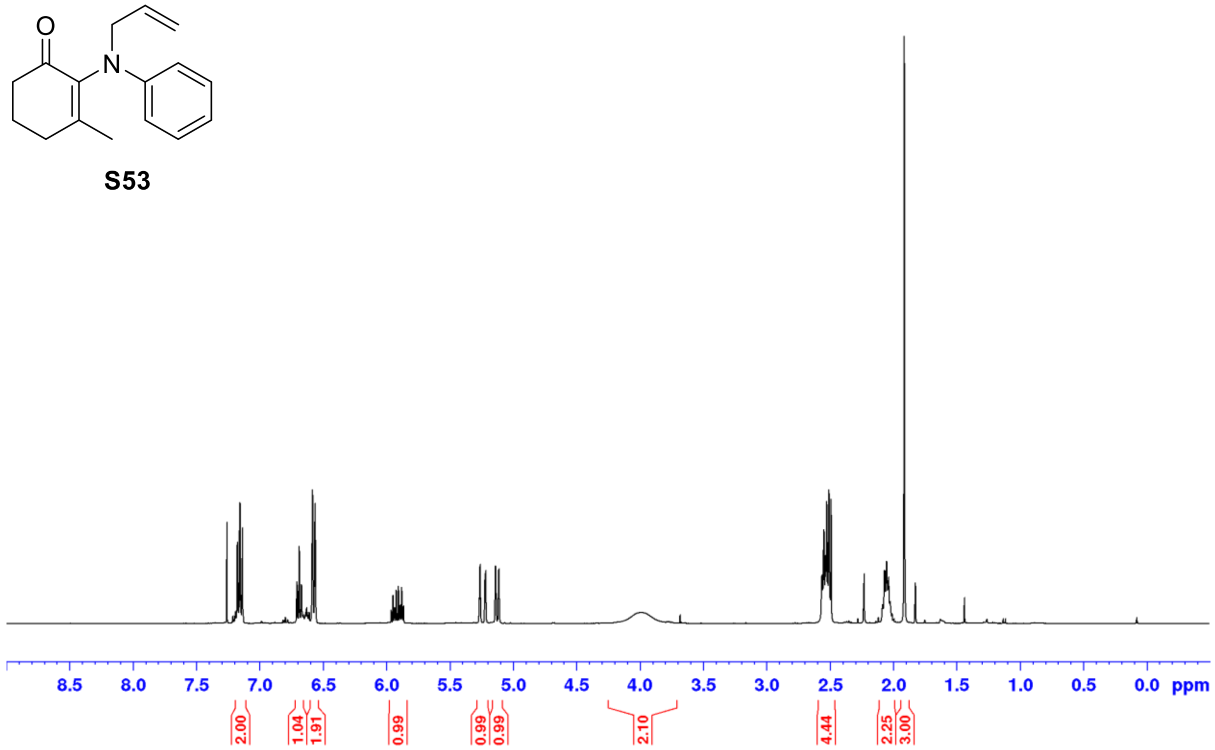

O
N
S53
8.5 8.0 7.5 7.0 6.5 6.0 5.5 5.0 4.5 4.0 3.5 3.0 2.5 2.0 1.5 1.0 0.5 0.0 ppm
2.00 1.04 1.91 0.99 0.99 0.99 2.10 4.44 2.25 3.00

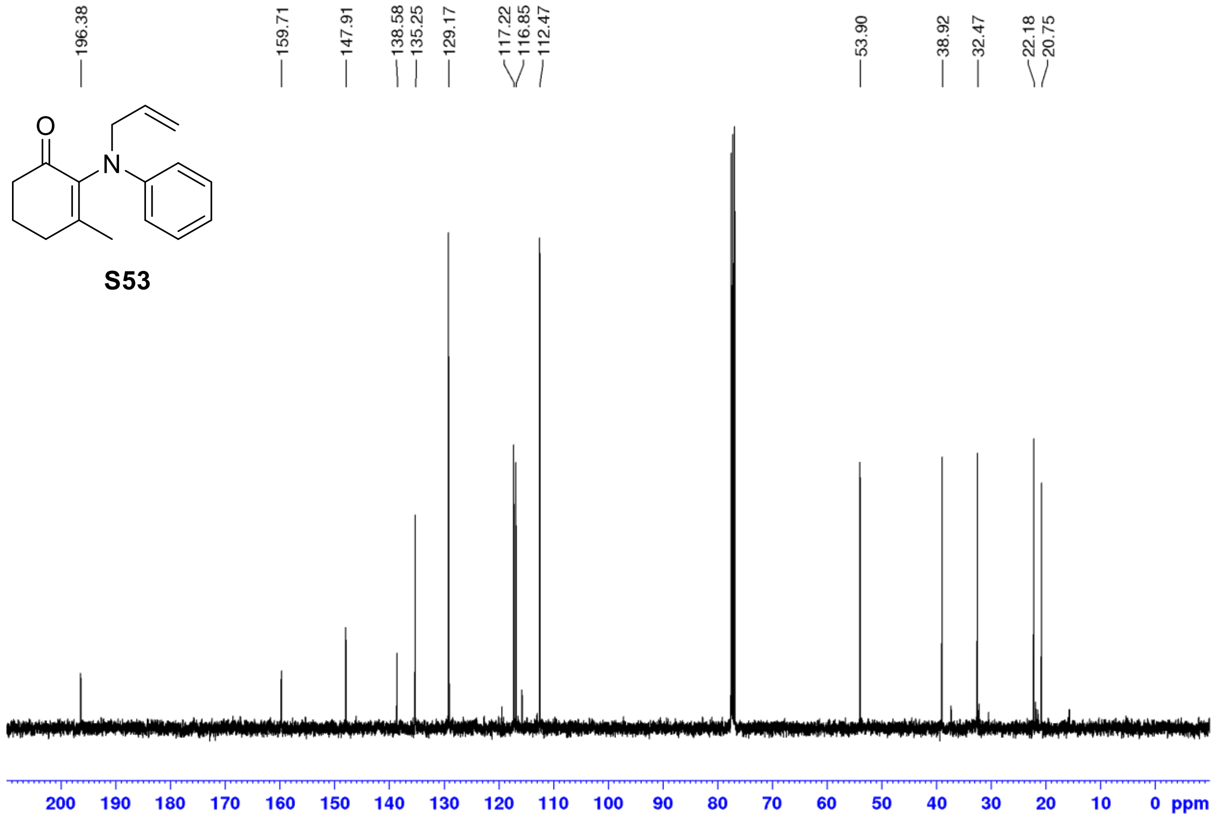

196.38
159.71
147.91
138.58
135.25
129.17
117.22
116.85
112.47
53.90
38.92
32.47
22.18
20.75
O
N
S53
200 190 180 170 160 150 140 130 120 110 100 90 80 70 60 50 40 30 20 10 0 ppm

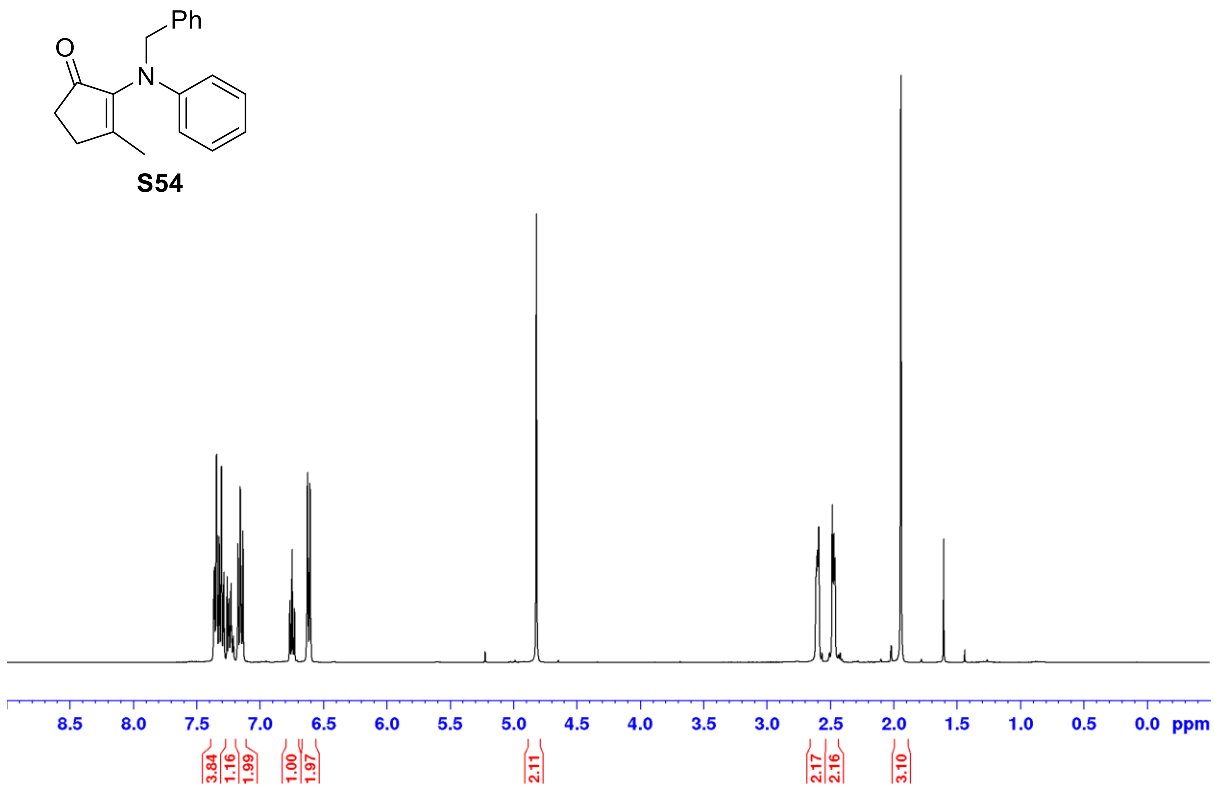
Ph
O
N
S54
8.5
8.0
7.5
7.0
6.5
6.0
5.5
5.0
4.5
4.0
3.5
3.0
2.5
2.0
1.5
1.0
0.5
0.0 ppm
3.84
1.16
1.99
1.00
1.97
2.11
2.17
2.16
3.10

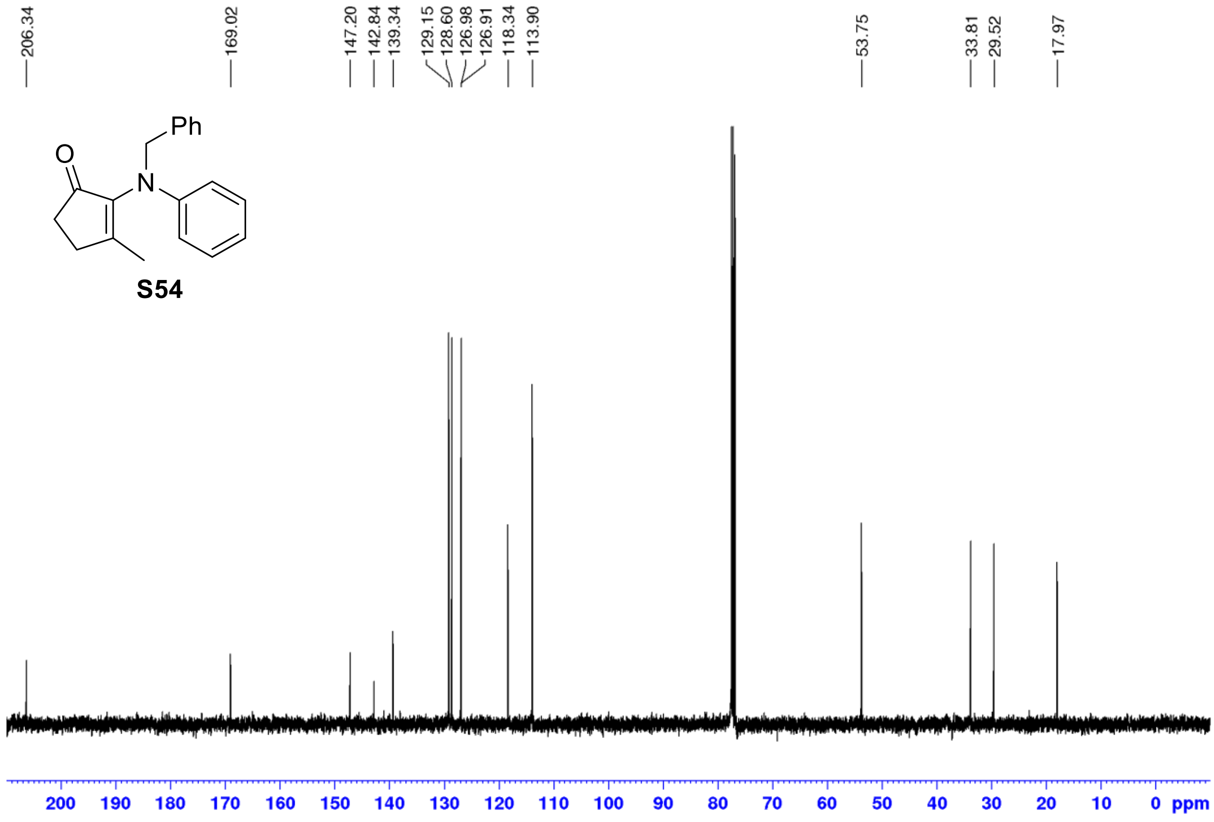
206.34
169.02
147.20
142.84
139.34
129.15
128.60
126.98
126.91
118.34
113.90
53.75
33.81
29.52
17.97
Ph
O
N
S54
200
190
180
170
160
150
140
130
120
110
100
90
80
70
60
50
40
30
20
10
0 ppm

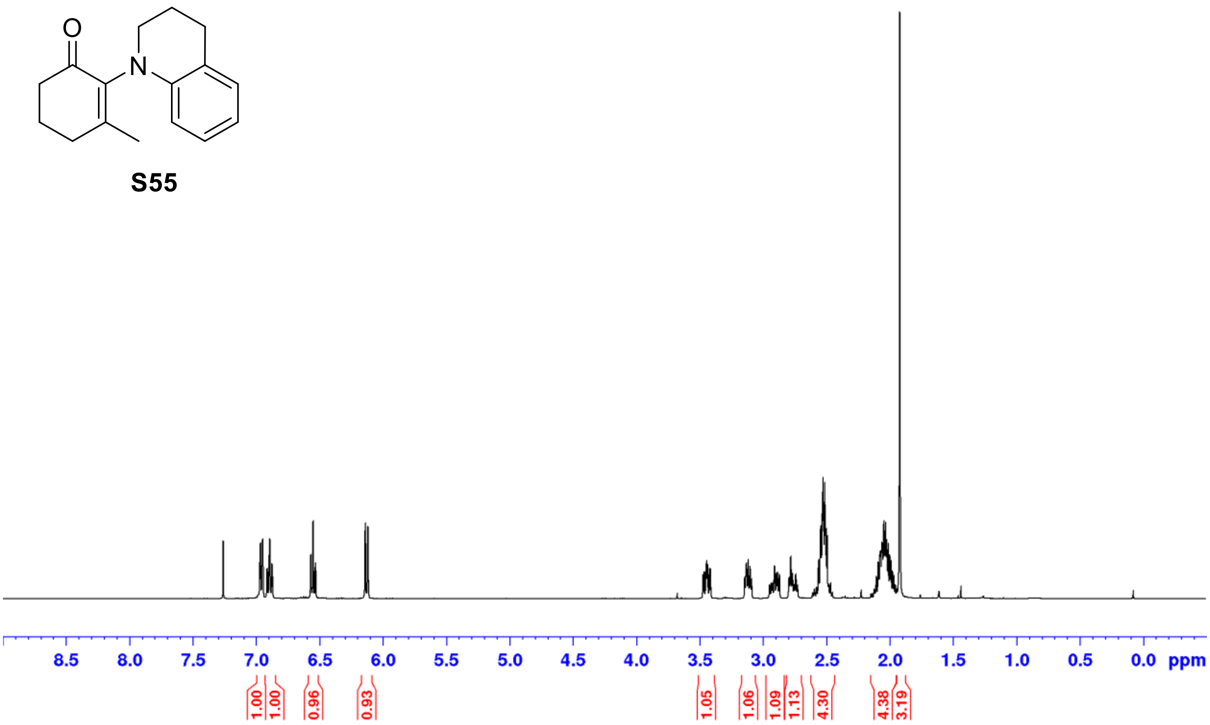
S55
8.5 8.0 7.5 7.0 6.5 6.0 5.5 5.0 4.5 4.0 3.5 3.0 2.5 2.0 1.5 1.0 0.5 0.0 ppm
1.00 1.00 0.96 0.93 1.05 1.06 1.09 1.13 4.30 4.38 3.19

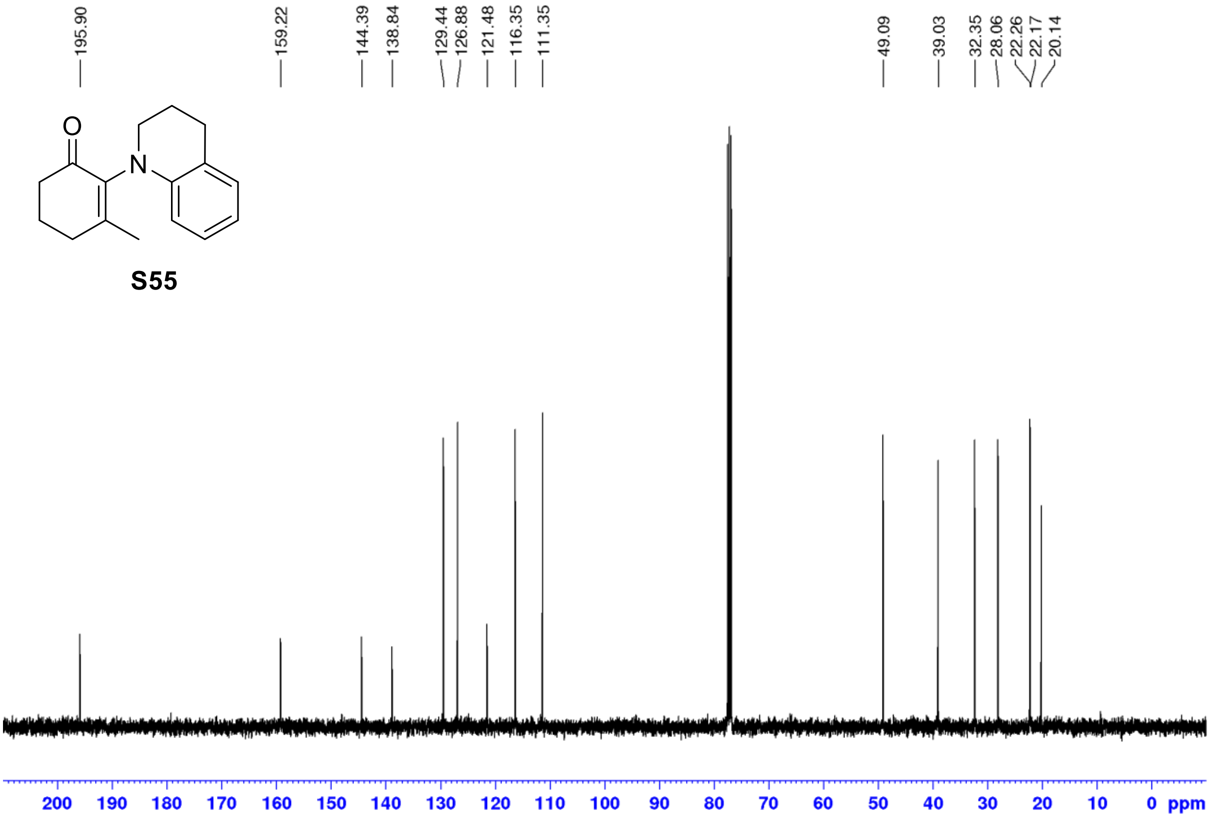
195.90
159.22
144.39
138.84
129.44
126.88
121.48
116.35
111.35
49.09
39.03
32.35
28.06
22.26
22.17
20.14
S55
200 190 180 170 160 150 140 130 120 110 100 90 80 70 60 50 40 30 20 10 0 ppm

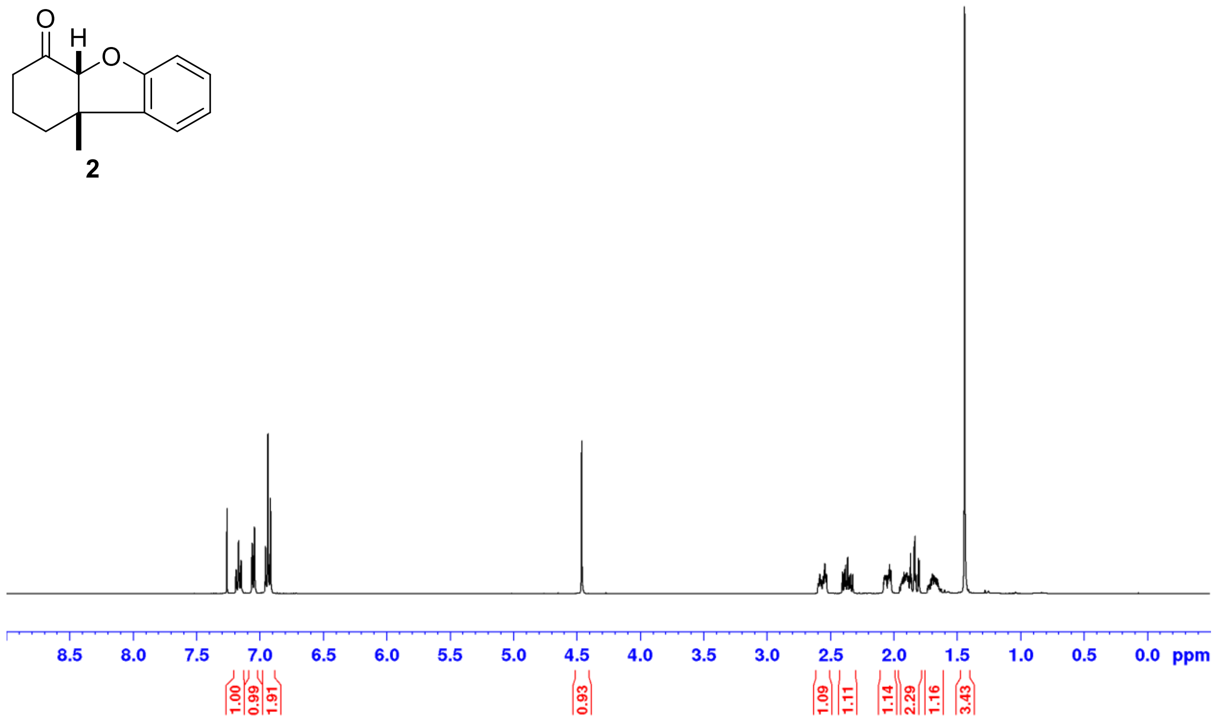
O
H
O
2
8.5
8.0
7.5
7.0
6.5
6.0
5.5
5.0
4.5
4.0
3.5
3.0
2.5
2.0
1.5
1.0
0.5
0.0 ppm
1.00
0.99
1.91
0.93
1.09
1.11
1.14
2.29
1.16
3.43

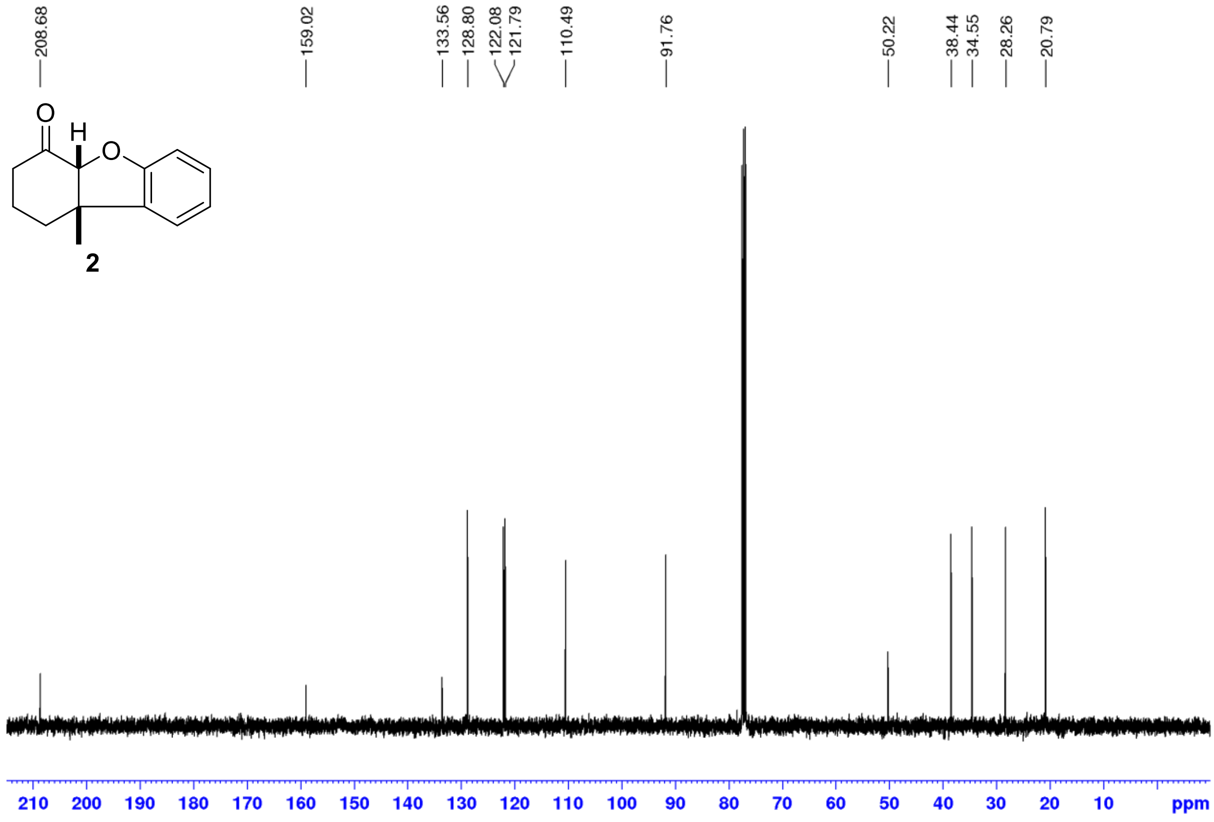
208.68
159.02
133.56
128.80
122.08
121.79
110.49
91.76
50.22
38.44
34.55
28.26
20.79
O
H
O
2
210
200
190
180
170
160
150
140
130
120
110
100
90
80
70
60
50
40
30
20
10
ppm

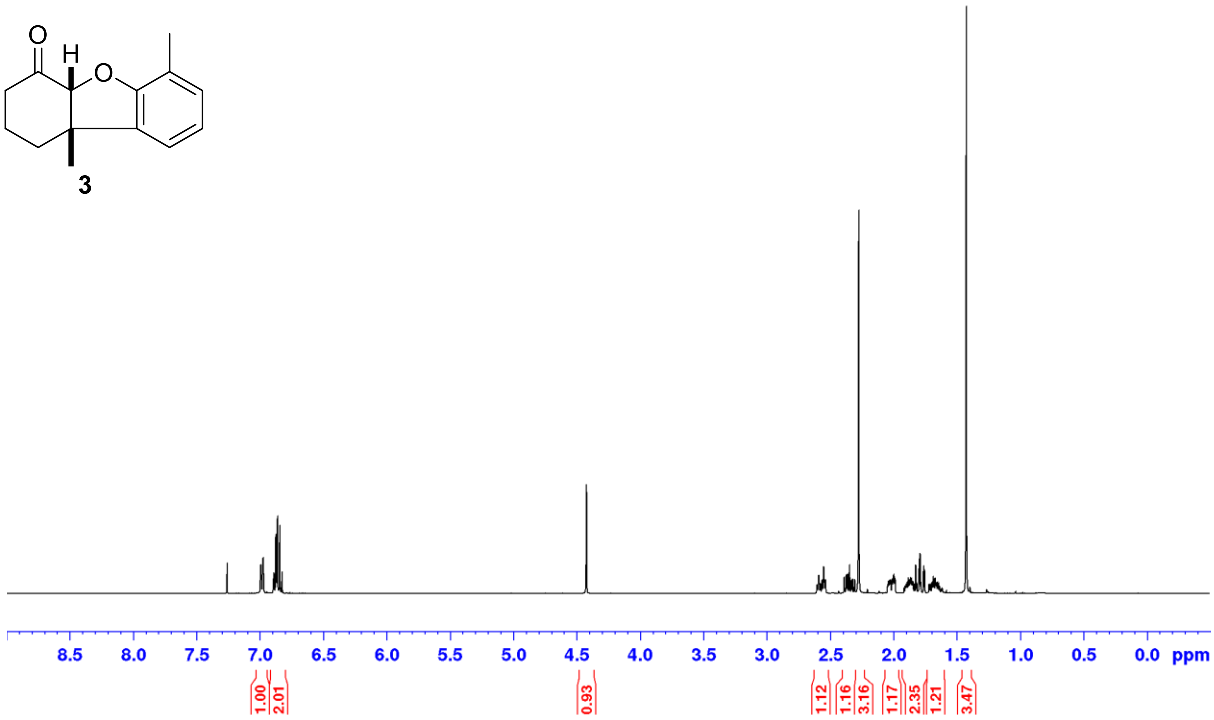
O
H
O
3
8.5
8.0
7.5
7.0
6.5
6.0
5.5
5.0
4.5
4.0
3.5
3.0
2.5
2.0
1.5
1.0
0.5
0.0
ppm
1.00
2.01
0.93
1.12
1.16
3.16
1.17
2.35
1.21
3.47

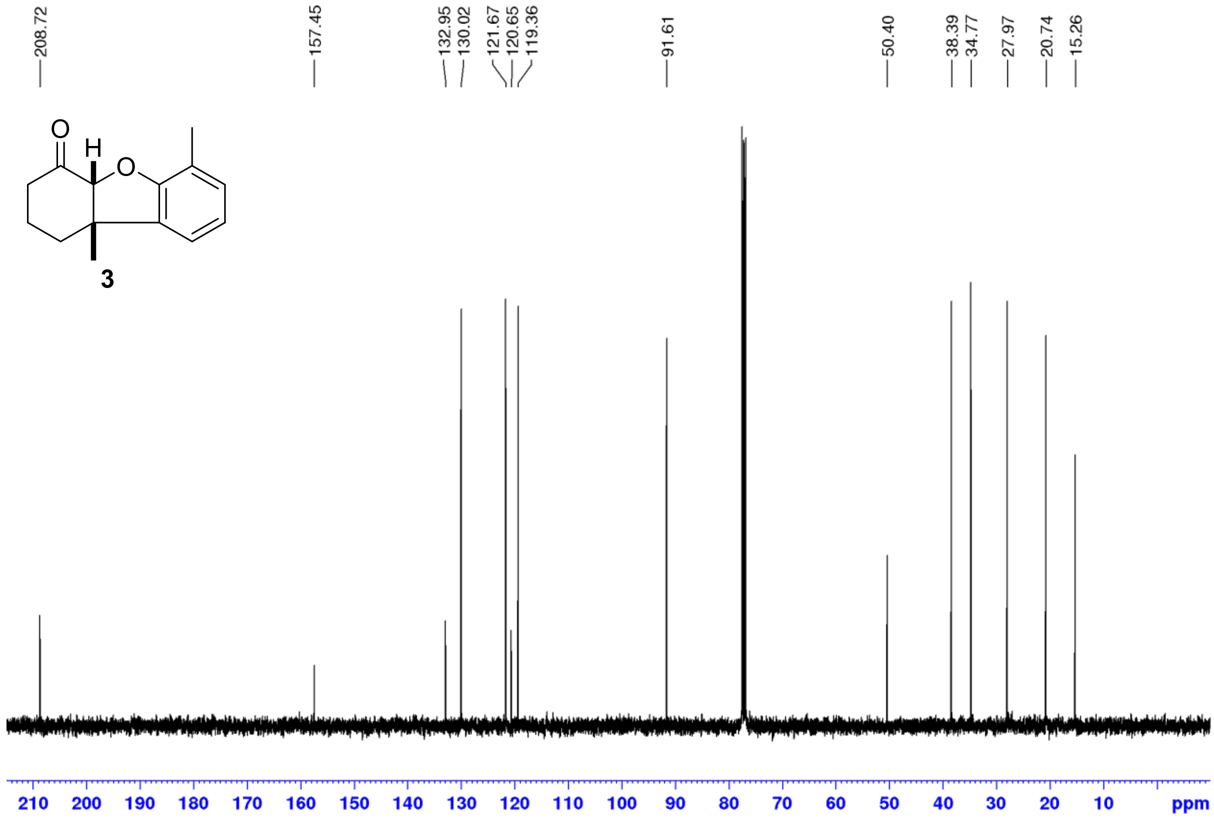
208.72
157.45
132.95
130.02
121.67
120.65
119.36
91.61
50.40
38.39
34.77
27.97
20.74
15.26
O
H
O
3
210
200
190
180
170
160
150
140
130
120
110
100
90
80
70
60
50
40
30
20
10
ppm

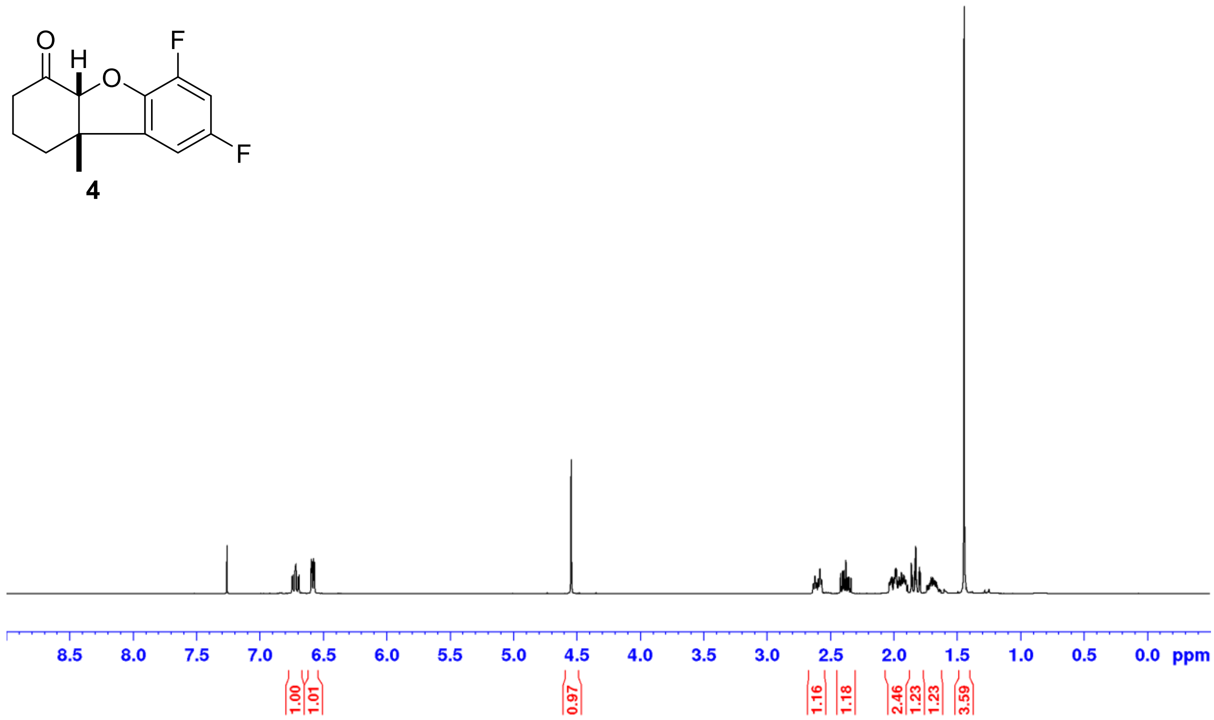
O
H
O
F
F
4
8.5
8.0
7.5
7.0
6.5
6.0
5.5
5.0
4.5
4.0
3.5
3.0
2.5
2.0
1.5
1.0
0.5
0.0
ppm
1.00
1.01
0.97
1.16
1.18
2.46
1.23
1.23
3.59

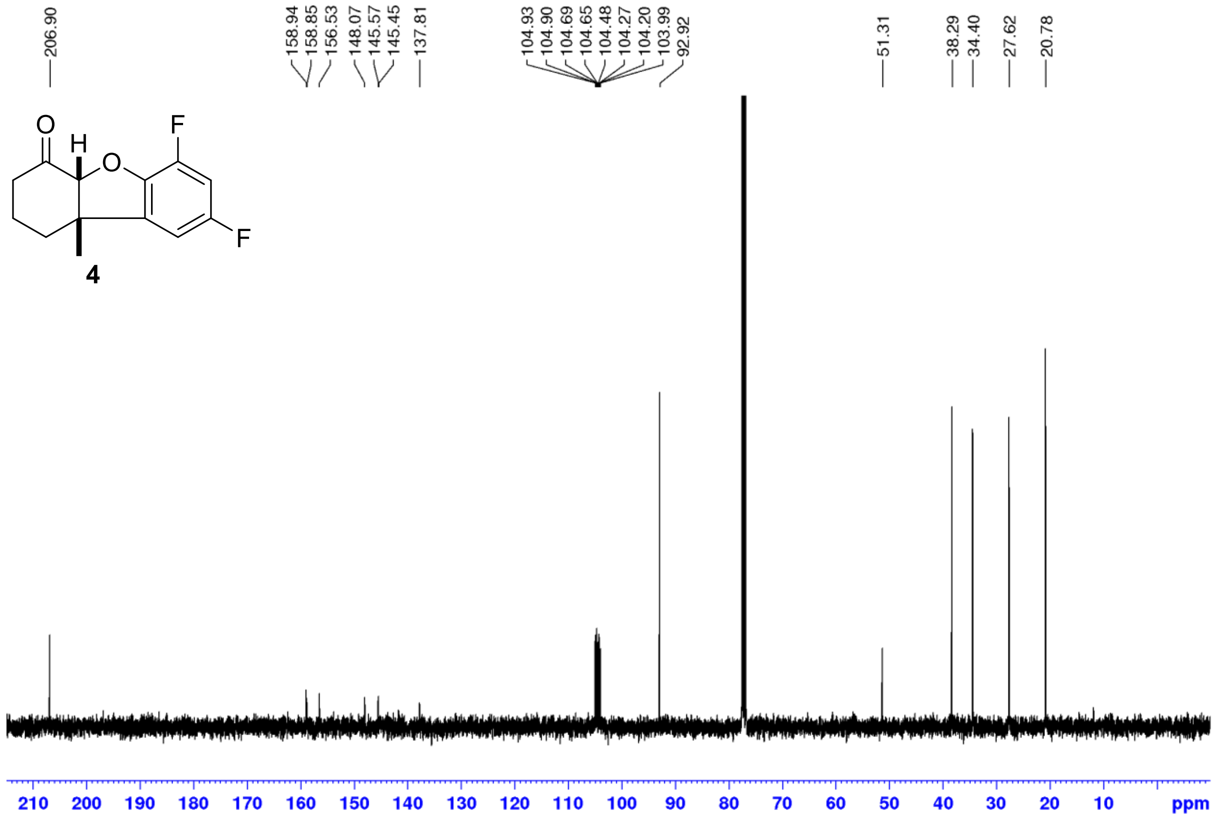
206.90
158.94
158.85
156.53
148.07
145.57
145.45
137.81
104.93
104.90
104.69
104.65
104.48
104.27
104.20
103.99
92.92
51.31
38.29
34.40
27.62
20.78
O
H
O
F
F
4
210
200
190
180
170
160
150
140
130
120
110
100
90
80
70
60
50
40
30
20
10
ppm

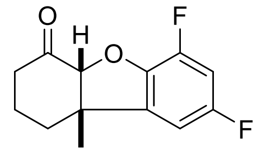

4

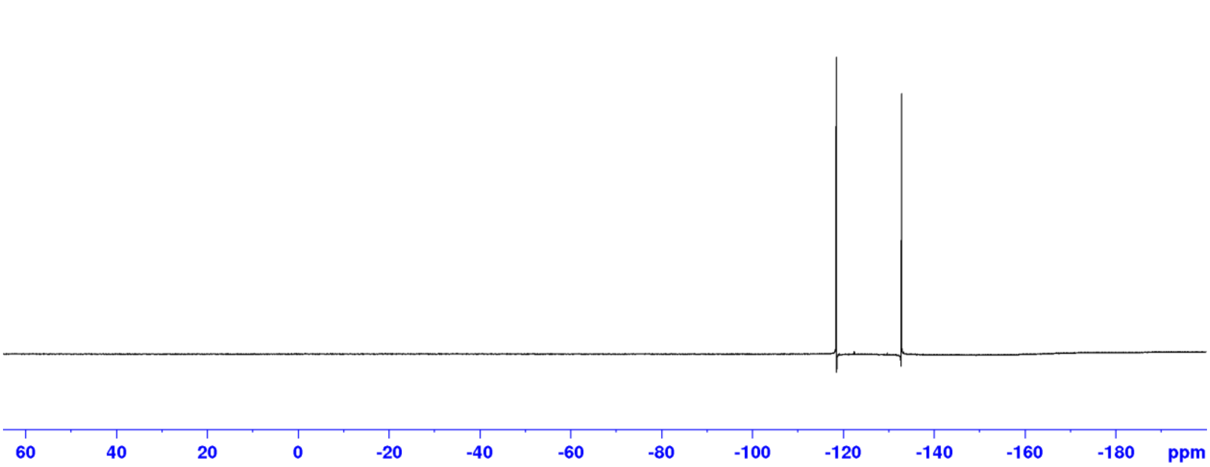

-118.50
-118.52
-118.54
-132.86
-132.89
60 40 20 0 -20 -40 -60 -80 -100 -120 -140 -160 -180 ppm

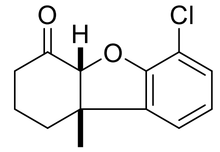

5

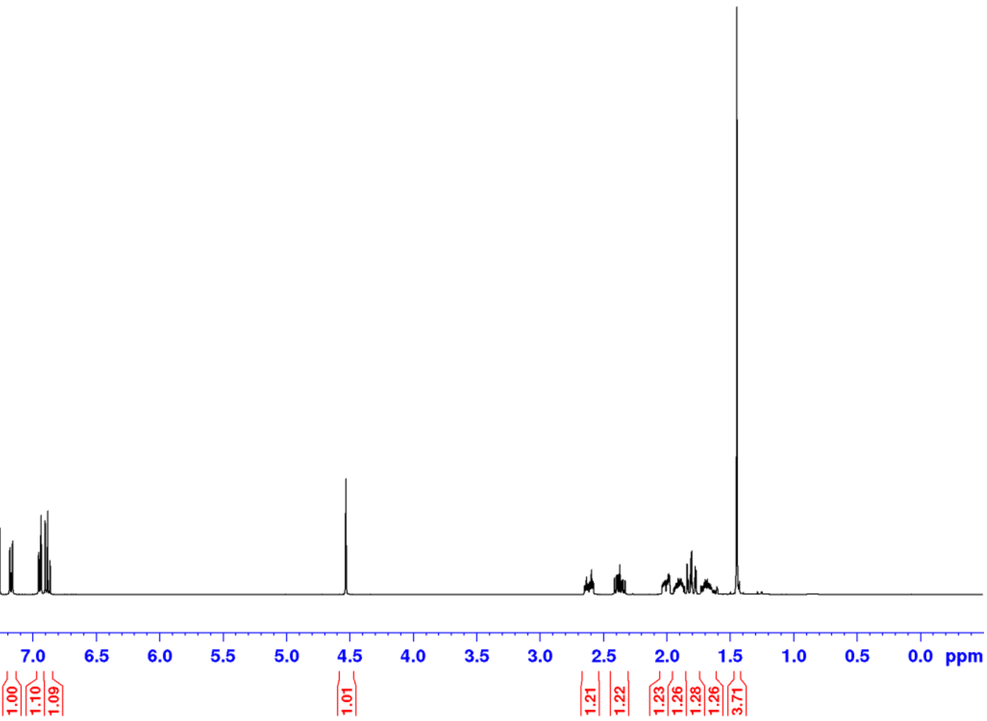

8.5 8.0 7.5 7.0 6.5 6.0 5.5 5.0 4.5 4.0 3.5 3.0 2.5 2.0 1.5 1.0 0.5 0.0 ppm
1.00 1.10 1.09 1.01 1.21 1.22 1.23 1.26 1.28 1.26 3.71

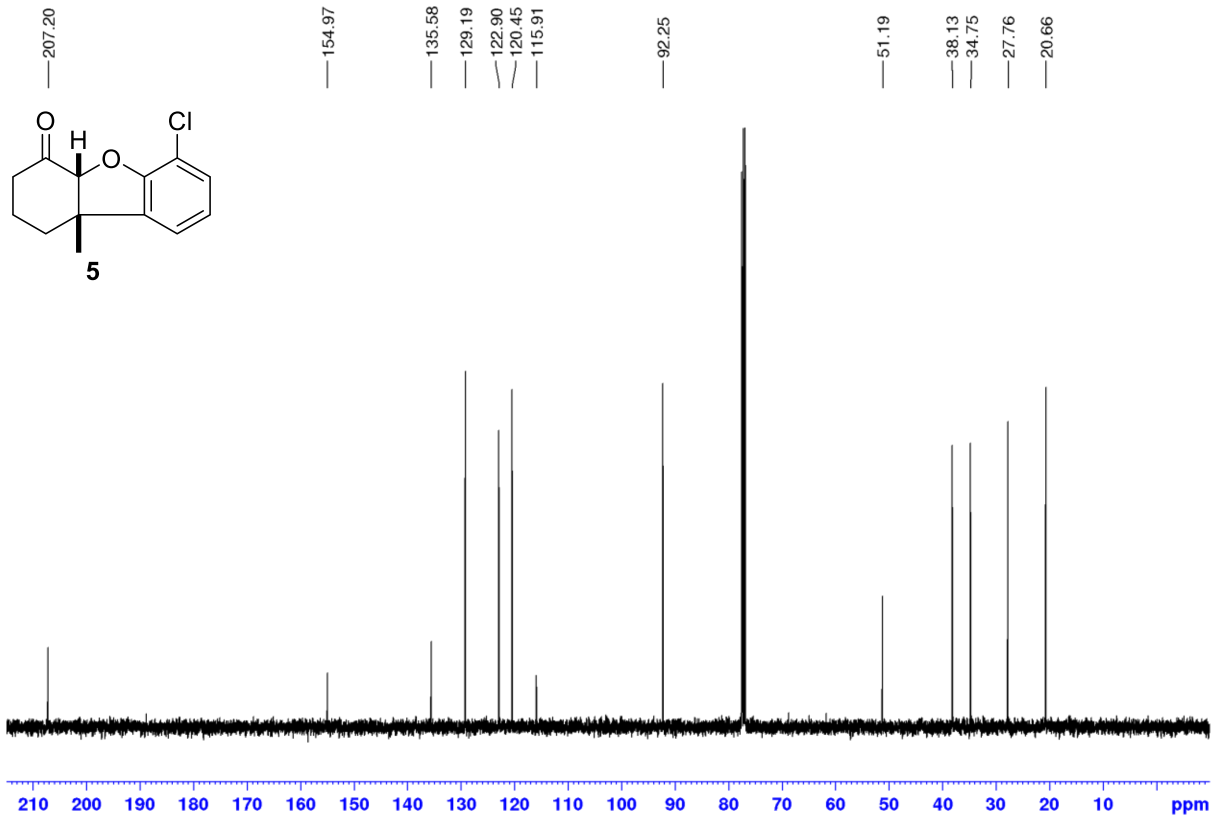
207.20
154.97
135.58
129.19
122.90
120.45
115.91
92.25
51.19
38.13
34.75
27.76
20.66
O
H
O
Cl
5
210 200 190 180 170 160 150 140 130 120 110 100 90 80 70 60 50 40 30 20 10 ppm

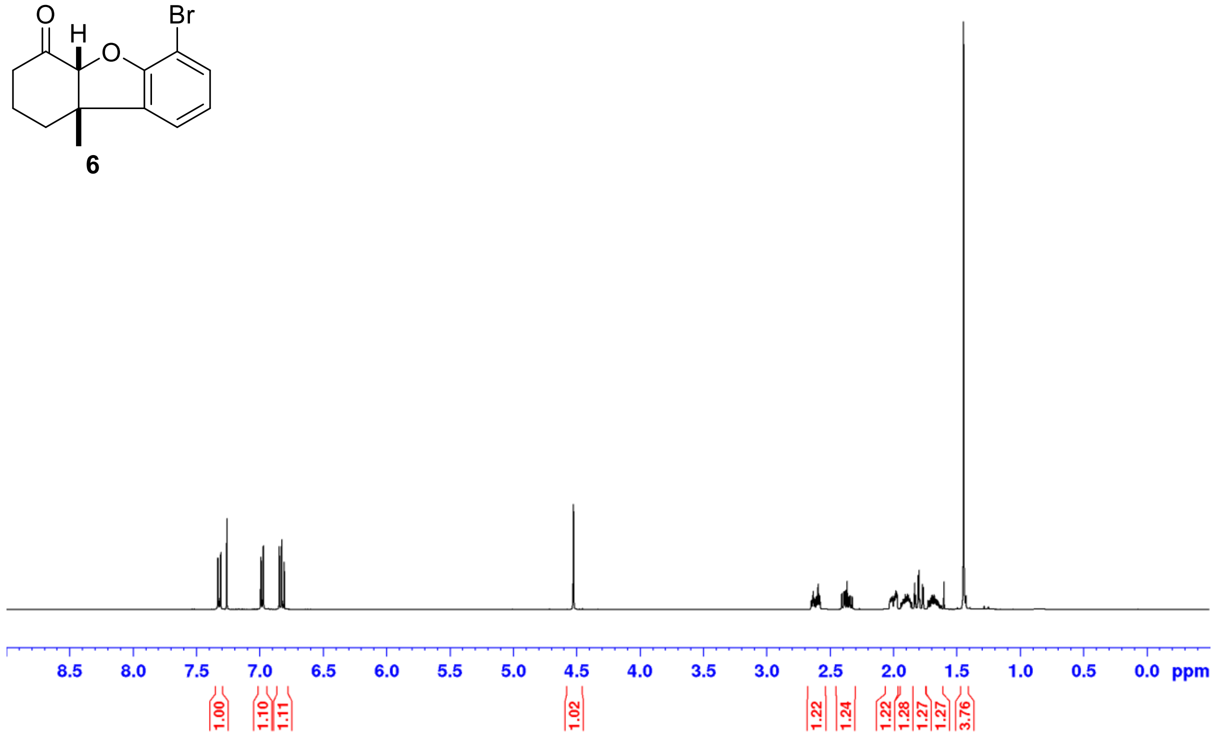
O
H
O
Br
6
8.5 8.0 7.5 7.0 6.5 6.0 5.5 5.0 4.5 4.0 3.5 3.0 2.5 2.0 1.5 1.0 0.5 0.0 ppm
1.00
1.10
1.11
1.02
1.22
1.24
1.22
1.28
1.27
1.27
3.76

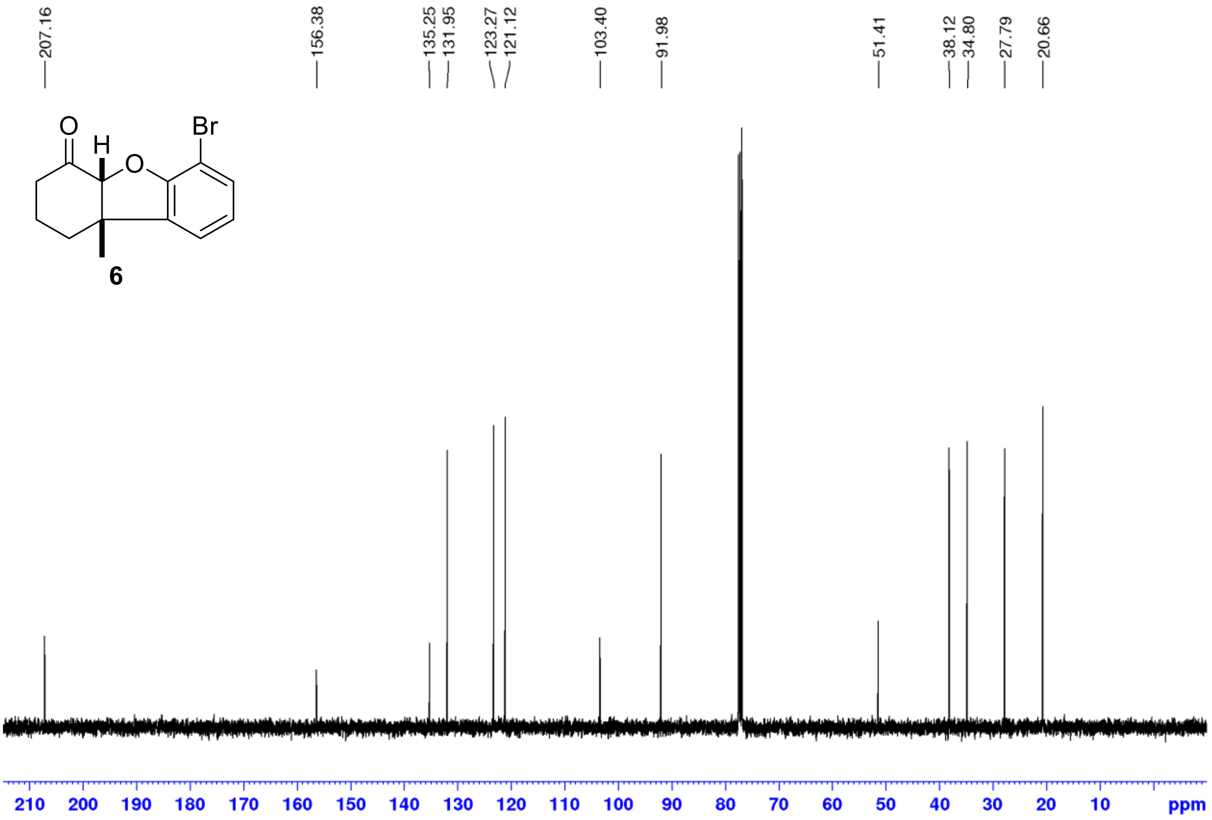
207.16
156.38
135.25
131.95
123.27
121.12
103.40
91.98
51.41
38.12
34.80
27.79
20.66
O
H
O
Br
6
210 200 190 180 170 160 150 140 130 120 110 100 90 80 70 60 50 40 30 20 10 ppm

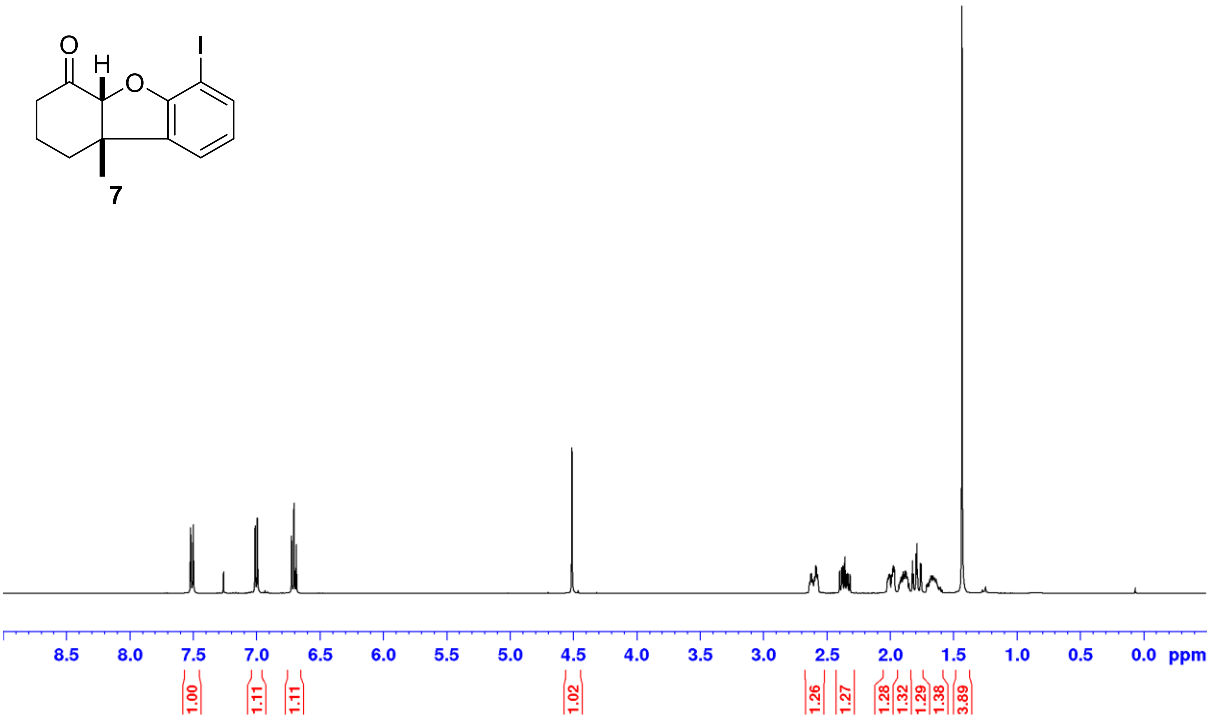
O
H
O
I
7
8.5 8.0 7.5 7.0 6.5 6.0 5.5 5.0 4.5 4.0 3.5 3.0 2.5 2.0 1.5 1.0 0.5 0.0 ppm
1.00
1.11
1.11
1.02
1.26
1.27
1.28
1.32
1.29
1.38
3.89

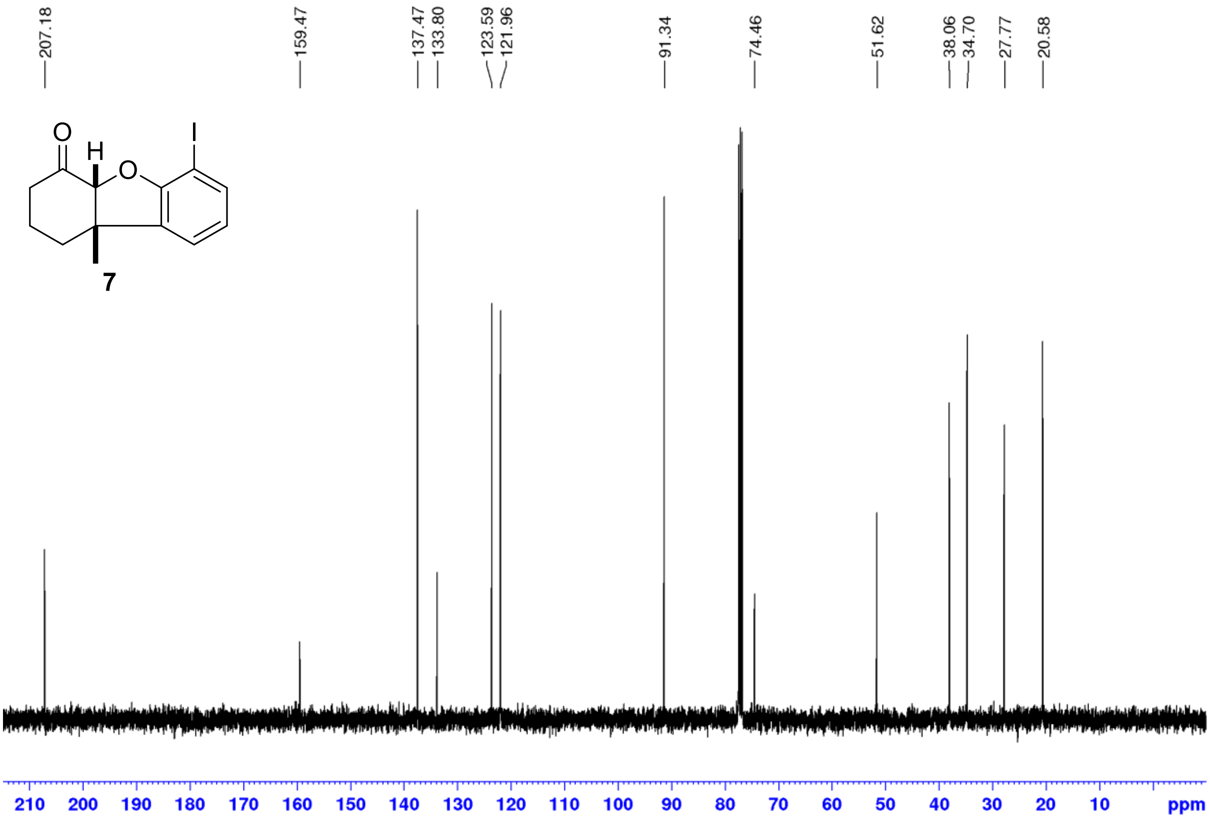

207.18
159.47
137.47
133.80
123.59
121.96
91.34
74.46
51.62
38.06
34.70
27.77
20.58
O
H
O
I
210 200 190 180 170 160 150 140 130 120 110 100 90 80 70 60 50 40 30 20 10 ppm

7

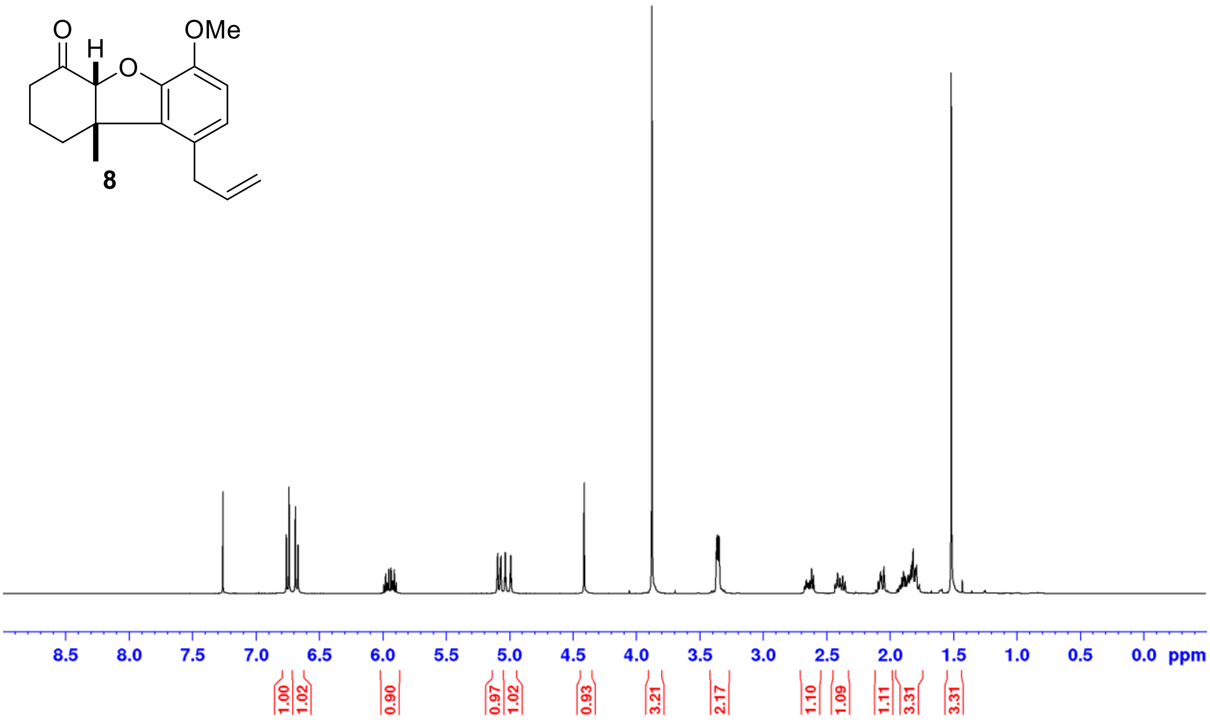

O
H
O
OMe
8.5 8.0 7.5 7.0 6.5 6.0 5.5 5.0 4.5 4.0 3.5 3.0 2.5 2.0 1.5 1.0 0.5 0.0 ppm
1.00
1.02
0.90
0.97
1.02
0.93
3.21
2.17
1.10
1.09
1.11
3.31
3.31

8

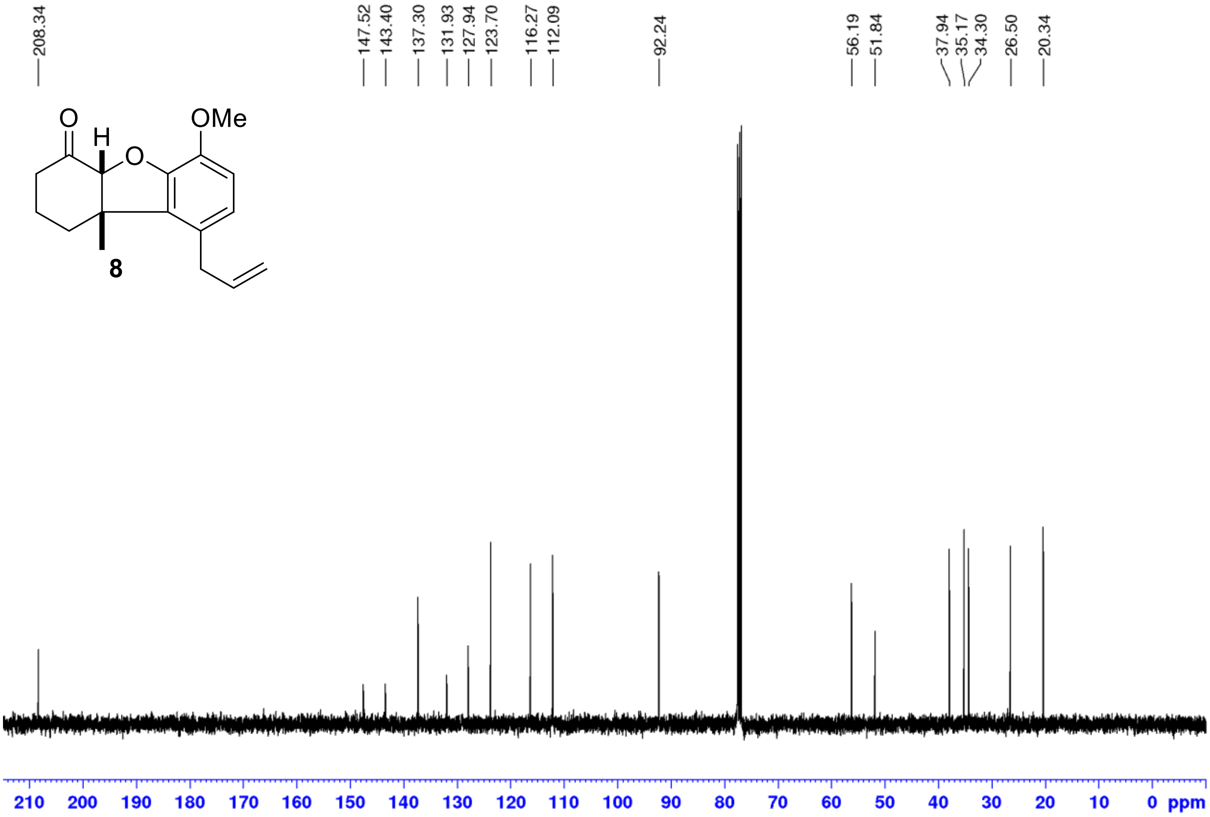
208.34
147.52
143.40
137.30
131.93
127.94
123.70
116.27
112.09
92.24
56.19
51.84
37.94
35.17
34.30
26.50
20.34
O
H
O
OMe
8
210 200 190 180 170 160 150 140 130 120 110 100 90 80 70 60 50 40 30 20 10 0 ppm

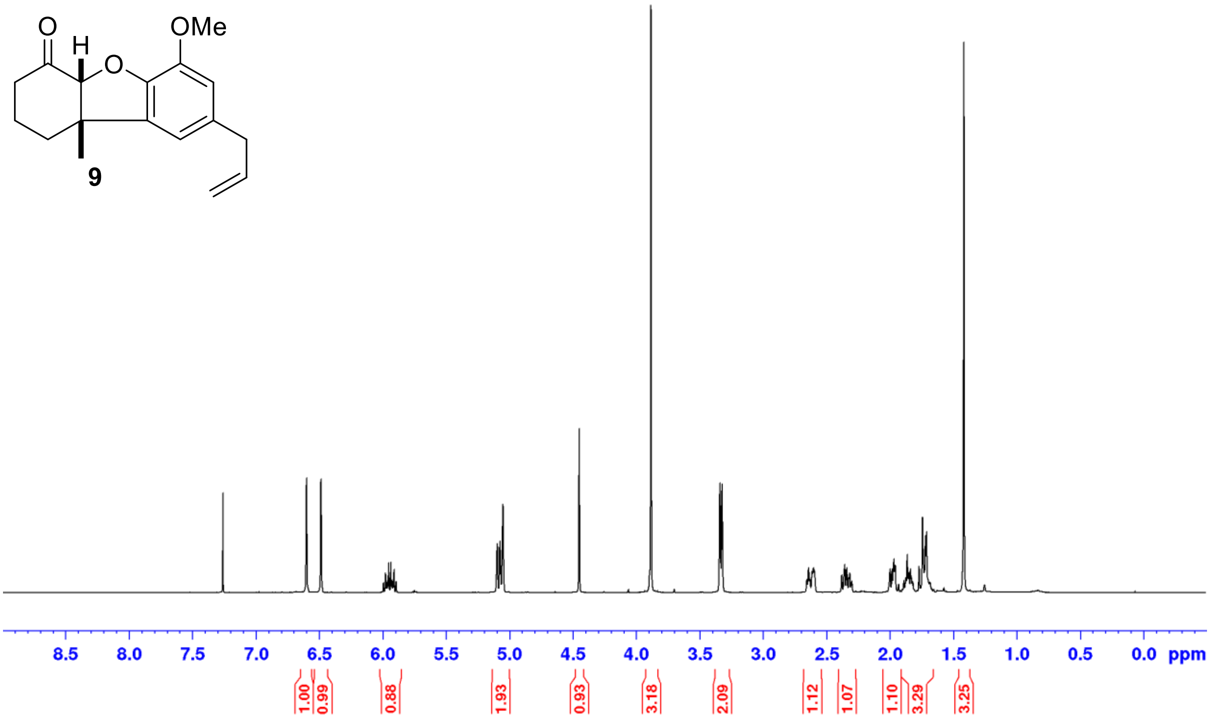
O
H
O
OMe
9
8.5 8.0 7.5 7.0 6.5 6.0 5.5 5.0 4.5 4.0 3.5 3.0 2.5 2.0 1.5 1.0 0.5 0.0 ppm
1.00
0.99
0.88
1.93
0.93
3.18
2.09
1.12
1.07
1.10
3.29
3.25

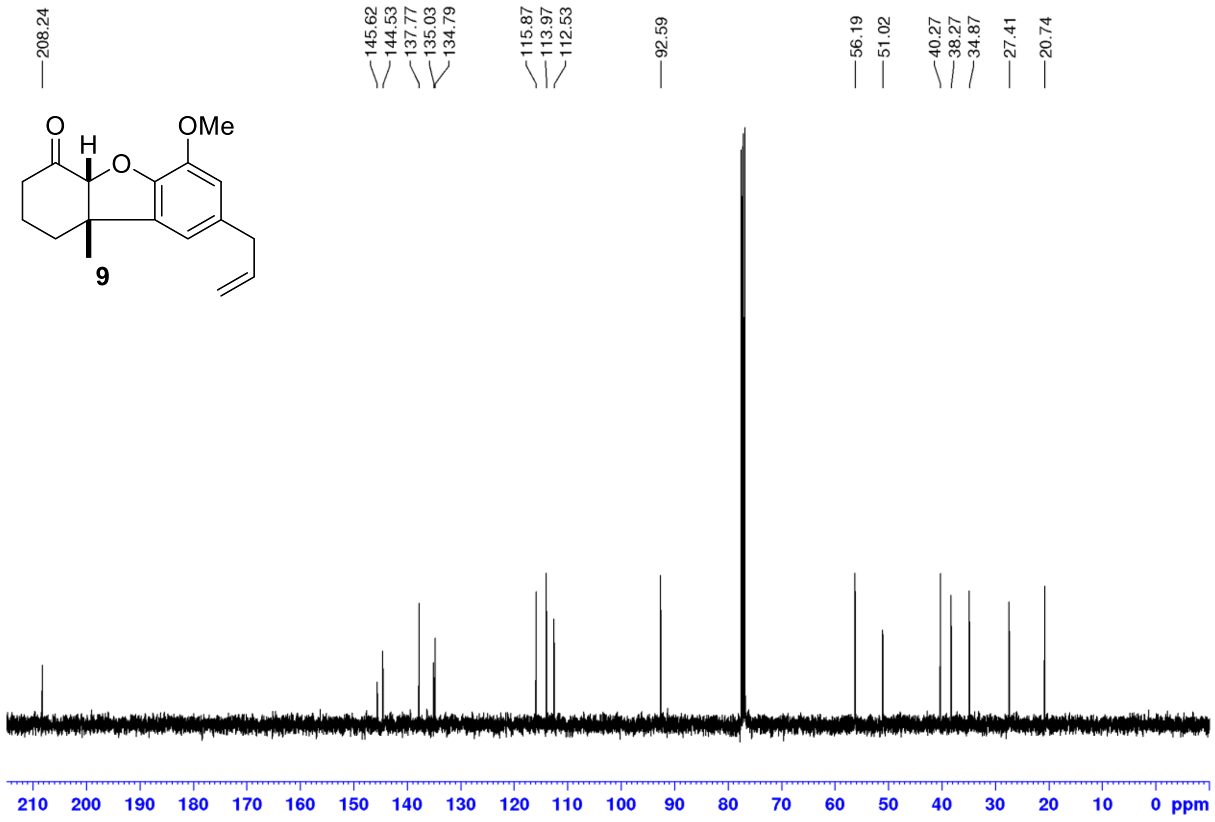
208.24
145.62
144.53
137.77
135.03
134.79
115.87
113.97
112.53
92.59
56.19
51.02
40.27
38.27
34.87
27.41
20.74
OMe
H
O
O
9
210 200 190 180 170 160 150 140 130 120 110 100 90 80 70 60 50 40 30 20 10 0 ppm

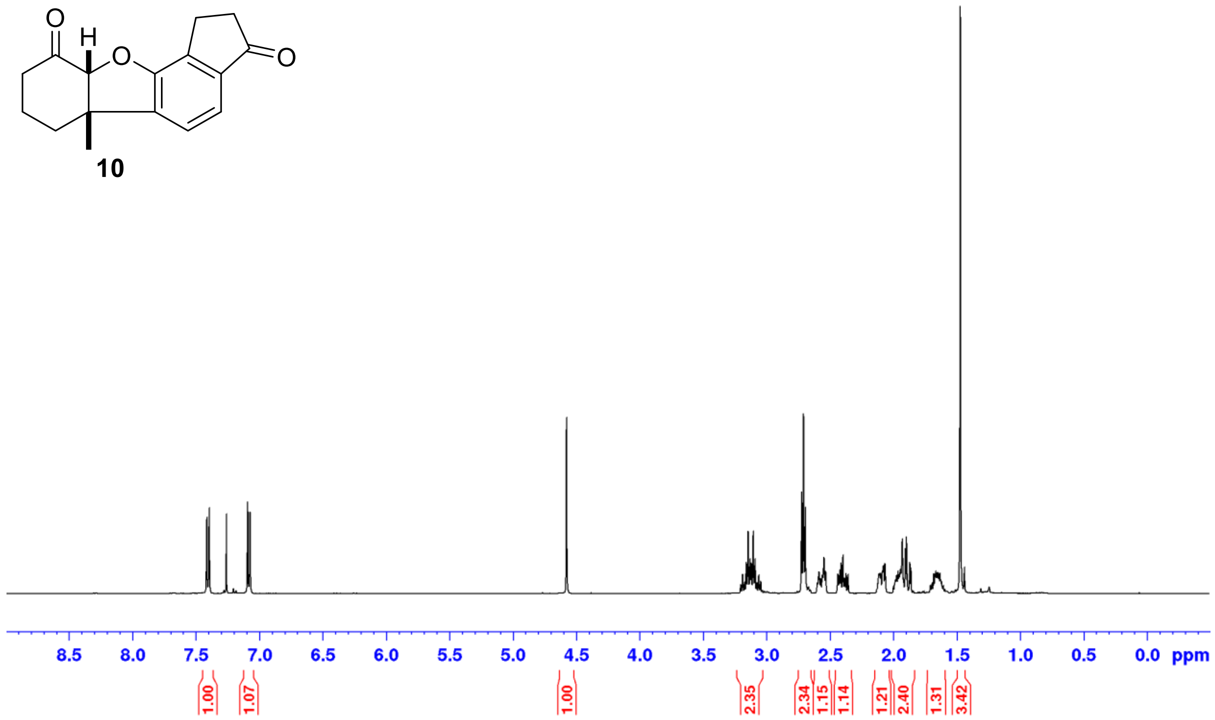
O
H
O
O
10
8.5 8.0 7.5 7.0 6.5 6.0 5.5 5.0 4.5 4.0 3.5 3.0 2.5 2.0 1.5 1.0 0.5 0.0 ppm
1.00
1.07
1.00
2.35
2.34
1.15
1.14
1.21
2.40
1.31
3.42

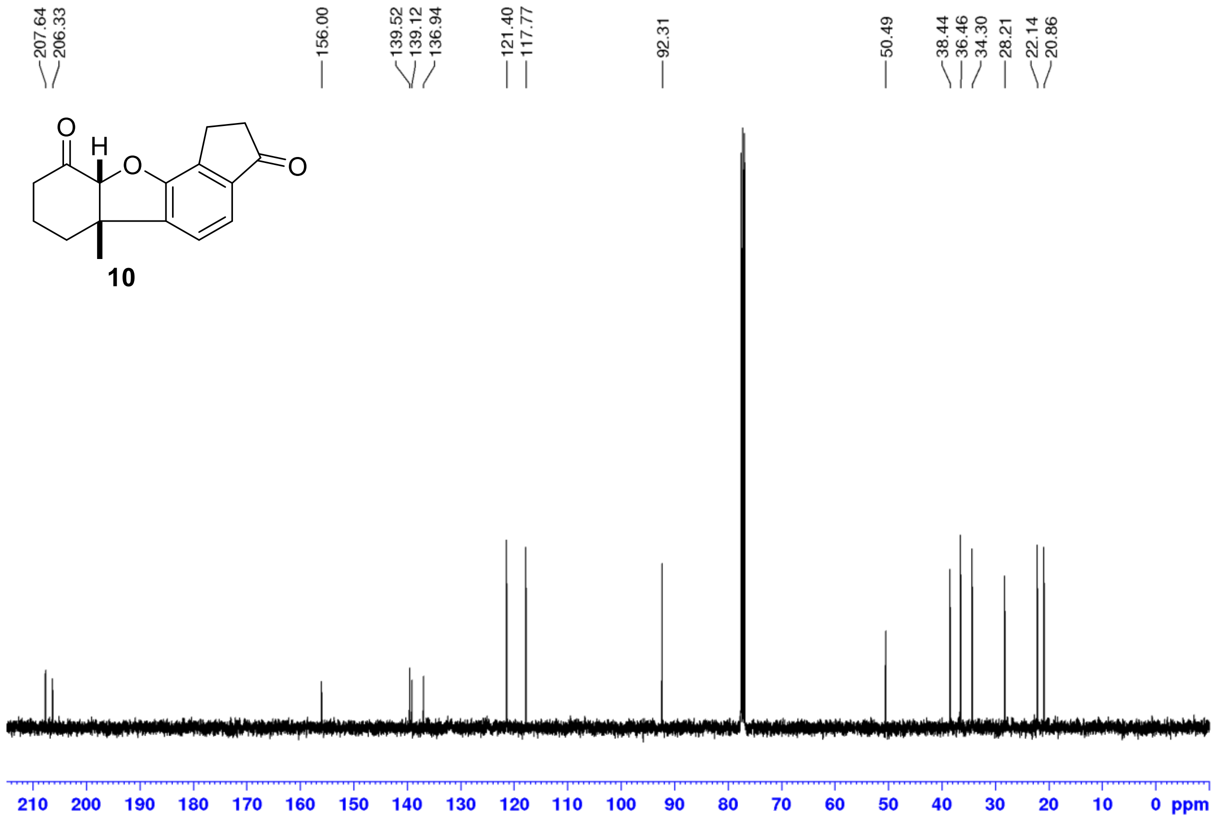
207.64
206.33
156.00
139.52
139.12
136.94
121.40
117.77
92.31
50.49
38.44
36.46
34.30
28.21
22.14
20.86
O
H
O
O
10
210
200
190
180
170
160
150
140
130
120
110
100
90
80
70
60
50
40
30
20
10
0
ppm

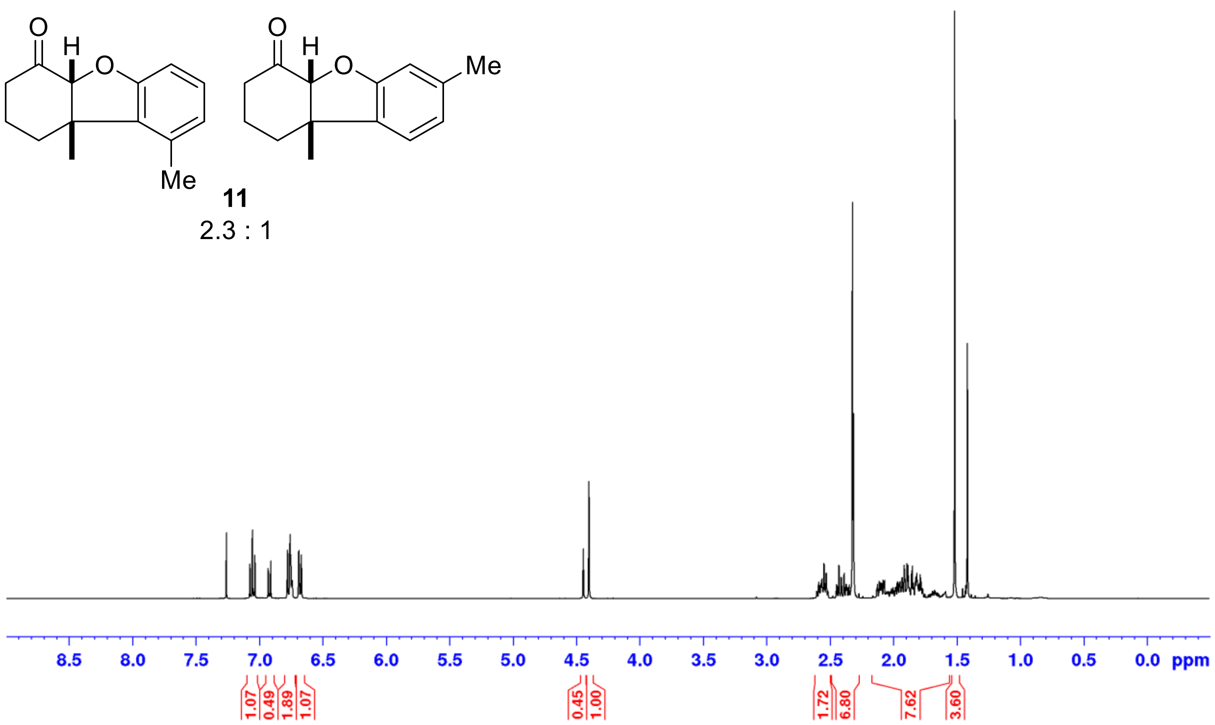
O
H
O
Me
O
H
O
Me
11
2.3 : 1
8.5
8.0
7.5
7.0
6.5
6.0
5.5
5.0
4.5
4.0
3.5
3.0
2.5
2.0
1.5
1.0
0.5
0.0
ppm
1.07
0.49
1.89
1.07
0.45
1.00
1.72
6.80
7.62
3.60

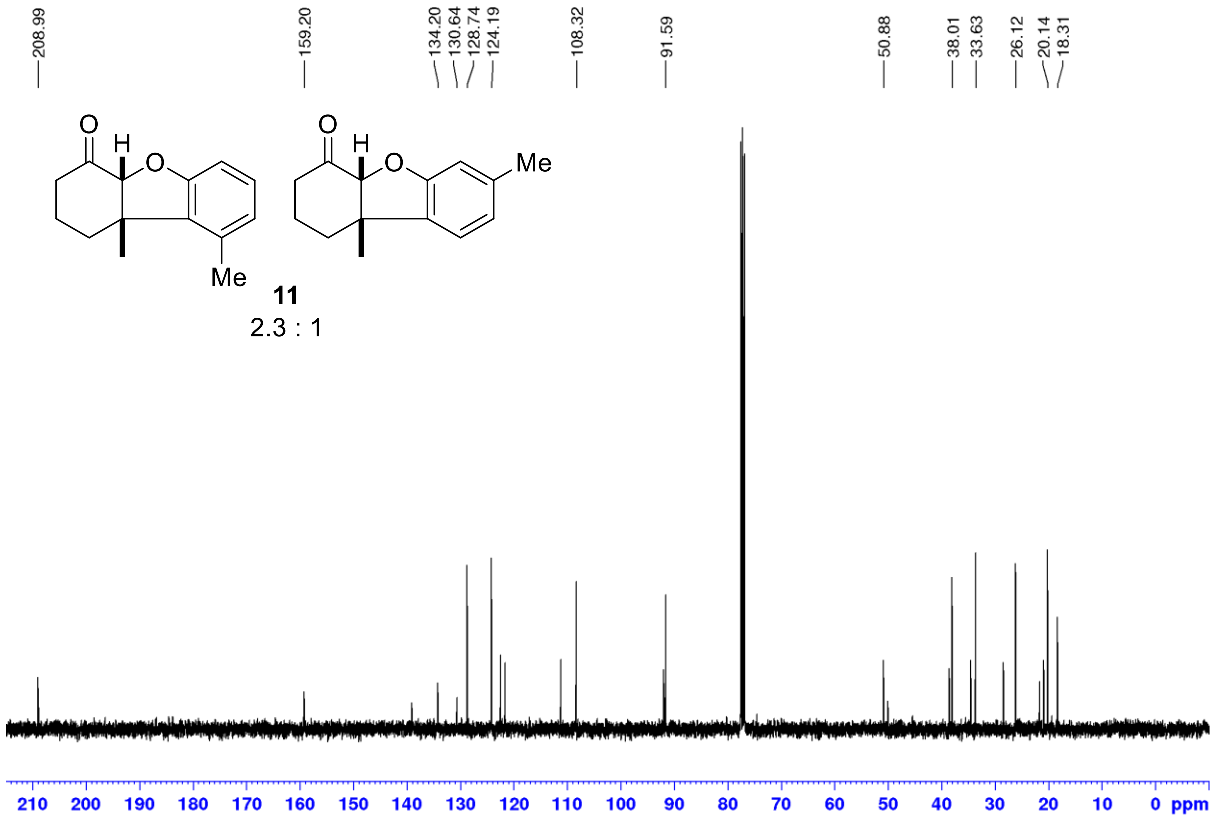

208.99
159.20
134.20
130.64
128.74
124.19
108.32
91.59
50.88
38.01
33.63
26.12
20.14
18.31
O
H
O
Me
11
2.3 : 1
210 200 190 180 170 160 150 140 130 120 110 100 90 80 70 60 50 40 30 20 10 0 ppm

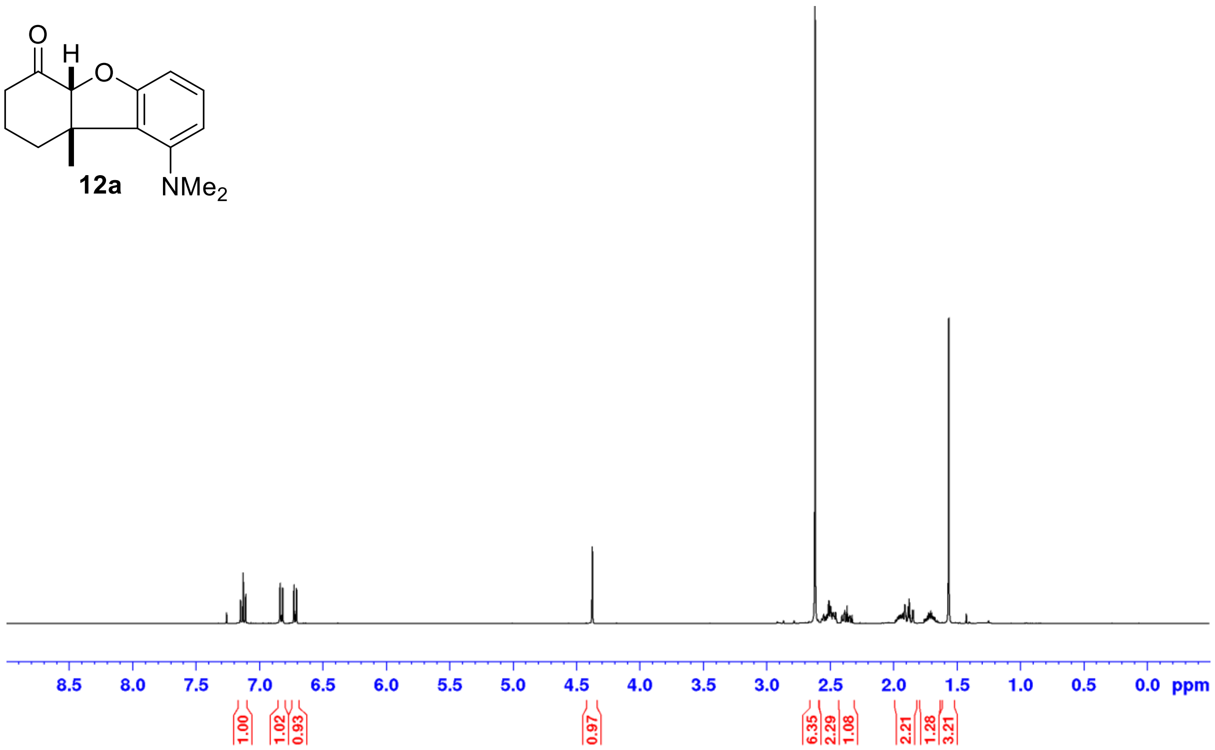

O
H
O
12a
NMe2
8.5 8.0 7.5 7.0 6.5 6.0 5.5 5.0 4.5 4.0 3.5 3.0 2.5 2.0 1.5 1.0 0.5 0.0 ppm
1.00
1.02
0.93
0.97
6.35
2.29
1.08
2.21
1.28
3.21

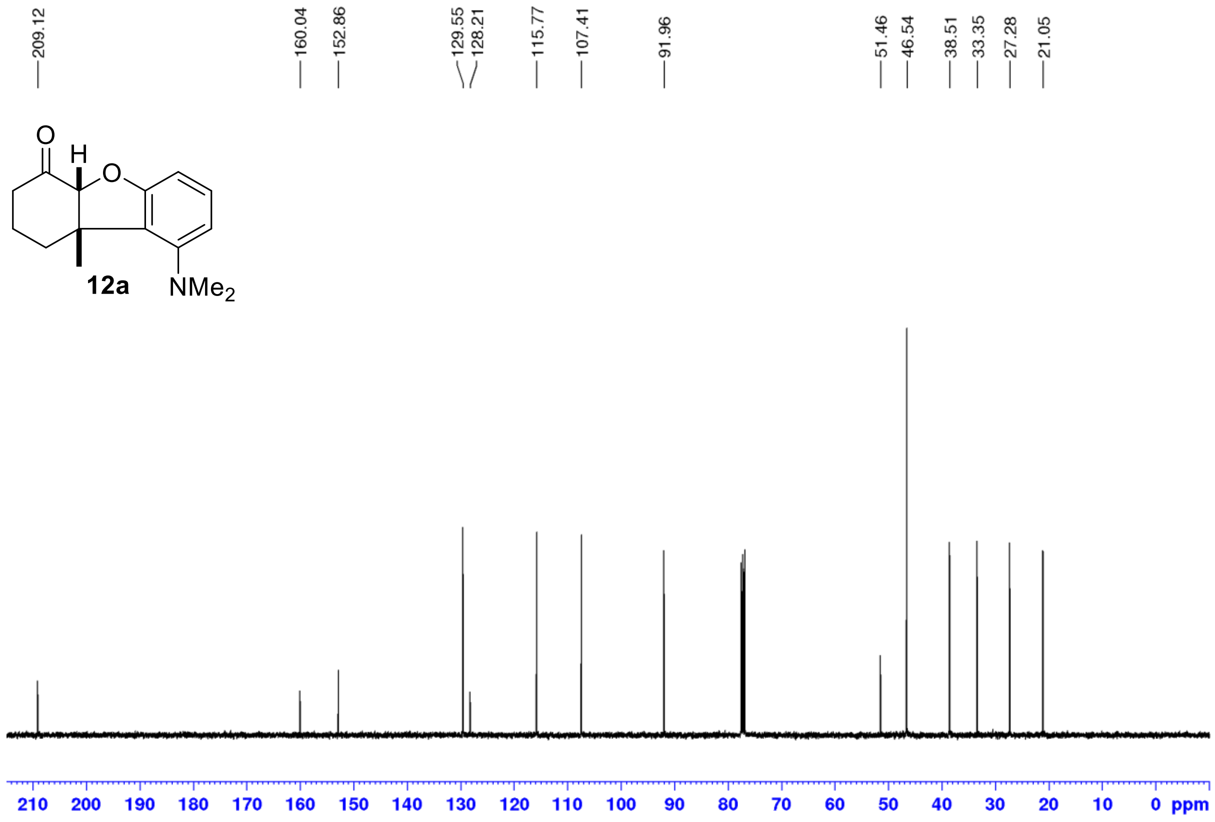
209.12
160.04
152.86
129.55
128.21
115.77
107.41
91.96
51.46
46.54
38.51
33.35
27.28
21.05
O
H
O
NMe2
210 200 190 180 170 160 150 140 130 120 110 100 90 80 70 60 50 40 30 20 10 0 ppm

12a

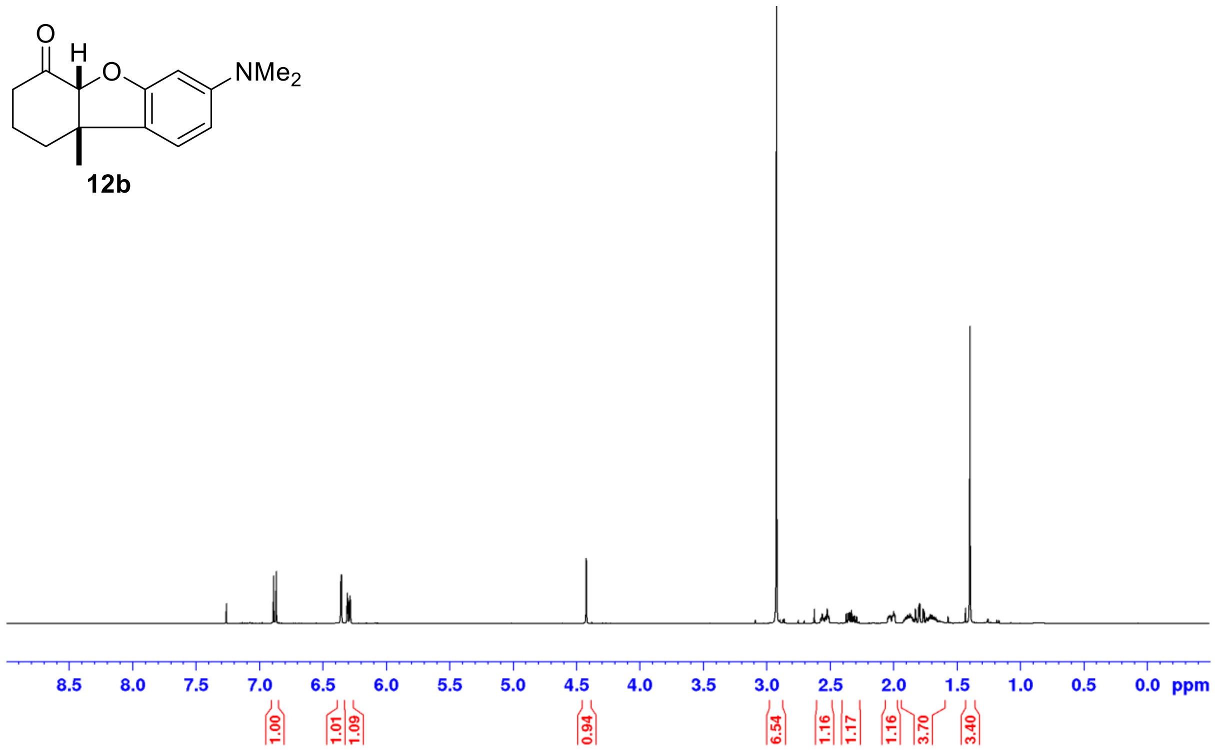
O
H
O
NMe2
8.5 8.0 7.5 7.0 6.5 6.0 5.5 5.0 4.5 4.0 3.5 3.0 2.5 2.0 1.5 1.0 0.5 0.0 ppm
1.00
1.01
1.09
0.94
6.54
1.16
1.17
1.16
3.70
3.40

12b

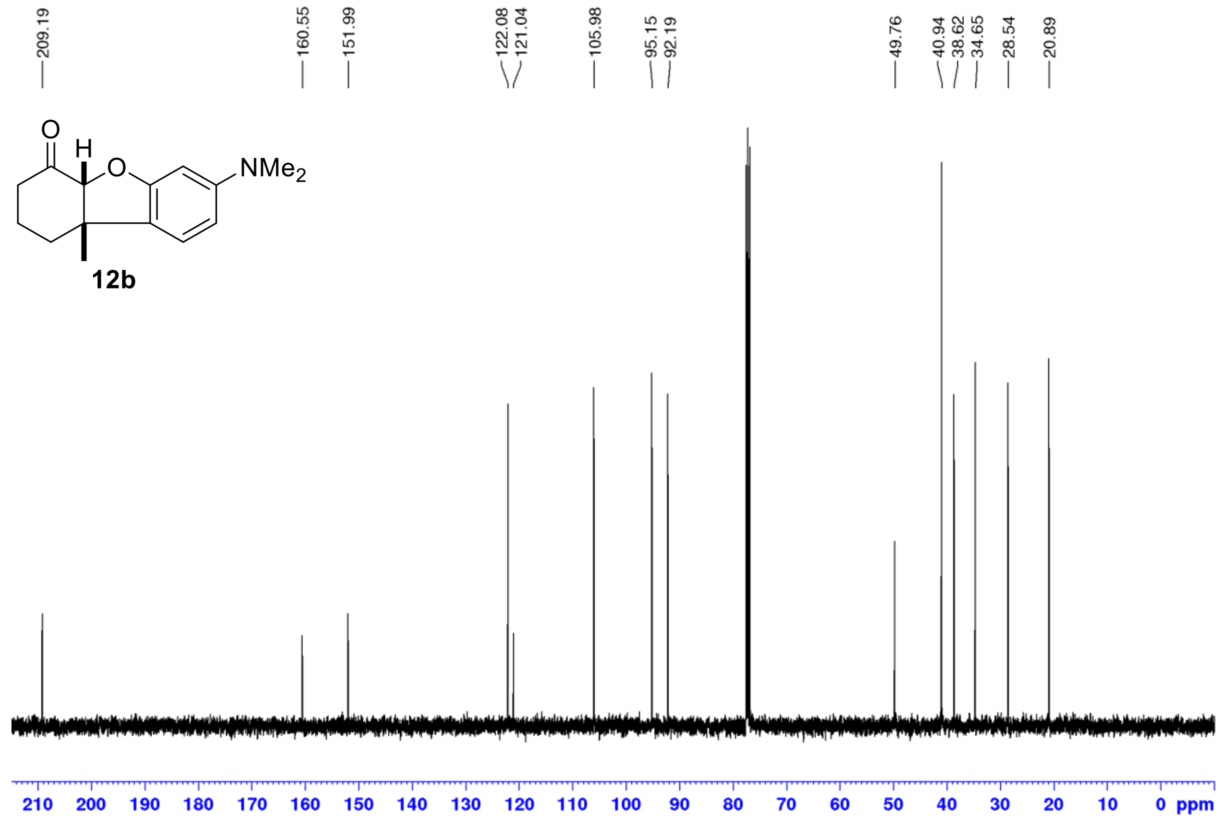
209.19
160.55
151.99
122.08
121.04
105.98
95.15
92.19
49.76
40.94
38.62
34.65
28.54
20.89
NMe2
12b
ppm

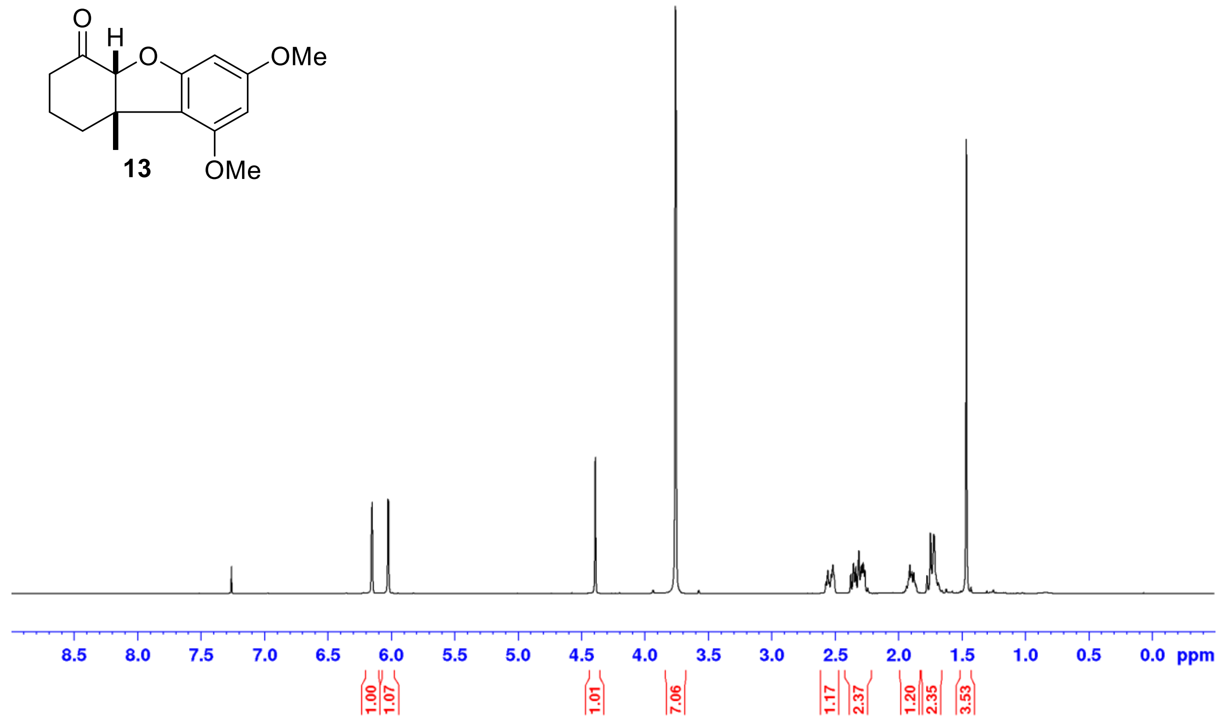
OMe
OMe
13
ppm
1.00
1.07
1.01
7.06
1.17
2.37
1.20
2.35
3.53

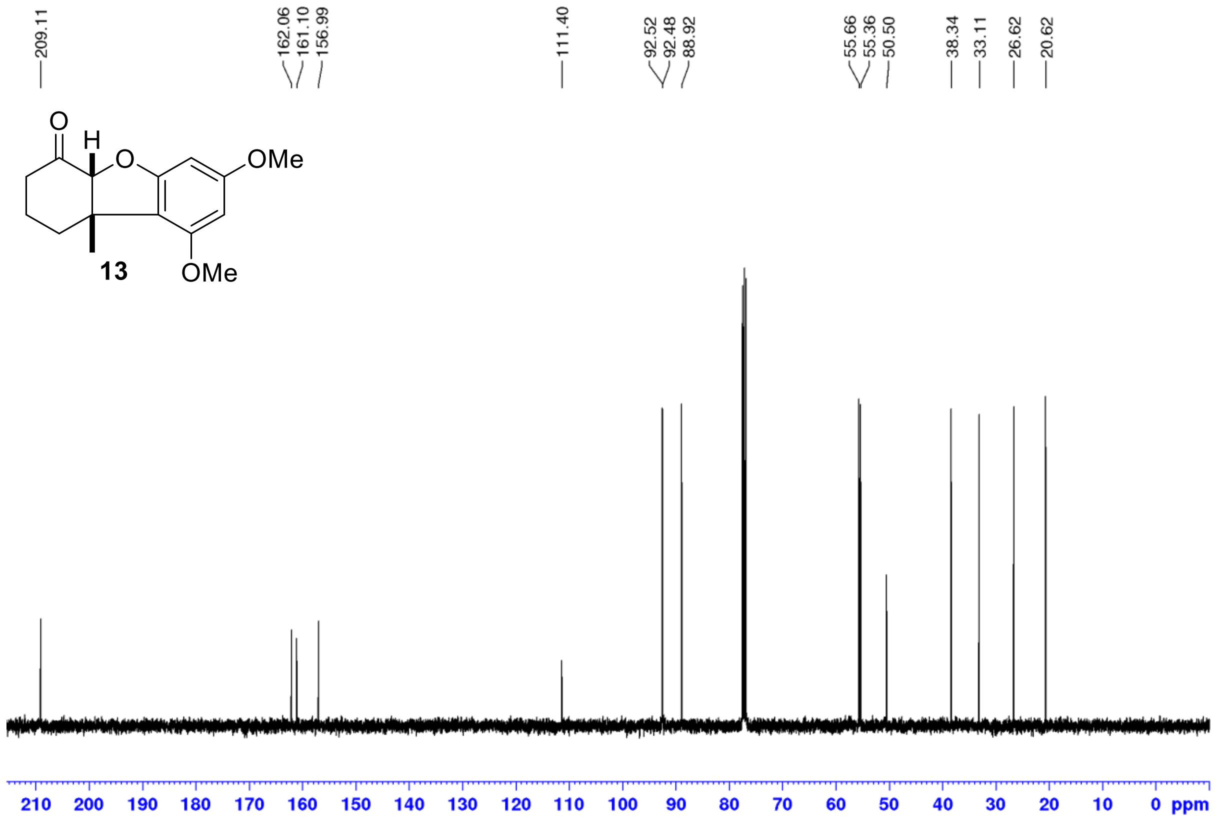
209.11
162.06
161.10
156.99
111.40
92.52
92.48
88.92
55.66
55.36
50.50
38.34
33.11
26.62
20.62
O
H
O
OMe
13
OMe
210 200 190 180 170 160 150 140 130 120 110 100 90 80 70 60 50 40 30 20 10 0 ppm

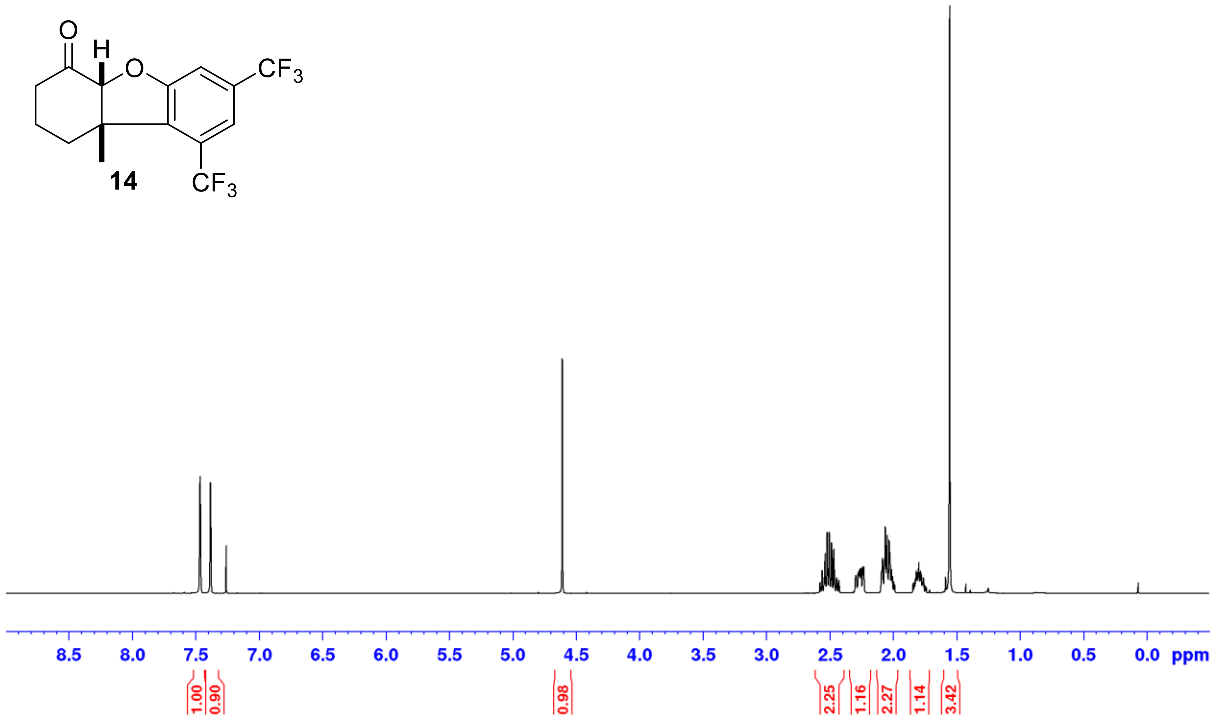
O
H
O
CF3
14
CF3
8.5 8.0 7.5 7.0 6.5 6.0 5.5 5.0 4.5 4.0 3.5 3.0 2.5 2.0 1.5 1.0 0.5 0.0 ppm
1.00
0.90
0.98
2.25
1.16
2.27
1.14
3.42

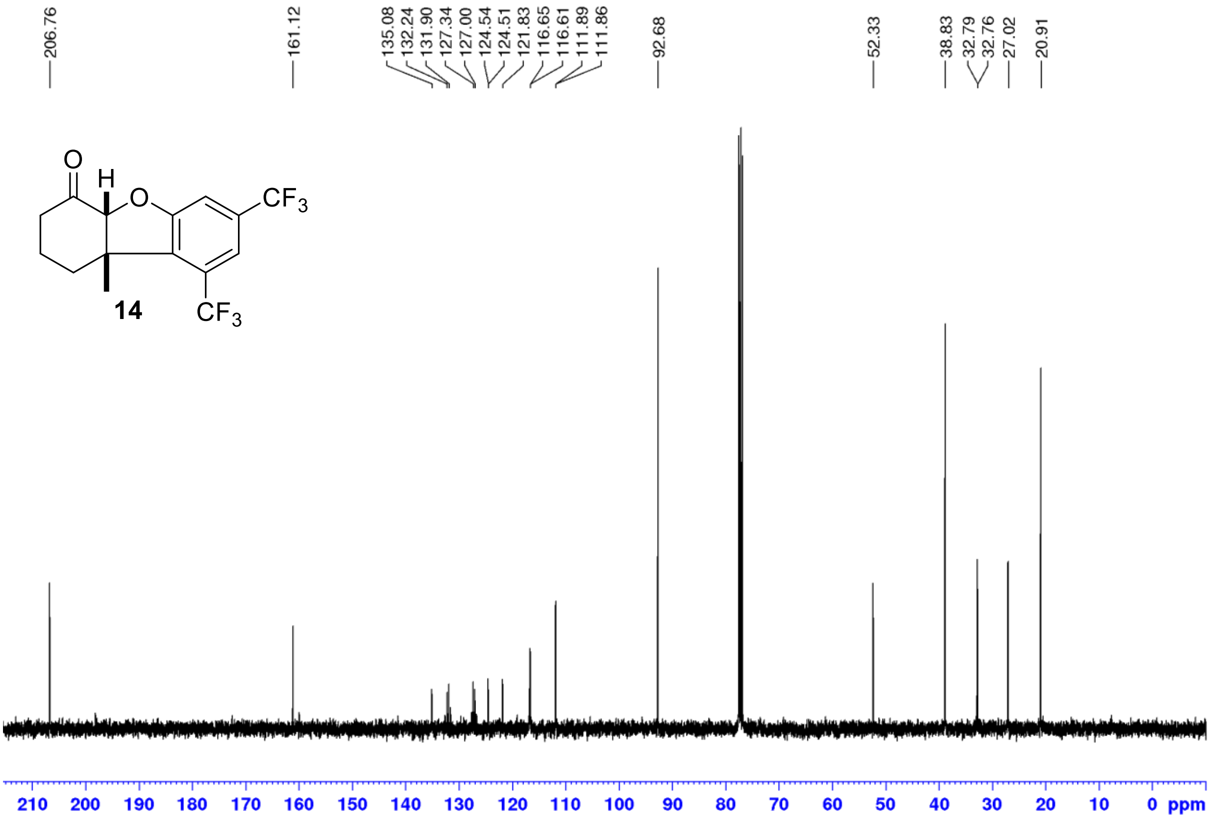
206.76
161.12
135.08
132.24
131.90
127.34
127.00
124.54
124.51
121.83
116.65
116.61
111.89
111.86
92.68
52.33
38.83
32.79
32.76
27.02
20.91
O
H
O
CF3
CF3
14
210
200
190
180
170
160
150
140
130
120
110
100
90
80
70
60
50
40
30
20
10
0
ppm

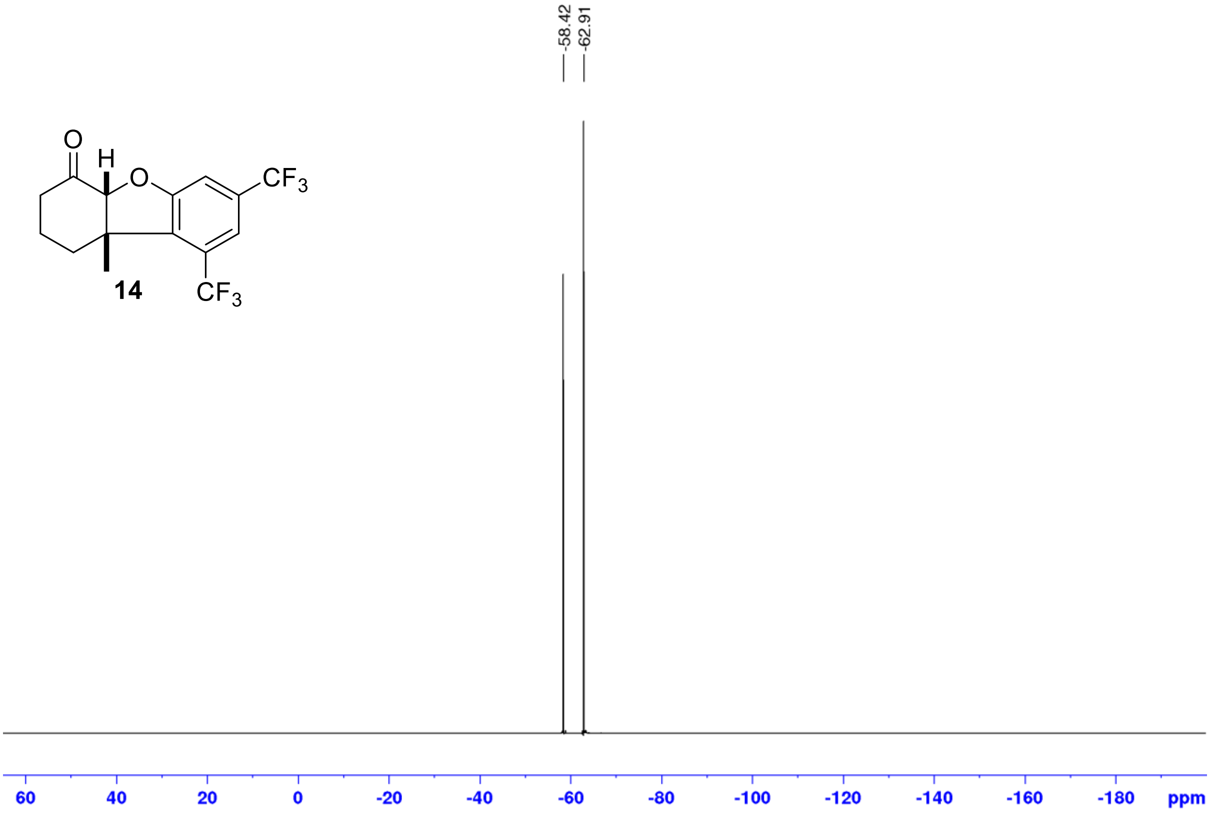
-58.42
-62.91
O
H
O
CF3
CF3
14
60
40
20
0
-20
-40
-60
-80
-100
-120
-140
-160
-180
ppm

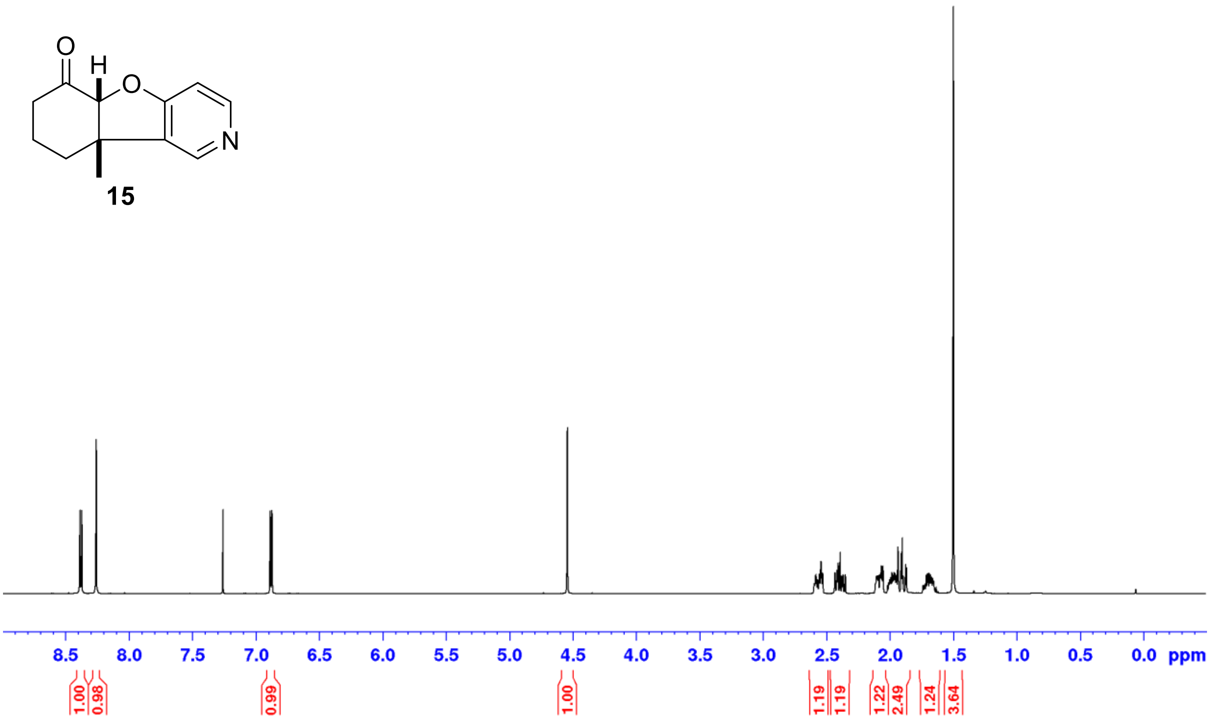
15
8.5 8.0 7.5 7.0 6.5 6.0 5.5 5.0 4.5 4.0 3.5 3.0 2.5 2.0 1.5 1.0 0.5 0.0 ppm
1.00 0.98 0.99 1.00 1.19 1.19 1.22 2.49 1.24 3.64

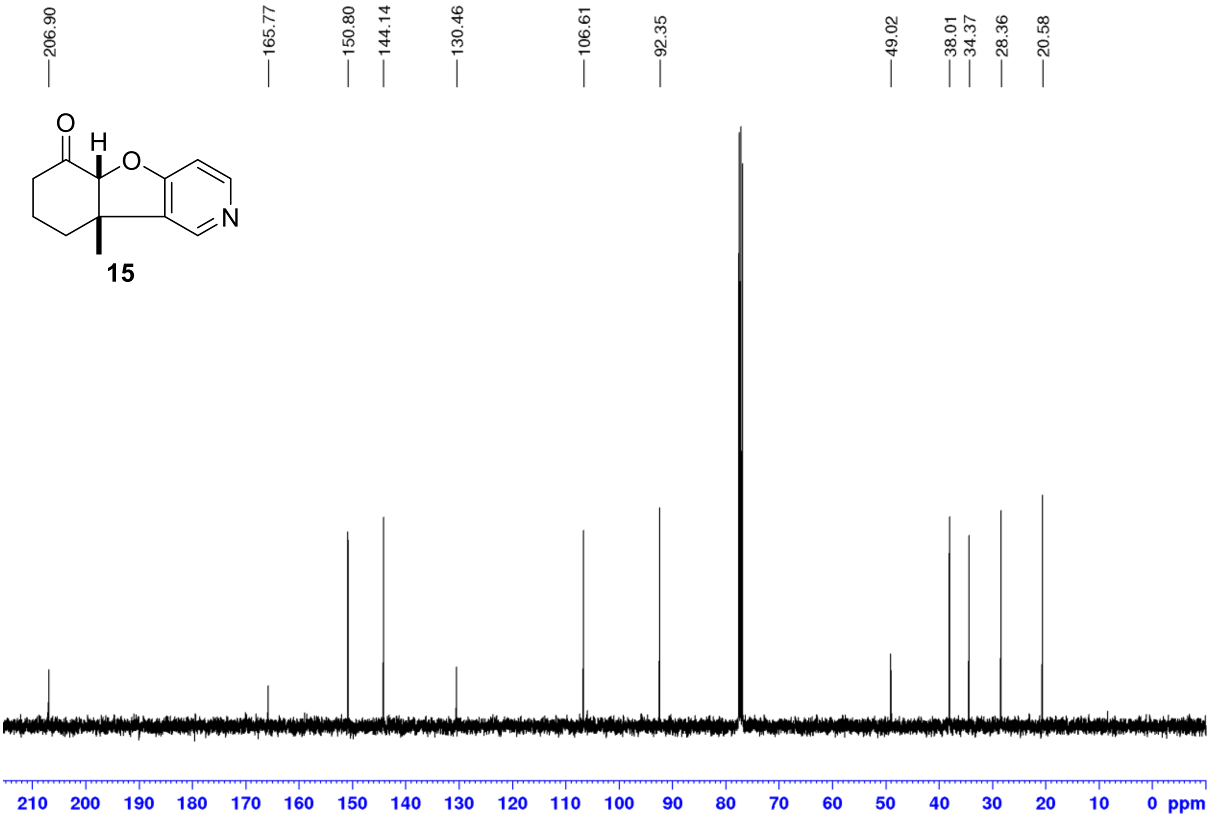
206.90 165.77 150.80 144.14 130.46 106.61 92.35 49.02 38.01 34.37 28.36 20.58
15
210 200 190 180 170 160 150 140 130 120 110 100 90 80 70 60 50 40 30 20 10 0 ppm

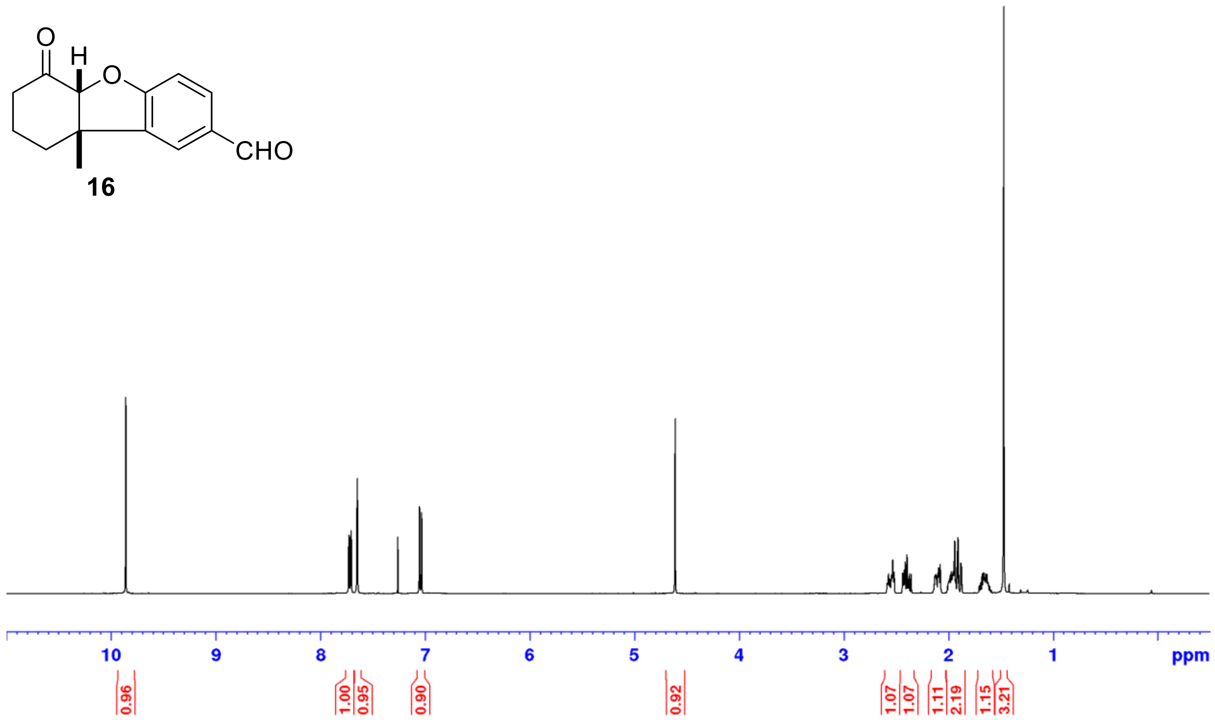
O
H
O
CHO
16
10
9
8
7
6
5
4
3
2
1
ppm
0.96
1.00
0.95
0.90
0.92
1.07
1.07
1.11
2.19
1.15
3.21

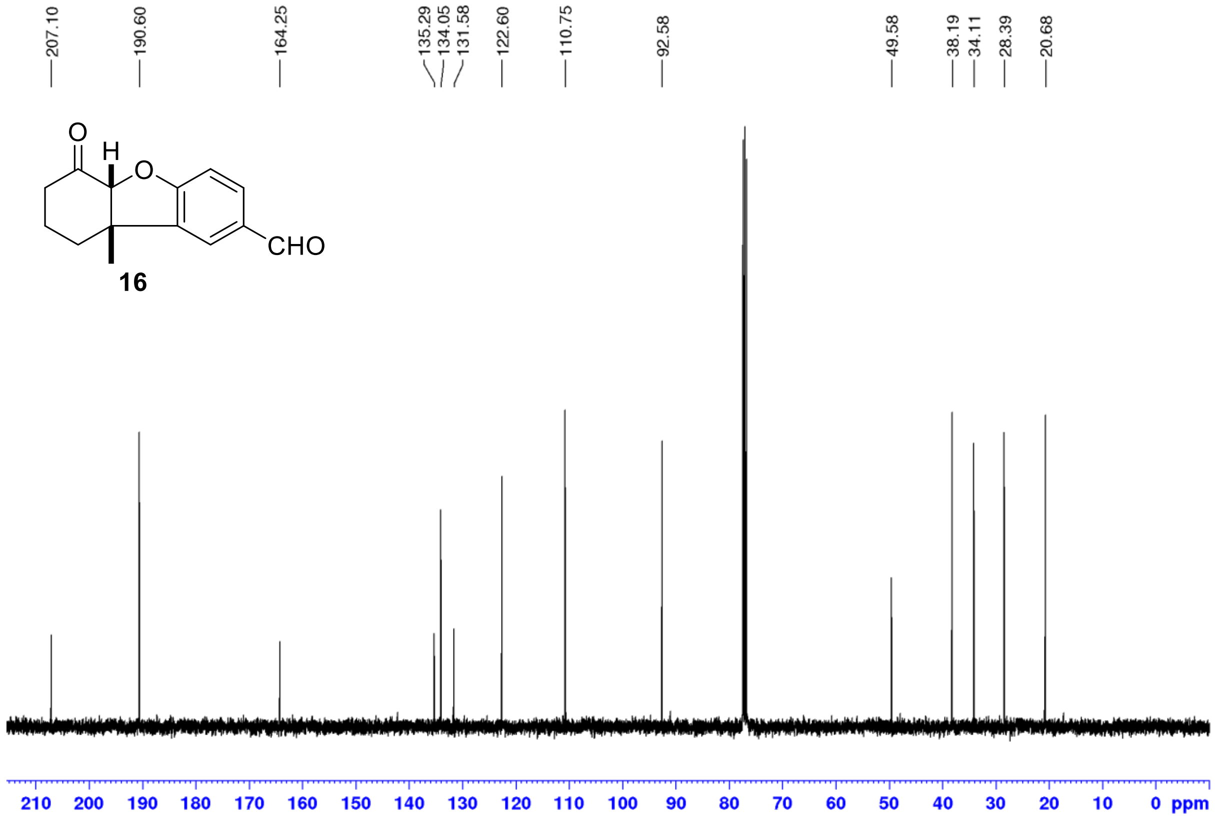
207.10
190.60
164.25
135.29
134.05
131.58
122.60
110.75
92.58
49.58
38.19
34.11
28.39
20.68
O
H
O
CHO
16
210
200
190
180
170
160
150
140
130
120
110
100
90
80
70
60
50
40
30
20
10
0 ppm

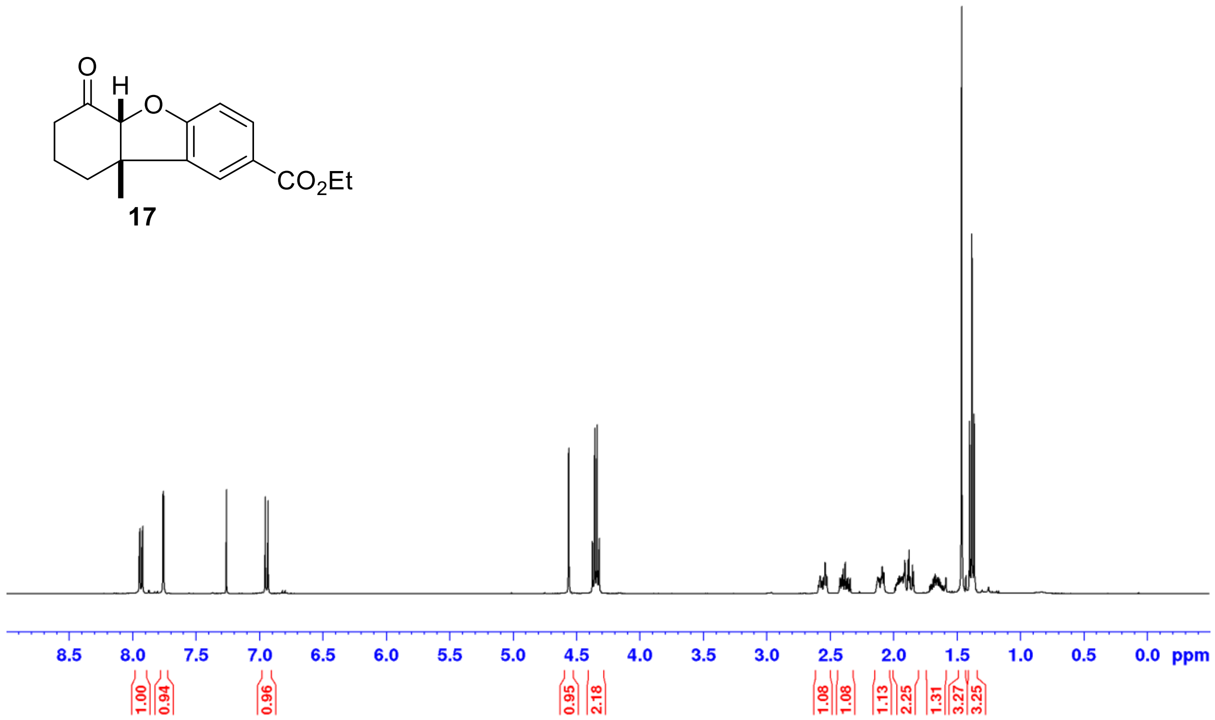
O
H
O
CO_2Et
17
8.5
8.0
7.5
7.0
6.5
6.0
5.5
5.0
4.5
4.0
3.5
3.0
2.5
2.0
1.5
1.0
0.5
0.0
ppm
1.00
0.94
0.96
0.95
2.18
1.08
1.08
1.13
2.25
1.31
3.27
3.25

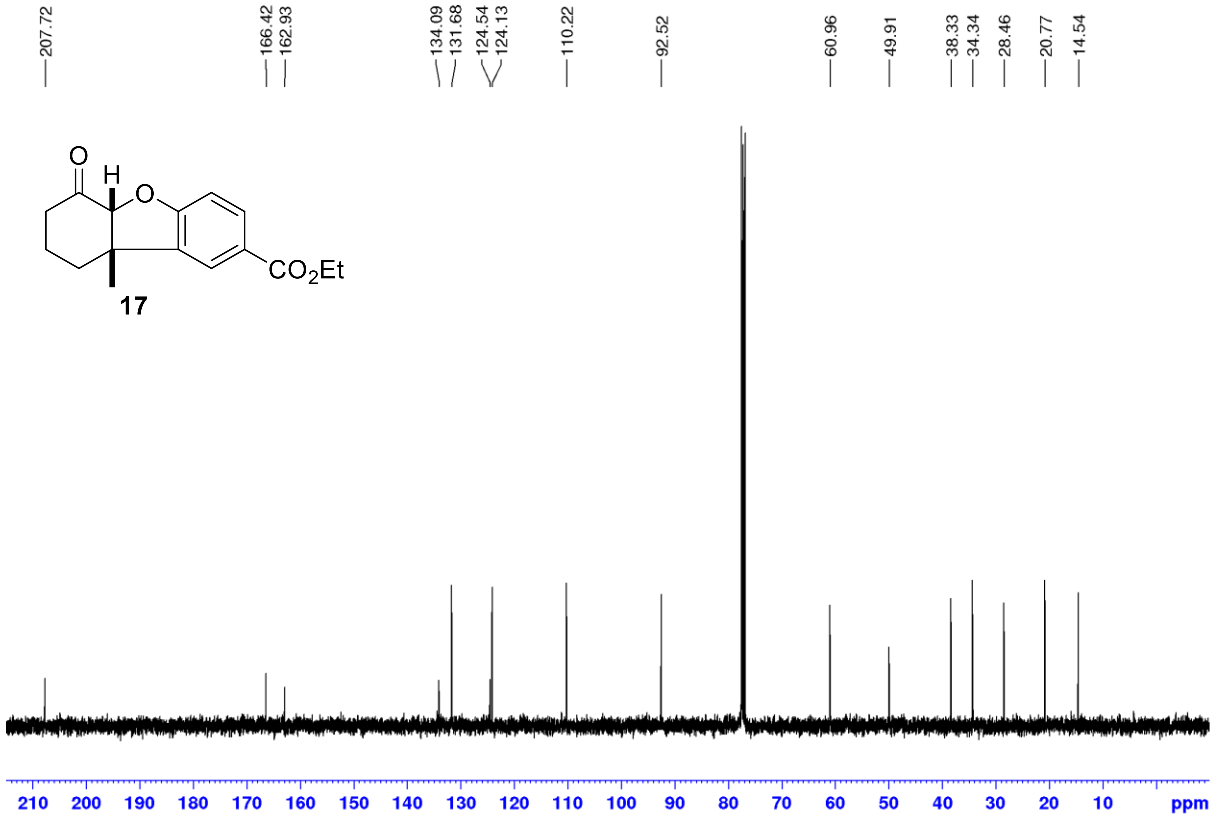
207.72
166.42
162.93
134.09
131.68
124.54
124.13
110.22
92.52
60.96
49.91
38.33
34.34
28.46
20.77
14.54
O
H
O
CO_2Et
17
210
200
190
180
170
160
150
140
130
120
110
100
90
80
70
60
50
40
30
20
10
ppm

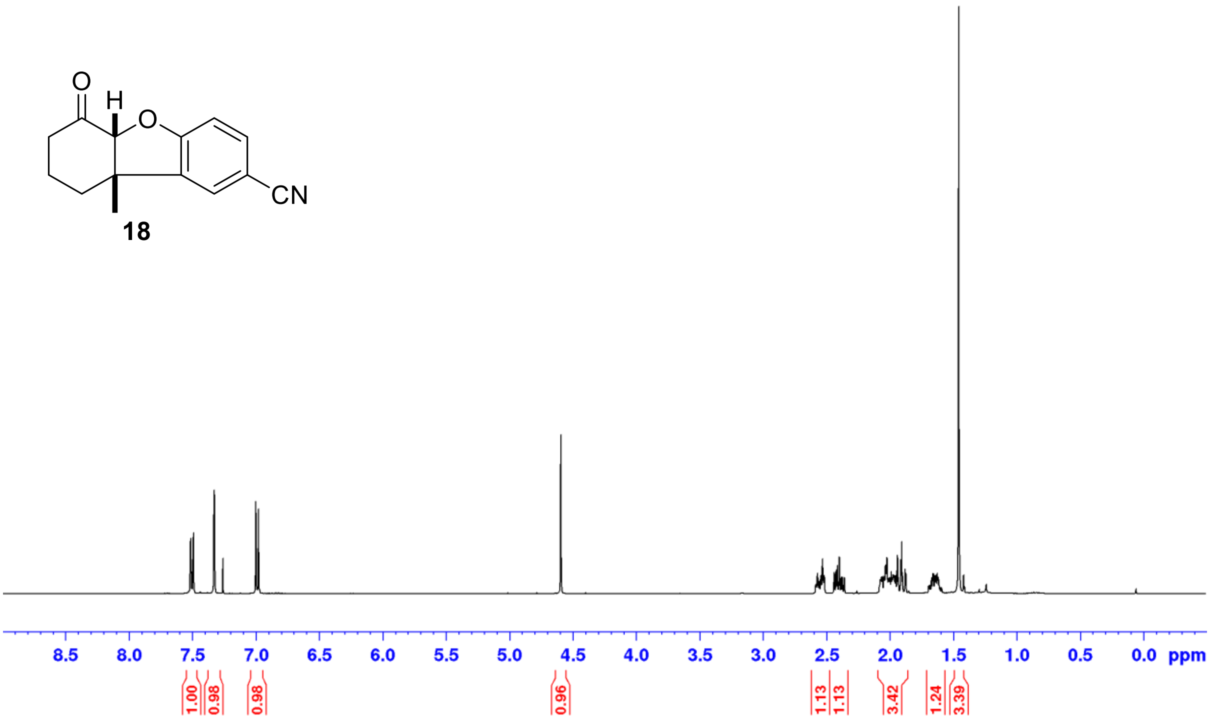
O
H
O
CN
18
8.5 8.0 7.5 7.0 6.5 6.0 5.5 5.0 4.5 4.0 3.5 3.0 2.5 2.0 1.5 1.0 0.5 0.0 ppm
1.00
0.98
0.98
0.96
1.13
1.13
3.42
1.24
3.39

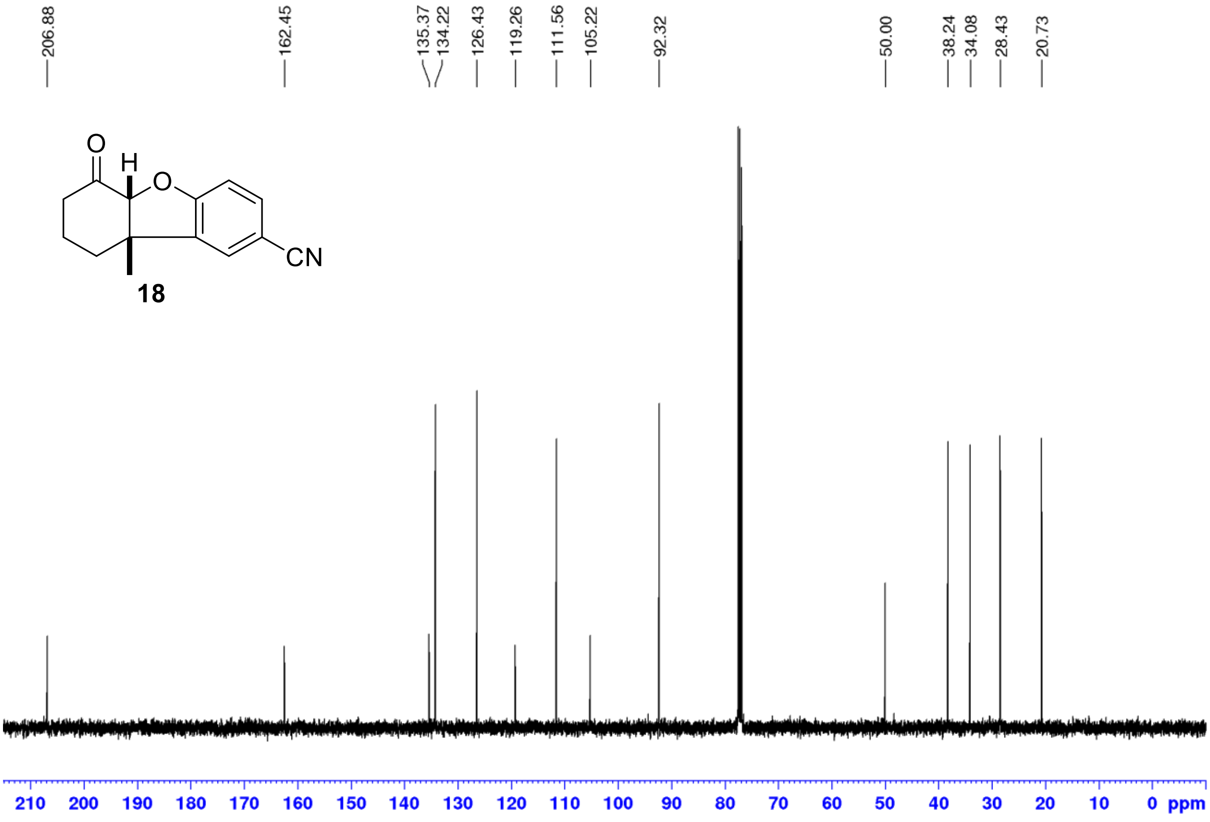
206.88
162.45
135.37
134.22
126.43
119.26
111.56
105.22
92.32
50.00
38.24
34.08
28.43
20.73
O
H
O
CN
18
210 200 190 180 170 160 150 140 130 120 110 100 90 80 70 60 50 40 30 20 10 0 ppm

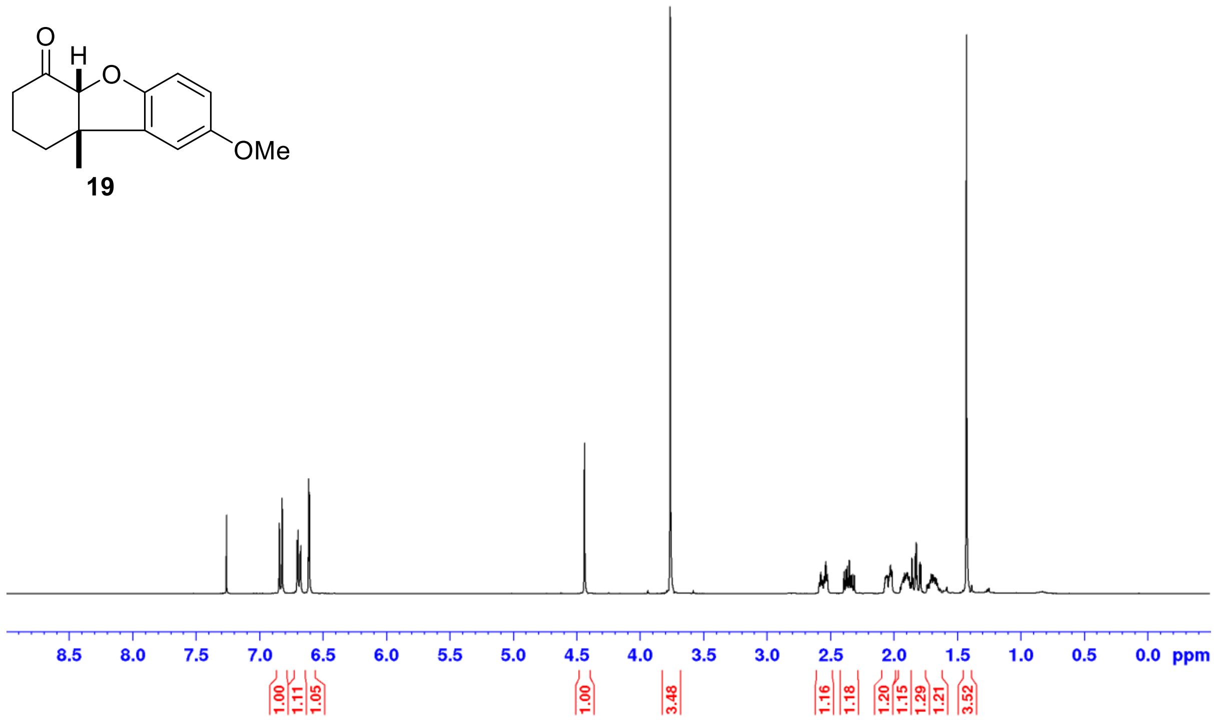
O
H
O
OMe
19
8.5
8.0
7.5
7.0
6.5
6.0
5.5
5.0
4.5
4.0
3.5
3.0
2.5
2.0
1.5
1.0
0.5
0.0 ppm
1.00
1.11
1.05
1.00
3.48
1.16
1.18
1.20
1.15
1.29
1.21
3.52

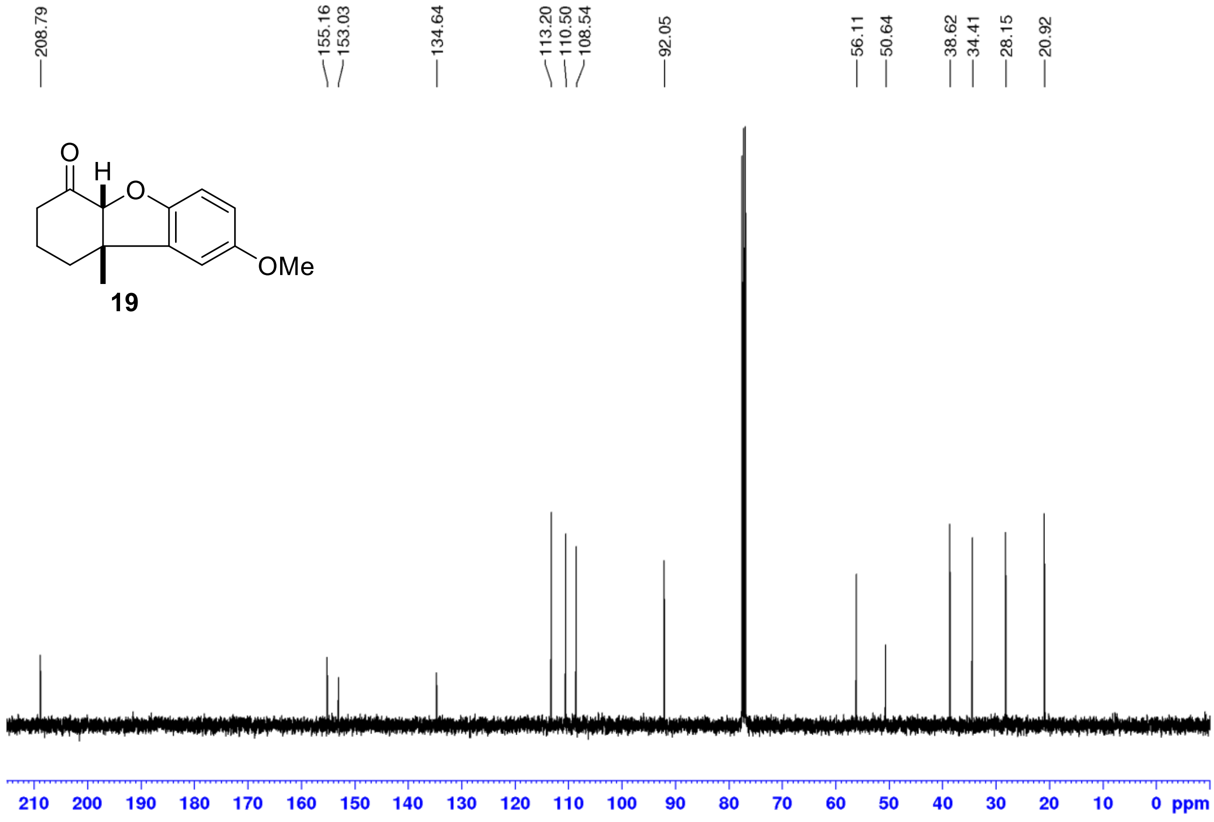
208.79
155.16
153.03
134.64
113.20
110.50
108.54
92.05
56.11
50.64
38.62
34.41
28.15
20.92
O
H
O
OMe
19
210
200
190
180
170
160
150
140
130
120
110
100
90
80
70
60
50
40
30
20
10
0 ppm

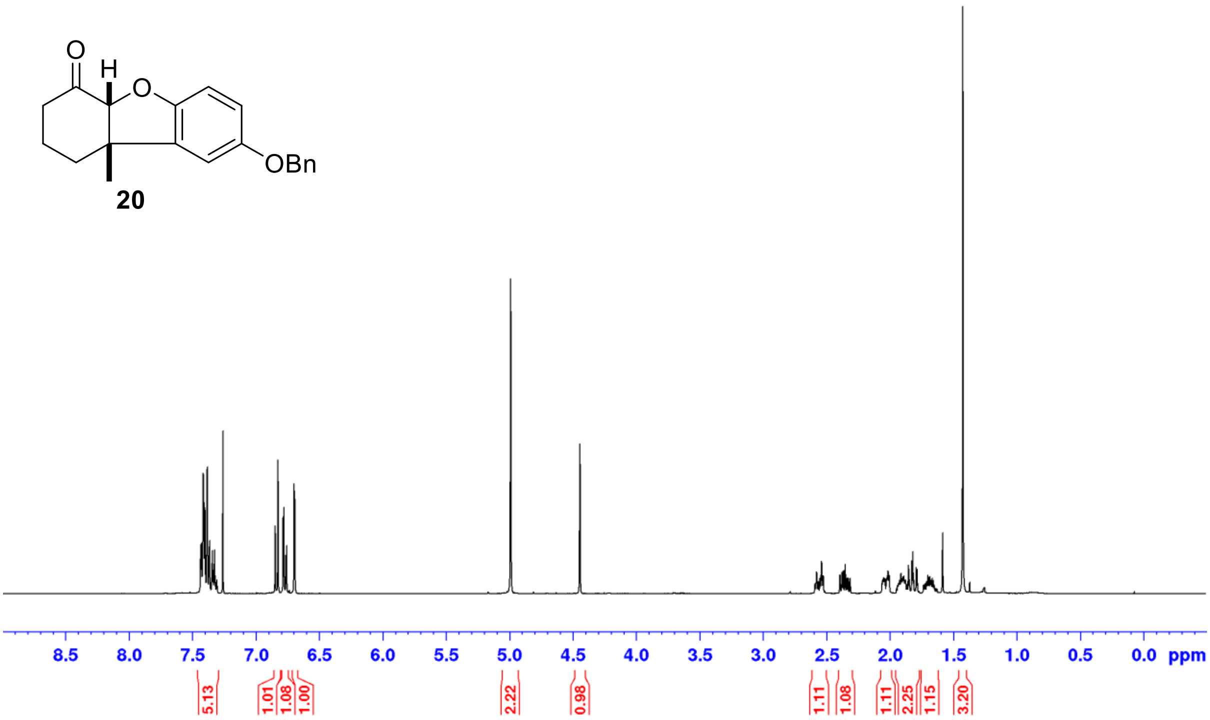

O
H
O
OBn
20
8.5
8.0
7.5
7.0
6.5
6.0
5.5
5.0
4.5
4.0
3.5
3.0
2.5
2.0
1.5
1.0
0.5
0.0 ppm
5.13
1.01
1.08
1.00
2.22
0.98
1.11
1.08
1.11
2.25
1.15
3.20

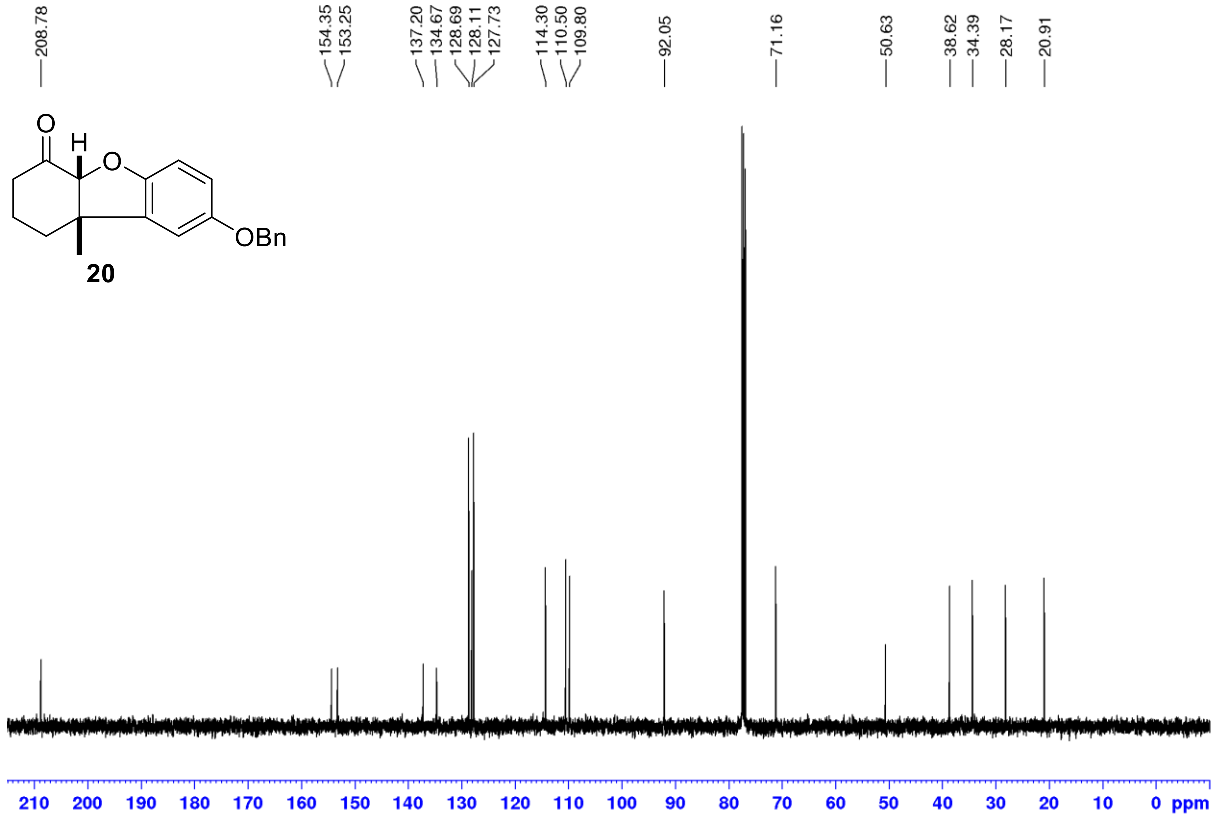

208.78
154.35
153.25
137.20
134.67
128.69
128.11
127.73
114.30
110.50
109.80
92.05
71.16
50.63
38.62
34.39
28.17
20.91
O
H
O
OBn
20
210
200
190
180
170
160
150
140
130
120
110
100
90
80
70
60
50
40
30
20
10
0 ppm

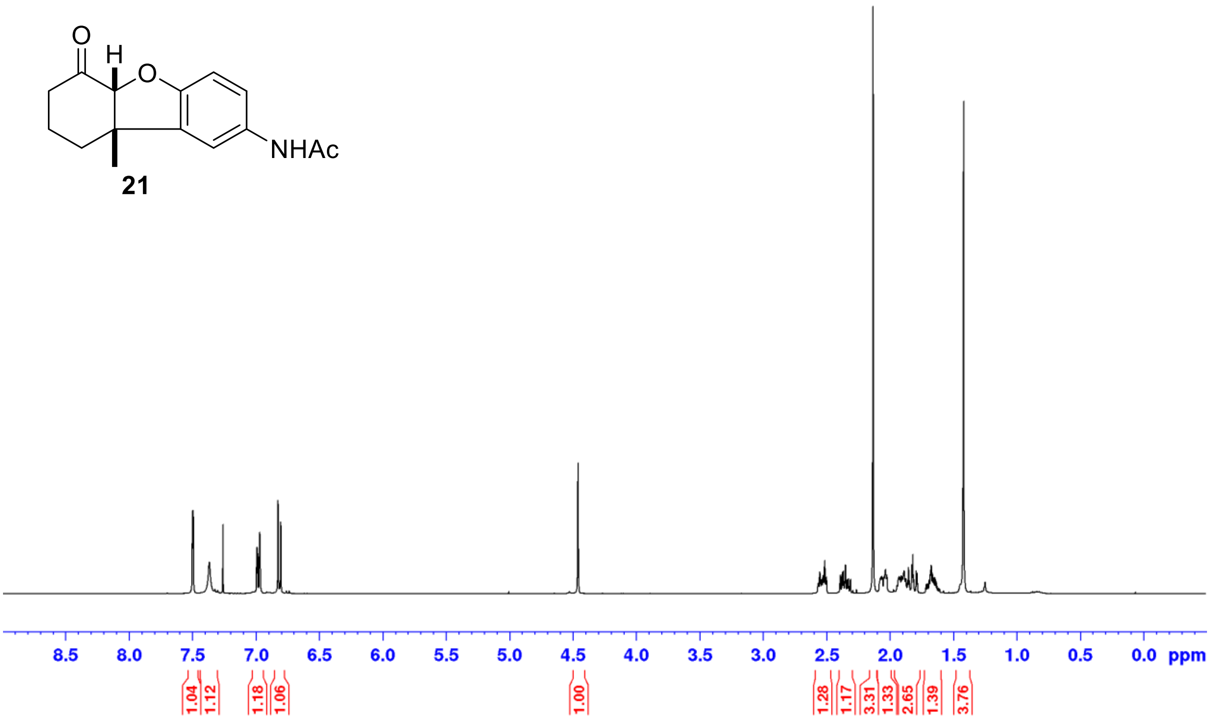
O
H
O
NHAc
21
8.5
8.0
7.5
7.0
6.5
6.0
5.5
5.0
4.5
4.0
3.5
3.0
2.5
2.0
1.5
1.0
0.5
0.0 ppm
1.04
1.12
1.18
1.06
1.00
1.28
1.17
3.31
1.33
2.65
1.39
3.76

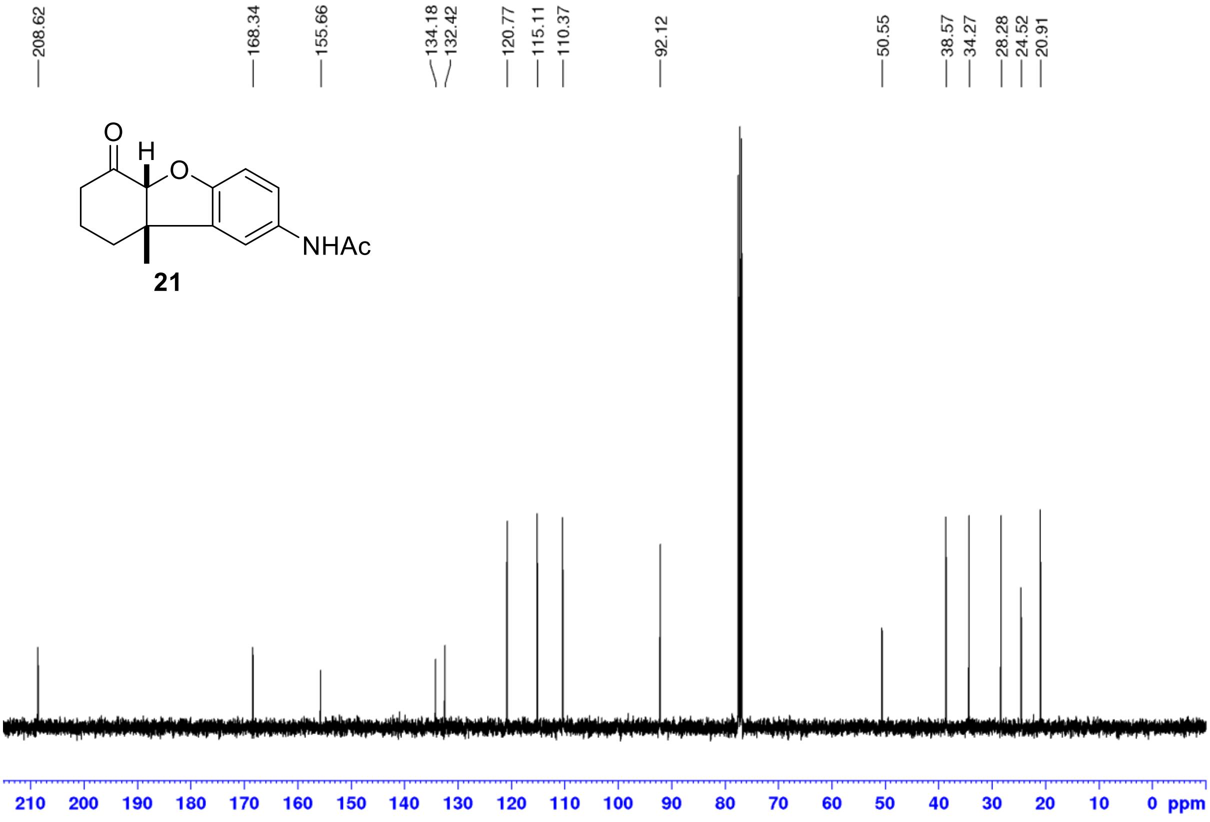
208.62
168.34
155.66
134.18
132.42
120.77
115.11
110.37
92.12
50.55
38.57
34.27
28.28
24.52
20.91
O
H
O
NHAc
21
210
200
190
180
170
160
150
140
130
120
110
100
90
80
70
60
50
40
30
20
10
0 ppm

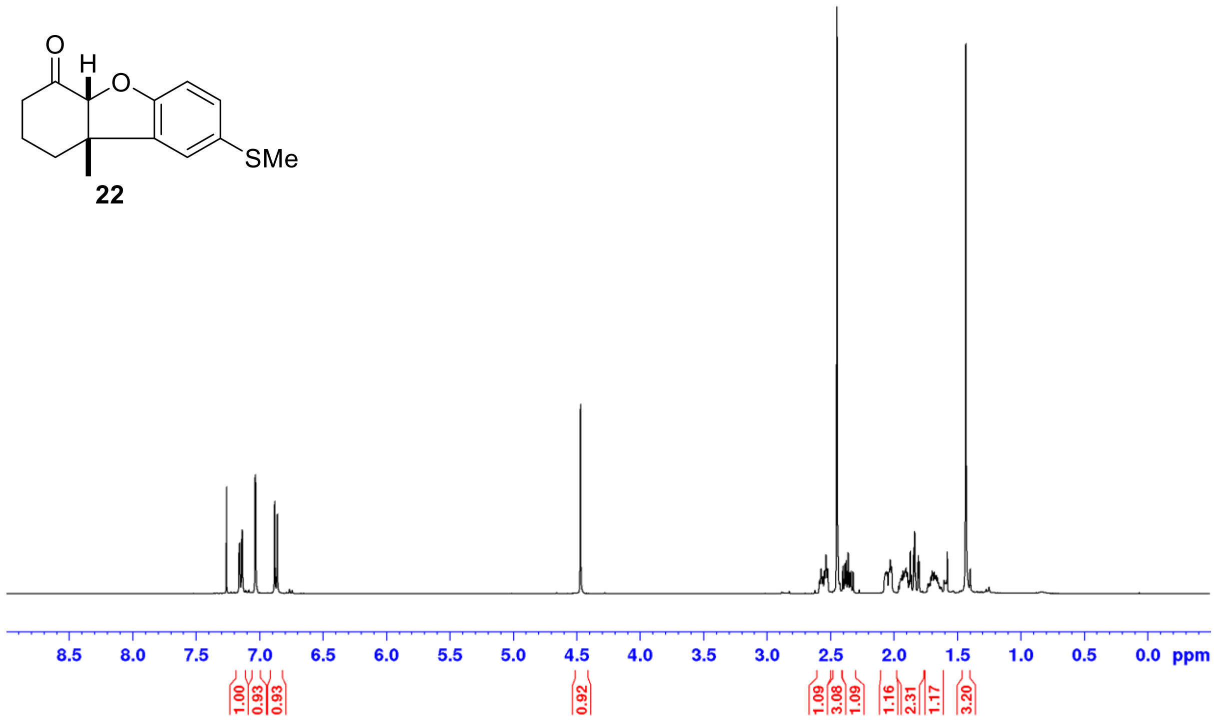
O
H
O
SMe
22
8.5
8.0
7.5
7.0
6.5
6.0
5.5
5.0
4.5
4.0
3.5
3.0
2.5
2.0
1.5
1.0
0.5
0.0
ppm
1.00
0.93
0.93
0.92
1.09
3.08
1.09
1.16
2.31
1.17
3.20

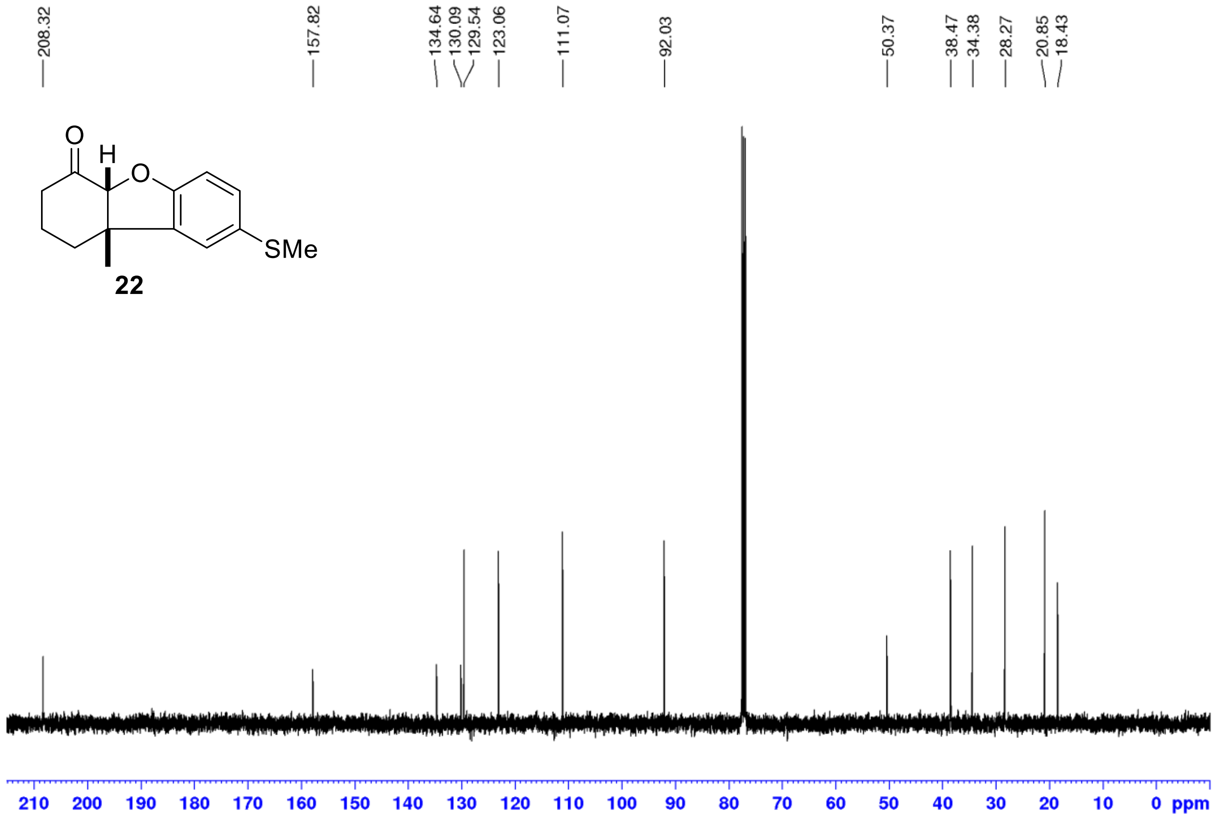
208.32
157.82
134.64
130.09
129.54
123.06
111.07
92.03
50.37
38.47
34.38
28.27
20.85
18.43
O
H
O
SMe
22
210
200
190
180
170
160
150
140
130
120
110
100
90
80
70
60
50
40
30
20
10
0
ppm

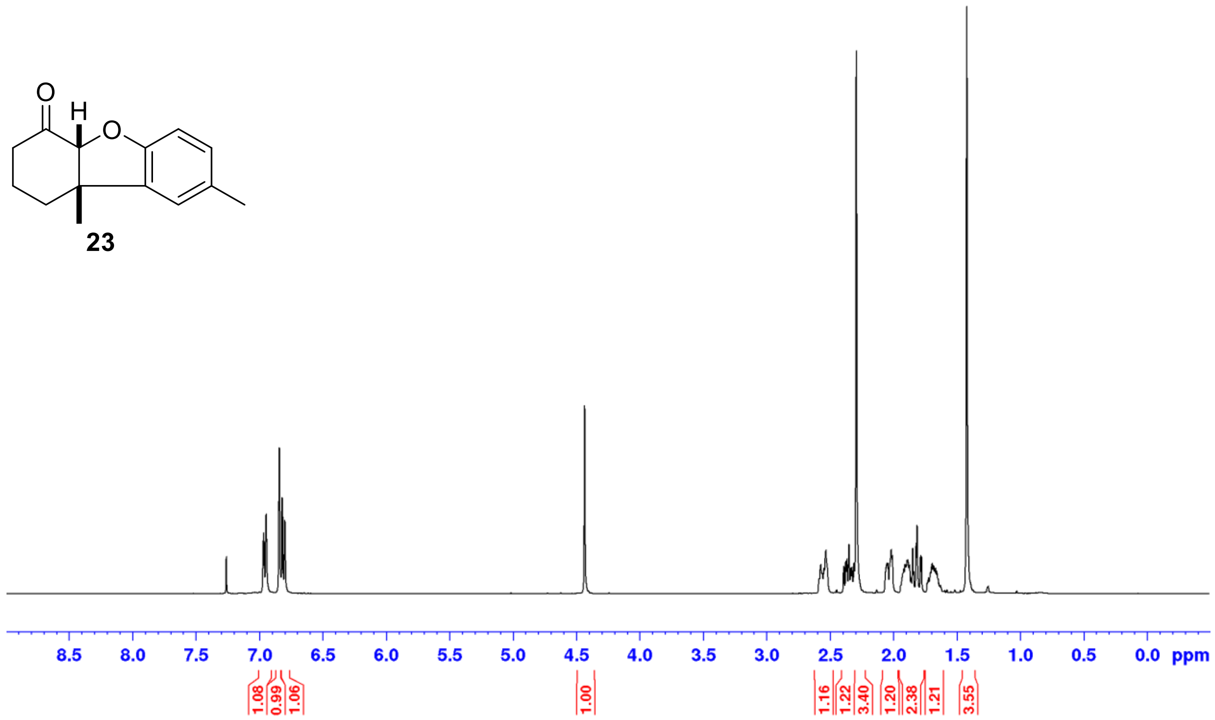
O
H
O
23
8.5 8.0 7.5 7.0 6.5 6.0 5.5 5.0 4.5 4.0 3.5 3.0 2.5 2.0 1.5 1.0 0.5 0.0 ppm
1.08 0.99 1.06
1.00
1.16 1.22 3.40 1.20 2.38 1.21 3.55

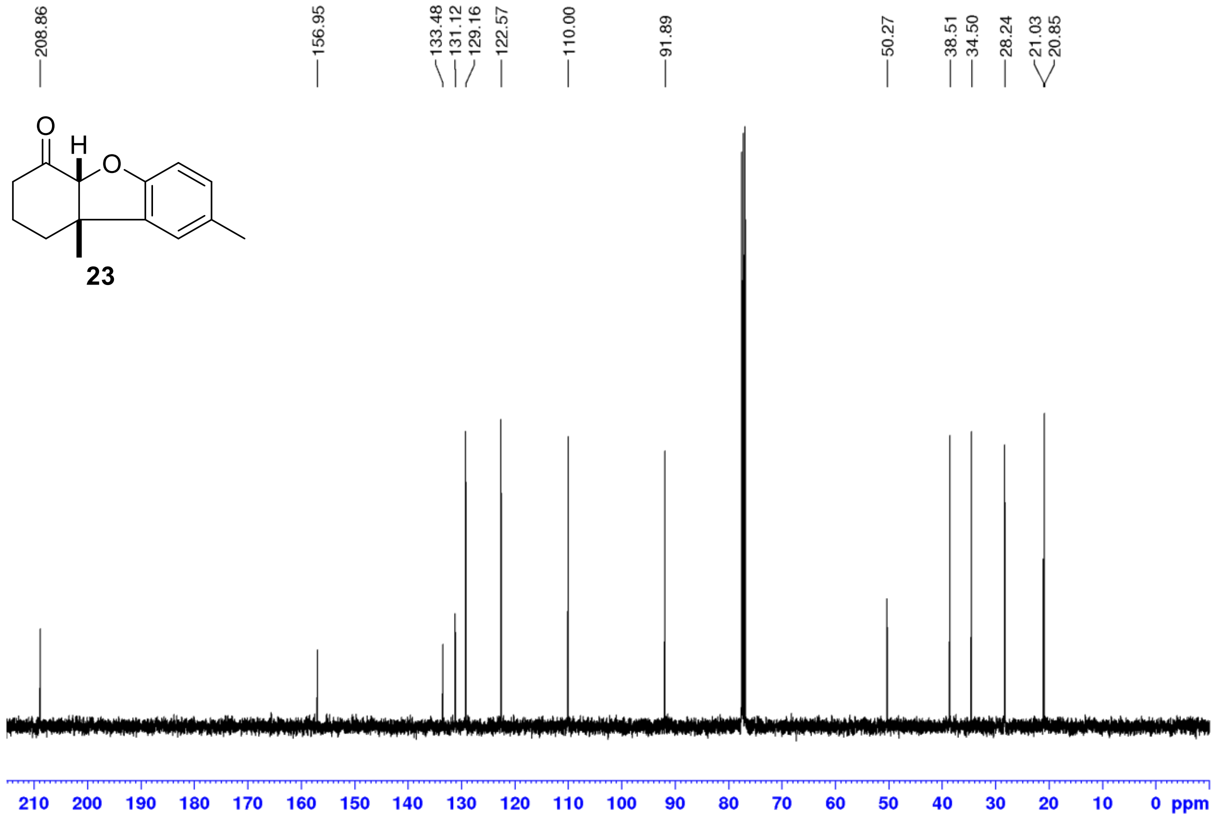
208.86
156.95
133.48 131.12 129.16 122.57
110.00
91.89
50.27
38.51 34.50
28.24
21.03 20.85
O
H
O
23
210 200 190 180 170 160 150 140 130 120 110 100 90 80 70 60 50 40 30 20 10 0 ppm

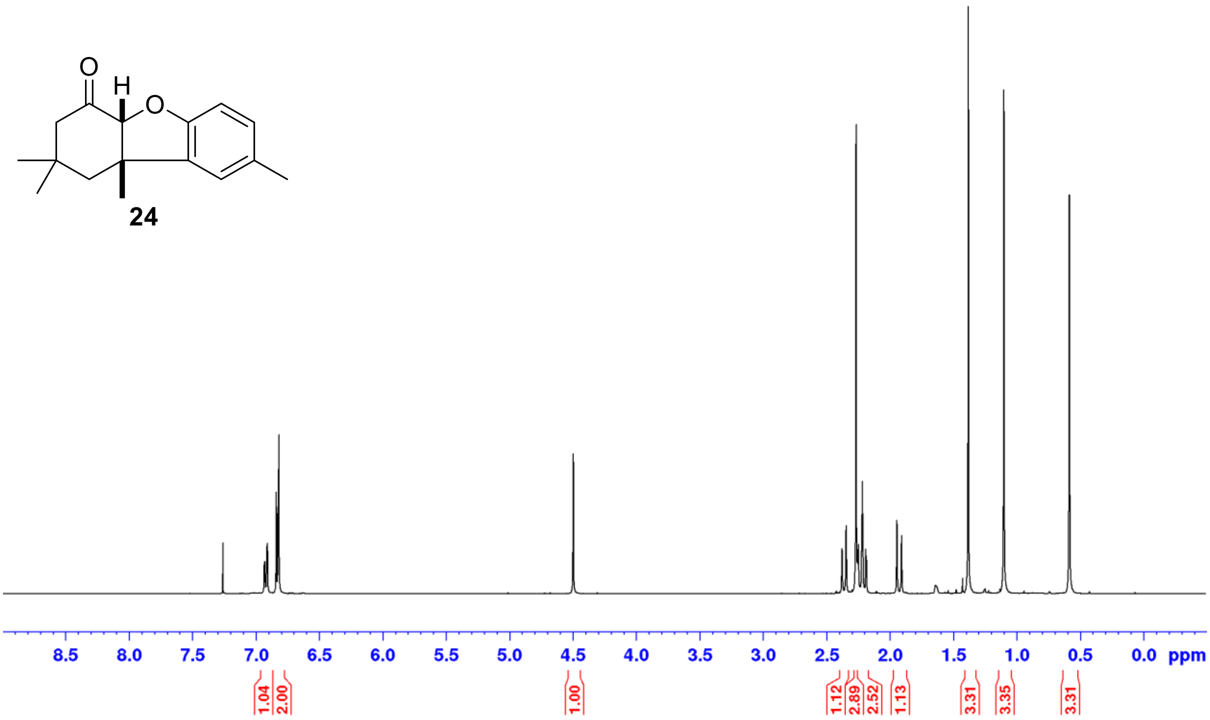
24
8.5 8.0 7.5 7.0 6.5 6.0 5.5 5.0 4.5 4.0 3.5 3.0 2.5 2.0 1.5 1.0 0.5 0.0 ppm
1.04 2.00 1.00 1.12 2.89 2.52 1.13 3.31 3.35 3.31

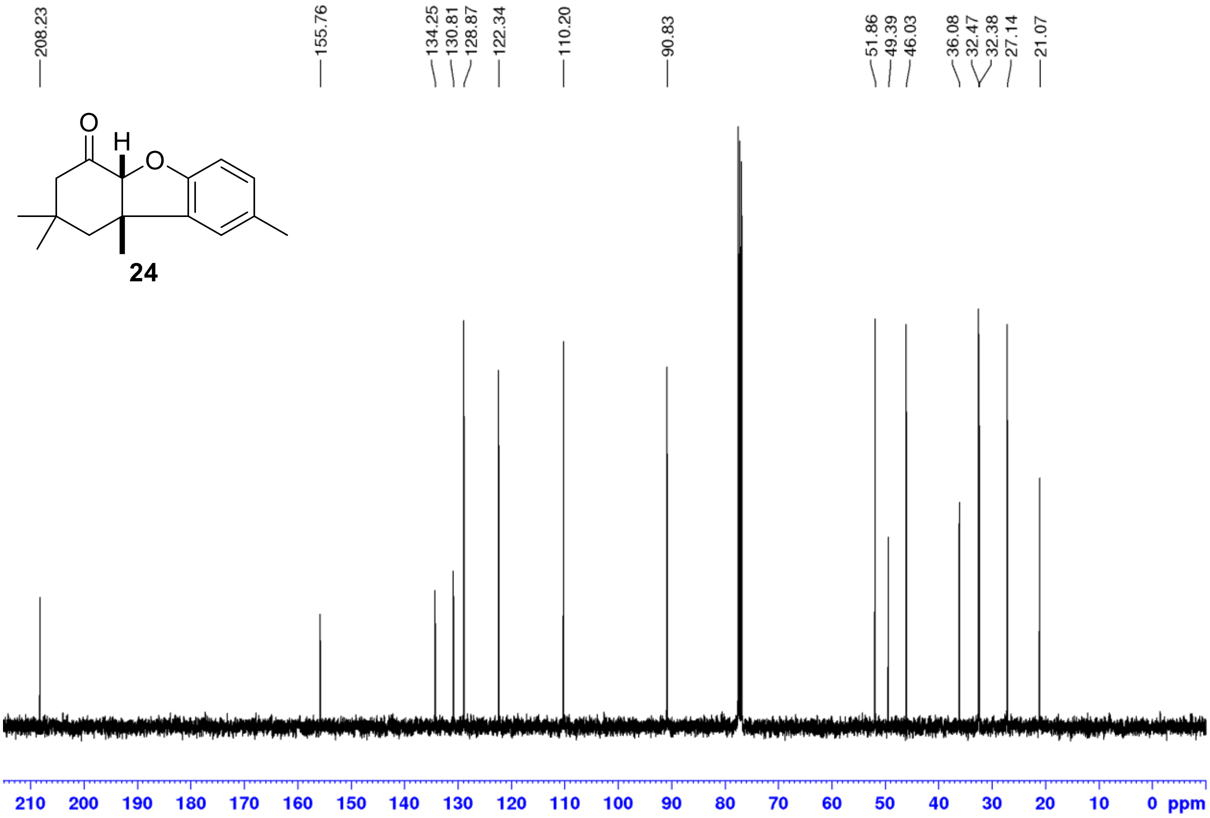
208.23
155.76
134.25 130.81 128.87 122.34
110.20
90.83
51.86 49.39 46.03
36.08 32.47 32.38 27.14
21.07
24
210 200 190 180 170 160 150 140 130 120 110 100 90 80 70 60 50 40 30 20 10 0 ppm

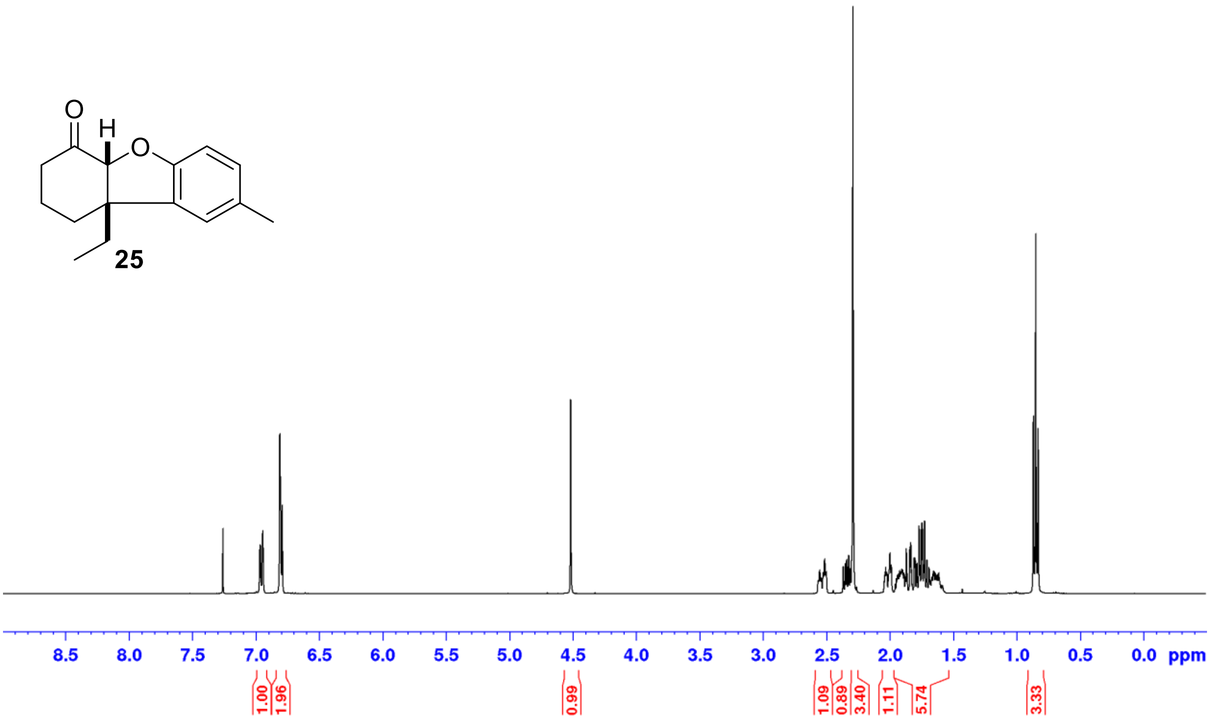
25
8.5 8.0 7.5 7.0 6.5 6.0 5.5 5.0 4.5 4.0 3.5 3.0 2.5 2.0 1.5 1.0 0.5 0.0 ppm
1.00 1.96 0.99 1.09 0.89 3.40 1.11 5.74 3.33

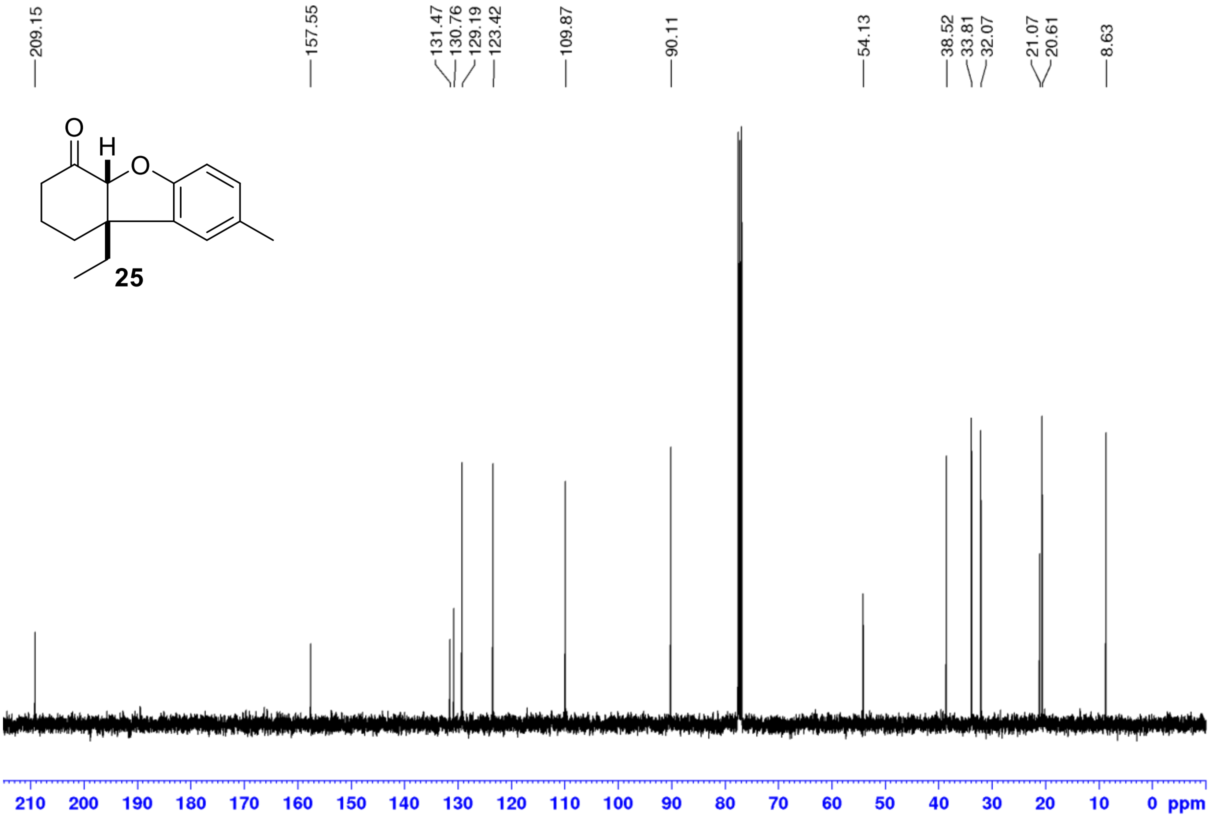
209.15
157.55
131.47 130.76 129.19 123.42
109.87
90.11
54.13
38.52 33.81 32.07
21.07 20.61
8.63
25
210 200 190 180 170 160 150 140 130 120 110 100 90 80 70 60 50 40 30 20 10 0 ppm

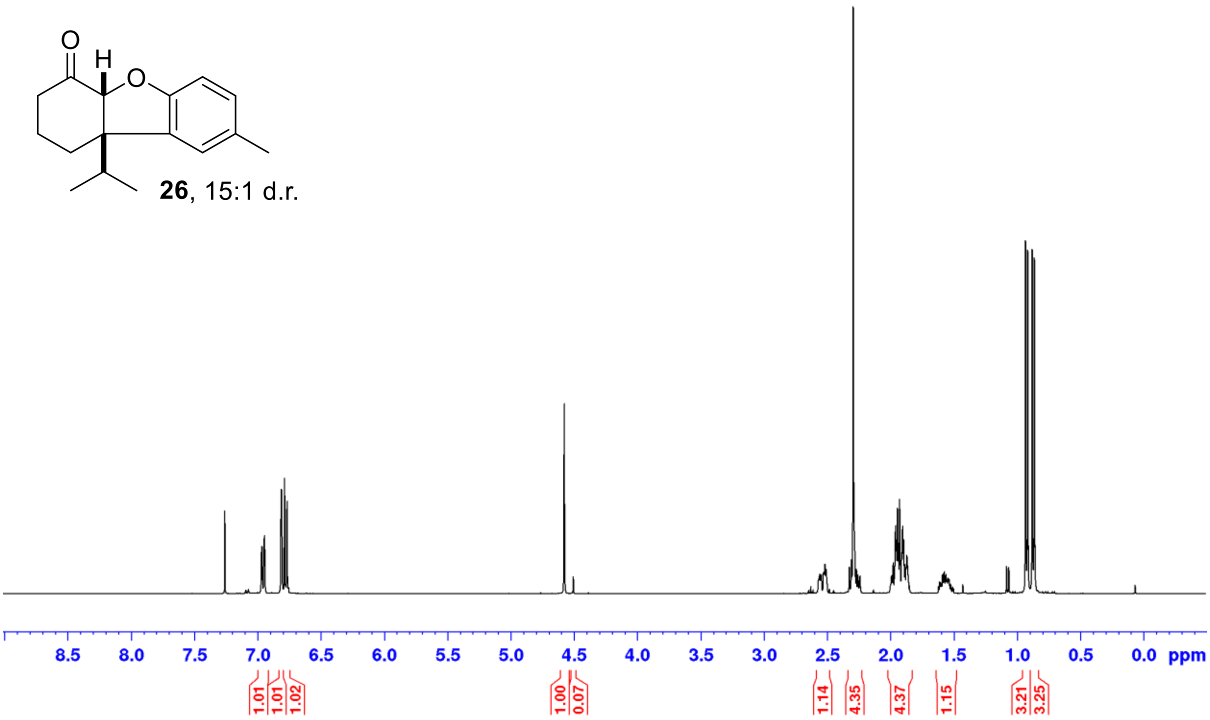
O
H
O
26, 15:1 d.r.
8.5
8.0
7.5
7.0
6.5
6.0
5.5
5.0
4.5
4.0
3.5
3.0
2.5
2.0
1.5
1.0
0.5
0.0
ppm
1.01
1.01
1.02
1.00
0.07
1.14
4.35
4.37
1.15
3.21
3.25

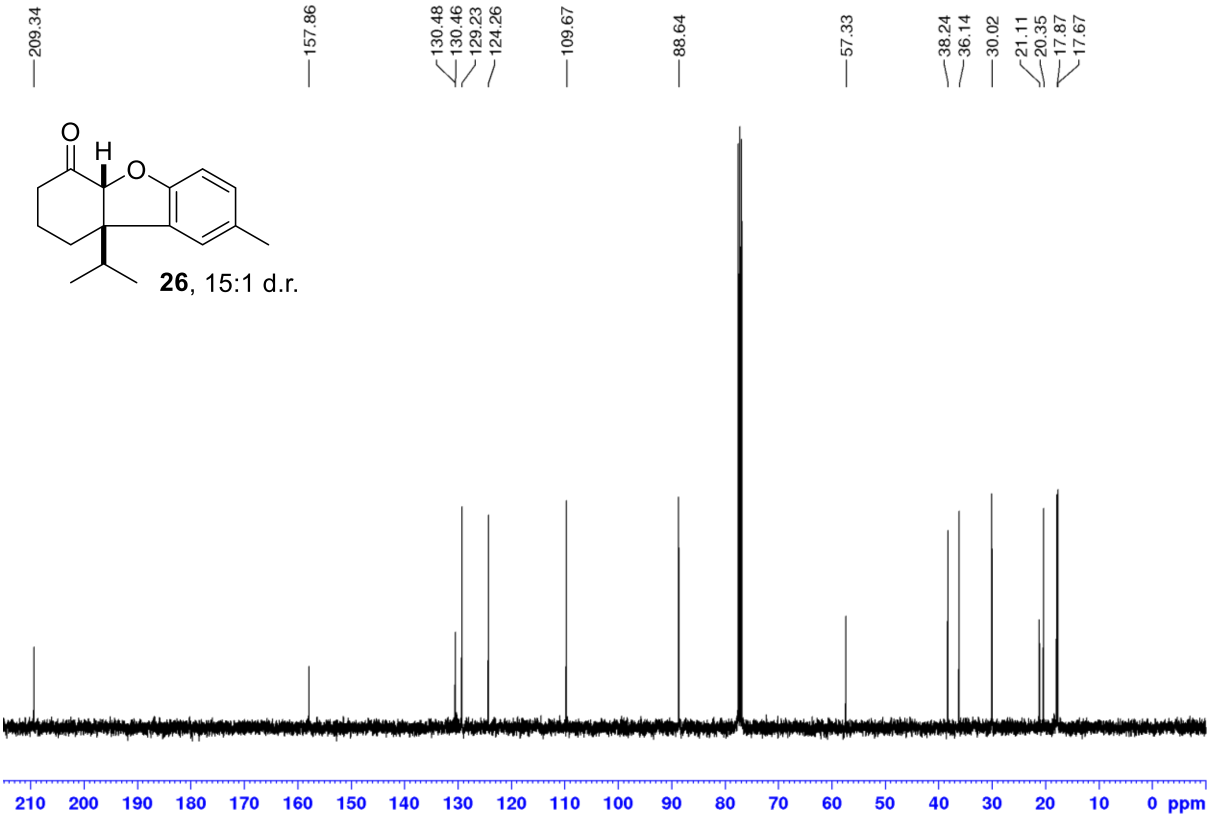
209.34
157.86
130.48
130.46
129.23
124.26
109.67
88.64
57.33
38.24
36.14
30.02
21.11
20.35
17.87
17.67
O
H
O
26, 15:1 d.r.
210
200
190
180
170
160
150
140
130
120
110
100
90
80
70
60
50
40
30
20
10
0
ppm

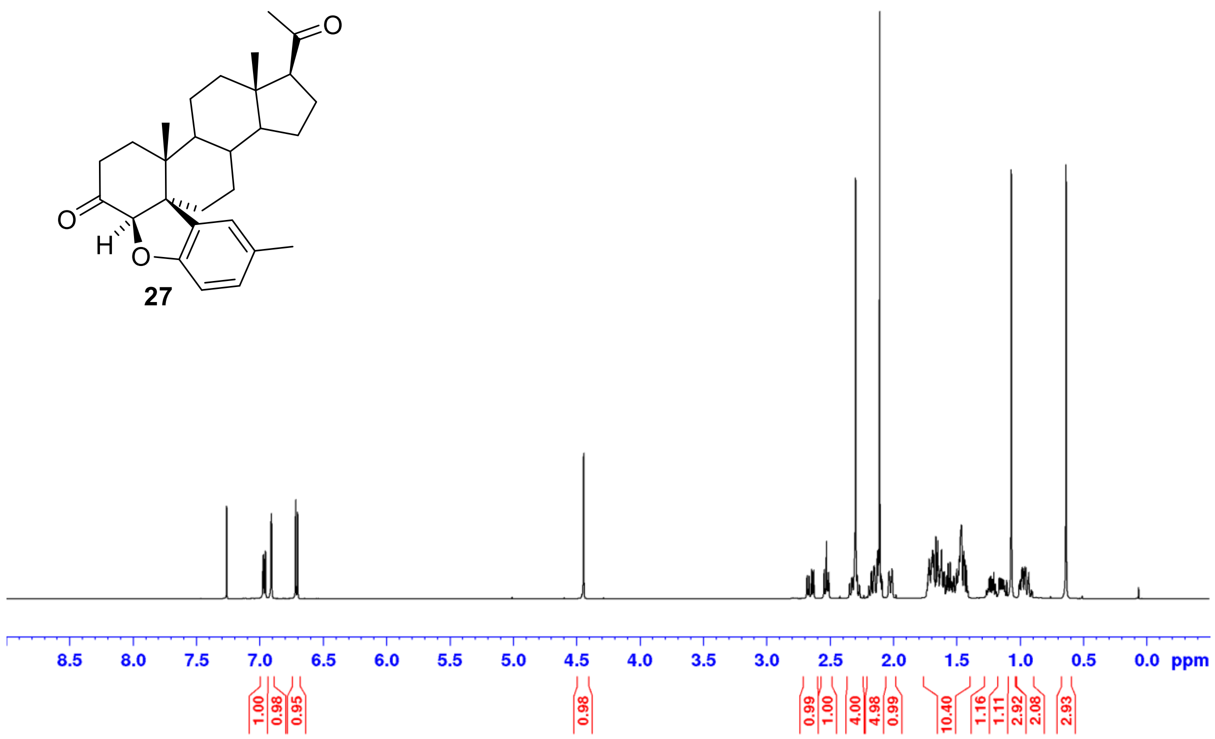

27
8.5
8.0
7.5
7.0
6.5
6.0
5.5
5.0
4.5
4.0
3.5
3.0
2.5
2.0
1.5
1.0
0.5
0.0
ppm
1.00
0.98
0.95
0.98
0.99
1.00
4.00
4.98
0.99
10.40
1.16
1.11
2.92
2.08
2.93

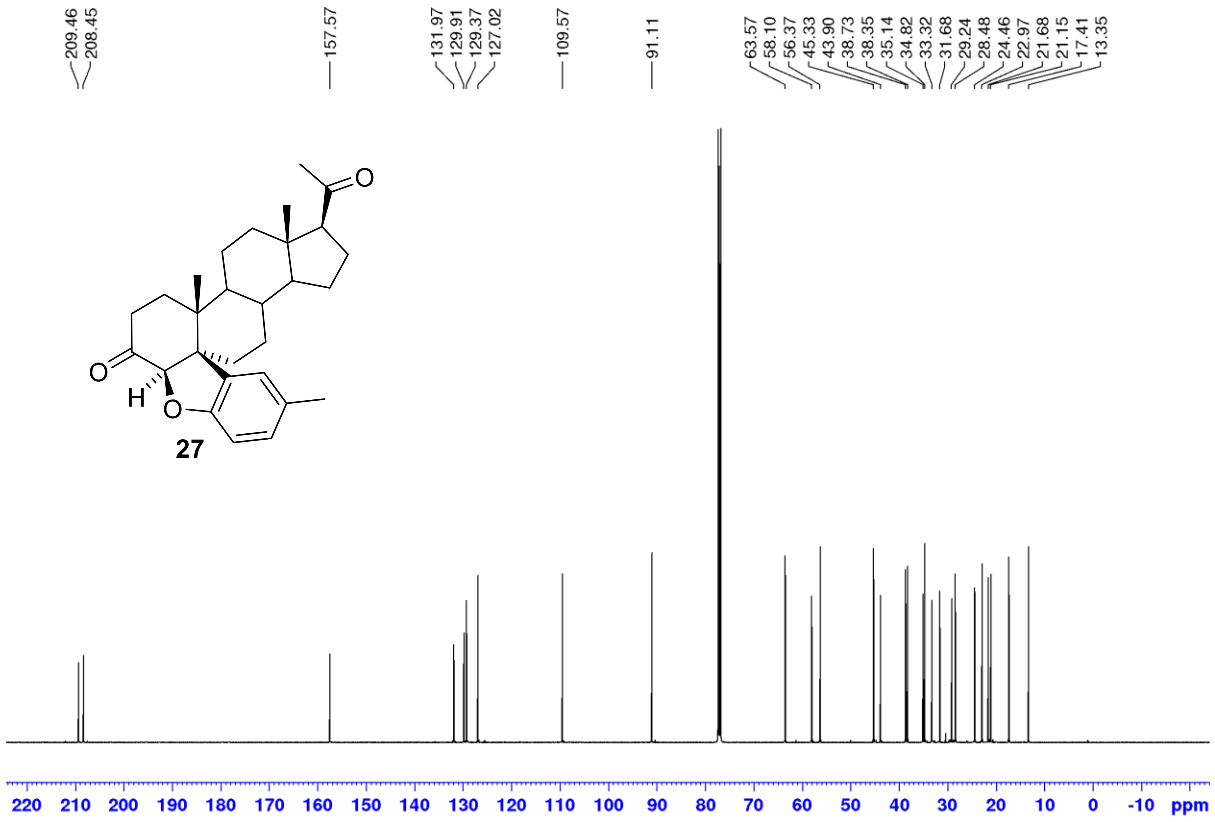

209.46
208.45
157.57
131.97
129.91
129.37
127.02
109.57
91.11
63.57
58.10
56.37
45.33
43.90
38.73
38.35
35.14
34.82
33.32
31.68
29.24
28.48
24.46
22.97
21.68
21.15
17.41
13.35
27
220
210
200
190
180
170
160
150
140
130
120
110
100
90
80
70
60
50
40
30
20
10
0
-10
ppm

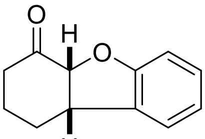

28

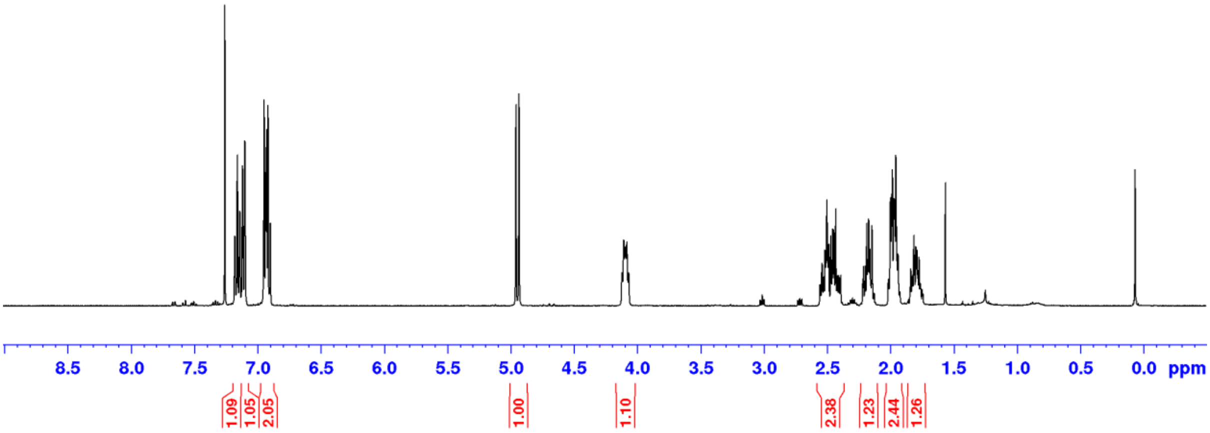

8.5
8.0
7.5
7.0
6.5
6.0
5.5
5.0
4.5
4.0
3.5
3.0
2.5
2.0
1.5
1.0
0.5
0.0 ppm
1.09
1.05
2.05
1.00
1.10
2.38
1.23
2.44
1.26

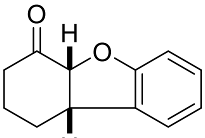

28

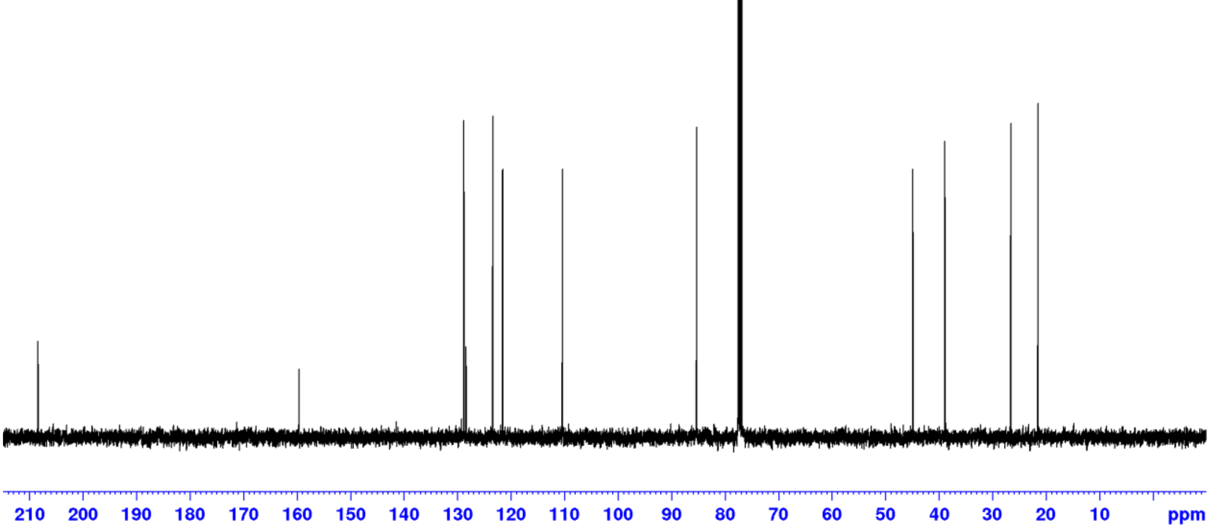

208.44
159.64
128.80
128.44
123.41
121.55
110.36
85.27
44.85
38.85
26.55
21.42
210
200
190
180
170
160
150
140
130
120
110
100
90
80
70
60
50
40
30
20
10
ppm

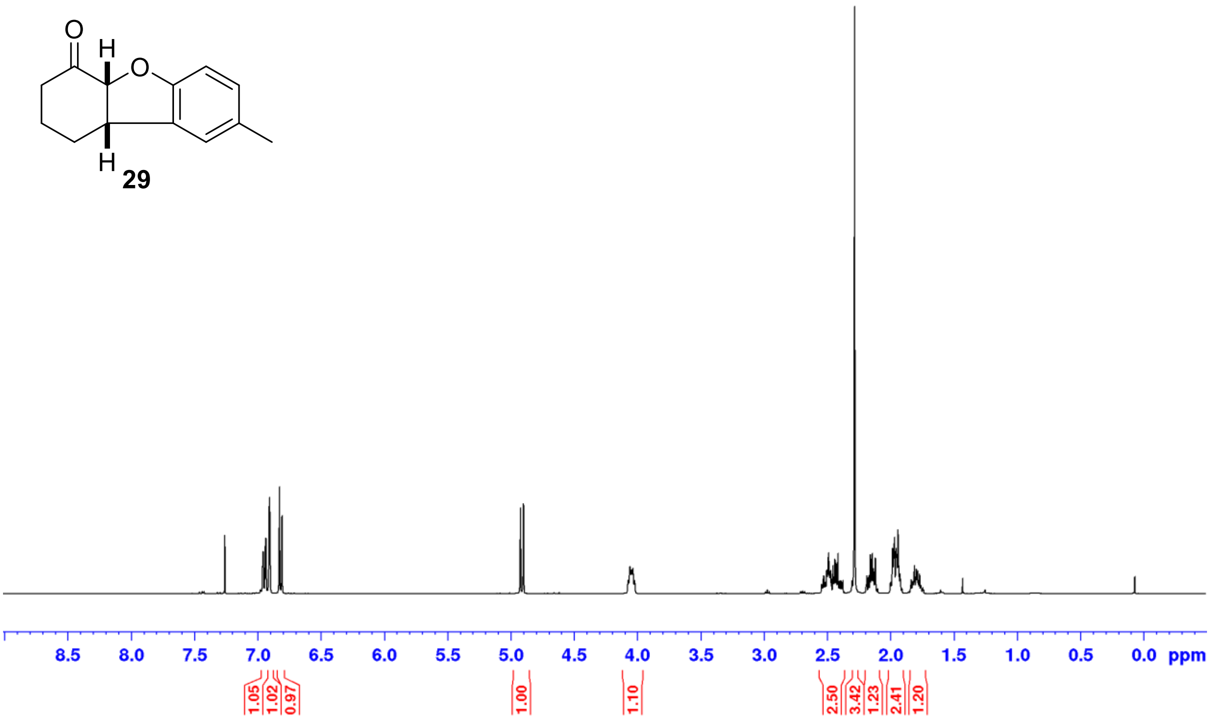
O
H
O
H
29
8.5
8.0
7.5
7.0
6.5
6.0
5.5
5.0
4.5
4.0
3.5
3.0
2.5
2.0
1.5
1.0
0.5
0.0 ppm
1.05
1.02
0.97
1.00
1.10
2.50
3.42
1.23
2.41
1.20

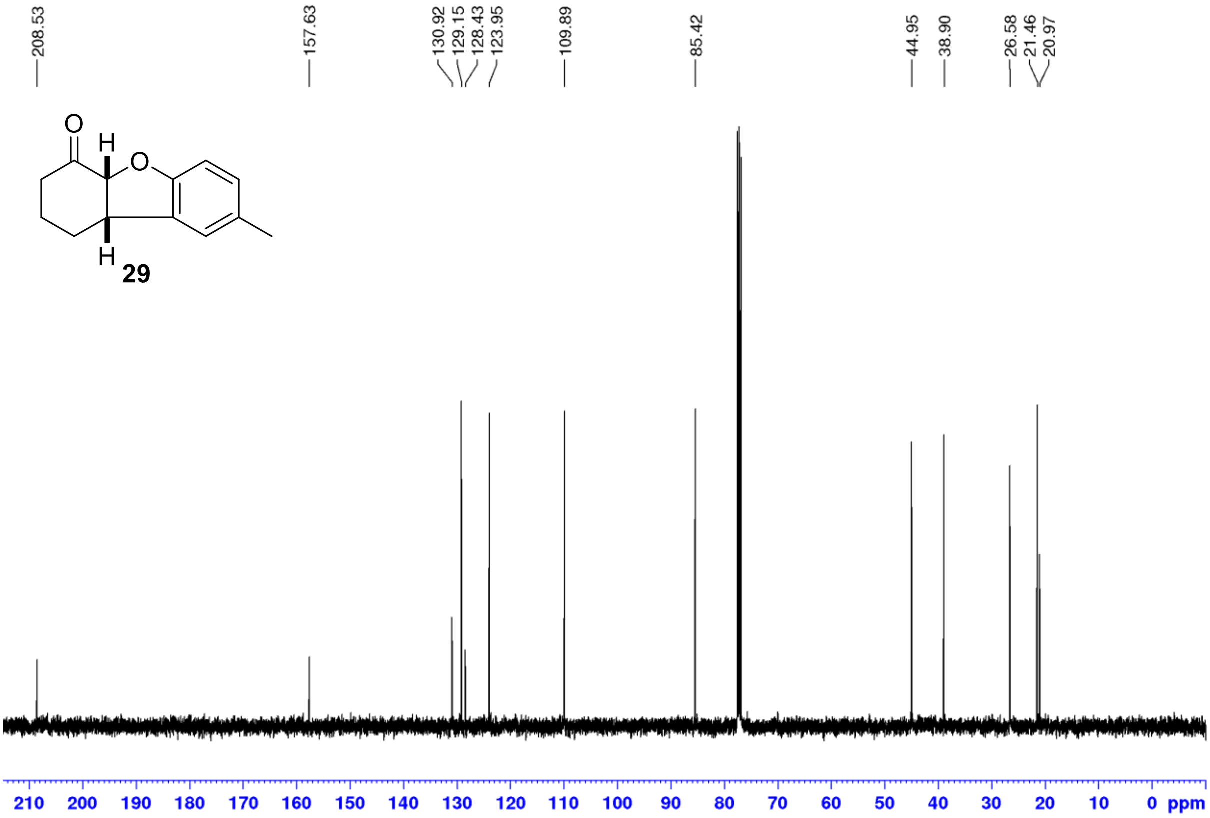
208.53
157.63
130.92
129.15
128.43
123.95
109.89
85.42
44.95
38.90
26.58
21.46
20.97
O
H
O
H
29
210
200
190
180
170
160
150
140
130
120
110
100
90
80
70
60
50
40
30
20
10
0 ppm

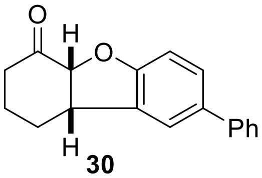
O
H
O
H
Ph

30

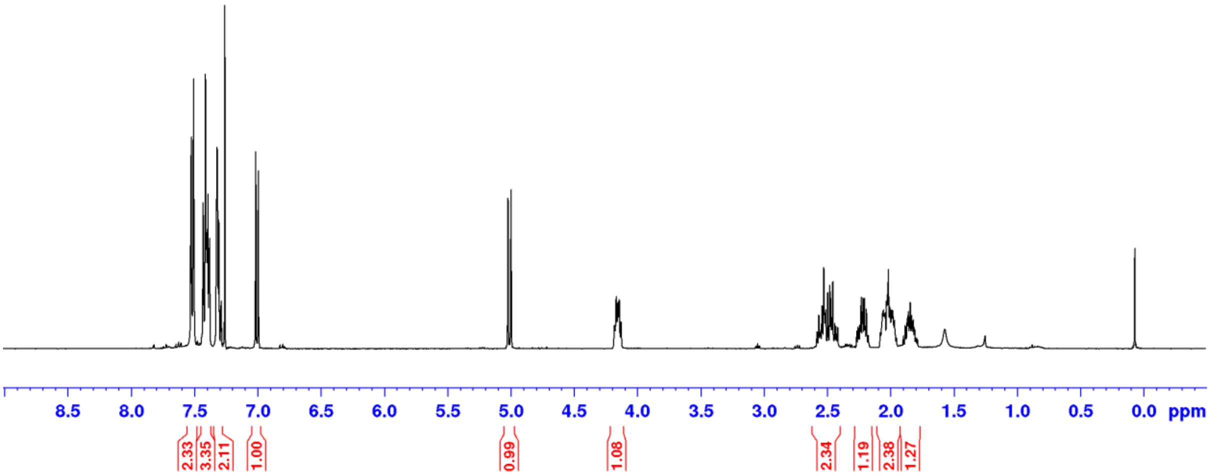
8.5
8.0
7.5
7.0
6.5
6.0
5.5
5.0
4.5
4.0
3.5
3.0
2.5
2.0
1.5
1.0
0.5
0.0 ppm
2.33
3.35
2.11
1.00
0.99
1.08
2.34
1.19
2.38
1.27

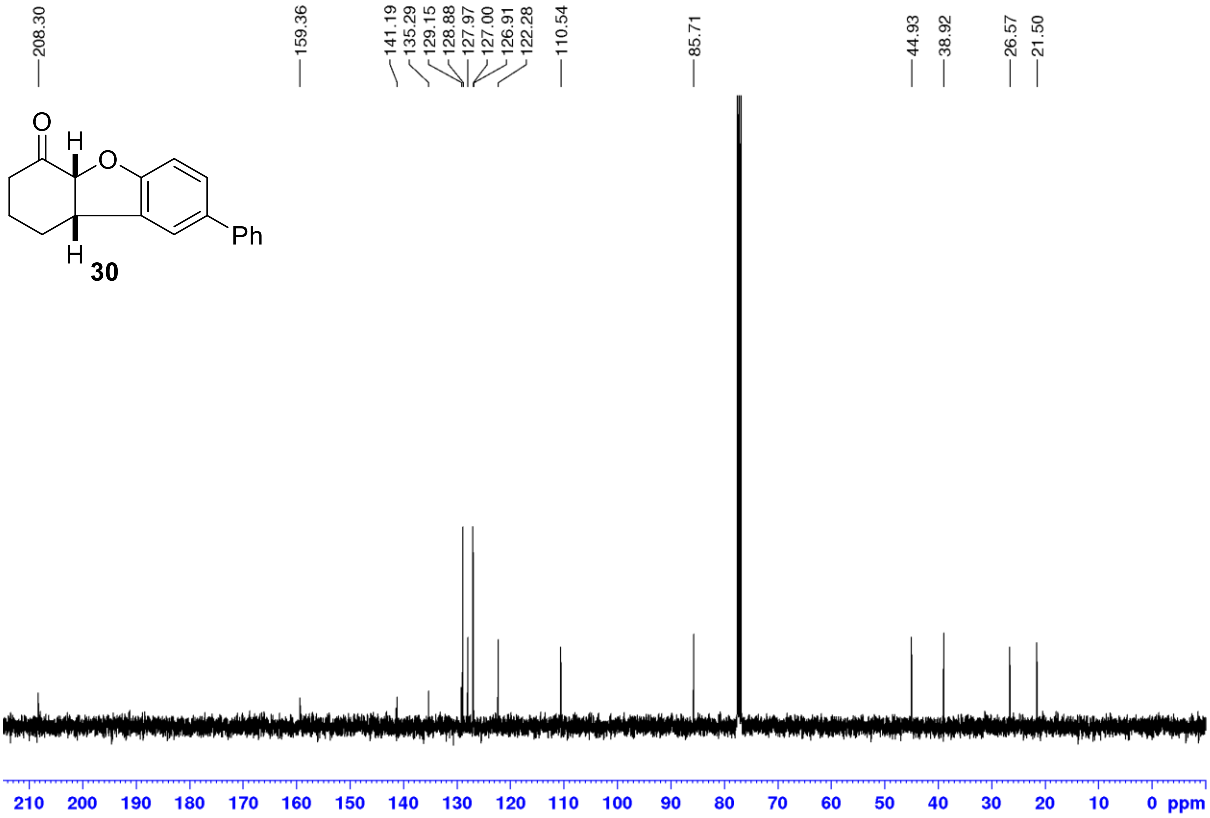
208.30
159.36
141.19
135.29
129.15
128.88
127.97
127.00
126.91
122.28
110.54
85.71
44.93
38.92
26.57
21.50
210
200
190
180
170
160
150
140
130
120
110
100
90
80
70
60
50
40
30
20
10
0 ppm
O
H
O
H
Ph

30

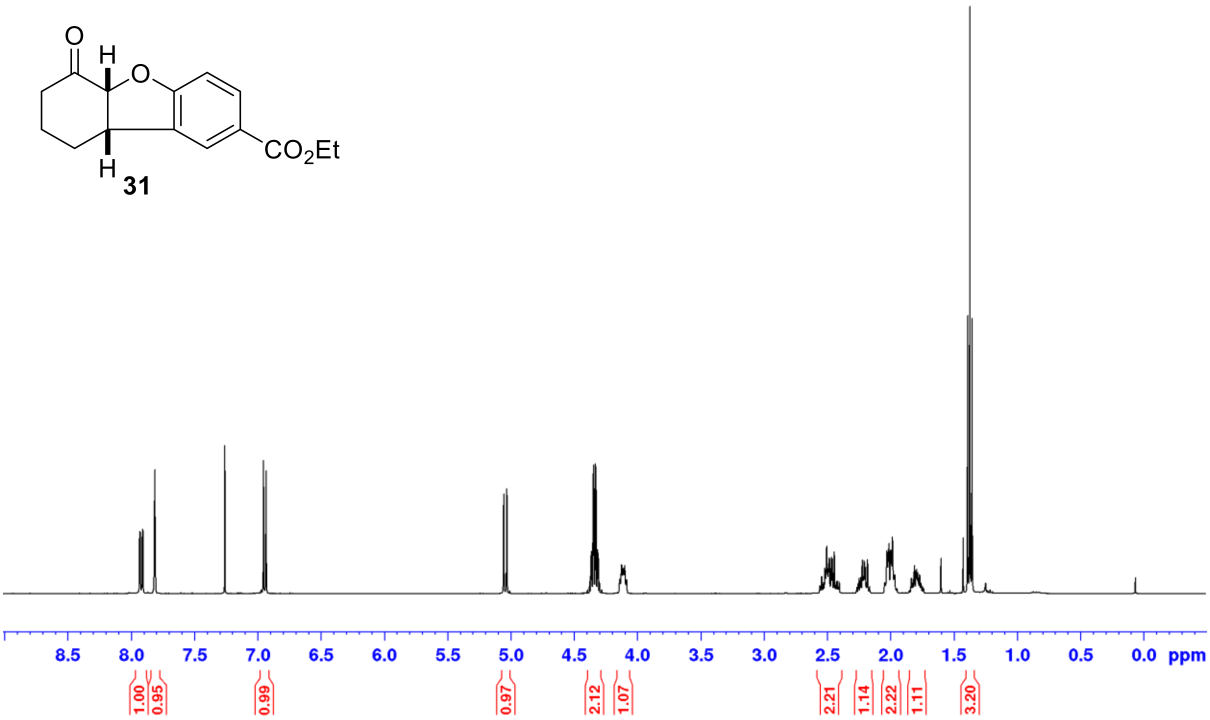

O
H
O
CO2Et
H
31
8.5 8.0 7.5 7.0 6.5 6.0 5.5 5.0 4.5 4.0 3.5 3.0 2.5 2.0 1.5 1.0 0.5 0.0 ppm
1.00 0.95 0.99 0.97 2.12 1.07 2.21 1.14 2.22 1.11 3.20

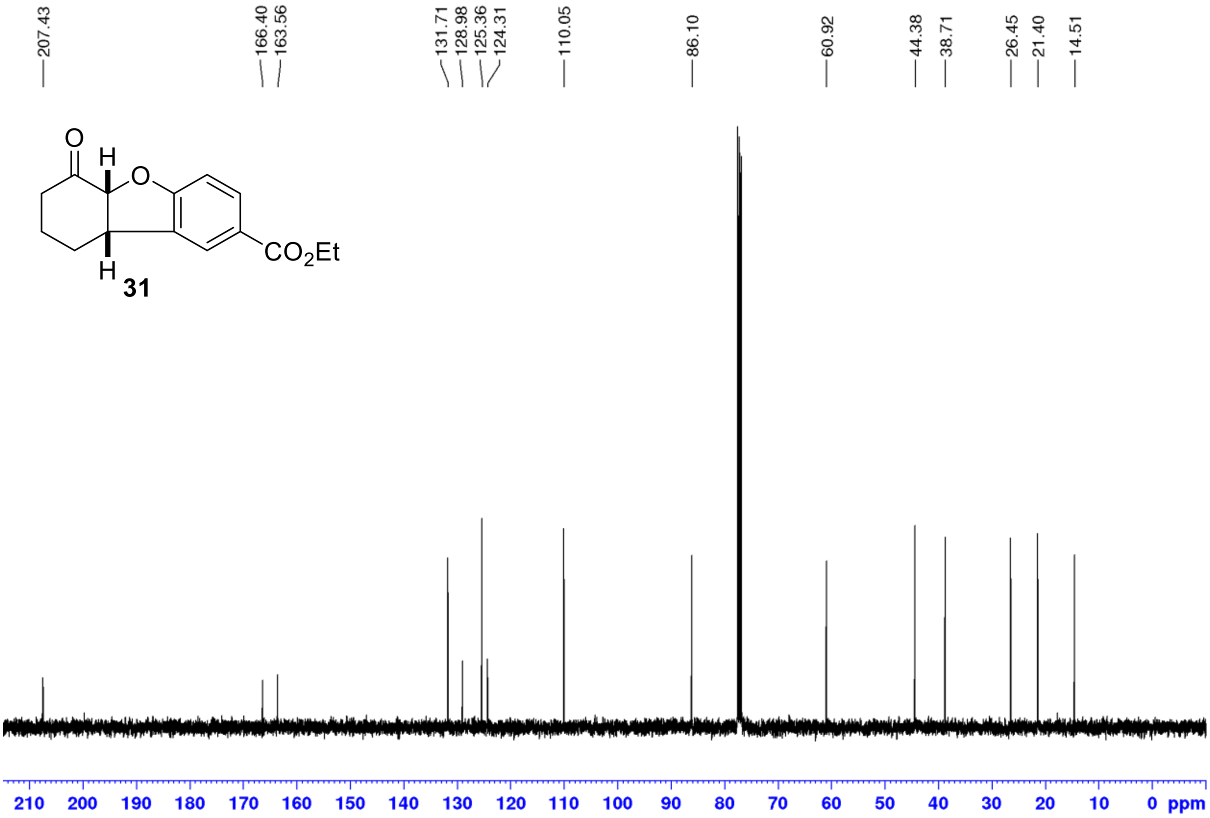

207.43
166.40
163.56
131.71
128.98
125.36
124.31
110.05
86.10
60.92
44.38
38.71
26.45
21.40
14.51
O
H
O
CO2Et
H
31
210 200 190 180 170 160 150 140 130 120 110 100 90 80 70 60 50 40 30 20 10 0 ppm

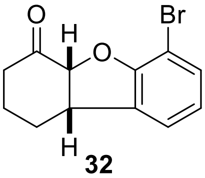

32

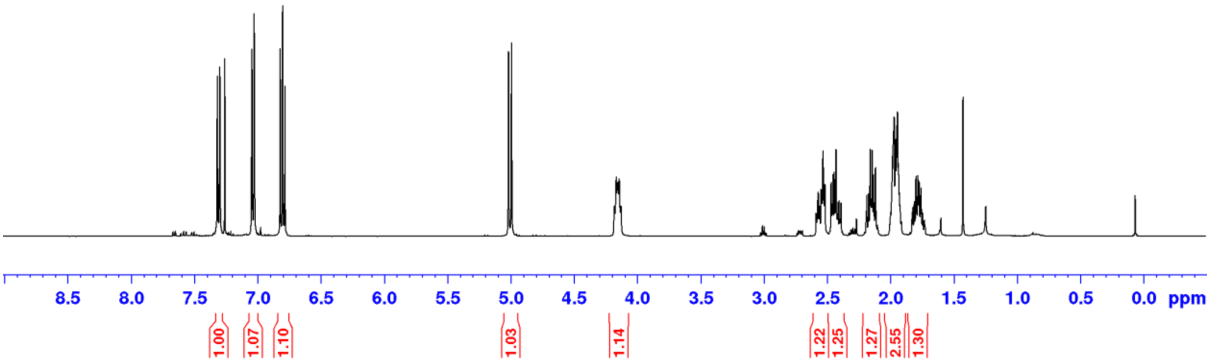
8.5 8.0 7.5 7.0 6.5 6.0 5.5 5.0 4.5 4.0 3.5 3.0 2.5 2.0 1.5 1.0 0.5 0.0 ppm
1.00 1.07 1.10 1.03 1.14 1.22 1.25 1.27 2.55 1.30

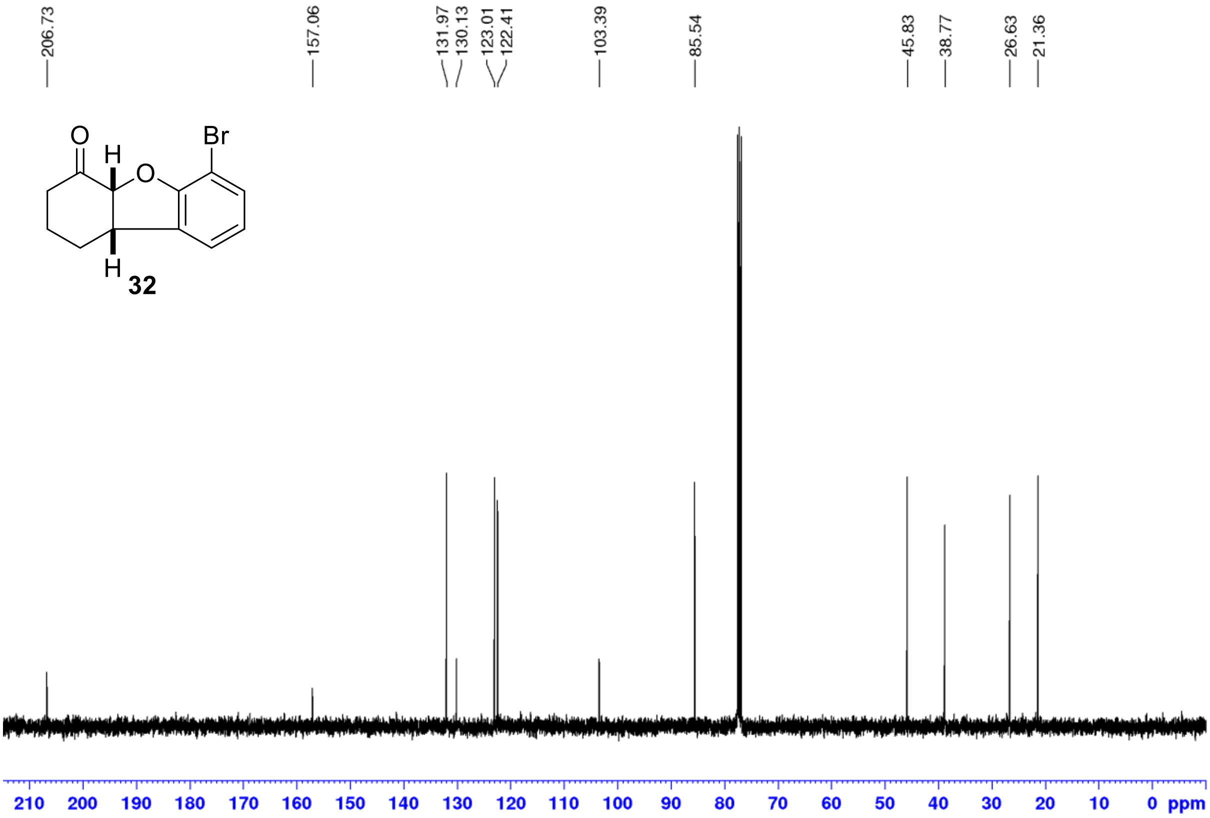

32
206.73 157.06 131.97 130.13 123.01 122.41 103.39 85.54 45.83 38.77 26.63 21.36
210 200 190 180 170 160 150 140 130 120 110 100 90 80 70 60 50 40 30 20 10 0 ppm

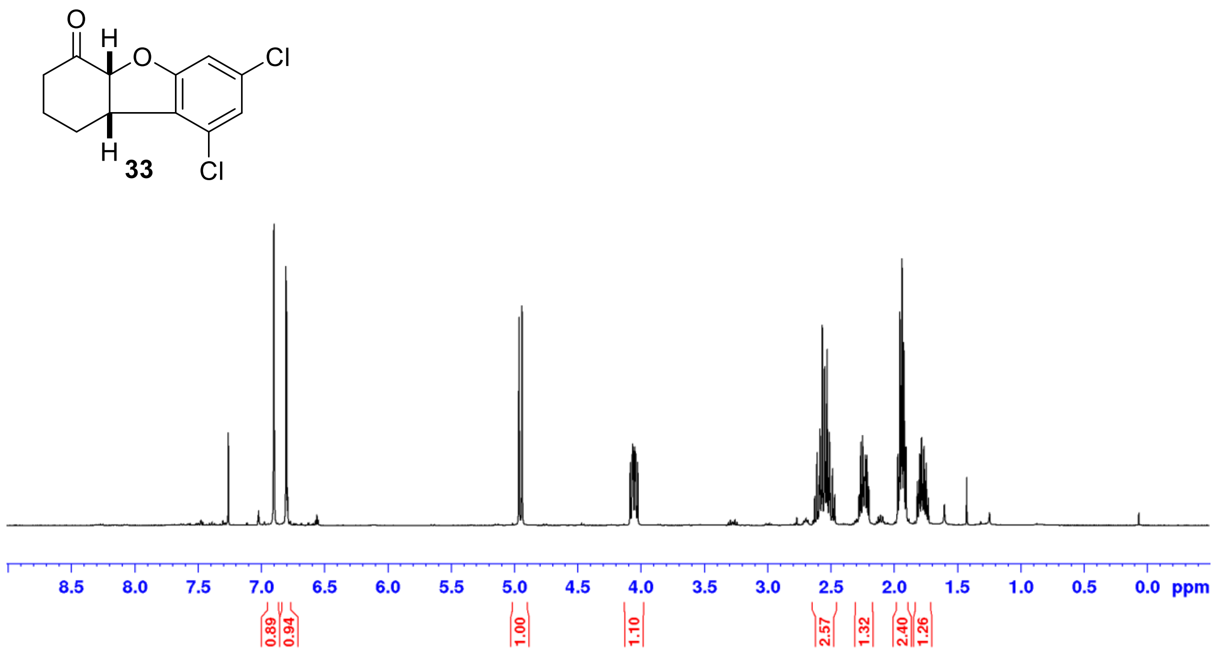

O
H
O
Cl
H
33
Cl
8.5
8.0
7.5
7.0
6.5
6.0
5.5
5.0
4.5
4.0
3.5
3.0
2.5
2.0
1.5
1.0
0.5
0.0 ppm
0.89
0.94
1.00
1.10
2.57
1.32
2.40
1.26

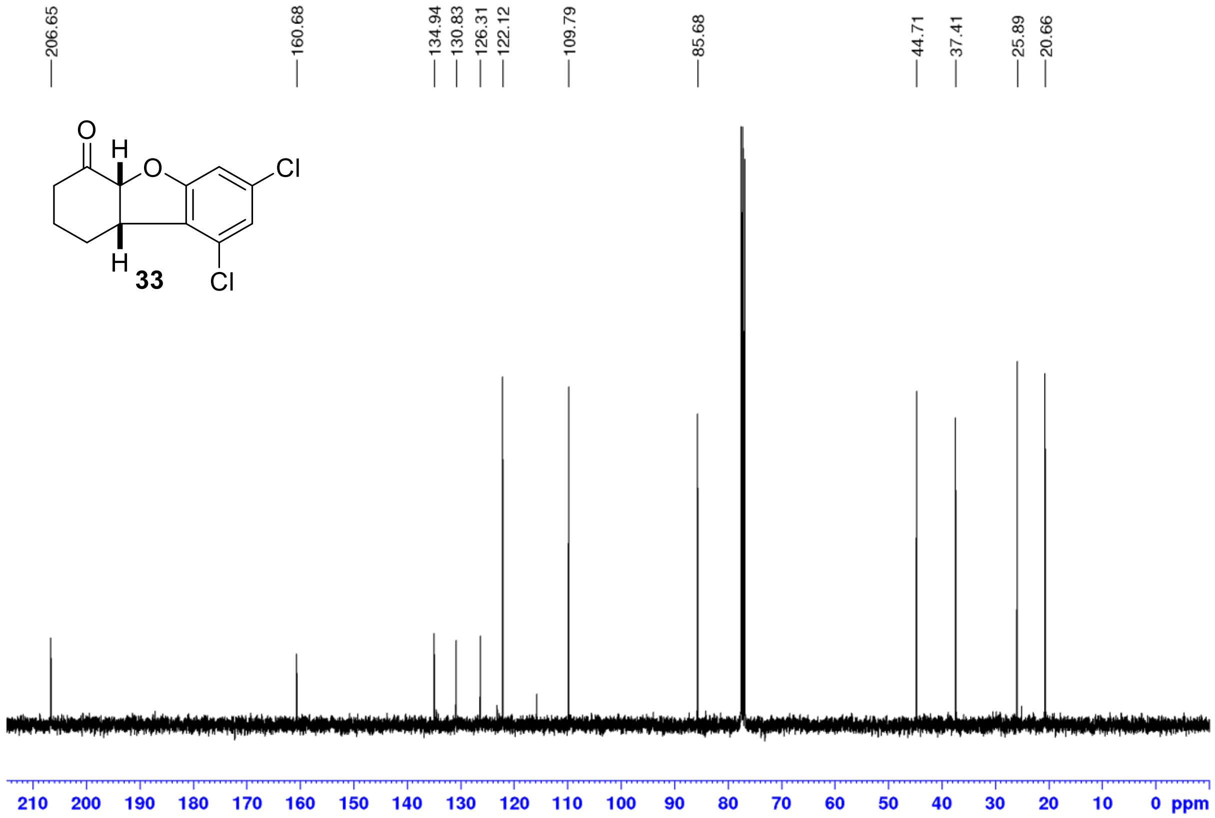

206.65
160.68
134.94
130.83
126.31
122.12
109.79
85.68
44.71
37.41
25.89
20.66
O
H
O
Cl
H
33
Cl
210
200
190
180
170
160
150
140
130
120
110
100
90
80
70
60
50
40
30
20
10
0 ppm

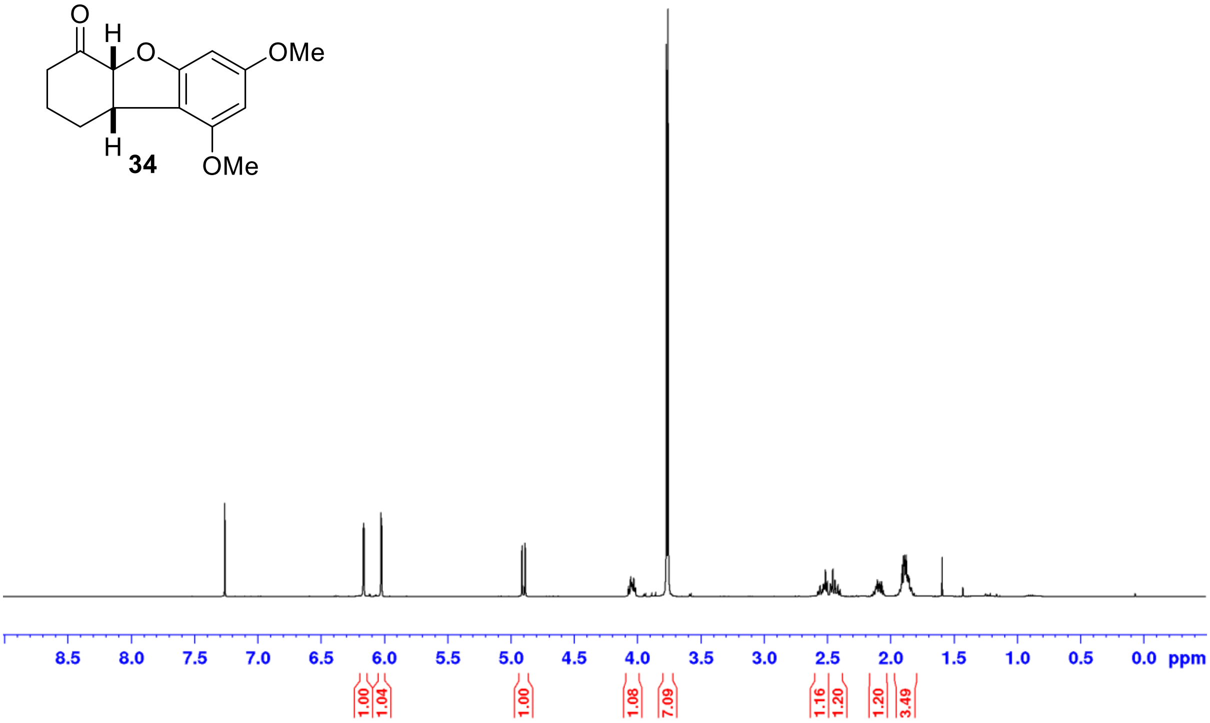
O
H
O
OMe
H
34
OMe
8.5
8.0
7.5
7.0
6.5
6.0
5.5
5.0
4.5
4.0
3.5
3.0
2.5
2.0
1.5
1.0
0.5
0.0 ppm
1.00
1.04
1.00
1.08
7.09
1.16
1.20
1.20
3.49

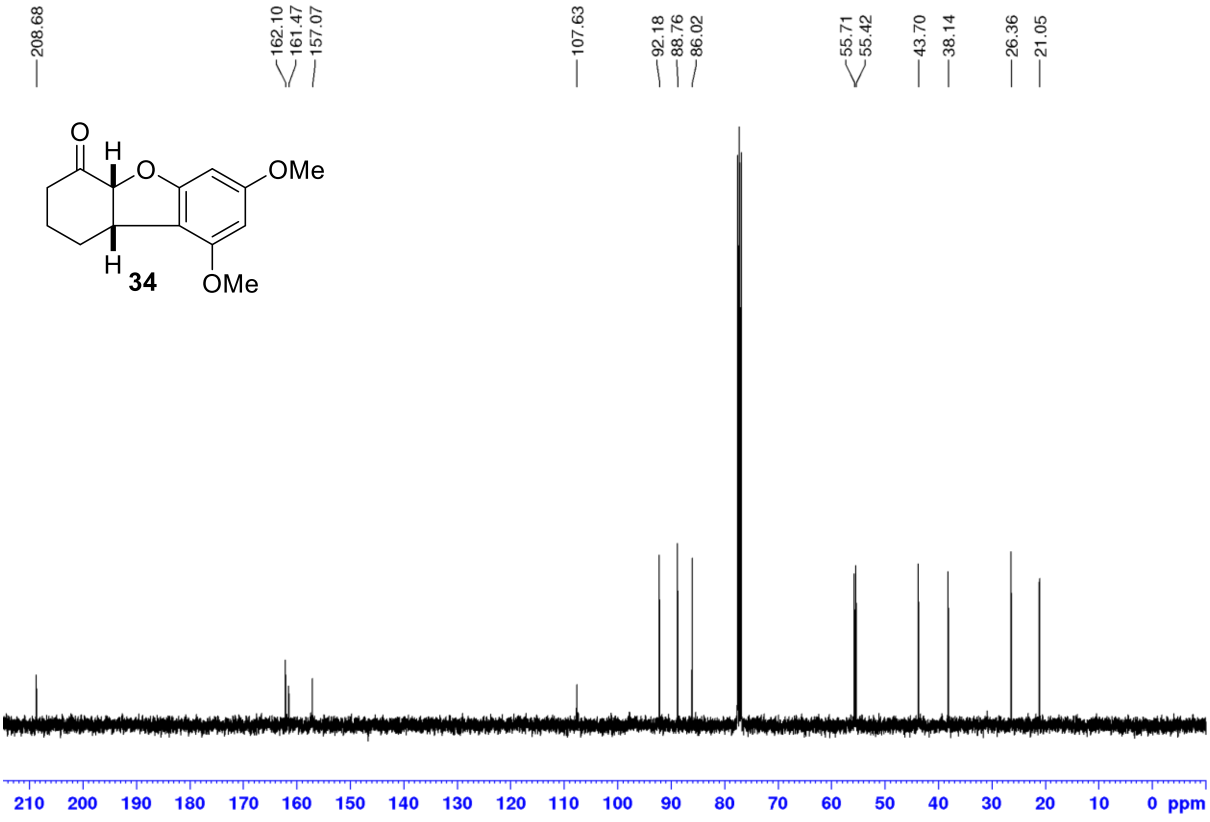
208.68
162.10
161.47
157.07
107.63
92.18
88.76
86.02
55.71
55.42
43.70
38.14
26.36
21.05
O
H
O
OMe
H
34
OMe
210
200
190
180
170
160
150
140
130
120
110
100
90
80
70
60
50
40
30
20
10
0 ppm

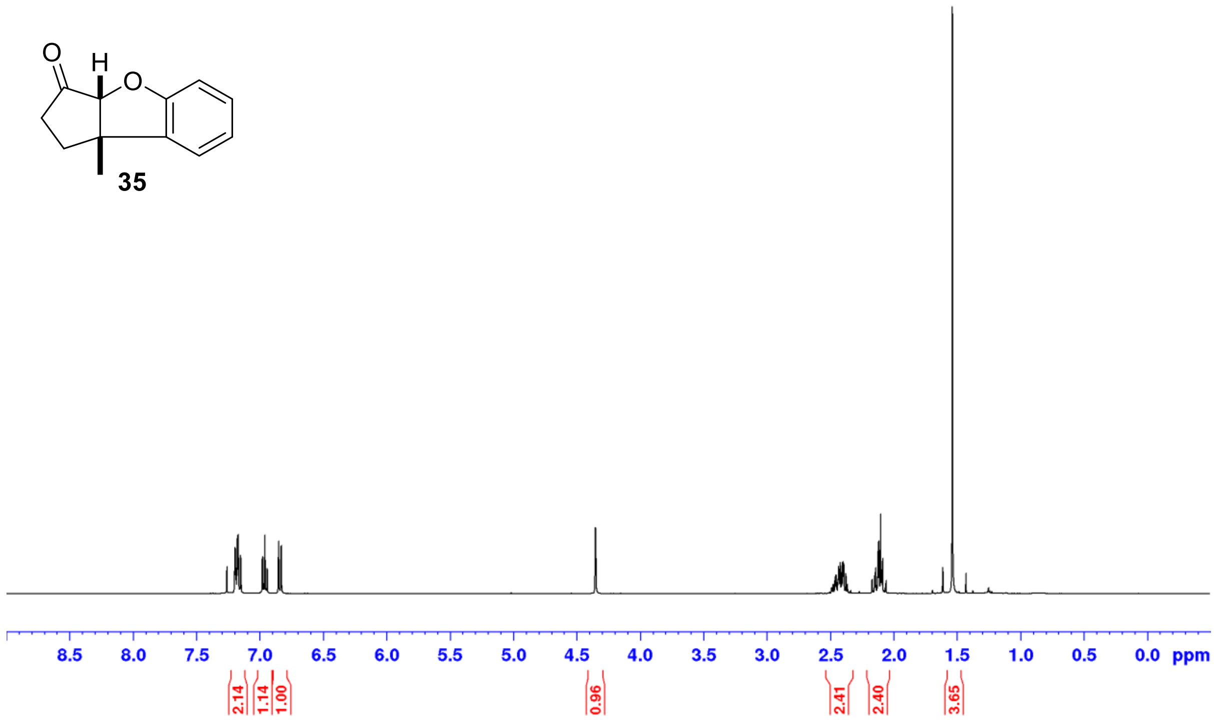
O
H
O
35
8.5
8.0
7.5
7.0
6.5
6.0
5.5
5.0
4.5
4.0
3.5
3.0
2.5
2.0
1.5
1.0
0.5
0.0
ppm
2.14
1.14
1.00
0.96
2.41
2.40
3.65

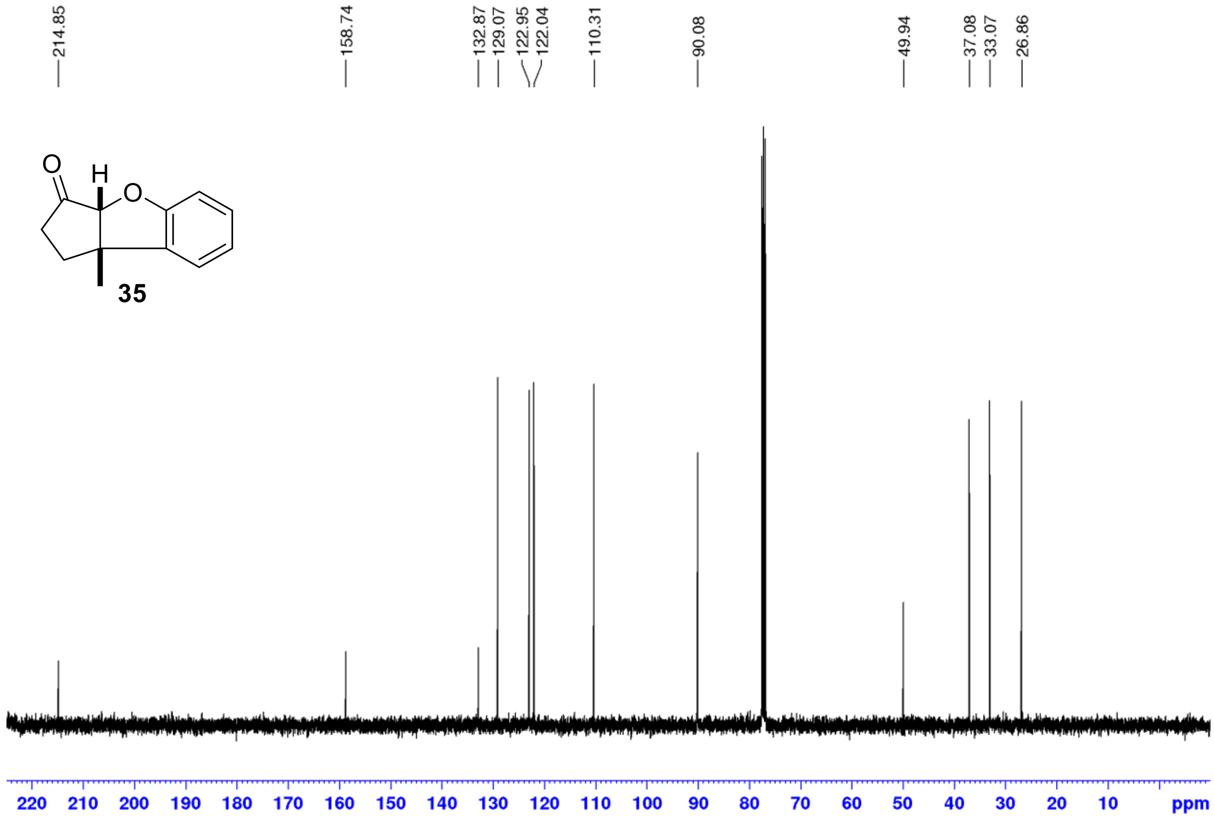
214.85
158.74
132.87
129.07
122.95
122.04
110.31
90.08
49.94
37.08
33.07
26.86
O
H
O
35
220
210
200
190
180
170
160
150
140
130
120
110
100
90
80
70
60
50
40
30
20
10
ppm

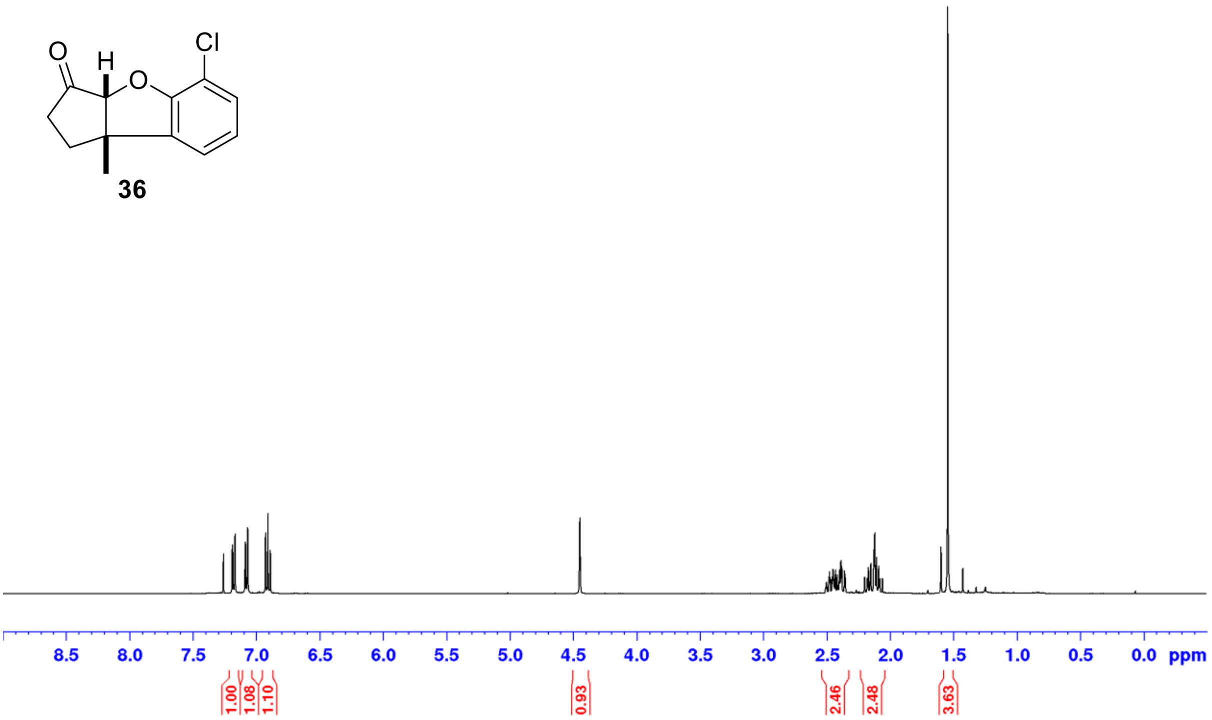
Cl
O
H
O
36
8.5
8.0
7.5
7.0
6.5
6.0
5.5
5.0
4.5
4.0
3.5
3.0
2.5
2.0
1.5
1.0
0.5
0.0
ppm
1.00
1.08
1.10
0.93
2.46
2.48
3.63

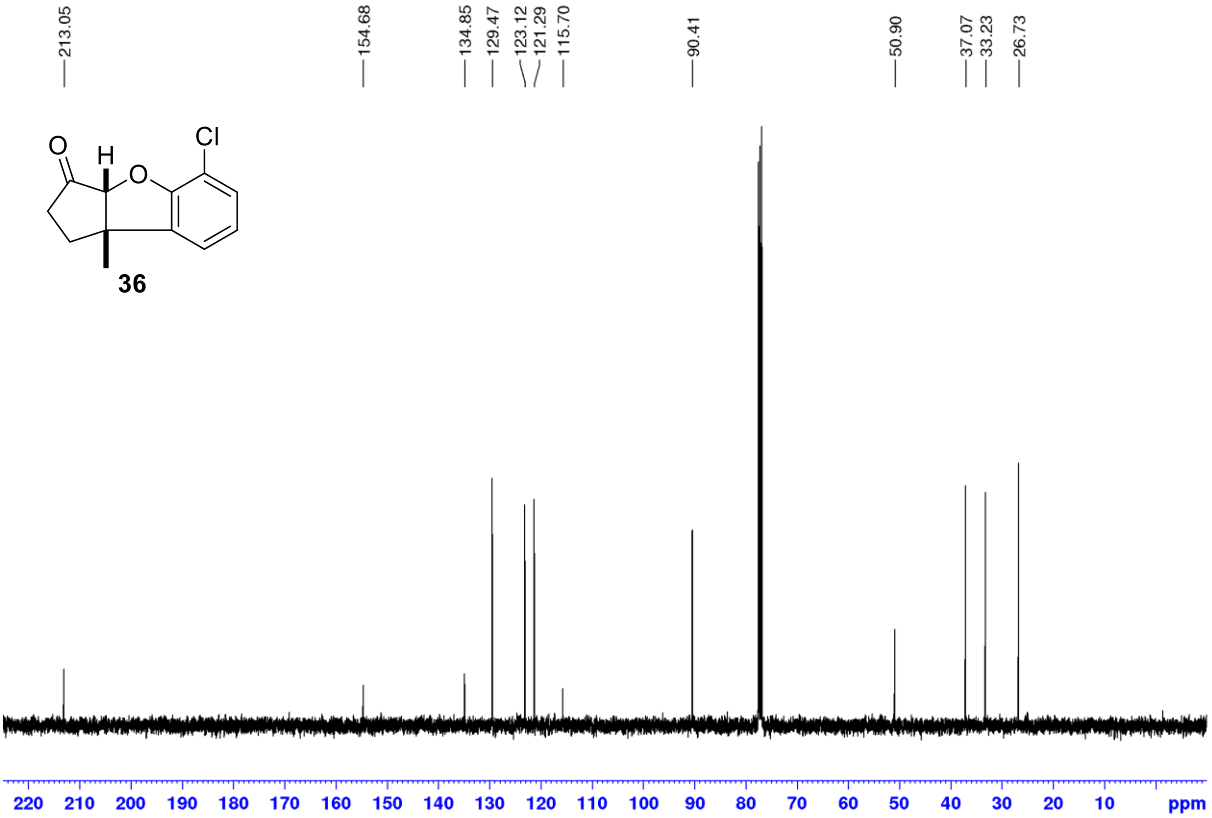
213.05
154.68
134.85
129.47
123.12
121.29
115.70
90.41
50.90
37.07
33.23
26.73
Cl
O
H
O
36
220
210
200
190
180
170
160
150
140
130
120
110
100
90
80
70
60
50
40
30
20
10
ppm

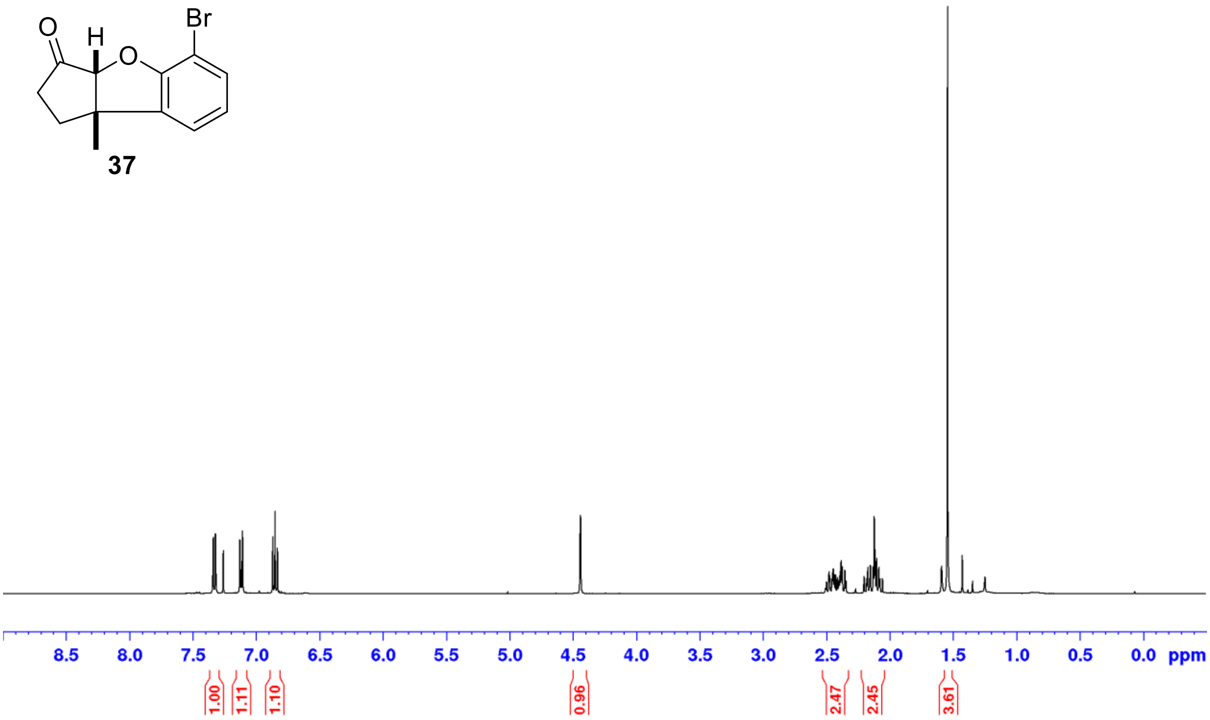

37
8.5
8.0
7.5
7.0
6.5
6.0
5.5
5.0
4.5
4.0
3.5
3.0
2.5
2.0
1.5
1.0
0.5
0.0
ppm
1.00
1.11
1.10
0.96
2.47
2.45
3.61

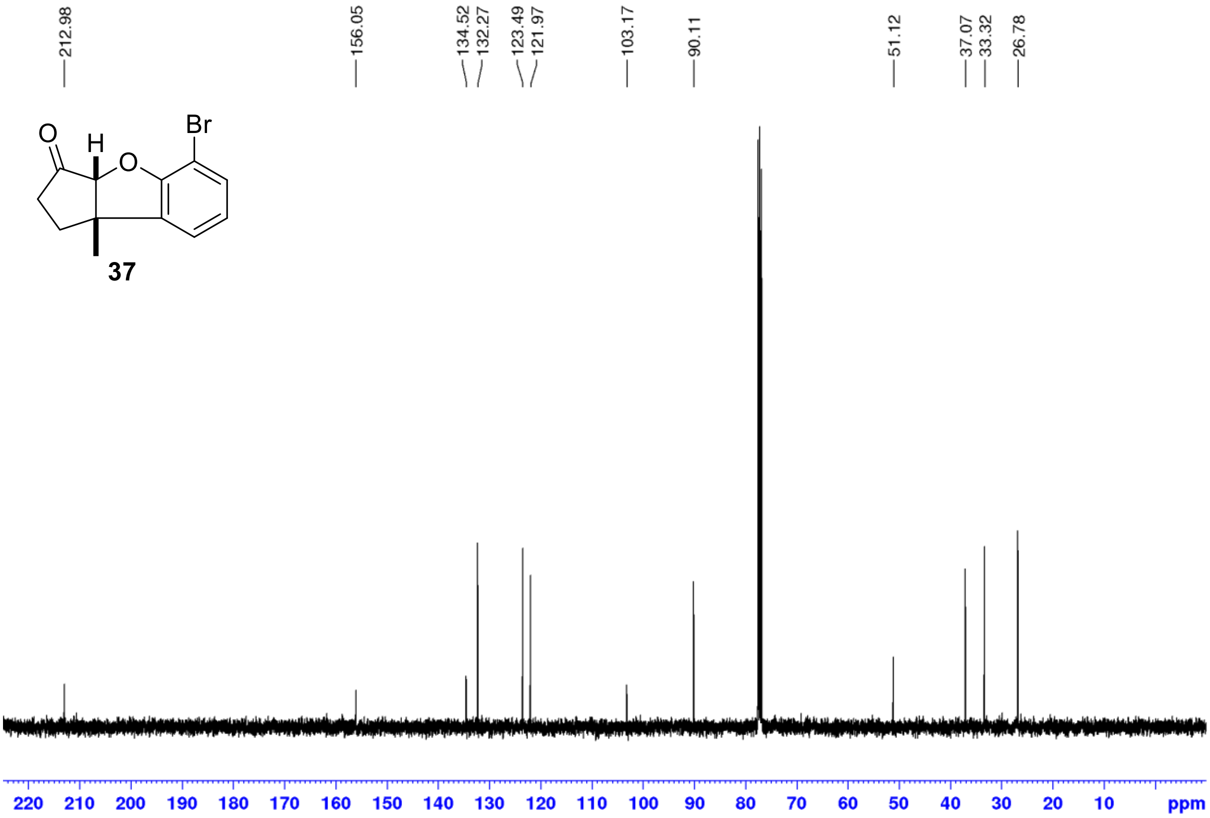

37
212.98
156.05
134.52
132.27
123.49
121.97
103.17
90.11
51.12
37.07
33.32
26.78
220
210
200
190
180
170
160
150
140
130
120
110
100
90
80
70
60
50
40
30
20
10
ppm

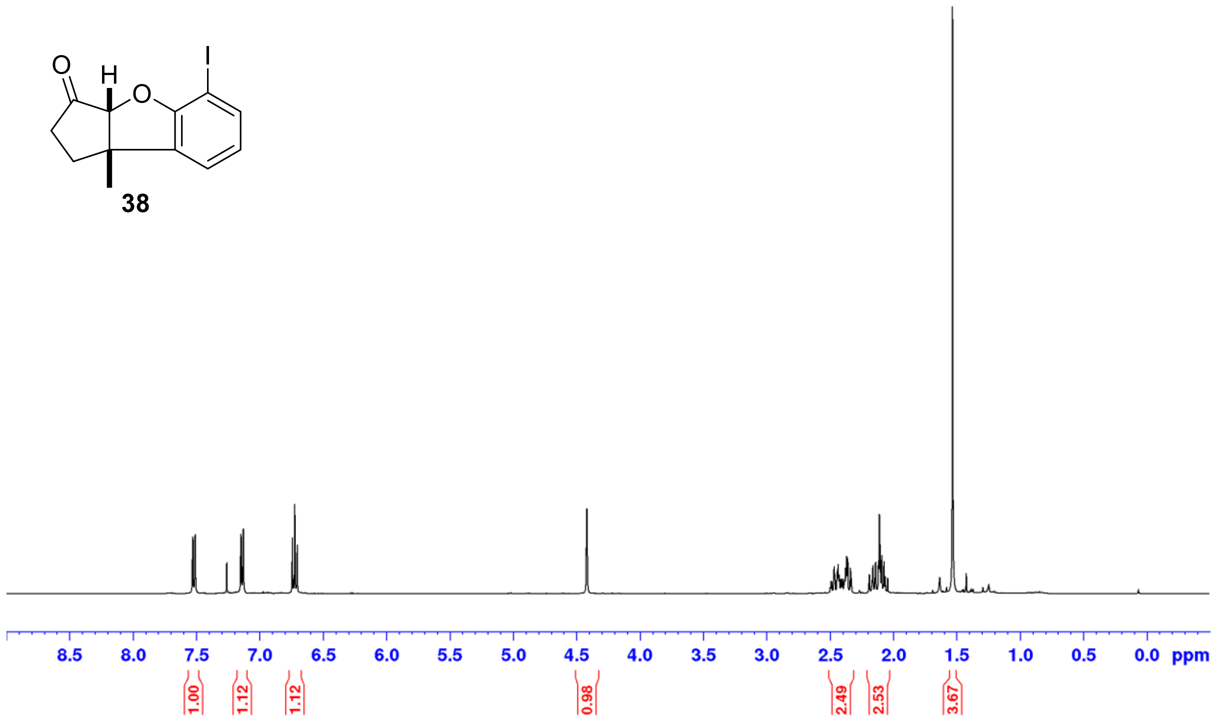
O
H
O
I
38
8.5 8.0 7.5 7.0 6.5 6.0 5.5 5.0 4.5 4.0 3.5 3.0 2.5 2.0 1.5 1.0 0.5 0.0 ppm
1.00
1.12
1.12
0.98
2.49
2.53
3.67

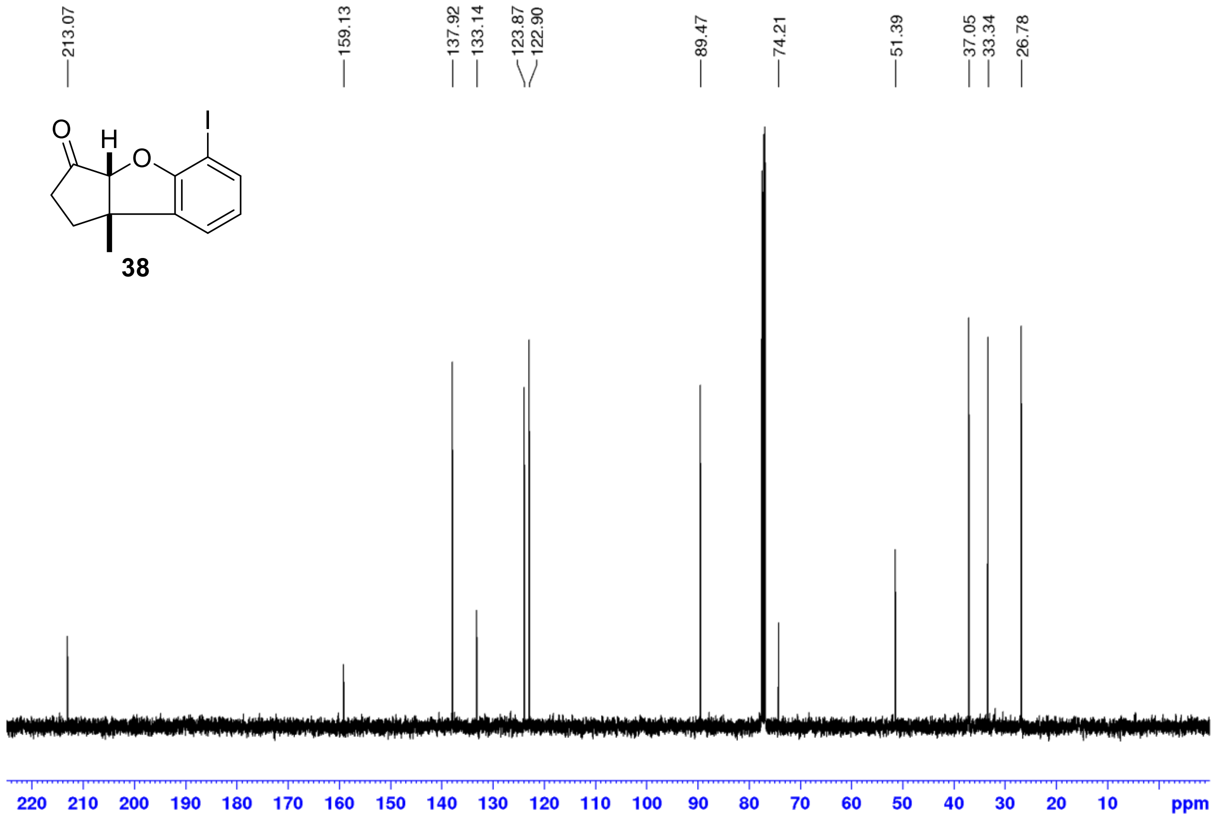
213.07
159.13
137.92
133.14
123.87
122.90
89.47
74.21
51.39
37.05
33.34
26.78
O
H
O
I
38
220 210 200 190 180 170 160 150 140 130 120 110 100 90 80 70 60 50 40 30 20 10 ppm

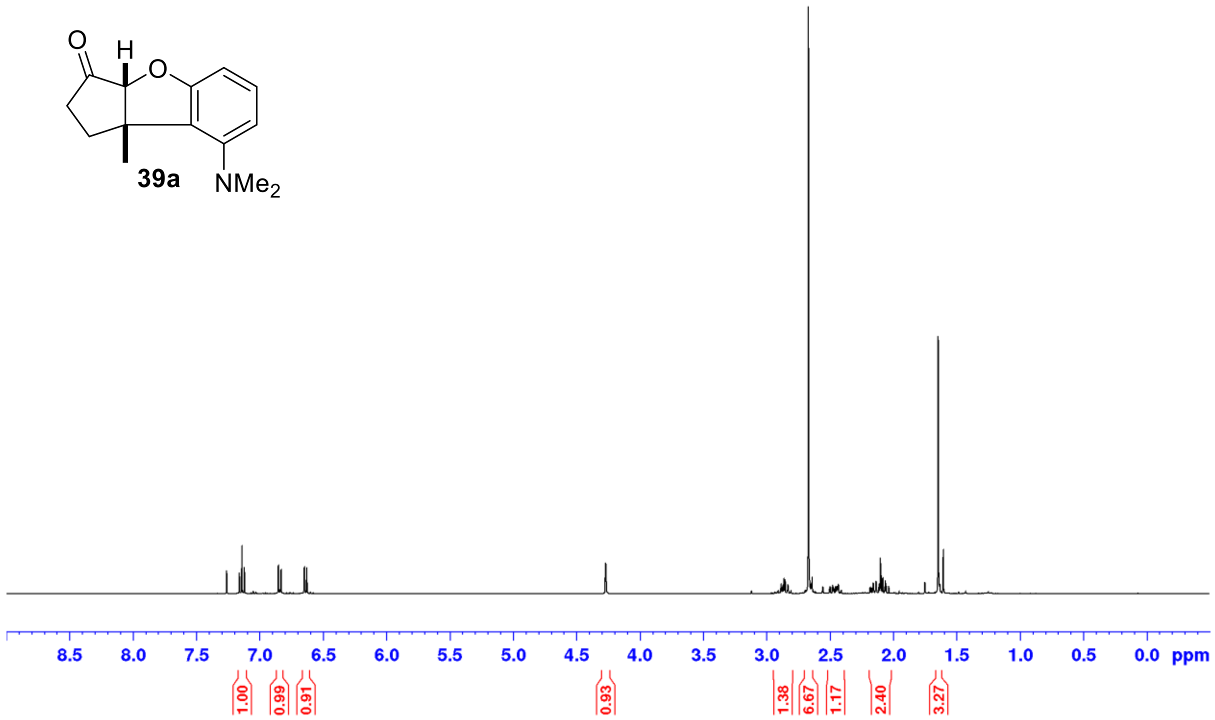
39a
NMe2
8.5
8.0
7.5
7.0
6.5
6.0
5.5
5.0
4.5
4.0
3.5
3.0
2.5
2.0
1.5
1.0
0.5
0.0
ppm
1.00
0.99
0.91
0.93
1.38
6.67
1.17
2.40
3.27

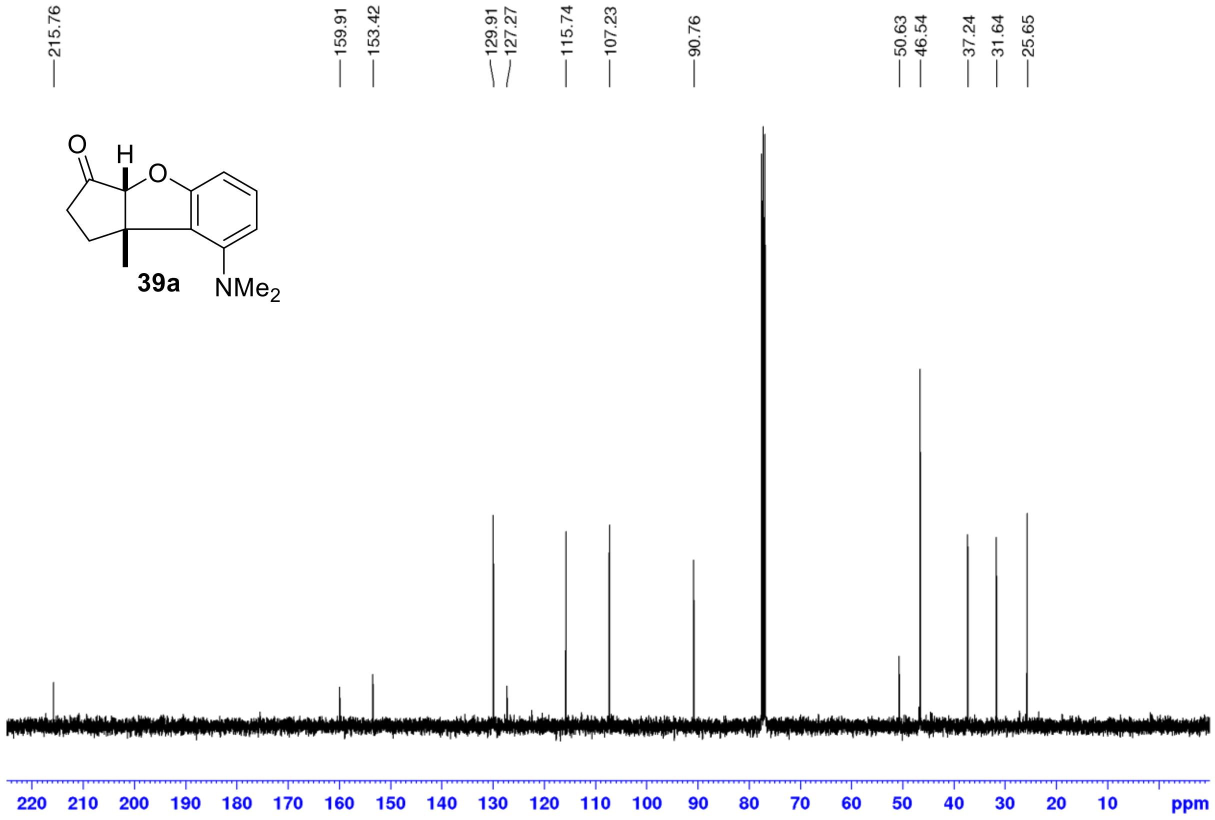
215.76
159.91
153.42
129.91
127.27
115.74
107.23
90.76
50.63
46.54
37.24
31.64
25.65
39a
NMe2
220
210
200
190
180
170
160
150
140
130
120
110
100
90
80
70
60
50
40
30
20
10
ppm

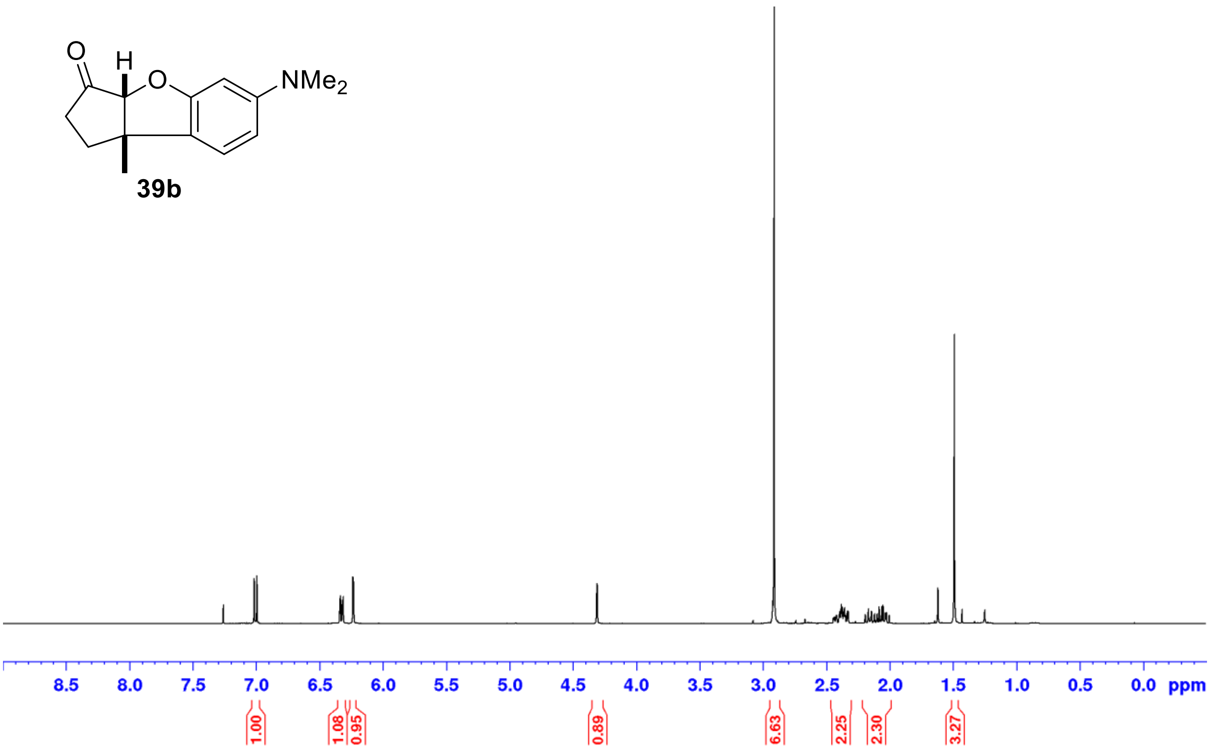

NMe2
39b
8.5
8.0
7.5
7.0
6.5
6.0
5.5
5.0
4.5
4.0
3.5
3.0
2.5
2.0
1.5
1.0
0.5
0.0
ppm
1.00
1.08
0.95
0.89
6.63
2.25
2.30
3.27

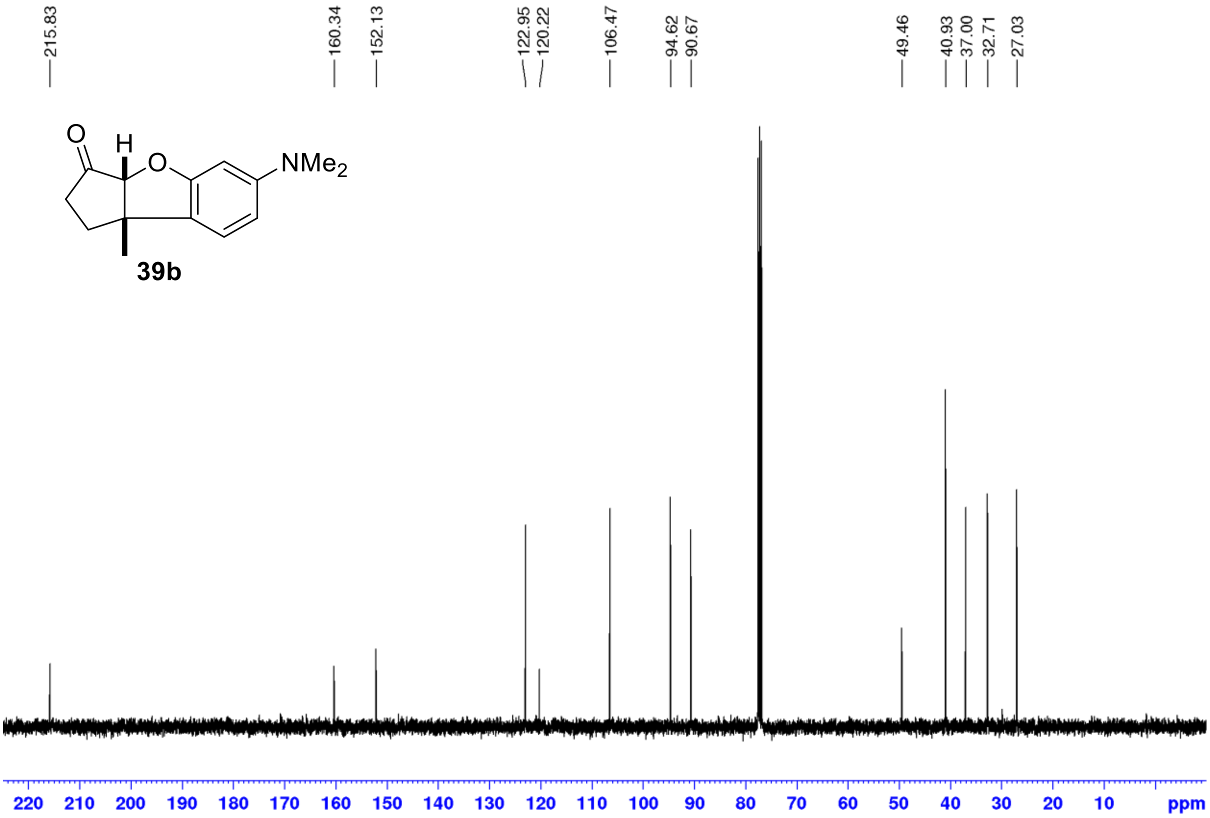

215.83
160.34
152.13
122.95
120.22
106.47
94.62
90.67
49.46
40.93
37.00
32.71
27.03
NMe2
39b
220
210
200
190
180
170
160
150
140
130
120
110
100
90
80
70
60
50
40
30
20
10
ppm

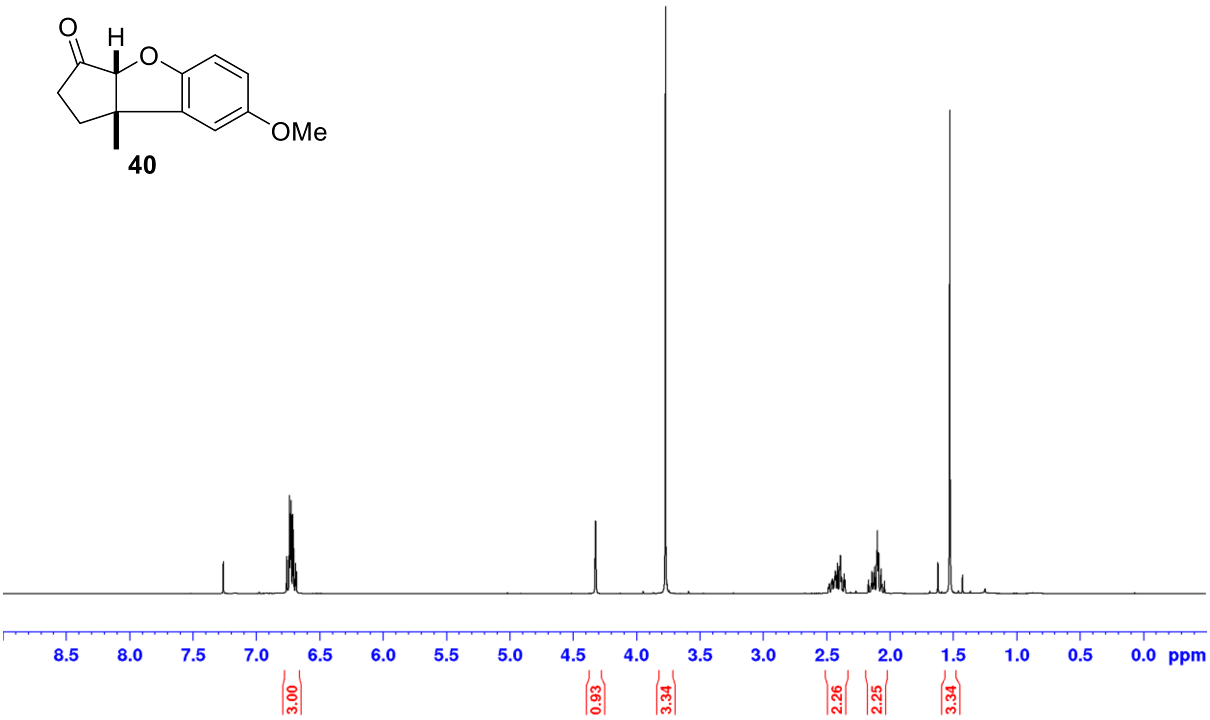
O
H
O
OMe
40
8.5
8.0
7.5
7.0
6.5
6.0
5.5
5.0
4.5
4.0
3.5
3.0
2.5
2.0
1.5
1.0
0.5
0.0
ppm
3.00
0.93
3.34
2.26
2.25
3.34

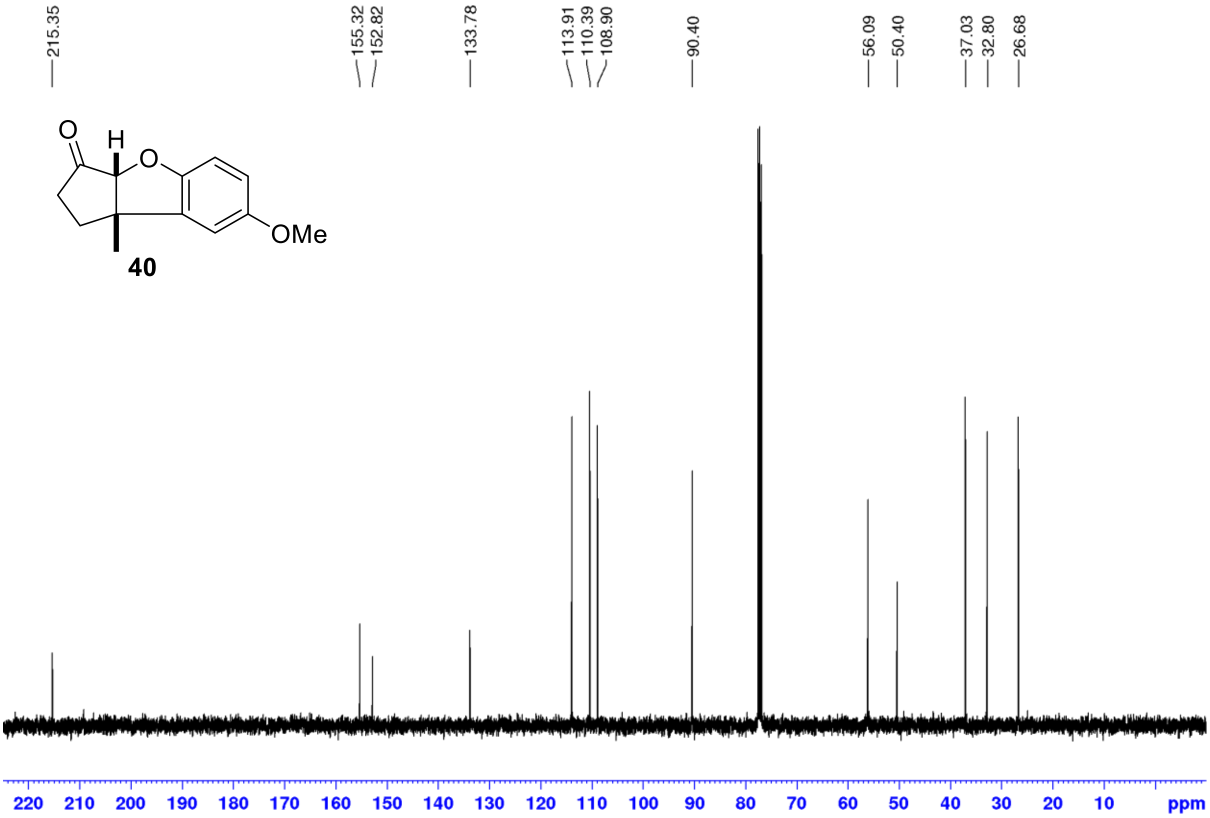
215.35
155.32
152.82
133.78
113.91
110.39
108.90
90.40
56.09
50.40
37.03
32.80
26.68
O
H
O
OMe
40
220
210
200
190
180
170
160
150
140
130
120
110
100
90
80
70
60
50
40
30
20
10
ppm

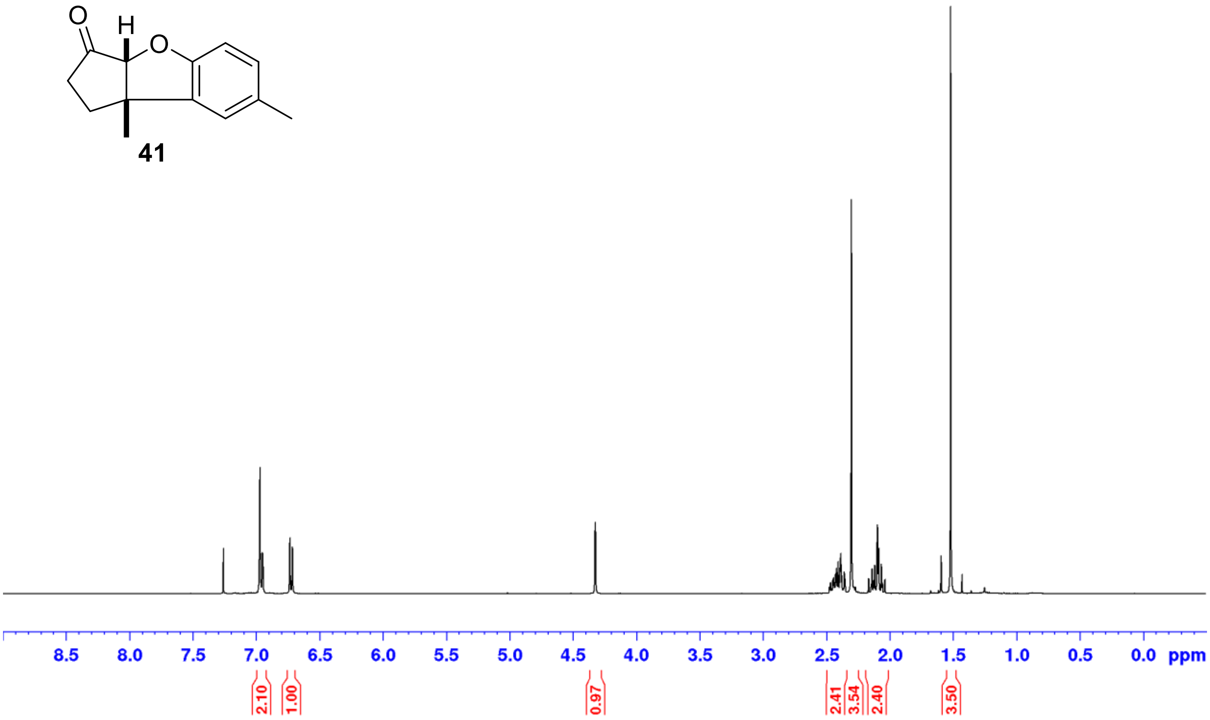

O
H
O
41
8.5
8.0
7.5
7.0
6.5
6.0
5.5
5.0
4.5
4.0
3.5
3.0
2.5
2.0
1.5
1.0
0.5
0.0
ppm
2.10
1.00
0.97
2.41
3.54
2.40
3.50

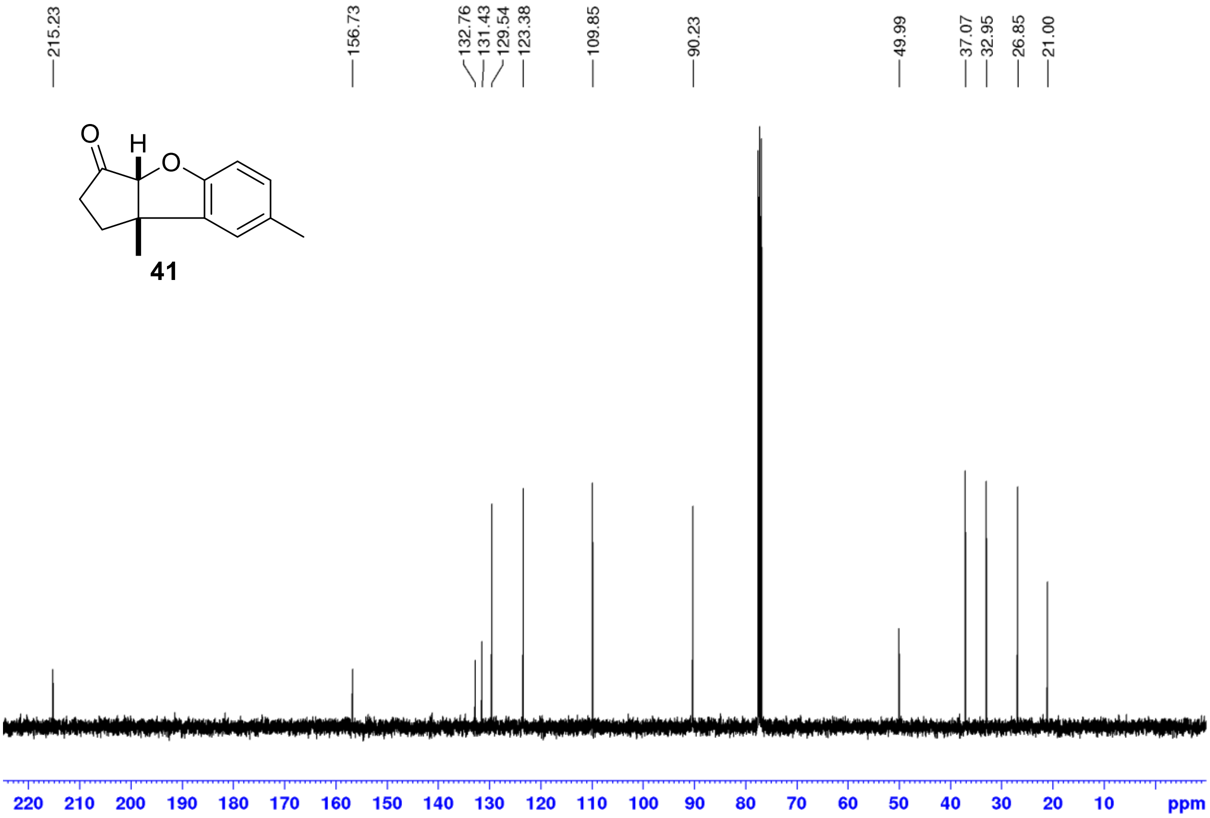

215.23
156.73
132.76
131.43
129.54
123.38
109.85
90.23
49.99
37.07
32.95
26.85
21.00
O
H
O
41
220
210
200
190
180
170
160
150
140
130
120
110
100
90
80
70
60
50
40
30
20
10
ppm

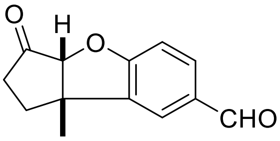

42

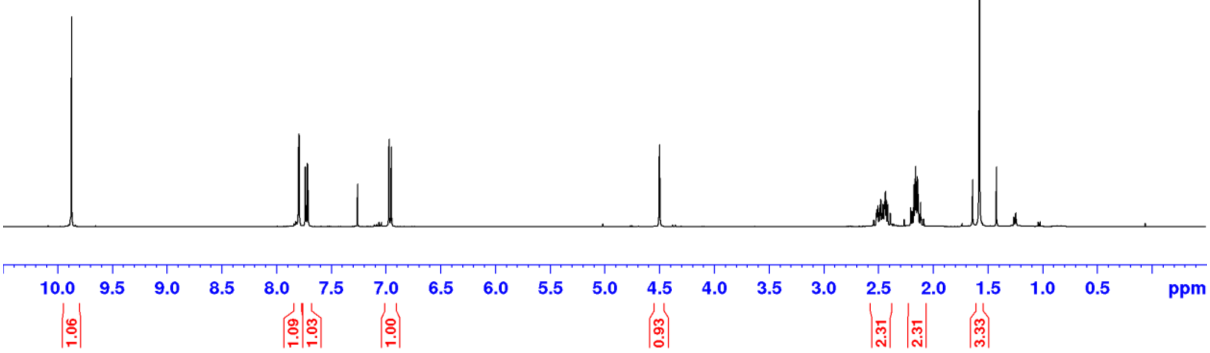
10.0 9.5 9.0 8.5 8.0 7.5 7.0 6.5 6.0 5.5 5.0 4.5 4.0 3.5 3.0 2.5 2.0 1.5 1.0 0.5 ppm
1.06 1.09 1.03 1.00 0.93 2.31 2.31 3.33

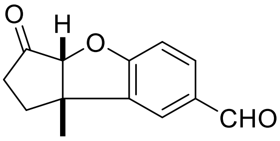

42

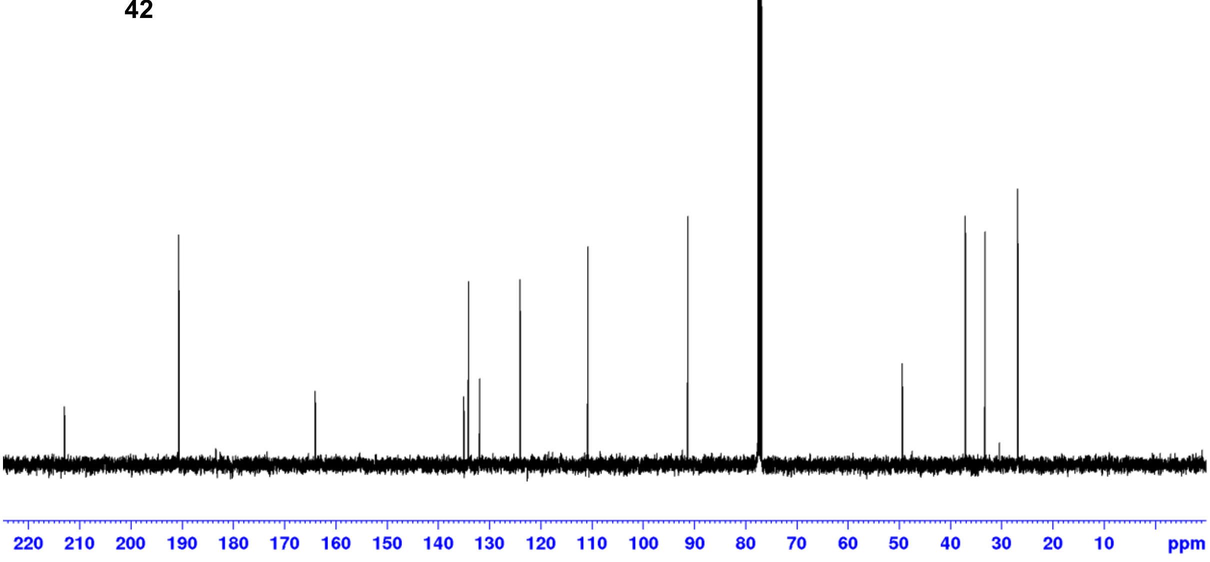
212.93 190.62 163.97 134.96 134.07 131.91 123.95 110.71 91.25 49.36 37.02 33.20 26.78
220 210 200 190 180 170 160 150 140 130 120 110 100 90 80 70 60 50 40 30 20 10 ppm

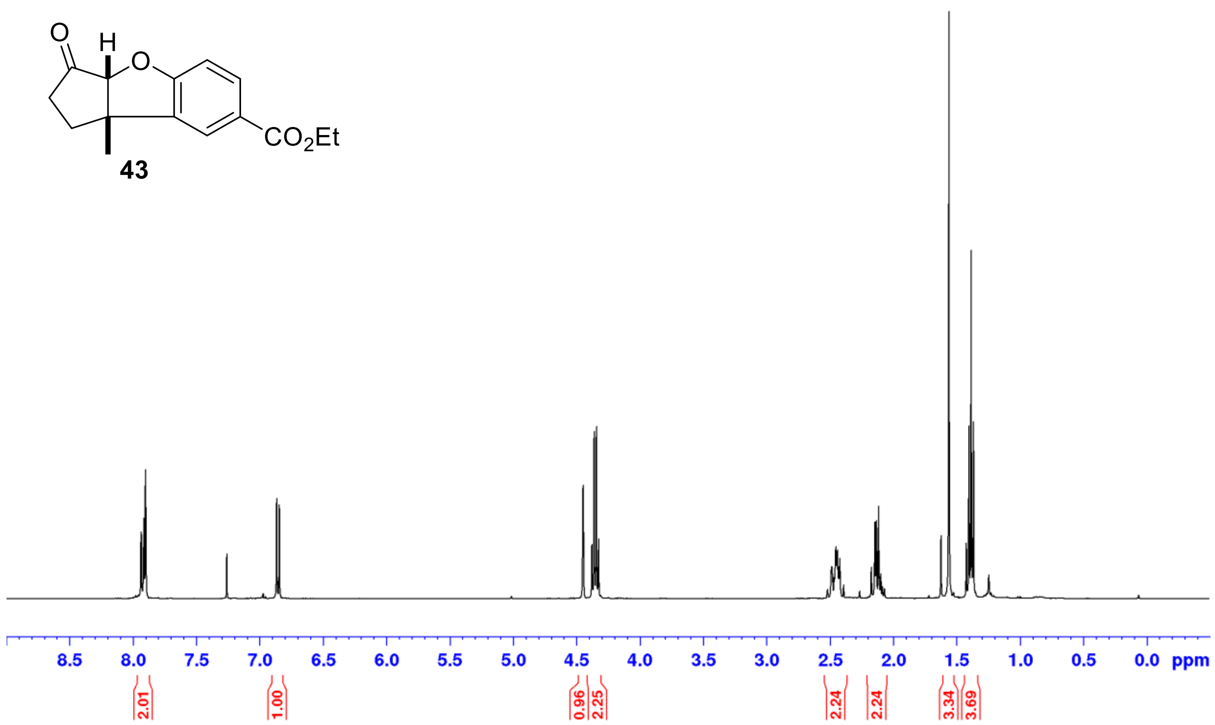
O
H
O
CO2Et
43
8.5
8.0
7.5
7.0
6.5
6.0
5.5
5.0
4.5
4.0
3.5
3.0
2.5
2.0
1.5
1.0
0.5
0.0
ppm
2.01
1.00
0.96
2.25
2.24
2.24
3.34
3.69

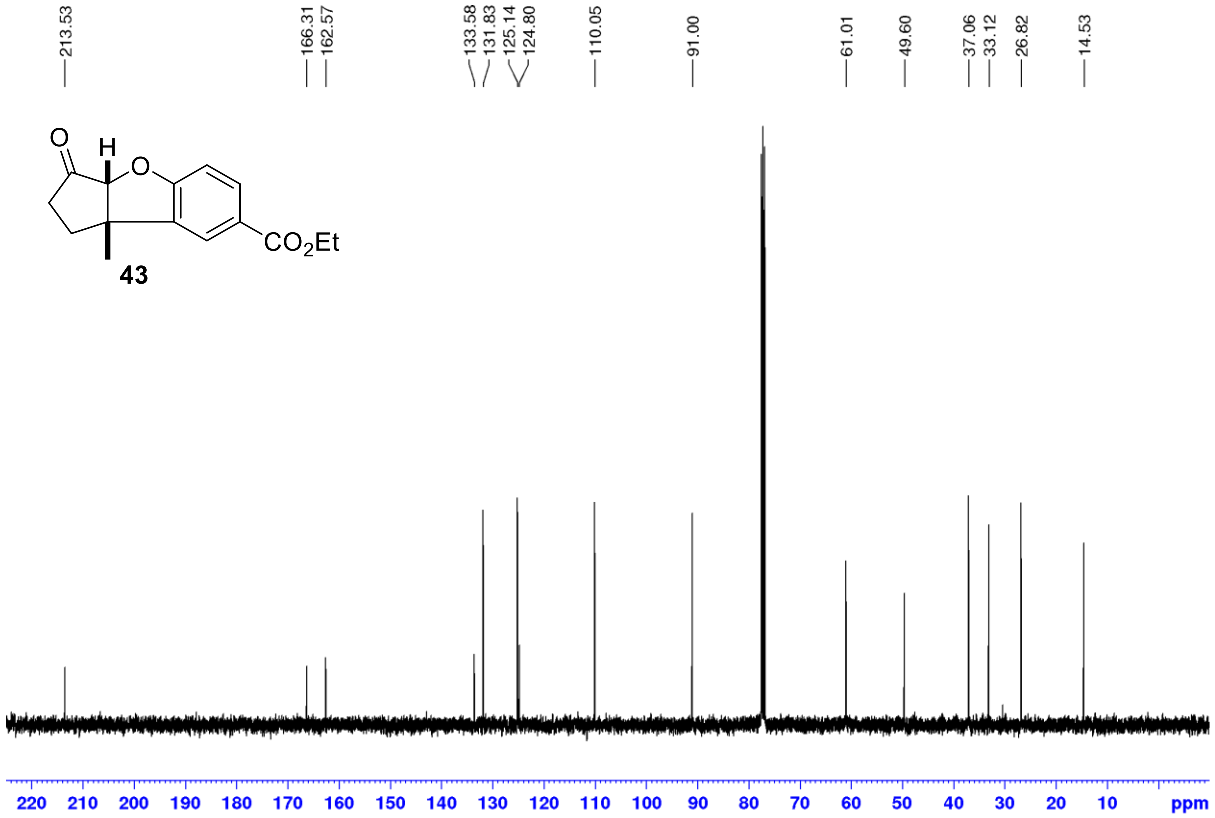
213.53
166.31
162.57
133.58
131.83
125.14
124.80
110.05
91.00
61.01
49.60
37.06
33.12
26.82
14.53
O
H
O
CO2Et
43
220
210
200
190
180
170
160
150
140
130
120
110
100
90
80
70
60
50
40
30
20
10
ppm

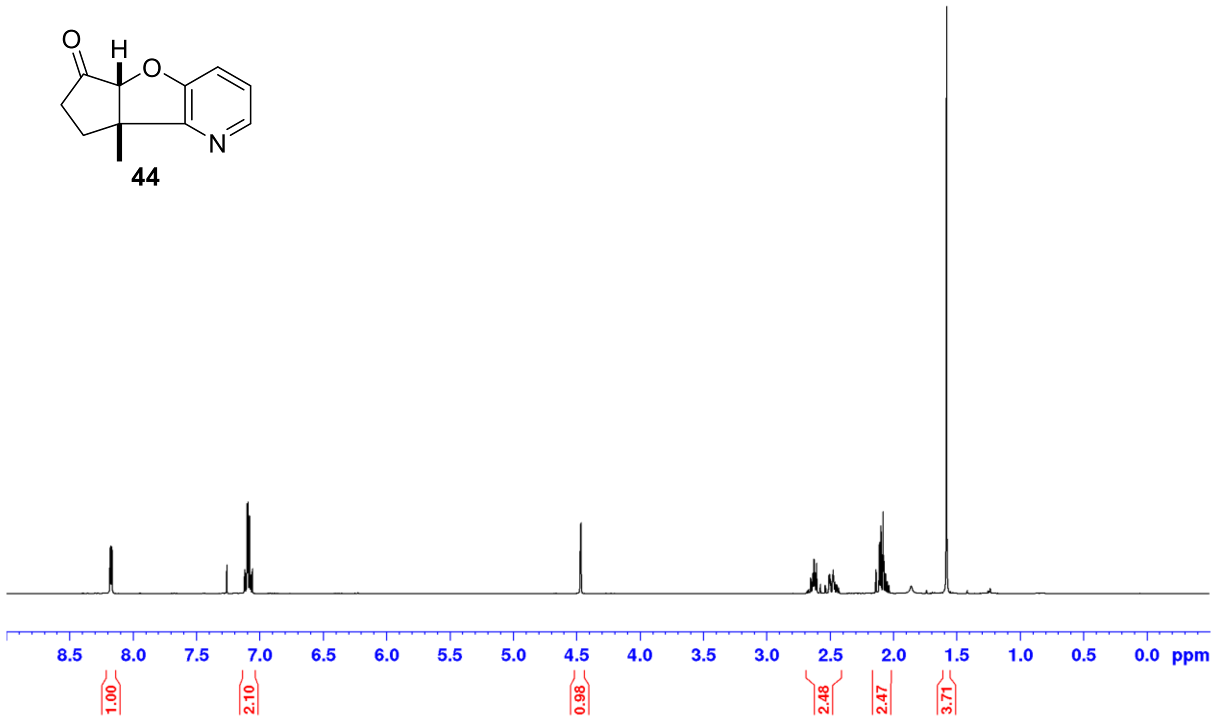

44
8.5 8.0 7.5 7.0 6.5 6.0 5.5 5.0 4.5 4.0 3.5 3.0 2.5 2.0 1.5 1.0 0.5 0.0 ppm
1.00
2.10
0.98
2.48
2.47
3.71

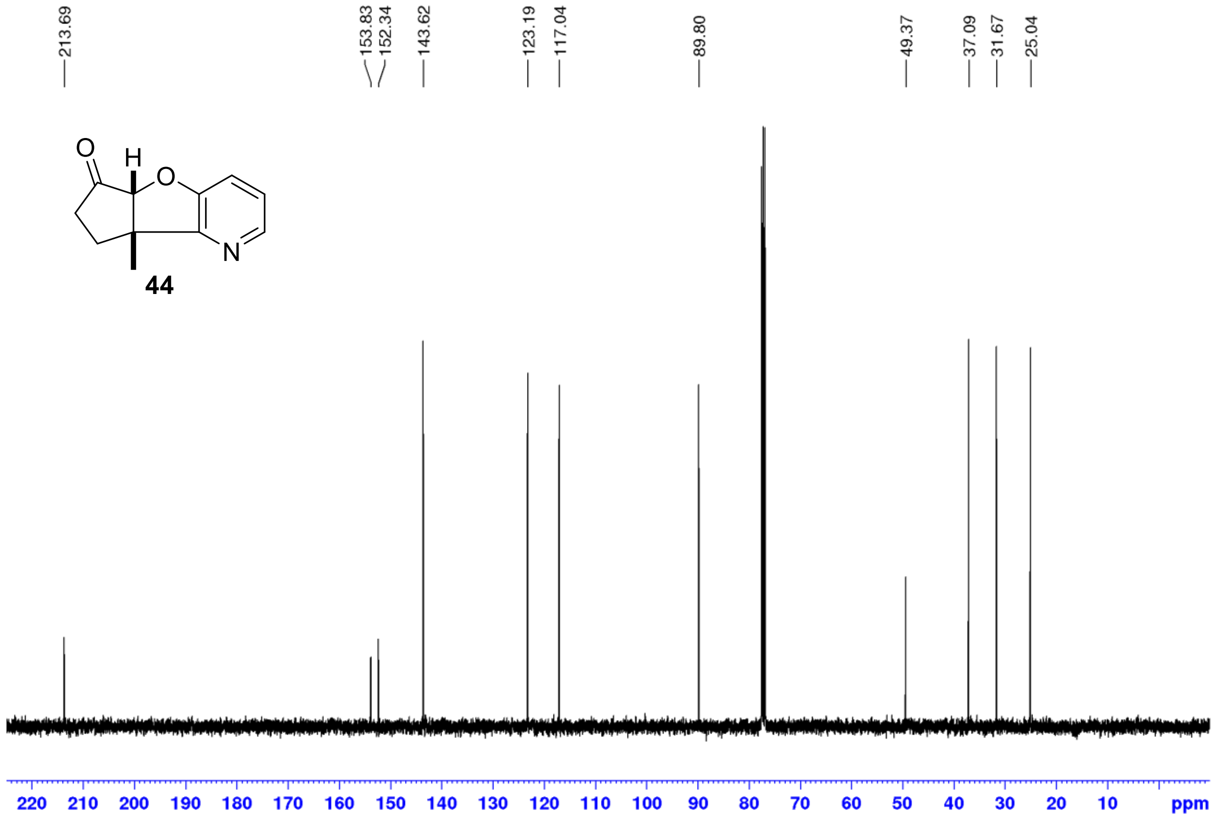

213.69
153.83
152.34
143.62
123.19
117.04
89.80
49.37
37.09
31.67
25.04
44
220 210 200 190 180 170 160 150 140 130 120 110 100 90 80 70 60 50 40 30 20 10 ppm

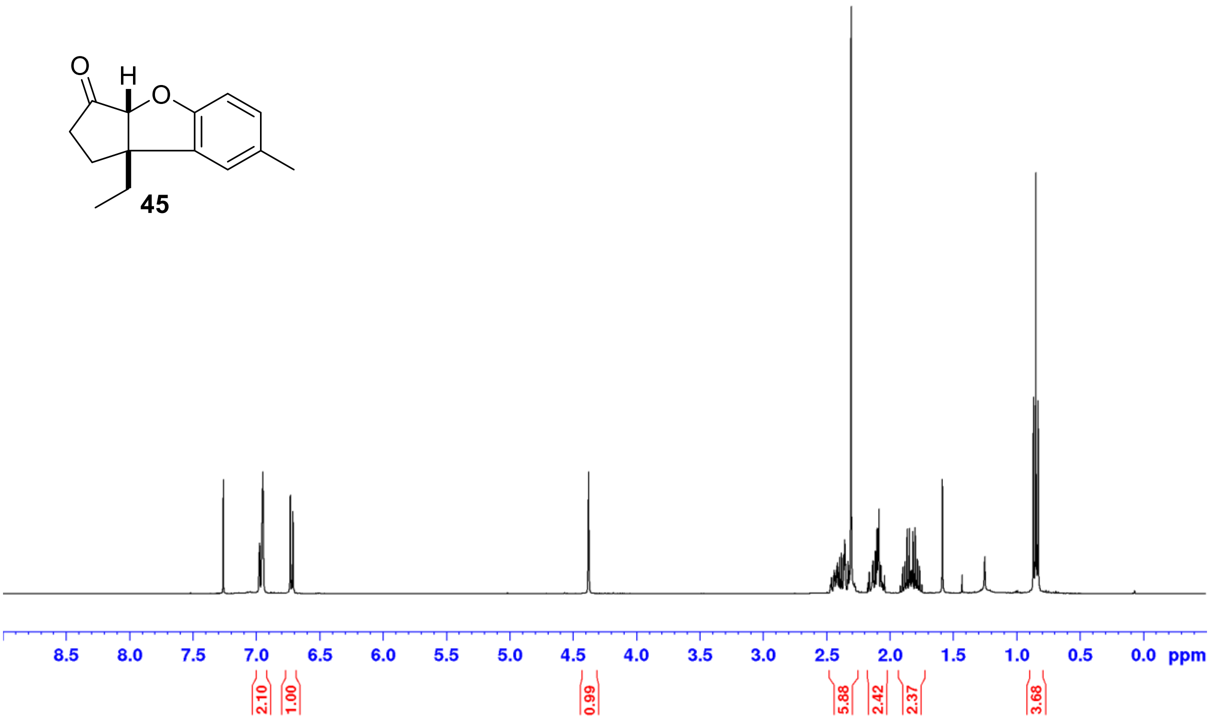
45
8.5 8.0 7.5 7.0 6.5 6.0 5.5 5.0 4.5 4.0 3.5 3.0 2.5 2.0 1.5 1.0 0.5 0.0 ppm
2.10
1.00
0.99
5.88
2.42
2.37
3.68

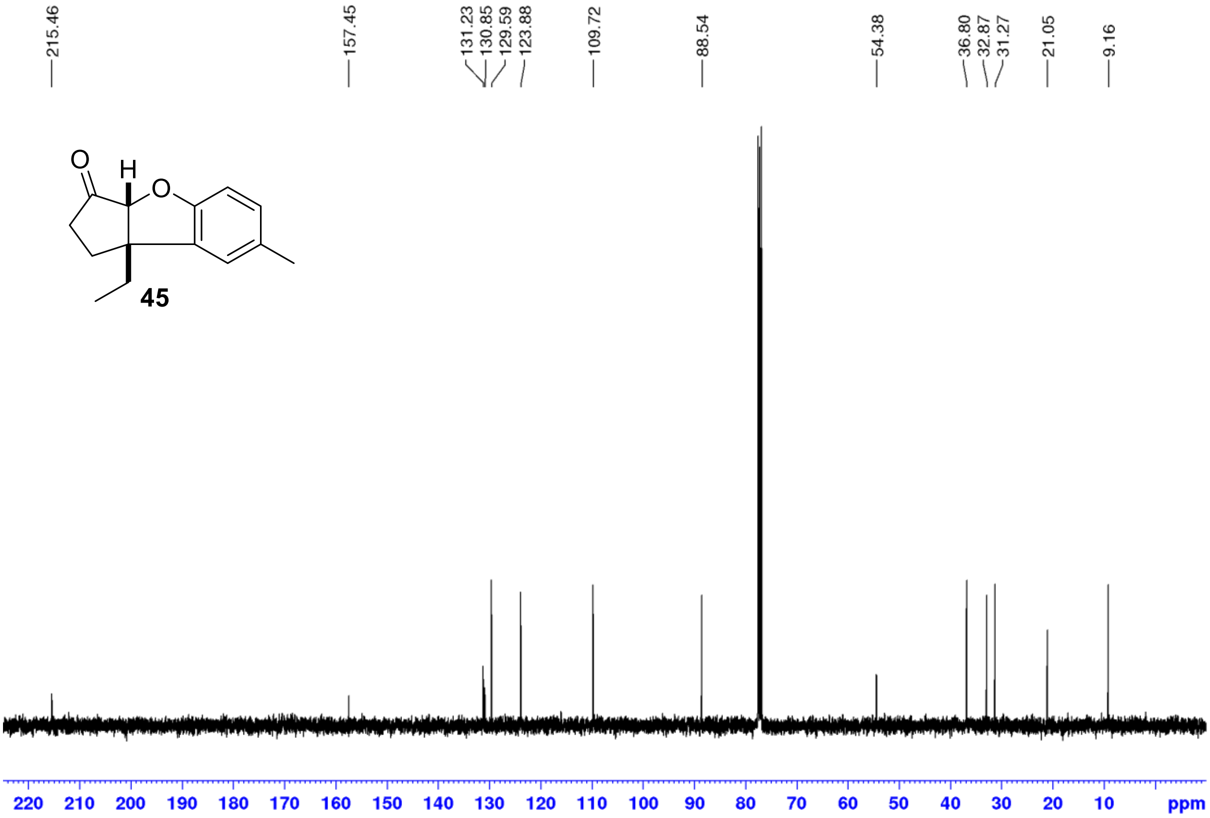
215.46
157.45
131.23
130.85
129.59
123.88
109.72
88.54
54.38
36.80
32.87
31.27
21.05
9.16
45
220 210 200 190 180 170 160 150 140 130 120 110 100 90 80 70 60 50 40 30 20 10 ppm

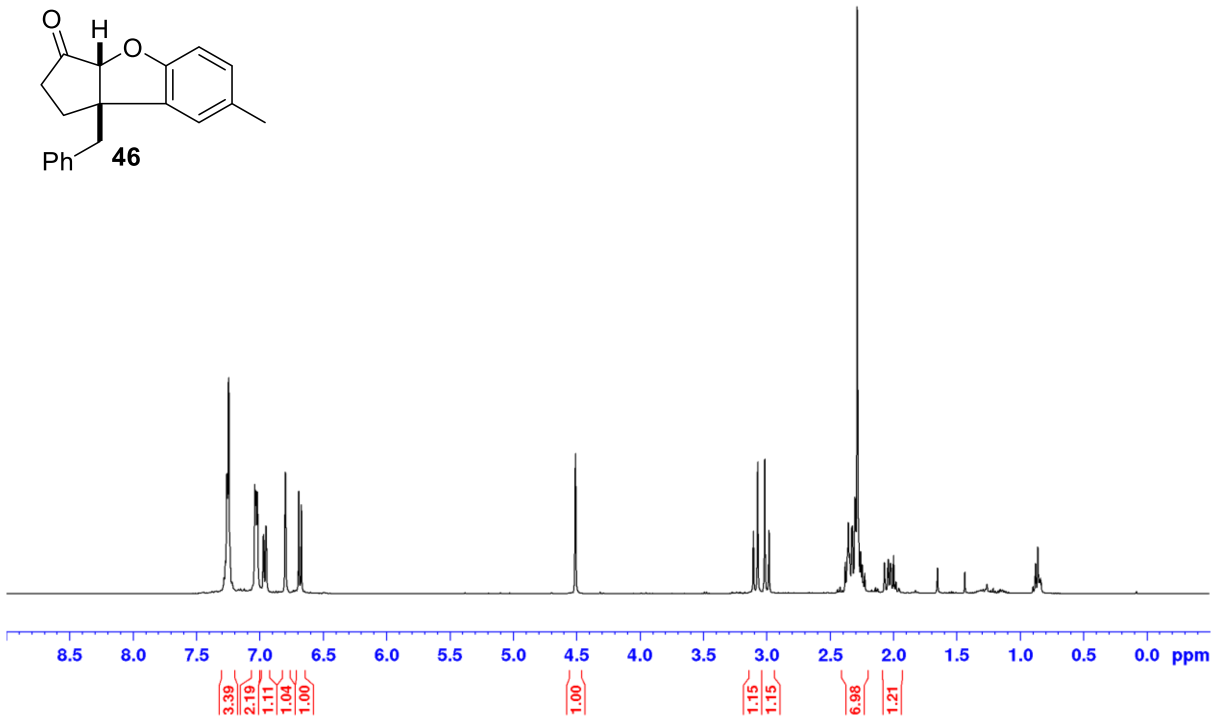
O
H
O
Ph
46
8.5
8.0
7.5
7.0
6.5
6.0
5.5
5.0
4.5
4.0
3.5
3.0
2.5
2.0
1.5
1.0
0.5
0.0
ppm
3.39
2.19
1.11
1.04
1.00
1.00
1.15
1.15
6.98
1.21

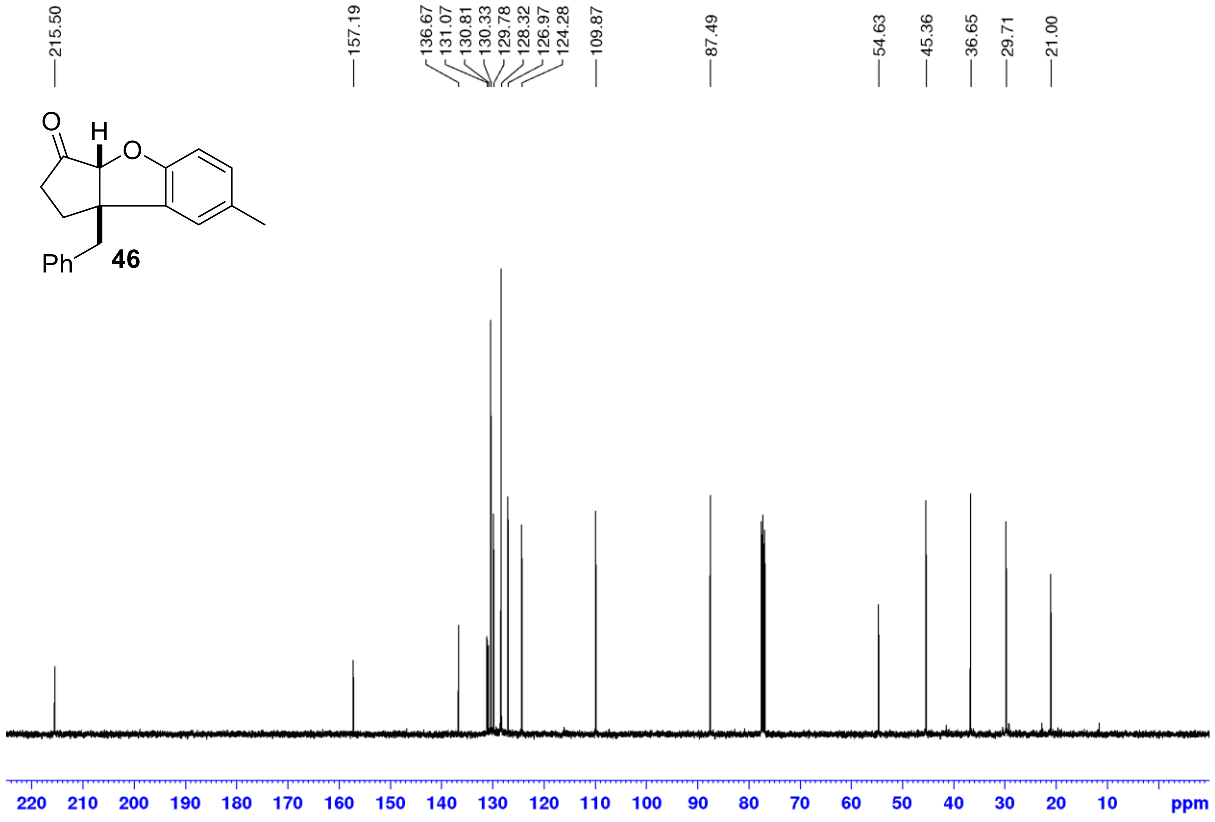
215.50
157.19
136.67
131.07
130.81
130.33
129.78
128.32
126.97
124.28
109.87
87.49
54.63
45.36
36.65
29.71
21.00
O
H
O
Ph
46
220
210
200
190
180
170
160
150
140
130
120
110
100
90
80
70
60
50
40
30
20
10
ppm

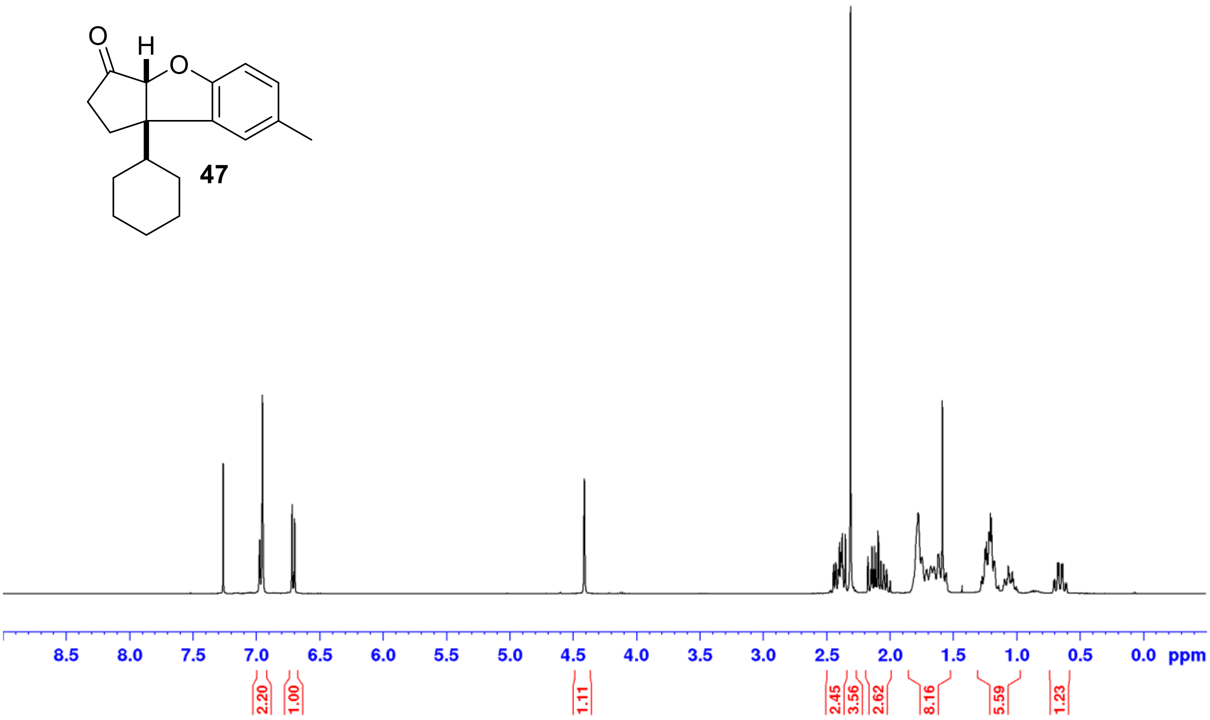
47
8.5 8.0 7.5 7.0 6.5 6.0 5.5 5.0 4.5 4.0 3.5 3.0 2.5 2.0 1.5 1.0 0.5 0.0 ppm
2.20 1.00 1.11 2.45 3.56 2.62 8.16 5.59 1.23

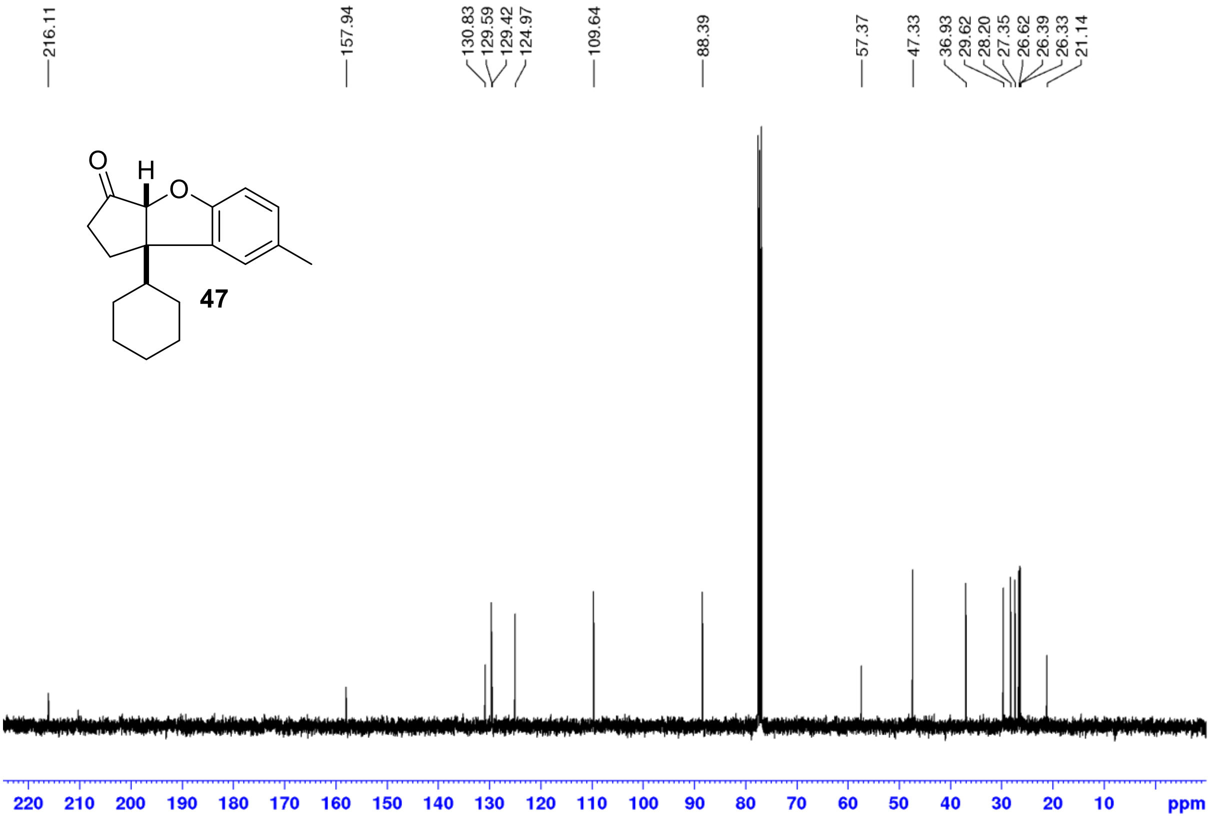
216.11
157.94
130.83
129.59
129.42
124.97
109.64
88.39
57.37
47.33
36.93
29.62
28.20
27.35
26.62
26.39
26.33
21.14
47
220 210 200 190 180 170 160 150 140 130 120 110 100 90 80 70 60 50 40 30 20 10 ppm

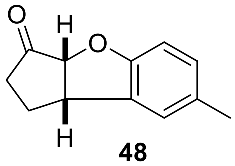

48

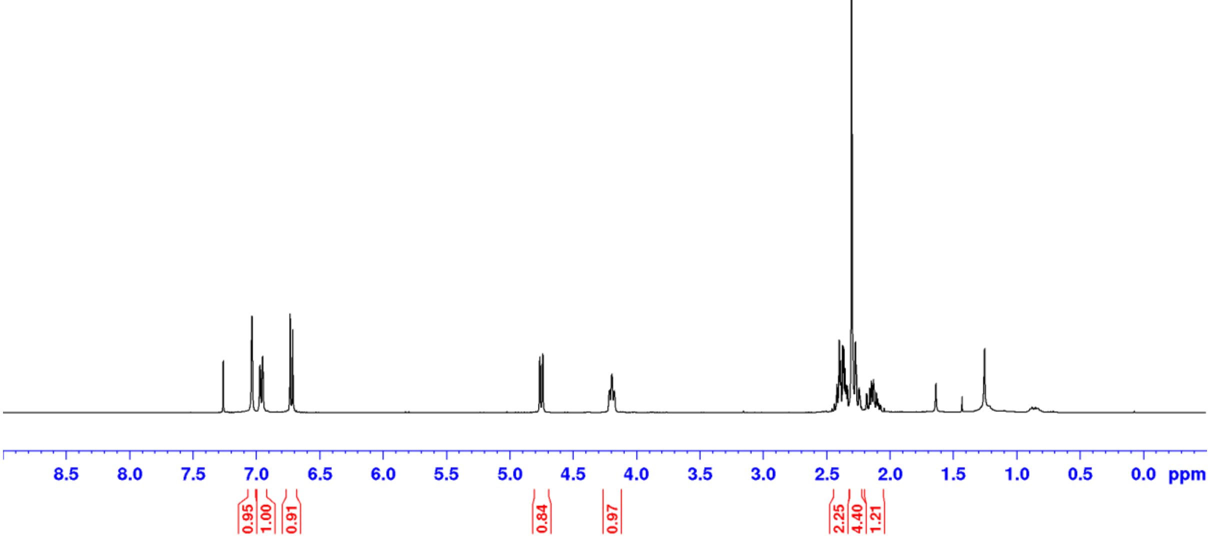
8.5
8.0
7.5
7.0
6.5
6.0
5.5
5.0
4.5
4.0
3.5
3.0
2.5
2.0
1.5
1.0
0.5
0.0
ppm
0.95
1.00
0.91
0.84
0.97
2.25
4.40
1.21

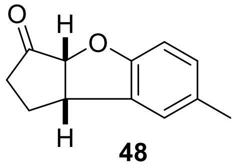

48

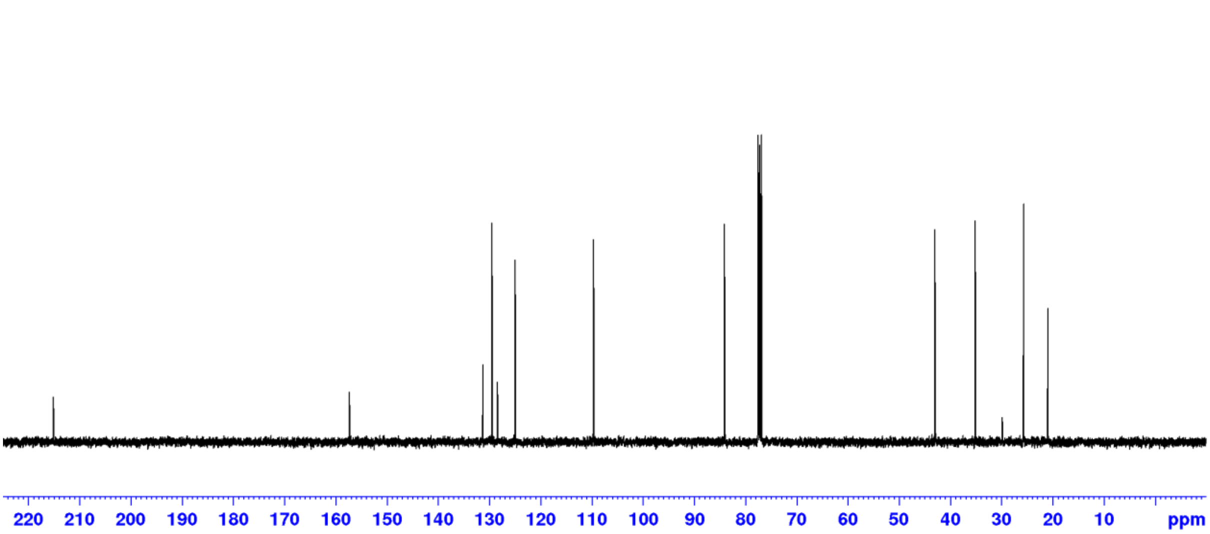
215.11
157.33
131.26
129.49
128.38
124.92
109.65
84.09
42.99
35.11
25.68
20.94
220
210
200
190
180
170
160
150
140
130
120
110
100
90
80
70
60
50
40
30
20
10
ppm

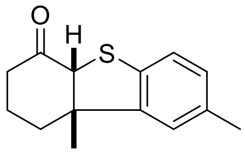
O
H
S

49

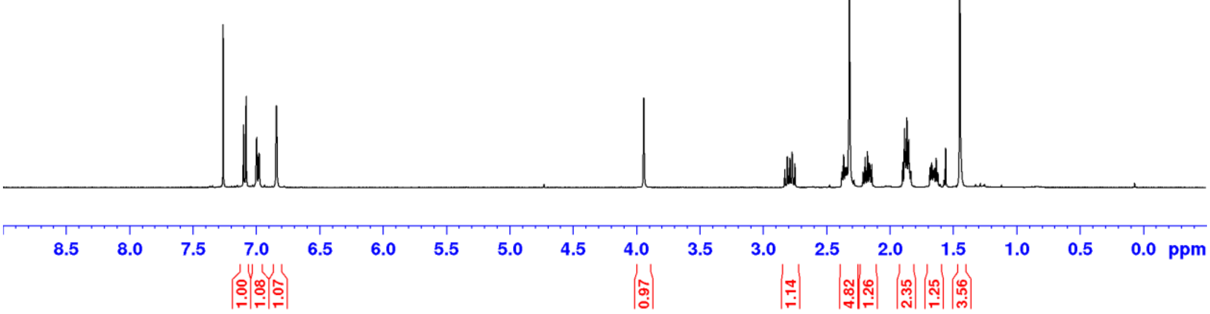
8.5 8.0 7.5 7.0 6.5 6.0 5.5 5.0 4.5 4.0 3.5 3.0 2.5 2.0 1.5 1.0 0.5 0.0 ppm
1.00 1.08 1.07 0.97 1.14 4.82 1.26 2.35 1.25 3.56

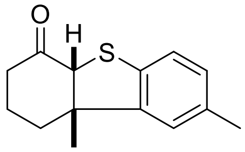
O
H
S

49

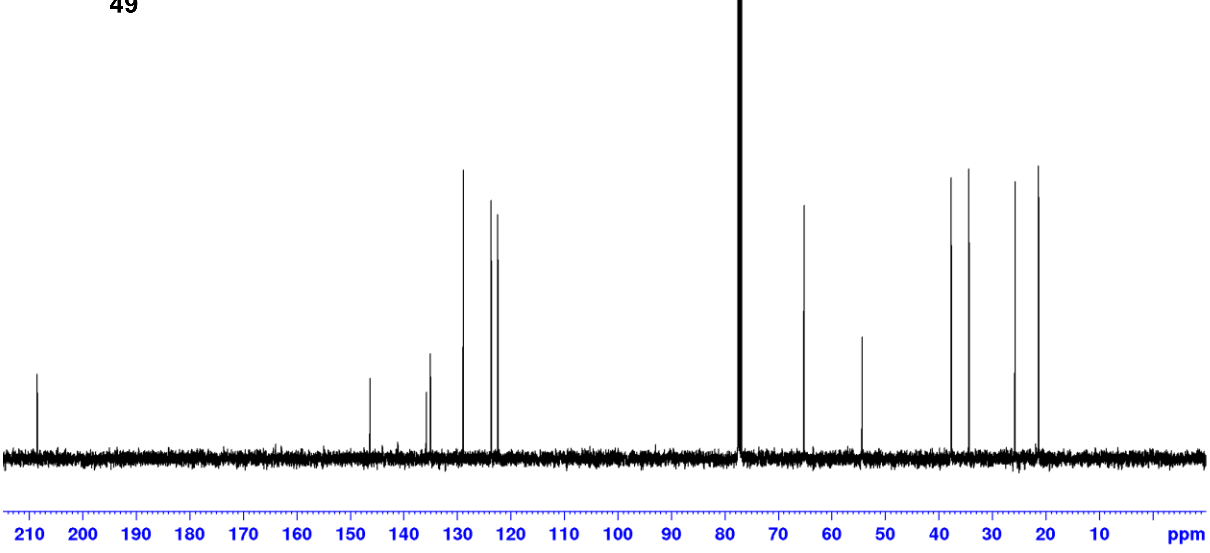
208.52
146.30
135.77
135.00
128.87
123.64
122.39
65.16
54.27
37.64
34.33
25.70
21.33
21.27
210 200 190 180 170 160 150 140 130 120 110 100 90 80 70 60 50 40 30 20 10 ppm

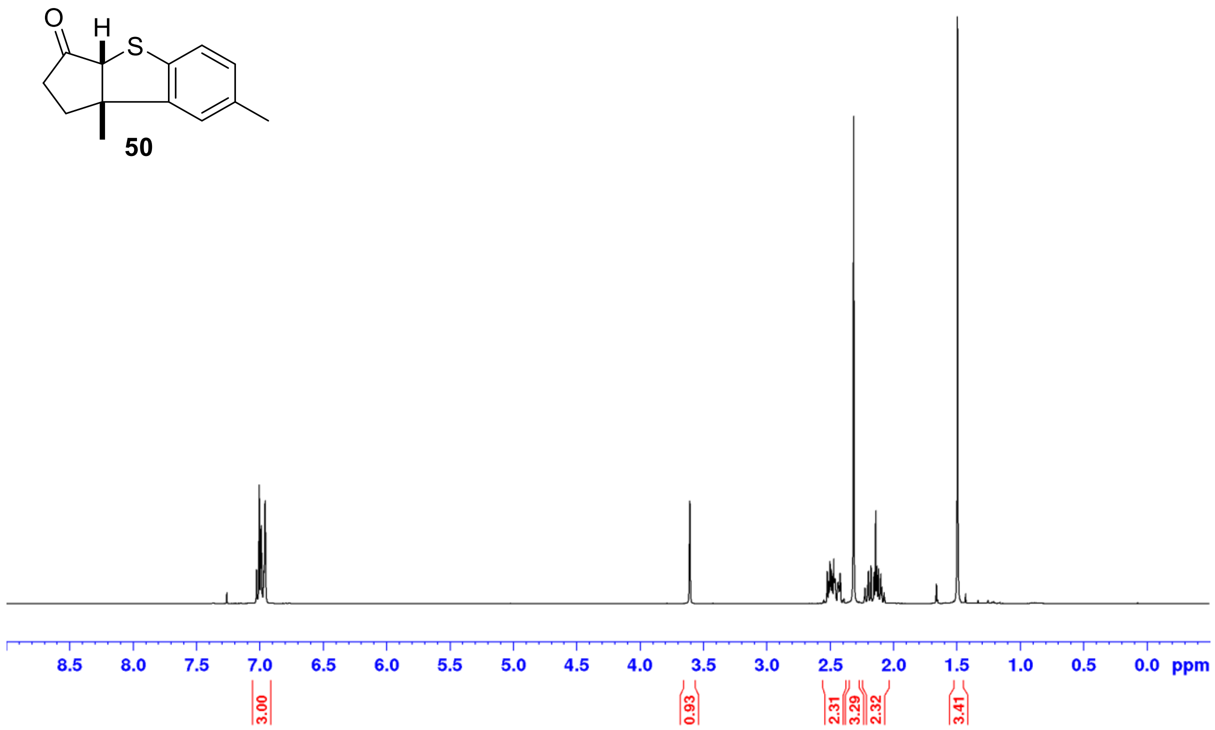
O
H
S
50
8.5
8.0
7.5
7.0
6.5
6.0
5.5
5.0
4.5
4.0
3.5
3.0
2.5
2.0
1.5
1.0
0.5
0.0
ppm
3.00
0.93
2.31
3.29
2.32
3.41

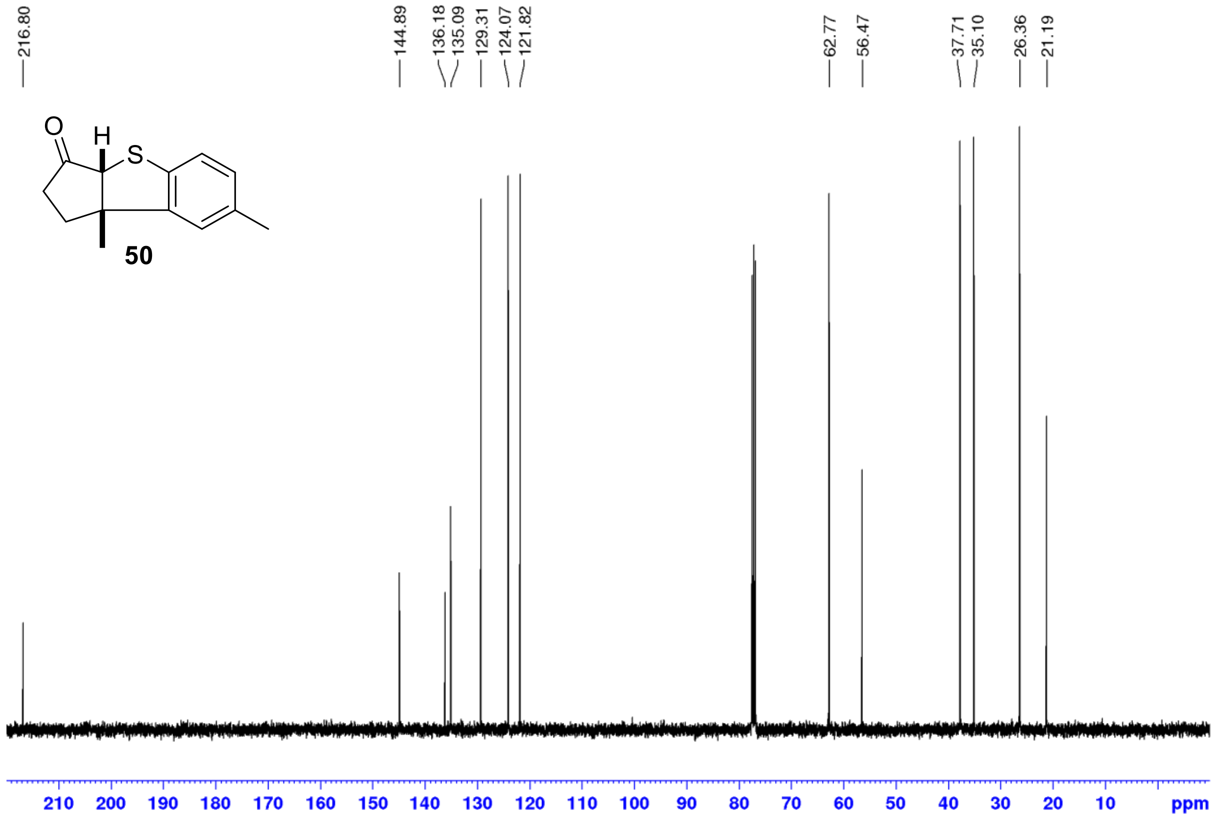
216.80
144.89
136.18
135.09
129.31
124.07
121.82
62.77
56.47
37.71
35.10
26.36
21.19
O
H
S
50
210
200
190
180
170
160
150
140
130
120
110
100
90
80
70
60
50
40
30
20
10
ppm

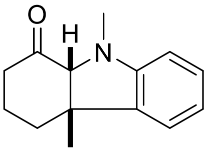

51

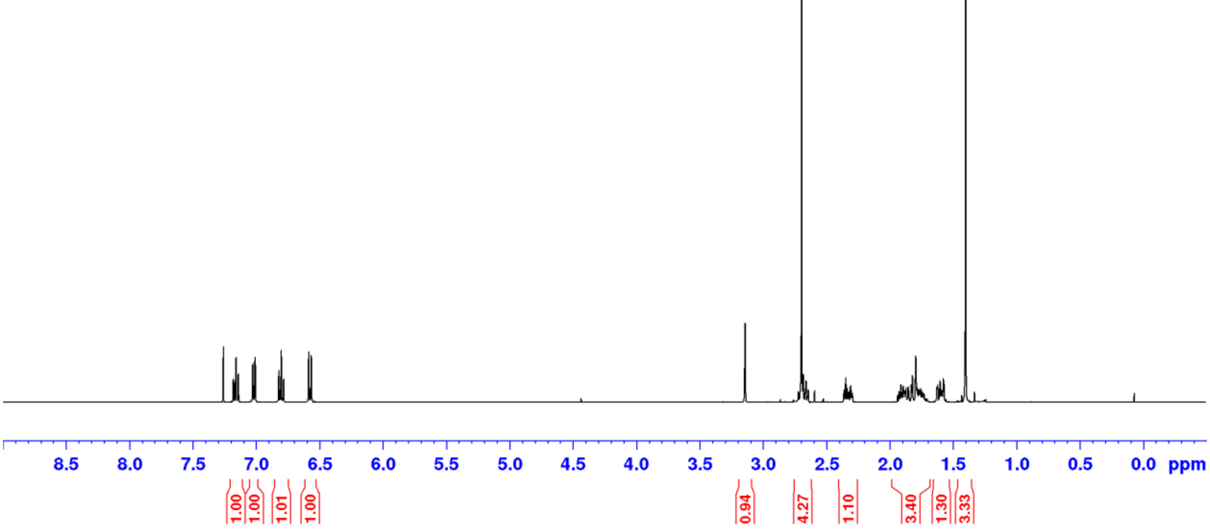
8.5
8.0
7.5
7.0
6.5
6.0
5.5
5.0
4.5
4.0
3.5
3.0
2.5
2.0
1.5
1.0
0.5
0.0
ppm
1.00
1.00
1.01
1.00
0.94
4.27
1.10
3.40
1.30
3.33

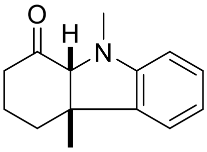

51

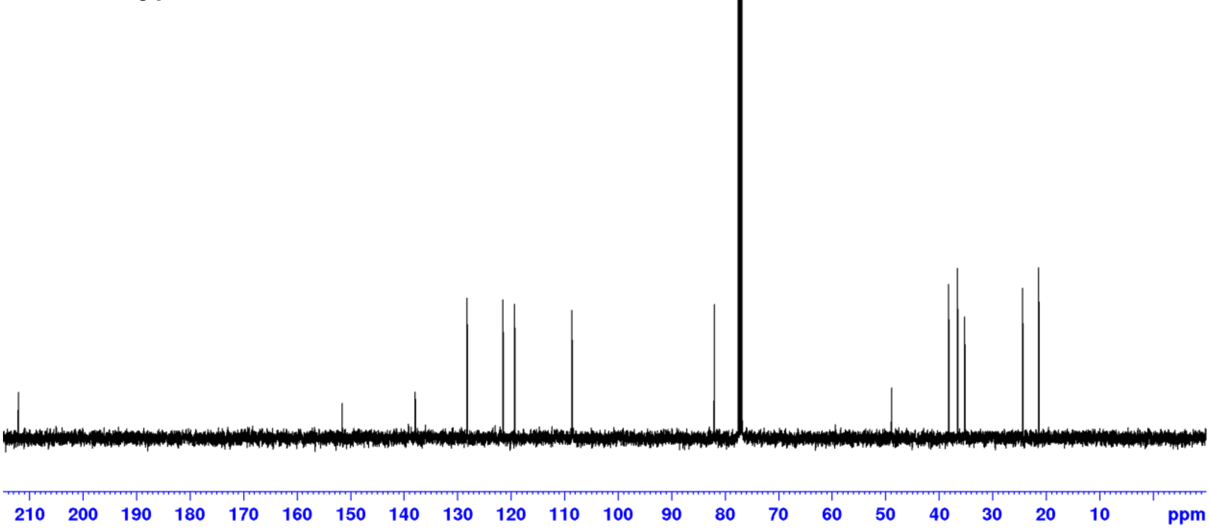
212.11
151.53
137.87
128.17
121.49
119.29
108.57
81.95
48.84
38.15
36.53
35.15
24.35
21.30
210
200
190
180
170
160
150
140
130
120
110
100
90
80
70
60
50
40
30
20
10
ppm

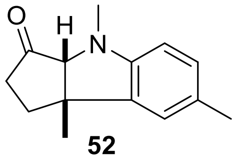

52

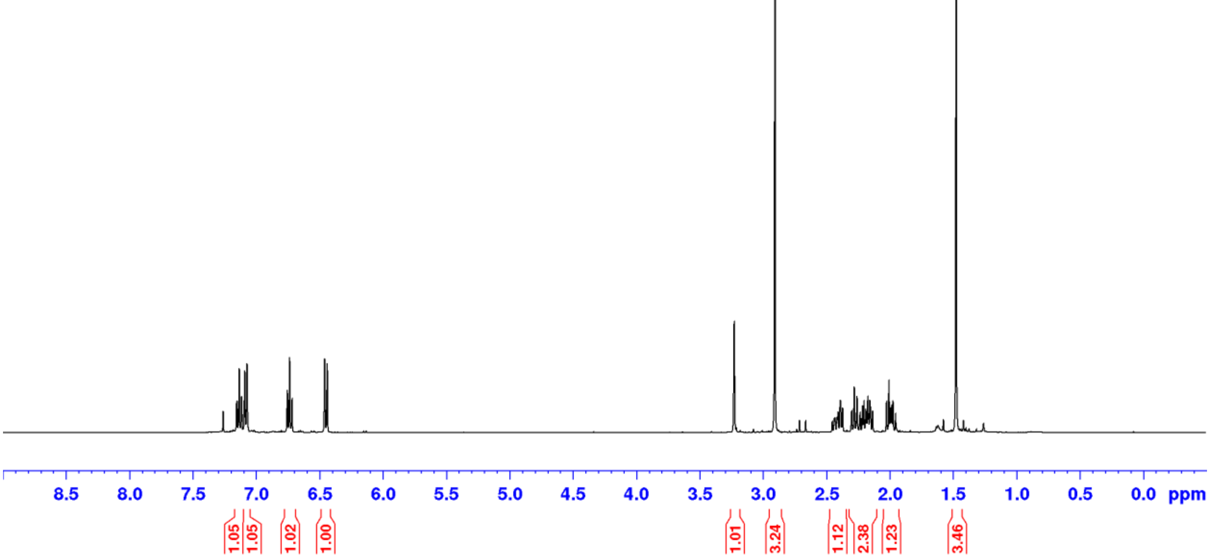
8.5 8.0 7.5 7.0 6.5 6.0 5.5 5.0 4.5 4.0 3.5 3.0 2.5 2.0 1.5 1.0 0.5 0.0 ppm
1.05 1.05 1.02 1.00 1.01 3.24 1.12 2.38 1.23 3.46

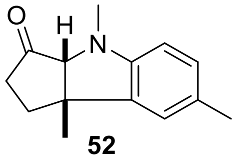

52

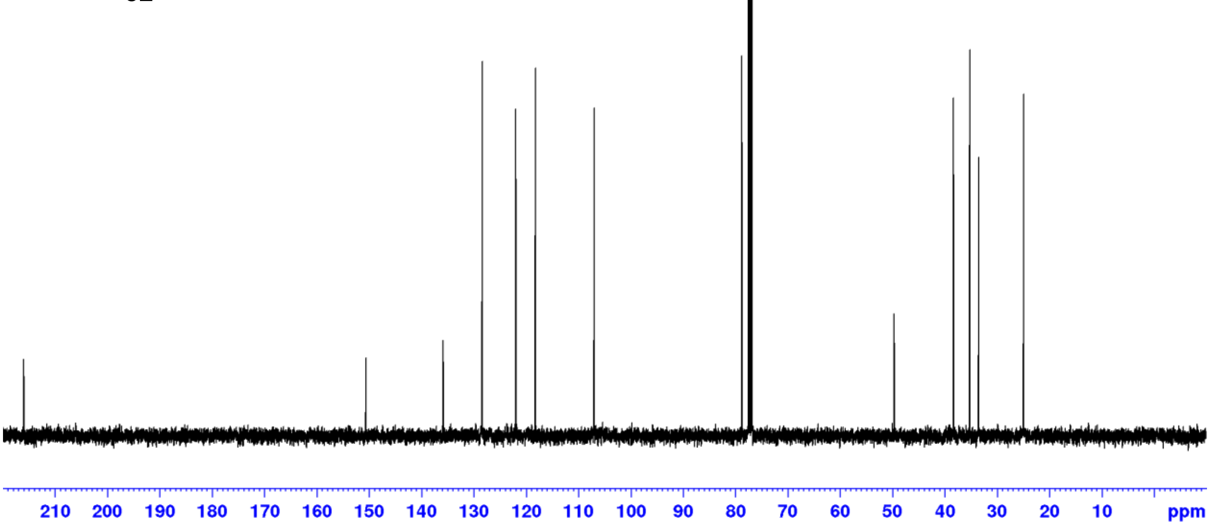
210 200 190 180 170 160 150 140 130 120 110 100 90 80 70 60 50 40 30 20 10 ppm

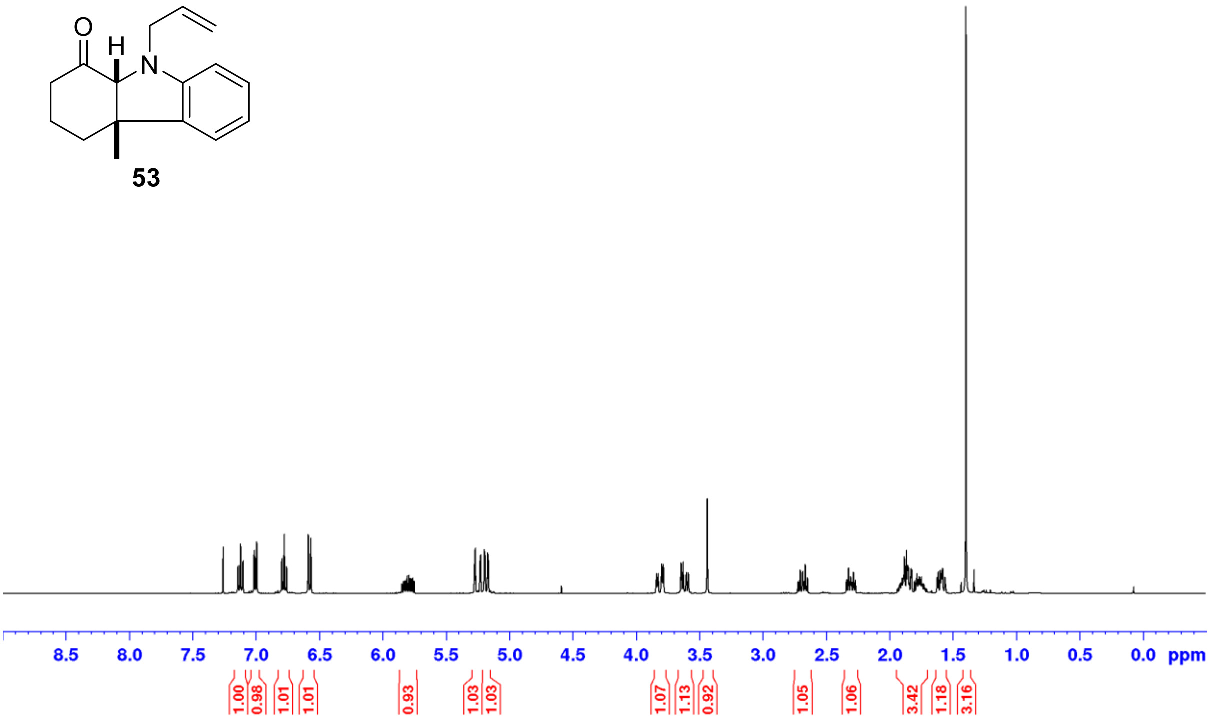
53
8.5
8.0
7.5
7.0
6.5
6.0
5.5
5.0
4.5
4.0
3.5
3.0
2.5
2.0
1.5
1.0
0.5
0.0
ppm
1.00
0.98
1.01
1.01
0.93
1.03
1.03
1.07
1.13
0.92
1.05
1.06
3.42
1.18
3.16

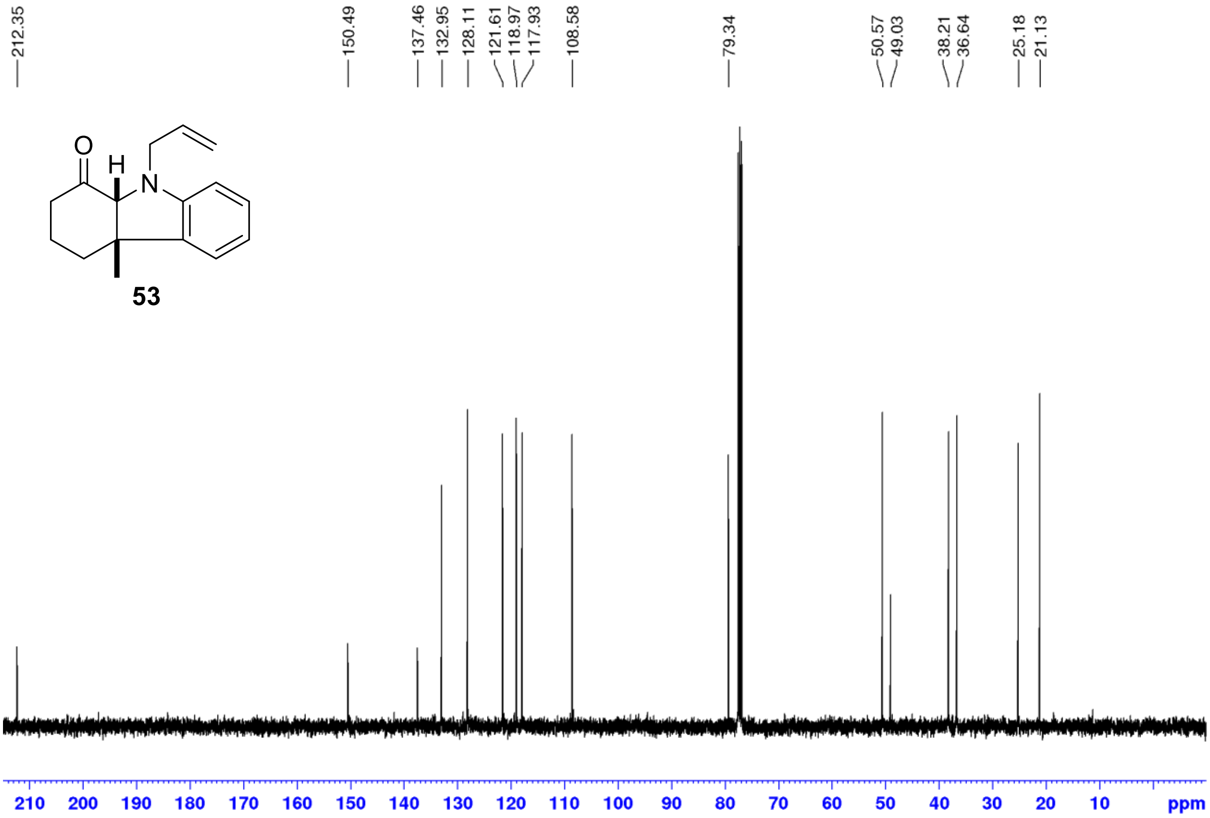
212.35
150.49
137.46
132.95
128.11
121.61
118.97
117.93
108.58
79.34
50.57
49.03
38.21
36.64
25.18
21.13
53
210
200
190
180
170
160
150
140
130
120
110
100
90
80
70
60
50
40
30
20
10
ppm

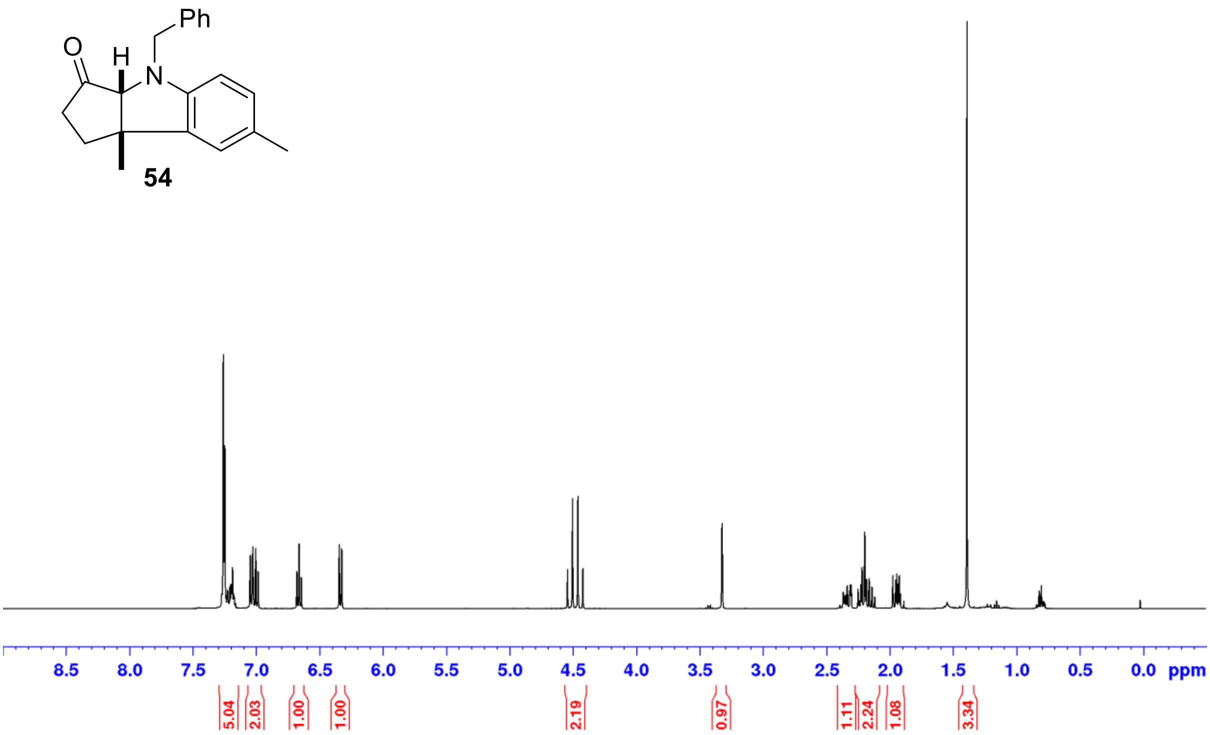
Ph
O
H
N
54
8.5
8.0
7.5
7.0
6.5
6.0
5.5
5.0
4.5
4.0
3.5
3.0
2.5
2.0
1.5
1.0
0.5
0.0
ppm
5.04
2.03
1.00
1.00
2.19
0.97
1.11
2.24
1.08
3.34

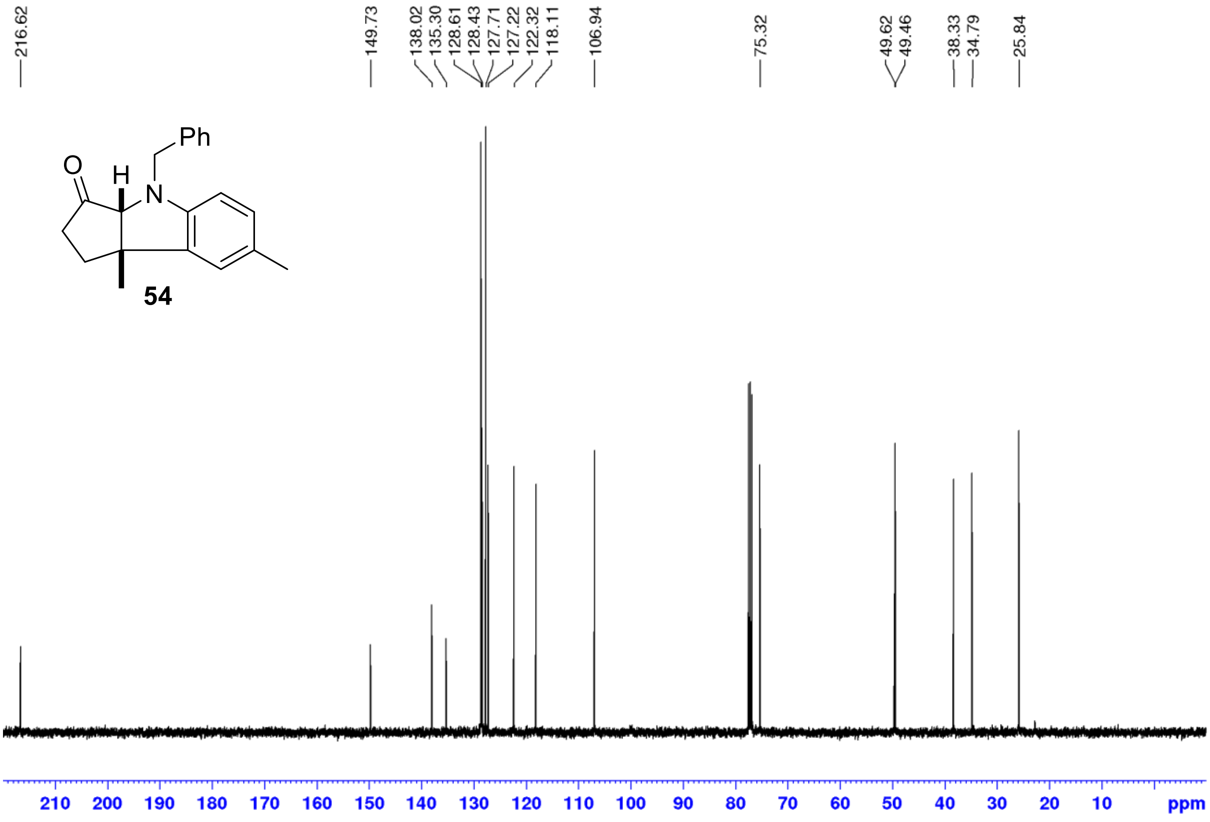
216.62
149.73
138.02
135.30
128.61
128.43
127.71
127.22
122.32
118.11
106.94
75.32
49.62
49.46
38.33
34.79
25.84
Ph
O
H
N
54
210
200
190
180
170
160
150
140
130
120
110
100
90
80
70
60
50
40
30
20
10
ppm

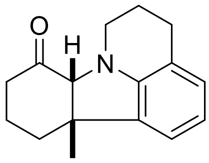

55

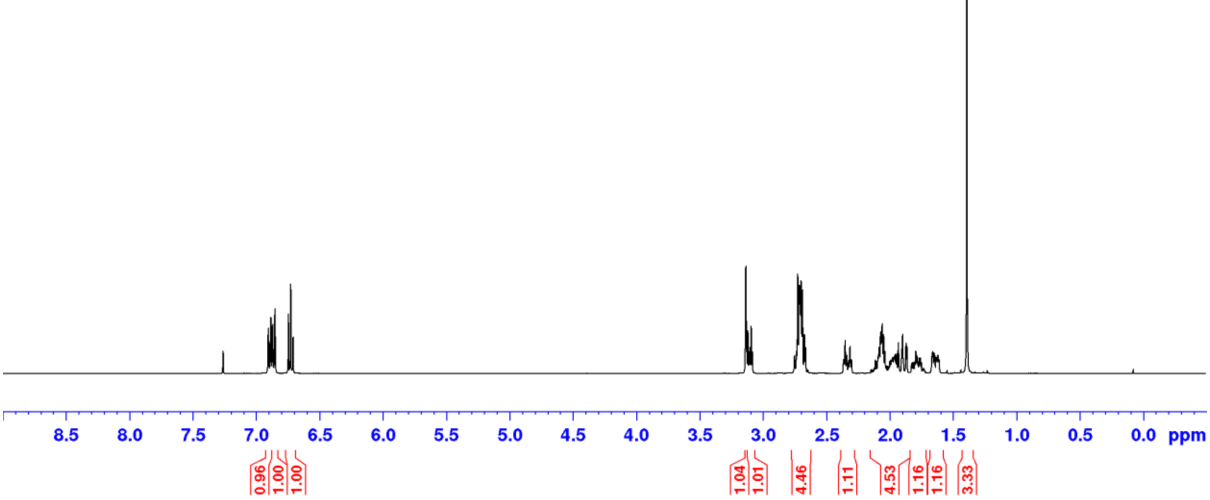
8.5 8.0 7.5 7.0 6.5 6.0 5.5 5.0 4.5 4.0 3.5 3.0 2.5 2.0 1.5 1.0 0.5 0.0 ppm
0.96
1.00
1.00
1.04
1.01
4.46
1.11
4.53
1.16
1.16
3.33

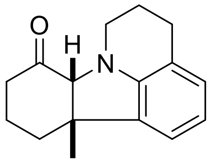

55

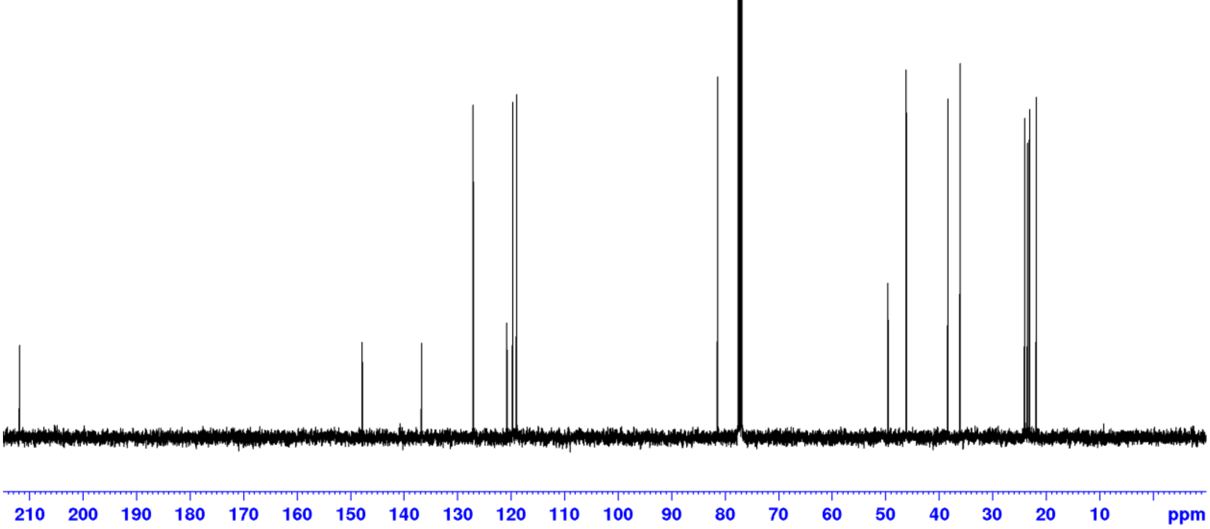
211.87
147.79
136.69
127.05
120.76
119.65
118.95
81.35
49.51
46.08
38.31
36.04
23.92
23.43
23.04
21.76
210 200 190 180 170 160 150 140 130 120 110 100 90 80 70 60 50 40 30 20 10 ppm

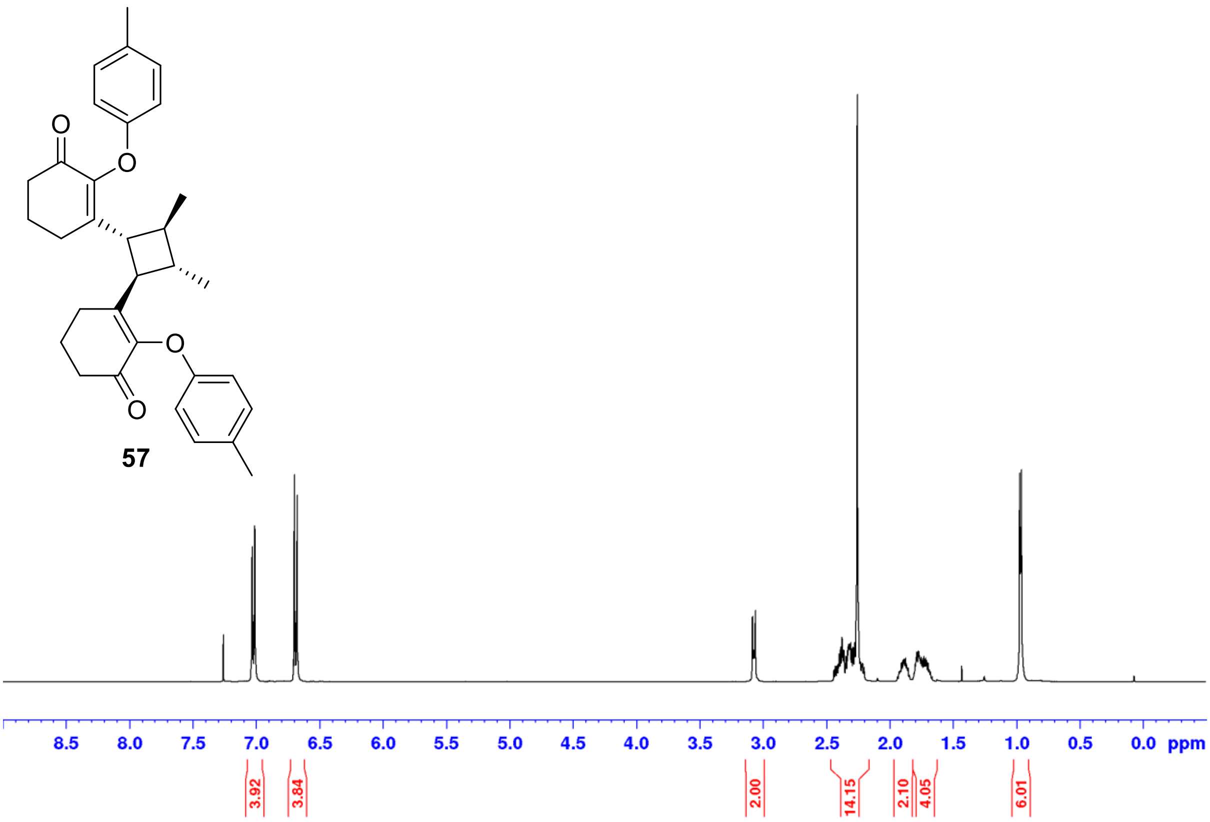
57
8.5 8.0 7.5 7.0 6.5 6.0 5.5 5.0 4.5 4.0 3.5 3.0 2.5 2.0 1.5 1.0 0.5 0.0 ppm
3.92
3.84
2.00
14.15
2.10
4.05
6.01

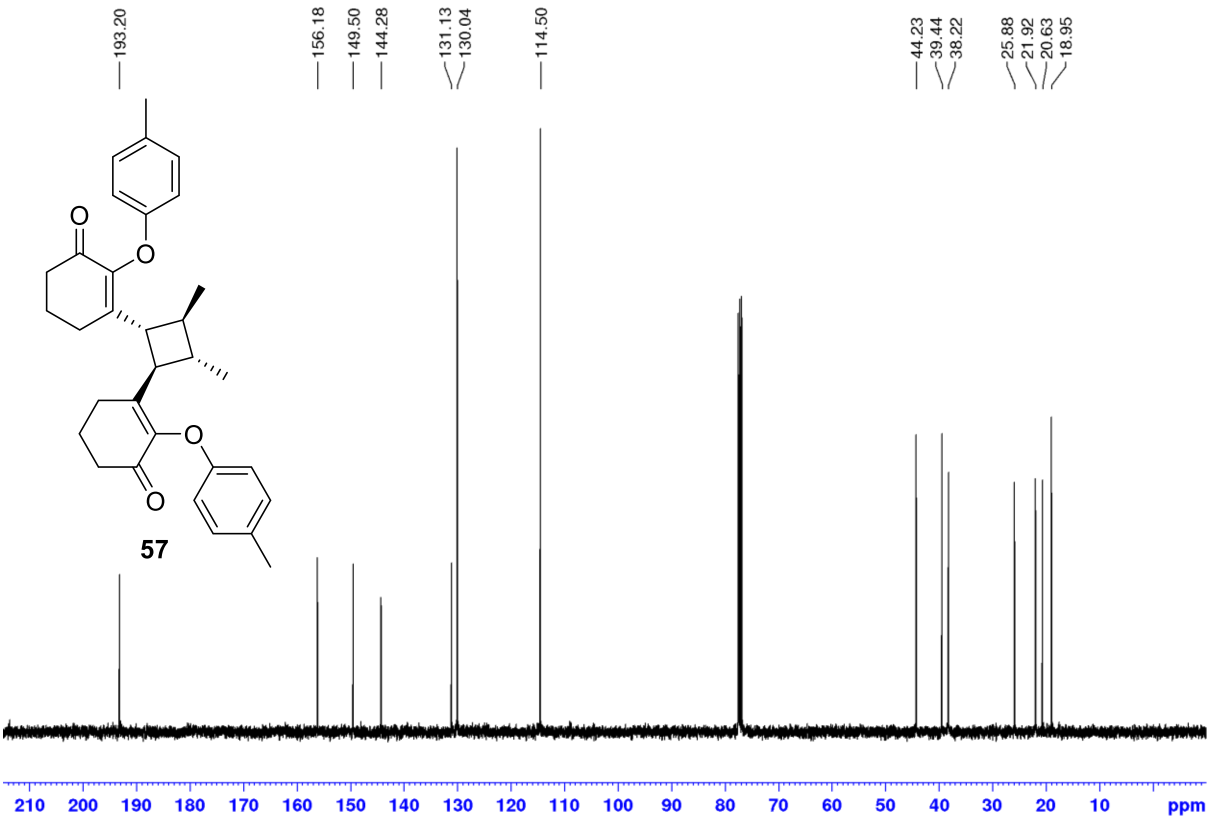
193.20
156.18
149.50
144.28
131.13
130.04
114.50
44.23
39.44
38.22
25.88
21.92
20.63
18.95
57
210 200 190 180 170 160 150 140 130 120 110 100 90 80 70 60 50 40 30 20 10 ppm

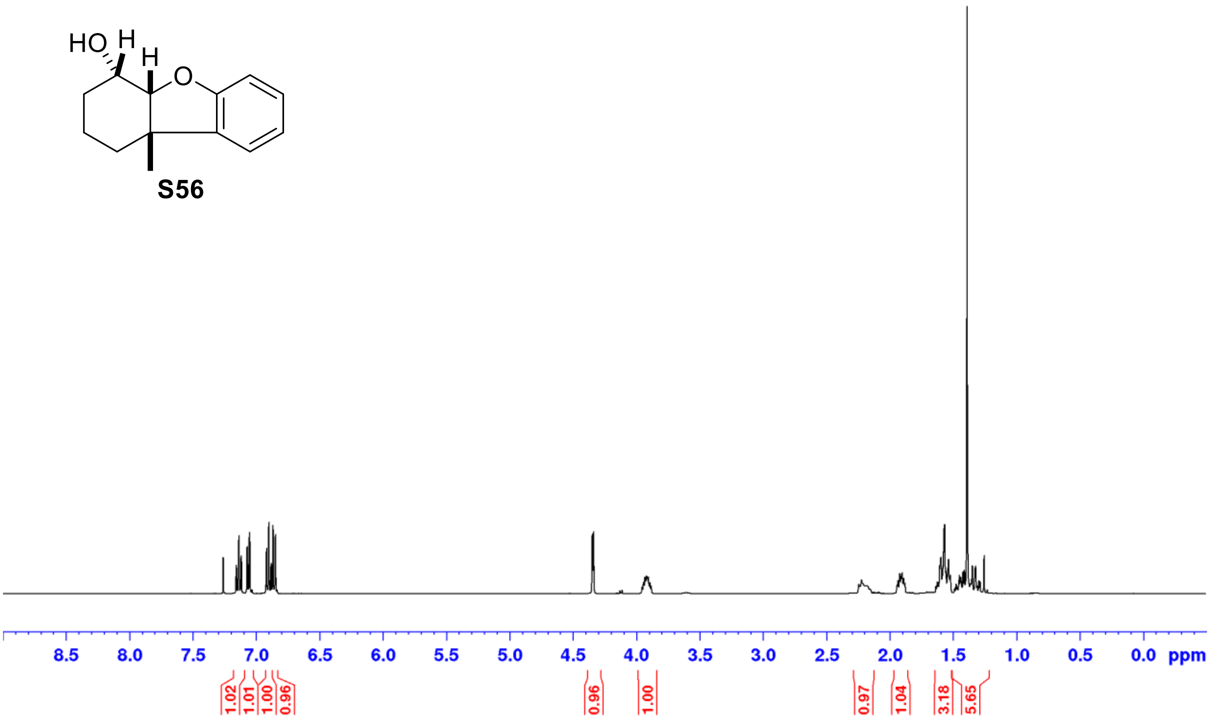
HO
H
H
O
S56
8.5
8.0
7.5
7.0
6.5
6.0
5.5
5.0
4.5
4.0
3.5
3.0
2.5
2.0
1.5
1.0
0.5
0.0
ppm
1.02
1.01
1.00
0.96
0.96
1.00
0.97
1.04
3.18
5.65

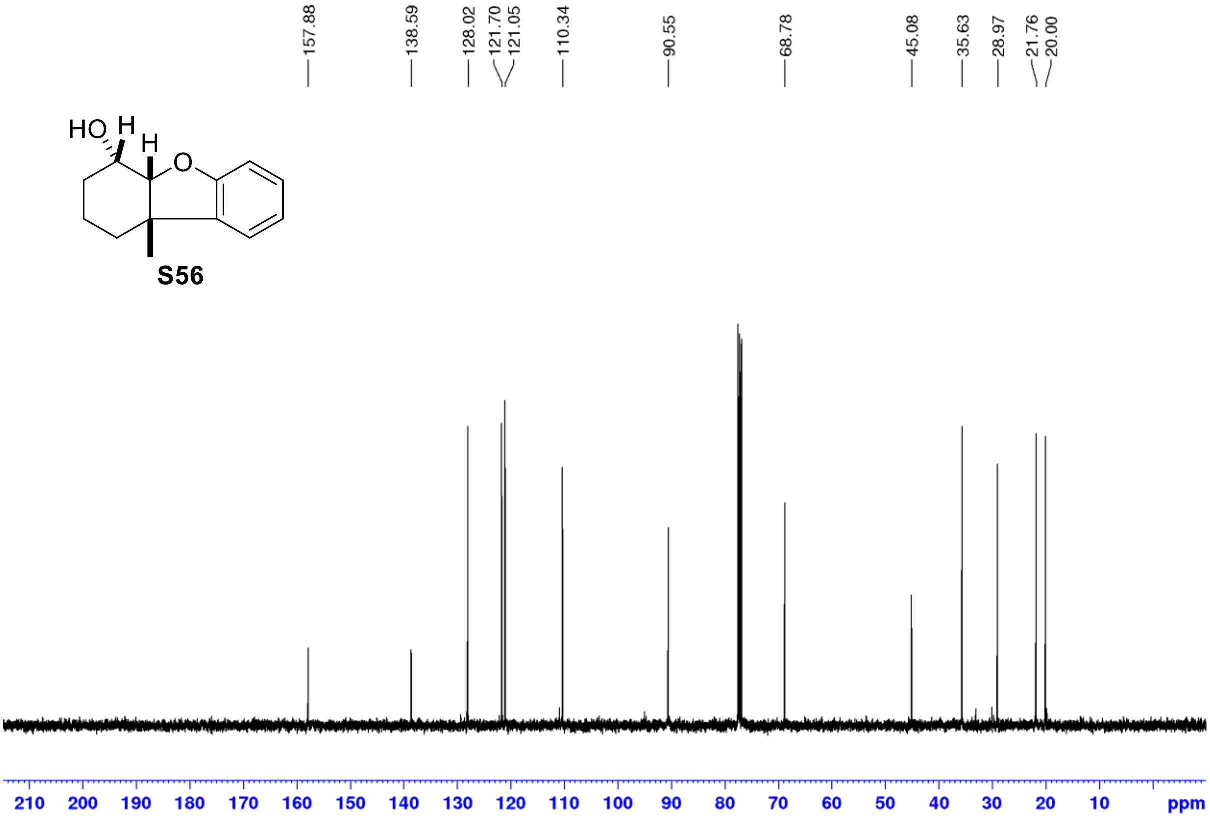
157.88
138.59
128.02
121.70
121.05
110.34
90.55
68.78
45.08
35.63
28.97
21.76
20.00
HO
H
H
O
S56
210
200
190
180
170
160
150
140
130
120
110
100
90
80
70
60
50
40
30
20
10
ppm

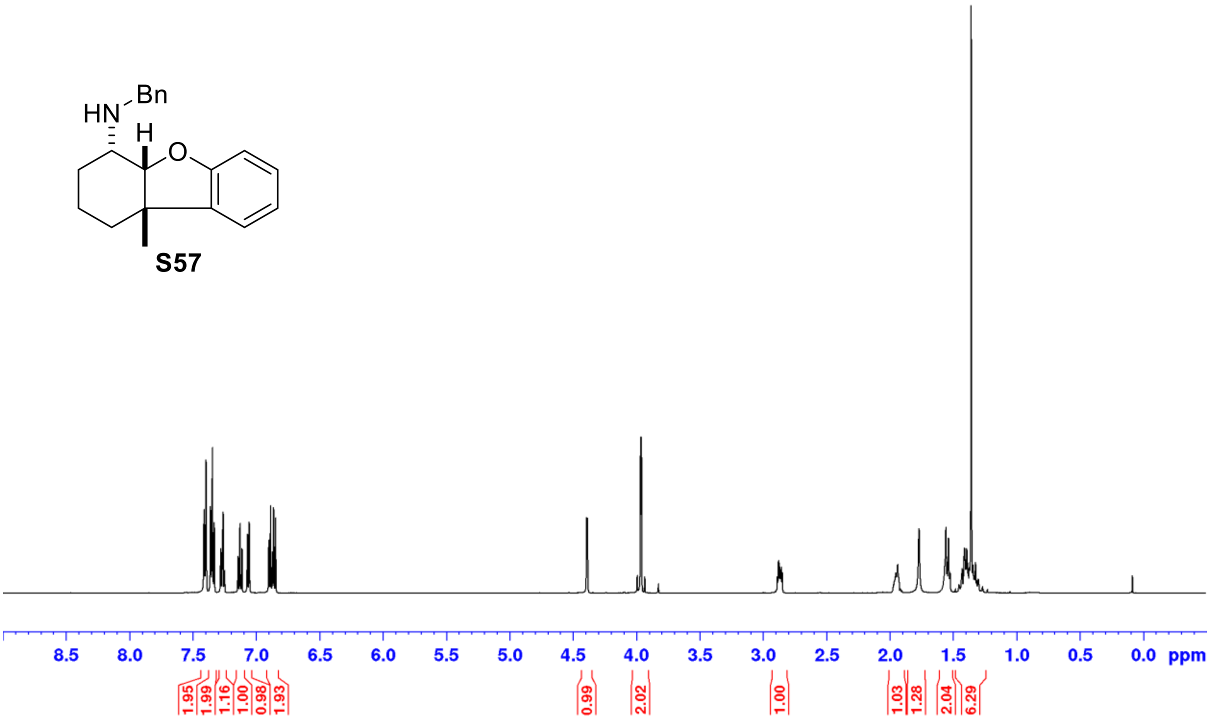
HN
Bn
H
O
S57
8.5 8.0 7.5 7.0 6.5 6.0 5.5 5.0 4.5 4.0 3.5 3.0 2.5 2.0 1.5 1.0 0.5 0.0 ppm
1.95 1.99 1.16 1.00 0.98 1.93 0.99 2.02 1.00 1.03 1.28 2.04 6.29

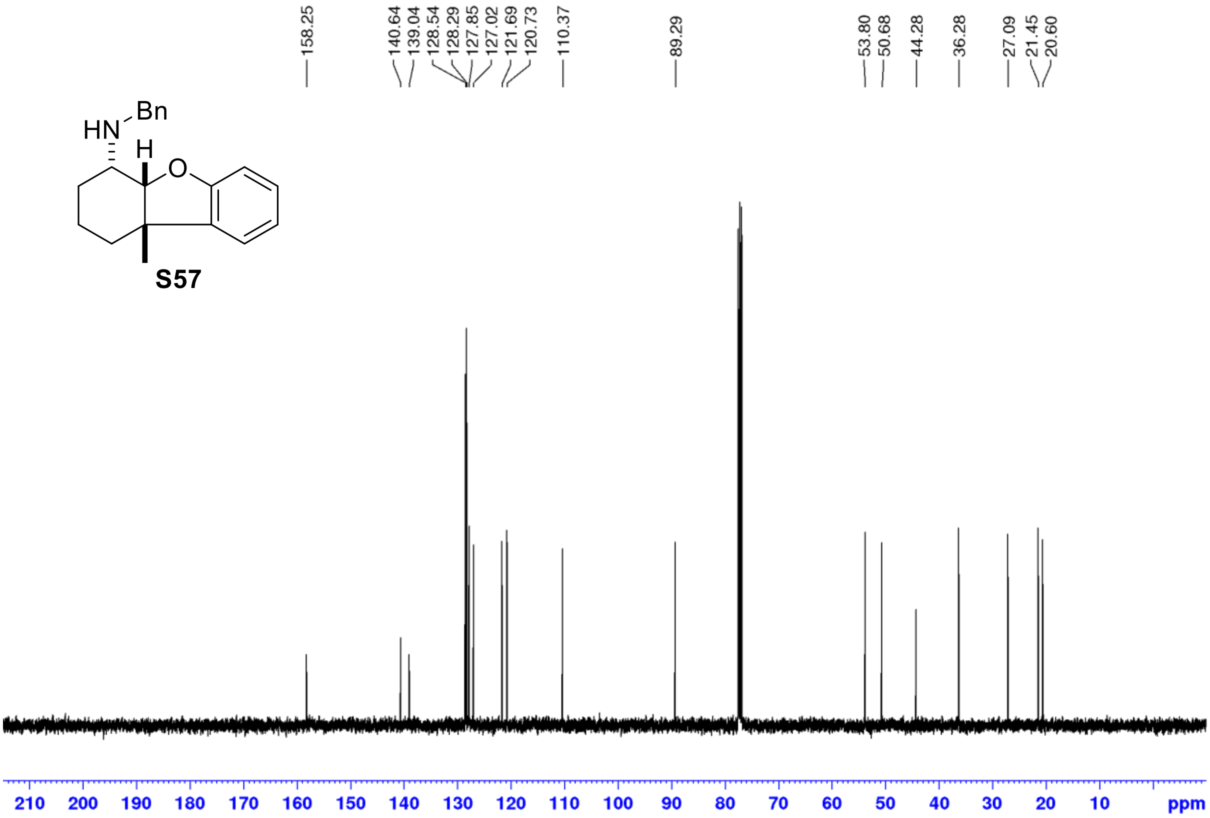
158.25
140.64 139.04 128.54 128.29 127.85 127.02 121.69 120.73
110.37
89.29
53.80 50.68 44.28 36.28 27.09 21.45 20.60
HN
Bn
H
O
S57
210 200 190 180 170 160 150 140 130 120 110 100 90 80 70 60 50 40 30 20 10 ppm

S58

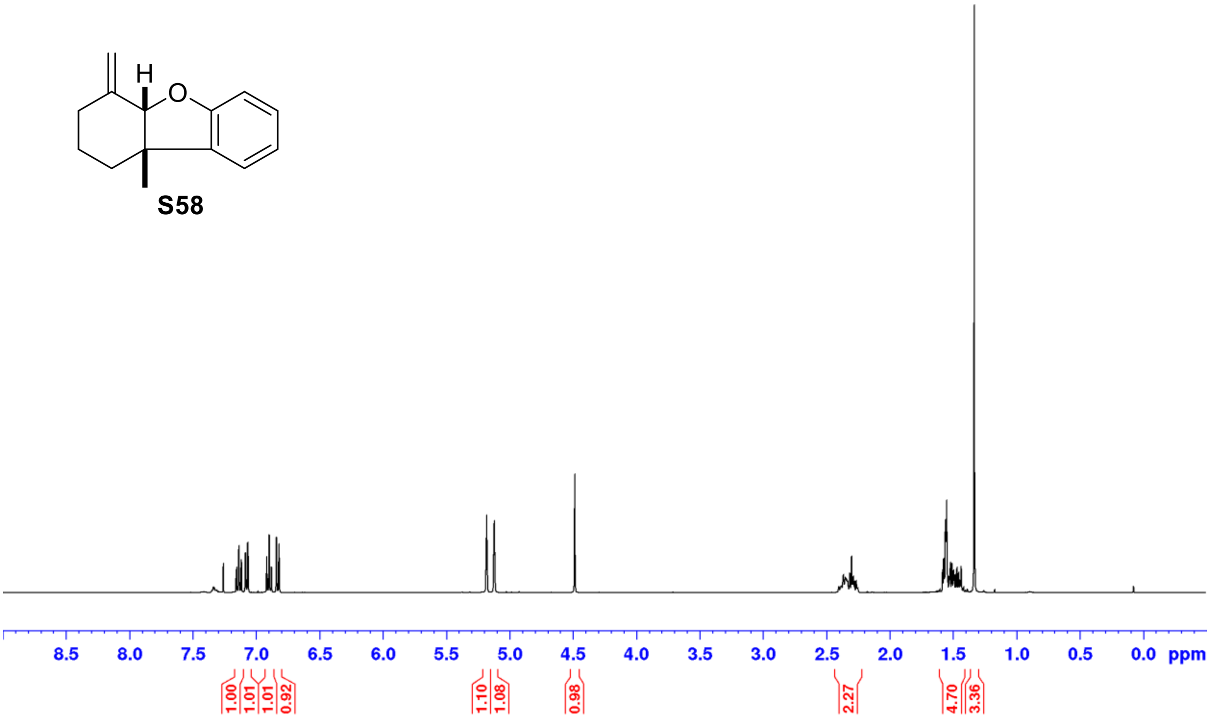
8.5 8.0 7.5 7.0 6.5 6.0 5.5 5.0 4.5 4.0 3.5 3.0 2.5 2.0 1.5 1.0 0.5 0.0 ppm
1.00 1.01 1.01 0.92 1.10 1.08 0.98 2.27 4.70 3.36

S58

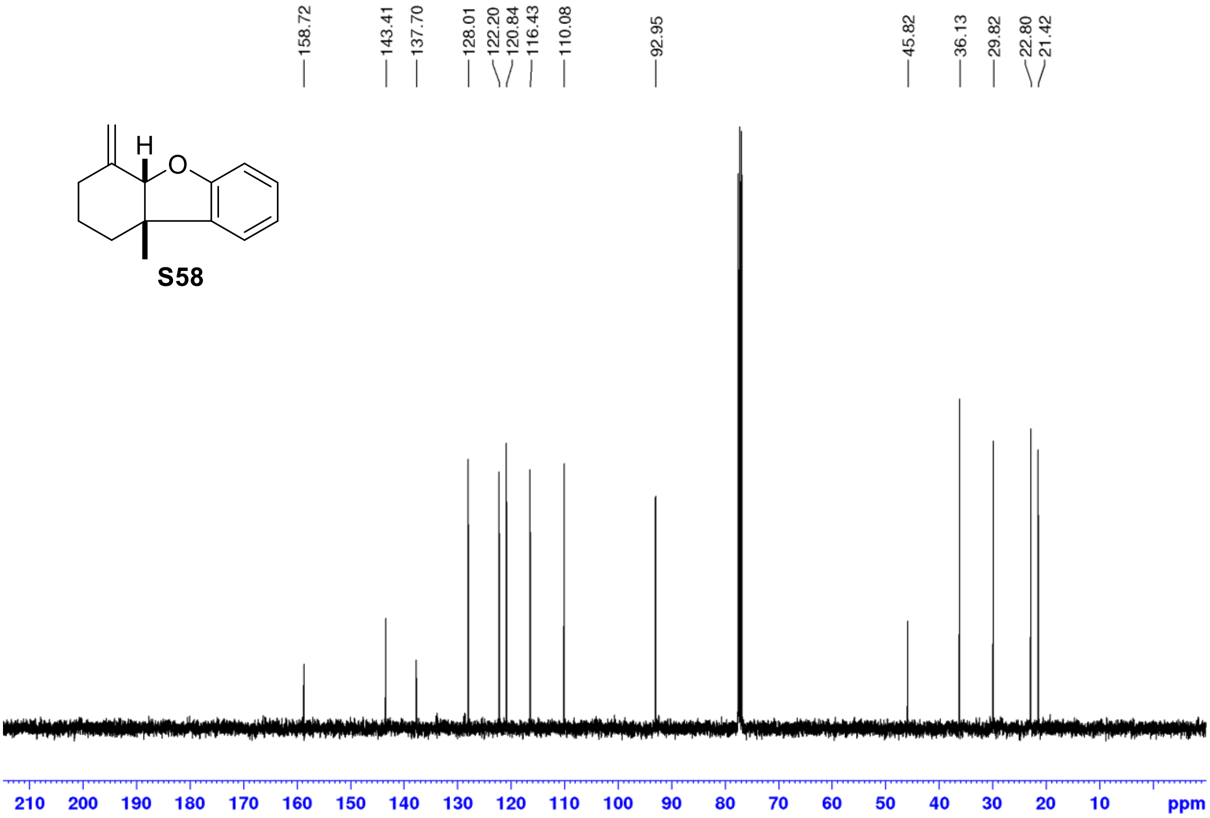
158.72 143.41 137.70 128.01 122.20 120.84 116.43 110.08 92.95 45.82 36.13 29.82 22.80 21.42
210 200 190 180 170 160 150 140 130 120 110 100 90 80 70 60 50 40 30 20 10 ppm

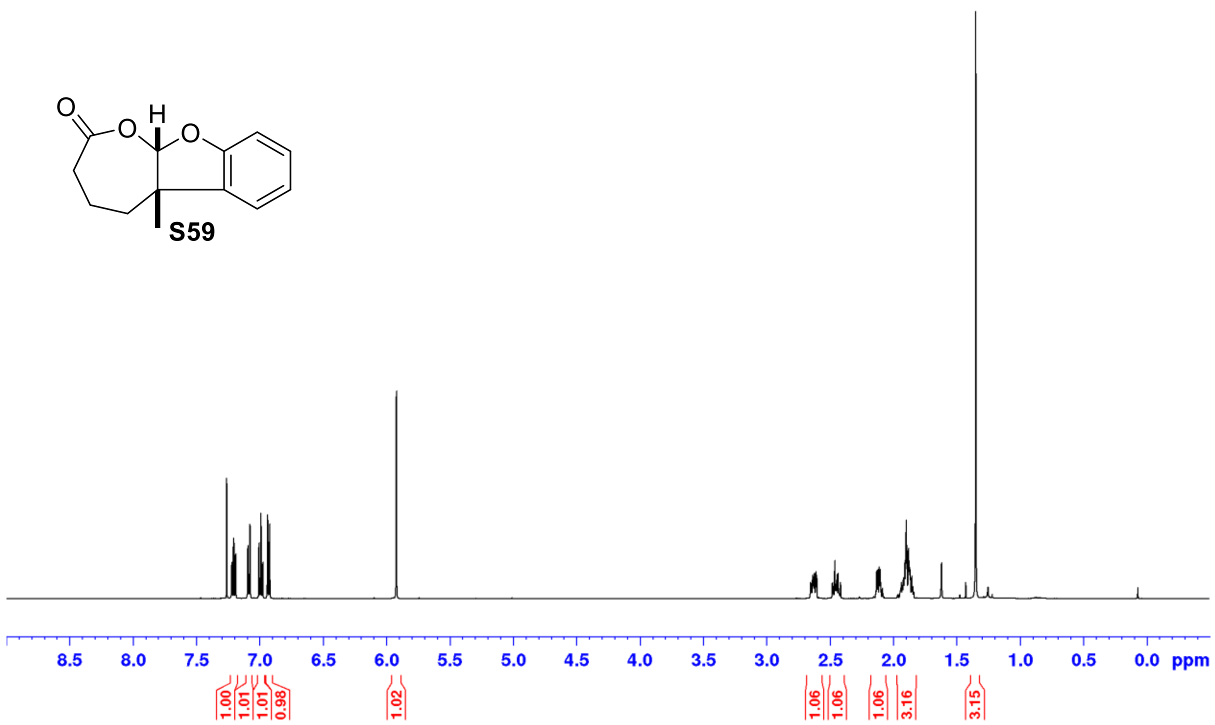

O
O
H
O
S59
8.5
8.0
7.5
7.0
6.5
6.0
5.5
5.0
4.5
4.0
3.5
3.0
2.5
2.0
1.5
1.0
0.5
0.0
ppm
1.00
1.01
1.01
0.98
1.02
1.06
1.06
1.06
3.16
3.15

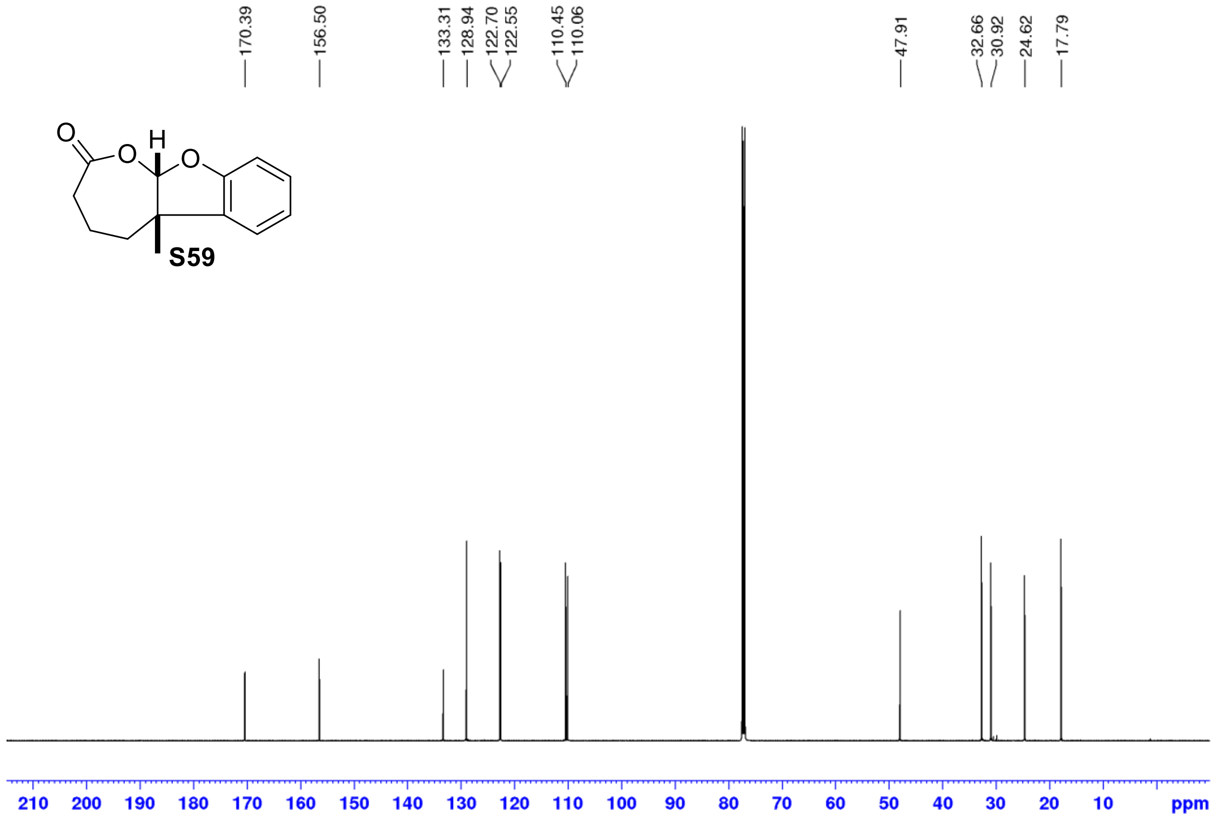

170.39
156.50
133.31
128.94
122.70
122.55
110.45
110.06
47.91
32.66
30.92
24.62
17.79
O
O
H
O
S59
210
200
190
180
170
160
150
140
130
120
110
100
90
80
70
60
50
40
30
20
10
ppm

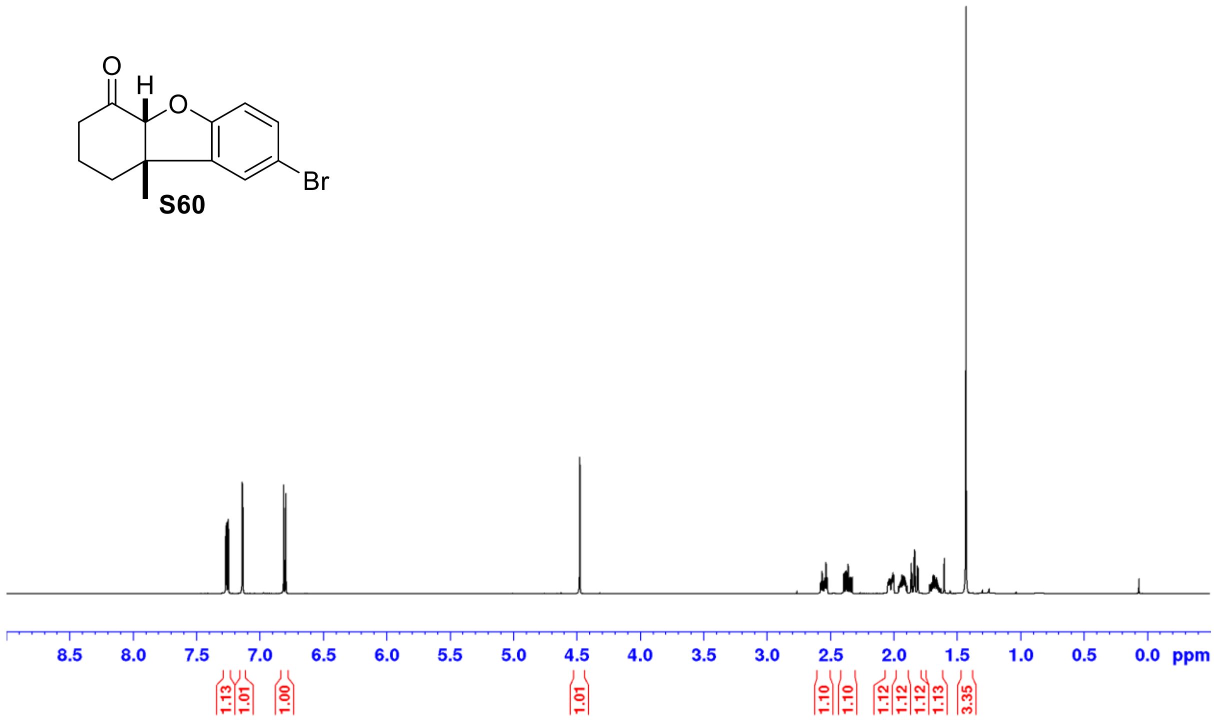
O
H
O
Br
S60
8.5
8.0
7.5
7.0
6.5
6.0
5.5
5.0
4.5
4.0
3.5
3.0
2.5
2.0
1.5
1.0
0.5
0.0
ppm
1.13
1.01
1.00
1.01
1.10
1.10
1.12
1.12
1.12
1.13
3.35

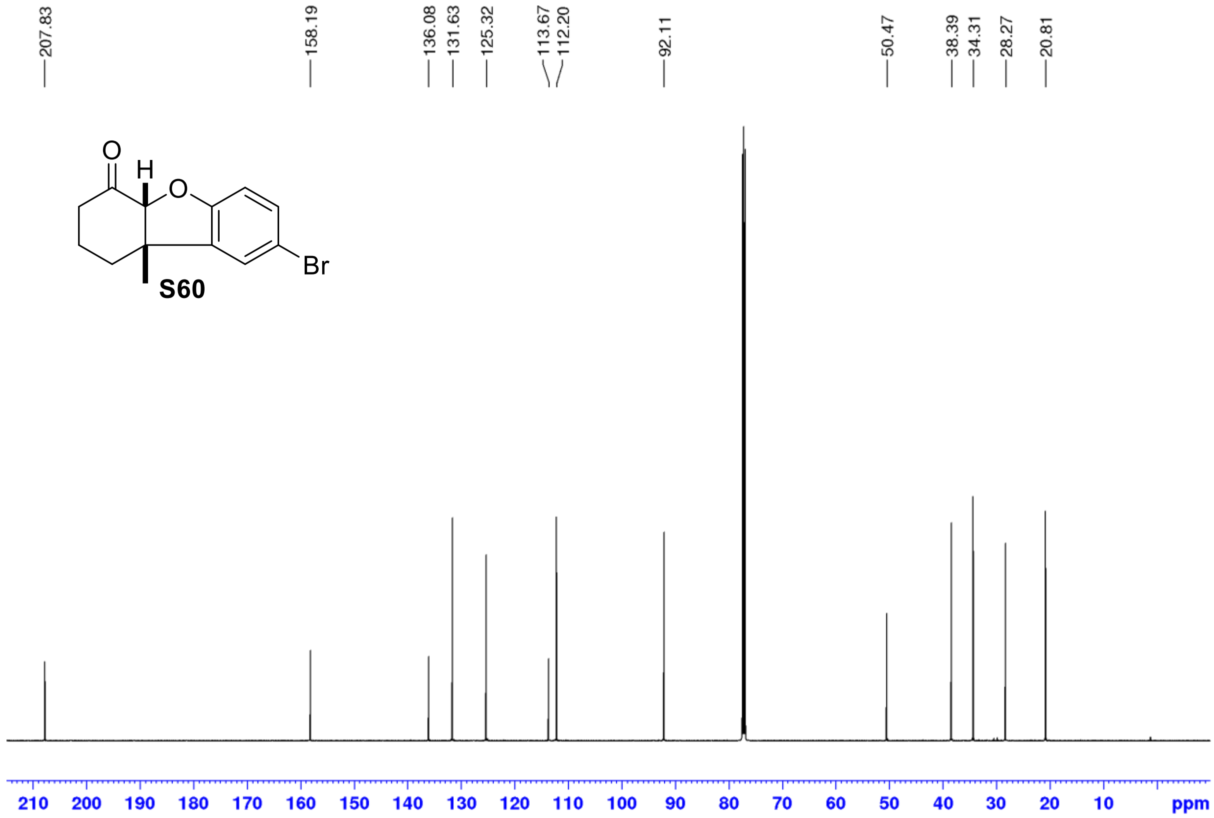
207.83
158.19
136.08
131.63
125.32
113.67
112.20
92.11
50.47
38.39
34.31
28.27
20.81
O
H
O
Br
S60
210
200
190
180
170
160
150
140
130
120
110
100
90
80
70
60
50
40
30
20
10
ppm
